# ﻿Four new endophytic species of *Diaporthe* (Diaporthaceae, Diaporthales) isolated from Cameroon

**DOI:** 10.3897/mycokeys.99.110043

**Published:** 2023-10-23

**Authors:** Christopher Lambert, Lena Schweizer, Blondelle Matio Kemkuignou, Elodie Gisèle M. Anoumedem, Simeon F. Kouam, Yasmina Marin-Felix

**Affiliations:** 1 Department of Microbial Drugs, Helmholtz Centre for Infection Research (HZI) and German Centre for Infection Research (DZIF), Partner Site Hannover/Braunschweig, Inhoffenstrasse 7, 38124 Braunschweig, Germany Department of Microbial Drugs, Helmholtz Centre for Infection Research (HZI) and German Centre for Infection Research (DZIF), Partner Site Hannover/Braunschweig Braunschweig Germany; 2 Institute of Microbiology, Technische Universität Braunschweig, Spielmannstraße 7, 38106 Braunschweig, Germany Technische Universität Braunschweig Braunschweig Germany; 3 Molecular Cell Biology Group, Helmholtz Centre for Infection Research (HZI), Inhoffenstrasse 7, 38124 Braunschweig, Germany Molecular Cell Biology Group, Helmholtz Centre for Infection Research (HZI) Braunschweig Germany; 4 Department of Chemistry, Higher Teacher Training College, University of Yaoundé I, Yaoundé P.O. Box 47, Cameroon University of Yaoundé I Yaounde Cameroon

**Keywords:** Endophytes, *Phomopsis*, Sordariomycetes, 4 new taxa

## Abstract

The genus *Diaporthe* (Diaporthaceae, Diaporthales) is a large group of fungi frequently reported as phytopathogens, with ubiquitous distribution across the globe. *Diaporthe* have traditionally been characterized by the morphology of their ana- and teleomorphic state, revealing a high degree of heterogeneity as soon as DNA sequencing was utilized across the different members of the group. Their relevance for biotechnology and agriculture attracts the attention of taxonomists and natural product chemists alike in context of plant protection and exploitation for their potential to produce bioactive secondary metabolites. While more than 1000 species are described to date, Africa, as a natural habitat, has so far been under-sampled. Several endophytic fungi belonging to *Diaporthe* were isolated from different plant hosts in Cameroon over the course of this study. Phylogenetic analyses based on DNA sequence data of the internal transcribed spacer region and intervening 5.8S nrRNA gene, and partial fragments of the calmodulin, beta-tubulin, histone and the translation elongation factor 1-α genes, demonstrated that these isolates represent four new species, i.e. *D.brideliae*, *D.cameroonensis*, *D.pseudoanacardii* and *D.rauvolfiae*. Moreover, the description of *D.isoberliniae* is here emended, now incorporating the morphology of beta and gamma conidia produced by two of our endophytic isolates, which had never been documented in previous records. Moreover, the paraphyletic nature of the genus is discussed and suggestions are made for future revision of the genus.

## ﻿Introduction

The genus *Diaporthe* (Diaporthaceae, Diaporthales, Sordariomycetes) is a group of fungi attracting considerable interest for its occurrence as plant pathogens, endophytes and saprobes, and its biotechnological potential as producers of secondary metabolites (Udayanga et al. 2011; Gomes et al. 2013; Chepkirui and Stadler 2017; Marin-Felix et al. 2019). Among plant diseases and disease symptoms causally linked to *Diaporthe* infections are leaf spots, cankers, dieback and fruit rots as well as decays and wilt (Thompson et al. 2011; Udayanga et al. 2011; Guarnaccia and Crous 2017; Guarnaccia et al. 2018). Historically, *Diaporthe* included teleomorphic species that produced ostiolate ascomata usually immersed in the substrate and often erumpent through a pseudostroma, unitunicate and clavate to cylindrical asci, and hyaline, fusioid, ellipsoid to cylindrical, septate ascospores, sometimes with appendages. On the other hand, the corresponding anamorphs were accommodated within the genus *Phomopsis*, which was characterized by ostiolate conidiomata and phialidic conidiogenous cells that may produce three types of hyaline conidia, *i.e.* alpha, beta and gamma (Udayanga et al. 2011). Synonymization following the one-fungus-one name paradigm linked both individual groups together, with the older name *Diaporthe* recieving priority over *Phomopsis* (Rossman et al. 2015). Taxonomical classification of *Diaporthe* relies on its host specificity, disease symptoms and morphological features such as that of ascomata, stroma and spore shapes (Udayanga et al. 2011, Gomes et al. 2013). Nowadays, morphological and ecological traits were shown to exhibit high degrees of homoplasy, as molecular phylogenetic studies over the years demonstrated – a common feature found in rising numbers of fungal groups (Gomes et al. 2013; Jaklitsch et al. 2016). In consequence, recent taxonomical surveys extend to multilocus sequencing, namely ITS, *cal*, *his3*, *tef1 and tub2*, and employ molecular phylogenetic concatenation-based methods for species description and delimitation (Udayanga et al. 2012a). However, most of the over 1000 records are not sequenced (213 species validated by sequence data and typification in Marin-Felix et al. 2019; 293 species and type strains surveyed by Norphanphoun et al. 2022), hence for the future of this genus, it will be critical to recollect and typify old records to bring an expectable high amount of synonyms together. This is the only option to long-term stabilize the taxonomy of *Diaporthe* (Dissanayake 2017).

Besides rarely occurring infections in immunocompromised human individuals, members of the genus *Diaporthe* are most well-known as phytopathogens in agriculture (Iriart et al. 2011; Rakita et al. 2017; Marin-Felix et al. 2019). Among the most economically impactful, infections of grapevines, forest trees and plants of ornamental value have to be named, with *D.eres* and *D.ampelina*, and more recently *D.rudis* from apple trees (Martino et al. 2023) being among the most frequently isolated ones in Europe (Pscheidt 1989; Mostert et al. 2001; Guarnaccia and Crous 2017; Guarnaccia et al. 2018; Yang et al. 2018; Manawasinghe et al. 2019). During pathogenesis, secondary metabolites were occasionally described as important virulence factors, ensuring plant infection (Tsurushima et al. 2005). This and their ubiquitous dispersion are conceivably among the main reasons why *Diaporthe* and the former *Phomopsis* spp. have been studied extensively for their capability to produce bioactive natural products (Chepkirui and Stadler 2017; Xu et al. 2021). For instance, Goddard et al. 2014 described the isolation of nine *Diaporthe* strains (described *as Phomopsis* spp.) from different vine cultivars with and without showing symptoms of esca decline, a plant trunk disease leading to diebacks of vineyards (Mostert et al. 2001). A set of secondary metabolites was subsequently isolated from cultures growing on petri dishes containing potato dextrose agar and tested for their phytotoxicity. Two compounds, namely cytosporone B and phomopsolide B, induced necrosis on leaf discs similar to eutypine, a phytotoxic polyketide from *Eutypalata*, another noteworthy threat for grape plants (Tey-Rulh et al. 1991). Occurrence in inflorescence and crude sap of infected plants even enabled discerning healthy from infected individuals due to the latter containing eutypine, instrumentalizing the association of fungal secondary metabolites with plant infections for phytopathological surveillance (Mahoney et al. 2005). However, toxin productive capabilities of *Diaporthe* spp. and esca disease symptoms were shown to not strictly correlate with each other (Goddard et al. 2014). Exploring the ecological impact of the more than 300 to-date described natural products will be an important parameter to study the phytopathogenesis of this group of fungi, as has been highlighted by other authors (Pusztahelyi et al. 2015; Chepkirui and Stadler 2017; Xu et al. 2021).

Further embarking on charting the biodiversity of this genus for biotechnological exploitation, we here aimed to describe species diversity in an almost unstudied habitat, the planta from Cameroon. This paper describes the isolation, morphological and molecular characterization of fungal endophytes that were assigned to the genus *Diaporthe*.

## ﻿Material and methods

### ﻿Taxonomy

Hyphal material (1 mm diam) was scratched from actively growing cultures on YM 6.3 agar (malt extract 10 g/L, yeast extract 4 g/L, D-glucose 4 g/L, agar 20 g/L, pH 6.3 before autoclaving) and transferred onto 9-cm-diam petri dishes containing 2% tap water agar supplemented with sterile pine needles (PNA) (Smith et al. 1996), potato dextrose agar (PDA), oatmeal agar (OA) and malt extract agar (MEA) (Crous et al. 2009). The plates were incubated at 21 °C in darkness. Pigment production and colony characters on PDA, OA and MEA were documented after 15 d. Colony colors were rated with the color chart of The Royal Horticultural Society London (1966). Colony diameters were measured after 5, 10 and 15 d. Cultures were examined periodically for development of ascomata and conidiomata. Morphological characters were examined by mounting fungal structures in clear lactic acid and 30 measurements at x1000 magnification were recorded for each isolate using a Nikon eclipse Ni-U (Nikon Europe BV, Amsterdam, Netherlands) microscope with differential interference contrast. Descriptions, nomenclature and illustrations of taxonomic novelties were deposited in MycoBank (www.MycoBank.org).

### ﻿DNA extraction, PCR amplification and sequencing

Genomic DNA was extracted using the EZ-10 SPIN column fungal genomic DNA minipreps Kit (Bio Basic Inc. Ontario, Canada) following manufacturer’s instructions. Six different loci were amplified, *i.e.* the internal transcribed spacer region (ITS), and fragments of the calmodulin (*cal*), histone 3 (*his3*), translation elongation factor 1-α (tef1) and beta-tubulin (*tub2*) genes. The ITS was amplified and sequenced using the primers ITS4 and ITS5 (White et al. 1990), *cal* with CAL-228 F and CAL-737R (Carbone and Kohn 1999), *his3* with CYCH3F and H3-1b (Crous et al. 2004; Glass and Donaldson 1995), *tef1* with EF-1-728F and EF-1-986R (Carbone and Kohn 1999) and *tub2* with Bt2a and Bt2b (Glass and Donaldson 1995). Amplicons were purified by using an EZ-10 spin column PCR purification Kit (Bio Basic Inc. Ontario, Canada) following the manufacturer’s instructions, and sequenced by employing Sanger sequencing with a commercial provider (Microsynth Seqlab GmbH, Göttingen). Consensus sequences were obtained using Geneious 7.1.9 (http://www.geneious.com, Kearse et al. 2012) and deposited in GenBank (accession numbers in Table 1).

**Table 1.** Isolates and reference strains of *Diaporthe* spp. included in the phylogenetic study. GenBank accession numbers in **bold** were newly generated in this study. Taxonomic novelties are indicated in ***bold italic***.

Species	Isolates^1^	GenBank accession numbers^2^					References
		*ITS*	*tub2*	*his3*	*tef1*	*cal*	
*Diaportheacaciarum*	CBS 138862^T^	KP004460	KP004509	KP004504	–	–	Crous et al. (2014b)
*D.acaciigena*	CBS 129521^T^	KC343005	KC343973	KC343489	KC343731	KC343247	Gomes et al. (2013)
*D.acericola*	MFLUCC 17-0956^T^	KY964224	KY964074	–	KY964180	KY964137	Dissanayake et al. (2017a)
*D.acerigena*	CFCC 52554^T^	MH121489	–	MH121449	MH121531	MH121413	Yang et al. (2018)
*D.acerina*	CBS 137.27	KC343006	KC343974	KC343490	KC343732	KC343248	Gomes et al. (2013)
*D.acuta*	PSCG 047^T^	MK626957	MK691225	MK726161	MK654802	MK691125	Guo et al. (2020)
*D.acutispora*	CGMCC 3.18285^T^	KX986764	KX999195	KX999235	KX999155	KX999274	Gao et al. (2017)
*D.aestuarium*	BRIP 59930a^T^	OM918686	OM960613	–	OM960595	–	Tan and Shivas (2022)
*D.africana*	CBS 150080^T^	OR198681	OR225229	OR225231	OR225227	OR225233	Matio Kemkuignou et al. (2023)
*D.afzeliae*	SDBR-CMU467^T^	OQ600199	OQ678279	OQ646886	OQ603502	OQ646882	Monkai et al. (2023)
*D.aitkeniae*	BRIP 58827a^T^	OR019750	OR039647	–	OR039640	–	Thompson et al. (2023)
*D.alangii*	CFCC 52556^T^	MH121491	MH121573	MH121451	MH121533	MH121415	Yang et al. (2018)
*D.albosinensis*	CFCC 53066^T^	MK432659	MK578059	MK443004	MK578133	MK442979	Yang et al. (2020)
*D.alleghaniensis*	CBS 495.72^T^	FJ889444	KC843228	KC343491	GQ250298	KC343249	Gomes et al. (2013)
*D.alnea*	CBS 146.46^T^	KC343008	KC343976	KC343492	KC343734	KC343250	Gomes et al. (2013)
*D.ambigua*	CBS 114015^T^	KC343010	KC343978	KC343494	KC343736	KC343252	Gomes et al. (2013)
*D.ampelina*	CBS 114016^T^	AF230751	JX275452	–	GQ250351	JX197443	Gomes et al. (2013)
*D.amygdali*	CBS 126679^T^	KC343022	KC343990	KC343506	KC343748	KC343264	Gomes et al. (2013)
*D.amygdali*	CGMCC 3.15183	KC153098	–	–	KC153089	–	Gao et al. (2014)
*D.anacardii*	CBS 720.97^T^	KC343024	KC343992	KC343508	KC343750	KC343266	Gomes et al. (2013)
*D.angelicae*	CBS 111592^T^	KC343026	KC343994	KC343511	KC343752	KC343268	Gomes et al. (2013)
*D.anhuiensis*	CNUCC 201902^T^	MN219727	MN227009	MN224550	MN224669	MN224556	Zhou and Hou (2019)
*D.annellsiae*	BRIP 59731a^T^	OM918687	OM960614	–	OM960596	–	Tan and Shivas (2022)
*D.antonovae*	BRIP 58824b^T^	OR019751	OR039648	–	OR039641	–	Thompson et al. (2023)
*D.apiculata*	LC 3418^T^	KP267896	KP293476	KP293550	KP267970	–	Gao et al. (2016)
*D.aquatica*	IFRDCC 3051^T^	JQ797437	–	–	–	–	Hu et al. (2012)
*D.araucanorum*	CBS 145285^T^	MN509711	MN509722	–	MN509733	MN974277	Zapata et al. (2020)
*D.arctii*	CBS 136.25	KC343031	KC343999	KC343515	KC343757	KC343273	Gomes et al. (2013)
*D.arecae*	CBS 161.64^T^	KC343032	KC344000	KC343516	KC343758	KC343274	Gomes et al. (2013)
*D.arengae*	CBS 114979^T^	KC343034	KC344002	KC343518	KC343760	KC343276	Gomes et al. (2013)
*D.arezzoensis*	MFLU 19-2880^T^	MT185503	MT454055	–	–	–	Li et al. (2020)
*D.aseana*	MFLUCC 12-0299a^T^	KT459414	KT459432	–	KT459448	KT459464	Hyde et al. (2016)
*D.asheicola*	CBS 136967^T^	KJ160562	KJ160518	–	KJ160594	KJ160542	Lombard et al. (2014)
*D.aspalathi*	CBS 117169^T^	KC343036	KC344004	KC343520	KC343762	KC343278	Van Rensburg *et al*. (2006)
*D.atlantica*	CECT 21217^T^	ON159893	ON364040	ON398810	ON398831	ON364019	Toghueo et al. (2023)
*D.australafricana*	CBS 111886^T^	KC343038	KC344006	KC343522	KC343764	KC343280	Gomes et al. (2013)
*D.australiana*	BRIP 66145^T^	MN708222	MN696530	–	MN696522	–	Wrona et al. (2020)
*D.australpacifica*	BRIP 60163d^T^	OM918688	OM960615	–	OM960597	–	Tan and Shivas (2022)
*D.averrhoae*	SCHM 3605^T^	AY618930	–	–	–	–	Chang et al. (2005)
*D.baccae*	CBS 136972^T^	KJ160565	MF418509	MF418264	KJ160597	–	Lombard et al. (2014)
*D.batatas*	CBS 122.21	KC343040	KC344008	KC343524	KC343766	KC343282	Gomes et al. (2013)
*D.bauhiniae*	CFCC 53071^T^	MK432648	MK578051	MK442995	MK578124	MK442970	Yang et al. (2021a)
*D.beasleyi*	BRIP 59326a^T^	OM918689	OM960616	–	OM960598	–	Tan and Shivas (2022)
*D.beckhausii*	CBS 138.27	KC343041	KC344009	KC343525	KC343767	KC343283	Gomes et al. (2013)
*D.beilharziae*	BRIP 54792^T^	JX862529	KF170921	–	JX862535	–	Thompson et al. (2015)
*D.benedicti*	ATCC MYA-4970^T^	KM669929	–	–	KM669785,	KM669862	Lawrence *et al*. (2015)
*D.berteroae*	BRIP 57900a^T^	OR019752	OR039649	–	OR039642	–	Thompson et al. (2023)
*D.betulae*	CFCC 50469^T^	KT732950	KT733020	KT732999	KT733016	KT732997	Du et al. (2016)
*D.betulicola*	CFCC 51128^T^	KX024653	KX024657	KX024661	KX024655	KX024659	Du et al. (2016)
*D.betulina*	CFCC 52562^T^	MH121497	MH121579	MH121457	MH121539	MH121421	Yang et al. (2018)
*D.biconispora*	CGMCC 3.17252^T^	KJ490597	KJ490418	KJ490539	KJ490476	–	Huang et al. (2015)
*D.bohemiae*	CBS 143347^T^	MG281015	MG281188	MG281361	MG281536	MG281710	Guarnaccia et al. (2018)
*D.bombacis*	SDBR-CMU468^T^	OQ600198	OQ678278	OQ646885	OQ603501	OQ646881	Monkai et al. (2023)
*D.bounty*	BRIP 59361a^T^	OM918690	OM960617	–	OM960599	–	Tan and Shivas (2022)
*D.brasiliensis*	CBS 133183^T^	KC343042	KC344010	KC343526	KC343768	KC343284	Gomes et al. (2013)
*D.breyniae*	CBS 148910^T^	ON400846	ON409186	ON409187	ON409188	ON409189	Matio Kemkuignou et al. (2022)
***D.brideliae***	**CBS 148911^T^**	**OR348649**	**OR468827**	**OR468807**	**OR468817**	**OR468837**	**Present study**
*D.brumptoniae*	BRIP 59403a^T^	OM918702	OM960629	–	OM960611	–	Tan and Shivas (2022)
*D.butterlyi*	BRIP 59194a^T^	OR019753	OR039650	–	OR039643	–	Thompson et al. (2023)
*D.caatingaensis*	CBS 141542^T^	KY085927	KY115600	KY115605	KY115603	KY115597	Crous et al. (2016a)
***D.cameroonensis***	**CBS 148913^T^**	**OR348650**	**OR468826**	**OR468806**	**OR468816**	**OR468836**	**Present study**
	**STMA 18289**	**OR348651**	**OR468825**	**OR468805**	**OR468815**	**OR468835**	**Present study**
	**STMA 18290**	**OR348652**	**OR468824**	**OR468804**	**OR468814**	**OR468834**	**Present study**
*D.camelliae-oleiferae*	HNZZ 027^T^	MZ509555	MZ504718	MZ504696	MZ504707	MZ504685	Yang et al. (2021b)
*D.camelliae-sinensis*	SAUCC 194.92^T^	MT822620	MT855817	MT855588	MT855932	MT855699	Sun et al. (2021)
*D.camporesii*	JZB 320143^T^	MN533805	MN561316	–	–	–	Hyde et al. (2020)
*D.canthii*	CBS 132533^T^	JX069864	KC843230	–	KC843120	KC843174	Crous et al. (2012)
*D.careyae*	SDBR-CMU469^T^	OQ600196	OQ678276	OQ646883	–	OQ646879	Monkai et al. (2023)
*D.carpini*	CBS 114437	KC343044	KC344012	KC343528	KC343770	KC343286	Gomes et al. (2013)
*D.carriae*	BRIP 59932a^T^	OM918691	OM960618	–	OM960600	–	Tan and Shivas (2022)
*D.caryae*	CFCC 52563^T^	MH121498	MH121580	MH121458	MH121540	MH121422	Yang et al. (2018)
*D.cassines*	CBS 136440^T^	KF777155	–	–	KF777244	–	Crous et al. (2013)
*D.caulivora*	CBS 127268^T^	KC343045	KC344013	KC343529	KC343771	KC343287	Gomes et al. (2013)
*D.celastrina*	CBS 139.27^T^	KC343047	KC344015	KC343531	KC343773	KC343289	Gomes et al. (2013)
*D.celeris*	CBS 143349^T^	MG281017	MG281190	MG281363	MG281538	MG281712	Guarnaccia et al. (2018)
*D.celticola*	CFCC 53074^T^	MK573948	MK574643	MK574603	MK574623	MK574587	Cao et al. (2022)
*D.celtidis*	NCYU 19-0357^T^	MW114346	MW148266	–	MW192209	–	Tennakoon et al. (2021)
*D.ceratozamiae*	CBS 131306^T^	JQ044420	–	–	–	–	Crous et al. (2011a)
*D.cercidis*	CFCC 52565^T^	MH121500	MH121582	MH121460	MH121542	MH121424	Yang et al. (2018)
*D.cerradensis*	CMRP 4331^T^	MN173198	MW751671	MW751663	MT311685	MW751655	Iantas et al. (2021)
D.cf.heveae 1	CBS 852.97	KC343116	KC344084	KC343600	KC343842	KC343358	Gomes et al. (2013)
D.cf.heveae 2	CBS 681.84	KC343117	KC344085	KC343601	KC343843	KC343359	Gomes et al. (2013)
*D.chamaeropis*	CBS 454.81	KC343048	KC344016	KC343532	KC343774	KC343290	Gomes et al. (2013)
*D.charlesworthii*	BRIP 54884m^T^	KJ197288	KJ197268	–	KJ197250	–	Thompson et al. (2015)
*D.chensiensis*	CFCC 52567^T^	MH121502	MH121584	MH121462	MH121544	MH121426	Yang et al. (2018)
*D.chiangmaiensis*	MFLUCC 18-0544^T^	OK393703	–	–	OL439483	–	de Silva et al. (2022)
*D.chimonanthi*	SCHM 3614^T^	AY622993	–	–	–	–	Chang et al. (2005)
*D.chinensis*	MFLUCC 19-0101^T^	MW187324	MW245013	–	MW205017	MW294199	de Silva et al. (2021)
*D.chongqingensis*	PSCG 435^T^	MK626916	MK691321	MK726257	MK654866	MK691209	Guo et al. (2020)
*D.chromolaenae*	MFLUCC 17-1422^T^	MH094275	–	–	–	–	Mapook et al. (2020)
*D.chrysalidocarpi*	SAUCC 194.35^T^	MT822563	MT855760	MT855532	MT855876	MT855646	Huang et al. (2021)
*D.cichorii*	MFLUCC 17-1023^T^	KY964220	KY964104	–	KY964176	KY964133	Dissanayake et al. (2017a)
*D.cinnamomi*	CFCC 52569^T^	MH121504	MH121586	MH121464	MH121546	–	Yang et al. (2018)
*D.cinerascens*	CBS 719.96	KC343050	KC344018	KC343534	KC343776	KC343292	Gomes et al. (2013)
*D.cissampeli*	CBS 141331^T^	KX228273	KX228384	KX228366	–	–	Crous et al. (2016b)
*D.citri*	CBS 135422^T^	KC843311	KC843187	MF418281	KC843071	KC843157	Udayanga et al. (2014b)
*D.citriasiana*	CBS 134240^T^	JQ954645	KC357459	MF418282	JQ954663	KC357491	Huang et al. (2013)
*D.citrichinensis*	CBS 134242^T^	JQ954648	MF418524	KJ420880	JQ954666	KC357494	Huang et al. (2013)
*D.clematidina*	MFLUCC 17-2060^T^	MT310657	MT394623	–	MT394669	MT394624	Phukhamsakda et al. (2020)
*D.collariana*	MFLUCC 17-2636^T^	MG806115	MG783041	–	MG783040	MG783042	Perera et al. (2018)
*D.compacta*	LC3083^T^	KP267854	KP293434	KP293508	KP267928	–	Gao et al. (2016)
*D.conica*	CFCC 52571^T^	MH121506	MH121588	MH121466	MH121548	MH121428	Yang et al. (2018)
*D.constrictospora*	CGMCC 3.20096^T^	MT385947	MT424702	MW022487	–	MT424718	Dissanayake et al. (2020)
*D.convolvuli*	CBS 124654	KC343054	KC344022	KC343538	KC343780	KC343296	Gomes et al. (2013)
*D.coryli*	CFCC 53083^T^	MK432661	MK578061	MK443006	MK578135	MK442981	Yang et al. (2020)
*D.corylicola*	CFCC 53986	MW839880	MW883977	MW836717	MW815894	MW836684	Gao et al. (2021)
*D.crataegi*	CBS 114435	KC343055	KC344023	KC343539	KC343781	KC343297	Gomes et al. (2013)
*D.crotalariae*	CBS 162.33^T^	KC343056	KC344024	KC343540	KC343782	KC343298	Gomes et al. (2013)
*D.crousii*	CAA823^T^	MK792311	MK837932	MK871450	MK828081	MK883835	Hilário et al. (2020)
*D.cucurbitae*	DAOM 42078^T^	KM453210	KP118848	KM453212	KM453211	–	Udayanga et al. (2015)
*D.cuppatea*	CBS 117499^T^	AY339322	JX275420	KC343541	AY339354	JX197414	Van Rensburg et al. (2006)
*D.cynaroidis*	CBS 122676	KC343058	KC344026	KC343542	KC343784	KC343300	Gomes et al. (2013)
*D.cytosporella*	CBS 137020^T^	KC843307	KC843221	MF418283	KC843116	KC843141	Udayanga et al. (2014b)
*D.decedens*	CBS 109772	KC343059	KC344027	KC343543	KC343785	KC343301	Gomes et al. (2013)
*D.delonicis*	MFLU 16-1059^T^	MT215490	MT212209	–	–	–	Perera et al. (2020)
*D.detrusa*	CBS 109770	KC343061	KC344029	KC343545	KC343787	KC343303	Gomes et al. (2013)
*D.diospyricola*	CBS 136552^T^	KF777156	–	–	–	–	Crous et al. (2013)
*D.discoidispora*	ICMP 20662^T^	KJ490624	KJ490445	KJ490566	KJ490503	–	Huang et al. (2015)
*D.drenthii*	BRIP 66524^T^	MN708229	MN696537	–	MN696526	–	Wrona et al. (2020)
*D.durionigena*	VTCC 930005^T^	MN453530	MT276159	–	MT276157	–	Crous et al. (2021)
*D.eleagni*	CBS 504.72	KC343064	KC344032	KC343548	KC343790	KC343306	Gomes et al. (2013)
*D.elaeagni-glabrae*	CGMCC 3.18287^T^	KX986779	KX999212	KX999251	KX999171	KX999281	Gao et al. (2017)
*D.ellipsospora*	CGMCC 3.20099^T^	MT385949	MT424704	MW022488	MT424684	MT424720	Dissanayake et al. (2020)
*D.endocitricola*	ZHKUCC 20-0012 ^T^	MT355682	MT409290	–	MT409336	MT409312	Dong et al. (2021)
*D.endophytica*	CBS 133811^T^	KC343065	KC344033	KC343549	KC343791	KC343307	Gomes et al. (2013)
*D.eres*	CBS 138594^T^	KJ210529	KJ420799	KJ420850	KJ210550	KJ434999	Udayanga et al. (2014a)
*D.eres*	CFCC 51632 (type strain of *D.camptothecicola*)	KY203726	KY228893	KY228881	KY228887	KY228877	Yang et al. (2017b)
*D.eres*	CGMCC 3.17089 (type strain of *D.longicicola*)	KF576267	KF576291	–	KF576242	–	Gao et al. (2015)
*D.eres*	MFLUCC 16-0113 (type strain of *D.momicola*)	KU557563	KU557587	–	KU557631	KU557611	Dissanayake et al. (2017b)
*D.eres*	CGMCC 3.15181 (strain originally named *D.mahothocarpi* Nom. Inval.)	KC153096	–	–	KC153087	–	Gao et al. (2014)
*D.eres*	CGMCC 3.17084 (type strain of *D.ellipicola*)	KF576270	KF576291	–	KF576245	–	Gao et al. (2015)
*D.eres*	CGMCC 3.17081 (type strain of *D.biguttusis*)	KF576282	KF576306	–	KF576257	–	Gao et al. (2015)
*D.etinsideae*	BRIP 64096a^T^	OM918692	OM960619	–	OM960601	–	Tan and Shivas (2022)
*D.eucalyptorum*	CBS 132525^T^	JX069862	–	–	–	–	Crous et al. (2012)
*D.eugeniae*	CBS 444.82	KC343098	KC344066	KC343582	KC343824	KC343340	Gomes et al. (2013)
*D.fibrosa*	CBS 109751	KC343099	KC344067	KC343583	KC343825	KC343341	Gomes et al. (2013)
*D.fici-septicae*	MFLU 18-2588^T^	MW114348	MW148268	–	MW192211	–	Tennakoon et al. (2021)
*D.foeniculina*	CBS 111553^T^	KC343101	KC344069	KC343585	KC343827	KC343343	Gomes et al. (2013)
*D.foeniculina*	CBS 129528	JF951146	KC843205	–	KC843100	KC843124	Crous et al. (2011b), Udayanga et al. (2014b)
*D.foikelawen*	CBS 145289^T^	MN509713	MN509724	–	MN509735	MN974278	Zapata et al. (2020)
*D.forlicesenica*	MFLUCC 17-1015^T^	KY964215	KY964099	–	KY964171	–	Dissanayake et al. (2017a)
*D.fraxini-angustifoliae*	BRIP 54781^T^	JX862528	KF170920	–	JX852534	–	Tan et al. (2013)
*D.fraxinicola*	CFCC 52582^T^	MH121517	–	–	MH121559	MH121435	Yang et al. (2018)
*D.fructicola*	MAFF 246408^T^	LC342734	LC342736	LC342737	LC342735	LC342738	Crous et al. (2019)
*D.fujianensis*	JZB 320149^T^	MW010212	MW056008	–	MW20523	MW205212	Manawasinghe et al. (2021)
*D.fukushii*	MAFF 625034	JQ807469	–	–	JQ807418	–	Baumgartner et al. (2013)
*D.fulvicolor*	PSCG 051^T^	MK626859	MK691236	MK726163	MK654806	MK691132	Guo et al. (2020)
*D.fusicola*	CGMCC 3.17087^T^	KF576281	KF576305	–	KF576256	KF576233	Gao et al. (2015)
*D.fusiformis*	JZB 320156^T^	MW010218	MW056014	–	MW205234	MW205218	Manawasinghe et al. (2021)
*D.ganjae*	CBS 180.91^T^	KC343112	KC344080	KC343596	KC343838	KC343354	Gomes et al. (2013)
*D.ganzhouensis*	CFCC 53087^T^	MK432665	MK578065	MK443010	MK578139	MK442985	Yang et al. (2021a)
*D.gardeniae*	CBS 288.56	KC343113	KC344081	KC343597	KC343839	KC343355	Gomes et al. (2013)
*D.garethjonesii*	MFLUCC 12-0542a^T^	KT459423	KT459441	–	KT459457	KT459470	Hyde et al. (2016)
*D.glabrae*	SCHM 3622^T^	AY601918	–	–	–	–	Chang et al. (2005)
*D.globoostiolata*	MFLUCC 23-0025^T^	OQ600200	OQ678280	–	OQ603503	–	Monkai et al. (2023)
*D.gossiae*	BRIP 59730a^T^	OM918693	OM960620	–	OM960602	–	Tan and Shivas (2022)
*D.goulteri*	BRIP 55657a^T^	KJ197290	KJ197270	–	KJ197252	–	Thompson et al. (2015)
*D.grandiflori*	SAUCC194.84^T^	MT822612	MT855809	MT85558	MT855924	MT855691	Sun et al. (2021)
*D.griceae*	BRIP 67014a^T^	OM918694	OM960621	–	OM960603	–	Tan and Shivas (2022)
*D.guangdongensis*	ZHKUCC 20-0014^T^	MT355684	MT409292	–	MT409338	MT409314	Dong et al. (2021)
*D.guangxiensis*	JZB 320094^T^	MK335772	MK500168	–	MK523566	MK736727	Manawasinghe et al. (2019)
*D.guizhouensis*	GZAAS 20-0338^T^	OM060254	OL961762	–	OL961761	OL961763	Bhunjun et al. (2022)
*D.gulyae*	BRIP 54025^T^	JF431299	KJ197271	–	JN645803	–	Thompson et al. (2015)
*D.guttulata*	CGMCC 3.20100^T^	MT385950	MT424705	MW022491	MT424685	MW022470	Dissanayake et al. (2020)
*D.hartii*	BRIP 60285e^T^	OR019754	OR039651	–	OR039644	–	Thompson et al. (2023)
*D.helianthi*	CBS 592.81^T^	KC343115	KC344083	KC343599	KC343841	JX197454	Gomes et al. (2013)
*D.helicis*	CBS 138596^T^	KJ210538	KJ420828	KJ420875	KJ210559	KJ435043	Udayanga et al. (2014a)
*D.heliconiae*	SAUCC 194.77^T^	MT822605	MT855802	MT855573	MT855917	MT855684	Sun et al. (2021)
*D.heterophyllae*	CBS 143769^T^	MG600222	MG600226	MG600220	MG600224	MG600218	Marin-Felix et al. (2019)
*D.heterostemmatis*	SAUCC 194.85^T^	MT822613	MT855810	MT855581	MT855925	MT855692	Sun et al. (2021)
*D.hickoriae*	CBS 145.26^T^	KC343118	KC344086	KC343602	KC343844	KC343360	Gomes et al. (2013)
*D.hispaniae*	CBS 143351^T^	MG281123	MG281296	MG281471	MG281644	MG281820	Guarnaccia et al. (2018)
*D.hongkongensis*	CBS 115448^T^	KC343119	KC344087	KC343603	KC343845	KC343361	Gomes et al. (2013)
*D.hordei*	CBS 481.92	KC343120	KC344088	KC343604	KC343846	KC343362	Gomes et al. (2013)
*D.howardiae*	BRIP 59697a^T^	OM918695	OM960622	–	OM960604	–	Tan and Shivas (2022)
*D.hsinchuensis*	NTUPPMCC 18-153-1^T^	MZ268409	MZ268430	MZ268493	MZ268472	MZ268451	Ariyawansa et al. (2021)
*D.huangshanensis*	CNUCC 201903^T^	MN219730	MN227011	MN224558	MN224678	–	Zhou and Hou (2019)
*D.hubeiensis*	JZB 320123^T^	MK335809	MK500148	–	MK523570	MK500235	Manawasinghe et al. (2019)
*D.humulicola*	CT2018-1^T^	MN152927	–	MN180213	MN180207	MN180204	Allan-Perkins et al. (2020)
*D.hunanensis*	HNZZ 023^T^	MZ509550	MZ504713	MZ504691	MZ504702	MZ504680	Yang et al. (2021b)
*D.hungariae*	CBS 143353^T^	MG281126	MG281299	MG281474	MG281647	MG281823	Guarnaccia et al. (2018)
*D.iberica*	CECT 21218^T^	ON159902	ON364049	ON398819	ON398841	ON364028	Toghueo et al. (2023)
*D.ilicicola*	FPH 2015502^T^	MH171064	MH171074	MH171084	–	–	Lin et al. (2018)
*D.impulsa*	CBS 114434	KC343121	KC344089	KC343605	KC343847	KC343363	Gomes et al. (2013)
*D.incompleta*	CGMCC 3.18288^T^	KX986794	KX999226	KX999265	KX999186	KX999289	Gao et al. (2017)
*D.inconspicua*	CBS 133813^T^	KC343123	KC344091	KC343607	KC343849	KC343365	Gomes et al. (2013)
*D.infecunda*	CBS 133812^T^	KC343126	KC344094	KC343610	KC343852	KC343368	Gomes et al. (2013)
*D.infertilis*	CBS 230.52^T^	KC343052	KC344020	KC343536	KC343778	KC343294	Guarnaccia and Crous (2017)
*D.irregularis*	CGMCC 3.20092^T^	MT385951	MT424706	–	MT424686	MT424721	Dissanayake et al. (2020)
*D.isoberliniae*	CBS 137981^T^	KJ869133	KJ869245	–	–	–	Crous et al. (2014a)
	**STMA18291**	**OR348654**	**OR468822**	**OR468802**	**OR468812**	**OR468832**	**Present study**
	**STMA18245**	**OR348653**	**OR468823**	**OR468803**	**OR468813**	**OR468833**	**Present study**
*D.italiana*	MFLUCC 18-0090^T^	MH846237	MH853688	–	MH853686	MH853690	Hyde et al. (2019)
*D.jinxiu*	CGMCC3.20269^T^	MW477881	MW480877	MW480865	MW480873	MW480869	Wang et al. (2021)
*D.juglandia*	CBS 121004^T^	KC343134	KC344102	KC343618	KC343860	KC343376	Gomes et al. (2013)
*D.juglandicola*	CFCC 51134^T^	MW477881	KX024634	–	KX024628	KX024616	Yang et al. (2017a)
*D.kadsurae*	CFCC 52586^T^	MH121521	MH121600	MH121479	MH121563	MH121439	Yang et al. (2018)
*D.kochmanii*	BRIP 54033^T^	JF431295	–	–	JN645809	–	Thompson et al. (2011)
*D.kongii*	BRIP 54031^T^	JF431301	KJ197272	–	JN645797	–	Thompson et al. (2011)
*D.krabiensis*	MFLUCC 17-2481^T^	MN047101	MN431495	–	MN433215		Dayarathne et al. (2020)
*D.lenispora*	CGMCC 3.20101^T^	MT385952	MT424707	MW022493	MT424687	MW022472	Dissanayake et al. (2020)
*D.leptostromiformis*	CBS 558.93	KC343244	KC344212	KC343728	KC343970	KC343486	Gomes et al. (2013)
*D.leucospermi*	CBS 111980^T^	JN712460	KY435673	KY435653	KY435632	KY435663	Crous et al. (2011c)
*D.limonicola*	CBS 142549^T^	MF418422	MF418582	MF418342	MF418501	MF418256	Guarnaccia and Crous (2017)
*D.liquidambaris*	SCHM 3621^T^	AY601919	–	–	–	–	Chang et al. (2005)
*D.litchicola*	BRIP 54900^T^	JX862533	KF170925	–	JX862539	–	Tan et al. (2013)
*D.litchii*	SAUCC 194.22^T^	MT822550	MT855747	MT855519	MT855863	MT855635	Sun et al. (2021)
*D.lithocarpi*	CGMCC 3.15175^T^	KC153104	KF576311	–	KC153095	–	Gao et al. (2014)
*D.litoricola*	MFLUCC 16-1195^T^	MF190139	–	–	–	–	Senanayake *et al*. (2017)
*D.longicolla*	FAU 599^T^	KJ590728	KJ610883	KJ659188	KJ590767	KJ612124	Udayanga et al. (2015)
*D.longispora*	CBS 194.36^T^	KC343135	KC344103	KC343619	KC343861	KC343377	Gomes et al. (2013)
*D.lonicerae*	MFLUCC 17-0963^T^	KY964190	KY964073	–	KY964146	KY964116	Dissanayake et al. (2017a)
*D.lovelaceae*	BRIP 60163a^T^	OM918696	OM960623	–	OM960605	–	Tan and Shivas (2022)
*D.lusitanicae*	CBS 123212^T^	KC343136	KC344104	KC343620	KC343862	KC343378	Gomes et al. (2013)
*D.lutescens*	SAUCC 194.36^T^	MT822564	MT855761	MT855533	MT855877	MT855647	Sun et al. (2021)
*D.macadamiae*	BRIP 66526^T^	MN708230	MN696539	–	MN696528	–	Wrona et al. (2020)
*D.machili*	SAUCC 194.111^T^	MT822639	MT855836	MT855606	MT855951	MT855718	Huang et al. (2021)
*D.macintoshii*	BRIP 55064a^T^	KJ197289	KJ197269	–	KJ197251	–	Thompson et al. (2015)
*D.malorum*	CBS142383^T^	KY435638	KY435668	KY435648	KY435627	KY435658	Santos et al. (2017)
*D.manihotia*	CBS 505.76	KC343138	KC344106	KC343622	KC343864	KC343380	Gomes et al. (2013)
*D.marina*	MFLU 17-2622^T^	MN047102	–	–	–	–	Dayarathne et al. (2020)
*D.maritima*	DAOMC 250563^T^	KU552025	KU574615	–	KU552023	–	Tanney et al. (2016)
*D.masirevicii*	BRIP 57892a^T^	KJ197277	KJ197257	–	KJ197239	–	Thompson et al. (2015)
*D.mayteni*	CBS 133185^T^	KC343139	KC344107	KC343623	KC343865	KC343381	Gomes et al. (2013)
*D.maytenicola*	CBS 136441^T^	KF777157	KF777250	–	–	–	Crous et al. (2013)
*D.mclennaniae*	BRIP 60072a^T^	OM918697	OM960624	–	OM960606	–	Tan and Shivas (2022)
*D.mediterranea*	DAL-34	MT007489	MT006686	MT007095	MT006989	MT006761	Beluzán et al. (2021)
*D.megalospora*	CBS 143.27	KC343140	KC344108	KC343624	KC343866	KC343382	Gomes et al. (2013)
*D.melastomatis*	SAUCC 194.55^T^	MT822583	MT855780	MT855551	MT855896	MT855664	Sun et al. (2021)
*D.meliae*	CFCC 53089^T^	MK432657	MK578057	ON081662	ON081654	–	Cao et al. (2022)
*D.melitensis*	CBS 142551^T^	MF418424	MF418584	MF418344	MF418503	MF418258	Guarnaccia and Crous (2017)
*D.melonis*	CBS 507.78^T^	KC343142	KC344110	KC343626	KC343868	KC343384	Gomes et al. (2013)
*D.micheliae*	SCHM 3603	AY620820	–	–	–	–	Chang et al. (2005)
*D.middletonii*	BRIP 54884e^T^	KJ197286	KJ197266	–	KJ197248	–	Thompson et al. (2015)
*D.millettiae*	GUCC 9167^T^	MK398674	MK502089	–	MK480609	MK502086	Long et al. (2019)
*D.minima*	CGMCC 3.20097^T^	MT385953	MT424708	MW022496	MT424688	MT424722	Dissanayake et al. (2020)
*D.minusculata*	CGMCC 3.20098^T^	MT385957	MT424712	MW022499	MT424692	MW022475	Dissanayake et al. (2020)
*D.miriciae*	BRIP 54736j^T^	KJ197283	KJ197263	–	KJ197245	–	Thompson et al. (2015)
*D.monetii*	MF-Ha18-049^T^	MW008494	MW008505	MZ671965	MW008516	MZ671939	Gomzhina and Gannibal (2022)
*D.moorei*	BRIP 61500b^T^	OR019755	OR039652	–	OR039645	–	Thompson et al. (2023)
*D.moriniae*	BRIP 60190a^T^	OM918698	OM960625	–	OM960607	–	Tan and Shivas (2022)
*D.multigutullata*	ICMP 20656^T^	KJ490633	KJ490454	KJ490575	KJ490512	–	Huang et al. (2015)
*D.musigena*	CBS 129519^T^	KC343143	KC344111	KC343627	KC343869	KC343385	Gomes et al. (2013)
*D.myracrodruonis*	URM 7972^T^	MK205289	MK205291	–	MK213408	MK205290	da Silva et al. (2019)
*D.neatei*	BRIP 60289a^T^	OR019756	OR039653	–	OR039646	–	Thompson et al. (2023)
*D.nebulae*	PMM 1681^T^	KY511337	KY511369	–	MH708552	–	Lesuthu et al. (2019)
*D.neilliae*	CBS 144.27^T^	KC343144	KC344112	KC343628	KC343870	KC343386	Gomes et al. (2013)
*D.neoarctii*	CBS 109490	KC343145	KC344113	KC343629	KC343871	KC343387	Gomes et al. (2013)
*D.neoraonikayaporum*	MFLUCC 14-1136^T^	KU712449	KU743988	–	KU749369	KU749356	Doilom et al. (2017)
*D.nigra*	JZB 320170^T^	MN653009	MN887113	–	MN892277	–	Hyde et al. (2020)
*D.nobilis*	CBS 587.79	KC343153	KC344121	KC343637	KC343879	KC343395	Gomes et al. (2013)
*D.nomurai*	CBS 157.29	KC343154	KC344122	KC343638	KC343880	KC343396	Gomes et al. (2013)
*D.norfolkensis*	BRIP 59718a^T^	OM918699	OM960626	–	OM960608	–	Tan and Shivas (2022)
*D.nothofagi*	BRIP 54801^T^	JX862530	KF170922	–	JX862536	–	Tan et al. (2013)
*D.novem*	CBS 127271^T^	KC343157	KC344125	KC343641	KC343883	KC343399	Gomes et al. (2013)
*D.novem*	CBS 117165	DQ286285	–	–	DQ286259	–	Petrović et al. (2018)
*D.obtusifoliae*	CBS 143449^T^	MG386072	–	MG386137	–	–	Crous et al. (2017)
*D.ocoteae*	CBS 141330^T^	KX228293	KX228388	–	–	–	Crous et al. (2016b)
*D.oculi*	HHUF 30565^T^	LC373515	LC373519	–	LC373517	–	Ozawa et al. (2019)
*D.oncostoma*	CBS 589.78	KC343162	KC344130	KC343646	KC343888	KC343404	Gomes et al. (2013)
*D.oraccinii*	LC 3166^T^	KP267863	KP293443	KP293517	KP267937	–	Gao et al. (2016)
*D.orixae*	HKAS 121465^T^	OK283041	OK432278	OK484486	OK432279	OK484485	Lu et al. (2022)
*D.osmanthi*	GUCC 9165^T^	MK398675	MK502090	–	MK480610	MK502087	Long et al. (2019)
*D.ovalispora*	ICMP 20659^T^	KJ490628	KJ490449	KJ490570	KJ490507	–	Huang et al. (2015)
*D.ovoidea*	CGMCC 3.17092^T^	KF576264	KF576288	–	KF576239	KF576222	Gao et al. (2015)
*D.oxe*	CBS 133186^T^	KC343164	KC344132	KC343648	KC343890	KC343406	Gomes et al. (2013)
*D.pachirae*	COAD 2074^T^	MG559537	MG559541	–	MG559539	MG559535	Milagres et al. (2018)
D.padivar.padi	CBS 114200	KC343169	KC344137	KC343653	KC343895	KC343411	Gomes et al. (2013)
*D.padina*	CFCC 52590^T^	MH121525	MH121604	MH121483	MH121567	MH121443	Yang et al. (2018)
*D.pandanicola*	MFLUCC 17-0607^T^	MG646974	MG646930	–	–	–	Tibpromma et al. (2018)
*D.paranensis*	CBS 133184	KC343171	KC344139	KC343655	KC343897	KC343413	Gomes et al. (2013)
*D.parapterocarpi*	CBS 137986^T^	KJ869138	KJ869248	–	–	–	Crous et al. (2014a)
*D.parva*	PSCG 034^T^	MK626919	MK691248	MK726210	MK654858	–	Guo et al. (2020)
*D.pascoei*	BRIP 54847^T^	JX862532	KF170924	–	JX862538	–	Tan et al. (2013)
*D.passiflorae*	CBS 132527^T^	JX069860	KY435674	KY435654	KY435633	KY435664	Crous et al. (2012)
*D.passifloricola*	CBS 141329^T^	KX228292	KX228387	KX228367	–	–	Crous et al. (2016b)
*D.patagonica*	CBS 145291^T^	MN509717	MN509728	–	MN509739	MN974279	Zapata et al. (2020)
*D.penetriteum*	LC 3353	KP714505	KP714529	KP714493	KP714517	–	Gao et al. (2016)
*D.perjuncta*	CBS 109745^T^	KC343172	KC344140	KC343656	KC343898	KC343414	Gomes et al. (2013)
*D.perniciosa*	CBS 124030	KC343149	KC344117	KC343633	KC343875	KC343391	Gomes et al. (2013)
*D.perseae*	CBS 151.73	KC343173	KC344141	KC343657	KC343899	KC343415	Gomes et al. (2013)
*D.pescicola*	MFLUCC 16-0105^T^	KU557555	KU557579	–	KU557623	KU557603	Dissanayake et al. (2017b)
*D.phaseolorum*	CBS 113425	KC343174	KC344142	KC343658	KC343900	KC343416	Gomes et al. (2013)
*D.phillipsii*	CAA 817^T^	MK792305	MN000351	MK871445	MK828076	MK883831	Hilário et al. (2020)
*D.phragmitis*	CBS 138897^T^	KP004445	KP004507	KP004503	–	–	Crous et al. (2014b)
*D.phyllanthicola*	SCHM 3680^T^	AY620819	–	–	–	–	Chang et al. (2005)
*D.platzii*	BRIP 60353a^T^	OM918700	OM960627	–	OM960609	–	Tan and Shivas (2022)
*D.podocarpi-macrophylli*	CGMCC 3.18281^T^	KX986774	KX999207	KX999246	KX999167	KX999278	Gao et al. (2017)
*D.poincianellae*	URM 7932^T^	MH989509	MH989537	MH989539	MH989538	MH989540	Crous et al. (2018a)
*D.pometiae*	SAUCC 194.72^T^	MT822600	MT855797	MT855568	MT855912	MT855679	Huang et al. (2021)
*D.portugallica*	CBS 144228^T^	MH063905	MH063917	MH063899	MH063911	MH063893	Guarnaccia and Crous (2018)
***D.pseudoanacardii***	**CBS 148909^T^**	**OR348655**	**OR468821**	**OR468801**	**OR468811**	**OR468831**	**Present study**
	**STMA 18247**	**OR348656**	**OR468820**	**OR468800**	**OR468810**	**OR468830**	**Present study**
	**STMA 18292**	**OR348657**	**OR468819**	**OR468799**	**OR468809**	**OR468829**	**Present study**
*D.pseudoalnea*	CFCC 54190^T^	MZ727037	MZ753487	MZ781302	MZ816343	MZ753468	Jiang et al. (2021)
*D.pseudobiguttulata*	ICMP 20657^T^	KJ490582	KJ490403	KJ490524	KJ490461	–	Huang et al. (2015)
*D.pseudoinconspicua*	URM 7874^T^	MH122538	MH122524	MH122517	MH122533	MH122528	Crous et al. (2018b)
*D.pseudomangiferae*	CBS 101339^T^	KC343181	KC344149	KC343665	KC343907	KC343423	Gomes et al. (2013)
*D.pseudooculi*	HHUF 30617^T^	LC373515	LC373519	–	LC373517	–	Ozawa et al. (2019)
*D.pseudophoenicicola*	CBS 462.69^T^	KC343184	KC344152	KC343668	KC343910	KC343426	Gomes et al. (2013)
*D.pseudotsugae*	MFLU 15-3228^T^	KY964225	KY964108	–	KY964181	KY964138	Dissanayake et al. (2017a)
*D.psoraleae*	CBS 136412^T^	KF777158	KF777251	–	KF777245	–	Crous et al. (2013)
*D.psoraleae-pinnatae*	CBS 136413^T^	KF777159	KF777252	–	–	–	Crous et al. (2013)
*D.pterocarpi*	MFLUCC 10-0571	JQ619899	JX275460	–	JX275416	JX197451	Udayanga et al. (2012b)
*D.pterocarpicola*	MFLUCC 10-0580a	JQ619887	JX275441	–	JX275403	JX197433	Udayanga et al. (2012b)
*D.pulla*	CBS 338.89^T^	KC343152	KC344120	KC343636	KC343878	KC343394	Gomes et al. (2013)
*D.pungensis*	SAUCC 194.112^T^	MT822640	MT855837	MT855607	MT855952	MT855719	Sun et al. (2021)
*D.pustulata*	CBS 109742	KC343185	KC344153	KC343669	KC343911	KC343427	Gomes et al. (2013)
*D.pyracanthae*	CBS142384^T^	KY435635	KY435666	KY435645	KY435625	KY435656	Santos et al. (2017)
*D.quercicola*	CSUFTCC 104^T^	ON076567	–	ON081667	ON081659	ON081670	Cao et al. 2022
*D.racemosae*	CBS 143770^T^	MG600223	MG600227	MG600221	MG600225	MG600219	Marin-Felix et al. (2019)
*D.raonikayaporum*	CBS 133182^T^	KC343188	KC344156	KC343672	KC343914	KC343430	Gomes et al. (2013)
***D.rauvolfiae***	**CBS 148912^T^**	**OR348658**	**OR468818**	**OR468798**	**OR468808**	**OR468828**	**Present study**
*D.ravennica*	MFLUCC 15–0479^T^	KU900335	KX432254	–	KX365197	–	Dissanayake et al. (2017a)
*D.rhodomyrti*	CFCC 53101^T^	MK432643	MK578046	MK442990	MK578119	MK442965	Cao et al. (2022)
*D.rhoina*	CBS 146.27	KC343189	KC344157	KC343673	KC343915	KC343431	Gomes et al. (2013)
*D.rizhaoensis*	CFCC 57562^T^	OP955930	OP959773	OP959785	OP959767	OP959782	Zhu et al. (2023)
*D.rosae*	MFLUCC 17-2658^T^	MG828894	MG843878	–	–	MG829273	Wanasinghe et al. (2018)
*D.rosicola*	MFLU 17-0646^T^	MG828895	MG843877	–	MG829270	MG829274	Wanasinghe et al. (2018)
*D.rosiphthora*	COAD 2913^T^	MT311196	–	–	MT313692	MT313690	Pereira et al. (2021)
*D.rossmaniae*	CAA 762^T^	MK792290	MK837914	MK871432	MK828063	MK883822	Hilário et al. (2020)
*D.rostrata*	CFCC 50062^T^	KP208847	KP208855	KP208851	KP208853	KP208849	Fan et al. (2015)
*D.rudis*	CBS 113201	KC343234	KC344202	KC343718	KC343960	KC343476	Udayanga et al. (2014b)
*D.rumicicola*	MFLUCC 18-0739^T^	MH84623	MK049555	–	MK049554	–	Hyde et al. (2019)
*D.saccarata*	CBS 116311^T^	KC343190	KC344158	KC343674	KC343916	KC343432	Gomes et al. (2013)
*D.sackstonii*	BRIP 54669b^T^	KJ197287	KJ197267	–	KJ197249	–	Thompson et al. (2015)
*D.salicicola*	BRIP 54825^T^	JX862531	KF170923	–	JX862537	–	Tan et al. (2013)
*D.salinicola*	MFLU 18-0553^T^	MN047098	–	–	MN077073	–	Dayarathne et al. (2020)
*D.samaneae*	SDBR-CMU470^T^	OQ600197	OQ678277	OQ646880	OQ603500	OQ646884	Monkai et al. (2023)
*D.sambuci*	CFCC 51986	KY852495	KY852511	KY852503	KY852507	KY852499	Yang et al. (2018)
*D.sapindicola*	CFCC 55344^T^	MW881507	MW898937	MW898940	MW898934	MW898943	Si et al. (2022)
*D.schimae*	CFCC 53103^T^	MK432640	MK578043	MK442987	MK578116	MK442962	Yang et al. (2021a)
*D.schini*	CBS 133181^T^	KC343191	KC344159	KC343675	KC343917	KC343433	Gomes et al. (2013)
*D.schisandrae*	CFCC 51988^T^	KY852497	KY852513	KY852505	KY852509	KY852501	Yang *et al*. (2018)
*D.schoeni*	MFLU 15-1279^T^	KY964226	KY964109	–	KY964182	KY964139	Dissanayake et al. (2017a)
*D.sclerotioides*	CBS 296.67^T^	KC343193	KC344161	KC343677	KC343919	KC343435	Gomes et al. (2013)
*D.scobina*	CBS 251.38	KC343195	KC344163	KC343679	KC343921	KC343437	Gomes et al. (2013)
*D.searlei*	BRIP 66528^T^	MN708231	MN696540	–	–	–	Wrona et al. (2020)
*D.sennae*	CFCC 51636^T^	KY203724	KY228891	–	KY228885	KY228875	Yang et al. (2017c)
*D.sennicola*	CFCC 51634^T^	KY203722	KY228889	–	KY228883	KY228873	Yang et al. (2017c)
*D.serafiniae*	BRIP 55665a^T^	KJ197274	KJ197254	–	KJ197236	–	Thompson et al. (2015)
*D.shaanxiensis*	CFCC 53106	MK432654	–	MK443001	MK578130	MK442976	Yang et al. (2020)
*D.shawiae*	BRIP 64534a^T^	OM918701	OM960628	–	OM960610	–	Tan and Shivas (2022)
*D.shennongjiaensis*	CNUCC201905^T^	MN216229	MN227012	MN224559	MN224672	MN224551	Zhou and Hou (2019)
*D.siamensis*	MFLUCC 10-0573a	JQ619879	JX275429	–	JX275393	–	Udayanga et al. (2012b)
*D.silvicola*	CFCC 54191^T^	MZ727041	MZ753491	MZ753481	MZ816347	MZ753472	Jiang et al. (2021)
*D.sinensis*	CGMCC 3.19521^T^	MK637451	MK660447	–	MK660449	–	Feng et al. (2019)
*D.smilacicola*	CFCC 54582^T^	OP955933	OP959776	OP959788	OP959770	OP959779	Zhu et al. (2023)
*D.sojae*	CBS 139282^T^	KJ590719	KJ610875	KJ659208	KJ590762	KJ612116	Udayanga et al. (2015)
*D.spartinicola*	CBS 140003^T^	KR611879	KR857695	KR857696	–	–	Crous et al. (2015a)
*D.spinosa*	PSCG 383^T^	MK626849	MK691234	MK726156	MK654811	MK691129	Guo et al. (2020)
*D.sterilis*	CBS 136969^T^	KJ160579	KJ160528	MF418350	KJ160611	KJ160548	Lombard et al. (2014)
*D.stewartii*	CBS 193.36	FJ889448	–	–	GQ250324	–	Santos et al. (2010)
*D.stictica*	CBS 370.54	KC343212	KC344180	KC343696	KC343938	KC343454	Gomes et al. (2013)
*D.subclavata*	ICMP 20663^T^	KJ490630	KJ490451	KJ490572	KJ490509	–	Huang et al. (2015)
*D.subcylindrospora*	KUMCC 17-0151^T^	MG746629	MG746631	–	MG746630	–	Hyde et al. (2018)
*D.subellipicola*	KUMCC 17-0153^T^	MG746632	MG746634	–	MG746633	–	Hyde et al. (2018)
*D.subordinaria*	CBS 101711	KC343213	KC344181	KC343697	KC343939	KC343455	Gomes et al. (2013)
*D.taoicola*	MFLUCC 16-0117^T^	KU557567	KU557591	–	KU557635	–	Dissanayake et al. (2017b)
*D.tarchonanthi*	CBS 146073^T^	MT223794	MT223733	MT223759	–	–	Crous et al. (2020)
*D.tecomae*	CBS 100547	KC343215	KC344183	KC343699	KC343941	KC343457	Gomes et al.(2013)
*D.tectonae*	MFLUCC 12-0777^T^	KU712430	KU743977	–	KU749359	KU749345	Doilom et al. (2017)
*D.tectonendophytica*	MFLUCC 13-0471^T^	KU712439	KU743986	–	KU749367	KU749354	Doilom et al. (2017)
*D.tectonigena*	MFLUCC 12-0767^T^	KU712429	KU743976	–	KU749371	KU749358	Doilom et al. (2017)
*D.terebinthifolii*	CBS 133180^T^	KC343216	KC344184	KC343700	KC343942	KC343458	Gomes et al. (2013)
*D.thunbergiae*	MFLUCC 10-0756a	JQ619893	JX275449	–	JX275409	JX197440	Udayanga et al. (2012b)
*D.thunbergiicola*	MFLUCC 12-0033^T^	KP715097	–	–	KP715098	–	Liu et al. (2015)
*D.tibetensis*	CFCC 51999^T^	MF279843	MF279873	MF279828	MF279858	MF279888	Fan et al. (2018)
*D.torilicola*	MFLUCC 17-1051^T^	KY964212	KY964096	–	KY964168	KY964127	Dissanayake et al. (2017a)
*D.toxica*	CBS 534.93^T^	KC343220	KC344188	KC343704	KC343946	KC343462	Gomes et al.(2013)
*D.toxicodendri*	FFPRI 420987	LC275192	LC275224	LC275216	LC275216	LC275200	Ando et al. (2017)
*D.trevorrowii*	BRIP 70737a^T^	OM918703	OM960630	–	OM960612	–	Tan and Shivas (2022)
*D.tulliensis*	BRIP 62248a	KR936130	KR936132	–	KR936133	–	Crous et al. (2015b)
*D.tuyouyouiae*	BRIP 75017a^T^	OQ917074	OQ889559	–	OQ889558	–	Tan and Shivas (2023)
*D.ueckeri*	FAU 656	KJ590726	KJ610881	KJ659215	KJ590747	KJ612122	Huang et al. (2015)
*D.ukurunduensis*	CFCC 52592^T^	MH121527	–	MH121485	MH121569	MH121445	Yang et al. (2018)
*D.undulata*	CGMCC 3.18293^T^	KX986798	KX999230	KX999269	KX999190	–	Gao et al. (2017)
*D.unshiuensis*	CGMCC3.17569^T^	KJ490587	KJ490408	KJ490529	KJ490466	–	Huang et al. (2015)
*D.vaccinii*	CBS 160.32^T^	AF317578	KC344196	KC343712	GQ250326	KC343470	Gomes et al. (2013)
*D.vacuae*	CAA 830^T^	MK792309	MK837931	MK871449	MK828080	MK883834	Hilário et al. (2020)
*D.vangoghii*	MF-Ha18-046^T^	MW008492	MW008503	MZ671963	MW008514	MZ671937	Gomzhina and Gannibal (2022)
*D.vangueriae*	CBS 137985^T^	KJ869137	KJ869247	–	–	–	Crous et al. (2014a)
*D.vawdreyi*	BRIP 57887a	KR936126	KR936128	–	KR936129	–	Crous et al. (2015b)
*D.velutina*	CGMCC 3.18286^T^	KX986790	KX999223	KX999261	KX999182	–	Gao et al. (2017)
*D.verniciicola*	CFCC 53109^T^	MK573944	MK574639	MK574599	MK574619	MK574583	Yang et al. (2021a)
*D.vexans*	CBS 127.14	KC343229	KC344197	KC343713	KC343955	KC343471	Gomes et al.(2013)
*D.viciae*	JZB 320179^T^	OP626092	OP627281	OP627279	OP627280	–	Abeywickrama et al. (2023)
*D.viniferae*	JZB 320071^T^	MK341551	MK500112	–	MK500107	MK500119	Manawasinghe et al. 2019
*D.virgiliae*	CBS 138788^T^	KP247573	KP247582	–	–	–	Machingambi et al. (2015)
*D.vitimegaspora*	STE-U 2675	AF230749	–	–	–	–	Mostert et al. (2001)
*D.vochysiae*	LGMF 1583^T^	MG976391	MK007527	MK033323	MK007526	MK007528	Noriler et al. (2019)
*D.woolworthii*	CBS 148.27	KC343245	KC344213	KC343729	KC343971	KC343487	Gomes et al. (2013)
*D.xishuangbanica*	CGMCC 3.18282^T^	KX986783	KX999216	KX999255	KX999175	–	Gao et al. (2017)
*D.xunwuensis*	CFCC 53085^T^	MK432663	MK578063	MK443008	MK578137	MK442983	Yang et al. (2021a)
*D.yunnanensis*	CGMCC 3.18289^T^	KX986796	KX999228	KX999267	KX999188	KX999290	Gao et al. (2017)
*D.zaobaisu*	PSCG 031^T^	MK626922	MK691245	MK726207	MK654855	–	Guo et al. (2020)
*D.zaofenghuang*	CGMCC3.20271^T^	MW477883	MW480875	–	MW480871	MW480867	Wang et al. (2021)
*Diaporthellacorylina*	CBS 121124	KC343004	KC343972	KC343488	KC343730	KC343246	Gomes et al. (2013)

^1^ATCC: American Type Culture Collection, Virginia, USA; BRIP: Queensland Plant Pathology Herbarium, Brisbane, Australia; CAA: Collection of Artur Alves housed at Department of Biology, University of Aveiro, Portugal; CBS: Westerdijk Fungal Biodiversity Institute, Utrecht, the Netherlands; CECT: Spanish Type Culture Collection at University of Valencia, Valencia, Spain; CFCC: China Forestry Culture Collection Center, Beijing, China; CGMCC: Chinese General Microbiological Culture Collection Center, Beijing, China; CMRP: Taxonline Microbiological Collections of Paraná Network, at the Federal University of Paraná, Brazil; CNUCC: Capital Normal University Culture Collection Center, Beijing, China; COAD: Culture Collection of Octávio de Almeida Drumond. Universidade Federal de Viçosa, Viçosa, Brasil; CPC: Culture collection of Pedro Crous, housed at Westerdijk Fungal Biodiversity Institute; CSUFTCC: Central South University of Forestry and Technology Culture Collection, Hunan, China; DAOM: Plant Research Institute, Department of Agriculture (Mycology), Ottawa, Canada; DAL: strains deposited in fungal collection of the Instituto Agroforestal Mediterráneo–Universitat Politècnica de València, Valencia, Spain; DAOMC: Canadian Collection of Fungal Cultures, Ottawa, Canada; FPH: personal collection of Francesca Peduto Hand, Department of Plant Pathology, The Ohio State University, Columbus; GUCC: Culture Collection at the Department of Plant Pathology, Agriculture College, Guizhou University, China; GZAAS: Herbarium of Guizhou Academy of Agricultural Sciences, Guiyang, China; FAU: Isolates in culture collection of Systematic Mycology and Microbiology Laboratory; FFPRI: the Forestry and Forest Products Research Institute culture collection, Tsukuba, Japan; HKAS: Chinese Academy of Sciences, Kunming, China; HNZZ: Central South University of Forestry and Technology, Changsha, China; ICMP: International Collection of Micro-organisms from Plants, Landcare Research, Private Bag 92170, Auckland, New Zealand; IFRDCC: International Fungal Research and Development Culture Collection, Kunming, China; KUMCC: Kunming Institute of Botany, Kunming, China; JZB: Culture collection of Institute of Plant and Environment Protection, Beijing, China; LC: Working collection of Lei Cai, housed at Institute of Microbiology, Chinese Academy of Sciences, Beijing, China; LGMF, Laboratório de Genética de Microrganismos (LabGeM) culture collection, at the Federal University of Paraná, Brazil; MAFF: Ministry of Agriculture, Forestry and Fisheries, Tokyo, Japan; MFLU: Mae Fah Luang University herbarium, Thailand; MFLUCC: Mae Fah Luang University Culture Collection, Chiang Rai, Thailand; NCYU: Department of Plant Medicine, National Chiayi University, Chiayi, Taiwan; NTUPPMCC: Department of Plant Pathology and Microbiology, National Taiwan University Culture Collection, PMM: collection of Providence Moyo at the University of Stellenbosch, Stellenbosch, South Africa; PSCG: Personal Culture Collection Y.S. Guo, China; SAUCC: Shandong Agricultural University Culture Collection, Shandong, China; SCHM: Mycological Herbarium of South China Agricultural University, Guangzhou, China; SDBR-CMU: Culture Collection of Sustainable Development of Biological Resources Laboratory at Chiang Mai University, Chiang Mai, Thailand; URM: Culture Collection at the Universidade Federal de Pernambuco, Recife, Brazil; VTCC: Vietnam Type Culture Collection, Center of Biotechnology, Vietnam National University, Hanoi, Vietnam; ZHKUCC: Culture Collection of Zhongkai University of Agriculture and Engineering, Guangzhou, China. ^T^ indicates ex-type material. ^2^ITS: internal transcribed spacers and intervening 5.8S nrDNA; *tub2*: partial β-tubulin gene; *his3*: partial histone H3 gene; *tef1*: partial elongation factor 1-alpha gene; *cal*: partial calmodulin gene.

### ﻿Molecular phylogenetic inference

**Figure 1.**
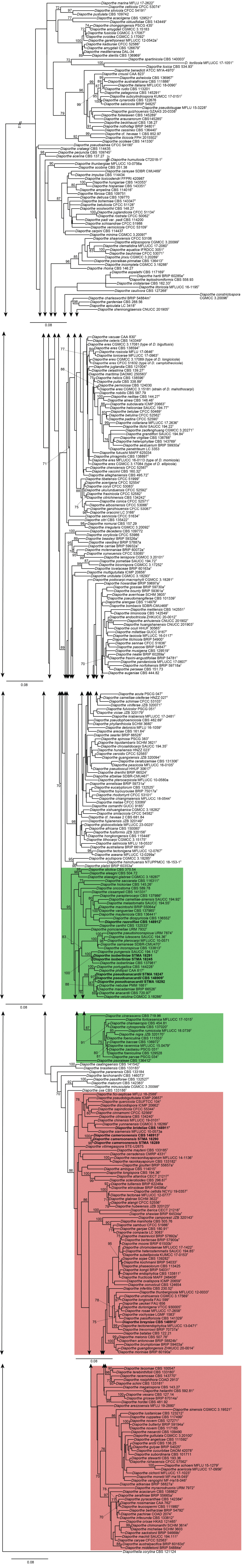
Maximum Likelihood phylogram (lLn = -56509.790498) obtained from the combined ITS, *cal*, *his3*, *tef1* and *tub2* sequences of our strain and reference strains of *Diaporthe* spp. *Diaporthellacorylina* CBS 121124 was used as outgroup. Bootstrap support values ≥70 are indicated along branches. Branch lengths are proportional to distance. Figure legend refers to nucleotide substitutions per site.

To further put the sampled strains and obtained sequences into their taxonomic context, a molecular phylogenetic inference using the taxon selection and program settings presented by Matio Kemkuignou et al. (2022) was performed. Briefly, five MAFFT alignments (Katoh and Standley 2013) were calculated featuring all surveyed sequences of the respective loci (Table 1) and curated by using Gblocks (Talavera and Castresana 2007; see Suppl. material 1: table S1). Maximum-likelihood (ML) analysis using RAxML (-HPC BlackBox v8.2.12 with default parameters, Stamatakis 2014) as implemented in the CIPRES portal (www.phylo.org) was performed for the combined aligned data, which was obtained concatenating the single locus alignments in SequenceMatrix 1.8 (Vaidya et al. 2011). The phylogenetic tree is shown in Fig. 1. After evaluation of the inferred tree, the alignment was then split into two sections (Fig. 1 shown in reddish-pink and green) and re-aligned using MAFFT. Instead of automatic filtering for conserved positions, alignments were now manually curated, correcting for alignment mistakes and subjected to the earlier described maximum-likelihood molecular phylogenetic inference using IQTree, with the option to approximate Bayesian posterior probability values (-abayes). In addition, single locus trees were calculated and checked visually for congruence with the multi-locus phylogenetic inference among the closest related sequences clustering with the here reported sequences. Support values regarded as significant (bootstrap (bs) >70%; posterior probabilities (pp) >95%) were mapped on the final maximum likelihood tree for each analysis. All alignments are deposited in the supplementary material; all used sequences, as well as the GenBank numbers for the newly generated ones, can be found in Table 1.

## ﻿Results

The lengths of the fragments of the five loci used in the combined dataset were 458 bp (ITS), 331 bp (*cal*), 296 bp (*his3*), 157 bp (*tef1*) and 510 bp (*tub2*). The length of the final alignment was 1752 bp. The phylogenetic tree obtained from the RAxML analysis of the combined dataset is shown in Fig. 1. In this tree our endophytic strains isolated from different Cameroonian host plants were located within a clade considered to represent the genus *Diaporthe*. As the surveyed isolates clustered in two larger clades of *Diaporthe*, the subsequent molecular phylogenetic inference was split into two to allow for a more accurate and efficient analysis.

**Figure 2.**
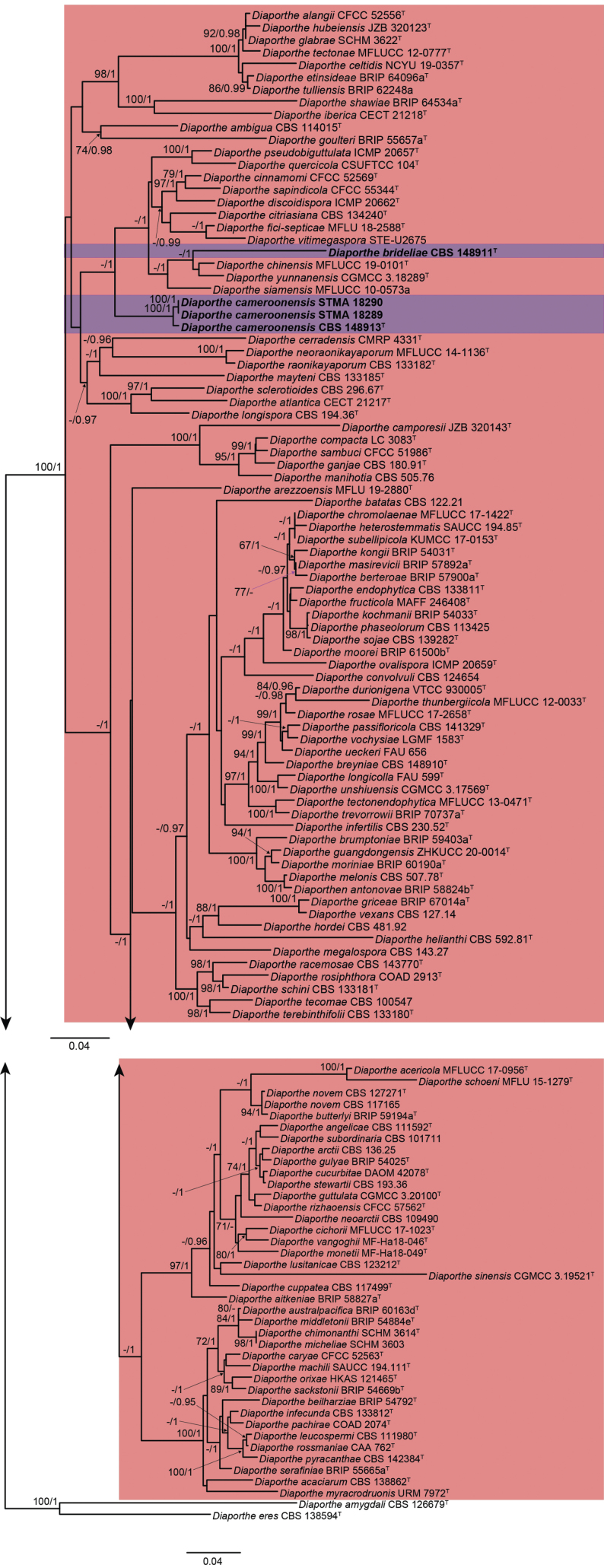
Maximum-Likelihood phylogram (lLn = -13511.2844) obtained from the combined ITS, *cal*, *his3*, *tef1* and *tub2* sequences of our strain and related *Diaporthe* spp. *Diaportheamygdali* CBS 126679^T^ and *D.eres* CBS 138594^T^ were used as outgroup. Bootstrap support values ≥ 70/Bayesian posterior probability scores ≥ 0.95 are indicated along branches. Branch lengths are proportional to distance. Novelties are indicated in **bold**. Type material of the different species is indicated with ^T^. Figure legend refers to nucleotide substitutions per site.

**Figure 3.**
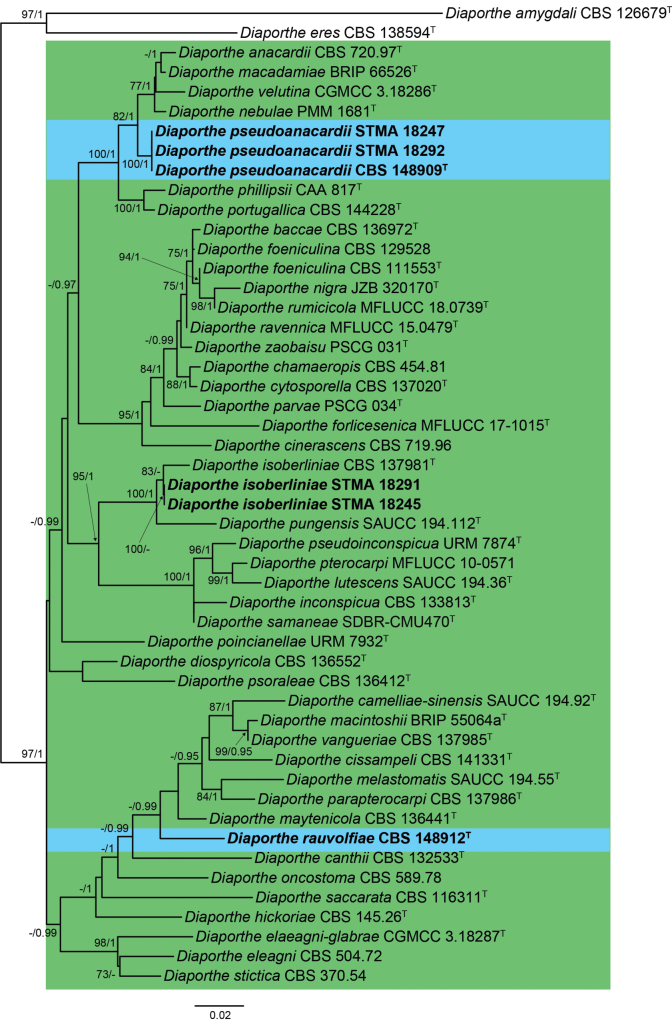
Maximum Likelihood phylogram obtained (lLn = -10678.2613) from the combined ITS, *cal*, *his3*, *tef1* and *tub2* sequences of our strain and related *Diaporthe* spp. *Diaportheamygdali* CBS 126679^T^ and *D.eres* CBS 138594^T^ were used as outgroup. Bootstrap support values ≥ 70/Bayesian posterior probability scores ≥ 0.95 are indicated along branches. Branch lengths are proportional to distance. Novelties and emended taxa are indicated in **bold**. Type material of the different species is indicated with ^T^. Figure legend refers to nucleotide substitutions per site.

The first restricted clade analysis featured 561 bp (ITS), 453 bp (*cal*), 373 bp (*his3*), 434 bp (*tef1*), 820 bp (*tub2*) for each respective locus, spanning in total 121 taxa and 2641 sites in total (Fig. 2). The second restricted clade analysis, on the other hand, resulted in an alignment featuring 550 bp (ITS), 426 bp (*cal*), 400 bp (*his3*), 362 bp (*tef1*), 719 bp (*tub2*) for each respective locus, consisting of, in total, 49 taxa and 2457 sites (Fig. 3). The first analysis showed the formation of two large clades in sister position to each other (100 bs / 1 pp), in which the isolates STMA 18289, STMA 18290, CBS 148913 and CBS 148911 clustered within an unsupported smaller one. The former three strains formed a well-supported clade (100 bs /1 pp), while strain CBS 148911 was located in an independent lineage apart from the other *Diaporthe* spp. The second phylogenetic inference revealed two highly supported clades, in which strains STMA 18291 and STMA 18245, and STMA 18247, STMA 18292 and CBS 148909 nested in, respectively. The first two resolved close to *D.isoberliniae* (100 bs / 1 pp), while the latter formed a well-supported clade (82 bs / 1 pp) within a cluster formed by *D.anacardii*, *D.macadamiae*, *D.nebulae* and *D.velutina*. The position of strain CBS 148912 did not receive bootstrap support, but was located in an independent lineage showing a higher nucleotide difference compared to other closely related species.

### ﻿Taxonomy

#### ﻿ Diaporthe brideliae

Taxon classificationFungiDiaporthalesDiaporthaceae

L. Schweizer, C. Lamb. & Y. Marín sp. nov.

DBAE24AF-3A20-543B-B97A-0BBE8EAF2E3C

 843234

Fig. 4

##### Etymology.

Name refers to the host genus that this fungus was isolated from, *Bridelia*.

**Figure 4.**
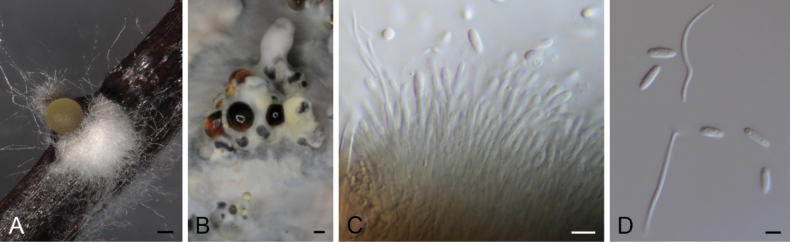
*Diaporthebrideliae* (ex-type strain CBS 148911) **A** conidioma in PNA**B** conidiomata in OA**C** conidiophores and conidia **D** alpha and beta conidia. Scale bars: 100 μm (**A**); 500 μm (**B**); 5 μm (**C, D**).

##### Description.

Conidiomata pycnidial in culture on PNA, globose or irregular, dark brown to black, solitary or in groups, embedded, erumpent, 240–500 μm diam, white to cream or yellow conidial drops exuded from ostioles; conidiomatal wall pale olivaceous green to brown, composed of 1–3 layers, textura angularis. Conidiophores cylindrical to subcylindrical, base pale olivaceous to pale yellow, apex hyaline to subhyaline, straight, densely aggregated, smooth-walled, aseptate or 1(–2) septate, (6–)12–22.5 × 1–3 μm. Conidiogenous cells phialidic, cylindrical, tapering towards the apex, hyaline, mostly terminal, rarely lateral, (7–)8–15.5 × 1–3 μm. Paraphyses not observed. Alpha conidia ovoid to ellipsoidal, hyaline, apex acutely rounded, base acutate, biguttulate, aseptate, (3–)4–6.5 × 1.5–2.5 μm. Beta conidia filiform, curved, tapering towards apex, hyaline, not guttulate, aseptate, 18–32.5 × 1–2 μm. Gamma conidia not observed.

##### Culture characters.

Colonies on PDA covering the surface of the Petri dish in 2 weeks, grayed white (156B–C) with a grayed orange (174B) ring and grayed orange (163A) margins, velvety to cottony, flat to raised in some zones, margins filamentous to fimbriate; reverse center gray brown (199A) with a yellow orange or grayed orange (167A) zones. Colonies on MEA covering the surface of the Petri dish in 2 weeks, yellow green (153B) with white to grayed yellow (160C) margins, velvety to cottony, flat to raised in some zones, margins filamentous to fimbriate; reverse black (202A) with gray brown (199A) mycelia and yellow green (153B) margins. Colonies on OA covering the surface of the Petri dish in 2 weeks, grayed green (198B) to white mycelium with a yellow green (151B) ring, cottony, flat to raised in some zones, margins filamentous; reverse yellow green (153B) with grayed yellow (161C) margins.

##### Specimen examined.

Cameroon, Kala Mountain, from *Brideliandellensis*, 03 Jan. 2019, *S.C.N. Wouamba* (holotype: CBS H-24921, culture ex-type CBS 148911 = STMA 18286).

##### Notes.

*Diaporthebrideliae* is the only report in *Bridelia* (Phyllanthaceae) from Cameroon. The phylogenetically most related species are *D.chinensis*, *D.siamensis* and *D.yunnanensis*. *Diaporthechinensis* can be distinguished by the absence of beta conidia, which are produced by the other three species. *Diaporthesiamensis* is the only species mentioned here that produces gamma conidia (Udayanga et al. 2012b). *Diaporthebrideliae* can be distinguished from *D.yunnanensis* by the production of smaller conidiomata (up to 500 μm diam in *D.brideliae* vs. 880 μm diam in *D.yunnanensis*).

#### ﻿ Diaporthe cameroonensis

Taxon classificationFungiDiaporthalesDiaporthaceae

L. Schweizer, C. Lamb. & Y. Marín sp. nov.

7C011D2C-17C4-5063-83A9-9AEB756FC7D2

 843235

Fig. 5

##### Etymology.

Named for the country where it was isolated from, Cameroon.

**Figure 5.**
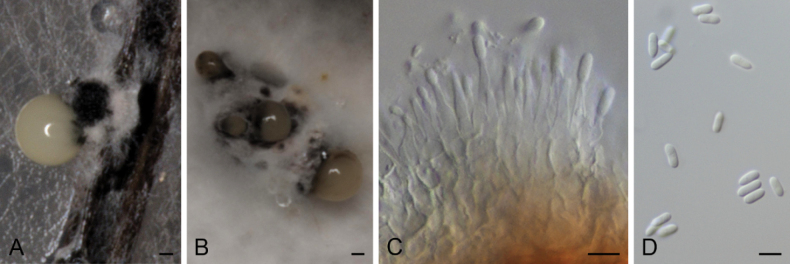
*Diaporthecameroonensis* (ex-type strain CBS 148913) **A** conidioma in PNA**B** conidiomata in OA**C** conidiophores and conidia **D** alpha conidia. Scale bars: 100 μm (**A**); 500 μm (**B**); 5 μm (**C, D**).

##### Description.

Conidiomata pycnidial in culture on PNA, globose or irregular, dark brown to black, solitary or in groups, embedded, erumpent, 220–550 μm diam, white to cream conidial drops exuded from ostioles; conidiomatal wall pale olivaceous green to olivaceous brown, composed of 1–3 layers, textura angularis. Conidiophores cylindrical to subcylindrical, tapering towards apex, base subhyaline to pale yellow or pale olivaceous, apex hyaline to subhyaline, straight, densely aggregated, smooth-walled, 1(–3) septate, 12.5–28 × 1–3.5 μm. Conidiogenous cells phialidic, cylindrical to subcylindrical, tapering towards apex, hyaline, terminal, 6–11(–12) × 1.5–3 μm. Paraphyses not observed. Alpha conidia ellipsoidal, hyaline, apex rounded, base rounded to slightly acutate, biguttulate, aseptate, 4.5–6 × (1–)1.5–2.5 μm. Beta and gamma conidia not observed.

##### Culture characters.

Colonies on PDA covering the surface of the Petri dish in 2 weeks, grayed yellow (161C–D) with transparent margins and white mycelia, cottony to slightly feathery, flat to raised in some zones, lobate, margins filamentous to fimbriate; reverse grayed yellow (161A–D) with transparent margins. Colonies on MEA covering the surface of the Petri dish in 2 weeks, grayed white (156A–B) with transparent margins and yellow white (158D) mycelia, or grayed-orange (165A) with white mycelia and yellow green (153D) margins, cottony to slightly feathery, flat to raised in some zones, margins filamentous to fimbriate; reverse grayed yellow (161A–D) with transparent margins or grayed orange (165A–B) with yellow green (153D) margins. Colonies on OA covering the surface of the Petri dish in 2 weeks, white with grayed white (156C) patches and grayed green (197D) or gray brown (199D) margins, or yellow green (152B) with brown (200A) patches and yellow-white (158A) mycelia, cottony to slightly feathery, flat to raised in some zones, margins filamentous to fimbriate; reverse grayed green (195A) with yellow green centre (152C) or fully yellow green (152C–D).

##### Specimens examined.

Cameroon, Kala Mountain, from *Atractogynegabonii*, 02 Jan. 2019, *E. G. M. Anoumedem* (holotype CBS H-24922; culture ex-type CBS 148913 = STMA 18288); from *Tremaguineensis*,11 Apr. 2019, *E. G. M. Anoumedem* (STMA 18289); from *Tremaguineensis*, 11 Apr. 2019, *E. G. M. Anoumedem* (STMA 18290).

##### Notes.

Different strains belonging to this new species formed a well-supported independent clade (100 bs / 1 pp) apart from all surveyed *Diaporthe* spp. This species was isolated from *Trema* (Cannabaceae) and *Atractogyne* (Rubiaceae). To the best of our knowledge, this is the first *Diaporthe* species to be isolated from *Atractogyne*. *Diaporthepseudoanacardii*, which is introduced further below, has also been isolated from *Trema* collected in Cameroon. However, both species can easily be distinguished by the length of their conidiogenous cells (12.5–28 μm in *D.cameroonensis* vs (7.5–)10–45 μm in *D.pseudoanacardii*) and conidia (4.5–6 μm in *D.cameroonensis* vs (5–)6–8(–9) μm in *D.pseudoanacardii*).

#### ﻿ Diaporthe pseudoanacardii

Taxon classificationFungiDiaporthalesDiaporthaceae

L. Schweizer, C. Lamb. & Y. Marín sp. nov.

F6416B15-FDAE-5E1B-A439-1FD3C73B7427

 843236

Fig. 6

##### Etymology.

Named after its close phylogenetic relation to *Diaportheanacardii*.

**Figure 6.**
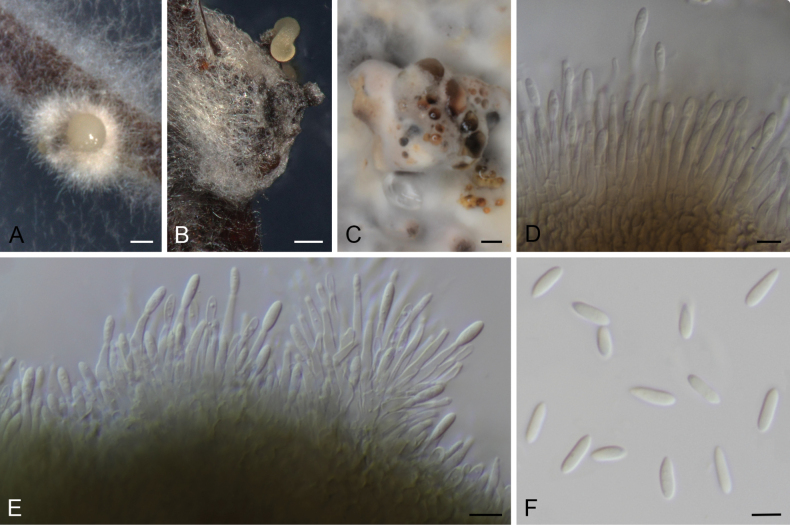
*Diaporthepseudoanacardii***A, B** conidioma in PNA**C** conidiomata in OA**D, E** conidiophores and conidia **F** alpha conidia **A, C, E, F** ex-type strain CBS 148909 **B, D** STMA 18292. Scale bars: 200 μm (**A, B**); 1000 μm (**C**); 5 μm (**D–F**).

##### Description.

Conidiomata pycnidial in culture on PNA, globose or irregular, dark brown to black, solitary or in groups, embedded, erumpent, 190–700(–820) μm diam, white to yellow or cream conidial drops and cirrus exuded from ostioles; conidiomatal wall pale olivaceous to olivaceous brown, composed of 1–2 layers, textura angularis. Conidiophores cylindrical to subcylindrical, base subhyaline to pale yellow or pale olivaceous, apex hyaline to subhyaline, straight, densely aggregated, smooth-walled, 1–2(–3) septate, rarely aseptate, (7.5–)10–45 × 1–3.5(–4) μm. Conidiogenous cells phialidic, cylindrical, tapering towards apex, hyaline to subhyaline, terminal or lateral, 7–28 × 1–3.5(–4) μm. Paraphyses not observed. Alpha conidia ovoid to ellipsoidal, hyaline, apex acutely rounded, base acutate, granular to guttulate, aseptate, (5–)6–8(–9) × 1.5–3 μm. Beta and gamma conidia not observed.

##### Culture characters.

Colonies on PDA covering the surface of the Petri dish in 2 weeks, white to grayed yellow (162C–D) or grayed white (156A–B), sometimes with transparent margins and white, yellow green (153B–C) and grayed green (195A–B) zones, granulous to cottony or slightly feathery, flat to raised in some zones, margins filamentous to fimbriate; reverse grayed yellow (161C–D or 162D) and brown (200A) or black (202A–B) center, sometimes with transparent margins. Colonies on MEA reaching 59–85 in 2 weeks, white or grayed yellow (161B–C) with normally a white ring, sometimes with grayed green zones (197A–D) and transparent margins, cottony to slightly feathery, lobate, flat to raised in some zones, margins filamentous to fimbriate; reverse grayed green (197A) to brown (200A) with grayed yellow (161B) margins, or grayed green (197A) with grayed yellow (160D) and yellow green (152B) zones and black (202A) margin, or grayed yellow (161 A–B) and transparent margins. Colonies on OA covering the surface of the Petri dish in 2 weeks, grayed green (195A–D) with white margins and yellow (4A–B) or grayed yellow (160D) center, or grayed white (156A–C) with grayed orange (163B–C) center and yellow white (158B–C) margins, cottony to slightly feathery, raised, margins filamentous to fimbriate; reverse yellow green (147B) with gray brown (199B) margins or entire gray brown (199A–B) or grayed green (195A with 198A centre).

##### Specimens examined.

Cameroon, Kala Mountain, from *Tremaguineensis*, 11 Apr. 2019, *E.G.M. Anoumedem* (holotype CBS H-24923; culture ex-type CBS 148909 = STMA 18283); Tonga, West Region, from *Pittosporummanii*, 19 Jun. 2019, *E.G.M. Anoumedem* (STMA 18247, STMA 18292).

##### Notes.

This species resolved in a well-supported clade (82 bs / 1 pp) together with *D.anacardii*, *D.macadamiae*, *D.nebulae* and *D.velutina*. *Diaporthepseudoanacardii* can be easily distinguished from all the other species by the absence of beta conidia. All these species are reported from Africa (Gomes et al. 2013; Lesuthu et al. 2019; Wrona et al. 2020), except of *D.velutina*, which was found in Asia (Gao et al. 2017).

#### ﻿ Diaporthe rauvolfiae

Taxon classificationFungiDiaporthalesDiaporthaceae

Y. Marín, C. Lamb., Kouam & L. Schweizer sp. nov.

6557A14B-6F0B-5227-8FBD-5F45AECD35CF

 843237

Fig. 7

##### Etymology.

Name refers to the host genus that this fungus was isolated from, *Rauvolfia*.

**Figure 7.**
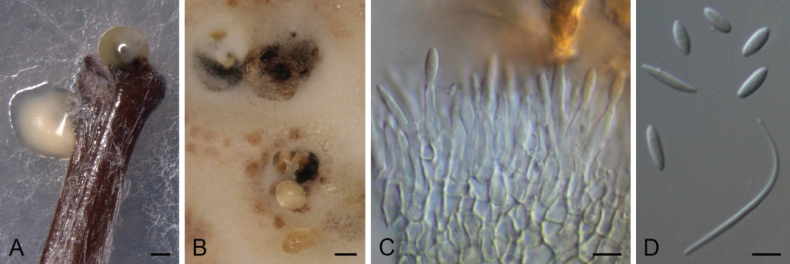
*Diaportherauvolfiae* (ex-type strain CBS 148912) **A** conidioma in PNA**B** conidiomata in OA**C** conidiophores and conidia **D** alpha, beta and alpha conidia. Scale bars: 200 μm (**A**); 500 μm (**B**); 5 μm (**C, D**).

##### Description.

Conidiomata pycnidial in culture on PNA, globose or irregular, dark brown to black, solitary or in groups, embedded, erumpent, 210–450(–530) μm diam, white to cream conidial drops exuded from ostioles; conidiomatal wall yellowish brown to olivaceous brown or brown, composed of 1–2 layers, textura angularis. Conidiophores cylindrical to subcylindrical, tapering towards apex, base subhyaline to pale yellow or pale olivaceous, apex hyaline to subhyaline, densely aggregated, smooth-walled, (0–)1–2 septate, 9–19.5 × 1.5–3.5 μm. Conidiogenous cells phialidic, cylindrical to subcylindrical, tapering towards apex, hyaline, mostly terminal, 6.5–13.5 × 1.5–3 μm. Paraphyses not observed. Alpha conidia broadly fusiform to obovoid, hyaline, apex rounded or acute, base acutate, biguttulate to multiguttulate, aseptate, 6.5–9 × 2–3 μm. Beta conidia filiform, curved, tapering towards apex, hyaline, not guttulate, aseptate, 20–36.5 × 1–2 μm. Gamma conidia less frequent, fusiform to obovoid, straight to slightly curved, rarely sinuose, acutate ends or one acutate and other round, hyaline, multiguttulate, aseptate, (8–)9–13 × 1.5–2.5 μm.

##### Culture characters.

Colonies on PDA reaching 72–76 mm in 2 weeks, grayed yellow (160B–C) with white ring and transparent margins, cottony to slightly feathery, raised, lobate, margins filamentous; reverse grayed yellow (160B–C) with white ring and transparent margins. Colonies on MEA covering the surface of the Petri dish in 2 weeks, white to grayed white (156B), cottony to slightly feathery, raised, margins filamentous; reverse grayed green (197A) to gray brown (199A) with black (202A) center. Colonies on OA covering the surface of the Petri dish in 2 weeks, grayed white (156B–D), cottony to slightly feathery, raised, margins filamentous; reverse gray brown (199A).

##### Specimen examined.

Cameroon, Tonga, West Region, from *Rauvolfiavomitoria*, 19 Jun. 2019, *E.G.M. Anoumedem* (holotype CBS H-24924, culture ex-type CBS 148912 = STMA 18287).

##### Notes.

*Diaportherauvolfiae* was located in an independent branch far from other species of *Diaporthe* (Fig. 3). This species is characterized by the production of alpha, beta and gamma conidia, which were not observed in other species reported from Cameroon except of *D.isoberliniae*. This latter species differs from *D.rauvolfiae* in the length of the conidiophores (13–42 μm in *D.isoberliniae* vs 9–19.5 μm in *D.rauvolfiae*), beta conidia (11.5–27.5 μm in *D.isoberliniae* vs 20–36.5 μm in *D.rauvolfiae*) and gamma conidia (10–18.5(–21) μm in *D.isoberliniae* vs (8–)9–13 μm in *D.rauvolfiae*). Both species are not phylogenetically related (Fig. 3).

#### ﻿ Diaporthe isoberliniae

Taxon classificationFungiDiaporthalesDiaporthaceae

Crous, Persoonia 32: 221. 2014. emend. L. Schweizer, C. Lamb. & Y. Marín

4F731F40-4DE0-527A-841A-A0BD3F50A38A

 808909

Fig. 8

##### Description.

Conidiomata pycnidial in culture on PNA, globose or irregular, dark brown to black, solitary or in groups, embedded, erumpent, 200–460 μm diam, white to cream or yellow conidial drops exuded from ostioles; conidiomatal wall yellowish brown to olivaceous brown or brown, composed of 1–6 layers, textura angularis. Conidiophores cylindrical to subcylindrical, base subhyaline to pale olivaceous, apex hyaline, densely aggregated, smooth-walled, 1–3-septate, 13–42 × 1.5–4 μm. Conidiogenous cells phialidic, cylindrical to subcylindrical, tapering towards apex, hyaline, terminal or lateral, (5.5–)6.5–14 × 1.5–3 μm. Paraphyses not observed. Alpha conidia ellipsoidal to obovoid, or fusoid-ellipsoid, hyaline, apex rounded or subobtuse, base acutate or subtruncate, biguttulate to multiguttulate, aseptate, 5.5–9(–10) × 2–3(–3.5) μm. Beta conidia less frequent, filiform, curved, tapering towards apex, hyaline, not guttulate, aseptate, 11.5–27.5 × 1–2 μm. Gamma conidia less frequent, broadly fusiform, straight to slightly curved, rarely sinuose, apex acutate or filiform, base filiform, hyaline, multiguttulate, aseptate, 10–18.5(–21) × 1.5–2.5 μm.

**Figure 8.**
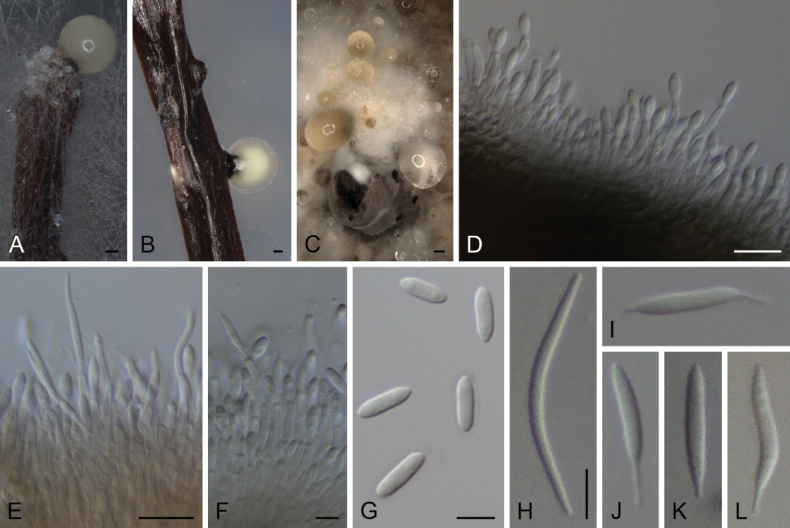
*Diaportheisoberliniae***A, B** conidioma in PNA**C** conidiomata in OA**D–F** conidiophores and conidia **G** alpha conidia **H** beta conidia **I–L** Gamma conidia **A, C, D–I** STMA 18291 **B, J–L** STMA 18245. Scale bars: 100 μm (**A, B**); 500 μm (**C**), 10 μm (**D, E**), 5 μm (**F–L**).

##### Culture characters.

Colonies on PDA reaching 63–72 mm or covering the surface of the Petri dish in 2 weeks, white with a grayed yellow (160C) ring and transparent margins, lobate, cottony to slightly feathery, flat to raised in some zones or fully raised, lobate, margins filamentous to fimbriate; reverse grayed yellow (160B–D). Colonies on MEA covering the surface of the Petri dish in 2 weeks, grayed yellow (161A) with a white ring and white to transparent margins, cottony to slightly feathery, flat to raised in some zones or fully raised, margins filamentous to fimbriate; reverse grayed yellow (162A–C) with transparent margins and sometimes with gray brown (199A) center. Colonies on OA covering the surface of the Petri dish in 2 weeks, white to grayed white (156A) with grayed yellow (161A–B) margins or grayed yellow (161C) with brown (200A) dots and white center and margins, cottony to slightly feathery, raised, margins filamentous to fimbriate; reverse grayed green (197B) to/or gray brown (199C–D).

##### Specimens examined.

Cameroon, Tonga, West Region, from *Pittosporummanii*, 19 Jun. 2019, *E. G. M. Anoumedem* (STMA 18245); *ibid.* STMA 18291.

##### Notes.

*Diaportheisoberliniae* was described based on a specimen isolated from Zambia on *Isoberliniaangolensis* (Fabaceae) (Crous et al. 2014b). To the best of our knowledge, this species had not been recollected since then. We isolated two strains belonging to *D.isoberliniae* from Cameroon on *Pittosporummanii* (Pittosporaceae). The description of this species is here emended with beta and gamma conidia, as the shared observations are the first to report on them. The isolate STMA 18245 did not produce beta conidia, but produced gamma conidia, while isolate STMA 18291 produced beta conidia more frequently than gamma conidia. The type strain produced fusoid-ellipsoid alpha conidia of similar sizes, while these are ellipsoid to obovoid in our two Cameroonian strains.

*Diaportheisoberliniae* is related to *D.pungensis*. This latter species can be distinguished by the absence of gamma conidia and the production of shorter conidiophores (11–14.5 μm in *D.pungensis* vs 13–42 μm in *D.isoberliniae*) (Sun et al. 2021).

## ﻿Discussion

This study reports on the isolation and assignment of a group of fungi isolated from plant material to the genus *Diaporthe*. A characterization by sequencing was followed-up with a concatenation-based molecular phylogenetic inference, which afforded heterogenous sequence placements among a phylogenetic dataset featuring DNA sequence data substantially derived from type strains. Taken together with an analysis of the taxon placements in single-locus trees (data not shown), we concluded that the placement pattern among each strain was unique, which combined with the traditional morphological descriptions let us to propose the erection of four new species to accommodate the isolated strains. Secluding species description to either morphology, ecological observations (such as host occurrence or lifestyle) or molecular data alone has been shown to be problematic in *Diaporthe*, indicating that the commonly observed morphological features that are recorded and observed by taxonomists are not under strict evolutionary selection pressure (Gao et al. 2015, Fan et al. 2018). Additionally, only a limited set of loci are sequenced even for typified *Diaporthe* spp., which act as a strong limiting factor for comprehensive molecular phylogenetic analysis. That this undersampling may become problematic is best exemplified by highlighting that the ITS region, long treated as unequivocal fungal barcode, is a poor choice for species delimitation and should be utilized with caution. More specifically, multiple copies may occur in fungal genomes potentially showing significant differences, which, if accidentally sequenced independent from each other, may erroneously lead taxonomists to treat specimens, which are in truth only one species, as separate ones. The fact that this scenario is plausible for *Diaporthe* has recently been reported by Hilário et al. (2021), who found multiple ITS paralogues for a newly sequenced genome of *D.novem*. A similar finding was already described and discussed for the xylarialean genus *Hypoxylon* (Stadler et al. 2020). Most interestingly, Hilário et al. (2021) reported indications for a hybrid species, which could, if this was also assumed to be the case for other members of the genus as well, explain the convoluted systematic status of *Diaporthe* in its current form (Fan et al. 2018). This makes it all the more necessary to treat molecular data cautiously, especially since not all loci commonly used to infer phylogenies are well covered across all described species of *Diaporthe*. A careful in-depth phylogenetic analysis of species assigned to the *D.amygdali* “complex”, rigorously applying Genealogical Concordance Phylogenetic Species Recognition (GCPSR) and coalescence based evolutionary principles by Hilário et al. (2021), for example, showed an unexpectedly high genetic heterogeneity. This complex consisted of seven species forming nine statistically supported clades in concatenation-based phylogenies of ITS, *cal*, *his3*, *tef1 and tub2* – loci also used in this paper – which, however, could not be resolved into reasonably distinct lineages representing individual species in either single-locus trees, or coalescent-based analysis. While this hopefully remains an extreme example inside the systematics of *Diaporthe*, this finding clearly shows that 1) adding new species based on single-locus sequencing will further destabilize *Diaporthe* taxonomy and 2) molecular phylogeneticists have to be aware of the possibility that the inferred supermatrix tree may not reflect the evolutionary history of the underlying loci, possibly leading to artificial lineage resolutions. A later study by Norphanphoun et al. (2022) attempted to classify a large collection of *Diaporthe* species into molecularly distinctly resolving species complexes. They showed that different loci harbored distinct resolution power for members of each complex (Norphanphoun et al. 2022) – a phenomenon that we also observed for our strains (data not shown) – which raises doubt that molecular data alone is enough to justify the classification of species complexes, even if it is just for the mere purpose of easing communication. In our study, we followed a polyphasic strategy combining multi-locus sequencing with morphological characterization, which clearly showed that the collected strains are separable by multiple phenotypic traits – a necessity in this convoluted genus. Given that intraspecific variation is repeatedly shown to be unexpectedly high, this should be complemented by screening for additional well-established discriminative characters, such as secondary metabolite production for chemotaxonomic purposes in the future. Furthermore, aiming for the generation of high-quality genome sequences would enable studies on the genetics governing ecology, lifestyle and speciation. The genomic toolbox to meaningfully embark on this has already been established for other complicated genera such as *Penicillium* and *Aspergillus* (Frisvad and Larsen 2015, Kocsubé et al. 2016, Tsang et al. 2018). This would help to find reasons for the frequently reported paraphyly, poor phylogenetic resolution and by consequence, enable the establishment of sound species boundaries for the inevitable revision of the genus (Dissanayake 2017; Gao et al. 2017; Marin-Felix et al. 2019; Hilário et al. 2021). A recent study published by Hongsanan et al. (2023) formalized necessary taxonomic changes with data that is already available today, indicating the huge potential of a more sophisticated follow-up analysis. Lastly, an additional epitypification campaign is imperative to further stabilize the taxonomy of *Diaporthe* and allies, hopefully enabling species differentiation between saprobes and important phytopathogens for e.g. diagnostic purposes.

## Supplementary Material

XML Treatment for Diaporthe brideliae

XML Treatment for Diaporthe cameroonensis

XML Treatment for Diaporthe pseudoanacardii

XML Treatment for Diaporthe rauvolfiae

XML Treatment for Diaporthe isoberliniae

## ﻿Additional information

### Author ORCIDs

Christopher Lambert https://orcid.org/0000-0002-1899-8214

Lena Schweizer https://orcid.org/0000-0003-1296-5486

Simeon F. Kouam https://orcid.org/0000-0003-0191-0527

Yasmina Marin-Felix https://orcid.org/0000-0001-8045-4798

### Data availability

All of the data that support the findings of this study are available in the main text or Supplementary Information.

## Supplementary materials

Supplementary material 1 Phylogenetic study data Data type docx https://binary.pensoft.net/file/925263This dataset is made available under the Open Database License (http://opendatacommons.org/licenses/odbl/1.0/). The Open Database License (ODbL) is a license agreement intended to allow users to freely share, modify, and use this Dataset while maintaining this same freedom for others, provided that the original source and author(s) are credited.Christopher Lambert, Lena Schweizer, Blondelle Matio Kemkuignou, Elodie Gisèle M. Anoumedem, Simeon F. Kouam, Yasmina Marin-Felix
