# Supplementary material for: ﻿Four new endophytic species of Diaporthe (Diaporthaceae, Diaporthales) isolated from Cameroon
Source: MycoKeys. 2023 Oct 23;99:319–62. doi: 10.3897/mycokeys.99.110043 (PMC10616871; doi:10.3897/mycokeys.99.110043)
Supplement: Supplementary material 1 — Phylogenetic study data [file mycokeys-99-319-s001.docx]

**Four new endophytic species of *Diaporthe* (Diaporthaceae, Diaporthales) isolated from Cameroon**

Christopher Lambert^1,2,3^, Lena Schweizer^1^, Blondelle Matio Kemkuignou^1,2^, Elodie Gisèle M Anoumedem^4^, Simeon F. Kouam^4^, Yasmina Marin-Felix^1,2,^*

^1^Department of Microbial Drugs, Helmholtz Centre for Infection Research (HZI) and German Centre for Infection Research (DZIF), Partner Site Hannover/Braunschweig, Inhoffenstrasse 7, 38124 Braunschweig, Germany.

^2^Institute of Microbiology, Technische Universität Braunschweig, Spielmannstraße 7, 38106 Braunschweig, Germany.

^3^Molecular Cell Biology Group, Helmholtz Centre for Infection Research (HZI), Inhoffenstrasse 7, 38124 Braunschweig, Germany.

^4^Department of Chemistry, Higher Teacher Training College, University of Yaoundé I, Yaoundé P.O. Box 47, Cameroon.

*Corresponding author

[Yasmina.MarinFelix@helmholtz-hzi.de](mailto:Yasmina.MarinFelix@helmholtz-hzi.de)

Contents

[Table S1: Flanking positions of gblocks curated MAFFT alignments of the first phylogenetic analysis. Characteristics of the alignments subsequently used for multigene phylogenetic inference using IQTree 2. 3](#_Toc141194694)

[Table S2: Characteristics of the first restricted MAFFT alignments used for phylogenetic analysis with IQTree 2. 3](#_Toc141194695)

[Table S3: Selected edge-linked proportional partition substitution models of the first restricted phylogenetic inference subjected to IQTree2, calculated with ModelFinder, using Bayesian information criterion (BIC). 4](#_Toc141194696)

[Table S4: Characteristics of the first restricted MAFFT alignments used for phylogenetic analysis with IQTree 2. 4](#_Toc141194697)

[Table S5: Selected edge-linked proportional partition substitution models for the restricted phylogenetic analysis subjected to IQTree2 calculated with ModelFinder using Bayesian information criterion (BIC). 4](#_Toc141194698)

[⮚ Concatenated sequence alignments of ITS, *cal*, *his3*, *tef1* and *tub2* used for the initial phylogenetic study 5](#_Toc141194699)

[⮚ Alignment of the ITS sequences used in the first restricted phylogenetic study 402](#_Toc141194700)

[⮚ Alignment of the *cal* sequences used in the first restricted phylogenetic study 427](#_Toc141194701)

[⮚ Alignment of the *his3* sequences used in the first restricted phylogenetic study 442](#_Toc141194702)

[⮚ Alignment of the *tef1* sequences used in the first restricted phylogenetic study 453](#_Toc141194703)

[⮚ Alignment of the *tub2* sequences used in the first restricted phylogenetic study 472](#_Toc141194704)

[⮚ Alignment of the ITS sequences used in the second restricted phylogenetic study 509](#_Toc141194705)

[⮚ Alignment of the *cal* sequences used in the second restricted phylogenetic study 521](#_Toc141194706)

[⮚ Alignment of the *his3* sequences used in the second restricted phylogenetic study 527](#_Toc141194707)

[⮚ Alignment of the *tef1* sequences used in the second restricted phylogenetic study 533](#_Toc141194708)

[⮚ Alignment of the *tub2* sequences used in the second restricted phylogenetic study 540](#_Toc141194709)

# **Table S1: Flanking positions of gblocks curated MAFFT alignments of the first phylogenetic analysis. Characteristics of the alignments subsequently used for multigene phylogenetic inference using IQTree 2.**

| **DNA locus** | **Flanking positions** | **Proportion of selected positions** | **Unique** | **Infor** | **Invar** | **Const** |
| --- | --- | --- | --- | --- | --- | --- |
| **ITS** | [178-203], [205-220], [234-239], [254-258], [261-266], [268-272], [274-278], [282-287], [296-310], [315-322], [347-356], [371-376], [378-397], [399-407], [409-414], [416-433], [459-608], [749-778], [782-790], [792-804], [837-860], [871-877], [879-887], [889-895], [905-911], [921-929], [1009-1018], [1021-1025], [1044-1049], [1191-1195] | 458/1259 (36%) | 305 | 189 | 172 | 172 |
| ***cal*** | [212-218], [227-236], [250-255], [268-275], [283-287], [289-294], [306-313], [315-321], [324-357], [359-367], [403-411], [417-445], [447-455], [492-501], [506-512], [527-536], [553-559], [571-697], [733-747], [749-756] | 331/1362 (24%) | 245 | 206 | 100 | 100 |
| ***his3*** | [61-194], [291-376], [398-435], [457-494] | 296/585 (50%) | 126 | 65 | 192 | 192 |
| ***tef1*** | [152-173], [291-295], [316-322], [324-335], [368-372], [374-393], [409-414], [439-447], [460-464], [479-488], [575-581], [587-592], [678-682], [689-696], [731-736], [738-761] | 157/1252 (12%) | 147 | 113 | 19 | 19 |
| ***tub2*** | [489-493], [525-530], [543-558], [563-567], [605-611], [617-625], [636-645], [648-660], [670-717], [733-738], [748-769], [771-777], [796-826], [834-847], [937-941], [943-954], [992-1000], [1002-1009], [1024-1033], [1044-1093], [1097-1103], [1108-1113], [1120-1125], [1140-1145], [1165-1169], [1194-1201], [1203-1270], [1288-1294], [1409-1413], [1423-1431], [1483-1496], [1502-1506], [1550-1562], [1614-1636], [1688-1722] | 510/1824 (27%) | 449 | 301 | 71 | 71 |

# **Table S2: Characteristics of the first restricted MAFFT alignments used for phylogenetic analysis with IQTree 2.**

| **DNA Locus** | **Sequences** | **Sites** | **Unique** | **Informative** | **Invariant** | **Constant** |
| --- | --- | --- | --- | --- | --- | --- |
| **ITS** | 121 | 561 | 285 | 136 | 331 | 331 |
| ***cal*** | 74 | 453 | 288 | 189 | 201 | 201 |
| ***his3*** | 69 | 373 | 151 | 90 | 240 | 240 |
| ***tef1*** | 111 | 434 | 375 | 266 | 109 | 109 |
| ***tub2*** | 110 | 820 | 577 | 330 | 367 | 367 |

# **Table S3: Selected edge-linked proportional partition substitution models of the first restricted phylogenetic inference subjected to IQTree2, calculated with ModelFinder, using Bayesian information criterion (BIC).**

| **DNA Locus** | **Model** | **Speed** | **Parameters** |
| --- | --- | --- | --- |
| **ITS** | TNe+R4 | 0.7143 | TNe{3.66268,7.95852}+FQ+R4{0.687106,0.0645323,0.200509,0.957125,0.0738989,3.92157,0.0384865,12.3146} |
| ***cal*** | HKY+F+I+G4 | 0.7561 | HKY{4.74623}+F{0.212633,0.31179,0.247722,0.227856}+I{0.278139}+G4{1.2986} |
| ***his3*** | TN+F+I+I+R3 | 1.1518 | TN{0.725383,4.90722}+F{0.173687,0.374506,0.239611,0.212196}+I{0.474828}+R3{0.309156,0.191453,0.159818,2.22135,0.0561981,10.4238} |
| ***tef1*** | HKY+F+R3 | 1.9074 | HKY{2.8875}+F{0.219038,0.330181,0.207197,0.243584}+R3{0.306533,0.114168,0.375962,0.929062,0.317505,1.93922} |
| ***tub2*** | HKY+F+I+G4 | 0.7809 | HKY{3.93041}+F{0.200329,0.358431,0.22512,0.21612}+I{0.296379}+G4{1.3624} |

# **Table S4: Characteristics of the first restricted MAFFT alignments used for phylogenetic analysis with IQTree 2.**

| **DNA Locus** | **Sequences** | **Sites** | **Unique** | **Informative** | **Invariant** | **Constant** |
| --- | --- | --- | --- | --- | --- | --- |
| **ITS** | 50 | 550 | 175 | 68 | 427 | 427 |
| ***cal*** | 32 | 426 | 230 | 103 | 218 | 217 |
| ***his3*** | 34 | 400 | 97 | 46 | 325 | 325 |
| ***tef1*** | 44 | 362 | 269 | 159 | 135 | 135 |
| ***tub2*** | 49 | 719 | 344 | 132 | 442 | 442 |

# **Table S5: Selected edge-linked proportional partition substitution models for the restricted phylogenetic analysis subjected to IQTree2 calculated with ModelFinder using Bayesian information criterion (BIC).**

| Locus | **Model** | **Speed** | **Parameters** |
| --- | --- | --- | --- |
| **ITS** | K3P+I+I+R2 | 1.2001 | K3P{3.01784,0.572573}+FQ+I{0.675534}+R2{0.251561,0.622901,0.0729053,11.5671} |
| ***cal*** | TNe+G4 | 0.8419 | TNe{3.13358,6.04463}+FQ+G4{1.57017} |
| ***his3*** | TN+F+G4 | 0.5264 | TN{2.52226,9.36257}+F{0.176592,0.374271,0.241093,0.208043}+G4{0.128286} |
| ***tef1*** | TIM2e+G4 | 1.9214 | TIM2e{1.59326,3.30685,5.71558}+FQ+G4{0.826598} |
| ***tub2*** | HKY+F+G4 | 0.7402 | HKY{3.54837}+F{0.208133,0.350269,0.22751,0.214089}+G4{0.522683} |

# Concatenated sequence alignments of ITS, *cal*, *his3*, *tef1* and *tub2* used for the initial phylogenetic study

>Diaporthe_acaciarum_CBS_138862

????????????????????????????????????????????????????????????

????????????????????????????????????????????????????????????

????????????????????????????????????????????????????????????

????????????????????????????????????????????????????????????

????????????????????????????????????????????????????????????

???????????????????????????????TCCGCGCCCTCCACCGGAGGTGTCAAGAA

GCCTCACCGCTACAAGCCTGGTACCGTCGCTCTGCGTGAGATCCGTCGCTACCAGAAGAG

CACCGAGCTGCTGATCCGCAAGCTGCCCTTCCAGCGTCTGGTATGCAGGTCCGTGAGATC

GCCCAGGACTTCAAGTCCGACCTGCGTTTCCAGTCTTCCGCCATCGGTGCCCTTCAGGAG

TCCGTCGAGTCTTACCTCGTCTCCCTCTTTGAGGACACCAACCTGTGCGCCATCCACGCN

NNNNNNNNNNNNNNNNNNNNNNNNNNNNNNNNNNNNNCCGTTGGTGAACCAGCGGAGGGA

TCATTGCTGCTTC-GGCGCACCCAGAAACCCTTTGTGAACTTA--CTGTTGCCTCGGCAG

GCCGGGAGGCCCCCCGAGACGGGGAGCA-GCCCGCCGGCGGCCAACTAAACTCTTGTTTC

TACAGTGAATCTCTAAATGAATCAAAACTTTCAACAACGGATCTCTTGGTTCTGGCATCG

ATGAAGAACGCAGCGAAATGCGATAAGTAATGTGAATTGCAGAATTCAGTGAATCATCGA

ATCTTTGAACGCACATTGCGCCCCCTGGTATTCCGGGGGGCATGCCTGTTCGAGCGTCAT

TTCAACCCTCAAGCCTGGCTTGGTGATGGGGCACTAGGGAGTAGGCCCTGAAATTCAGTG

GCGAGCTCGCCAGGACCCCGAGCGTAGTAGA-TCTCGCTAAGGCCCTGGCGGTGCCCTGC

TGAAA???????????????????????????????????????????????????????

????????????????????????????????????????????????????????????

??????????????????????????????????????????TCGCACCTCAGCCCCACC

ATCGCGACCCACCCCCTGCGACACCCAAATAAGACGCGTCGATTGCTAACGTGTTTTTCT

CGCCCACAGGTTCACCTTCAGACCGGCCAATGCGTAAGTTGCTGTCACCGCCCGACCTTA

T---CGCCACCCATAGCACGTTTCCCAGGGTAACCAAATCGGTGCTGCTTTCTGGTGCGT

CCCGAGCCTCCCACCACGAGACTAGCTCGCAACATACTGATCTCGTAGGCAAACCATCTC

TGGCGAGCACGGTCTCGACAGCAATGGCGTGTATGTACCTCCTGTTCCTGGCAGTCTCGT

CCTCCCTGATGATCGCACAGTTACAACGGCACTTCCGAGCTCCAGCTCGAGCGCATGAAC

GTCTACTTCAACGAGGTAAGCACGTCTTTTTGA-CCGTCTCGGCCTTGCTAACGCGCTCT

CAGGCCTCCGGCAACAAGTATGTGCCTCGCGCCGTCCTCGTCGATCTCGAGCCTGGTACC

ATGGACGCCGTC

>Diaporthe_acaciigena_CBS_129521

CTTTGTAAGTCATTTTT------ACCGCCCTTACTGTCGCGCATGCTAACGGACCGTTTC

CGGCCTTCAGGATAAGGATGGCGATGGTTAGTGCGGCAGCTCCCACTCTAGACGCGTCAC

GATCGACCCGCCGCGACCGTCTGCGCGCGAACATGCACCATCGCTATCAGGAGTTACTAA

GGTGTAGGGCAAATCACTACAAAGGAGCTCGGCACGGTCATGCGATCCCTGGGCCAAAAC

CCTTCCGAGTCTGAGTTGCAGGACATGATCAACGAGGTCGACGCCGACAACAATGGCACC

ATCGACTTTGGTACGTCCAAATGCGTGTTCATCCGCGCCCTCCACCGGAGGTGTCAAGAA

GCCTCACCGCTACAAGCCCGGTACCGTCGCTCTGCGTGAGATCCGTCGCTACCAGAAGTC

CACCGAGCTCCTGATCCGCAAGCTGCCCTTCCAGCGTCTGGTAGGTAGGTTCGTGAGATT

GCCCAGGACTTCAAGTCCGACCTCCGCTTCCAGTCTTCCGCCATCGGTGCCCTGCAGGAG

TCCGTCGAGTCCTACCTCGTCTCCCTTTTCGAGGACACCAACCTGTGCGCCATCCACGCC

AAGCGTGTCACCATCCAGTCGGTATGTAACAAGGTCTCCGTTGGTGAACCAGCGGAGGGA

TCATTGCTGCCCCAGGCGCACCCAGAAACCCTTTGTGAACTTATACTGTTGCCTCGGCAG

GCCGGGGGGCCCCTCCGGACGAGGAGACGGCCCGCCGGCGGCCCATTTAACTCTTGTTTT

TAACCTGGAACTCTAAATGAATCAAAACTTTCAACAACGGATCTCTTGGTTCTGGCATCG

ATGAAGAACGCAGCGAAATGCGATAAGTAATGTGAATTGCAGAATTCAGTGAATCATCGA

ATCTTTGAACGCACATTGCGCCCTCCGGTATTCCGGAGGGCATGCCTGTTCGAGCGTCAT

TTCAACCCTCAAGCCTGGCTTGGTGATGGGGCACTGCGAGGCAGGCCCTGAAATTCAGTG

GCGAGCTCGCCAGGACTCCGAGCGCAGTAGACCCTCGCTAAGG-ACTGGCGGTGCCCTGC

TGAAAGAGAAGGAAGGTTAGTAAATATCCTCATGTTTGCACGGCGTCATCAGCGCATTTT

CACCCCTCGCTCTGGATTTCAGGGGTGCGGGGCTTAGAGCTTATCTCACTGGCTCAATCT

GGAACATGCTAATATCTTACCAGCCGCCGAACTTGGCAAGGGTCGCACCTGAGCCCCACT

ATCGCGACCCGCCCCCTGCGACTACCAGATGAGACGCGTCGATTGCTAACTCGTCTTCCT

CG-CAATAGGTTCACCTTCAGACCGGCCAATGCGTAAGTTGCCGTCACCAGCGCACCTTA

TCGCCGCCGACCGTAGCACGTTTCCCAGGGTAACCAAATCGGTGCTGCTTTCTGGTGCGT

C----ATCCATCGTCGCGGCACGACCTCGCAACATACTGACCTCGTAGGCAAACCATCTC

TGGCGAGCACGGCCTCGACACCAATGGCGTGTATGTACCTCCTATCCCTGCCTATCTCGA

CTCGGCTGACAACTGCACAGTTACAACGGCACTTCCGAGCTCCAGCTCGAGCGCATGAAC

GTCTACTTCAACGAGGTACGTATGTCGTTTCGACGAGCCCGAGCTTTGCTGACACGTTAT

CAGGCTTCCGGCAACAAGTATGTCCCTCGCGCCGTCCTTGTCGATCTCGAGCCCGGTACC

ATGGATGCCGTC

>Diaporthe_acericola_MFLUCC_17_0956

NNNNNNNNNNNNNNNNNNCCTCGCCCGCCATTGCCTTTGCGCATGCTAACGGACCGTTTT

CGGCCTGCAGGATAAGGATGGCGATGGTTAGTGCGGTCACTCTCAGCTACCACGCGTCAT

ACTCGATCCGCCGCGACGGTCTGCGCGTGAGCGAGCGACCTCATGATCACGAGATGCTAA

GGTGTAGGACAAATCACCACCAAGGAGCTCGGCACAGTCATGCGGTCCCTTGGTCAAAAC

CCTTCCGAGTCCGAGCTGCAGGACATGATCAACGAGGTCGACGCCGACAACAATGGCACC

ATTGACTTTGGTAAGTCTACATGTCCACCCA?????????????????????????????

????????????????????????????????????????????????????????????

????????????????????????????????????????????????????????????

????????????????????????????????????????????????????????????

????????????????????????????????????????????????????????????

???????????????????????????NNNNNNNNNNNNNNNNNNNNNNNNNNNNNNNNN

NNNNNNNNNNNNNNNNNNNNNNCCAGAACCCTTTGTG-ACTTA--CTGTTGCCTCGGCAG

GCCGGGAGGCCCCCTGAAACAGGGAGCA-GCCCGCCGGTGGCCAACTAAACTC-TGTTTC

TATAGTGAATCTCTAAATGAATCAAAACTTTCAACAACGGATCTCTTGGTTCTGGCATCG

ATGAAGAACGCAGCGAAATGCGATAAGTAATGTGAATTGCAGAATTCAGTGAATCATCGA

ATCTTTGAACGCACATTGCGCCCTCTGGTATTCCGGAGGGCATGCCTGTTCGAGCGTCAT

TTCAACCCTCAAGCCTGGCTTGGTGATGGGGCACTGAAGGGCAGGCCCTGAAATCTAGTG

GCGAGCTCGCCAGGACCCCGAGCGTAGTAGA-TCTCGCTAAGGCCCTGGCGGTGCCCTGC

TGAAANNNNNNNNNNNNNNNNNNNNNNCATTGTGCCTGCGCGGCTTCAT--AACCATTTT

CACCCCTCCCTCTGGGTTTTCAGGGTGCGGGGCTTACCGCTTATCTCACCGCCAACACCG

AATAGAAGCTGATTTCCACACAGCCGCCGAGCTGGGTAAGGGNNNNNNNNNNNNNNNNNN

NNNNNNNNNNNNNNNNNNNNNNNNNNNNNNNNNNNNNNNNNNNNNNNNNNNNNNNNNNNN

NNNNNNNNNNNNNNNNNNNNNNNNNNNNNNNNNNNNNNNNNNNNNNNNNNNNNNNNNNNN

NNNNNNNNNNNNNNNNNNNNNNNNNNNNNNNNNNNNNNNNNNNNNNNNNNNNNNNNNNNN

NNNNNNNNNNNNATCGCGACAGGACCTCGCAACATATTGACTTCGTAGGCAAACCATCTC

TGGCGAGCACGGCCTCGACAGCAATGGCGTGTATGTACCTCCCATTCCTACTCGTCTCGT

CCGCCCTGACAGCTTCACAGTTACAACGGCTCTTCTGAGCTCCAGCTCGAGCGCATGAAC

GTCTACTTCAACGAGGTCAGTTTTTTATACCCACGATCTCCAACTTTGCTGACACCTTAT

CAGGCTTCCGGCAACAAGTATGTGCCTCGCGCTGTCCTCGTCGATCTCGAGCCCGGTACC

ATGGACGCCGTC

>Diaporthe_acerigena_CFCC_52554

NNNNNNNNNNNNNNNNNNNNNNNNCCGCCCTTGCTGGTGCGCATGCTAACGGACCGTTTT

CGGCTTGTAGGATAAGGATGGCGATGGTTAGTGCGGCCGCTCTCACCTAGCACGCGTCTT

GCTCGAACCGCCGCGACAGCCTGCGCGCAACCACGCGTTATCACTATCACGAGTTGCTGA

GGTGTAGGACAAATCACCACCAAGGAGCTCGGCACGGTCATGCGGTCCCTTGGTCAGAAC

CCGTCCGAGTCTGAGCTGCAAGATATGATTAACGAGGTCGACGCCGACAACAATGGCACC

ATTGACTTTGGTACGTCCAGATGCTCGCCCTTCCGCGCCCTCCACCGGAGGTGTCAAGAA

GCCTCACCGCTACAAGCCTGGTACCGTCGCTCTGCGTGAGATCCGTCGCTACCAGAAGAG

CACTGAGCTGCTGATCCGCAAGCTCCCCTTCCAGCGTCTGGTATGCAGGTCCGTGAGATC

GCCCAGGACTTCAAGTCCGACCTCCGCTTCCAGTCTTCCGCCATCGGCGCCCTGCAGGAG

TCGGTCGAGTCTTACCTCGTCTCCCTCTTCGAGGACACCAACCTGTGCGCCATCCACGCC

AAGCGTGTCACCATCCAGTCGGTATGTNNNNNNNNNNNNNNNNNNNNNNNNNNNNNNNNN

NNNNNNNNNNNNNNNNNNNNNNNNNNNNCCCTTTGTGAACTTTTACTGTTGCCTCGGAAG

GCTGGGGGGCCCCTCCGGGTGTTGAGACAGCCCGCCGGCGGCCAAGTTAACTCTTGTTTT

TACACTGAAACTCTAAATGAATCAAAACTTTCAACAACGGATCTCTTGGTTCTGGCATCG

ATGAAGAACGCAGCGAAATGCGATAAGTAATGTGAATTGCAGAATTCAGTGAATCATCGA

ATCTTTGAACGCACATTGCGCCCTCTGGTATTCCGGAGGGCATGCCTGTTCGAGCGTCAT

TTCAACCCTCAAGCCTGGCTTGGTGATGGGGCACTGAGGAGCAGGCCCTGAAATTCAGTG

GCGAGCTCGCCAGGACCCCGAGCGCAGTAGACCCTCGCTAAGGCCCTGGCGGTGCCCTGC

TGAAAGAGAAGGAAGGTTAGTAAACACCATGATGCTCGTGTGGCTTCGTCAGCGCATTTT

CACCCCTCGCTTTGGATTTTCAGGGTGCGGGGCTTAGGGCTTATCTCACCACCACTACCG

AACATATGCTGATATCTACACAGCCGCCGAGCTTGGTAAGGG??????????????????

????????????????????????????????????????????????????????????

????????????????????????????????????????????????????????????

????????????????????????????????????????????????????????????

????????????????????????????????????????????????????????????

????????????????????????????????????????????????????????????

????????????????????????????????????????????????????????????

????????????????????????????????????????????????????????????

????????????????????????????????????????????????????????????

????????????

>Diaporthe_acerina_CBS_137_27

NNNNNNNNNNNNNNNNNNNNNNNCCCGCCCTTGCTGTTGCGCATGCTAACGGACCGTTTT

CGGCCTGCAGGATAAGGATGGCGACGGTTAGTGCGGCTGCTCCCACTCAGCACGCGTCAC

GCTCGATCCGCCCCGATGGCCTGCGCGAGACCAAGCGTCATCACTATCAGGAGTTGCTAA

GGTGTAGGACAAATCACCACAAAGGAGCTCGGCACGGTCATGCGATCCCTGGGTCAGAAC

CCGTCCGAGTCTGAGCTGCAAGATATGATTAACGAGGTCGACGCCGACAACAATGGCACC

ATTGACTTTGGTACGTTCAGATGCTCACTCGTCCGCGCCCTCCACCGGAGGTGTCAAGAA

GCCTCACCGCTACAAGCCCGGTACCGTCGCTCTGCGTGAGATCCGTCGCTACCAGAAGTC

CACCGAGCTGCTGATCCGCAAGCTCCCCTTCCAGCGTCTGGTATGCAGGTTCGTGAGATC

GCCCAGGACTTCAAGTCCGACCTGCGCTTCCAGTCCTCCGCCATCGGTGCCCTGCAGGAG

TCTGTCGAGTCTTACCTCGTCTCCCTCTTCGAGGACACCAACCTGTGCGCCATCCACGCC

AAGCGTGTCACCATCCAGTCGGTATGTAACAAGGTCTCCGTTGGTGAACCAGCGGAGGGA

TCATTGCTGCCCCAGGCGCACCCAGAAACCCTTTGTGAACTTTTACTGTTGCCTCGGCAG

GCCGGGGGGCCCCTTTCTCCAAGGAGCAGGCCCGCCGGCGGCCAAGCCAACTCTTGTTTT

TACACTGAAGCTCTAAATGAATCAAAACTTTCAACAACGGATCTCTTGGTTCTGGCATCG

ATGAAGAACGCAGCGAAATGCGATAAGTAATGTGAATTGCAGAATTCAGTGAATCATCGA

ATCTTTGAACGCACATTGCGCCCTCTGGTATTCCGGAGGGCATGCCTGTTCGAGCGTCAT

TTCAACCCTCAAGCCTGGCTTGGTGATGGGGCACTGGAGGGCAGGCCCTGAAATCTAGTG

GCGGGCTCGCCAGGACCCCGAGCGCAGTAGACCCTCGCTAAGGCCCTGGCGGTGCCCTGC

TGAAAGAGAAGGAAGGTTAGTAAACATCCTCGTGTTTGCGCGGCTTCGTCACCGCATTTT

CACCCCTCGCTCTGGATTTTCAGGGTGCGGGGCTTAGAGCTTATCTCACCATCACCCCCG

GAAACATGCTGACATTTACACAGCCGCCGAGCTTGGTAAGGGTCGCACCTGAGCCCCACC

ATCGCGACCCACCCCCTGAGACACCCAGATAAGACGCGTCGATTGCTAACGTGTTTTTCT

CGCCCACAGGTTCACCTTCAGACCGGCCAATGCGTAAGTTGCTGTCACCACCGGACCTTA

T---CGCCACATGTAGCACGTTTCCCAGGGTAACCAAATCGGTGCTGCTTTCTGGTGCGT

CCATAATCCACCACCGCGATACGACCTCGCAGCATACTGACCTCGTAGGCAAACCATCTC

TGGCGAGCACGGCCTCGACAGCAATGGCGTGTATGCACCTCCTACCCCTGCCCATCTCGT

CCTCCCTGACAATGGCACAGTTACAACGGCACTTCCGAGCTCCAGCTCGAGCGCATGAAC

GTCTACTTCAACGAGGTAAGTACATCGTCGACACCATCTACAGCCTTGCTAACGCGTTAT

CAGGCCTCCGGCAACAAGTATGTGCCTCGCGCCGTCCTCGTCGATCTCGAGCCCGGTACC

ATGGACGCCGTC

>Diaporthe_acuta_PSCG_047

NNNNNNNNNNNNNNNNNNNNNNNCCCGCCCTTGCTGTTGCGCATGCTAACGGACCGTTTT

CGGTTTGTAGGATAAGGATGGCGATGGTTAGTGCGGCCGCTCTCACACAGCACGCGTCAT

GCTCGATCCTCCGCGACGGCCTGCGCGCAACCAAGCGTTATCACTATTGCGAGTTGCTGA

GGTGCAGGACAAATCACCACCAAGGAGCTCGGCACCGTCATGCGATCCCTGGGCCAGAAC

CCTTCCGAGTCTGAGCTGCAAGATATGATTAACGAGGTCGACGCCGACAACAACGGCACC

ATTGACTTTGGTACGTCCAGATATACGCCCATCCGCGCCCTCCACCGGAGGTGTCAAGAA

GCCTCACCGCTACAAGCCTGGTACCGTCGCTCTGCGTGAGATCCGTCGCTACCAGAAGAG

CACTGAGCTGCTGATCCGCAAGCTCCCCTTCCAGCGTCTGGTATGCAGGTCCGTGAGATC

GCCCAGGACTTCAAGTCCGACCTCCGCTTCCAGTCCTCCGCCATCGGTGCCCTGCAGGAG

TCCGTCGAGTCTTACCTCGTCTCCCTCTTCGAGGACACCAACCTGTGCGCCATCCACGCC

AAGCGTGTCACCATCCAGTCGGTACGTNNNNNNNNNNNNNNNNNNNNNNNNNNNNNNNNN

NNNNNNNNNNNNNNNNNNNNNNNNNNNNCCCTATGTGAACT---TCTGTTGCCTCGGCTG

CTGGTTAGGCCCTTTGTGGTAAAGAGAAGGCACGCCGGCGGCCAAAACAACTC-TGTTTT

TATACTGAAACTCTAAATGAATCAAAACTTTCAACAACGGATCTCTTGGTTCTGGCATCG

ATGAAGAACGCAGCGAAATGCGATAAGTAATGTGAATTGCAGAATTCAGTGAATCATCGA

ATCTTTGAACGCACATTGCGCCCTCTGGTATTCCGGAGGGCATGCCTGTTCGAGCGTCAT

TTCAACCCTCAAGCATTGCTTGGTGTTGGGGCACTGCGAAGCAGGCCCTGAAATCTAGTG

GCGAGCGCGCCAGGACCCCGAGCGTAGTAGACCCTCGCTAAGGCCCTGGCGGTGCCCTGC

TGAAANNNNNNNNNNNNNNNNNNNNNNCATCACGTTGGTACGGCTTCGTCAGCGCATTTT

CACCCCTCGCTCTGGATTTTCAGGGTGCGGGGCTTAGAGCTTATCT-----CTACCGCTG

GAAACATGCTGATATCTACATAGCCGCTGAGCTTGGTAAGGGNNNNNNNNNNNNNNNNNN

NNNNNNNNNNNNNNNNNNNNNNNNNNNNNNNNNNNNNNNNNNNNNNNNNNNNNNNNNNNN

NNNNNNNNNNNNNNNNNNNNNNNNNNNNNNNNNNNNNNNNNNNNNNNNNNNNNNNNNNNN

NNNNNNNNNNNNNNNNNNNNNNNNNNNNNNNNNNNNNNNNNNNNNNNNNNNNNNNGTT--

-----GGGCTCCACCGCGACATGACCTCGAGCCATACTGACCTCGCAGGCAAACCATCTC

TGGCGAGCACGGCCTCGACAGCAATGGCGTGTATGCACCTCCTATTTCTGCCTTTCTCGT

CTGCCCTGACAATCACACAGTTACAACGGCACTTCCGAGCTCCAGCTCGAGCGCATGAAC

GTCTACTTCAACGAGGTAAGTACGTCG--TTGATCATCTGCAGCCTTGCTAACGCGTTAT

CAGGCCTCCGGCAACAAGTATGTCCCTCGCGCCGTCCTCGTCGATCTCGAGCCCGGTACC

ATGGACGCCGTC

>Diaporthe_acutispora_CGMCC_3_18285

CTTTGTAATCTCCATTCGACATGCCCGCCCTTGCTGTTGCGCATGCTAACGAACCGTTTT

CGGCTTGTAGGATAAGGATGGCGATGGTTAGTGCGGACGTCTCCCCCCACCACGCGTCAC

CCTCGATCCGCCGCGACGGTCTGCGCGCAACCAAGCGTTATCATTATCGCGAGTTGCTGA

GGTGTAGGACAAATCACCACCAAGGAGCTCGGCACGGTCATGCGATCACTGGGTCAGAAC

CCGTCCGAGTCGGAGCTGCAAGATATGATTAACGAGGTCGACGCCGACAACAACGGCACC

ATTGACTTTGGTATGTCCAGATGTGCGCCCGTCCGCGCCCTCCACCGGAGGTGTCAAGAA

GCCTCACCGCTACAAGCCTGGTACCGTCGCTCTGCGTGAGATCCGTCGCTACCAGAAGAG

CACCGAGCTGCTGATCCGCAAGCTCCCCTTCCAGCGTCTGGTATGCAGGTCCGTGAGATC

GCCCAGGACTTCAAGTCCGACCTCCGCTTCCAGTCTTCCGCCATCGGTGCCCTGCAGGAG

TCGGTCGAGTCTTACCTCGTCTCCCTCTTCGAGGACACCAACCTGTGCGCCATCCACGCC

AAGCGTGTTACCATCCAGTCGGTACGTNNNNNNNNNNNNNNNNNNNNNNNNNNGGAGGGA

TCATTGCTGCCCCAGGCGCACCCAGAAACCCTTTGTGAACTTTTACTGTTGCCTCGGCAT

GCCGGGGGGCCCCTCAGACGGAGGAGCAGGCACGCCGGCGGCCAAGTTAACTCTTGTTTT

TACACTGAAACTCTAAATGAATCAAAACTTTCAACAACGGATCTCTTGGTTCTGGCATCG

ATGAAGAACGCAGCGAAATGCGATAAGTAATGTGAATTGCAGAATTCAGTGAATCATCGA

ATCTTTGAACGCACATTGCGCCCTCTGGTATTCCGGAGGGCATGCCTGTTCGAGCGTCAT

TTCAACCCTCAAGCCTGGCTTGGTGTTGGGGCACTGGAGAGCAGGCCCTGAAATTCAGTG

GCGAGCTCGCTAGGACCCCGAGCGCAGTAGACCCTCGCTGAGGCCCTGGCGGTGCCCTGC

TGAAAGAGAAGGAAGGTTAGTAAACACCATCATGCTCGTGCGACTTCGTCAGCGCATTTT

CACCCCCC-CTCTGGATTTTCAGGGTGCGGGGCTTAGAGCTTATCTCACCATCACT---G

AATTTATGCTGAAATCTACACAGCCGCCGAACTTGGTAAGGGNNNNNNNNNNNNNNNNNN

NNNNNNNNNNNNNNNNNNNNNNNNNNNNNNNNNNNNNNNNNNNNNNNNNNNNNNNNNNNN

NNNNNNNNNNNNNNNNNNNNNNNNNNNNNNNNNNNNNNNNNNNNNNNNNNNNNNNNNNNN

NNNNNNNNNNNNNNNNNNNNNNNNNNNNNNNNNNNNNNNNGGTGCTGCTTTCTGGTGCGT

CC-AAGTCCACCACCGCGATACGACCTCGAACCATACTGACATCGTAGGCAAACCATCTC

TGGCGAGCACGGCCTCGACAGCAATGGCGTGTATGCACCTCCTATTCCTGCCTTTC----

-TGCCCTGACAATCACACAGTTACAACGGCACTTCCGAGCTCCAGCTCGAGCGCATGAAC

GTCTACTTCAACGAGGTAGGTGCGTCG--CTGACCATCTACAGCTTTGCTAACGCGTTAT

CAGGCCTCCGGCAACAAGTATGTGCCCCGCGCCGTCCTCGTCGATCTCGAGCCCGGTACC

ATGGACGCCGTC

>Diaporthe_aestuarium_BRIP_59930a

????????????????????????????????????????????????????????????

????????????????????????????????????????????????????????????

????????????????????????????????????????????????????????????

????????????????????????????????????????????????????????????

????????????????????????????????????????????????????????????

????????????????????????????????????????????????????????????

????????????????????????????????????????????????????????????

????????????????????????????????????????????????????????????

????????????????????????????????????????????????????????????

????????????????????????????????????????????????????????????

???????????????????????????AACAAGGTCTCCGTTGGTGAACCAGCGGAGGGA

TCATTGCTGCCCCAGGCGCACCCAGAAACCCTTTGTGAACTTTTACTGTTGCCTCGGCTT

GCCGGGGGGCCCCTCGAGACGAGGAGCAGGCACGCCGGCGGCCAAGTTAACTCTTGTTTT

TACACTGAAACTCTAAATGAATCAAAACTTTCAACAACGGATCTCTTGGTTCTGGCATCG

ATGAAGAACGCAGCGAAATGCGATAAGTAATGTGAATTGCAGAATTCAGTGAATCATCGA

ATCTTTGAACGCACATTGCGCCCTCTGGTATTCCGGAGGGCATGCCTGTTCGAGCGTCAT

TTCAACCCTCAAGCCTGGCTTGGTGATGGGGCGCTGAAGGGCACGCCCTCAAATCTAGTG

GCGAGCTCGCCAGGACCCCGAGCGCAGTAGACCCTCGCTAAGGCCCTGGCGGTGCCCTGC

?????NAGAAGGAAGGTTAGTAAACACCATCATGCTCGCGCGGCTTCGTCAGCGCATTTT

CACCCCTCGCTTTGGATTTTCAGGGTGCGGGGCTTAGGGCTTATCTCCCA-CCAACACGG

AATCCATGCTGATATTTCCACAGCCGCCGAGCTTGGCAAGGGTCGCACCTGAGCCCCACC

ATCGCGACCCACCACCTGGGACACCCAGATAAGACGCGTCGATTGCTAACGTGTTTTTCT

CGCCTTTAGGTTCACCTTCAGACCGGCCAATGCGTAAGTTGCTGTCAC-AGCGGACCTTA

TCATCGCCA-CTGTAGCACGTTTCCCAGGGTAACCAAATCGGTGCTGCTTTCTGGTGCGT

CCCAAGTCCACCGCCGCGATACGACCTCGAAGCATACTGACCTCGTAGGCAAACCATCTC

TGGCGAGCACGGCCTCGACAGCAATGGCGTGTATGCACCTCCTATTCCTGCCCATCTTGG

CTTCCCTGACAATTGCACAGTTACAACGGCAGTTCTGAGCTCCAGCTCGAGCGCATGAAC

GTCTACTTCAACGAGGTAAGTACATCATTCCGACCATCTCCAACCTTGCTAACGCGTTAT

CAGGCCTCCGGCAACAAGTATGTTCCCCGCGCCGTCCTCGTCGATCTCGAGCCCGGTACC

ATGGACGCCGTC

>Diaporthe_africana

CTTTGTAATCTCCAGCCGACACGCCCGCCCTTGCTGTTGCGCATGCTAACGGACCGTTTT

CGGCTTGTAGGATAAGGATGGCGATGGTTAGTGCGGCCGCTCTCACACAGCACGCGTCAC

GCTCGATCCGCCGCGACGGCCTGCGCGCAACCAAGCGTTATCACTATTGCGAGTTGCTGA

GGTGTAGGACAAATCACCACCAAGGAGCTCGGCACGGTCATGCGATCCCTGGGACAGAAC

CCGTCCGAGTCTGAGCTGCAAGATATGATTAACGAGGTCGACGCCGACAACAACGGCACC

ATTGACTTTGGTACGTCCAAAAGCACGCCCGTCCGCGCCCTCCACCGGAGGTGTCAAGAA

GCCTCACCGCTACAAGCCTGGTACCGTCGCTCTGCGTGAGATCCGTCGCTACCAGAAGAG

CACTGAGCTGCTGATCCGCAAGCTCCCCTTCCAGCGTCTGGTATGCAGGTTCGTGAGATC

GCCCAGGACTTCAAGTCCGACCTCCGCTTCCAGTCTTCCGCCATCGGTGCCCTGCAGGAG

TCTGTCGAGTCTTACCTCGTCTCCCTCTTCGAGGACACCAACCTGTGCGCCATCCACGCC

AAGCGTGTCACCATCCAGTCGGTATGTAACAAGGTCTCCGTTGGTGAACCAGCGGAGGGA

TCATTGCTGCCCCAGGCGCACCCAGAAACCCTTTGTGAACTTTTACTGTTGCCTCGGCAT

GCTGGGGGGCCCCTCGAGACGAGGAGCAGGCACGCCGGCGGCCAAGTTAACTCTTGTTTT

TACACTGAAACTCTAAATGAATCAAAACTTTCAACAACGGATCTCTTGGTTCTGGCATCG

ATGAAGAACGCAGCGAAATGCGATAAGTAATGTGAATTGCAGAATTCAGTGAATCATCGA

ATCTTTGAACGCACATTGCGCCCTCTGGTATTCCGGAGGGCATGCCTGTTCGAGCGTCAT

TTCAACCCTCAAGCATTGCTTGGTGTTGGGGCACTGAAAAGCAGGCCCTGAAATCTAGTG

GCGAGCTCGCCAGGACCCCGAGCGCAGTAGACCCTCGCTAAGGCCCTGGCGGTGCCCTGC

TGAAAGAGAAGGAAGGTTAGTAAACATCATCATGTTGGTACGGCTTCGTCAGCGCATTTT

CACCCCTCGCTCTGGA-TTTCAGGGTGCGGGGCTTAGAGCTTATCT-----ATGCCGTT?

??????????????????????????????????????????TCGCACCTGAGCCCCACC

ATCGCGACCCACCCCCTGGGACACCCAAATAAAACGCGTCGATTGCTAACGTGTTTTTCT

TGACTACAGGTTCACCTTCAGACCGGCCAATGCGTAAGTCGCTGTCACGCCGGA-CCTTA

TCATCGCCACCCGTAGCACGTTTCCCAGGGTAACCAAATCGGTGCTGCTTTCTGGTGCGT

CA-AAGTCCACCACCGCGATACGACCTCGAACCATACTGACATCGCAGGCAAACCATCTC

TGGCGAGCACGGCCTCGACAGCAATGGCGTGTATGCACCTCCTATTCCTACTCTTCTTGT

CTGCCCTGACAATCACACAGTTACAATGGCTCTTCCGAGCTCCAGCTCGAGCGCATGAAC

GTCTACTTCAACGAGGTAAGTACGTCG--TTGACCATCTGCAGCCTTGCTAACGCGTTAT

CAGGCCTCCGGCAACAAGTATGTTCCTCGCGCCGTCCTCGTCGATCTCGAGCCCGGTACC

ATGGACGCCGTC

>Diaporthe_afzeliae_SDBR_CMU467

CTTTGTAAGTACCATCTGACACGCCCGCCCTTGCTGTTGCGCATGCTAACGGACCGTTTT

CGGCTTGTAGGATAAGGATGGCGATGGTTAGTGCGGCCGCTCTCACACAGCACGCGTCAT

GCTCGATCCTCCGCGACGGCCTGCGCGCAACCAAGCGTGATCACTATTGCGAGTTGCTGA

GGTGCAGGACAAATCACCACCAAGGAGCTCGGCACCGTCATGCGATCCCTGGGCCAGAAC

CCTTCCGAGTCTGAGCTGCAAGACATGATTAACGAGGTCGACGCCGACAACAACGGCACC

ATTGACTTTGGTACGTCCAGATATACGCCCATCCGCGCCCTCCACCGGAGGTGTCAAGAA

GCCTCACCGCTACAAGCCTGGTACCGTCGCTCTGCGTGAGATCCGTCGCTACCAGAAGAG

CACTGAGCTGCTGATCCGCAAGCTCCCCTTCCAGCGTCTGGTATGCAGGTCCGTGAGATC

GCCCAGGACTTCAAGTCCGACCTTCGCTTCCAGTCCTCCGCCATCGGTGCCCTGCAGGAG

TCCGTCGAGTCTTACCTCGTCTCCCTCTTCGAGGACACCAACCTGTGCGCCATCCACGCC

AAGCGTGTCACCATCCAGTCGGTACGTAACAAGGTCTCCGTTGGTGAACCAGCGGAGGGA

TCATTGCTGCCCAAGGCGCACCCAGAAACCCTTTGTGAACTTTTACTGTTGCCTCGGCAT

GCTGGGGGGTCCCCTGAGACAGGGAGCAGGCACGCCGGCGGCCAAGTTAACTCTTGTTTT

TACACTGAAACTCTAAATGAATCAAAACTTTCAACAACGGATCTCTTGGTTCTGGCATCG

ATGAAGAACGCAGCGAAATGCGATAAGTAATGTGAATTGCAGAATTCAGTGAATCATCGA

ATCTTTGAACGCACATTGCGCCCTCTGGTATTCCGGAGGGCATGCCTGTTCGAGCGTCAT

TTCAACCCTCAAGCATTGCTTGGTGTTGGGGCACTGAAGGGCAGGCCCTGAAATCTAGTG

GCGAGCTCGCCAGGACCCCGAGCGCAGTAGACCCTCGCTAAGGCCCTGGCGGTGCCCTGC

TGAAAGAGAAGGAAGGTTAGTAAACATCATCATGTTGGTACAGCTTCGTCAGCGCATTTT

CACCCCTCGCTCTGGATTTTCAGGGTGCGGGGCTTAGAGCTTATCTAGTTATTACCACCG

GAAACATGCTGATATCTACATAGCCGCCGAGCTTGGTAAGGGNNNNNNNNNNNNNNNNNN

NNNNNNNNNNNNNNNNNNNNNNNNNNNNNNNNNNNNNNNNNNNNNNNNNNNNNNNNNNNN

NNNNNNNNNNNNNNNNNNNNNNNNNNNNNNNNNNNNNNNNNNNNNNNNNNNNNNNNNNNN

NNNNNNNNNNNNNNNNNNNNNNNNNNNTGGTAACCAAATCGGTGCTGCTTTCTGGTGCG-

------TCCACCACCGCGATACGACCTCGAACCATACTGACCTCGCAGGCAAACCATCTC

TGGCGAGCACGGCCTCGACAGCAATGGCGTGTATGCACCTCCTATTTCTGCCTTTCTCGT

CTGCCCTGACAATCACACAGTTACAACGGCACTTCCGAGCTCCAGCTCGAGCGCATGAAC

GTCTACTTCAACGAGGTAAGTACGTCA--TTGACCATCTGCAGCTTTGCTAACGCGTTAT

CAGGCCTCCGGCAACAAGTATGTCCCTCGCGCCGTCCTCGTCGATCTCGAGCCCGGTACC

ATGGACGCCGTC

>Diaporthe_aitkeniae_BRIP_58827a

????????????????????????????????????????????????????????????

????????????????????????????????????????????????????????????

????????????????????????????????????????????????????????????

????????????????????????????????????????????????????????????

????????????????????????????????????????????????????????????

????????????????????????????????????????????????????????????

????????????????????????????????????????????????????????????

????????????????????????????????????????????????????????????

????????????????????????????????????????????????????????????

????????????????????????????????????????????????????????????

???????????????????????????AACAAGGTCTCCGTTGGTGAACCAGCGGAGGGA

TCATTGCTGCCTC-GGCGCACCCAGAAACCCTTTGTGAACTTATACTGTTGCCTCGGCAG

GCCGGAAGGCCCCCTGAGACAGGGAGCA-GTCCGCCGGCGGCCAACCAAACTC-TGTTTC

TATAGTGAATCTCTAAATGAATCAAAACTTTCAACAACGGATCTCTTGGTTCTGGCATCG

ATGAAGAACGCAGCGAAATGCGATAAGTAATGTGAATTGCAGAATTCAGTGAATCATCGA

ATCTTTGAACGCACATTGCGCCCTCTGGTATTCCGGAGGGCATGCCTGTTCGAGCGTCAT

TTCAACCCTCAAGCCTGGCTTGGTGATGGGGCACTGAAGGGCAGGCCCTGAAATCTAGTG

GCGAGCTCGCCAGGACCCCGAGCGTAGTAGA-TCTCGCTAAGGCCCTGGCGGTGCCCTGC

TGAAANNNNNGGAAGGTCAGTAAACATCATTGTGCCTGCGCGGCTTCGTCAAGCCATTTT

CACCCCTCCCTCTGGGTTTTCAGGGTGCGGGGCTTACCGCTTATCTCACCGTCAACACCG

AACAGACGCTGATTTTCATACAGCTGCCGAGCTGGGCAAGGGTCGCACCTCAGCCCCACC

ATCGCGACCCACCCCCTGCGACACCAAGATAAGACGCGTCCATTGCTAACATG--TTTCT

CCTCCACAGGTTCACCTTCAGACCGGCCAATGCGTAAGTTGCTGTCATCGCCCGACCTTA

T---CGCCACCCGTAGCACGTTTCCCAGGGTAACCAAATCGGTGCTGCTTTCTGGTGCGT

ACCGAGCCTGCCACCGCGATACTAGCTCGCAATATGCTGACCTCGCAGGCAAACCATCTC

TGGCGAGCACGGTCTCGACAGCAATGGCGTGTATGTACCTCCTATTCCNNNNNNNNNNNN

NNNNNNNNNNNNNNNNNNNNNNNNNNNNNNNNNNNNNNNNNNNNNNNNNNNNNNNNNNNN

NNNNNNNNNNNNNNNNNNNNNNNNNNNNNNNNNNNNNNNNNNNNNNNNNNNNNNNNNNNN

NNNNNNNNNNNNNNNNNNNNNNNNNNNNNNNNNNNNNNNNNNNNNNNNNNNNNNNNNNNN

NNNNNNNNNNNN

>Diaporthe_alangii_CFCC_52556

NNNNNNNNNNNNNNNNNNNNNNNNCCGCCACTGCTGTT---CATGCTAACGGACCGTTTT

CGGCCCGCAGGATAAGGATGGCGATGGTTAGTGTGGTCACCCTCAGCTAGCACGCGTCAT

GCTCGATCCGCCGCGACGGCCTGCGCGCAAGCGACCGTCATCG-CATCACCAGTTGCTAA

GGTGCAGGACAAATCACCACCAAGGAGCTAGGCACGGTCATGCGGTCCCTGGGTCAAAAC

CCCTCCGAGTCTGAGCTGCAGGACATGATTAACGAGGTCGATGCCGACAACAATGGCACC

ATTGACTTTGGTAAGCCAAGATGCTCGCCCGTCCGCGCCCTCCACCGGAGGTGTCAAGAA

GCCTCACCGCTACAAGCCTGGTACCGTCGCTCTGCGTGAGATCCGTCGCTACCAGAAGAG

CACCGAGCTGCTGATCCGCAAGCTCCCCTTCCAGCGTCTGGTATGCAGGTCCGTGAGATC

GCCCAGGACTTCAAGTCCGACCTGCGCTTCCAGTCTTCTGCCATTGGTGCCCTGCAGGAG

TCCGTCGAGTCTTACCTCGTCTCTCTCTTCGAGGACACCAACCTGTGCGCCATCCACGCC

AAGCGTGTCACCATCCAGTCGGTACGTNNNNNNNNNNNNNNNNNNNNNNNNNNNNNNNNN

NNNNNNNNNNNNNNNNNNNNNNNNNNNNCCCTTTGTGAACTTATACTGTTGCCTCGGCTG

GCCGGGAGGCCCCCTGAGACAGGGAGCA-GCCCGCCGGCGGCCAAACAAACTCTTGTTTC

T-TAGTGAATCTCTAAATGAATCAAAACTTTCAACAACGGATCTCTTGGTTCTGGCATCG

ATGAAGAACGCAGCGAAATGCGATAAGTAATGTGAATTGCAGAATTCAGTGAATCATCGA

ATCTTTGAACGCACATTGCGCCCTCTGGTATTCCGGAGGGCATGCCTGTTCGAGCGTCAT

TTCAACCCTCAAGCCTGGCTTGGTGTTGGGGCACCGAAGGGCGGGCCCTGAAATCTAGTG

GCGAGCTCGCCAGGACCCCGAGCGTAGTAGA-TCTCGTTAAGGCCCTGGCGGTGCCCTGC

TGAAAGAGAAGGAAGGTGAGTAAACATTACTGCGTTTGCGCGGCTTCGTCAGGGCATTTT

CACCCCTCCCTCTGGATTTTCAGGGTGCGGGGCTTACGGCTTATCTCGCTGCCACCACCA

ATCAGAAGCTGACATCTCTATAGCCGCCGAGCTCGGTAAGGGTCGCACCTCAGCCCCACC

ATCGCGACCCACCCCCTGGGACACCCAGATAAGACGCGTCGATTGCTAACATGTTTTTCT

TGCCCACAGGTTCACCTCCAGACTGGCCAATGCGTAAGTTGCTGTCACCGCCCGACCTTA

T---CGCCACCTCTAGCACGTTTCCCAGGGTAACCAAATCGGTGCTGCTTTCTGGTGCGT

CCAGAGTCGACCACCGCGACAGTAGCTCGTAGCATACTGACATCGTAGGCAAACCATCTC

TGGCGAGCACGGCCTCGACAGCAATGGCGTGTATGCACCTCCTATTCCTGCCTATCTCGT

CCTACCTGACAATGGCACAGCTACAACGGCACTTCTGAGCTCCAGCTCGAGCGCATGAAC

GTCTACTTCAACGAGGTAAGCACGTTGTTTTGACCACCTGCAGCCTAGCTAACGCGTTAT

CAGGCCTCCGGCAACAAGTATGTGCCTCGCGCCGTCCTCGTCGATCTCGAGCCCGGTACC

ATGGATGCCGNN

>Diaporthe_albosinensis_CFCC_53066

CTTTGTAATCTCCAGCCGACACGCCCGCCCTTGCTGGTGCGCATGCTAACGGACCGTTTT

CGGCTTGTAGGATAAGGATGGCGATGGTTAGTGCGGCCGCTCTCACCTAGCACGCGTCTT

GCTCGAACCGCCGCGACAGCCTGCGCGCAACCACGCGTTATCACTATCACGAGTTGCTGA

GGTGTAGGACAAATCACCACCAAGGAGCTCGGCACGGTCATGCGGTCCCTTGGTCAGAAC

CCGTCCGAGTCTGAGCTGCAAGATATGATTAACGAGGTCGACGCCGACAACAATGGCACC

ATTGACTTTGGTACGTCCAGATGCTCGCCCTTCCGCGCCCTCCACCGGAGGTGTCAAGAA

GCCTCACCGCTACAAGCCTGGTACCGTCGCTCTGCGTGAGATCCGTCGCTACCAGAAGAG

CACTGAGCTGCTGATCCGCAAGCTCCCCTTCCAGCGTCTGGTATGCAGGTCCGTGAGATC

GCCCAGGACTTCAAGTCCGACCTCCGCTTCCAGTCTTCCGCCATCGGCGCCCTGCAGGAG

TCGGTCGAGTCTTACCTCGTCTCCCTCTTCGAGGACACCAACCTGTGCGCCATCCACGCC

AAGCGTGTCACCATCCAGTCGGTATGTNNNNNNNNNNNNNNNNNNNNNNNNNNNNNNNNN

NNNNNNNNNNNNNNNNNNNNNNNNNNNNCCCTTTGTGAACTTTTACTGTTGCCTCGGAAG

GCTGGGGGGCCCCTCCGGGTGTTGAGACAGCCCGCCGGCGGCCAAGTTAACTCTTGTTTT

TACACTGAAACTCTAAATGAATCAAAACTTTCAACAACGGATCTCTTGGTTCTGGCATCG

ATGAAGAACGCAGCGAAATGCGATAAGTAATGTGAATTGCAGAATTCAGTGAATCATCGA

ATCTTTGAACGCACATTGCGCCCTCTGGTATTCCGGAGGGCATGCCTGTTCGAGCGTCAT

TTCAACCCTCAAGCCTGGCTTGGTGATGGGGCACTGAGAAGCAGGCCCTGAAATTCAGTG

GCGAGCTCGCCAGGACCCCGAGCGCAGTAGACCCTCGCTAAGGCCCTGGCGGTGNNNNNN

NNNNNNNNNNNNAAGGTTAGTAAACACCATGATGCTCGTGTGGCTTCGTCAGCGCATTTT

CACCCCTCGCTTTGGATTTTCAGGGTGCGGGGCTTAGGGCTTATCTCACCACCACTACCG

AACATATGCTGATATCTACACAGCCGCCGAGCTTGGTAANNNTCGCACCTGAGCCCCACC

ATCGCGACCCACCCCCTGGGACACCCAGATAAGACGCGTCGATTGCTAACGTGTTTTTCT

CGCCTATAGGTTCACCTTCAGACCGGCCAATGCGTAAGTTGCTGTCAC-AGTGGACCTTA

TCATCGCCACCTGTAGCACGTTTCCCAGGGTAACCAAATCGGTGCTGCTTTCTGGTGCGT

CCCAAGTCCACCACCGCGATACGACCTCGAGGCATACTGACCTCGTAGGCAAACCATCTC

TGGCGAGCACGGCCTCGACAGCAATGGCGTGTATGCACCTCCTATTCCTGCCCATCTTGG

CTTTCCTGACAATTGCACAGTTACAACGGCACCTCTGAGCTCCAGCTCGAGCGCATGAAC

GTCTACTTCAACGAGGTAAGTACGGCATTTTGACCATCTGCAACCTTGCTAACGCGTTAT

CAGGCCTCCGGCAACAAGTATGTTCCTCGCGCCGTCCTCGTCGATCTCGAGCCCGGTACC

ATGGACGCCGTN

>Diaporthe_alleghaniensis_CBS_495_72

CTTTGTAATCTCCAGCCGACACGCCCGCCCTTGCTGGTGCGCATGCTAACGGACCGTTTT

CGGCTTGTAGGATAAGGATGGCGATGGTTAGTGCGGCCGCTCCCACCTAGCACGCGTCAT

GTTCGATCCGCCGCGACAGCCTGCGCGCAACCAAGCGTTATCACTATCACGAGTTGCTGA

GGTGTAGGGCAAATCACCACCAAGGAGCTCGGCACGGTCATGCGATCCCTGGGTCAGAAC

CCGTCCGAGTCTGAGTTGCAAGATATGATTAACGAGGTCGACGCCGACAACAATGGCACC

ATTGACTTTGGTACGTCCAGATGCTCGCGCTTCCGCGCCCTCCACCGGAGGTGTCAAGAA

GCCTCACCGCTACAAGCCTGGTACCGTCGCTCTGCGTGAGATCCGTCGCTACCAGAAGAG

CACCGAGCTGCTGATCCGCAAGCTCCCTTTCCAGCGTCTGGTATGCAGGTCCGTGAGATC

GCCCAGGACTTCAAGTCCGACCTCCGCTTCCAGTCTTCCGCCATCGGCGCCCTGCAGGAG

TCGGTCGAGTCCTACCTCGTCTCCCTCTTCGAGGACACCAACCTGTGCGCCATCCACGCC

AAGCGTGTCACTATCCAGTCGGTATGTNNNNNNNNNNNNNNNNNNNNNNNNNNNNAGGGA

TCATTGCTGCCCCAGGCGCACCCAGAAACCCTTTGTGAACTTTTACTGTTGCCTCGGCTA

GCTGGGGGGCCCCTCCGGGTGTTGAGACGGCCCGCCGGCGGCCAACCTAACTCTTGTTTT

TACACTGAATCTCTAAATGAATCAAAACTTTCAACAACGGATCTCTTGGTTCTGGCATCG

ATGAAGAACGCAGCGAAATGCGATAAGTAATGTGAATTGCAGAATTCAGTGAATCATCGA

ATCTTTGAACGCACATTGCGCCCTCTGGTATTCCGGAGGGCATGCCTGTTCGAGCGTCAT

TTCAACCCTCAAGCCTGGCTTGGTGATGGGGCACTGAAAAGCAGGCCCTGAAATTCAGTG

GCGAGCTCGCCAGGACCCCGAGCGCAGTAGACCCTCGCTAAGGCCCTGGCGGTGCCCTGC

TGAAANNNNNNNAAGGTTAGTAAATACCATCATGCTCGCGCGGCTTCGTCAGCGCATTTT

CACCCCTCGCTTTGGATTTTCAGGGTGCGGGGCTTAGGGCTTATCTCGCCACCACCACCG

AACATATGCTGATATCTACACAGCCGCCGAGCTTNNNNNNNNNNNNNNNNNNNNNNNNNN

NNNNNNNNNNNNNNNNNNNNNNNNNNNNNNNNNNNNNNNNNNNNNNNNNNNNNNNNNNNN

NNNNNNNNNNNNNNNNNNNNNNNNNNNNNNNNNNNNNNNNNNNNNNNNNNNNNNNNNNNN

NNNNNNNNNNNNNNNNNNNNNNNNNNNNNNNNNNNNNNNNGAGGCTGCTTTCTGGTGCGT

TCCAAGTCCACCGCCGCGATACGACCTCGAAGCATGCTGACCTCGTAGGCAAACCATCTC

TGGCGAGCACGGCCTCGACAGCAATGGCGTGTATGCACCTCCTATTCCTGCCCATCTTGG

CTTCCCTGACAATCGCATAGTTACAACGGCACTTCTGAGCTCCAGCTCGAGCGCATGAAC

GTCTACTTCAACGAGGTAAGTACATCATTCCGACCATCTCCAACCTCGCTAACGTGTTAT

CAGGCCTCCGGCAACAAGTATGTTCCTCGCGCCGTCCTCGTCGATCTCGAGCCCGGTACC

ATGGACGCCGTC

>Diaporthe_alnea_CBS_146_46

CTTTGTAATCTCCAGCCGACATGCCCGCCCTTGCTGGTGCGCATGCTAATGGACCGTTTT

CGGCTTGTAGGATAAGGATGGCGATGGTTAGTGCGGCCGCTCCAACCTAGCACGCGTCAT

GTTCGATCCGCTGCGACAGCCTGCGCGCAACCAAGCGTTATCACTATCACGAGTTGCTGA

GGTGTAGGACAAATCACCACCAAGGAGCTCGGCACGGTCATGCGATCCCTGGGTCAGAAC

CCGTCCGAGTCTGAGCTGCAAGATATGATTAACGAGGTCGATGCCGACAACAATGGCACC

ATTGACTTTGGTACGTCCAGATGCTCACGCTTCCGCGCCCTCCACCGGAGGTGTCAAGAA

GCCTCACCGCTACAAGCCTGGTACCGTCGCTCTGCGTGAGATCCGTCGCTACCAGAAGAG

CACCGAGCTGCTGATCCGCAAGCTCCCCTTCCAGCGTCTGGTACGCAGGTCCGTGAGATC

GCCCAGGACTTCAAGTCCGACCTCCGCTTCCAGTCTTCCGCCATCGGTGCCCTGCAGGAG

TCGGTCGAGTCTTACCTCGTCTCCCTCTTCGAGGACACCAATCTGTGCGCCATCCACGCC

AAGCGTGTCACCATCCAGTCGGTACGTAACAAGGTCTCCGTTGGTGAACCAGCGGAGGGA

TCATTGCTGCCCCAGGCGCACCCAGAAACCCTTTGTGAACTTTTACTGTTGCCTCGGCAT

GCCGGGGGGCCCCTCCCCCGGAGGAGCAGGCACGCCGGCGGCCAGCCCAACTCTTGTTTT

TACACTGAAACTCTAAATGAATCAAAACTTTCAACAACGGATCTCTTGGTTCTGGCATCG

ATGAAGAACGCAGCGAAATGCGATAAGTAATGTGAATTGCAGAATTCAGTGAATCATCGA

ATCTTTGAACGCACATTGCGCCCTCTGGTATTCCGGAGGGCATGCCTGTTCGAGCGTCAT

TTCAACCCTCAAGCCTGGCTTGGTGATGGGGCACTGGGGAGCAGGCCCTGAAATTCAGTG

GCGAGCTCGCCAGGACCCCGAGCGCAGTAGACCCTCGCTAAGGCCCTGGCGGTGTCCTGC

TGAAAGAGAAGGAAGGTTAGTAAATATCATCATGCTCGCGCGGCTTCGTCAGCGCATTTT

CACCCCTCGCTTTGGATTTTCAGGGTGCGGGGCTTAGGGCTTATCTTGTCACCACCACCG

AATATATGCTGATATCTACACAGCCGCCGAGCTTGGTAAGGGTCGCACCTGAGCCCCACC

ATCGCGACCCACCCCCGGGGACACCCAGATAAGACGCGTCGATTGCTAACGTGTATTTCT

CGCCTCTAGGTTCACCTTCAGACCGGCCAATGCGTAAGTTGCTGTCAC-AGCGGACCTTA

TCATCGCCACCTGTAGCACGTTTCCCAGGGTAACCAAATCGGTGCTGCTTTCTGGTGCGT

CCCAAGTCCACCGCCGCGATACGAACTTGAAGCATACTGACCTCGTAGGCAAACCATCTC

TGGCGAGCATGGCCTCGACAGCAATGGCGTGTATGCACCTCCTATTCCTGCCTATCTTGG

CTTTCCTGACAATTGCACAGTTACAACGGCACTTCTGAGCTCCAGCTTGAGCGCATGAAC

GTCTACTTCAACGAGGTAAGTACATCATTCCGACCATCTCCAACCTTGCTAACGCGTCAT

CAGGCCTCCGGCAACAAGTATGTTCCTCGCGCCGTCCTCGTCGATCTCGAGCCCGGTACC

ATGGACGCCGTC

>Diaporthe_ambigua_CBS_114015

CTTTGTAAGTTATCTTCGCCTTGCCCGCCGTTGCCGTTGCGCATGCTAACGGCCCGTTTT

CGGCCTGCAGGATAAGGATGGCGATGGTTAGTGCAGTCACTCCCAGCTAGCACGCGTCAC

ACTCGATCCGCCGCGACGGTCTGCGCGCAGACGACCGCCATCACCATCTCGAGTTGCTAA

GGTGTAGGACAAATCACCACCAAGGAGCTCGGCACGGTCATGCGTTCCCTGGGCCAGAAC

CCCTCCGAGTCCGAGCTGCAGGATATGATTAACGAGGTCGACGCCGACAACAATGGCACC

ATTGACTTTGGTAAGTCCAGATGCTCGCGCATCCGCGCCGTCCACCGGAGGTGTCAAGAA

GCCTCACCGCTACAAGCCTGGTACCGTCGCTCTGCGTGAGATCCGTCGCTACCAGAAGAG

CACCGAGCTGCTGATCCGCAAGCTCCCCTTCCAGCGTCTGGTACGAAGGTTCGTGAGATC

GCCCAGGACTTCAAGTCCGACCTGCGCTTCCAGTCTTCCGCCATCGGTGCCCTGCAGGAG

TCCGTCGAGTCTTACCTCGTCTCCCTCTTCGAGGACACCAACCTGTGCGCCATCCACGCC

AAGCGTGTCACCATCCAGTCGGTACGTAACAAGGTCTCCGTTGGTGAACCAGCGGAGGGA

TCATTGCTGCCTC-GGCGCACCCAGAAACCCTTTGTGAACTTA--TCGTTGCCTCGGAAG

GCCGGGAGGCCCCTTGGAACAAGGAGCA-GCCCGCCGGCGGCCAACCAAACTCTTGTTTC

T-TAGTGAATCTCTAAATGAATCAAAACTTTCAACAACGGATCTCTTGGTTCTGGCATCG

ATGAAGAACGCAGCGAAATGCGATAAGTAATGTGAATTGCAGAATTCAGTGAATCATCGA

ATCTTTGAACGCACATTGCGCCCTCTGGTATTCCGGAGGGCATGCCTGTTCGAGCGTCAT

TTCAACCCTCAAGCCTGGCTTGGTGATGGGGCACTGGGGAGCAGGCCCTGAAATCTAGTG

GCGAGCTCGCCAGGACCCCGAGCGTAGTAGA-TCTCGCTAAGGCCCTGGCGGTGCCCTGC

TGAAAGAGAAGGAAGGTCAGTAAACATCACTTCGTTTGCGCGGCTTCGCCAAGGCATTTT

CACCCCTCCCTCTGGATTTTCAGGGTGCGGGGCTTACGGCTTATCTTGCTGTTATCACCG

AACACATGCTGATTCCCACACAGCCGCCGAGCTGGGTAAGGGTCGCACCTGAGCCCCACC

ATCGCGACCCACCCCCTGGGACACCCAGATAAGACGCGTCGATTGCTAACATGTTTTTCT

CGCCCACAGGTTCACCTTCAGACCGGCCAATGCGTAAGTTGCTGTCACACCCGGACCTTA

T---CGCCACCTGTAGCACGTTTCCCAGGGTAACCAAATCGGTGCTGCTTTCTGGTGCGT

CCCAAGTCTACAACCGCGACACTAGCTCGGGGCATACTGACCTCATAGGCAAACCATCTC

TGGCGAGCACGGTCTCGACAGCAATGGCGTGTACGTACCTCGTATCCCTGCCCATCTCGT

CCCTCCTGACAACTGCACAGTTACAACGGCACTTCCGAGCTCCAGCTCGAGCGCATGAAC

GTCTACTTCAACGAGGTAAGTACGTCGTTTTGACAACCTACGGCCTTGCTAACGCATTAT

CAGGCCTCCGGCAACAAGTATGTGCCTCGCGCCGTCCTCGTCGATCTCGAGCCCGGTACC

ATGGACGCCGTC

>Diaporthe_ampelina_CBS_114016

NNNNNNNNNNNNNNNNNNNNNNNNNNGCCCTTGCTGTTGCGCATGCTAACGGGCCGTTTT

CGGCCTGCAGGATAAGGATGGCGATGGTTAGTGTGCCCGCACCCACGGAGCACGCGTCAT

GCTCGATCCGCCGCGACGGCCTGCGCGTGAACAGGCGTCAGCACTATCAGGGATTGCTAA

GGCGTAGGACAAATCACCACGAAGGAGCTCGGCACTGTCATGCGGTCTCTGGGCCAGAAC

CCGTCCGAGTCTGAACTGCAAGATATGATTAACGAGGTCGATGCCGACAACAATGGCACC

ATTGACTTTGGTACGTCCACACGCTCGCCCA?????????????????????????????

????????????????????????????????????????????????????????????

????????????????????????????????????????????????????????????

????????????????????????????????????????????????????????????

????????????????????????????????????????????????????????????

???????????????????????????NNNNNNNNNNNNNNNNNNNNNNNNNNNNNNNNN

NNNNNNNNNNNNNNNNNNNNNNNNNNAACCCTTTGTGAACTCTTACCGTTGCCTCGGCAG

GCCGGGGGGCCCCTCGAGACGAGGAGCAGGCCCGCCGGCGGCCAAGTTAACTCTTGTTTT

TACACTGAAACTCTAAATGAATCAAAACTTTCAACAACGGATCTCTTGGTTCTGGCATCG

ATGAAGAACGCAGCGAAATGCGATAAGTAATGTGAATTGCAGAATTCAGTGAATCATCGA

ATCTTTGAACGCACATTGCGCCCTCTGGTATTCCGGAGGGCATGCCTGTTCGAGCGTCAT

TTCAACCCTCAAGCCTGGCTTGGTGATGGGGCACTGGGGAGCAGGCCCTGAAATCCAGTG

GCGAGCTCGCCAGGACCCCGAGCGCAGTAGACCCTCGCTGAGGCCCTGGCGGTGCCCTGC

TGAAANNNNNNNAAGGTTAGTAAACATCATCGCGCCTGTGCAGCTCCGTCAGCGCATTTT

CACCCCTCGTTCTGGATTTTCAGGGTGCGGGGCTTAGAGCTTATCTCCCCACCACC---G

GAATGAAGCTAACATCTATACAGCCGCCGAGCTTNNNNNNNNNNNNNNNNNNNNNNNNNN

NNNNNNNNNNNNNNNNNNNNNNNNNNNNNNNNNNNNNNNNNNNNNNNNNNNNNNNNNNNN

NNNNNNNNNNNNNNNNNNNNNNNNNNNNNNNNNNNNNNNNNNNNNNNNNNNNNNNNNNNN

NNNNNNNNNNNNNNNNNNNNNNNNNNNNNNNNNNNNNNNNNNNNNNNNNNNNNNNNNNNN

NNNNNNNNNNNNNNNNNNNNNNNACCTCGCGGCATACTGACCTCGTAGGCAAACCATCTC

TGGCGAGCACGGCCTCGACAGCAATGGCGTGTATGCACCTCCTATCCCTGCACATCTCGC

CCTCCCTGACAATTTCACAGTTACAACGGCACTTCCGAGCTCCAGCTCGAGCGCATGAAC

GTCTACTTCAACGAGGTATGTGCGTTCTTTCGGTCATCGCCAACCTTGCTAATGCGTTAT

CAGGCCTCCGGCAACAAGTATGTTCCTCGCGCGGTCCTCGTCGATCTCGAGCCCGGTACC

ATGGACGCCGTC

>Diaporthe_amygdali_CBS_126679

CTTTGTAAGTTATTTTC------GCAGCCCTCACTGTCGCGCATGCTAACGGACCGTTCT

CGGCCTCCAGGATAAGGATGGCGATGGTTAGTGCAGCTGCTCCCAGCTTGTACGCGTCAC

GATCGACCCGCCGCGACGGCTTGCGCGCAACCAAGC-CCATAACTACTATGAGCTGCTAA

GGTGTAGGACAAATCACCACAAAGGAGCTCGGCACGGTCATGCGATCTCTGGGTCAGAAC

CCGTCCGAGTCTGAGCTGCAGGATATGATCAACGAGGTCGACGCCGACAACAATGGAACC

ATCGACTTTGGTACGTCCAGATGCTCGCTTGTCCGCGCCCTCCACCGGAGGTGTCAAGAA

GCCTCACCGCTACAAGCCTGGTACCGTCGCTCTGCGTGAGATTCGTCGCTACCAGAAGTC

CACTGAGCTTCTGATCCGCAAGCTGCCCTTCCAGCGTCTGGTACGCAGGTTCGTGAGATT

GCCCAGGACTTCAAGTCCGACCTCCGCTTCCAGTCCTCCGCCATCGGTGCCCTGCAGGAG

TCCGTCGAGTCCTACCTCGTCTCCCTCTTCGAGGACACCAACCTGTGCGCCATCCACGCC

AAGCGTGTCACCATCCAGTCGGTATGTAACAAGGTCTCCGTTGGTGAACCAGCGGAGGGA

TCATTGCTGCCTC-GGCGCACCCAGAAACCCTTTGTGAACTTTTACTGTTGCCTCGGCAG

GCCGGGGGGCCCCTCCTGACGAGGAGCAGGCTCGCCGGCGGCCAAGTTAACTCTTGTTTT

TAATTTGAAACTCTAAATGAATCAAAACTTTCAACAACGGATCTCTTGGTTCTGGCATCG

ATGAAGAACGCAGCGAAATGCGATAAGTAATGTGAATTGCAGAATTCAGTGAATCATCGA

ATCTTTGAACGCACATTGCGCCCTCTGGTATTCCGGAGGGCATGCCTGTTCGAGCGTCAT

TTCAACCCTCAAGCCTGGCTTGGTGATGGGGCACTGGAAGGCAGGCCCTGAAATTCAGTG

GCGAGCTCGCCAGGACTCCGAGCGCAGTAGACCCTCGCTAAGG-ACTGGCGGTGCCCTGC

TGAAAGAGAAGGAAGGTTAGTAAACATCCTGGCGTTCCCACAGCGTCATCAGCGCATTTT

CACCCCTCGCTCTGGATTTTCAGGGTGCGGGGCTTAGAGCTTATCTCACC---------T

TGGACATGCTGACATCTTCACAGCCGCTGAGCTCGGTAAGGGTCGCACCTGAGCCCCACC

ATCGCGACCCACCCCCTGAAACACTTCCATAAGACGCGTCGATTGCTAACATGTTTTTCT

CGCCCACAGGTTCATCTCCAGACCGGCCAATGCGTAAGTTGCTGTCACCACCGCACCTTA

TCGCCG---CCTGTAGCACGTTTCCCAGGGTAACCAAATCGGTGCTGCTTTCTGGTGCGT

C----GTCCATCACCGCGACACGACCTCGCAACATACTGACCTCGTAGGCAAACCATCTC

TGGCGAGCACGGCCTCGACACCAATGGCGTGTATGCACCTCCTATTCCTGCCCATCTCGG

CTCGGCTGACAATTGCACAGCTACAACGGCACTTCCGAGCTCCAGCTCGAGCGCATGAAC

GTCTACTTCAACGAGGTAAGTATGTCATGTTAACGAGCCCCCGCTTTGCTGACCGCTTAT

CAGGCCTCCGGCAACAAGTATGTTCCCCGCGCCGTCCTCGTCGATCTCGAGCCCGGTACC

ATGGACGCCGTC

>Diaporthe_anacardii_CBS_720_97

CTTTGTAATCCCCAGCCGATATGCCCGCC--TGCTGTTGCGCATGCTAACGGACCGTTTT

CGGCCTGCAGGATAAGGATGGCGATGGTTAGTGCGGCTGCACCCACCCAGTACGCGTCAC

GCTCGATCCGCCGCGACGGCCTGCGCACAACCTACCGTTGCCACTATCACGAGTTGCTGA

GGTGTAGGACAAATCACCACCAAGGAGCTCGGCACGGTCATGCGGTCCTTGGGTCAGAAC

CCGTCCGAGTCCGAGCTGCAAGATATGATCAACGAGGTCGACGCCGACAACAACGGCACC

ATTGACTTTGGTACGTTCAGATGCTCGCCCCTCCGCGCCCTCCACCGGAGGTGTCAAGAA

GCCTCACCGCTACAAGCCTGGTACCGTCGCTCTGCGTGAGATCCGTCGCTACCAGAAGAG

CACCGAGCTGCTGATCCGCAAGCTCCCCTTCCAGCGTCTGGTATGCAGGTTCGTGAGATC

GCCCAGGACTTCAAGTCCGACCTGCGCTTCCAGTCCTCCGCCATCGGTGCCCTGCAGGAG

TCCGTCGAGTCTTACCTCGTCTCCCTGTTCGAGGACACCAACCTGTGCGCCATCCACGCC

AAGCGTGTCACCATCCAGTCGGTACGTAACAAGGTCTCCGTTGGTGAACCAGCGGAGGGA

TCATTGCTGCCCCTGGCGCACCCAGAAACCCTTTGTGAACTTTTACTGTTGCCTCGGCAG

GCCGTGGGGTCCCTT--AACAAGGAGCA-GCCGGCCGGTGGCCAAATTAACTC-TGTTTT

TACACTGAAACTCTAAATGAATCAAAACTTTCAACAACGGATCTCTTGGTTCTGGCATCG

ATGAAGAACGCAGCGAAATGCGATAAGTAATGTGAATTGCAGAATTCAGTGAATCATCGA

ATCTTTGAACGCACATTGCGCCCTCTGGTATTCCGGAGGGCATGCCTGTTCGAGCGTCAT

TTCAACCCTCAAGCCTGGCTTGGTGTTGGGGCACTGAAGGGCAGGCCCTGAAATATAGTG

GCGAGCTCGCCAGGACTCCGAGCGTAGTAGACCCTCGCTAAGG-CCTGGCGGTGCCCTGC

TGAAAGAGAAGGAAGGTCAGTAAACACCATCATGTTCGTGCGGCTTCGTTGGCGCATTTT

CACCCCTCCCTCTGGATTTTCAGGGTGCGGGGCTTAGAGCTTATCTTACC---------A

AAAATATGCTGACATCTACACAGCCGCCGAGCTTGGTAAGGGTCGCACCTGAGCCCCACC

ATCGCGACCCTCCCCCTGGGACACCCAGTTAAGACGCGTCGATTGCTAACATGTTTTTCT

CGCCCACAGGTTCACCTTCAGACCGGCCAATGCGTAAGTCA------CACCGGA-CCTTA

TCATCGCCACCTGTAGCACGTTTCCCAGGGTAACCAAATCGGTGCTGCTTTCTGGTGCGT

CC-AAGTCCACCGCCGCGAGTCGACCCCGAAGCATACTGACCTCGCAGGCAAACCATCTC

TGGCGAGCACGGTCTCGACAGCAATGGCGTGTATGCACCTCCTTATTCTGTCTTTCTCGT

-TGCCCTGACAATCACACAGTTACAACGGCACTTCCGAGCTCCAGCTCGAGCGCATGAAC

GTCTACTTCAACGAGGTAAGTACGTTG--TTGACCATCTAAAGCTCTGCTAATGCGTTAT

CAGGCCTCCGGCAACAAGTATGTGCCCCGCGCCGTCCTCGTCGATCTCGAGCCCGGTACC

ATGGACGCCGTC

>Diaporthe_angelicae_CBS_111592

CTTTGTAAGTTATTCTCGCCTTGCCCGCCATTGCTGTTGCGCATGCTAACGGACCGTTTT

CGGCCTGCAGGATAAGGATGGCGATGGTTAGTGCGGTCACTCTCAGCTACCACGCGTCAT

ACTCGATCCGCCGCGACGGTCTGCGCGTGAGCGAGCGGCCTCATGATCACGAGATGCTAA

GGTGTAGGACAAATCACCACCAAGGAGCTCGGCACAGTCATGCGGTCCCTTGGTCAAAAC

CCTTCCGAGTCCGAGCTGCAGGACATGATCAACGAGGTCGACGCCGACAACAATGGCACC

ATTGACTTTGGTAAGTCTAGATGTCCACCCATCCGCGCCCTCCACCGGAGGTGTCAAGAA

GCCTCACCGCTACAAGCCTGGTACCGTCGCTCTGCGTGAGATCCGTCGCTACCAGAAGAG

CACCGAGCTGCTCATCCGCAAGCTCCCCTTCCAGCGTCTGGTAAGCAGGTTCGTGAGATC

GCCCAGGACTTCAAGTCCGACCTGCGCTTCCAGTCTTCCGCCATCGGCGCCCTCCAGGAG

TCTGTCGAGTCTTACCTCGTCTCCCTCTTCGAGGACACCAACCTGTGCGCCATCCACGCC

AAGCGTGTCACCATCCAGTCGGTACGTAACAAGGTCTCCGTTGGTGAACCAGCGGAGGGA

TCATTGCTGCCTC-GGCGCACCCAGAAACCCTTTGTGAACTTATACTGTTGCCTCGGCAG

GCCGGAAGGCCCCCTGAGACAGGGAGCA-GCCCGCCGGCGGCCAGCCAAACTC-TGTTTC

TATAGTGGATCTCTAAATGAATCAAAACTTTCAACAACGGATCTCTTGGTTCTGGCATCG

ATGAAGAACGCAGCGAAATGCGATAAGTAATGTGAATTGCAGAATTCAGTGAATCATCGA

ATCTTTGAACGCACATTGCGCCCTCTGGTATTCCGGAGGGCATGCCTGTTCGAGCGTCAT

TTCAACCCTCAAGCCTGGCTTGGTGATGGGGCACTGAAGGGCAGGCCCTGAAATCTAGTG

GCGAGCTCGCCAGGACCCCGAGCGCAGTAGA-TCTCGCTGAGGCCCTGGCGGTGCCCTGC

TGAAAGAGAAGGAAGGTCAGTAAACATCATTGTGCCTGCGCGGCTTGGTCAAGCCATTTT

CACCCCT-CCTCTGGGTTTTCAGGGTGCGGGGGTTACCGCTTATCTCACCGTCAACACCG

AACAGACGCTGATTTCCACACAGCCGCCGAGCTGGGCAAGGGTCGCACCTCAGCCCCACC

ATCGCGACCCACCCCCTGCGACACCCAGATAAGACGCGTCGATTGCTAACATGTTTCTCT

CTCCTACAGGTTCACCTTCAGACCGGCCAATGCGTAAGTCGCTGTCACGCCGGGACCTTA

T---CGCCACCCGTAGCACGTTTCCCAGGGTAACCAAATCGGTGCTGCTTTCTGGTGCGT

CAAACCGCCACGGCCGCGAGATTAGCTCGCAACATACTGACCTCGTAGGCAAACCATCTC

TGGCGAGCACGGTCTCGACAGCAATGGCGTGTATGGACCTCCTATTCCTGCCCATCTCGT

CCTCCCTGATGTTTGCACAGTTACAACGGCACTTCTGAGCTCCAGCTCGAGCGCATGAAC

GTCTACTTCAACGAGGTGAGTACGTCTTTTTGAGCCTTCTCGCCTTTACTGACGCGCTTT

CAGGCCTCCGGCAACAAGTATGTGCCCCGCGCCGTCCTCGTCGATCTCGAGCCCGGTACC

ATGGACGCCGTC

>Diaporthe_anhuiensis_CNUCC_201902

NNNNNNNCTATCTCAACGACACGCCCGCCCTTGCTGTTGCGCATGCTAACGGACCGTTTT

CGGCTTGTAGGATAAGGATGGCGATGGTTAGTGCGACCGCTCTCACACAGCACGCGTCAT

GCTCGATCCTCCGCGACGGCCTGCGCGCAACCAAACGTTATCACTATTGCGAGTTGCTGA

GGTGCAGGACAAATCACCACAAAGGAGCTCGGCACGGTCATGCGATCCCTGGGCCAGAAC

CCTTCCGAGTCTGAGCTGCAAGATATGATTAACGAGGTCGACGCCGACAACAACGGCACC

ATTGACTTTGGTACGTCCAGATATACGCCCATCCGCGCCCTCCACCGGAGGTGTCAAGAA

GCCTCACCGCTACAAGCCTGGTACCGTCGCTCTGCGTGAGATCCGTCGCTACCAGAAGAG

CACTGAGCTGCTGATCCGCAAGCTCCCCTTCCAGCGTCTGGTATGCAGGTCCGTGAGATC

GCCCAGGACTTCAAGTCCGACCTCCGCTTCCAGTCTTCCGCCATCGGTGCCCTGCAGGAG

TCCGTCGAGTCTTACCTCGTCTCCCTCTTCGAGGACACCAACCTGTGCGCCATCCACGCC

AAGCGTGTCACCATCCAGTCGGTACGTNNNNNNNNNNNNNNNNNNNNNNNNNNNNNGTGG

CGTCGGCTTCCCAGGGCGCACCCAGAAACCCTTTGTGAACTTTT-TTGTTGCCTCGGCAT

GCTGGTAGGCCCCTC-CGGTGAGGAGAAGGCACGCCGGCGGCCAAGTTAACTCTTGTTTT

TACACTGAAACTCTAAATGAATCAAAACTTTCAACAACGGATCTCTTGGTTCTGGCATCG

ATGAAGAACGCAGCGAAATGCGATAAGTAATGTGAATTGCAGAATTCAGTGAATCATCGA

ATCTTTGAACGCACATTGCGCCCTCTGGTATTCCGGAGGGCATGCCTGTTCGAGCGTCAT

TTCAACCCTCAAGCATTGCTTGGTGTTGGGGCACTGCCAAGCAGGCCCTGAAATCTAGTG

GCGAGCTCGCCAGGACCCCGAGCGCAGTAGACCCTCGCTAAGGCCCTGGCGGTGCCCTGC

TGAAANNNNNNNNNNNTTGGGGTATCACATCATGTTGGTACGGCTTCGTCAGCGCATTTT

CACCCCTCGCTTTGGATTTTCAGGGTGCGGGGCTTAGAGCTTATCTCGCTACTACCACCG

GAAACATGCTGATACCTACACAGCCGCCGAGCTTGGTAAGGGNNNNNNNNNNNNNNNNNN

NNNNNNNNNNNNNNNNNNNNNNNNNNNNNNNNNNNNNNNNNNNNNNNNNNNNNNNNNNNN

NNNNNNNNNNNNNNNNNNNNNNNNNNNNNNNNNNNNNNNNNNNNNNNNNNNNNNNNNNNN

NNNNNNNNNNNNNNNNNNNNNNNNNNNNNNNNNNNNNNNNNNNNNNNNNNNNNNNNNNNN

NNNNNNNNNNTAACCGCGATATGACCTCGAGCCATACTGACCTCGCAGGCAAACCATCTC

TGGCGAGCACGGCCTCGACAGCAATGGCGTGTATGCACCTCCTATTCCTACCTTTCTCGT

CTGCCCTGACAATCATACAGTTACAACGGCAGCTCCGAGCTCCAGCTCGAGCGCATGAAC

GTCTACTTCAACGAGGTAAGTACGTCG--TTAACCATCTGCGGCCTTGCTAACGCGTTAT

CAGGCCTCCGGCAACAAGTATGTCCCTCGCGCCGTCCTCGTCGATCTCGAGCCCGGTACC

ATGGACGCCGTC

>Diaporthe_annellsiae_BRIP_59731a

????????????????????????????????????????????????????????????

????????????????????????????????????????????????????????????

????????????????????????????????????????????????????????????

????????????????????????????????????????????????????????????

????????????????????????????????????????????????????????????

????????????????????????????????????????????????????????????

????????????????????????????????????????????????????????????

????????????????????????????????????????????????????????????

????????????????????????????????????????????????????????????

????????????????????????????????????????????????????????????

???????????????????????????AACAAGGTCTCCGTTGGTGAACCAGCGGAGGGA

TCATTGCTGCCCCAGGCGCACCCAGAAACCCTTTGTGAACTTTTACTGTTGCCTCGGAAT

GCTGGGGGGTCCCCTGAGACAGGGAGCAGGCACGCCGGCGGCCAAGTTAACTCTTGTTTT

TACACTGAAACTCTAAATGAATCAAAACTTTCAACAACGGATCTCTTGGTTCTGGCATCG

ATGAAGAACGCAGCGAAATGCGATAAGTAATGTGAATTGCAGAATTCAGTGAATCATCGA

ATCTTTGAACGCACATTGCGCCCTCTGGTATTCCGGAGGGCATGCCTGTTCGAGCGTCAT

TTCAACCCTCAAGCATTGCTTGGTGTTGGGGCACTGAAGGGCAGGCCCTGAAATCTAGTG

GCGAGCTCGCCAGGACCCCGAGCGTAGTAGACCCTCGCTAAGGCCCTGGCGGTGCCCTGC

NNNNNGAGAAGGAAGGTTAGTAAACATCATCATGTTGGTACGGCTTCGTCAGCGCATTTT

CACCCCTCGCTCTGGATTTTCAGGGTGCGGGGCTTAGAGCTTATCTACCA-CCACTTCAG

GAAACATGCTGATATCTACACAGCCGCCGAGCTTGGTAANNNTCGCACCTGAGCCCCACC

ATCGCGACCCACCCCCTGGGACACCCAGATAAAACGCGTCGATTGCTAACGTGTTTTTCT

CGACTACAGGTTCACCTTCAGACCGGCCAATGCGTAAGTTGCTGTCACGCCGGA-CCTTA

TCATCGCCACCCGTAGCACGTTTCCCAGGGTAACCAAATCGGTGCTGCTTTCTGGTGC--

-----GTCCACCACCGCGATACGACCTCGAGCCATACTGACCTCGCAGGCAAACCATCTC

TGGCGAGCACGGCCTCGACAGCAATGGCGTGTATGCACCTCCTATTCTTACCTCTCTCGT

CTGCCCTGACAATCATACAGTTACAACGGCACTTCCGAGCTCCAGCTCGAGCGCATGAAC

GTCTACTTCAACGAGGTAAGTACGTCG--TTGACCATCTGCAGCCTTGCTAACGCGTTAT

CAGGCCTCCGGCAACAAGTATGTCCCTCGCGCCGTCCTCGTCGATCTCGAGCCCGGTACC

ATGGACGCCGTC

>Diaporthe_antonovae_BRIP_58824b

????????????????????????????????????????????????????????????

????????????????????????????????????????????????????????????

????????????????????????????????????????????????????????????

????????????????????????????????????????????????????????????

????????????????????????????????????????????????????????????

????????????????????????????????????????????????????????????

????????????????????????????????????????????????????????????

????????????????????????????????????????????????????????????

????????????????????????????????????????????????????????????

????????????????????????????????????????????????????????????

???????????????????????????AACAAGGTCTCCGTTGGTGAACCAGCGGAGGGA

TCATTGCTGCCCC-GGCGCACCCAGAAACCCTTTGTGAACTT-TACTGTTGCCTCGGCAG

GCCGGAAGGCCCCCTGAGACAGGGAGCA-GCCCGCCGGCGGCCAACCAAACTCTTGTTTC

TATAGTGAATCTCTAAATGAATCAAAACTTTCAACAACGGATCTCTTGGTTCTGGCATCG

ATGAAGAACGCAGCGAAATGCGATAAGTAATGTGAATTGCAGAATTCAGTGAATCATCGA

ATCTTTGAACGCACATTGCGCCCTCTGGTATTCCGGAGGGCATGCCTGTTCGAGCGTCAT

TTCAACCCTCAAGCCTGGCTTGGTGATGGGGCACTGGGGAGCAGGCCCTGAAATCTAGTG

GCGAGCTCGCCAGGACCCCGAGCGTAGTAGA-TCTCGCTAAGGCCCTGGCGGTGCCCTGC

TGAAANNNAAGGAAGGTTAGTAAACATCGTTGTGCCTGCGGGGCTTCGTCAAGGCATTTT

CACCCCTCCCTCCGGATTTTCAGGGTGCGGGGCTTACCGCTTATCTCTGTGAC-GCACCG

AAAGCATGCTGATTTCTGTACAGCCGCCGAGCTGGGCAAGGGTCGCACCTCAGCCCCACC

ATCGCGACCCACCCCCTGCAACTCCAAGATAATACGCGTCGATTGCTAACATGTTTTTCT

CGCCCACAGGTTCACCTTCAGACCGGCCAATGCGTAAGTTGTTGTCACCGCCAGACCTTA

T---CGCCACCCGTAGCACGTTTCCCAGGGTAACCAAATCGGTGCTGCTTTCTGGTGCGT

CCCGAGCCTACCACCGCGAGGCGAGCTCGAAACAGACTGACCTCGTAGGCAAACCATCTC

TGGCGAGCACGGTCTCGACAGCAATGGCGTGTATGGACCTCCTATTCCTGACTA------

CCTCTCTGATGATCGCACAGTTACAACGGCACTTCCGAGCTCCAGCTCGAGCGCATGAAC

GTCTACTTCAACGAGGTAAGTACGTCGCCTTGA-CCGTCTCGCCCTTGCTAACGCGTTTT

CAGGCCTCCGGCAACAAGTATGTGCCCCGCGCCGTCCTCGTCGATCTCGAGCCCGGTACC

ATGGACGCCGTC

>Diaporthe_apiculatum_LC_3418

????????????????????????????????????????????????????????????

????????????????????????????????????????????????????????????

????????????????????????????????????????????????????????????

????????????????????????????????????????????????????????????

????????????????????????????????????????????????????????????

???????????????????????????????TCCGCGCCCTCCACCGGAGGTGTCAAGAA

GCCTCACCGCTACAAGCCTGGTACCGTCGCTCTGCGTGAGATCCGTCGCTACCAGAAGAG

CACTGAGCTGCTGATCCGCAAGCTCCCCTTCCAGCGTCTGGTATGCAGGTCCGTGAGATC

GCCCAGGACTTCAAGTCCGACCTCCGCTTCCAGTCTTCCGCCATCGGTGCCCTGCAGGAG

TCCGTCGAGTCTTACCTCGTCTCCCTCTTTGAGGACACCAACCTGTGCGCCATCCACGCC

AAGCGTGTCACCATCCAGTCGGTATGTNNNNNNNNNNNNNNNNNNNNNNNNNNNNNGGGA

TCATTGCTGCCCCAGGCGCACCCAGAAACCCTTTGTGAACTTTTACCGTTGCCTCGGCCA

GCTGGGGGGCCCCTCCGGGTGTTGAGAGAGCACGCCGGCGGCCAACCTAACTCTTGTTTT

TACACTGAAACTCTAAATGAATCAAAACTTTCAACAACGGATCTCTTGGTTCTGGCATCG

ATGAAGAACGCAGCGAAATGCGATAAGTAATGTGAATTGCAGAATTCAGTGAATCATCGA

ATCTTTGAACGCACATTGCGCCCTCTGGTATTCCGGAGGGCATGCCTGTTCGAGCGTCAT

TTCAACCCTCAAGCCTGGCTTGGTGATGGGGCACTGAAGGGCAGGCCCTGAAATACAGTG

GCGAGCTCGCCAGGACCCCGAGCGCAGTAGACCCTCGCTAAGGCCCTGGCGGTGCCCTGC

TGAAAGAGAAGGAAGGTTAGTAAATACCGTCGTGTTCGTGCGGCTTCGTCAGCGCATTTT

CACCCCTCGTTCTGGATTTTCAGGGTGCGGGGCTTAGGGCTTATCTCACTACCAACACCG

AATGCATGCTGATATCTACATAGCCGCCGNNNNNNNNNNNNNNNNNNNNNNNNNNNNNNN

NNNNNNNNNNNNNNNNNNNNNNNNNNNNNNNNNNNNNNNNNNNNNNNNNNNNNNNNNNNN

NNNNNNNNNNNNNNNNNNNNNNNNNNNNNNNNNNNNNNNNNNNNNNNNNNNNNNNNNNNN

NNNNNNNNNNNNNNNNNNNNNNNNNNNNNNNNNNNNNNNNNNNNNNNNNNNNNNNNNNNN

NNNNNNNNNNNNNNNNNNNNNNNACCTCGAAGCATATTGACCTCGTAGGCAAACCATCTC

TGGCGAGCACGGCCTCGACAGCAATGGCGTGTATGCACCTCCTATTCCTGCCCATCTTGG

CTTCCCTGACAATTGCACAGTTACAACGGCACTTCTGAGCTCCAGCTCGAGCGCATGAAC

GTCTACTTCAACGAGGTATGTACATCATTCCGACCATCCTCGACCTTGCTAACGCGTTTT

CAGGCTTCCGGCAACAAGTATGTTCCTCGCGCCGTCCTCGTCGATCTTGAGCCCGGTACT

ATGGACGCCGTC

>Diaporthe_aquatica_IFRDCC_3051

????????????????????????????????????????????????????????????

????????????????????????????????????????????????????????????

????????????????????????????????????????????????????????????

????????????????????????????????????????????????????????????

????????????????????????????????????????????????????????????

????????????????????????????????????????????????????????????

????????????????????????????????????????????????????????????

????????????????????????????????????????????????????????????

????????????????????????????????????????????????????????????

????????????????????????????????????????????????????????????

???????????????????????????NNNNNNNNNNNNNNNNNNNNNNNNNNNNNNNNN

NNNNNNNNNNNNNNNNNNNNNNNNNNNNNNNNNNNNNNNNNNNNNNNNNNNNNNNNNNNN

NNNNNNNNNNCTCACCCGGTGAGGAGCG-GCGCGCCGGCGGCCCACCCAACTCTGTTTCT

T--TGTGAATCTCTAAATGAATCAAAACTTTCAACAACGGATCTCTTGGTTCTGGCATCG

ATGAAGAACGCAGCGAAATGCGATAAGTAATGTGAATTGCAGAATTCAGTGAATCATCGA

ATCTTTGAACGCACATTGCGCCCTCTGGTATTCCGGAGGGCATGCCTGTTCGAGCGTCAT

TTCAACCCTCAAGCCTGGCTTGGTGTTGGGGCACTGGAGAGCAGGCCCTGAAATTCAGTG

NNNNNNNNNNNNNNNNNNNNNNNNNNNNNNNNNNNNNNNNNNNNNNNNNNNNNNNNNNNN

NNNNN???????????????????????????????????????????????????????

????????????????????????????????????????????????????????????

????????????????????????????????????????????????????????????

????????????????????????????????????????????????????????????

????????????????????????????????????????????????????????????

????????????????????????????????????????????????????????????

????????????????????????????????????????????????????????????

????????????????????????????????????????????????????????????

????????????????????????????????????????????????????????????

????????????????????????????????????????????????????????????

????????????????????????????????????????????????????????????

????????????

>Diaporthe_araucanorum_CBS145285

CTTTGTAAGTTCTCTTC------CCCGTCGTTGCTGTTGCGCATGCTAACGCACCGTTTT

CGGCCTCCAGGATAAGGATGGCGATGGTTAGTGCGGCTGCCCCTACCCTATACGCGTCGC

GATCGGTTCGCCGCGACAGCCTGCGCGCGATCAAGCGCCATCACTACCAGGAGTTGCTAA

GGTGTAGGACAAATCACCACAAAGGAGCTCGGAACGGTTATGCGATCGCTGGGTCAAAAC

CCGTCCGAATCTGAGCTGCAAGATATGATCAACGAGGTCGATGCCGACAACAATGGCACC

ATCGACTTTGGTACGTTCAGGTGTCTGCTCA?????????????????????????????

????????????????????????????????????????????????????????????

????????????????????????????????????????????????????????????

????????????????????????????????????????????????????????????

????????????????????????????????????????????????????????????

???????????????????????????NNNNNNNNNNNNNNNNNNNNNNNTGCGGAGGGA

TCATTGTTGCTCAC-GCGCATCCAGAAACCCTTTGTGAACTTATACTGTTGCCTCGGCAG

GCTGGGGGGCCCCTCTCGGTGAGGAGCAGGCCCGCCGGCGGCCAAGTTAACTCTTGTTTT

TACACTGAAACTCTAAATGAATCAAAACTTTCAACAACGGATCTCTTGGTTCTGGCATCG

ATGAAGAACGCAGCGAAATGCGATAAGTAATGTGAATTGCAGAATTCAGTGAATCATCGA

ATCTTTGAACGCACATTGCGCCCTCTGGTATTCCGGAGGGCATGCCTGTTCGAGCGTCAT

TTCAACCCTCAAGCCTGGCTTGGTGATGGGGCACTGCAAGGCAGGCCCTTAAATTCAGTG

GCGAGCTCGCCAGGACCCCGAGCGCAGTAGACCCTCGCTAAGGTCTTGGTGCGGCCCTGC

TGAAANNGAAGGAAGGTTAGTAAATATCCACACGCTCGCACGGCTGCGTCATCGCGTTTT

CACCCCTCGCTCTGGATTTTCAGGGTGCGGGGCTTAGAGCTTATCTCACAACCATCACCT

GAAATATGCTAACACTTTAACAGCCGCCGAGCTTGGCAAGGGNNNNNNNNNNNNNNNNNN

NNNNNNNNNNNNNNNNNNNNNNNNNNNNNNNNNNNNNNNNNNNNNNNNNNNNNNNNNNNN

NNNNNNNNNNNNNNNNNNNNNNNNNNNNNNTGCGTAAGTTGCTGTCACCTCCGCACCTTA

T---CTCCCCCTGTAGCACGTTTTCCAGGGTAACCAAATCGGTGCTGCTTTCTGGTGCGT

T----GTCCATCATCGCGACACGACCCCGAAACATATTGACTTCATAGGCAAACCATCTC

TGGCGAGCACGGCCTCGACAGCAATGGCGTGTACGTACCTCCTATTTCTACCCGTTTCGT

CCGCCCTGACAGCTTCACAGTTACAACGGCTCTTCTGAGCTCCAGCTCGAGCGCATGAAC

GTCTACTTCAACGAGGTCAGTGTTTTATACCCACGATCTCCAGCTTTGCTGACACCTTAT

CAGGCCTCCGGCAACAAGTATGTTCCTCGCGCTGTCCTCGTCGATCTCGAGCCCGGTACC

ATGGACGCCGTC

>Diaporthe_arctii_CBS_136_25

NNNNNNNNNNNNNNNNCGCCTTGCCCGCCATTGCCTTTGCGCATGCTAACGGACCGTTTT

CGGCCTGCAGGATAAGGATGGCGATGGTTAGTGCGGTCACTCTCAGCTACCACGCGTCAT

ACTCGATCCGCCGCGACGGTCTGCGCGTGAGCGAGCGGCCTCATGATCACGAGATGCTAA

GGTGTAGGACAAATCACCACCAAGGAGCTCGGCACAGTCATGCGGTCCCTTGGTCAAAAC

CCTTCCGAGTCCGAGCTGCAGGACATGATCAACGAGGTCGACGCCGACAACAATGGCACC

ATTGACTTTGGTAAGTCTAAATGTCCACCCATCCGCGCCCTCCACCGGAGGTGTCAAGAA

GCCTCACCGCTACAAGCCTGGTACCGTCGCTCTGCGTGAGATCCGTCGCTACCAGAAGAG

CACTGAGCTGCTCATCCGCAAGCTGCCCTTCCAGCGTCTGGTAAGCAGGTTCGTGAGATC

GCCCAGGACTTCAAGTCCGACCTGCGCTTCCAGTCTTCCGCCATCGGCGCCCTCCAGGAG

TCCGTCGAGTCCTACCTCGTCTCCCTCTTCGAGGACACCAACCTGTGCGCCATCCACGCC

AAGCGTGTCACCATCCAGTCGGTACGTAACAAGGTCTCCGTTGGTGAACCAGCGGAGGGA

TCATTGCTGCCTC-GGCGCACCCAGAAACCCTTTGTGAACTCATACTGTTGCCTCGGCAG

GCCGGAAGGCCCCCTGAGACAGGGAGCA-GCCCGCCGGCGGCCAACCAAACTC-TGTTTC

TATAGTGGATCTCTAAATGAATCAAAACTTTCAACAACGGATCTCTTGGTTCTGGCATCG

ATGAAGAACGCAGCGAAATGCGATAAGTAATGTGAATTGCAGAATTCAGTGAATCATCGA

ATCTTTGAACGCACATTGCGCCCTCTGGTATTCCGGAGGGCATGCCTGTTCGAGCGTCAT

TTCAACCCTCAAGCCTGGCTTGGTGATGGGGCACTGAAGGGCAGGCCCTGAAATCTAGTG

GCGAGCTCGCCAGGACCCCGAGCGTAGTAGA-TCTCGCTAAGGCCCTGGCGGTGCCCTGC

TGAAAGAGAAGGAAGGTCAGTAAACATCATTGTGCCTGCGCGGCTTCGTCAAGCCATTTT

CACCCCTCCCTCTGGGTTTTCAGGGTGCGGGGCTTACCGCTTATCTCACCGTCAACACCG

AACAGACGCTGATTTCCATACAGCCGCCGAGCTGGGCAAGGGTCGCACCTCAGCCCCACC

ATCGCGACCCACCCCCTGCGACACCCAGATAAGACGCGTCGATTGCTAACATGTTGTTCT

CTCCTACAGGTTCACCTTCAGACCGGCCAATGCGTAAGTTGCTGTCACGCCGAGACCTTA

T---CGCCACCCGTAACACGTTTCCCAGGGTAACCAAATCGGTGCTGCTTTCTGGTGCGT

CAAGCCGCCACGGCCGCGAGATTAGCTCGCAACACACTGACCTCGTAGGCAAACCATCTC

TGGCGAGCACGGTCTCGACAGCAATGGCGTGTATGCACCTCCTATTCCTGCCCATCTCGT

CCTCCCTGATGTTTGCACAGTTACAACGGCACTTCTGAGCTCCAGCTCGAGCGCATGAAC

GTCTACTTCAACGAGGTGAGTACGTTTTTTTGAGCCTTCTCGCCTTTACTGACGCGCTTT

CAGGCCTCCGGCAACAAGTATGTGCCTCGCGCCGTCCTCGTCGATCTCGAGCCCGGTACC

ATGGACGCCGTC

>Diaporthe_arecae_CBS_161_64

CTTTGTAATCTCCAACCGACACGCCCGCCCTTGCTGCTGCGCATGCTAACGGACCGTTTT

CGGCTTGTAGGATAAGGATGGCGATGGTTAGTGCGGCCGCTCTCACACAGCACGCGTCAT

GCTCGATCTTCCGCGACGGCCTGCGCGCAACTAAGCGTTATCACTATTGCGAGTTGCTGA

GGTGCAGGACAAATCACCACAAAGGAGCTCGGCACGGTCATGCGATCCCTGGGCCAGAAC

CCTTCCGAGTCCGAGCTGCAAGATATGATTAACGAGGTCGACGCCGACAACAACGGCACC

ATTGACTTTGGTACGTCCAGATATACGCCCATCCGCGCCCTCCACCGGAGGTGTCAAGAA

GCCTCACCGCTACAAGCCTGGTACCGTCGCTCTGCGTGAGATCCGTCGCTACCAGAAGAG

CACTGAGCTGCTGATCCGCAAGCTGCCCTTCCAGCGTCTGGTATGCAGGTCCGTGAGATC

GCCCAGGACTTCAAGTCCGACCTCCGCTTCCAGTCTTCCGCCATCGGTGCCCTGCAGGAG

TCCGTCGAGTCTTACCTCGTCTCCCTCTTCGAGGACACCAACCTGTGCGCCATCCACGCC

AAGCGTGTCACCATCCAGTCGGTACGTAACAAGGTCTCCGTTGGTGAACCAGCGGAGGGA

TCATTGCTGCCCTAGGCGCACCCAGAAACCCTTTGTGAACTTTTACTGTTGCCTCGGCAT

GCTGGGGGGCCCCCTGAGACAGGGAGCAGGCACGCCGGCGGCCAAGTTAACTCTTGTTTT

TACACTGAAACTCTAAATGAATCAAAACTTTCAACAACGGATCTCTTGGTTCTGGCATCG

ATGAAGAACGCAGCGAAATGCGATAAGTAATGTGAATTGCAGAATTCAGTGAATCATCGA

ATCTTTGAACGCACATTGCGCCCTCTGGTATTCCGGAGGGCATGCCTGTTCGAGCGTCAT

TTCAACCCTCAAGCATTGCTTGGTGTTGGGGCACTGCCGAGCAGGCCCTGAAATCTAGTG

GCGAGCTCGCCAGGACCCCGAGCGCAGTAGACCCTCGCTAAGGCCCTGGCGGTGCCCTGC

TGAAAGAGAAGGAAGGTTAGTAAACATCATCATGTTGGTACAGCTTCGTCAGCGCATTTT

CACCCCTCGCTCTGGATTTTCAGGGTGCGGGGCTTAGAGCTTATCTAGTTATTACCACCG

GAAACATGCTGATATCTACATAGCCGCCGAGCTTGGTAAGGGTCGCACATGAGCCCCACC

ATCGCGACCCACCCCCTGGGGGACCCAAATAAAACGCGTCGATTGCTAACGTGTTTTTCT

CGACTACAGGTTCACCTTCAGACCGGTCAATGCGTAAGTTGCCGTCACTCCAGA-CCTTA

TCATCGCCACCCGTAGCACGTTTCCCAGGGTAACCAAATCGGTGCTGCTTTCTGGTGC--

-----GTCCACCACCGCGATACGATCTCGAACCATACTGACCTCGCAGGCAAACCATCTC

TGGCGAGCACGGCCTCGACAGCAATGGCGTGTATGCACCTCCTATTTCTGCCTTTCTCGT

CTGCCCTGACAATCATACAGTTACAACGGCACTTCCGAGCTCCAGCTCGAGCGCATGAAC

GTCTACTTCAACGAGGTAAGCACGTCG--TTGACCATGTGCAGCTTTGCTGACGCGTTAT

CAGGCCTCCGGCAACAAGTATGTCCCTCGCGCCGTCCTCGTCGATCTCGAGCCCGGTACC

ATGGACGCCGTC

>Diaporthe_arengae_CBS_114979

NNNNNNNNTCTCCAACCGACACGCCCGC---TACTGTTGCGCATGCTAACGGACCGTTTT

CGGCTTGTAGGATAAGGATGGCGATGGTTAGTGCGGCGGCTCTCACACAGCACGCGTCAT

GCTCGATCCTTCGCGACGGCCTGCGCGCAACCAAGCGTTATCACTATTGCGAGTTGCTGA

GGTGCAGGACAAATCACCACCAAGGAGCTCGGCACGGTCATGCGATCCCTGGGCCAGAAC

CCTTCCGAGTCCGAGCTGCAAGATATGATTAACGAGGTCGACGCCGACAACAACGGCACC

ATTGACTTTGGTACGTCTAGATATACGCCCATCCGCGCCCTCCACCGGAGGTGTCAAGAA

GCCTCACCGCTACAAGCCTGGTACCGTCGCTCTGCGTGAGATCCGTCGCTACCAGAAGAG

CACTGAGCTGCTGATCCGCAAGCTCCCCTTCCAGCGTCTGGTATGCAGGTCCGTGAGATC

GCCCAGGACTTCAAGTCCGACCTCCGCTTCCAGTCCTCCGCCATCGGTGCCCTGCAGGAG

TCCGTCGAGTCTTACCTCGTCTCCCTCTTCGAGGACACCAACCTGTGCGCCATCCACGCC

AAGCGTGTCACCATCCAGTCGGTACGTAACAAGGTCTCCGTTGGTGAACCAGCGGAGGGA

TCATTGCTGCCCCAGGCGCACCCAGAAACCCTTTGTGAACTTTT-TTGTTGCCTCGGCAT

GCTGGTAGGCCCCTC-CGGTGAGGAGAAGGCACGCCGGCGGCCAAGTTAACTCTTGTTTT

TACACTGAAACTCTAAATGAATCAAAACTTTCAACAACGGATCTCTTGGTTCTGGCATCG

ATGAAGAACGCAGCGAAATGCGATAAGTAATGTGAATTGCAGAATTCAGTGAATCATCGA

ATCTTTGAACGCACATTGCGCCCTCTGGTATTCCGGAGGGCATGCCTGTTCGAGCGTCAT

TTCAACCCTCAAGCATTGCTTGGTGTTGGGGCACTGCGAAGCAGGCCCTGAAATCTAGTG

GCGAGCTCGCCAGGACCCCGAGCGCAGTAGACCCTCGCTAAGGCCCTGGCGGTGCCCTGC

TGAAAGAGAAGGAAGGTTAGTAAACATCATCATGTTGGTACAGCTTCGTCAGCGCATTTT

CACCCCTCGCTCTGGATTTTCAGGGTGCGGGGCTTAGAGCTTATCTAGTTACTACCACCG

GAAACATGCTGATATCTACATAGCCGCCGAGCTTGGTAAGGGTCGCACCTGAGCCCCACC

ATCGCGACCCACCCCCTGGGACACCCAGATAAAACGCGTCGATTGCTAACGTGTTTTTCT

CGACTACAGGTTCACCTTCAGACCGGCCAATGCGTAAGTTGCTGTCACGACGGA-CCTTA

TCATCGCCACCCGTAGCACGTTTCCCAGGGTAACCAAATCGGTGCTGCTTTCTGGTGC--

-----GTCCACCACCGCGATACGACCTCGAAACATACTGACCTCGCAGGCAAACCATCTC

TGGCGAGCACGGCCTCGACAGCAATGGCGTGTATGCACCTCCTATTCCTACCTTTCTCGT

CTGCCCTGACAATCATACAGTTACAACGGCACTTCCGAGCTCCAGCTCGAGCGCATGAAC

GTCTACTTCAACGAGGTAAGTACGTCG--TTGGCCATCTGCACCCTTGCTAACGCGTTAT

CAGGCCTCCGGCAACAAGTATGTCCCTCGCGCCGTCCTCGTCGATCTCGAGCCCGGTACC

ATGGACGCCGTC

>Diaporthe_arezzoensis_MFLU_19_2880

????????????????????????????????????????????????????????????

????????????????????????????????????????????????????????????

????????????????????????????????????????????????????????????

????????????????????????????????????????????????????????????

????????????????????????????????????????????????????????????

????????????????????????????????????????????????????????????

????????????????????????????????????????????????????????????

????????????????????????????????????????????????????????????

????????????????????????????????????????????????????????????

????????????????????????????????????????????????????????????

???????????????????????????AACAAGGTCTCCGTTGGTGAACCAGCGGAGGGA

TCATTGCTGCCCC-GGCGCACCCAGAAACCCTTTGTGAACTCAT-CTGTTGCCTCGGCAG

GCCGGGGGGCCCCCCGAGACGGGGAGCA-GCCCGCCGGCGGCCAACCAAACTCTTGTTTC

TACAGTGTATCTCTAAATGAATCAAAACTTTCAACAACGGATCTCTTGGTTCTGGCATCG

ATGAAGAACGCAGCGAAATGCGATAAGTAATGTGAATTGCAGAATTCAGTGAATCATCGA

ATCTTTGAACGCACATTGCGCCCTCTGGTATTCCGGAGGGCATGCCTGTTCGAGCGTCAT

TTCAACCCTCAAGCCTGGCTTGGTGATGGGGCACTGAAGGGCAGGCCCTGAAATCTAGTG

GCGAGCTCGCCAGGACCCCGAGCGTAGTAGA-TCTCGCTAAGGCCCTGGCGGTGCCCTGC

TGAAA???????????????????????????????????????????????????????

????????????????????????????????????????????????????????????

??????????????????????????????????????????NNNNNNNNNNNNNNNNNN

NNNNNNNNNNNNNNNNNNNNNNNNNNNNNNNNNNNNNNNNNNNNNNNNNNNNNNNNNNNN

NNNNNNNNNNNNNNNNNNNNNNNNNNNNNNNNNNNNNNNNNNNNNNNNNNNNNNNNNNNN

NNNNNNNNNNNNNNNNNNNNNNNNNNNNNNNNNNNNNNTCGGTGCTGCTTTCTGGTGCGT

CCCGAGCCTGCCACCGCGACACTAGCTCGCAACATACTGACCTCGTAGGCAAACCATCTC

TGGCGAGCACGGTCTCGACAGCAATGGCGTGTATGCACCTCCTATTCCTGCCCATCTCGT

CCTCCCTGATGATTATACAGTTACAACGGCACTTCCGAGCTCCAGCTCGAGCGCATGAGC

GTCTACTTCAACGAGGTATGTACGTCGTCTTGA-CCGTCTCGGCCTTGCTAACGCGTCTT

CAGGCCTCCGGCAACAAGTATGTGCCTCGCGCCGTCCTCGTCGATCTCGAGCCCGGTACC

ATGGACGCCGTC

>Diaporthe_aseana_MFLUCC_12_0299a

NNNNNNNNNNNNNNNNNNNNNNNCCTGCCCCTGCCGTTGCGCATGCTAACGGACCGTTTT

CGGCTTGTAGGATAAGGATGGCGATGGTTAGTGCGGCCGCTCTCACACAGCACGCGTCAC

GCTCGATCCGCCGCGACGGCCTGCGCGCAACCAAGCGTTATCATTATTGCGAGTTGCTGA

GGTGTAGGACAAATCACCACCAAGGAGCTCGGCACGGTCATGCGATCCCTGGGACAGAAC

CCGTCCGAGTCTGAGCTGCAAGATATGATTAACGAGGTCGACGCTGACAACAACGGCACC

ATTGACTTTGGTACGTCCAGATATACGCCCG?????????????????????????????

????????????????????????????????????????????????????????????

????????????????????????????????????????????????????????????

????????????????????????????????????????????????????????????

????????????????????????????????????????????????????????????

???????????????????????????NNNNNNNNNNNNNNNNNNNNNNNNNCGGAGGGA

TCATTGCTGCCCCAGGCGCACCCAGAAACCCTTTGTGAACTTTTACTGTTGCCTCGGCAT

GCTGGGGGGTCCCTCGAGACGAGGAGCAGGCACGCCGGCGGCCAAGTTAACTCTTGTTTT

TACACTGAAACTCTAAATGAATCAAAACTTTCAACAACGGATCTCTTGGTTCTGGCATCG

ATGAAGAACGCAGCGAAATGCGATAAGTAATGTGAATTGCAGAATTCAGTGAATCATCGA

ATCTTTGAACGCACATTGCGCCCTCTGGTATTCCGGAGGGCATGCCTGTTCGAGCGTCAT

TTCAACCCTCAAGCATTGCTTGGTGTTGGGGCACTGACGGGCAGGCCCTGAAATCTAGTG

GCGAGCTCGCCAGGACCCCGAGCGCAGTAGACCCTCGCTAAGGCCCTGGCGGTGCCCTGC

TGAAAGAGAAGGAAGGTTAGTAAACATCATCATGTTGGTATGGCTTCGTCAGCGCATTTT

CACCCCTCGCTTTGGA-TTTCAGGGTGCGGGGCTTAGAGCTTATCTCGCTACTACCACCG

AAAACATGCTGATTTCTACACAGCCGCCGAGCTTGGTAAGGGNNNNNNNNNNNNNNNNNN

NNNNNNNNNNNNNNNNNNNNNNNNNNNNNNNNNNNNNNNNNNNNNNNNNNNNNNNNNNNN

NNNNNNNNNNNNNNNNNNNNNNNNNNNNNNNNNNNNNNNNNNNNNNNNNNNNNNNNNNNN

NNNNNNNNNNNNNNNNNNNNNNNNNNNNNNNNNNNNNNNNAACGTTCTTCTGTGGTCC--

-----TAGTCCCCACTCGATACGA-TTCGATCCATACTGACCTCGCAGGCAAACCATCTC

TGGCGAGCACGGCCTCGACAGCAATGGCGTGTATGCACCTCCTATTCCTACCTCTCTCGT

CTGCCTTGACAATCATACAGTTACAACGGCACTTCCGAGCTCCAGCTCGAGCGCATGAAC

GTCTACTTCAACGAGGTAAGTACGTCG--TTGACCATCTGCAGCTTTGCTAACGCGTTAT

CAGGCCTCCGGCAACAAGTATGTTCCTCGCGCCGTCCTTGTCGATCTCGAGCCCGGTACC

ATGGACGCCGTC

>Diaporthe_asheicola_CBS_136967

CTTTGTAAGTTCTCTTC------CCCACCGTGGCTGTTGCGCATGCTAACGGACCGTTGT

CGGCCTCCAGGATAAGGATGGCGATGGTGAGTGCGGCTGCCTCTACCCTAAACGCGTCAC

GATCGATCCGCCGCGACAGCTTGCGCGCGATCAAGCGCCATCACTAGCAGGAGTTGCTAA

AGTGTAGGACAAATCACCACGAAGGAGCTCGGAACGGTCATGCGATCGCTGGGTCAGAAC

CCGTCCGAGTCTGAGCTGCAAGATATGATCAACGAGGTCGATGCCGACAACAATGGCACC

ATCGATTTTGGTACGTTCAGATGTCTGCTGA?????????????????????????????

????????????????????????????????????????????????????????????

????????????????????????????????????????????????????????????

????????????????????????????????????????????????????????????

????????????????????????????????????????????????????????????

???????????????????????????NNNNNNNNNNNNNNNNNNNNNNNNNNNNAGGGA

TCATTGCTGCCCCAGGCGCACCCAGAAACCCTTTGTGAACTTTTACTGTTGCCTCGGCTA

GCTGGGGGGCCCCTCCGGGTGTTGAGACAGCCCGTCGGCGGCCAACCTAACTCTTGTTTT

TACACTGAAACTCTAAATGAATCAAAACTTTCAACAACGGATCTCTTGGTTCTGGCATCG

ATGAAGAACGCAGCGAAATGCGATAAGTAATGTGAATTGCAGAATTCAGTGAATCATCGA

ATCTTTGAACGCACATTGCGCCCTCTGGTATTCCGGAGGGCATGCCTGTTCGAGCGTCAT

TTCAACCCTCAAGCCTGGCTTGGTGATGGGGCACTGAGAAGCAGGCCCTGAAATTCAGTG

GCGAGCTCGCCAGGACCCCGAGCGCAGTAGACCCTCGCTAAGGCCCTGGCGGTGCCCTGC

TGAAAGAGAAGGAAGGTCAGTAAATATCCACACGCCTCTACGGCTGCGTCATCGCATTTT

CACCCCTCGTTCTGGATTTTCAGGGTGCGGGGCTTAGACCTTATCTCACAACCACCACCG

GAAACGTGCTGATACTTTGCCAGCCGCCGAGCTTGGCAAGGGNNNNNNNNNNNNNNNNNN

NNNNNNNNNNNNNNNNNNNNNNNNNNNNNNNNNNNNNNNNNNNNNNNNNNNNNNNNNNNN

NNNNNNNNNNNNNNNNNNNNNNNNNNNNNNNNNNNNNNNNNNNNNNNNNNNNNNNNNNNN

NNNNNNNNNNNNNNNNNNNNNNNNNNNTGGTAACCAAAACGGTGCTGCTTTCTGGTGCGT

C----CTCCATCATCGCGACACGACCTCGCAACATATTGACTTCGTAGGCAAACCATCTC

TGGCGAGCACGGCCTCGACAGCAATGGCGTGTATGTACCTCCTATTCCTACCCGTCTCGT

CCGCCCTGACATCTTCACAGTTACAACGGCTCTTCTGAGCTCCAGCTCGAGCGCATGAAC

GTCTACTTCAACGAGGTCAGTTTTTTATACCCATGATCTCCAGCTTTGCTGACACCTTAT

CAGGC-TCCGGCAACAAGTATGTGCCTCGCGCTGTCCTCGTCGATCTCGAGCCCGGTACC

ATGGACGCCGTC

>Diaporthe_aspalathi_CBS_117169

CTTTGTA-CCCCCGGCTGACATGCCCGCCGTACCCGTCGCGCATGCTAACGGACCGTTTT

CGGCTTGTAGGATAAGGATGGTGATGGTTAGTCCAGCCGGTCCCACCCAATACGCGTCAC

GCTCGA-CCGCCGCGACGGCCTGCGCGCTTACAGGGGACGTCGATATCGCGAGTTGCTGA

G--GTAGGACAAATCACCACCAAGGAGCTCGGCACAGTCATGCGGTCCCTGGGCCAGAAC

CCCTCCGAGTCCGAGCTGCAAGACATGATCAACGAGGTCGACGCCGACAACAATGGCACC

ATTGACTTTGGTACGTGCAGCTGCTCGCCCATCCGCACCCTCCACCGGAGGTGTCAAGAA

GCCTCACCGCTACAAGCCTGGTACCGTCGCTCTGCGTGAGATCCGTCGCTACCAGAAGAG

CACCGAGCTGCTGATCCGCAAGCTCCCCTTCCAGCGTCTGGTATGCAGGTCCGTGAGATC

GCCCAGGACTTCAAGTCCGACCTCCGCTTCCAGTCTTCCGCCATCGGTGCCCTGCAGGAG

TCTGTCGAGTCTTACCTCGTCTCCCTCTTCGAGGACACCAACCTGTGCGCCATCCACGCC

AAGCGTGTCACCATCCAGTCGGTACGTAACAAGGTCTCCGTTGGTGAACCAGCGGAGGGA

TCATTGCTGCCCCAGGCGCACCCAGAAACCCTTTGTGAACTCTTACTGTTGCCTCGGCAG

GCCGGGGGGCCCCTCGAGACGAGGAGCAGGCCCGCCGGCGGCCAAGCCAACTCTTGTTTT

TACACCGAAACTCTAAATGAATCAAAACTTTCAACAACGGATCTCTTGGTTCTGGCATCG

ATGAAGAACGCAGCGAAATGCGATAAGTAATGTGAATTGCAGAATTCAGTGAATCATCGA

ATCTTTGAACGCACATTGCGCCCTCTGGTATTCCGGAGGGCATGCCTGTTCGAGCGTCAT

TTCAACCCTCAAGCCTGGCTTGGTGTTGGGGCACTGAAGGGCAGGCCCTGAAATCTAGTG

GCGGGCTCGCCAGGACCCCGAGCGCAGTAGACCCTCGCTGAGGCCCTGGCGGTGCCCTGC

TGAAAGAGAAGGAAGGTCAGTAAACATCCCTTCGTTTGCGCCGCGTCGCCACCGCATTTT

CACCCCTCGCTCTGGATTTTCAGAGTGCGGGGCTTAGTGCTTATCT------CACCACTG

AGAAGATGCTGACATTTCTACAGCCGCTGAGCTCGGTAAGGGTCGCACCTGAGCCCCACC

ATCGCGACCGTCAGCCTGCGACACCTGGATGAGACGCGCCCATTGCTAACTTATTTTTCT

TGCCTCCAGGTTCACCTCCAGACCGGCCAATGCGTAAGTTGCTTTCACCACCAGACCTTA

TCATCGCCACCCATAGCACGTTTCGCAGGGTAACCAAATCGGTGCTGCTTTCTGGTGCGT

GCTGAGCCTGCCACCGCGATACGTCCTAGAAGCATGCTGACCTCCTAGGCAAACCATCTC

TGGCGAGCACGGTCTCGACAGCAATGGCGTGTATGCACCTCCTATTCCTGCTCTTCTCGC

CCTCCCTGACAATTGCACAGCTATAACGGCACCTCCGAGCTCCAGCTCGAGCGCATGAAC

GTCTACTTCAACGAGGTAAGTTTGTCCT---GACCCCGCCCTTCCTTGCTGACGCATTAT

CAGGCTTCCGGCAACAAATATGTCCCTCGCGCCGTCCTCGTCGATCTCGAGCCCGGTACC

ATGGACGCTGTC

>Diaporthe_atlantica_CECT_21217

NNNNNNNNNNNNNNNNNNNNNNNNNNNNNNNNNNNNNNNCGCATGCTAACGGACCGTTTT

CGGCCTGCAGGATAAGGATGGCGATGGTTAGTGTGGCCACTGCTAGCTAGCACGCGTCAC

ACTCGATCCGCCGCGACGGTCTGCGCGCGAGCGACCGTCGTCACCGTCA-GGATTGCTAA

GGTGTAGGACAAATCACCACCAAGGAGCTCGGCACGGTCATGCGTTCCCTGGGTCAAAAC

CCCTCCGAGTCCGAGCTGCAGGATATGATCAACGAGGTCGACGCCGACAACAATGGCACC

ATTGACTTNNNNNNNNNNNNNNNNNNNNNNNTCCGCGCCCTCCACCGGAGGTGTCAAGAA

GCCTCACCGCTACAAGCCCGGTACCGTCGCTCTGCGTGAGATCCGTCGCTACCAGAAGAG

CACCGAGCTGCTGATCCGCAAGCTCCCCTTCCAGCGTCTGGTATGCAGGTTCGTGAGATC

GCCCAGGACTTCAAGTCCGACCTGCGCTTTCAGTCTTCCGCCATCGGTGCCCTGCAGGAG

TCTGTCGAGTCTTACCTCGTCTCTCTCTTCGAGGACACCAACCTGTGCGCCATCCACGCC

AAGCGTGTCACCATCCNNNNNNNNNNNNNNNNNNNNNNNNNNNNNNNNNNNNNNNNNNNN

NNNNNNNNNNNNNNNNNNNNNNNNNNNNNNNNNNNNNNNNNNTTACTGTTGCCTCGGCAG

GCCGGGAGGCCCCTCGAAACGAGGAGCA-GCCCGCCGGCGGCCAACCAAACTCTTGTTTC

T-TAGTGAATCTCTAAATGAATCAAAACTTTCAACAACGGATCTCTTGGTTCTGGCATCG

ATGAAGAACGCAGCGAAATGCGATAAGTAATGTGAATTGCAGAATTCAGTGAATCATCGA

ATCTTTGAACGCACATTGCGCCCTCTGGTATTCCGGAGGGCATGCCTGTTCGAGCGTCAT

TTCAACCCTCAAGCATTGCTTGGTGTTGGGGCACCGAAGGGCGGGCCCTGAAATCTAGTG

GCGAGCTCGCCAGGACCCCGAGCGTAGTAAA-TTTCGTTAAGGCCCTGGCGGTGCCCTGC

NNNNNNNNNNNNNNNNNNNNNNNNNNNNNNNNCGTTTGCCCGGCTGTGTCCAGGCATTTT

CACCCCTCCCTCTGGATTTCCAGGGTGCGGGGCTTACGGCTTATCTTGCTGCCACCACCG

CTATCTCATCAACCCCCCCCCCCCCCCCAAAAAAAAACCCGGTCGCACCTCAGCCCCACC

ATCGCGACCCACCCCCTGGGACACCCAGATAAGACGCGTCGATTGCTAACATGTTTTTCT

CACACACAGGTTCACCTTCAGACCGGCCAATGCGTAAGTTGCTGTCACACCCGGACCTTA

T---CGCCACCTGTAGCACGTTTCCCAGGGTAACCAAATCGGTGCTGCTTTCTGGTGCGT

CCCAAGACTATCGCCGCGACATTAGCTTGCTGCATACTGACCTCGTAGGCAAACCATCTC

TGGCGAGCACGGTCTCGACAGCAATGGCGTGTATGTACCTCCTATTCCTGCCCATCTCGT

CCTCTCTGACAATTGCACAGTTACAACGGCACTTCTGAGCTCCAGCTCGAGCGCATGAAC

GTCTACTTCAACGAGGTAAGTACGCTGTTTCGACCACCTACGGCCTTGCTAACGCGTTAT

CAGGCCTCCGGCAACAAGTATGTGCCTCGCGCCGTCCTCGTCGATCTCGAGCCCGGTACC

ATGGACGCCGTC

>Diaporthe_australafricana_CBS_111886

CTTTGTAAGTTATCTTC------CCCACCGTGGCTGTTGCGCATGCTAACGGACCATTAT

CGGCCTCCAGGATAAGGACGGCGATGGTGAGTGCGGCTGCCTCTACCCTAAACGCGTCAC

GATCGATCCGCCGCGACAGCTTGCGCGCGATCAAGCGCCATCACTAGCAGGAGTTGCTAA

AGTGTAGGACAAATCACCACGAAGGAGCTCGGAACGGTCATGCGATCGCTGGGTCAGAAC

CCGTCCGAGTCTGAGCTGCAAGATATGATCAACGAGGTCGATGCCGACAACAATGGCACC

ATCGATTTTGGTACGTTCAGATGTCTGCTGATCCGCGCCCTCCACCGGAGGTGTCAAGAA

GCCTCACCGTTACAAGCCCGGTACCGTCGCTCTGCGTGAGATCCGTCGCTACCAGAAGTC

CACTGAGCTGCTGATCCGCAAGCTCCCCTTCCAGCGTCTTGTATGCAGGTTCGTGAGATC

GCCCAGGACTTCAAGTCCGATCTCCGTTTCCAGTCCTCCGCCATCGGTGCCCTGCAGGAG

TCTGTCGAGTCTTACCTCGTCTCCCTGTTCGAGGACACCAACCTGTGCGCCATCCACGCC

AAGCGTGTCACCATCCAGTCGGTACGTAACAAGGTCTCCGTTGGTGAACCAGCGGAGGGA

TCATTGTTGCTCCG-GCGCATCCAGAAACCCTTTGTGAACTTATACTGTTGCCTCGGCAG

GCTGGGGGGTCCCTCTCGGTGAGGAGCAGGCCCGCCGGCGGCCAAGTTAACTCTTGTTTT

TACACTGAAACTCTAAATGAATCAAAACTTTCAACAACGGATCTCTTGGTTCTGGCATCG

ATGAAGAACGCAGCGAAATGCGATAAGTAATGTGAATTGCAGAATTCAGTGAATCATCGA

ATCTTTGAACGCACATTGCGCCCTCTGGTATTCCGGAGGGCATGCCTGTTCGAGCGTCAT

TTCAACCCTCAAGCCTGGCTTGGTGATGGGGCACTGGAAGGCAGGCCCTGAAATTCAGTG

GCGAGCTCGCCAGGACCCCGAGCGCAGTAGACCCTCGCTAAGGTCTTGGTGCGGCCCTGC

TGAAAGAGAAGGAAGGTTAGTAAATATCCACACGCCTCTACGGCTGCGTCATCGCATTTT

CACCCCTCGTTCTGGATTTTCAGGGTGCGGGGCTTAGACCTTATCTCACAACCACCACCG

GAAACGTGCTGATACTTTGCCAGCCGCCGAGCTTGGTAAGGGTCGCACCTGAGCCCCACC

ATCGCGACCCACCCCCTGAGACACTCAGATAAGACGCGTCGAATGCTGACCTTTTTTTCT

CGTTCACAGGTTCACCTTCAGACCGGCCAATGCGTAAGTTGCTGTCACCACCGCACCTTA

T---CGCCCCCTGTAGCACGTTTTCCAGGGTAACCAAATCGGTGCCGCTTTCTGGTGCGT

C----CTCCATCATCGCGACACGACCTCGCAACATATTGACTTCGTAGGCAAACCATCTC

TGGCGAGCACGGCCTCGACAGCAATGGCGTGTATGTACCTCCTATTCCTACCCGTCTCGT

CCGCCCTGACATCTTCACAGTTACAACGGCTCTTCTGAGCTCCAGCTCGAGCGCATGAGC

GTCTACTTCAACGAGGTCAGTTTTTTATACCCATGATCTCCAGCTTTGCTGACACCTTAT

CAGGCTTCCGGCAACAAGTATGTGCCTCGCGCTGTCCTCGTCGATCTCGAGCCCGGTACC

ATGGACGCCGTC

>Diaporthe_australiana_BRIP_66145

????????????????????????????????????????????????????????????

????????????????????????????????????????????????????????????

????????????????????????????????????????????????????????????

????????????????????????????????????????????????????????????

????????????????????????????????????????????????????????????

????????????????????????????????????????????????????????????

????????????????????????????????????????????????????????????

????????????????????????????????????????????????????????????

????????????????????????????????????????????????????????????

????????????????????????????????????????????????????????????

???????????????????????????AACAAGGTCTCCGTTGGTGAACCAGCGGAGGGA

TCATTGCTGCCCCAGGCGCACCCAGAAACCCTTTGTGAACTTTTACTGTTGCCTCGGCAT

GCTGGGGGGTCCCTCCCGATGAGGAGCAGGCACGCCGGCGGCCAAGTTAACTCTTGTTTT

TACACTGAAACTCTAAATGAATCAAAACTTTCAACAACGGATCTCTTGGTTCTGGCATCG

ATGAAGAACGCAGCGAAATGCGATAAGTAATGTGAATTGCAGAATTCAGTGAATCATCGA

ATCTTTGAACGCACATTGCGCCCTCTGGTATTCCGGAGGGCATGCCTGTTCGAGCGTCAT

TTCAACCCTCAAGCACTGCTTGGTGTTGGGGCACTGAAGGGCAGGCCCTGAAATCTAGTG

GCGAGCTCGCCAGGACCCCGAGCGCAGTAGACCCTCGCTAAGGCCCTGGCGGTGCCCTGC

TGAAAGAGAAGGAAGGTTAGTAAACATCATCATGTTGGTACGGCTTCGTCAGCGCATTTT

CACCCCTCGCTCTGGT-TTTCAGGGTGCGGGGCTTAGAGCTTATCT-----ATGCTGTTC

GAAACATGCTGATATCTACACAGCCGCCGAGCTTGGCAAGGGTCGCACCTGAGCCCCACC

ATCGCGACCCACCCCCTGGGACAACCAGATAAAACGCGTCGATTGCTAACGTGTTTTTCC

CGCCTACAGGTTCACCTTCAGACCGGCCAATGCGTAAGTTGCTGTCACGCCGGA-CCTTA

TCATCGCTACCCGTAGCACGTTTCGCAGGGTAACCAAATCGGTGCTGCTTTCTGGTGC--

-----GTCCACCACCGCGATACGACCTCGAACCATACTGACCTCGCAGGCAAACCATCTC

TGGCGAGCACGGCCTCGACAGCAATGGCGTGTATGCACCTCCTATTCCTACCTCTCTCGT

CTGCCCTGACAATCATACAGTTACAACGGCAGTTCCGAGCTCCAGCTCGAGCGCATGAAT

GTCTACTTCAACGAGGTAAGTACGTCG--TTGACCATGTGCAGCCTTGCTAACGCGTTAT

CAGGCCTCCGGCAACAAGTATGTCCCTCGCGCCGTCCTCGTCGATCTCGAGCCCGGTACC

ATGGACGCCGTC

>Diaporthe_australpacifica_BRIP_60163d

????????????????????????????????????????????????????????????

????????????????????????????????????????????????????????????

????????????????????????????????????????????????????????????

????????????????????????????????????????????????????????????

????????????????????????????????????????????????????????????

????????????????????????????????????????????????????????????

????????????????????????????????????????????????????????????

????????????????????????????????????????????????????????????

????????????????????????????????????????????????????????????

????????????????????????????????????????????????????????????

???????????????????????????AACAAGGTCTCCGTGGGTGACCCAGCGGAGGGA

TCATTGCTG-TTC-GGCGCACCCAGAAACCCTTTGTGAACTT-TATTGTTGCCTCGGCAG

GCCGGGAGGCCCCCCGAGACGGGGAGCA-GCCCGCCGGCGGCCAACTAAACTCTTGTTTC

TATAGTGAATCTCTAAATGAATCAAAACTTTCAACAACGGATCTCTTGGTTCTGGCATCG

ATGAAGAACGCAGCGAAATGCGATAAGTAATGTGAATTGCAGAATTCAGTGAATCATCGA

ATCTTTGAACGCACATTGCGCCCCCTGGTATTCCGGGGGGCATGCCTGTTCGAGCGTCAT

TTCAACCCTCAAGCCTGGCTTGGTGATGGGGCACTGAGGAGCAGGCCCTGAAATTCAGTG

GCGAGCTCGCCAGGACCCCGAGCGTAGTAGA-TCTCGCTAAGGCCCTGGCGGTGCCCTGC

TGAAAGAGAAGGAAGGTCAGTAAACATCATGGTTTTTGCGCGGCTGCGTCAAGCCATTTT

CGCCCCTCCCTCTGGATTTTCAGGGTGCGGGGCTTACCGCTTATCTCACTGCCAGCTCCG

ACAACATGCTGATTCCCACACAGCCGCCGAGCTGGGCAAGGGTCGCACCTCAGCCCCACC

ATCGCGACCCACCCCCTGCGACACCAAGATAAGACGCGTCCATTGCTAACATGTTTCTCC

T--CCACAGGTTCACCTTCAGACCGGCCAATGCGTAAGTTGCTGTCATCGCCCGACCTTA

T---CGCCACCCGTAGCACGTTTCCCAGGGTAACCAAATCGGTGCTGCTTTCTGGTGCGT

ACCGAGCCTGCCACCGCGATACTAGCTCGCAATATGCTGACCTCGCAGGCAAACCATCTC

TGGCGAGCACGGTCTCGACAGCAATGGCGTGTATGTACCTCCTATTCCTGCCGATCTCAT

CCTCCCTGATGATCGCACAGTTACAACGGCACTTCCGAGCTCCAGCTCGAGCGCATGAAC

GTCTACTTCAACGAGGTAAGCACGTCTTTTTGA-CCGTCTCGACCTTGCTAATGCGCTCT

CAGGCCTCCGGCAACAAGTATGTGCCTCGCGCCGTCCTCGTCGATCTCGAGCCCGGTACC

ATGGACGCCGNN

>Diaporthe_baccae_CBS_136972

????????????????????????????????????????????????????????????

????????????????????????????????????????????????????????????

????????????????????????????????????????????????????????????

????????????????????????????????????????????????????????????

????????????????????????????????????????????????????????????

???????????????????????????????TCCGCGCCCTCCACCGGAGGTGTCAAGAA

GCCTCACCGCTACAAGCCTGGTACCGTCGCTCTGCGTGAGATCCGTCGTTACCAGAAGAG

CACTGAGCTGCTGATCCGCAAGCTCCCCTTCCAGCGTCTGGTATGCAGGTTCGTGAGATC

GCCCAGGACTTCAAGTCCGACCTCCGCTTCCAGTCCTCCGCCATCGGTGCCCTGCAGGAG

TCCGTCGAGTCTTACCTCGTCTCCCTGTTCGAGGACACCAACTTGTGCGCCATCCACGCC

AAGCGTGTCACCATCCAGTCGGTACGTAACAAGGTCTCCGTTGGTGAACCAGCGGAGGGA

TCATTGCTGCCCCTGGCGCACCCAGAAACCCTTTGTGAACTTTTACTGTTGCCTCGGCAG

GCCGTGGGGTCCCTT--AACGAGGAGCA-GCCGGCCGGTGGCCAAATTAACTC-TGTTTT

TAAACTGAAACTCTAAATGAATCAAAACTTTCAACAACGGATCTCTTGGTTCTGGCATCG

ATGAAGAACGCAGCGAAATGCGATAAGTAATGTGAATTGCAGAATTCAGTGAATCATCGA

ATCTTTGAACGCACATTGCGCCCTCTGGTATTCCGGAGGGCATGCCTGTTCGAGCGTCAT

TTCAACCCTCAAGCCTGGCTTGGTGTTGGGGCACTGGAGAGCAGGCCCTGAAATATAGTG

GCGAGCTCGCCAGGACTCCGAGCGTAGTAGACCCTCGCTAAGG-CCTGGCGGTGCCCTGC

CTGAAGAGAAGGAAGGTTAGTAAACACCATCATGTTCGTGTGGCTTCGTCGGCGCATTTT

CACCCCGCCCTCTGGATTTTCAGGGTGCGGGGCTTAGAGCTTATCTCACA---------A

AAAACATGCTGACTTCTTCACAGCCGCCGAGCTTGGCAAGGGTTCGCGCCTGAACCCCAC

CATCGCGACACACCCCTAGGACACTCAGGTAAGACGCGTCGATTGCTAACATGTTTTTCT

CGCCTACAGGTTCACCTTCAGACCGGTCAATGCGTAAGTTGCTGTCACACCGGA-CCTTA

TCATCGCCACCTGTAGCACGTTTCCCAGGGTAACCAAATCGGTGCTGCTTTCTGGTGCGT

CC-AAGTCAACCACCGCGAGTCGACCTCGAAGCATACTGACCTCGCAGGCAAACCATCTC

TGGCGAGCACGGTCTCGACAGCAATGGCGTGTACGCACCTCCTATTCCTGTCTTTCTCGT

-TGCACTGACAATCACACAGTTACAACGGCACTTCCGAGCTCCAGCTCGAGCGCATGAAC

GTCTACTTCAACGAGGTAAGTACGTTG--TT-ACCATCTACAGCTTTGCTAACGCGTTAT

CAGGCCTCCGGCAACAAGTATGTGCCCCGCGCCGTCCTCGTCGATCTCGAGCCCGGTACC

ATGGACGCCGNN

>Diaporthe_batatas_CBS_122_21

CTTTGTAAGTTATCTTCGCCTTGCCCGCCGTTTCCGTTGCGCATGCTAACCGACCGTTTT

CGGCATGTAGGATAAGGATGGCGATGGTTAGTGCGGTCACTCTCAGCTAGCACGCGTCAT

ACTCGATCCACCGCGACGGTCTGCGCGCGAGCGACCGTCACCGATATCACGAGATACTAA

GGTGTAGGACAAATCACCACCAAGGAGCTCGGCACAGTCATGCGGTCCCTTGGTCAGAAC

CCTTCCGAGTCCGAGCTGCAGGACATGATCAACGAGGTCGACGCCGACAACAATGGCACC

ATTGACTTTGGTAAGTCTGGATTCCCACACATCCGCGCCCTCCACCGGAGGTGTCAAGAA

GCCTCACCGCTACAAGCCTGGTACCGTCGCTCTGCGTGAGATCCGTCGCTACCAGAAGAG

CACCGAGCTGCTGATCCGCAAGCTCCCCTTCCAGCGTCTGGTATGCAGGTCCGTGAGATC

GCCCAGGACTTCAAGTCCGACCTGCGCTTCCAGTCCTCCGCCATCGGTGCTCTCCAGGAG

TCCGTCGAGTCTTACCTCGTCTCCCTCTTTGAGGACACCAACCTGTGCGCCATCCACGCC

AAGCGTGTCACCATCCAGTCGGTACGTAACAAGGTCTCCGTTGGTGAACCAGCGGAGGGA

TCATTGCTGCCCCTGGCGCACCCAGAAACCCTTTGTGAACTT---CTGTTGCCTCGGCAG

GCCGGGAGGCCCCCCGAGACGGGGAGCA-GCCCGCCGGCGGCCAACCAAACTCTTGTTTC

TATAGTGAATCTCTAAATGAATCAAAACTTTCAACAACGGATCTCTTGGTTCTGGCATCG

ATGAAGAACGCAGCGAAATGCGATAAGTAATGTGAATTGCAGAATTCAGTGAATCATCGA

ATCTTTGAACGCACATTGCGCCCTCTGGTATTCCGGAGGGCATGCCTGTTCGAGCGTCAT

TTCAACCCTCAAGCCTGGCTTGGTGATGGGGCACTGGGGGGCAGGCCCTGAAATCTAGTG

GCGAGCTCGCCAGGACCCCGAGCGTAGTAGA-TCTCGCTAAGGCCCTGGCGGTGCCCTGC

TGAAANNNNNNNNNNNNNNNNNNNNNNCCTTGTGTCTGTGACGCTTCGTCAAAGCATTTT

CACCCCTCCCTCTGGATTTTCAGGGTGCGGGGCTTACCGCTTATCTCAGTGAC-ATTC-G

GAAACATGCTGATCTCTACACAGCCGCCGAGCTGGGTAAGGGTCGCACCTAAGCCCCACC

ATCGCGACCCACCCCCTGCGACACCCAGGTAACACGCGTCGATTGCTAACGTGTTTTTCT

CGCCCACAGGTTCACCTCCAGACCGGCCAATGCGTAAGTTGCTGTCACCGCCCGACCTTA

T---CGCCACCCGTAGCACGTTTCCCAGGGTAACCAAATCGGTGCTGCTTTCTGGTGCGT

CCCAAGCCTACCACCGCGAGGCGAGCTCGAAGCATACTGACCTCGTAGGCAAACCATCTC

TGGCGAGCACGGTCTCGACAGCAATGGCGTGTATGCACCTCCTAATCC------CCTCGT

CCTCCCTGACGATCGCACAGTTACAACGGCACTTCCGAGCTCCAGCTCGAGCGCATGAAC

GTCTACTTCAACGAGGTAAGTACGTCGTCTTGA-CCGCCTCGCCCTTGCTAACGCGTTTT

CAGGCCTCCGGCAACAAGTATGTTCCTCGCGCCGTCCTCGTCGATCTCGAGCCCGGTACC

ATGGACGCCGTC

>Diaporthe_bauhiniae_CFCC_53071

CTTTGTAATCTCCAGCCGACACGCCCGCCTTTGTTGTCGCGCATGCTAACGGACCGTTTT

CGGCTTGCAGGATAAGGATGGCGATGGTTAGTGCGGCCGCTCTCACCTAGCACGCGTCAT

GCTCGATCCGCCGCGACAGCCTGCGCGCAAGCTGGCGATATCACTTTCACGAGTTGCTGA

GGTGTAGGACAAATCACCACCAAGGAGCTGGGCACGGTCATGCGGTCCCTGGGCCAGAAC

CCGTCCGAGTCTGAGCTGCAAGACATGATTAACGAGGTCGACGCCGACAACAATGGCACC

ATTGACTTTGGTACGTCCGGATGCGCGCCCATCCGCGCCCTCCACCGGAGGTGTCAAGAA

GCCTCACCGCTACAAGCCTGGTACCGTCGCTCTGCGTGAGATCCGTCGCTACCAGAAGAG

CACCGAGCTGCTGATCCGCAAGCTCCCCTTCCAGCGTCTGGTAAGCAGGTCCGTGAGATC

GCCCAGGACTTCAAGTCCGACCTCCGCTTCCAGTCCTCGGCCATCGGTGCCCTGCAGGAG

TCGGTCGAGTCCTACCTCGTCTCCCTCTTCGAGGACACCAACCTGTGCGCCATCCACGCC

AAGCGTGTCACCATCCAGTCGGTATGTNNNNNNNNNNNNNNNNNNNNNNNNNNNNNNNNN

NNNNNNNNNNNNNNNNNNNNNNNNNNNNCCCTTTGTGAACTT-TACTGTTGCCTCGGTTA

GCTGGGGCCCCTCACCCGGTGAGGAGCA-GCGCGCCGGCGGCCCACCTAACTCTGTTTCT

T--TGTGAATCTCTAAATGAATCAAAACTTTCAACAACGGATCTCTTGGTTCTGGCATCG

ATGAAGAACGCAGCGAAATGCGATAAGTAATGTGAATTGCAGAATTCAGTGAATCATCGA

ATCTTTGAACGCACATTGCGCCCTCTGGTATTCCGGAGGGCATGCCTGTTCGAGCGTCAT

TTCAACCCTCAAGCCTGGCTTGGTGTTGGGGCACTGGAGAGCAGGCCCTGAAATTCAGTG

GCGAGCTCGCCAGGACTCCGAGCGTAGTAG-ATCTCGTTAAGG-CCTGGCGGTGNNNNNN

NNNNNNNNNNNNNNNNNNNNTAAACATATCAGTGGTTGCGACTGTCTGTTGGCGCATTTT

CACCCCTCGCCCTGGAATTTCAGGGTGCGGGGCTTAGGGCTTATCTCACCACTATAATCA

CAACCACGCTGATATTCCCACAGCCGCCGAGCTTGGTAANNNTCGCACCCAAGCCCCACC

ATCGCGACCCACCCCCTGCGACACCCAGATAAGACGCGTCGATTGCTAACGTGTTTTTCT

CGCCCACAGGTTCACCTTCAGACCGGCCAATGCGTAAGTTGCTGTCACCGCCGGACCTTA

TCATCGCCACCTGTAGCACGTTTCCCAGGGTAACCAAATCGGTGCTGCTTTCTGGTGCGT

CCCAAGCCCACCACCGCGGTACGACCTCGAAGCATACTGACCCCGCAGGCAAACCATCTC

TGGCGAGCACGGCCTCGACAGCAATGGCGTGTATGTACCTCCTATTCCTGCCCATCTCGT

CCTCCTTGACAATCGCACAGTTACAACGGCACTTCCGAGCTCCAGCTCGAGCGCATGAAT

GTCTACTTCAACGAGGTATGTACGCCGTTCTGAGCATCCACAGCCTTGCTAACGCGTTAT

CAGGCCTCCGGCAACAAGTATGTGCCTCGCGCCGTCCTCGTCGATCTCGAGCCCGGTACC

ATGGACGCCGTN

>Diaporthe_beasleyi_BRIP_59326a

????????????????????????????????????????????????????????????

????????????????????????????????????????????????????????????

????????????????????????????????????????????????????????????

????????????????????????????????????????????????????????????

????????????????????????????????????????????????????????????

????????????????????????????????????????????????????????????

????????????????????????????????????????????????????????????

????????????????????????????????????????????????????????????

????????????????????????????????????????????????????????????

????????????????????????????????????????????????????????????

???????????????????????????AACAAGGTCTCCGTTGGTGAACCAGCGGAGGGA

TCATTGCTGCCCCAGGCGCACCCAGAAACCCTTTGTGAACTTTTACTGTTGCCTCGGCAT

GCCGGGGGGCCCCTCGAGACGAGGAGCAGGCACGCCGGCGGCCAAGTTAACTCTTGTTTT

TACACTGAAACTCTAAATGAATCAAAACTTTCAACAACGGATCTCTTGGTTCTGGCATCG

ATGAAGAACGCAGCGAAATGCGATAAGTAATGTGAATTGCAGAATTCAGTGAATCATCGA

ATCTTTGAACGCACATTGCGCCCTCTGGTATTCCGGAGGGCATGCCTGTTCGAGCGTCAT

TTCAACCCTCAAGCCTGGCTTGGTGATGGGGCACTGAAGGGCACGCCCTCAAATCTAGTG

GCGAGCTCGCCAGGACCCCGAGCGCAGTAGACCCTCGCTAAGGCCCTGGCGGTGCCCTGC

TGAAANAGAAGGAAGGTTAGTAAACATCACCATGTTCATGCGGCCTCGTCAGCGCATTTT

CACCCCTCGCTCTGGACTTTCAGGGTGCGGGGCTTAGAGCTTATCTACCG-ACACCAAGG

AAATCATGCTGACGTCTACACAGCCGCCGAGCTCGGCAAGGGTCGCACCTGAGCCCCACC

ATCGCGACCCACCCCCTGGGACACCCAGGTAAGACGCGTCGATTGCTAACGTGTTTTTCT

CGCCTGCAGGTTCACCTTCAGACCGGCCAATGCGTAAGTTGCTGTCACAGCGGA-CCTTA

TCATCGCCACCTGTAGCACGTTTCCCAGGGTAACCAAATCGGTGCTGCTTTCTGGTGCGT

CC-AAGCCCACCACCGCGGTACGACCTCGAAGCATACTGACCTCGTAGGCAAACCATCTC

TGGCGAGCACGGCCTCGACAGCAATGGCGTGTATGCACCTCCTATTTCTGCCCTTCTCGT

CTGCCCTGACAATCACACAGTTACAATGGCTCTTCCGAGCTCCAGCTCGAGCGCATGAAC

GTCTACTTCAACGAGGTAAGTACGTCG--CTGGCCATCTACAACCTTGCTAACGCGTTAT

CAGGCCTCCGGCAACAAGTATGTTCCCCGCGCCGTCCTCGTCGATCTCGAGCCCGGTACC

ATGGACGCCGTC

>Diaporthe_beckhausii_CBS_138_27

CTTTGTAAGTTCTCTTC------CCCGCCGATGCTGTTGCGCATGCTAACGGACCGTTTC

CGGCCTCCAGGATAAGGATGGCGATGGTTAGTGCGGCTGCCTCTACTCTATACGCGTCCC

AATCTATCTGCCGCGACGGCTTGCGCGCGATCAAGCGCCATCACTACCAGGAGTTGCTAA

GGTGTAGGACAAATCACCACAAAGGAGCTCGGAACGGTCATGCGATCGCTGGGTCAGAAC

CCGTCTGAGTCTGAGCTGCAGGATATGATCAACGAGGTCGATGCCGACAACAATGGCACC

ATTGACTTTGGTACGTTCAGATATCTGCTCATCCGCGCCCTCCACCGGAGGTGTCAAGAA

GCCTCACCGCTACAAGCCTGGTACCGTCGCTCTTCGTGAGATTCGTCGCTACCAGAAGTC

CACTGAGCTCCTGATCCGCAAGCTCCCCTTCCAGCGTCTGGTATGCAGGTTCGTGAGATC

GCCCAGGACTTCAAGTCCGACCTCCGTTTCCAGTCCTCCGCCATCGGTGCCCTGCAGGAG

TCTGTCGAGTCTTACCTCGTCTCCCTGTTCGAGGACACCAACTTGTGCGCCATCCACGCC

AAGCGTGTCACCATCCAGTCGGTACGTAACAAGGTCTCCGTTGGTGAACCAGCGGAGGGA

TCATTGTTGCTCCG-GCGCATCCAGAAACCCTTTGTGAACTTATACTGTTGCCTCGGCAG

GCTGGGGGGCCCCCCCCGGTGGGGAGCAGGCCCGCCGGCGGCCAAGTTAACTCTTGTTTT

TACACTGAAACTCTAAATGAATCAAAACTTTCAACAACGGATCTCTTGGTTCTGGCATCG

ATGAAGAACGCAGCGAAATGCGATAAGTAATGTGAATTGCAGAATTCAGTGAATCATCGA

ATCTTTGAACGCACATTGCGCCCTCTGGTATTCCGGAGGGCATGCCTGTTCGAGCGTCAT

TTCAACCCTCAAGCCTGGCTTGGTGATGGGGCACTGCAGGGCAGGCCCTTAAATTCAGTG

GCGAGCTCGCCAGGACCCCGAGCGCAGTAGACCCTCGCTAAGGTCTTGGTGCGGCCTGGC

TGAAAGAGAAGGAAGGTTAGTAAATATGCACACGCTTGCAAGGCTGCATCGTCGCATTTT

CACCCCTCGTTCTGGACTTTCAGGGTGCGGGGCTTAGAGCTTATCTCACAACCATCACCT

GAATTGTGCTGACACTTAACCAGCCGCCGAGCTTGGTAAGGGTCGCACCTGAGCCCCACC

ATCGCGACCCACCCCCTGAGACACTCAGATAAGACGCGTCGAATGCTGACTTTTTTTTCT

CCTTCACAGGTCCACCTTCAGACCGGCCAATGCGTAAGTTGCTGTCACCACCGCACCTTA

T---CGCCCCCTGTAGCACGTTTTCCAGGGTAACCAAATCGGTGCTGCTTTCTGGTGCGT

T----GTCCATCATCGCGACACGCCCTCGCAACATATTGACTTCGTAGGCAAACCATCTC

TGGCGAGCACGGCCTCGACAGCAATGGCGTGTACGTACCTCCTATTCCTACCCGTCTCGT

CCGCCCTGACAGCTTCACAGTTACAACGGCTCTTCTGAGCTCCAGCTCGAGCGCATGAAC

GTCTACTTCAACGAGGTCAGTATTTTATACCCACGATCTTCAGCTTTGCTGACACCTTAT

CAGGCCTCCGGCAACAAGTATGTTCCTCGCGCTGTCCTCGTCGATCTCGAGCCCGGTACC

ATGGACGCCGTC

>Diaporthe_beilharziae_BRIP_54792

????????????????????????????????????????????????????????????

????????????????????????????????????????????????????????????

????????????????????????????????????????????????????????????

????????????????????????????????????????????????????????????

????????????????????????????????????????????????????????????

????????????????????????????????????????????????????????????

????????????????????????????????????????????????????????????

????????????????????????????????????????????????????????????

????????????????????????????????????????????????????????????

????????????????????????????????????????????????????????????

???????????????????????????AACAAGGTCTCCGTTGGTGAACCAGCGGAGGGA

TCATTGCTGCCTC-GGCGCACCCAGAAACCCTTTGTGAACTTA--TCGTTGCCTCGGTCG

GCCGGGAGGCCCCCTGAAACGGGGAGCA-GCCCGCCGGCGGCCAACCAAACTCTTGTTTC

TATAGTGAATCTCTAAATGAATCAAAACTTTCAACAACGGATCTCTTGGTTCTGGCATCG

ATGAAGAACGCAGCGAAATGCGATAAGTAATGTGAATTGCAGAATTCAGTGAATCATCGA

ATCTTTGAACGCACATTGCGCCCCCTGGTATTCCGGGGGGCATGCCTGTTCGAGCGTCAT

TTCAACCCTCAAGCCTGGCTTGGTGATGGGGCACTAGGGAGTAGGCCCTGAAATTCAGTG

GCGAGCTCGCCAGGACCCCGAGCGTAGTAGA-TCTCGCTAAGGCCCTGGCGGTGCCCTGC

TGAAANNGAAGGAAGGTCAGTTAATATCATGGTGTTTGCGCGGCTGCGTCAAGCCATTTT

CACCCCTCCCTCTGGATTTTCAGGGTGCGGGGCTTACCGCTTATCTCACTGCCAGCACCG

ACAACATACTGATTCCCACACAGCCGCCGAGCTGGGCAAGGGTCGCACCTCAGCCCCACC

ATCGCGACCCACCCCCTGCGACACCCAGATAAGACGCGTCGATTGCTAACATGTTTTTCT

TGCCCACAGGTTCACCTTCAGACCGGCCAATGCGTAAGTTGCTGTCACCGCCCGACCTTA

T---CGCCACCCGTAGCACGTTTCCCAGGGTAACCAAATCGGTGCTGCTTTCTGGTGCGT

ACCGAGCCTGCCACTGCGAGACCACCTCCTAGCATATTGACCTCGTAGGCAAACCATCTC

TGGCGAGCACGGTCTCGACAGCAATGGCGTGTATGTACCTCCTATTCCGGCCCATCTCGT

CCTCCCTGATGATCGCACAGTTACAATGGCTCTTCCGAGCTCCAGCTCGAGCGCATGAAC

GTCTACTTCAACGAGGTAAGCACGTCTTTTTGA-CCGTCTCGGCCTTGCTAACGCGCTCT

CAGGCCTCCGGCAACAAGTATGTGCCTCGCGCCGTCCTCGTCGATCTCGAGCCTGGTACC

ATGGACGCCGTC

>Diaporthe_benedicti_ATCC_MYA_4970

NNNNNNNNNNNNNNNNNNNNNNNCCCGGCTTTGCTGTTGCGCATACTAACGGACCGTTTT

CGGCCTCCAGGATAAGGATGGCGATGGTTAGTGCGGCTGCCTCCAACCTATACGCGTCAC

GATCCATTCGCCGCGACAGCTTGCGCGCGATCAAGCGCTTTCACTACCAGGAGTTGCTAA

AGTGTAGGACAAATCACCACAAAGGAGCTCGGAACGGTCATGCGATCGCTGGGTCAGAAC

CCGTCCGAGTCTGAGCTGCAAGATATGATCAACGAGGTTGATGCCGACAACAATGGCACC

ATCGACTTCGGTACGTTCAGGTGTCTGCTCA?????????????????????????????

????????????????????????????????????????????????????????????

????????????????????????????????????????????????????????????

????????????????????????????????????????????????????????????

????????????????????????????????????????????????????????????

???????????????????????????NNNNNNNNNNNNNNNNNNNNNNNNNNNNNNNNN

NNNNNNNNNNNNNNNNNNNNNNNNNNNNCCCTTTGTGAACTTACACTGTTGCCTCGGCAG

GCCGGGGGGCCCCCTTTGGTGGGGAGCAGGCCCGCCGGCGGCCAAGTCAACTCTTGTTTT

TACACTGGAACTCTAAATGAATCAAAACTTTCAACAACGGATCTCTTGGTTCTGGCATCG

ATGAAGAACGCAGCGAAATGCGATAAGTAATGTGAATTGCAGAATTCAGTGAATCATCGA

ATCTTTGAACGCACATTGCGCCCTCTGGTATTCCGGAGGGCATGCCTGTTCGAGCGTCAT

TTCAACCCTCAAGCCTGGCTTGGTGATGGGGCACTGCAGGGCAGGCCCCCAAATTCAGCG

GCGGGCTCGCCGGGACCCCGAGCGCAGTAGACCCTCGCTAAGGCCCCGGTGCGGCCCTGC

NNNNNNNNNNNNNNNGTTAGTAAATATCCCCACGCTTGCACTGCTGCGTCATCGCATTTT

CACCCCTCGTTCTGGATTTTCA--GTGCGGGGCTTAGCGCTTATCTCACAATCACAACAA

AACACATGCTGACGCTTTACCAGCCGCCGAGCTTNNNNNNNN??????????????????

????????????????????????????????????????????????????????????

????????????????????????????????????????????????????????????

????????????????????????????????????????????????????????????

????????????????????????????????????????????????????????????

????????????????????????????????????????????????????????????

????????????????????????????????????????????????????????????

????????????????????????????????????????????????????????????

????????????????????????????????????????????????????????????

????????????

>Diaporthe_berteroae_BRIP_57900a

????????????????????????????????????????????????????????????

????????????????????????????????????????????????????????????

????????????????????????????????????????????????????????????

????????????????????????????????????????????????????????????

????????????????????????????????????????????????????????????

????????????????????????????????????????????????????????????

????????????????????????????????????????????????????????????

????????????????????????????????????????????????????????????

????????????????????????????????????????????????????????????

????????????????????????????????????????????????????????????

???????????????????????????AACAAGGTCTCCGTTGGTGAACCAGCGGAGGGA

TCATTGCTGCTTC-GGCGCACCCAGAAACCCTTTGTGAACTT-TATTGTTGCCTCGGTAG

GCCGGGAGGCCCCCTGAAACAGGGAGCA-GCCCGCCGGCGGCCAACCAAACTCTTGTTTC

TATAGTGAATCTCTAAATGAATCAAAACTTTCAACAACGGATCTCTTGGTTCTGGCATCG

ATGAAGAACGCAGTGAAATGCGATAAGTAATGTGAATTGCAGAATTCAGTGAATCATCGA

ATCTTTGAACGCACATTGCGCCCTCTGGTATTCCGGAGGGCATGCCTGTTCGAGCGTCAT

TTCAACCCTCAAGCCTGGCTTGGTGATGGGGCACTGGAGGTTAGGCCCTGAAATCTAGTG

GCGAGCTCGCCAGGACCCCGAGCGTAGTAAA-TCTCGCTAAGGCCCTGGCGGTGCCCTGC

TGAANNNGAAGGAAGGTTAGTAAACATCGTTGTGTCTGCGAGGCTTCGTCAAGGCATTTT

CACCCCTCCCTCTGGATTTTCAGGGTGCGGGGCTTACCGCTTATCTCAGTGAC-GTCCCG

AAAAGATGCTGATTTCTGAACAGCCGCCGAGCTGGGCAAGGGTCGCACCTGAGCCCCACC

ATCGCGACCCACCCCCTGCGACACCCAGATAACACGCGTCGATTGCTAACATGTTTTTCT

CGCCCACAGGTTCACCTTCAGACCGGCCAATGCGTAAGTTGCTGTCACCGCCAGACCTTA

T---CGCCACCCGTAGCACGTTTCCCAGGGTAACCAAATCGGTGCTGCTTTCTGGTGCGT

CTCAAGCCTACCACCGCGAGGCGAGCTCGAAGCATACTGACCTCGTAGGCAAACCATCTC

TGGCGAGCACGGTCTCGACAGCAATGGCGTGTATGCACCTCCTATTCCTGCCCACCTGGT

CCTCCCTGATGATCGCACAGTTACAACGGCACTTCCGAGCTCCAGCTCGAGCGCATGAAC

GTCTACTTCAACGAGGTATGTACGTCGTTTTGA-ACCTCTCGCCCTTGCTAACGCGTTTT

CAGGCTTCCGGCAACAAGTATGTGCCCCGCGCCGTCCTCGTCGATCTCGAGCCCGGTACC

ATGGACGCCGTC

>Diaporthe_betulae_CFCC_50469

CTTTGTAATCTCTAGCCGACATGCCCGCCCTTGCTGGTGCGCATGCTAACGGCCCGTTTT

CGGCTTGTAGGATAAGGACGGCGATGGTTAGTGCGGCCGCTCTCACCTAGCACGCGTCAT

GTTCGATCCACCGCGACAGTCTGCGCGCAGCCAAGCGTTATCACTATCACGAGTTGCTGA

GGTGTAGGACAAATCACCACCAAGGAGCTCGGCACGGTCATGCGATCCCTGGGCCAGAAC

CCGTCCGAGTCTGAGCTGCAAGATATGATTAACGAGGTCGACGCCGACAACAATGGCACC

ATTGACTTNNNNNNNNNNNNNNNNNNNNNNNTCCGCGCCCTCCACCGGAGGTGTCAAGAA

GCCTCACCGCTACAAGCCTGGTACCGTCGCTCTGCGTGAGATCCGTCGCTACCAGAAGAG

CACCGAGCTGCTGATCCGCAAGCTCCCTTTCCAGCGTCTGGTATGCAGGTCCGTGAGATC

GCCCAGGACTTCAAGTCCGACCTCCGCTTCCAGTCTTCCGCCATCGGCGCCCTGCAGGAG

TCGGTCGAGTCTTACCTCGTCTCCCTCTTCGAGGACACCAACCTGTGCGCCATCCACGCC

AAGCGTGTCACCATCCAGTCGGTATGTNNNNNNNNNNNNNNNNNNNNNNNNNNNNNNNNN

NNNNNNNNNNNNNNNNNNNNNNNNNNNNCCCTTTGTGAACTTTTACTGTTGCCTCGGCAC

GCCGGGGGGCCCCTCTCCTGGAGGAGCAGGCACGCCGGCGGCCAACCTAACTCTTGCTTT

TACACTGAAACTCTAAATGAATCAAAACTTTCAACAACGGATCTCTTGGTTCTGGCATCG

ATGAAGAACGCAGCGAAATGCGATAAGTAATGTGAATTGCAGAATTCAGTGAATCATCGA

ATCTTTGAACGCACATTGCGCCCTCTGGTATTCCGGAGGGCATGCCTGTTCGAGCGTCAT

TTCAACCCTCAAGCCTGGCTTGGTGATGGGGCACTGAAGGGCAGGCCCTGAAATTCAGTG

GCGAGCTCGCCAGGACCCCGAGCGCAGTAGACCCTCGCTAAGGCCCTGGCGGTGCCCTGC

TGAAAGAGAAGGAAGGTTAGTAAATACCATCATGCTCGCGCGGCTTCGTCAGCGCATTTT

CACCCCTCGCTTTGGATTTTCAGGGTGCGGGGCTTAGGGCTTATCTCACCACCACCACCG

AACATATGCTGATACTTACACAGCCGCCGAGCTTGGCAAGGGTCGCACCTGAGCCCCACC

ATCGCGACCCACTCCCTGGGACACCCAGATAAGACGCGTCGATTGCTAACGTGTTTTTCT

CGCCTCTAGGTTCACCTTCAGACCGGCCAATGCGTAAGTTGCTGTCAC-AGCGGACCTTA

TCATCGCCACCTGTAGCACGTTTCCCAGGGTAACCAAATCGGTGCTGCTTTCTGGTGCGT

TCCAAGTTCACCGCCGCGATACGACCTCGAAGCATGCTGACCTCGTAGGCAAACCATCTC

TGGCGAGCACGGCCTCGACAGCAATGGCGTGTATGCACCTCCTATTCCTGCCCATCTTGG

CTTCCCTGACAATCGCACAGTTACAACGGCACTTCTGAGCTCCAGCTCGAGCGCATGAAC

GTCTACTTCAACGAGGTAAGTACATCATTCCGACCATCTCCAACCTCGCTAACGCGTCAT

CAGGCCTCTGGCAACAAGTATGTTCCTCGCGCCGTCCTCGTCGATCTCGAGCCCGGTACC

ATGGACGCCGTC

>Diaporthe_betulicola_CFCC_51128

CTTTGTAAGTCATTCTCCACATGCCCGCCCTTGCTGTTGCGCATGCTAAAGGACCGTTTT

CGGCCTGCAGGATAAGGATGGCGATGGTAAGTGTGGCCGCACCCACCGAGCACGCGTCAT

GCTCGATCCGCCGTGACGGCCTGCGCGCGAGCAGGCGTCAGCACTATCAGGGATTGCTAA

GGCGTAGGACAAATCACCACGAAGGAGCTCGGCACTGTCATGCGATCCCTGGGCCAGAAC

CCATCCGAGTCTGAGCTGCAAGATATGATTAACGAGGTCGATGCCGACAACAACGGCACC

ATTGACTTTGGTATGTCCAGACGCTCGCCTATCCGCGCCCTCCACCGGAGGTGTCAAGAA

GCCTCACCGCTACAAGCCTGGTACCGTCGCTCTGCGTGAGATCCGTCGTTACCAGAAGAG

CACCGAGCTGCTGATCCGCAAGCTGCCTTTCCAGCGTCTGGTACGCAGGTTCGTGAGATC

GCTCAGGACTTCAAGTCCGACCTCCGCTTCCAGTCTTCCGCCATCGGTGCCCTGCAGGAG

TCCGTCGAGTCTTACCTCGTCTCCCTCTTTGAGGACACCAACCTGTGCGCCATCCACGCC

AAGCGTGTCACCATCCAGTCGGTATGTNNNNNNNNNNNNNNNNNNNNNNNNNNNNNNNNN

NNNNNNNNNNNNNNNNNNNNNNNNNNNNCCCTTTGTGAACTTTTACTGTTGCCTCGGCAG

GCCGGGGGGCCCCTCGAGACGAGGAGCAGGCCCGCCGGCGGCCAAGTTAACTCTTGTTTT

TACACTGAAACTCTAAATGAATCAAAACTTTCAACAACGGATCTCTTGGTTCTGGCATCG

ATGAAGAACGCAGCGAAATGCGATAAGTAATGTGAATTGCAGAATTCAGTGAATCATCGA

ATCTTTGAACGCACATTGCGCCCTCTGGTATTCCGGAGGGCATGCCTGTTCGAGCGTCAT

TTCAACCCTCAAGCCCGGCTTGGTGATGGGGCACTGAGAAGCAGGCCCTGAAACTCAGTG

GCGAGCTCGCCAGGACCCCGAGCGCAGTAGACCCTCGCTAAGGCCCTGGCGGTGCCCTGC

TGAAAGAGAAGGAAGGTTAGTAAACATCATCGCTCTTGCGCGGCTCCGCCAGCGCATTTT

CACCCCTCGTTCTGGATTTTCAGGGTGCGGGGCTTAGAGCTTATCTCACTACCACC---G

GAAAGATGCTAACAATTATACAGCCGCCGAGCTTGGCAAGGGTCGCACCTGAGCCCCACC

ATCGCGACCCACCCCCTGCGACACCCGGATAAGACGCGTCGATTGCTAACGTGTTTTCCT

CGGCCGCAGGTTCACCTTCAGACCGGCCAATGCGTAAGTTGCTGTCACACCGGACCCTTA

T---CGCCACCTGTAGCACGTTTCCCAGGGTAACCAAATCGGTGCTGCTTTCTGGTGCGT

CCCAAACTCACCACCGCGACACGACCTCGCGGCATACTGACCCCGTAGGCAAACCATCTC

TGGCGAGCACGGCCTCGACAGCAATGGCGTGTATGCACCTCCTATTCCTGCACATCTCGC

CCTCCTTGACAATCGCACAGTTACAACGGCACTTCCGAGCTCCAGCTCGAGCGCATGAGC

GTCTACTTCAACGAGGTATGTACATTGTTTCGACCGTCTTCAACCTCGCTAACGCGTTAT

TAGGCCTCCGGCAACAAGTATGTTCCTCGCGCGGTCCTCGTCGATCTTGAGCCCGGTACC

ATGGACGCCGTC

>Diaporthe_betulina_CFCC_52562

NNNNNNNNNNNNNNNNNNNNNNNNCCGCCCTTGCTGGTGCGCATGCTAACGGCCCGTTTT

CGGCTTGTAGGATAAGGACGGCGATGGTTAGTGCGGCCGCTCTCACCTAGCACGCGTCAT

GTTCGATCCACCGCGACAGTCTGCGCGCAGCCAAGCGTTATCACTATCACGAGTTGCTGA

GGTGTAGGACAAATCACCACCAAGGAGCTCGGCACGGTCATGCGATCCCTGGGCCAGAAC

CCGTCCGAGTCTGAGCTGCAAGATATGATTAACGAGGTCGACGCCGACAACAATGGCACC

ATTGACTTTGGTACGTCCAGATGCTCGCGCTTCCGCGCCCTCCACCGGAGGTGTCAAGAA

GCCTCACCGCTACAAGCCCGGTACCGTCGCTCTGCGTGAGATCCGTCGCTACCAGAAGAG

CACCGAGCTGCTGATCCGCAAGCTGCCCTTCCAGCGTCTGGTATGCAGGTCCGTGAGATC

GCCCAGGACTTCAAGTCCGACCTCCGCTTCCAGTCTTCCGCCATCGGCGCCCTGCAGGAG

TCGGTCGAGTCTTACCTCGTCTCCCTCTTCGAGGACACCAACCTGTGCGCCATCCACGCC

AAGCGTGTCACCATCCAGTCGGTATGTNNNNNNNNNNNNNNNNNNNNNNNNNNNNNNNNN

NNNNNNNNNNNNNNNNNNNNNNNNNNNNCCCTTTGTGAACTTTTACTGTTGCCTCGGCAT

GCCGGGGGGCCCCTCTTCTGGAGGAGCAGGCACGCCGGCGGCCAACCTAACTCTTGTTTT

TACACTGAAACTCTAAATGAATCAAAACTTTCAACAACGGATCTCTTGGTTCTGGCATCG

ATGAAGAACGCAGCGAAATGCGATAAGTAATGTGAATTGCAGAATTCAGTGAATCATCGA

ATCTTTGAACGCACATTGCGCCCTCTGGTATTCCGGAGGGCATGCCTGTTCGAGCGTCAT

TTCAACCCTCAAGCCTGGCTTGGTGATGGGGCACTGAAAAGCAGGCCCTGAAATTCAGTG

GCGAGCTCGCCAGGACCCCGAGCGCAGTAGACCCTCGCTAAGGCCCTGGCGGTGCCCTGC

TGAAAGAGAAGGAAGGTTAGTAAATACCATCATGCTCGCGCGGCTTCGTCAGCGCATTTT

CACCCCTCGCTTTGGATTTTCAGGGTGCGGGGCTTAGGGCTTATCTCACCACCACCACCG

AACATATGCTGATATCTACACAGCCGCCGAGCTTGGTAAGGGTCGCACCTGAGCCCCACC

ATCGCGACCCACCCCCTGGGACACCCAGATAAGACGCGTCGATTGCTAACGTGTTTTTCT

CGCCTCTAGGTTCACCTTCAGACCGGCCAATGCGTAAGTTGCTGTCAC-AGCGGACCTTA

TCATCGCCACCTGTAGCACGTTTCCCAGGGTAACCAAATCGGTGCTGCTTTCTGGTGCGT

TCCAAGTCCACCGCCGCGATACGACCTCGAAGCATGCTGACCTCGTAGGCAAACCATCTC

TGGCGAGCACGGCCTCGACAGCAATGGCGTGTATGCACCTCCTATTCCTGCCCATCTTGG

CTTCCCTGACAATCGCACAGTTACAACGGCACTTCTGAGCTCCAGCTCGAGCGCATGAAC

GTCTACTTCAACGAGGTAAGTACATCATTCCGACCATCTCCAACCTCGCTAACGCGTCAT

CAGGCCTCAGGCAACAAGTATGTTCCTCGCGCCGTCCTCGTCGATCTCGAGCCCGGTACC

ATGGACGCCGNN

>Diaporthe_juglandia_CBS_121004

NNNNNNNNNCTCCAGCCGACACGCCCGCCCTTGCTGGTGCGCATGCTAACGGACCGTTTT

CGGCTTGTAGGATAAGGATGGCGATGGTTAGTGCGGCCGCTCCCACCTAGCACGCGTCAT

GTTCGATCCGCCGCGACAGCCTGCGCGCAACCAAGCGTTATCACTATCACGAGTTGCTGA

GGTGTAGGGCAAATCACCACCAAGGAGCTCGGCACGGTCATGCGATCCCTGGGTCAGAAC

CCGTCCGAGTCTGAGCTGCAAGATATGATTAACGAGGTCGACGCCGACAACAATGGCACC

ATTGACTTTGGTACGTCCAGATGCTCGCGCTTCCGCGCCCTCCACCGGAGGTGTCAAGAA

GCCTCACCGCTACAAGCCTGGTACCGTCGCTCTGCGTGAGATCCGTCGCTACCAGAAGAG

CACCGAGCTGCTGATCCGCAAGCTCCCTTTCCAGCGTCTGGTATGCAGGTCCGTGAGATC

GCCCAGGACTTCAAGTCCGACCTCCGTTTCCAGTCTTCCGCCATCGGCGCCCTGCAGGAG

TCGGTCGAGTCCTACCTCGTCTCCCTCTTCGAGGACACCAACCTGTGCGCCATCCACGCC

AAGCGTGTCACTATCCAGTCGGTATGTAACAAGGTCTCCGTTGGTGAACCAGCGGAGGGA

TCATTGCTGCCCCAGGCGCACCCAGAAACCCTTTGTGAACTTTTACTGTTGCCTCGGCTG

GCTGGGGGGCCCCTCCGGGTGTTGAGACGGCCCGCCGGCGGCCAACCTGACTCTTGTTTT

TACACCGAAACTCTAAATGAATCAAAACTTTCAACAACGGATCTCTTGGTTCTGGCATCG

ATGAAGAACGCAGCGAAATGCGATAAGTAATGTGAATTGCAGAATTCAGTGAATCATCGA

ATCTTTGAACGCACATTGCGCCCTCTGGTATTCCGGAGGGCATGCCTGTTCGAGCGTCAT

TTCAACCCTCAAGCCTGGCTTGGTGATGGGGCACTGAAAAGCAGGCCCTGAAATTCAGTG

GCGAGCTCGCCAGGACCCCGAGCGCAGTAGACCCTCGCTAAGGCCCTGGCGGTGCCCTGC

TGAAAGAGAAGGAAGGTTAGTAAATATCATCATGCTCGCGCGGCCTCGCCAGCGCATTTT

CACCCCTCGCTTTGGATTTTCAGAGTGCGGGGCTTAGGGCTTATCTTGTCACCACCACCG

AATATATGCTGATATCTACACAGCCGCCGAGCTTGGTAAGGGTCGCACCTGAGCCCCACC

ATGGCGACTCACCCCCTGGGACACCCAGATAAGACGCGTCGATTGCTAACGTGTTTTTCT

CGCCTCTAGGTGCACCTTCAGACCGGCCAATGCGTAAGTTCCTGTCAC-AGCGGACCTTA

TCATCGCCACCTGTAGCACGTTTCCCAGGGTAACCAAATCGGTGCTGCTTTCTGGTGCGT

TCCAAGTCCACCGCCGCGATACGACCTCGAAGCATGCTGACCTCGTAGGCAAACCATCTC

TGGCGAGCACGGCCTCGACAGCAATGGCGTGTATGCACCTCCTATTCCTGCCCATCTTAG

CTTCCCTGACAATCGCATAGTTACAACGGCACTTCTGAGCTCCAGCTCGAGCGCATGAAC

GTCTACTTCAACGAGGTAAGTACATCATTCCGACCATCTCCAACCTCGCTAACGTGTTAT

CAGGCCTCCGGCAACAAGTATGTTCCTCGCGCCGTCCTCGTCGATCTCGAGCCCGGTACC

ATGGACGCCGTC

>Diaporthe_biconispora_CGMCC_3_17252

????????????????????????????????????????????????????????????

????????????????????????????????????????????????????????????

????????????????????????????????????????????????????????????

????????????????????????????????????????????????????????????

????????????????????????????????????????????????????????????

???????????????????????????????TCCGCGCCCTCCACCGGAGGTGTCAAGAA

GCCTCACCGCTACAAGCCTGGTACCGTCGCTCTGCGTGAGATCCGTCGTTACCAGAAGAG

CACCGAGCTGCTGATCCGCAAGCTCCCCTTCCAGCGTCTGGTATGCAGGTCCGTGAGATC

GCCCAGGACTTCAAGTCCGACCTCCGCTTCCAGTCTTCCGCCATCGGTGCCCTGCAGGAG

TCCGTCGAGTCTTACCTCGTCTCCCTCTTTGAGGACACCAACCTGTGCGCCATCCACGCC

AAGCGCGTCACCATCCAGTCGGTACGTNNNNNNNNNNNNNNNNNNNNNNNNNNNNNNNNN

NNNNNNCTGCCCCAGGCGCACCCAGAAACCCTTTGTGAACTTTTACTGTTGCCTCGGTAC

GCTGGGGGTCCCTTTGACAAAAGGAGCAGGCGCGCCGGCGGCCAAGTTAACTCTTGTTTT

TACACTGAAACTCTAAATGAATCAAAACTTTCAACAACGGATCTCTTGGTTCTGGCATCG

ATGAAGAACGCAGCGAAATGCGATAAGTAATGTGAATTGCAGAATTCAGTGAATCATCGA

ATCTTTGAACGCACATTGCGCCCTCTGGTATTCCGGAGGGCATGCCTGTTCGAGCGTCAT

TTCAACCCTCAAGCCTGGCTTGGTGTTGGGGCACTGGGAAGCAGGCCCTCAAATCTAGTG

GCGAGCTCGCCAGGACCCCGAGCGTAGTAGACCCTCGCTAAGGCCCTGGCGGTGCCCTGC

TGAAAGAGAAGGAAGGTTAGTAAACATCATCATGTTCGTGTGGCTTCGTCAGCGCATTTT

CACCCCTCGCTCTGGATTTTCAGGGTGCGGGGCTTAGAGCTTATCTCACCACCAACACCG

AAAACATGCTAACACCTCCTCAGCCGCCGAGCTTGGTAAGGGNNNNNNNNNNNNNNNNNN

NNNNNNNNNNNNNNNNNNNNNNNNNNNNNNNNNNNNNNNNNNNNNNNNNNNNNNNNNNNN

NNNNNNNNNNNNNNNNNNNNNNNNNNNNNNNNNNNNNNNNNNNNNNNNNNNNNNNNNNNN

NNNNNNNNNNNNNNNNNNNNNNNNNNNNNNNNNNNNNNNNNNNNNNNNNNNNNNNNNNNN

NNNNNNNNNNNNNNNNNNNNNNNACCTCGAAGCATACTGACCTCGTAGGCAAACCATCTC

TGGCGAGCACGGCCTCGACAGCAATGGCGTGTATGCACCTCCTATTCCTGTCCATTCTGT

CCTCCCTGACAATCATACAGTTACAACGGCACTTCCGAGCTCCAGCTCGAGCGCATGAAC

GTCTACTTCAACGAGGTAAGTACGTCG--CTGACCATCTACAGCTTTGCTAACGCGTTAT

CAGGCCTCCGGCAACAAGTATGTTCCTCGCGCCGTCCTCGTCGATCTCGAGCCCGGTACC

ATGGACGCCGTC

>Diaporthe_pseudobiguttulata_ICMP20657

????????????????????????????????????????????????????????????

????????????????????????????????????????????????????????????

????????????????????????????????????????????????????????????

????????????????????????????????????????????????????????????

????????????????????????????????????????????????????????????

???????????????????????????????TCCGCGCCCTCCACCGGAGGTGTCAAGAA

GCCTCACCGCTACAAGCCTGGTACCGTCGCTCTGCGTGAGATCCGTCGCTACCAGAAGAG

CACCGAGCTGCTGATCCGCAAGCTCCCCTTCCAGCGTCTGGTATGCAGGTTCGTGAGATC

GCCCAGGACTTCAAGTCCGACCTGCGCTTCCAGTCTTCCGCCATCGGTGCCCTGCAGGAG

TCCGTCGAGTCCTACCTCGTCTCCCTCTTTGAGGACACCAACCTGTGCGCCATCCACGCC

AAGCGTGTCACCATCCAGTCGGTACGTNNNNNNNNNNNNNNNNNNNNNNNNNNNNNNNNN

NNNNNNCTGCCTC-GGCGCACCCAGAAACCCTTTGTGAACTTTA-CTGTTGCCTCGGCAG

GCCGGGAGGCCCCCCGAGACGGGGAGCA-GCCCGCCGGCGGCCAACCTAACTCTTGTTTT

TACACTGTATCTCTAAATGAATCAAAACTTTCAACAACGGATCTCTTGGTTCTGGCATCG

ATGAAGAACGCAGCGAAATGCGATAAGTAATGTGAATTGCAGAATTCAGTGAATCATCGA

ATCTTTGAACGCACATTGCGCCCTCTGGTATTCCGGAGGGCATGCCTGTTCGAGCGTCAT

TTCAACCCTCAAGCCTGGCTTGGTGTTGGGGCACTGAGAAGCAGGCCCTGAAATCTAGTG

GCGAGCTCGCCAGGACCCCGAGCGTAGTAGA-TCTCGTTAAGGCCCTGGCGGTGCCCTGC

TGAAAGAGAAGGAAGGTTAGTAAACATTATCGCATCTGCGCGGCTTCGTCAAGGCATTTT

CACCCCTCGCTCTGGATTTTCAGGGTGCGGGGCTTACGGCTTATCTCGC-GCTACCACCG

GAACTATGCTGATTTCTACACAGCCGCCGAGCTTGGTAAGGGNNNNNNNNNNNNNNNNNN

NNNNNNNNNNNNNNNNNNNNNNNNNNNNNNNNNNNNNNNNNNNNNNNNNNNNNNNNNNNN

NNNNNNNNNNNNNNNNNNNNNNNNNNNNNNNNNNNNNNNNNNNNNNNNNNNNNNNNNNNN

NNNNNNNNNNNNNNNNNNNNNNNNNNNNNNNNNNNNNNNNNNNNNNNNNNNNNNNNNNNN

NNNNNNNNNNNNNNNNNNNNNNNAGCTCGCAGCATACTGACATCTCAGGCAAACCATCTC

TGGCGAGCACGGCCTCGACAGCAATGGCGTGTATGCACCTCCTATTCCTGCCCATCTCGT

CCTCCCTGACAATTGCACAGTTACAACGGCACTTCCGAGCTCCAGCTCGAGCGCATGAAC

GTCTACTTCAACGAGGTAAGTACGTCGTTCTATCCATCTACGGTCTTGCTAACGCGTTAT

CAGGCCTCCGGCAACAAGTATGTGCCTCGCGCTGTCCTCGTCGATCTCGAGCCCGGTACC

ATGGACGCCGTC

>Diaporthe_eres_CGMCC_3_17081

????????????????????????????????????????????????????????????

????????????????????????????????????????????????????????????

????????????????????????????????????????????????????????????

????????????????????????????????????????????????????????????

????????????????????????????????????????????????????????????

????????????????????????????????????????????????????????????

????????????????????????????????????????????????????????????

????????????????????????????????????????????????????????????

????????????????????????????????????????????????????????????

????????????????????????????????????????????????????????????

???????????????????????????NNNNNNNNNNNNNNNNNNNNNNNNNNNNNNNNN

NNNNNNNNNNNNNNNNNNNNNNNNNNNACCCTTTGTGAACTTTTACTGTTGCCTCGGCTA

GCTGGGGGGCCCCTCCGGGTGTTGAGACAGCCCGCCGGCGGCCAACCTAACTCTTGTTTT

TACACTGAAACTCTAAATGAATCAAAACTTTCAACAACGGATCTCTTGGTTCTGGCATCG

ATGAAGAACGCAGCGAAATGCGATAAGTAATGTGAATTGCAGAATTCAGTGAATCATCGA

ATCTTTGAACGCACATTGCGCCCTCTGGTATTCCGGAGGGCATGCCTGTTCGAGCGTCAT

TTCAACCCTCAAGCCTGGCTTGGTGATGGGGCACTGAAAAGCAGGCCCTGAAATTCAGTG

GCGAGCTCGCCAGGACCCCGAGCGCAGTAGACCCTCGCTAAGGCCCTGGCGGTGCCCTGC

TGAAAGAGAAGGAAGGTTAGTAAATATCATCATGCTCGCGCGGCCTCGCCAGCGCATTTT

CACCCCTCGCTTTGGATTTTCAGAGTGCGGGGCTTAGGGCTTATCTTGTCACCACCACCG

AATATATGCTGATATCTACACAGCCGCCGAGCTTGGTAAGGGNNNNNNNNNNNNNNNNNN

NNNNNNNNNNNNNNNNNNNNNNNNNNNNNNNNNNNNNNNNNNNNNNNNNNNNNNNNNNNN

NNNNNNNNNNNNNNNNNNNNNNNNNNNNNNNNNNNNNNNNNNNNNNNNNNNNNNNNNNNN

NNNNNNNNNNNNNNNNNNNNNNNNNNNNNNNNNNNNNNNNGGTGCTGCTTTCTGGTGCGT

TCCAAGTCCACCACCGCGATACGACCTCGAAGCATGCTGACCTCGTAGGCAAACCATCTC

TGGCGAGCACGGCCTCGACAGCAATGGCGTGTATGCACCTCCTATTCCTGCCCATCTTGG

CTTCCCTGACAATCGCATAGTTACAACGGCACTTCTGAGCTCCAGCTCGAGCGCATGAAC

GTCTACTTCAACGAGGTAAGTACATCATTCCGACCATCTCCAACCTCGCTAACGTGTTAT

CAGGCCTCCGGCAACAAGTATGTTCCTCGCGCCGTCCTCGTCGATCTCGAGCCCGGTACC

ATGGACGCCGTC

>Diaporthe_bohemiae_CBS_143347

NNNNNNNNNNNNNNNNNNNNNNNNNNNNCCTTGCTGTTGCGCATGCTAACGGACCGTTTT

CGGCCTGCAGGATAAGGATGGCGATGGTTAGTGTGGCCGCACCCACCGAGCACGCGTCAT

GCTCGATCCGCCGCGACGGCCGGCGCGCGAGCAGGCGTCAGCACTACCAGGGATTGCTAA

GGCGTAGGACAAATCACCACGAAGGAGCTCGGCACCGTCATGCGATCCCTGGGCCAGAAC

CCATCCGAGTCTGAGCTGCAAGATATGATTAACGAGGTCGATGCCGACAACAATGGCACC

ATTGACTTTGGTACGTCCAGACGCTCGCCTGTCCGCGCCCTCCACCGGAGGTGTCAAGAA

GCCTCACCGCTACAAGCCTGGTACTGTCGCTCTGCGTGAGATCCGTCGTTACCAGAAGAG

CACCGAGCTGCTCATCCGCAAGCTGCCCTTCCAGCGTCTGGTACGCAGGTTCGTGAGATC

GCCCAGGACTTCAAGTCCGACCTCCGCTTCCAGTCTTCCGCCATCGGTGCCCTGCAGGAG

TCCGTCGAGTCTTACCTCGTCTCCCTCTTCGAGGACACCAACCTGTGCGCCATCCACGCC

AAGCGTGTCACCATCCAGTCGGTATGTAACAAGGTCTCCGTTGGTGAACCAGCGGAGGGA

TCATTGCTGCCCCCGGCGCACCCAGAAACCCTTTGTGAACTCCTACTGTTGCCTCGGCAG

GCCGGGGGGCCCCTCGAGACGAGGAGCAGGCCGGCCGGCGGCCTAGTTAACTCTTGTTTT

TACACTGAAACTCTAAATGAATCAAAACTTTCAACAACGGATCTCTTGGTTCTGGCATCG

ATGAAGAACGCAGCGAAATGCGATAAGTAATGTGAATTGCAGAATTCAGTGAATCATCGA

ATCTTTGAACGCACATTGCGCCCTCTGGTATTCCGGAGGGCATGCCTGTTCGAGCGTCAT

TTCAACCCTCAAGCCTGGCTTGGTGATGGGGCACTGAAGGGCAGGCCCTGAAATTCAGTG

GCGAGCTCGCCAGGACCCCGAGCGCAGTAGACCCTCGCTAAGGCCCTGGCGGTGCCCTGC

TGAAAGAGAAGGAAGGTTAGTAAACATCATCGCTCCTGCGCAGCTCCGTGAGCGCATTTT

CACCCCACGTCCTGGATTTTCAGGGTGCGGGGCTTAGAGCTTATCTCACTACCACCACCG

GAAAGATGCTAACAACTATATAGCCGCCGAGCTTGGCAAGGGNNNNNNNNNNNNNNNNNN

NNNNNNNNNNNNNNNNNNNNNNNNNNNNNNNNNNNNNNNNNNNNNNNNNNNNNNNNNNNN

NNNNNNNNNNNNNNNNNNNNNNNNNNNNNNNNNNNNNNNNNNNNNNNNNNNNNNNNNNNN

NNNNNNNNNNNNNNNNNNNNNNNNNNNNNNNAACCAAATCGGTGCTGCTTTCTGGTGCGT

CCCAAACTCACCACCGCGACACGGCCTCGCGGCATACTGACCTCGTAGGCAAACCATCTC

TGGCGAGCACGGCCTCGACAGCAATGGCGTGTATGCACCTCCTATTCCTGCACATCTCGC

CCTCCCTGACAGTTGCACAGTTACAACGGCACTTCCGAGCTCCAGCTCGAGCGCATGAAC

GTCTACTTCAACGAGGTATGTATATTGTTCCGACCGT-TTCAACCTCGCTGACGCGTTAT

CAGGCCTCCGGCAACAAGTATGTTCCTCGTGCGGTCCTCGTCGATCTCGAGCCCGGTACC

ATGGACGCCGTC

>Diaporthe_bombacis_SDBR_CMU468

CTTTGTAAGTACCATCTGACACGCCCGCCCTTGCTGTTGCGCATGCTAACGGACCGTTTT

CGGCTTGTAGGATAAGGATGGCGATGGTTAGTGCGGCCGCTCTCACACAGCACGCGTCAT

GCTCGATCCTCCGCGACGGCCTGCGCGCAACCAAGCGTGATCACTATTGCGAGTTGCTGA

GGTGCAGGACAAATCACCACCAAGGAGCTCGGCACCGTCATGCGATCCCTGGGCCAGAAC

CCTTCCGAGTCTGAGCTGCAAGACATGATTAACGAGGTCGACGCCGACAACAACGGCACC

ATTGACTTTGGTACGTCCAGATATACGCCCATCCGCGCCCTCCACCGGAGGTGTCAAGAA

GCCTCACCGCTACAAGCCTGGTACCGTCGCTCTGCGTGAGATCCGTCGCTACCAGAAGAG

CACTGAGCTGCTGATCCGCAAGCTCCCCTTCCAGCGTCTGGTATGCAGGTCCGTGAGATC

GCCCAGGACTTCAAGTCCGACCTTCGCTTCCAGTCCTCCGCCATCGGTGCCCTGCAGGAG

TCCGTCGAGTCTTACCTCGTCTCCCTCTTCGAGGACACCAACCTGTGCGCCATCCACGCC

AAGCGTGTCACCATCCAGTCGGTACGTAACAAGGTCTCCGTTGGTGAACCAGCGGAGGGA

TCATTGCTGCCCCAGGCGCACCCAGAAACCCTTTGTGAACTTTT-TTGTTGCCTCGGCAT

GCTGGTAGGCCCCTC-CGGTGAGGAGAAGGCACGCCGGCGGCCAAGTTAACTCTTGTTTT

TACACTGAAACTCTAAATGAATCAAAACTTTCAACAACGGATCTCTTGGTTCTGGCATCG

ATGAAGAACGCAGCGAAATGCGATAAGTAATGTGAATTGCAGAATTCAGTGAATCATCGA

ATCTTTGAACGCACATTGCGCCCTCTGGTATTCCGGAGGGCATGCCTGTTCGAGCGTCAT

TTCAACCCTCAAGCATTGCTTGGTGTTGGGGCACTGCGAAGCAGGCCCTGAAATCTAGTG

GCGAGCTCGCCAGGACCCCGAGCGCAGTAGACCCTCGCTAAGGCCCTGGCGGTGCCCTGC

TGAAAGAGAAGGAAGGTTAGTAAACATCATCATGTTGGTACAGCTTCGTCAGCGCATTTT

CACCCCTCGCTCTGGATTTTCAGGGTGCGGGGCTTAGAGCTTATCTAGTTATTACCACCG

GAAACATGCTGATATCTACATAGCCGCCGAGCTTGGTAAGGGNNNNNNNNNNNNNNNNNN

NNNNNNNNNNNNNNNNNNNNNNNNNNNNNNNNNNNNNNNNNNNNNNNNNNNNNNNNNNNN

NNNNNNNNNNNNNNNNNNNNNNNNNNNNNNNNNNNNNNNNNNNNNNNNNNNNNNNNNNNN

NNNNNNNNNNNNNNNNNNNNNNNNNNNNNNNNNNNNNNNCGGTGCTGCTTTCTGGTGCG-

------TCCACCACCACGATACGACCTCGAAACATACTGACCTCGCAGGCAAACCATCTC

TGGCGAGCACGGCCTCGACAGCAATGGCGTGTATGCACCTCCTATTTCTGCCTTTCTCGT

CTGCCCTGACAATCACACAGTTACAACGGCACTTCCGAGCTCCAGCTCGAGCGCATGAAT

GTCTACTTCAACGAGGTAAGTACGTCG--TTGGCCATCTGCAGCTTTGCTAACGCGTTAT

CAGGCCTCCGGCAACAAGTATGTCCCTCGCGCCGTCCTCGTCGATCTCGAGCCCGGTACC

ATGGACGCCGTC

>Diaporthe_bounty_BRIP_59361a

????????????????????????????????????????????????????????????

????????????????????????????????????????????????????????????

????????????????????????????????????????????????????????????

????????????????????????????????????????????????????????????

????????????????????????????????????????????????????????????

????????????????????????????????????????????????????????????

????????????????????????????????????????????????????????????

????????????????????????????????????????????????????????????

????????????????????????????????????????????????????????????

????????????????????????????????????????????????????????????

???????????????????????????AACAAGGTCTCCGTTGGTGAACCAGCGGAGGGA

TCATTGCTGCCCCAGGCGCACCCAGAAACCCTTTGTGAACTTTTACTGTTGCCTCGGCAT

GCTGGTAGGCCCCTC-CGGTGAGGAGAAGGCACGCCGGCGGCCAAGTTAACTCTTGTTTT

TACACTGAAACTCTAAATGAATCAAAACTTTCAACAACGGATCTCTTGGTTCTGGCATCG

ATGAAGAACGCAGCGAAATGCGATAAGTAATGTGAATTGCAGAATTCAGTGAATCATCGA

ATCTTTGAACGCACATTGCGCCCTCTGGTATTCCGGAGGGCATGCCTGTTCGAGCGTCAT

TTCAACCCTCAAGCACTGCTTGGTGTTGGGGCACTGCGAAGCAGGCCCTGAAATCTAGTG

GCGAGCTCGCCAGGACCCCGAGCGCAGTAGACCCTCGCTAAGGCCCTGGCGGTGCCCTGC

NNNNNNNNNNNNNNNNNNNNNNNNNNNNATCATGTTGGTATGGCTTCGTCAGCGCATTTT

CACCCCTCGCTCTGGATTTTCAGGGTGCGGGGCTTAGAGCTTATCTACCA-CCACTTCAG

GAAACATGCTGATATCTATACAGCCGCCGAGCTTGGCAAGGGTCGCACCTGAGCCCCACC

ATCGCGACCCACCCCCTGGGACACCCAGATAAAACGCGTCGATTGCTAATGTGTTTTTCT

CGACCACAGGTTCACCTTCAGACCGGCCAATGCGTAAGTTGCTGTCACGACGGA-CCTTA

TCATCGCCACCCGTAGCACGTTTCCCAGGGTAACCAAATCGGTGCTGCTTTCTGGTGC--

-----GTCCACCACCGCGATACGACCTCGAAACATACTGACCTCGCAGGCAAACCATCTC

TGGCGAGCACGGCCTCGACAGCAATGGCGTGTATGCACCTCCTATTCCTACCTCACTCGT

CTGCCCTGACAATCACACAGTTACAACGGCACTTCCGAGCTCCAGCTCGAGCGCATGAAC

GTCTACTTCAACGAGGTAAGTACGTCG--TTGGCCATCTGCAGCCCTGCTAACGCGTTAT

CAGGCCTCCGGCAACAAGTATGTCCCTCGCGCCGTCCTCGTCGATCTCGAGCCCGGTACC

ATGGACGCCGNN

>Diaporthe_brasiliensis_CBS_133183

CTTCGTAAGTCACCTTCGCCATGCGCGCACTTGCTGTTCCGCATGCTAACGGACCGTTTT

CGGCCTGCAGGATAAGGATGGCGATGGTTAGTGTAGCCGCTCCCA-CTAGCACGCGTCAC

GCTCGATCCGCCGCGACGGCCTGCGCGTTAGCGAGCGTCATCACTATCAGGGGTTGCTAA

GGTGTAGGACAAATCACCACGAAGGAGCTCGGCACGGTCATGCGGTCCCTGGGTCAAAAC

CCGTCCGAGTCTGAGCTGCAAGATATGATTAACGAGGTTGACGCCGACAACAATGGCACC

ATCGACTTTGGTACGTCCAGATGCTCTCCCATCCGCACCCTCTACCGGAGGTGTCAAGAA

GCCTCACCGCTACAAGCCTGGTACCGTCGCTCTGCGTGAGATCCGTCGTTACCAGAAGAG

CACCGAGCTGCTGATCCGCAAGCTCCCCTTCCAGCGTCTGGTATGCAGGTTCGTGAGATC

GCCCAGGACTTCAAGTCCGACCTGCGTTTCCAGTCTTCCGCCATCGGTGCCCTGCAGGAG

TCCGTCGAGTCCTACCTCGTCTCCCTGTTCGAGGACACCAACCTGTGCGCCATCCACGCC

AAGCGTGTCACCATCCAGTCGGTACGTAACAAGGTCTCCGTTGGTGAACCAGCGGAGGGA

TCATTGCTGCCCTCGGCGCACCCAGAAACCCTTTGTGTACTTCTACTGTTGCCTCGGACA

GCCGGGGGGCCCCCCGAGACGGGGAGCAGGCACGCCGGCGGCCAAGTTAACTCTTGTTTT

TACACTGAAACTCTAAATGAATCAAAACTTTCAACAACGGATCTCTTGGTTCTGGCATCG

ATGAAGAACGCAGCGAAATGCGATAAGTAATGTGAATTGCAGAATTCAGTGAATCATCGA

ATCTTTGAACGCACATTGCGCCCTCTGGTATTCCGGAGGGCATGCCTGTTCGAGCGTCAT

TTCAACCCTCAAGCCTGGCTTGGTGTTGGGGCGCCGAAGGGCGGGCCCTGAAATTCAGTG

GCGAGCTCGCCAGGACCCCGAGCGCAGTAGACCCTCGCTAAGGTTCCTGGCGGTATCTGC

TTGAANNNNNGGAAGGTCAGTAAACTCCATCATGTTTGCGCGACTTCGTCAGGGCATTTT

CACCCCTCGCTCTGGATTTTCAGGGTGCGGGGCTTACAGCTTATCTCACCACTAAAACAG

AAAAGATGCTGACAACTGTGTAGCCGCTGAGCTTGGTAAGGNTCGCACCTCAGCCCCACC

ATCGCGACCCGCCCCCTGGGACACCCAGATAAGACGCGTCGATTGCTAACATGTTTTCCT

CATCCACAGGTTCATCTTCAGACCGGCCAATGCGTAAGTTGCTGTCACCTCCGGACTTTA

T---CGCCACCTGTAGCACGTTTCCCAGGGTAACCAAATCGGTGCTGCTTTCTGGTGCGT

CCCAAGTCTATCGCCGCGACACGACCTCGAAGCATACTGACCTCGCAGGCAAACCATCTC

TGGCGAGCACGGCCTCGACAGCAATGGCGTGTATGTACCTCCTATTCCTGCCCACCTCGT

TCTCCCTGACAATTGCACAGTTACAACGGCACTTCCGAGCTCCAGCTCGAGCGCATGAAC

GTCTACTTCAACGAGGTAAGTACGTCG-TTCGACCATCTACAGCCTTGCTAACGCGTTAT

CAGGCTTCCGGCAACAAGTATGTGCCTCGCGCCGTCCTCGTCGATCTCGAGCCCGGTACC

ATGGACGCCGTC

>Diaporthe_brideliae_CBS_148911

NNNNNNNNNNNNNNNNNNNNNTCCCCACCCTTGCTGTCGCGCATGCTAACGGACCGTTTT

CGGCTCGCAGGATAAGGATGGCGATGGTTAGTGCAGCCACTTCCAACTAGCACGCGTCAC

TCTTGATCCGCTACGACGGTCTACGCGCAACCGACCGTCATCACCATCACGAGTTGCTAA

GGTGTAGGACAAATCACCACCAAGGAGCTCGGCACGGTCATGCGGTCCCTGGGTCAAAAC

CCCTCCGAGTCCGAGCTGCAGGATATGATCAATGAGGTCGACGCCGACAACAATGGCACC

ATTGACTTTGGTAAGTCTAGATGCTCGCATATCCGCGCCCTCCACCGGAGGTGTCAAGAA

GCCTCACCGCTACAAGCCTGGTACCGTCGCTCTGCGTGAGATCCGTCGTTACCAGAAGAG

CACCGAGCTGCTGATCCGCAAGCTCCCCTTCCAGCGTCTGGTATGCAGGTTCGTGAGATC

GCCCAGGACTTCAAGTCCGACCTGCGCTTCCAGTCTTCCGCCATCGGTGCCCTGCAGGAG

TCCGTCGAGTCTTACCTCGTCTCCCTCTTTGAGGACACCAACCTGTGCGCCATCCACGCC

AAGCGTGTCACCATCCAGTCGGTACGTAACAAGGTCTCCGTTGGTGAACCAGCGGAGGGA

TCATTGCTGCTTC-GGCGCACCCAGAAACCCTTTGTGAACTTTA-CTGTTGCCTCGGCAG

GCCGGGAGGCCCCCCGAGACGGGGAGCA-GCCCGCCGGCGGCCAACCAAACTCTTGTTTC

TACAGTGAATCTCTAAATGAATCAAAACTTTCAACAACGGATCTCTTGGTTCTGGCATCG

ATGAAGAACGCAGCGAAATGCGATAAGTAATGTGAATTGCAGAATTCAGTGAATCATCGA

ATCTTTGAACGCACATTGCGCCCTCKGGTATTCCGGAGGGCATGCCTGTTCGAGCGTCAT

TTCAACCCTCAAGCCTGGCTTGGTGKTGGGGCACTGAAGGGCAGGCCCTGAAATCKAGTG

GCGAGCTCGCTAGGACCCCGAGCGTAGTAGR-TCTCGTTAAGGCCCTGGCGGTGCCCTGC

KGARAGAGAAGGAAGGTCAGTAAACATCATCATGCTCTCGTGGCTTCATCGGCGCATTTT

GACCCCTCCCTCTGGATTTTCAGGGTGCGGGGCTTAGAGCTTATCTCACCAACACGCCTG

GAAACATGCTGATATCTACACAGCCGCTGAGCTTGGTAAGGGNNNNNNNNNNNNNNNNNN

NNNNNNNNNNNNNNNNNNNNNNNNNNNNNNNNNNNNNNNNNNNNNNNNNNNNNNNNNNNN

NNNNNNNNNNNNNNNNNNNNNNNNNNNNNNNNNNNNNNNNNNNNNNNNNNNNNNNNNNNN

NNNNNNNNNNNNNNNNNNNNNNNNNNNNNNNNNNNNNNNNGGTGCTGCTTTCTGGTGCGT

CGCAAGCATACCATCGCGAAACTAGCTCGCAGCATACTGACATCTTAGGCAAACCATCTC

TGGCGAGCACGGCCTCGACAGCAATGGCGTGTATGTACCTCCTATTCCTGCCCATCTCGT

CCCCCCTGACAATTGCATAGTTACAACGGCACTTCCGAGCTTCAGCTCGAGCGCATGAAC

GTCTACTTCAACGAGGTTAGTACGTCGCTCTGACCATCTACGGTCTTGCTAACGCGTTAT

CAGGCCTCCGGCAACAAGTATGTGCCTCGCGCCGTCCTCGTCGATCTCGAGCCTGGTACC

ATGGATGCCGTC

>Diaporthe_brumptoniae_BRIP_59403a

????????????????????????????????????????????????????????????

????????????????????????????????????????????????????????????

????????????????????????????????????????????????????????????

????????????????????????????????????????????????????????????

????????????????????????????????????????????????????????????

????????????????????????????????????????????????????????????

????????????????????????????????????????????????????????????

????????????????????????????????????????????????????????????

????????????????????????????????????????????????????????????

????????????????????????????????????????????????????????????

???????????????????????????AACAAGGTCTCCGTTGGTGAACCAGCGGAGGGA

TCATTGCTGCTTC-GGCGCACCCAGAAACCCTTTGTGAACTT-TACTGTTGCCTCGGCAG

GCCGGGAGGCCCCCTGAAACAGGGAGCA-GCCCGCCGGCGGCCAAACAAACTCTTGTTTC

TACGGTGAATCTCTAAATGAATCAAAACTTTCAACAACGGATCTCTTGGTTCTGGCATCG

ATGAAGAACGCAGCGAAATGCGATAAGTAATGTGAATTGCAGAATTCAGTGAATCATCGA

ATCTTTGAACGCACATTGCGCCCTCTGGTATTCCGGAGGGCATGCCTGTTCGAGCGTCAT

TTCAACCCTCAAGCCTGGCTTGGTGATGGGGCACTGAAAGGCAGGCCCTGAAATCTAGTG

GCGAGCTCGCCAGGACCCCGAGCGTAGTAGA-TCTCGCTAAGGCCCTGGCGGTGCCCTGC

NNNNNNNNNNNNNNNNNNNNNNNNNNNCGTTGTGTCTGCGAGGCTTCGTCAAGGCATTTT

CACCCCTCCATCCGGATTTTCAGGGTGCGGGGCTTACCGCTTATCTCTGTGAC-GCACCG

GAAGCATGCTGATTTCTGTACAGCCGCCGAGCTGGGCAAGGGTCGCACCTCAGCCCCACC

ATCGCGGCCCACCCCCTGCAACACCAAGATAACACGCGTCGATTGCTAACATGTTTTTCT

CGCCCACAGGTTCACCTTCAGACCGGCCAATGCGTAAGTTGTTGTCACCGCCAGACCTTA

T---CGCCACCCGTAGCACGTTTCCCAGGGTAACCAAATCGGTGCTGCTTTCTGGTGCGT

CCCGAGCCTACCACCGCGAGGCGAGCTCGAAACAGACTGACCTCGTAGGCAAACCATCTC

TGGCGAGCACGGCCTCGACAGCAATGGCGTGTATGGACCTCCTATTCCTGACTACCTCGT

CCTCCCTGATGATCGCACAGTTACAACGGCACTTCCGAGCTCCAGCTCGAGCGCATGAAC

GTCTACTTCAACGAGGTAAGTACGTCGCCTTGA-CCGTCTTGCCCTTGCTAACGCGTTTT

CAGGCCTCCGGCAACAAGTATGTGCCCCGCGCCGTCCTCGTCGATCTCGAGCCCGGTACC

ATGGACGCCGTC

>Diaporthe_butterlyi_BRIP_59194a

????????????????????????????????????????????????????????????

????????????????????????????????????????????????????????????

????????????????????????????????????????????????????????????

????????????????????????????????????????????????????????????

????????????????????????????????????????????????????????????

????????????????????????????????????????????????????????????

????????????????????????????????????????????????????????????

????????????????????????????????????????????????????????????

????????????????????????????????????????????????????????????

????????????????????????????????????????????????????????????

???????????????????????????AACAAGGTCTCCGTTGGTGAACCAGCGGAGGGA

TCATTGCTGCTTC-GGCGCACCCAGAAACCCTTTGTGAACTT-CACTGTTGCCTCGGCAG

GCCGGGAGGCCCCCTGAAACAGGGAGCA-GCCCGCCGGTGGCCAACTAAACTC-TGTTTC

TATAGTGAATCTCTAAATGAATCAAAACTTTCAACAACGGATCTCTTGGTTCTGGCATCG

ATGAAGAACGCAGCGAAATGCGATAAGTAATGTGAATTGCAGAATTCAGTGAATCATCGA

ATCTTTGAACGCACATTGCGCCCTCTGGTATTCCGGAGGGCATGCCTGTTCGAGCGTCAT

TTCAACCCTCAAGCCTGGCTTGGTGATGGGGCACTGAAGGGCAGGCCCTGAAATCTAGTG

GCGAGCTCGCCAGGACCCCGAGCGTAGTAGA-TCTCGCTAAGGCCCTGGCGGTGCCCTGC

NNNNNNGGAAGGAAGGTCAGTAAATATCATTGTGCCTGCGCGGCTTCATC-AACCATTTT

CACCCCTCCCTCTGGGTTTTCAGGGTGCGGGGCTTACCGCTTATCTCACCGCCAACACCG

AATAGAAGCTGATCTCCACACAGCCGCCGAGCTGGGCAAGGGTCGCACCTCAGCCCCACC

ATCGCGACCCACCCCCTGCGACACCCAGATAAGACGCGTCGACTGCTAACATGTTTTCCT

CTCTTACAGGTTCACCTTCAGACCGGCCAATGCGTAAGTTGCTGTCACGCCGGGACCTTA

T---CGCCACCCGTAGCACGTTTCCCAGGGTAACCAAATCGGTGCTGCTTTCTGGTGCGT

CAAGCCGCCACGACCGCGAGACGAGCTCGAAACATACTGACCTCGTAGGCAAACCATCTC

TGGCGAGCACGGTCTCGACAGCAATGGCGTGTATGCACCTCCTATTCCTGCCCATCTCGT

TCTCCCTGATGTTTGCACAGTTACAACGGCACTTCTGAGCTCCAGCTCGAGCGCATGAAC

GTCTACTTCAACGAGGTAAGCACGTCTTTTTGAGCGTTCTCGACCTGACTGACGCGCTTT

CAGGCCTCCGGCAACAAGTATGTGCCTCGCGCCGTCCTCGTCGATCTCGAGCCCGGTACC

ATGGACGCCGTC

>Diaporthe_caatingaensis_CBS_141542

CTTCGTAAGTCATCTTCGCCATGCGCGCCCTTGCTGTTCCGCATGCTAACGGACCGTTTT

CGGCCTGCAGGATAAGGATGGCGATGGTTAGTGTAGCCGCTCCCA-CTAGCACGCGTTAC

GCTCGATCCGCCGCGACGGCCTGCGCGTGAGCGAGCGTCATCACTACCAGGAGTTGCTAA

GGTGTAGGACAAATCACCACGAAGGAGCTCGGCACGGTCATGCGGTCCCTGGGTCAAAAC

CCGTCCGAGTCTGAGCTGCAAGATATGATTAACGAGGTTGACGCCGACAACAATGGCACC

ATCGACTTTGGTACGT-CAGATGCTCTCATATCCGCACCCTCTACCGGAGGTGTCAAGAA

GCCTCACCGCTACAAGCCTGGTACCGTCGCTCTGCGTGAGATCCGTCGTTACCAGAAGAG

CACCGAGCTGCTGATCCGCAAGCTCCCCTTCCAGCGTCTGGTATGCAGGTTCGTGAGATC

GCCCAGGACTTCAAGTCCGACCTGCGTTTCCAGTCTTCCGCCATCGGTGCCCTGCAGGAG

TCCGTCGAGTCCTACCTCGTCTCCCTGTTCGAGGACACCAACCTGTGCGCCATCCACGCC

AAGCGTGTCACCATCCAGTCGGTACGTAACAAGGTCTCCGTTGGTGAACCAGCGGAGGGA

TCATTGCTGCCTC-GGCGCACCCAGAAACCCTTTGTGAACTTTTACTGTTGCCTCGGATA

GCTGGGGGGCCCCCCGAGACGGGGAGCAGGCACGCCGGCGGCCAAGTTAACTCTTGTTTT

TACACTGAAACTCTAAATGAATCAAAACTTTCAACAACGGATCTCTTGGTTCTGGCATCG

ATGAAGAACGCAGCGAAATGCGATAAGTAATGTGAATTGCAGAATTCAGTGAATCATCGA

ATCTTTGAACGCACATTGCGCCCTCTGGTATTCCGGAGGGCATGCCTGTTCGAGCGTCAT

TTCAACCCTCAAGCCTGGCTTGGTGTTGGGGCGCCGAAGGGCGGGCCCTGAAATTCAGTG

GCGAGCTCGCCAGGACCCCGAGCGCAGTAGACCCTCGCTAAGGTTCCTGGCGGTATCTGC

TGAAANNNNNGGAAGGTCAGTAAACATCATCATGTTTGCGCGGCTTCGTCAGGGCATTTT

CACCCCTCGCTCTGGATTTTCAGGGTGCGGGGCTTACAGCTTATCTCACCACTAAAACAG

AGAAGATACTGACAACTGTGTAGCCGCTGAGCTTGGTAAGGNTCGCACCTCAGCCCCACC

ATCGCGACCCGCCCCCTGGGACACCCAGATGAGACGCGTCGATTGCTAACATGTTTTTCT

CGCCCACAGGTTCACCTTCAGACCGGCCAATGCGTAAGTTGCTGTCACCTCCGGACCTTA

T---CGCCACCTGTAGCACGTTTCCCAGGGTAACCAAATCGGTGCTGCTTTCTGGTGCGT

CACAAGTCTATCGCCGCGACACGACCTCGAAGCATACTGACCTCGCAGGCAAACCATCTC

TGGCGAGCACGGCCTCGACAGCAATGGCGTGTATGTACCTCCTATTCCTGCCCACCTCGT

TCTCCCTGACAATTGCACAGTTACAACGGCACTTCCGAGCTCCAGCTCGAGCGCATGAAC

GTCTACTTCAACGAGGTAAGTACGTCG-TTCGACCATCTACAGCCTTGCTAACGCGTTAT

CAGGCTTCCGGCAACAAGTATGTGCCTCGCGCCGTCCTCGTCGATCTCGAGCCCGGTACC

ATGGACGCCGTC

>Diaporthe_camelliae_sinensis_SAUCC194_92

NNNNNNNNNTCTCTGGCGA-ATGCCGGGCCATGCTGTCGCGCGTGCTAACGGACCGTTTT

CGGCTTGCAGGATAAGGATGGCGATGGTCAGTGCGGTCACTCACACCCAACACGCGTAAA

GCCCGGTCCGACGCGACGGCCTGCGCGCAAACGGGCGTTACCGCTATCACGAATTGCTGA

GGTGTAGGACAAATCACCACCAAGGAGCTCGGCACGGTCATGCGGTCCCTGGGTCAGAAC

CCGTCCGAGTCTGAGCTGCAAGACATGATCAACGAGGTCGACGCCGACAACAATGGCACC

ATTGACTTTGGTACGTTCAGATGCTCGCCCATCCGCGCCCTCCACCGGAGGTGTCAAGAA

GCCTCACCGCTACAAGCCTGGTACCGTCGCTCTGCGTGAGATCCGTCGCTACCAGAAGAG

CACCGAGCTGCTGATCCGCAAGCTCCCCTTCCAGCGTCTGGTATGCAGGTTCGTGAGATC

GCCCAGGACTTCAAGTCCGACCTCCGCTTCCAGTCCTCCGCCATCGGTGCCCTGCAGGAG

TCCGTCGAGTCTTACCTCGTCTCCCTGTTCGAGGACACCAACCTGTGCGCCATCCACGCC

AAGCGTGTCACCATCCAGTCGGTACGTNNNNNNNTCTCCGTTGGTGAACCAGCGGAGGGA

TCATTGCTGCCCCTGGCGCACCCAGAAACCCTTTGTGAACTTTTATTGTTGCCTCGGCAG

GCCGTGGGGTCCCTTAGACAAAGGAGCA-GCCGGCCGGCGGCCAAGTTAACTC-TGTTTT

TAAACTGAAACTCTAAATGAATCAAAACTTTCAACAACGGATCTCTTGGTTCTGGCATCG

ATGAAGAACGCAGCGAAATGCGATAAGTAATGTGAATTGCAGAATTCAGTGAATCATCGA

ATCTTTGAACGCACATTGCGCCCTCTGGTATTCCGGAGGGCATGCCTGTTCGAGCGTCAT

TTCAACCCTCAAGCCTGGCTTGGTGTTGGGGCACTGGAGAGCAGGCCCTGAAATATAGTG

GCGAGCTCGCCAGGACTCCGAGCGCAGTAGACCCTCGCTAAGG-CCTGGCGGTGCCCTGC

TGAAANNNNNNNNNGGTTCTCGCAT--CATCATGCTC--GTGGCTTCATCGGCGCATTTT

GACCCCTCCCTCTGGATTTTCAGGGTGCGGGGCTTAGAGCTTATCTCACCAACACCACCA

GTACCATGCTGATATCTATACAGCCGCTGAGCTTNNNNNNNNNNNNNNNNNNNNNNNNNN

NNNNNNNNNNNNNNNNNNNNNNNNNNNNNNNNNNNNNNNNNNNNNNNNNNNNNNNNNNNN

NNNNNNNNNNNNNNNNNNNNNNNNNNNNNNNNNNNNNNNNNNNNNNNNNNNNNNNNNNNN

NNNNNNNNNNNNNNNNNNNNNNNNNNNNNNNNNNNNNNNNNNNNNNNNNNNNNNNNNNNN

NC-TCAGCCACCTCCGCGATACGACCTCGAAGCATACTGACCTCGCAGGCAAACCATCTC

TGGCGAGCACGGTCTCGACAGCAATGGCGTGTATGCACCTCCTATTTCTGTCTTTCTCGT

-TGCCCTGACAATCACACAGTTACAACGGCACTTCCGAGCTCCAGCTCGAGCGCATGAAC

GTCTACTTCAACGAGGTAAGTACGTTG--TTGACCACCCACAGCTTGGCTAACGCGTTAC

CAGGCCTCCGGCAACAAGTATGTGCCCCGCGCCGTCCTCGTCGATCTCGAGCCCGGTACC

ATGGACGCCGTC

>Diaporthe_camelliaeoleiferae_HNZZ027

NNNNNNNNNNNNNNNNNNNNNNNNNNGCCCTTGCTGTTGCGCATGCTAACGGACCGTTTT

CGGTTTGTAGGATAAGGATGGCGATGGTTAGTGCGGCCGCTCTCACACAGCACGCGTCAT

GCTCGATCCTCCGCGACGGCCTGCGCGCAACCAAGCGTTATCACTATTGCGAGTTGCTGA

GGTGCAGGACAAATCACCACCAAGGAGCTCGGCACCGTCATGCGATCCCTGGGCCAGAAC

CCTTCCGAGTCTGAGCTGCAAGATATGATTAACGAGGTCGACGCCGACAACAACGGCACC

ATTGACTTTGGTACGTCCAGATATACGCCCATCCGCGCCCTCCACCGGAGGTGTCAAGAA

GCCTCACCGCTACAAGCCTGGTACCGTCGCTCTGCGTGAGATCCGTCGCTACCAGAAGAG

CACTGAGCTGCTGATCCGCAAGCTCCCCTTCCAGCGTCTGGTATGCAGGTCCGTGAGATC

GCCCAGGACTTCAAGTCCGACCTCCGCTTCCAGTCCTCCGCCATCGGTGCCCTGCAGGAG

TCCGTCGAGTCTTACCTCGTCTCCCTCTTCGAGGACACCAACCTGTGCGCCATCCACGCC

AAGCGTGTCACCATCCAGTCGGTACGTNNNNNNNNNNNNNNNNNNNNNNNNNNNNNNNNN

NNNNNNNNNNNNNNNNNNNNNNNNNNNNCCCTTTGTGAACTTTT-TTGTTGCCTCGGCAT

GCTGGTAGGCCCCTC-CGGTGAGGAGACGGCACGCCGGCGGCCAAGTTAACTCTTGTTTT

TACACTGAAACTCTAAATGAATCAAAACTTTCAACAACGGATCTCTTGGTTCTGGCATCG

ATGAAGAACGCAGCGAAATGCGATAAGTAATGTGAATTGCAGAATTCAGTGAATCATCGA

ATCTTTGAACGCACATTGCGCCCTCTGGTATTCCGGAGGGCATGCCTGTTCGAGCGTCAT

TTCAACCCTCAAGCATTGCTTGGTGTTGGGGCACTGCCAAGCAGGCCCTGAAATCTAGTG

GCGAGCTCGCCAGGACCCCGAGCGCAGTAGACCCTCGCTAAGGCCCTGGCGGTGCCCTGC

TGAAANNNNNNNAAGGTTAGTAAACACCATCATGTTGGTACAGCTTCGTCAGCGCATTTT

CACCCCTCGCTCTGGATTTTCAGGGTGCGGGGCTTAGAGCTTATCTAGTTACTACCACCG

GAAACATGCTGATATCTACATAGCCGCCGAGCTTGGTAANNNNNNNNNNNNNNNNNNNNN

NNNNNNNNNNNNNNNNNNNNNNNNNNNNNNNNNNNNNNNNNNNNNNNNNNNNNNNNNNNN

NNNNNNNNNNNNNNNNNNNNNNNNNNNNNNNNNNNNNNNNNNNNNNNNNNNNNNNNNNNN

NNNNNNNNNNNNNNNNNNNNNNNNNNNNNNNNNNNNNNNNNNTGCTGCTTTCTGGTGC--

-----GTCCACCACCGCGACATGACCTCGAGCCATACTGACCTCGCAGGCAAACCATCTC

TGGCGAGCACGGCCTCGACAGCAATGGCGTGTATGCACCTCCTATTTCTGCCTTTCTCGT

CTGCCCTGACAATCACACAGTTACAACGGCACTTCCGAGCTCCAGCTCGAGCGCATGAAC

GTCTACTTCAATGAGGTAAGTACGTCA--TTGACCATCTGCAGCCTTGCTAACGCGTTAT

CAGGCCTCCGGCAACAAGTATGTCCCTCGCGCCGTCCTCGTCGATCTCGAGCCCGGTACT

ATGGACGCCGTN

>Diaporthe_cameroonensis_STMA_18290

CTTTGTAAGTCCTCCTCGCCCTGCCCGCCGTTGCTGTCGCGCATGCTAACGGACCGTTTT

CGGCTTGCAGGATAAGGATGGCGATGGTTAGTGCAACCACTGCCAACTAGCACGCGTCAC

GCTTGATCCGCTACGACGGTCTGCGCGCAAGCGACCGTCGTCCCCATCACGAGTTGCTAA

GGTGTAGGACAAATCACCACCAAGGAGCTCGGCACGGTCATGAGGTCCCTGGGTCAAAAC

CCCTCCGAGTCCGAGCTGCAGGACATGATCAACGAGGTCGACGCCGACAACAATGGCACC

ATTGACTTTGGTAAGTCCAGATGCTCGCCTATCCGCGCCCTCTACCGGAGGTGTCAAGAA

GCCTCACCGCTACAAGCCTGGTACCGTCGCTCTGCGTGAGATCCGTCGCTACCAGAAGAG

CACCGAGCTGCTGATCCGCAAGCTCCCCTTCCAGCGTCTGGTATGCAGGTTCGTGAGATC

GCCCAGGACTTCAAGTCCGACCTGCGCTTCCAGTCTTCCGCCATTGGTGCCCTGCAGGAG

TCCGTCGAGTCCTACCTCGTCTCCCTCTTCGAGGACACCAACCTGTGCGCCATCCACGCC

AAGCGTGTCACCATCCAGTCGGTACGTAACAAGGTCTCCGTTGGTGAACCAGCGGAGGGA

TCATTGCTGCCTC-GGCGCACCCAGAAACCCTTTGTGAACTTTA-TTGTTGCCTCGGGAG

GCCGGGAGGCCCCCCGAGACGGGGAGCA-GCCCGCCGGCGGCCAACCAAACTCTTGTTTC

T-TAGTGAATCTCTAAATGAATCAAAACTTTCAACAACGGATCTCTTGGTTCTGGCATCG

ATGAAGAACGCAGCGAAATGCGATAAGTAATGTGAATTGCAGAATTCAGTGAATCATCGA

ATCTTTGAACGCACATTGCGCCCTCTGGTATTCCGGAGGGCATGCCTGTTCGAGCGTCAT

TTCAACCCTCAAGCCTGGCTTGGTGTTGGGGCGCTGAGGAGCAGGCCCTGAAATCTAGTG

GCGAGCTCGCTAGGACCCCGAGCGTAGTAGA-TCTCGTTAAGGCCCTGGCGGCGCCCTGC

TGAAAGAGAAGGAAGGTTAGTAAACATCATTGCATCTGCGCGCCTTCGTCAAGGCATTTT

CACCCCTCGTTCTGGATTTTCAGGGTGCGGGGCTTACCGCTTATCTCTCTGCCACCACCG

GGAACATGCTGATTTCTACACAGCCGCCGAGCTCGGTAAGGNTCGCACCTCAGCCCCACC

ATCGCGACCCACCCCCTGGGACACCCAGATAAGACGCGTCCATTGCTAACATGTTTTTCT

CCCCCACAGGTTCACCTTCAGACCGGCCAATGCGTAAGTTGCTGTCACCACCGGACCTTA

T---CGCCACCTGTAGCACGTTTCCCAGGGTAACCAAATCGGTGCTGCTTTCTGGTGCGT

CCCGAGTCTACCACCGCGACACTAGCTCGTAGCATACTGACATCATAGGCAAACCATCTC

TGGCGAGCACGGCCTCGACAGCAATGGCGTGTATGCACCTCCTATTCCTGTCCATCTCGT

CCTCCCTGATAATTGTACAGTTACAACGGCACTTCCGAGCTCCAGCTCGAGCGCATGAAC

GTCTACTTCAACGAGGTAAGTACGTCGTTTTGACCATCTACGGCCTTGCTAACGCGCTAT

CAGGCTTCCGGCAACAAGTATGTGCCTCGCGCCGTCCTCGTCGATCTCGAGCCCGGTACC

ATGGACGCCGTC

>Diaporthe_cameroonensis_STMA_18289

CTTTGTAAGTCCTCCTCGCCCTGCCCGCCGTTGCTGTCGCGCATGCTAACGGACCGTTTT

CGGCTTGCAGGATAAGGATGGCGATGGTTAGTGCAACCACTGCCAACTAGCACGCGTCAC

GCTTGATCCGCTACGACGGTCTGCGCGCAAGCGACCGTCGTCCCCATCACGAGTTGCTAA

GGTGTAGGACAAATCACCACCAAGGAGCTCGGCACGGTCATGAGGTCCCTGGGTCAAAAC

CCCTCCGAGTCCGAGCTGCAGGACATGATCAACGAGGTCGACGCCGACAACAATGGCACC

ATTGACTTTGGTAAGTCCAGATGCTCGCCTATCCGCGCCCTCTACCGGAGGTGTCAAGAA

GCCTCACCGCTACAAGCCTGGTACCGTCGCTCTGCGTGAGATCCGTCGCTACCAGAAGAG

CACCGAGCTGCTGATCCGCAAGCTCCCCTTCCAGCGTCTGGTATGCAGGTTCGTGAGATC

GCCCAGGACTTCAAGTCCGACCTGCGCTTCCAGTCTTCCGCCATTGGTGCCCTGCAGGAG

TCCGTCGAGTCCTACCTCGTCTCCCTCTTCGAGGACACCAACCTGTGCGCCATCCACGCC

AAGCGTGTCACCATCCAGTCGGTACGTNNNNNNNNNNNNNNNNNNNNNNNNNNNNNNNNN

NCATTGCTGCTCG-GGCGCACCCAGAAACCCTTTGTGAACTTTA-TTGTTGCCTCGGGAG

GCCGGGAGGCCCCCCGAGACGGGGAGCA-GCCCGCCGGCGGCCAACCAAACTCTTGTTTC

T-TAGTGAATCTCTAAATGAATCAAAACTTTCAACAACGGATCTCTTGGTTCTGGCATCG

ATGAAGAACGCAGCGAAATGCGATAAGTAATGTGAATTGCAGAATTCAGTGAATCATCGA

ATCTTTGAACGCACATTGCGCCCTCTGGTATTCCGGAGGGCATGCCTGTTCGAGCGTCAT

TTCAACCCTCAAGCCTGGCTTGGTGTTGGGGCGCTGAGGAGCAGGCCCTGAAATCTAGTG

GCGAGCTCGCTAGGACCCCGAGCGTAGTAGA-TCTCGTTAAGGCCCTGGCGGCGCCCTGC

TGAAAGAGAAGGAAGGTTAGTAAACATCATTGCATCTGCGCGCCTTCGTCAAGGCATTTT

CACCCCTCGTTCTGGATTTTCAGGGTGCGGGGCTTACCGCTTATCTCTCTGCCACCACCG

GGAACATGCTGATTTCTACACAGCCGCCGAGCTCGGTAAGGGTCGCACCTCAGCCCCACC

ATCGCGACCCACCCCCTGGGACACCCAGATAAGACGCGTCCATTGCTAACATGTTTTTCT

CCCCCACAGGTTCACCTTCAGACCGGCCAATGCGTAAGTTGCTGTCACCACCGGACCTTA

T---CGCCACCTGTAGCACGTTTCCCAGGGTAACCAAATCGGTGCTGCTTTCTGGTGCGT

CCCGAGTCTACCACCGCGACACTAGCTCGTAGCATACTGACATCATAGGCAAACCATCTC

TGGCGAGCACGGCCTCGACAGCAATGGCGTGTATGCACCTCCTATTCCTGTCCATCTCGT

CCTCCCTGATAATTGTACAGTTACAACGGCACTTCCGAGCTCCAGCTCGAGCGCATGAAC

GTCTACTTCAACGAGGTAAGTACGTCGTTTTGACCATCTACGGCCTTGCTAACGCGCTAT

CAGGCTTCCGGCAACAAGTATGTGCCTCGCGCCGTCCTCGTCGATCTCGAGCCCGGTACC

ATGGACGCCGTC

>Diaporthe_cameroonensis_CBS_148913

CTTTGTAAGTCCTCCTCGCCCTGCCCGCCGTTGCTGTCGCGCATGCTAACGGACCGTTTT

CGGCTTGCAGGATAAGGATGGCGATGGTTAGTGCAACCACTACCAACTAGCACGCGTCAC

GCTTGATCCGCTACGACGGTCTGCGCGCAAGCGACCGTCGTCCCCATCACGAGTTGCTAA

GGTGTAGGACAAATCACCACCAAGGAGCTCGGCACGGTCATGAGGTCCCTGGGTCAAAAC

CCCTCCGAGTCCGAGCTGCAGGACATGATCAACGAGGTCGACGCCGACAACAATGGCACC

ATTGACTTTGGTAAGTCCAGATGCTCGCCTATCCGCGCCCTCCACCGGAGGTGTCAAGAA

GCCTCACCGCTACAAGCCTGGTACCGTCGCTCTGCGTGAGATCCGTCGCTACCAGAAGAG

CACCGAGCTGCTGATCCGCAAGCTCCCCTTCCAGCGTCTGGTATGCAGGTTCGTGAGATC

GCCCAGGACTTCAAGTCCGACCTGCGCTTCCAGTCTTCCGCCATTGGTGCCCTGCAGGAG

TCCGTCGAGTCCTACCTCGTCTCCCTCTTCGAGGACACCAACCTGTGCGCCATCCACGCC

AAGCGTGTCACCATCCAGTCGGTACGTAACAAGGTCTCCGTTGGTGAACCAGCGGAGGGA

TCATTGCTGCCTC-GGCGCACCCAGAAACCCTTTGTGAACTTTG-TTGTTGCCTCGGTAG

GCCGGGAGGCCCCCCGAAACGGGGAGCA-GCCCGCCGGCGGCCAACCAAACTCTTGTTTC

T-TAGTGAATCTCTAAATGAATCAAAACTTTCAACAACGGATCTCTTGGTTCTGGCATCG

ATGAAGAACGCAGCGAAATGCGATAAGTAATGTGAATTGCAGAATTCAGTGAATCATCGA

ATCTTTGAACGCACATTGCGCCCTCTGGTATTCCGGAGGGCATGCCTGTTCGAGCGTCAT

TTCAACCCTCAAGCCTGGCTTGGTGTTGGGGCGCTGAGGAGCAGGCCCTGAAATCTAGTG

GCGAGCTCGCTAGGACCCCGAGCGTAGTAGA-TCTCGTTAAGGCCCTGGCGGCGCCCTGC

TGAAAGAGAAGGAAGGTTAGTAAACATCATTGCATCTGCGCGCCTTCGTCAAGGCATTTT

CACCCCTCGTTCTGGATTTTCAGGGTGCGGGGCTTACCGCTTATCTCTCTGCCACCACCG

GGAACATGCTGATTTCTACACAGCCGCCGAGCTCGGTAAGGGNNNNNNNNNNNNNNNNNN

NNNNNNNNNNNNNNNNNNNNNNNNNNNNNNNNNNNNNNNNNNNNNNNNNNNNNNNNNNNN

NNNNNNNNNNNNNNNNNNNNNNNNNNNNNNNNNNNNNNNNNNNNNNNNNNNNNNNNNNNN

NNNNNNNNNNNNNNNNNNNNNNNNNNNNNNNNNNNNNNNCGGTGCTGCTTTCTGGTGCGT

CCCGAGTCTACCACCGCGACACTAGCTCGTAGCATACTGACACCATAGGCAAACCATCTC

TGGCGAGCACGGCCTCGACAGCAATGGCGTGTATGCACCTCCTATTCCTGCCCATCTCGT

CCTCCCTGATGATTGCACAGTTACAACGGCACTTCCGAGCTCCAGCTCGAGCGCATGAAC

GTCTACTTCAACGAGGTAAGTACGTCGTTTTGACCATCTACGGCCTTGCTAACGCGCTAT

CAGGCTTCCGGCAACAAGTATGTGCCTCGCGCCGTCCTCGTCGATCTCGAGCCCGGTACC

ATGGACGCCGTC

>Diaporthe_camporesii_JZB320143

????????????????????????????????????????????????????????????

????????????????????????????????????????????????????????????

????????????????????????????????????????????????????????????

????????????????????????????????????????????????????????????

????????????????????????????????????????????????????????????

????????????????????????????????????????????????????????????

????????????????????????????????????????????????????????????

????????????????????????????????????????????????????????????

????????????????????????????????????????????????????????????

????????????????????????????????????????????????????????????

???????????????????????????NNNNNNNNNNNNNNNCCCTCGCGACGCGGAGGG

ACATTGCTGCTTC-GGCGCACCCAGAAACCCTTTGTGAACTTA--TTGTTGCCTCGGTAG

GCCGGGAGGCCCCCTGAAACAGGGAGCA-GCCCGCCGGCGGCCAACCAAACTCTTGTTTC

TACAGTGAATCTCTAAATGAATCAAAACTTTCAACAACGGATCTCTTGGTTCTGGCATCG

ATGAAGAACGCAGCGAAATGCGATAAGTAATGTGAATTGCAGAATTCAGTGAATCATCGA

ATCTTTGAACGCACATTGCGCCCTCTGGTATTCCGGAGGGCATGCCTGTTCGAGCGTCAT

TTCAACCCTCAAGCCTGGCTTGGTGATGGGGCACTGGAAAGCAGGCCCTGAAATCTAGTG

GCGAGCTCGCCAGGACCCCGAGCGTAGTAGA-TCTCGCTAAGGCCCTGGCGGTGCCCTGC

TGAAA???????????????????????????????????????????????????????

????????????????????????????????????????????????????????????

??????????????????????????????????????????NNNNNNNNNNNNNNNNNN

NNNNNNNNNNNNNNNNNNNNNNNNNNNNNNNNNNNNNNNNNNNNNNNNNNNNNNNN-NNN

NNNNNNNNNNNNNNNNNNNNNNNNNNNNNNNNNNNNNNNNNNNNNNNNNNNNNNNNNNNN

NNNNNNNNNNNNNNNNNNNNNNNNNNNNNNNNNNNNNNNNNNNNNNNNNNNNNTATGAAC

ACGAAGCCTACCGCCGCGAGACCAGCTCGCAACATACTGACCTCGTAGGCAAACCATCTC

TGGCGAGCACGGCCTCGACAGCAATGGCGTGTATGCACCTCCTATCCCTGCCCATCTCGT

CCTCCCTGATGATTGCACAGCTACAACGGCACTTCCGAGCTCCAGCTCGAGCGCATGAAC

GTCTACTTCAACGAGGTATGTACGTCGTTTTGCCCATCTGCTGCCCTCCTAACGCGTTAT

CAGGCTTCCGGCAACAAGTATGTGCCCCGCGCTGTCCTCGTCGACCTCGAGCCCGGTACC

ATGGACGCCGTC

>Diaporthe_eres_CFCC_51632

NTTTGTAATCTCCAGCCGACACGCCCGCCCTTGCTGGTGCGCATGCTAACGGACCGTTTT

CGGCTTGTAGGATAAGGATGGCGATGGTTAGTGCGGCCGCTCCCACCTAGCACGCGTCAT

GTTCGATCCGCCGCGACAGCCTGCGCGCAACCAAGCGTTATCACTATCACGAGTTGCTGA

GGTGTAGGGCAAATCACCACCAAGGAGCTCGGCACGGTCATGCGATCCCTGGGTCAGAAC

CCGTCCGAGTCTGAGCTGCAAGATATGATTAACGAGGTCGACGCCGACAACAATGGCACC

ATTGACTTTGGTACGTCCAGATGCTCGCGCTTCCGCGCCCTCCACCGGAGGTGTCAAGAA

GCCTCACCGCTACAAGCCTGGTACCGTCGCTCTGCGTGAGATCCGTCGCTACCAGAAGAG

CACCGAGCTGCTGATCCGCAAGCTCCCCTTCCAGCGTCTGGTATGTAGGTCCGTGAGATC

GCCCAGGACTTCAAGTCCGACCTCCGCTTCCAGTCTTCCGCCATCGGTGCCCTGCAGGAG

TCGGTCGAGTCTTACCTCGTCTCCCTCTTCGAGGACACCAACCTGTGCGCCATCCACGCC

AAGCGTGTCACCATCCAGTCGGTACGTNNNNNNNNNNNNNNNNNNNNNNNNNNNNNNNNN

NNNNNNNNNNNNNNNNNNNNNNNNNNNNCCCTTTGTGAACTTTTACTGTTGCCTCGGCTA

GCTGGGGGGCCCCTCCGGGTGTTGAGACAGCCCGCCGGCGGCCAACCTAACGCTTGTTTT

TACACTGAAACTCTAAATGAATCAAAACTTTCAACAACGGATCTCTTGGTTCTGGCATCG

ATGAAGAACGCAGCGAAATGCGATAAGTAATGTGAATTGCAGAATTCAGTGAATCATCGA

ATCTTTGAACGCACATTGCGCCCTCTGGTATTCCGGAGGGCATGCCTGTTCGAGCGTCAT

TTCAACCCTCAAGCCTGGCTTGGTGATGGGGCACTGAAGGGCAGGCCCTGAAATTCAGTG

GCGAGCTCGCCAGGACCCCGAGCGCAGTAGACCCTCGCTAAGGCCCTGGCGGTGCCCTGC

TGAAANNNNNGGAAGGTTAGTAAATATCATCATGCTCGCGCGGCCTCGCCAGCGCATTTT

CACCCCTCGCTTTGGATTTTCAGAGTGCGGGGCTTAGGGCTTATCTTGTCACCACCACCG

AATATATGCTGATATCTACACAGCCGCCGAGCTTGGCAANNNTCGCACCTGAGCCCCACC

ATCGCGACCCACCCCCTGGGACACCCAGATAAGACGCGTCGATTGCTAACGTGTTTTTCT

CGCCTCTAGGTTCACCTTCAGACCGGCCAATGCGTAAGTTCCTGTCAC-AGCGGACCTTA

TCATCGCCACCTGTAGCACGTTTCCCAGGGTAACCAAATCGGTGCTGCTTTCTGGTGCGT

TCCAAGTCCACCGCCGCGATACGACCTCGAAACATGCTGACCTCGTAGGCAAACCATCTC

TGGCGAGCACGGCCTCGACAGCAATGGCGTGTATGCACCTCCTATTCCTGCCCATCTTGG

CTTCCCTGACAATCGCATAGTTACAACGGCACTTCTGAGCTCCAGCTCGAGCGCATGAAC

GTCTACTTCAACGAGGTAAGTAGAACATTCCGACCATCTCCAAGTTCGCTAACGCGGTAT

CAGGCCTCCGGCAACAAGTATGTTCCTCGCGCCGTCCTCGTCGATCTCGAGCCCGGTACC

ATGGACGCCGTC

>Diaporthe_canthii_CBS_132533

NNNNNNNNNNNNNNNNNNNNNNNNNNNNNNNNNNNNNNNNNNNNNNNNNNNNNNNNNNNN

NNNNNNNNNNNNNNNNNNNNNNNNNNNNNNNNNNNNNNNNNNNNNNNNNNNNNNNNNNNN

NNNNNNNNNNNNNNNNNNNNNNNNNNNNNNNNNNNNNNNNNNNNNNNNNNNNNNNNNNNN

NNNNNNNNNNNNNNNNNNNNCAAGGAGCTCGGCACGGTCATGCGGTCCCTGGGTCAGAAC

CCGTCCGAGTCTGAGCTGCAAGATATGATCAACGAGGTCGACGCCGACAACAATGGCACC

ATTGACTTTGGTACGTCCAGATGCTCGCCCA?????????????????????????????

????????????????????????????????????????????????????????????

????????????????????????????????????????????????????????????

????????????????????????????????????????????????????????????

????????????????????????????????????????????????????????????

???????????????????????????NACAAGGTCTCCGTTGGTGAACCAGCGGAGGGA

TCATTGCTGCTCCGGGCGCACCCAGAAACCCTTTGTGAACTTTTACTGTTGCCTCGGCAG

GCCGTGGGGTCCCTT--AACAAGGAGCA-GCCGGCCGGCGGCCAAGTTAACTC-TGTTTT

TAAACCTAATATCTAAATGAATCAAAACTTTCAACAACGGATCTCTTGGTTCTGGCATCG

ATGAAGAACGCAGCGAAATGCGATAAGTAATGTGAATTGCAGAATTCAGTGAATCATCGA

ATCTTTGAACGCACATTGCGCCCTCTGGTATTCCGGAGGGCATGCCTGTTCGAGCGTCAT

TTCAACCCTCAAGCCTGGCTTGGTGCTGGGGCACTGAGGAGCAGGCCCTGAAATATAGTG

GCGAGCTCGCCAGGACTCCGAGCGTAGTAGACCCTCGCTAAGG-CCTGGCGGTGCCCTGC

TGAAAGAGAAGGAAGGTTAGTAAACACCATCATGCTCTTGCGGC-TCGTCGGCGCATTTT

GACCCCTCCCTCTGGATTTTCAGGGTGCGGGGCTTAGAGCTTATCTCATC---------G

AAACCATACTAATATCTATACAGCCGCCGAGCTTGGTAAGGGNNNNNNNNNNNNNNNNNN

NNNNNNNNNNNNNNNNNNNNNNNNNNNNNNNNNNNNNNNNNNNNNNNNNNNNNNNNNNNN

NNNNNNNNNNNNNNNNNNNNNNNNNNNNNNNNNNNNNNNNNNNNNNNNNNNNNNNNNNNN

NNNNNNNNNNNNNNNNNNNNNNNNNNNNNNNNNNNNNNNNGGTGCTGCTTTCTGGTGCGT

AC-AAGTCCACCACCGCGATACGACCTCGAAGCATACTGACCTCGCAGGCAAACCATCTC

TGGCGAGCACGGTCTCGACAGCAATGGCGTGTATGCACCTCCTATTTCTGTCTTTCTCGT

-TGCTCTGACAATCACACAGTTACAACGGCACTTCTGAGCTCCAGCTCGAGCGCATGAAT

GTCTACTTCAACGAGGTAAGTACGTTT--TTGACCATCTACAGCTTGGCTAACGCGTTAT

CAGGCCTCCGGTAACAAGTATGTGCCCCGCGCCGTCCTCGTCGATCTCGAGCCCGGTACC

ATGGACGCCGTC

>Diaporthe_careyae_SDBR_CMU469

CTTTGTAAGTCATCCTC--AGCACCCGCCCTTGCTGTTGCGCATGCTAACGGGCCGTTTT

CGGCCTGCAGGATAAGGATGGCGATGGTTAGTGTGGCCGCACCCACGGAGCACGCGTCAT

GCTCGATCCGCCGCGACGGCCTGCGCCCGAGCGGGCGTCAGCACTATCAGGAATTGCTAA

GGCGTAGGACAAATCACCACGAAGGAGCTCGGCACTGTCATGCGGTCCCTGGGTCAGAAC

CCGTCCGAGTCTGAGCTGCAAGATATGATCAACGAGGTCGATGCCGACAACAACGGCACC

ATTGACTTTGGTACGTTCAGATGCTGGCCTATCCGCGCCCTCCACCGGAGGTGTCAAGAA

GCCTCACCGCTACAAGCCTGGAACCGTCGCTCTGCGTGAGATCCGCCGTTACCAGAAGAG

CACCGAGCTGCTGATTCGCAAGCTCCCCTTCCAGCGTCTGGTACGCAGGTTCGTGAGATC

GCCCAGGATTTCAAGTCCGACCTCCGCTTCCAGTCTTCTGCCATCGGTGCCCTGCAGGAG

TCTGTCGAGTCTTACCTTGTCTCCCTCTTTGAGGACACCAACCTGTGCGCCATCCACGCC

AAGCGTGTCACCATCCAGTCGGTATGTAACAAGGTCTCCGTTGGTGAACCAGCGGAGGGA

TCATTGCTGCCCTGGGCGCACCCAGAAACCCTTTGTGAACTTTTACTGTTGCCTCGGCAG

GCCGGGGGGCCCCTCGACACGAGGAGCAGGCCCGCCGGCGGCCAAGTTAACTCTTGTCTC

TACACTGGAACTCTAAATGAATCAAAACTTTCAACAACGGATCTCTTGGTTCTGGCATCG

ATGAAGAACGCAGCGAAATGCGATAAGTAATGTGAATTGCAGAATTCAGTGAATCATCGA

ATCTTTGAACGCACATTGCGCCCTCTGGTATTCCGGAGGGCATGCCTGTTCGAGCGTCAT

TTCAACCCTCAAGCCTGGCTTGGTGATGGGGCACTGAAGGGCAGGCCCTGAAATCCAGTG

GCGAGCTCGCCAGGACCCCGAGCGCAGTAGACCCTCGCTAAGGCCCTGGCGGTGCCCTGC

TGAAA???????????????????????????????????????????????????????

????????????????????????????????????????????????????????????

??????????????????????????????????????????NNNNNNNNNNNNNNNNNN

NNNNNNNNNNNNNNNNNNNNNNNNNNNNNNNNNNNNNNNNNNNNNNNNNNNNNNNNNNNN

NNNNNNNNNNNNNNNNNNNNNNNNNNNNNNNNNNNNNNNNNNNNNNNNNNNNNNNNNNNN

NNNNNNNNNNNNNNNNNNNNNNNNNNTTGGTAACCAAATCGGTGCTGCTTTCTGGTGCGT

C--AGTGACGCCGCAGCGGTACGAGCTCGCGACATACTGACCTCGTAGGCAAACCATCTC

TGGCGAGCACGGCCTCGACAGCAATGGCGTGTATGCACCTCCTATTCCTGCACAAATCGT

CCTCCCTGACAATTGCACAGTTACAACGGCACTTCCGAGCTCCAGCTCGAGCGCATGAAC

GTCTACTTCAACGAGGTATGTGCATTTTTTTGGTCATCGCCAACCTCGCTAACGCTTTGT

CAGGCCTCCGGCAACAAGTATGTGCCTCGCGCCGTCCTTGTCGATCTCGAGCCCGGTACC

ATGGACGCCGTC

>Diaporthe_carpini_CBS_114437

CTTTGTAAGTCATTCTCCACATGCCCGCCCTTGCTGTTGCGCATGCTAACGGACCGTTCT

CGGCCTGCAGGATAAGGATGGCGATGGTTAGTGTGGCCGCACCCACCGAGCACGCGTCAT

GCTCGATCCACCGCGACGGCCTGCGCGCGAGCAAGCGTCAGCACTATCAGGGATTGCTAA

GGCGTAGGACAAATCACCACGAAGGAGCTCGGCACTGTCATGCGGTCCCTGGGCCAGAAC

CCGTCCGAGTCTGAGCTGCAAGATATGATTAACGAGGTCGATGCCGACAACAATGGCACC

ATTGACTTTGGTATGTTCAGATGCTCGCTCATCCGCGCCCTCTACCGGAGGTGTCAAGAA

GCCTCACCGCTACAAGCCTGGTACCGTCGCTCTGCGTGAGATCCGCCGTTACCAGAAGAG

CACCGAGCTGCTGATCCGCAAGCTCCCCTTCCAGCGTCTGGTACGTAGGTTCGTGAGATC

GCCCAGGACTTCAAGTCCGACCTCCGCTTCCAGTCTTCCGCCATCGGTGCCCTGCAGGAG

TCCGTCGAGTCTTACCTCGTCTCCCTCTTCGAGGACACCAACCTGTGCGCCATCCACGCC

AAGCGTGTCACCATCCAGTCGGTATGTAACAAGGTCTCCGTTGGTGAACCAGCGGAGGGA

TCATTGCTGCCCCTGGCGCACCCAGAAACCCTTTGTGAACTTTTACTGTTGCCTCGGCAG

GCCGGGGGGCCCCTCGAGACGAGGAGCAGGCCCGCCGGCGGCCAAGCCAACTCTTGTTTT

TACACTGAAACTCTAAATGAATCAAAACTTTCAACAACGGATCTCTTGGTTCTGGCATCG

ATGAAGAACGCAGCGAAATGCGATAAGTAATGTGAATTGCAGAATTCAGTGAATCATCGA

ATCTTTGAACGCACATTGCGCCCTCTGGTATTCCGGAGGGCATGCCTGTTCGAGCGTCAT

TTCAACCCTCAAGCCTGGCTTGGTGATGGGGCACTGCAGGGCAGGCCCTGAAATTCAGTG

GCGAGCTCGCCAGGACCCCGAGCGCAGTAGACCCTCGCTAAGGCCCTGGCGGTGCCCTGC

TGAAAGAGAAGGAAGGTTAGTAAACATCACCGGTCCTGCGCAGCTCCGTCAGCGCATTTT

CACCCCTCGTTCTGGATTTTCAGAGTGCGGGGCTTACAGCTTATCTCACCACCACC---G

GAAAGATGCTAACAACTACACAGCCGCCGAGCTTGGCAAGGGTCGCACCTGAGCCCCACC

ATCGCGACCCACCCCCTCGGACACCCAGATAAGACGCGTCGATTGCTAACGTGTTTTCCT

CGCCCGCAGGTTCACCTTCAGACCGGCCAATGCGTAAGTTGCTGTAACCGCCGGACCTTA

T---CGCCACCTTTCGCACGTTTCCCAGGGTAACCAAATCGGTGCTGCTTTCTGGTGCGT

CCCAAACTCAACACCGCGATACGACCTCGCGGCATACTGACCTCGTAGGCAAACCATCTC

TGGCGAGCACGGCCTCGACAGCAATGGCGTGTATGCACCTCCTGTTCCTGCACATCTCGC

CTTCCCTGACAATTGCACAGTTACAACGGCACTTCCGAGCTCCAGCTCGAGCGCATGAAC

GTCTACTTCAACGAGGTATGTACATTGTTTCGACCGTCTTCAACCTTGCTAACGCGTTAT

CAGGCCTCCGGCAACAAGTATGTTCCTCGCGCGGTCCTCGTCGATCTCGAGCCCGGTACC

ATGGACGCCGTC

>Diaporthe_carriae_BRIP_59932a

????????????????????????????????????????????????????????????

????????????????????????????????????????????????????????????

????????????????????????????????????????????????????????????

????????????????????????????????????????????????????????????

????????????????????????????????????????????????????????????

????????????????????????????????????????????????????????????

????????????????????????????????????????????????????????????

????????????????????????????????????????????????????????????

????????????????????????????????????????????????????????????

????????????????????????????????????????????????????????????

???????????????????????????AACAAGGTCTCCGTTGGTGAACCAGCGGAGGGA

TCATTGCTGCCCCAGGCGCACCCAGAAACCCTTTGTGAACTTTTACTGTTGCCTCGGCTT

GCCGGGGGGCCCCTCGAGACGAGGAGCAGGCACGCCGGCGGCCAAGTTAACTCTTGTTTT

TACACTGAAACTCTAAATGAATCAAAACTTTCAACAACGGATCTCTTGGTTCTGGCATCG

ATGAAGAACGCAGCGAAATGCGATAAGTAATGTGAATTGCAGAATTCAGTGAATCATCGA

ATCTTTGAACGCACATTGCGCCCTCTGGTATTCCGGAGGGCATGCCTGTTCGAGCGTCAT

TTCAACCCTCAAGCCTGGCTTGGTGATGGGGCGCTGAAGGGCACGCCCTCAAATCTAGTG

GCGAGCTCGCCAGGACCCCGAGCGCAGTAGACCCTCGCTAAGGCCCTGGCGGTGCCCTGC

?????NAGAAGGAAGGTTAGTAAACATTGCCATGTTCGTGCAACTTCGTCAGCGCATTTT

CACCCCTCGCTCTGGACTTTCAGGGTGCGGGGCTTAGAGCTTATCTTCCA-ACACTACGG

GAAACATGCTGATATCTACACAGCCGCCGAGCTTGGCAAGGGTCGCACCTGAGCCCCACC

ATCGCGACCCACCCCCTGGGACACCCAGATAAGACGCGTCGATTGCTAACGTGTTTTTCT

CGCCTATAGGTTCACCTTCAGACCGGCCAATGCGTAAGTTGCTGTCACGGCGGA-CCTTA

TCATCGCCCATTGTAGCACGTTTCCCAGGGTAACCAAATCGGTGCTGCTTTCTGGTGCGT

CC-GAGTCCACCACCGCGATACGACCTCGAAACATACTGACCTCGTAGGCAAACCATCTC

TGGCGAGCACGGCCTCGACAGCAATGGCGTGTATGCACCTCCTATTTCCGCCTTTCTCGT

CTGCCCTGACAATCACACAGTTACAACGGCTCTTCCGAGCTCCAGCTCGAGCGCATGAAC

GTCTACTTCAACGAGGTAAGTACGTCG--TTGACCATCTACGGCTTTGCTAACGCGTTAT

CAGGCCTCCGGCAACAAGTTTGTTCCCCGCGCCGTCCTCGTCGATCTCGAGCCCGGTACC

ATGGACGCCGTA

>Diaporthe_caryae_CFCC_52563

NNNNNNNNNNNNNNNNNNNNNNNNCCGCCACTGCTTTTGCGCATGCTAACGGACCGTTTT

CGGCCTGCAGGATAAGGATGGCGATGGTTAGTGTGGTCACTCTCAGCTAGCACGCGTCAT

ATTCGATCCGCCGCGACGGTCTGCGCGCGAGCGACCGTCATCACTATCACGAGTTGCTAA

GGTGTAGGACAAATTACCACCAAGGAGCTCGGCACAGTCATGCGGTCCCTTGGTCAAAAC

CCTTCCGAGTCCGAGCTGCAGGACATGATCAACGAGGTCGACGCCGACAACAATGGCACC

ATTGACTTTGGTAAGTCTGGATGCTTATCCCTCCGCGCCCTCCACCGGAGGTGTCAAGAA

GCCTCACCGCTACAAGCCTGGTACCGTCGCTCTGCGTGAGATCCGTCGCTACCAGAAGAG

CACCGAGCTGCTGATCCGCAAGCTCCCCTTCCAGCGTCTGGTATGCAGGTCCGTGAGATC

GCCCAGGACTTCAAGTCCGACCTGCGCTTCCAGTCTTCCGCCATCGGTGCCCTTCAGGAG

TCCGTCGAGTCTTACCTCGTCTCCCTCTTTGAGGACACCAACCTGTGCGCCATCCACGCC

AAGCCAGTCACCATCCAGTCGGTACGTNNNNNNNNNNNNNNNNNNNNNNNNNNNNNNNNN

NNNNNNNNNNNNNNNNNNNNNNNNNNNNCCCTTTGTGAACTTA--CTGTTGCCTCGGCAG

GCCGGGGGGCCCCCCGAGACGGGGAGCA-GCCCGCCGGCGGCCAACTAAACTCTTGTTTC

TATAGTGAATCTCTAAATGAATCAAAACTTTCAACAACGGATCTCTTGGTTCTGGCATCG

ATGAAGAACGCAGCGAAATGCGATAAGTAATGTGAATTGCAGAATTCAGTGAATCATCGA

ATCTTTGAACGCACATTGCGCCCCCTGGTATTCCGGGGGGCATGCCTGTTCGAGCGTCAT

TTCAACCCTCAAGCCTGGCTTGGTGATGGGGCACTGAGGAGCAGGCCCTGAAATTCAGTG

GCGAGCTCGCCAGGACCCCGAGCGTAGTAGA-TCTCGCTAAGGCCCTGGCGGTGCCCTGC

TGAAAGAGAAGGAAGGTCAGTAAACATCATGGTTTTTGCACGGCTGCGTCAAGCCATTTT

CGCCCCTCCCTCTGGATTTTCAGGGTGCGGGGCTTACCGCTTATCTCACTGCCTGCTCCG

ACAACATGCTGAATCCCACACAGCCGCCGAGCTGGGTAAGGGTCGCACCTCAGCCCCACC

ATCGCGACCCACCCCCTGCGACACCCAGATAAGACGCGTCGATTGCTAACATGTTTTTCT

TGCCCACAGGTTCACCTTCAGACCGGCCAATGCGTAAGTTGCTGTCACCGCCCGACCTTA

T---CGCCACCCGTAGCACGTTTCCCAGGGTAACCAAATCGGTGCTGCTTTCTGGTGCGT

ACCGAGCCTGCCACCGCGAGACTAGCTCGCAACATACTGACCTCGTAGGCAAACCATCTC

TGGCGAGCACGGTCTCGACAGCAATGGCGTGTATGTACCTCCTATTCCTGCCCATCTCGT

CCTCCCTGATGATCGCACAGTTACAACGGCACTTCCGAGCTCCAGCTCGAGCGCATGAAT

GTCTACTTCAACGAGGTAAGCACGTCTTTTTGA-CCGTCTCGGCCTTGCTAATGCGCTCT

CAGGCCTCCGGCAACAAGTATGTGCCTCGCGCCGTCCTCGTCGATCTCGAGCCCGGTACC

ATGGACGCCGNN

>Diaporthe_cassines_CBS_136440

????????????????????????????????????????????????????????????

????????????????????????????????????????????????????????????

????????????????????????????????????????????????????????????

????????????????????????????????????????????????????????????

????????????????????????????????????????????????????????????

????????????????????????????????????????????????????????????

????????????????????????????????????????????????????????????

????????????????????????????????????????????????????????????

????????????????????????????????????????????????????????????

????????????????????????????????????????????????????????????

???????????????????????????AACAAGGTCTCCGTTGGTGAACCAGCGGAGGGA

TCATTGTTGCTCCG-GCGCATCCAGAAACCCTTTGTGAACTTATACTGTTGCCTCGGCAG

GCTGAGGGGTCCCTCTCGGTGAGGAGCAGGCCCGCCGGTGGCCAAGTTAACTCTTATTTT

TACACTGAAACTCTAAATGAATCAAAACTTTCAACAACGGATCTCTTGGTTCTGGCATCG

ATGAAGAACGCAGCGAAATGCGATAAGTAATGTGAATTGCAGAATTCAGTGAATCATCGA

ATCTTTGAACGCACATTGCGCCCTCCGGTACTCCGGAGGGCATGCCTGTTCGAGCGTCAT

TTCAACCCTCAAGCCTGGCTTGGTGATGGGGCACTGCAAGGCAGGCCCTTAAATTCAGTG

GCGAGCTCGCCAGGACCCTGAGCGCAGTAGACCCTCGCTAAGGTCTTGGTGTGGCCCTGC

TGAAAGAGAAGGAAGGTGAGTAAATATCCCCGCGCTTGCACGGCTGCGTCATCGCATTTT

CACCCCTCGTTCTGGATTTTCAGGGTGCGGGGCTTAGAGCTTATCTCACAACCACCATCC

GTAACGTGCTGACGCTTTACCAGCCGCCGAGCTTGGCAAGGG??????????????????

????????????????????????????????????????????????????????????

????????????????????????????????????????????????????????????

????????????????????????????????????????????????????????????

????????????????????????????????????????????????????????????

????????????????????????????????????????????????????????????

????????????????????????????????????????????????????????????

????????????????????????????????????????????????????????????

????????????????????????????????????????????????????????????

????????????

>Diaporthe_caulivora_CBS_127268

CTTTGTAACCCCCAGCCGACATGCCCGCCCTGCCCGTTGCGCATGCTAACGGACCGTTCT

CGGCTTGTAGGATAAGGATGGCGATGGTTAGTGCAGCCGGTTCCGCCTAACACGCGTCAC

GCTCGATCCGCCGCGACGGCCTGCGCGCTTTAAAGCGCTATCACTTTTGCGAATTGCTGA

GGTGTAGGACAAATCACCACCAAGGAGCTCGGCACAGTCATGCGGTCCCTGGGCCAGAAC

CCCTCCGAGTCTGAGCTGCAAGACATGATCAACGAGGTCGATGCCGACAACAATGGCACC

ATCGACTTTGGTACGTTCAGCTGCTGGTCCGTCCGCGCCCTCCACCGGAGGTGTCAAGAA

GCCTCACCGCTACAAGCCTGGTACCGTCGCTCTGCGTGAGATCCGTCGCTACCAGAAGAG

CACCGAGCTTCTGATCCGCAAGCTCCCCTTCCAGCGTCTGGTAAGCAGGTCCGTGAGATC

GCACAGGACTTCAAGTCCGACCTCCGCTTCCAGTCCTCCGCTATCGGTGCCCTGCAGGAG

TCCGTCGAGTCTTACCTCGTCTCCCTCTTCGAGGACACCAACCTGTGCGCCATCCACGCC

AAGCGTGTCACCATCCAGTCGGTACGTAACAAGGTCTCCGTTGGTGAACCAGCGGAGGGA

TCATTGCTGCCCCAGGCGCACCCAGAAACCCTTTGTGAACTTTTACTGTTGCCTCGGCAG

GCCGGGGGGCCCCCCGAGACGGGGAGCA-GCCCGCCGGCGGCCAAGCTAACTCTTGTTTT

TACACTGAAACTCTAAATGAATCAAAACTTTCAACAACGGATCTCTTGGTTCTGGCATCG

ATGAAGAACGCAGCGAAATGCGATAAGTAATGTGAATTGCAGAATTCAGTGAATCATCGA

ATCTTTGAACGCACATTGCGCCCTCTGGTATTCCGGAGGGCATGCCTGTTCGAGCGTCAT

TTCAACCCTCAAGCCTGGCTTGGTGTTGGGGCACTGAAGGGCAGGCCCTGAAATTCATTG

GCGAGCTCGCCAGGACCCCGAGCGTAGTAGACCCTCGCTAAGGCCCTGGCGGTGCCCTGC

TGAAANNNNNNNNNNNNNNNNNNNNNNTGTCTCATTTGCGCCGCATCATCAGCGCATTTT

CACCCCTCGCTCTGGATTTTCAGGGTGCGGGGCTTAGGGCTTATCTCACCATTTCCGCCA

AGCAGATGCTGATCTCTATATAGCCGCTGAGCTCGGTAAGGGTCGCACCTGAGCCCCACC

ATCGCGGCCCGCCCCTTGCGACACCCAGATCACACGCGTCGATTGCTAACTTGTTTTGCT

CGCTTCTAGGTTCACCTGCAGACCGGCCAATGCGTAAGCTGCTGTCACGGACGGGCCTTG

TCATCGCCACCCCTAGCACGTTTCCCAGGGTAACCAAATCGGTGCTGCTTTCTGGTGCGT

GCCAAGCCTGCCACCGCGATGCGACCTCGAGACACACTGACCCCGTAGGCAAACCATCTC

TGGCGAGCACGGTCTCGACAGCAATGGCGTGTATGCACTCC-TGTTCCTGCCCCTCGAGC

TCTCCCTGACAATTGCGCAGTTACAACGGCACTTCCGAGCTCCAGCTCGAGCGCATGAAC

GTCTACTTCAACGAGGTAAGCACGTCCT---GACCCGTCCTGGCCCTGCTGACCAGTTAT

CAGGCCTCCGGTAACAAGTATGTTCCCCGCGCCGTCCTCGTCGATCTCGAGCCCGGCACC

ATGGACGCTGTC

>Diaporthe_celastrina_CBS_139_27

NNNNNNNNNNNNNNGCCGACACGCCTGCCCTTGCTGGTGCGCATGCTAACGGACCGTTTT

CGGCTTGTAGGATAAGGATGGCGATGGTTAGTGCGGCCGCTCCCACCTAGCACGCGTCAT

GTTCGATCCGCCGCGACAGCCTGCGCGCAACCAAGCGTTATCACTATCACGAGTTGCTGA

GGTGTAGGGCAAATCACCACCAAGGAGCTCGGCACGGTCATGCGATCCCTGGGTCAGAAC

CCGTCCGAGTCTGAGCTGCAAGATATGATTAACGAGGTCGACGCCGACAACAATGGCACC

ATTGACTTTGGTACGTCCAGATGCTCGCGCTTCCGCGCCTTCCACCGGAGGTGTCAAGAA

GCCTCACCGCTACAAGCCTGGTACCGTCGCTCTGCGTGAGATCCGTCGCTACCAGAAGAG

CACCGAGCTGCTGATCCGCAAGCTCCCCTTCCAGCGTCTGGTATGTAGGTCCGTGAGATC

GCCCAGGACTTCAAGTCCGACCTCCGCTTCCAGTCTTCCGCCATCGGTGCCCTGCAGGAG

TCGGTCGAGTCTTACCTCGTCTCCCTCTTCGAGGACACCAACCTGTGCGCCATCCACGCC

AAGCGTGTCACCATCCAGTCGGTACGTAACAAGGTCTCCGTTGGTGAACCAGCGGAGGGA

TCATTGCTGCCCCAGGCGCACCCAGAAACCCTTTGTGAACTTTTACTGTTGCCTCGGCTA

GCTGGGGGGCCCCTCCGGGTGTTGAGACGGCCCGCCGGCGGCCAACCTAACTCTTGTTTT

TACACTGAAACTCTAAATGAATCAAAACTTTCAACAACGGATCTCTTGGTTCTGGCATCG

ATGAAGAACGCAGCGAAATGCGATAAGTAATGTGAATTGCAGAATTCAGTGAATCATCGA

ATCTTTGAACGCACATTGCGCCCTCTGGTATTCCGGAGGGCATGCCTGTTCGAGCGTCAT

TTCAACCCTCAAGCCTGGCTTGGTGATGGGGCACTGAAAAGCAGGCCCTGAAATTCAGTG

GCGAGCTCGCCAGGACCCCGAGCGCAGTAGACCCTCGCTAAGGCCCTGGCGGTGCCCTGC

TGAAAGAGAAGGAAGGTTAGTAAATATCATCATGCTCGCGCGGCCTCGCCAGCGCATTTT

CACCCCTCGCTTTGGATTTTCAGAGTGCGGGGCCTAGTGCTTATCTTGTCACCACCACCG

AATATATGCTGATATCTACACAGCCGCCGAGCTTGGTAAGGGTCGCACCTGAGCCCCACC

ATGGCGACTCACCCCCTGGGACACCCAGATAAGACGCGTCGATTGCTAACGTGTTTTTCC

CGCCTCTAGGTTCACCTTCAGACCGGCCAATGCGTAAGTTCCTGTCAC-AGCGGACCTTA

TCATCGCCACCTGTAGCACGTTTCCCAGGGTAACCAAATCGGTGCTGCTTTCTGGTGCGT

TCCAAGTCCACCGCCGCGATACGACCTCGAAGCATGCTGACCTCGTAGGCAAACCATCTC

TGGCGAGCACGGCCTCGACAGCAATGGCGTGTATGCACCTCCTATTCCTGCCCATCTTGG

CTTCCCTGACAATCGCATAGTTATAACGGCACTTCTGAGCTCCAGCTCGAGCGCATGAAC

GTCTACTTCAACGAGGTAAGTACATCATTCCGACCATCTCCAACCTCGCTAACGTGTTAT

CAGGCCTCCGGCAACAAGTATGTTCCTCGCGCCGTCCTCGTCGATCTCGAGCCCGGTACC

ATGGACGCCGTC

>Diaporthe_celeris_CBS_143349

NNNNNNNNNNNNNNNNNNNNNNNNNNNNNNNNNNTGGTGCGCATGCTAACGGACCGTTTT

CGGCTTGTAGGATAAGGATGGCGATGGTTAGTGCAGCCGCTCCCACCTAGCACGCGTCAT

GTTCGATCCGCCGCGACAGCCTGCGCGCAACCAAGCGTTATCACTATCACGAGTTGCTGA

GGTGTAGGACAAATCACCACCAAGGAGCTCGGCACGGTCATGCGATCCCTGGGTCAGAAC

CCGTCCGAGTCTGAGCTGCAAGATATGATTAACGAGGTCGACGCCGACAACAATGGCACC

ATTGACTTTGGTACGTCCAGATGCTCGCGCTTCCGCGCCCTCCACCGGAGGTGTCAAGAA

GCCTCACCGCTACAAGCCTGGTACCGTCGCTCTGCGTGAGATCCGTCGCTACCAGAAGAG

CACCGAGCTGCTGATCCGCAAGCTCCCCTTCCAGCGTCTGGTATGCAGGTCCGTGAGATC

GCCCAGGACTTCAAGTCCGACCTCCGCTTCCAGTCTTCCGCCATCGGTGCCCTGCAGGAG

TCGGTCGAGTCTTACCTCGTCTCCCTCTTCGAGGACACCAACCTGTGCGCCATCCACGCC

AAGCGTGTCACCATCCAGTCGGTACGTAACAAGGTCTCCGTTGGTGAACCAGCGGAGGGA

TCATTGCTGCCCCAGGCGCACCCAGAAACCCTTTGTGAACTTTTAATGTTGCCTCGGCTA

GCTGGGGGGCCCCTCCGGGTGTTGAGACAGCCCGCCGGCGGCCAACCCAACTCTTGTTTT

TACACTGAAACTCTAAATGAATCAAAACTTTCAACAACGGATCTCTTGGTTCTGGCATCG

ATGAAGAACGCAGCGAAATGCGATAAGTAATGTGAATTGCAGAATTCAGTGAATCATCGA

ATCTTTGAACGCACATTGCGCCCTCTGGTATTCCGGAGGGCATGCCTGTTCGAGCGTCAT

TTCAACCCTCAAGCCTGGCTTGGTGATGGGGCACTGAGAAGCAGGCCCTGAAATTCAGTG

GCGAGCTCGCCAGGACCCCGAGCGCAGTAGACCCTCGCTAAGGCCCTGGCGGTGCCCTGC

TGAAAGAAAGGAAGGTTTAGTAAATATCATCATGCTCGCGCGGCCTCGCCAGCGCATTTT

CACCCCTCGCTTTGGATTTTCAGAGTGCGGGGCTTAGGGCTTATCTTGTCACCACCACCG

AATATATGCTGATATCTACACAGCCGCCGAGCTTGGCAAGGGNNNNNNNNNNNNNNNNNN

NNNNNNNNNNNNNNNNNNNNNNNNNNNNNNNNNNNNNNNNNNNNNNNNNNNNNNNNNNNN

NNNNNNNNNNNNNNNNNNNNNNNNNNNNNNNNNNNNNNNNNNNNNNNNNNNNNNNNNNNN

NNNNNNNNNNNNNNNNNNNNNNNNNNNNNNNNNNNNNNNCGGTGCTGCTTTCTGGTGCGT

TCCAAGTCCACCGCCGCGATACGACCTCGAAACATGCTGACCTCGTAGGCAAACCATCTC

TGGCGAGCACGGCCTCGACAGCAATGGCGTGTATGCACCTCCTATTCCTGCCCATCTTGG

CTTCCCTGACAATCGCATAGTTACAACGGCACTTCTGAGCTCCAGCTCGAGCGCATGAAC

GTCTACTTCAACGAGGTAAGTACATCATTCCGATCATCTCCAACCTCGCTAACGTGTTAT

CAGGCCTCCGGCAACAAGTATGTTCCTCGCGCCGTCCTCGTCGATCTCGAGCCCGGTACC

ATGGACGCCGTC

>Diaporthe_celticola_CFCC_53074

NNNNNNNNNNNNNNNNNNNNNNNNNNGCCCTTACTGTTGCGCATGCTAACGGACCGTTCT

CGGCCCTCAGGATAAGGATGGCGATGGTTAGTGTGACCGCTTCCACCCTGTACGCGTCAC

GATCGAATGGCTGCGACGGCTTGCGCGCGACCAAACGAGACCATTATCAGCAGTTGCTAA

GGTGTAGGACAAATCACCACAAAGGAGCTCGGCACGGTCATGCGATCCCTGGGTCAGAAC

CCTTCCGAGTCTGAGCTACAGGACATGATTAACGAGGTCGACGCCGACAACAATGGCACC

ATCGACTTGGGTACGTTCAGCTGCTTGTTCGTCCGCGCCTTCCACTGGAGGTGTCAAGAA

GCCTCACCGCTACAAGCCTGGTACCGTCGCTCTGCGTGAGATTCGTCGCTACCAGAAGTC

CACCGAGCTTCTGATCCGCAAGCTCCCCTTCCAGCGTCTGGTAGGCAGGTTCGTGAGATC

GCACAGGACTTCAAGTCCGATCTTCGCTTCCAGTCCTCCGCCATCGGTGCCCTGCAGGAG

TCTGTTGAGTCCTACCTCGTCTCCCTCTTCGAAGACACCAATCTGTGTGCCATTCACGCC

AAGCGTGTCACCATCCAGTCGGTATGTNNNNNNNNNNNNNNNNNNNNNNNNNNNNNNNNN

NNNNNNNNNNNNNNNNNNNNNNNNNNNNCCCTTTGTGAACTTTTACTGTTGCCTCGGCAG

GCCGGGGGGCCCCTCCGGACGAGGAGCGGGCCCGCCGGCGGCCAAGTTAACTCTTATTTC

TACCCTGAAACTCTAAATGAATCAAAACTTTCAACAACGGATCTCTTGGTTCTGGCATCG

ATGAAGAACGCAGCGAAATGCGATAAGTAATGTGAATTGCAGAATTCAGTGAATCATCGA

ATCTTTGAACGCACATTGCGCCCTCTGGTATTCCGGAGGGCATGCCTGTTCGAGCGTCAT

TTCAACCCTCAAGCCTGGCTTGGTGATGGGGCACTGGAGGGCAGGCCCTGAAATACAGTG

GCGAGCTCGCCAGGACTCCGAGCGCAGTAGACCCTCGCTAAGG-ACTGGCGCGGCCCTGC

TGAAANNNNNNNAAGGTTAGTAAATAATCCCATAGTTGCACGGTGTCATCAGCGCATTTT

CACCCCTCGCTCCAGA-TTTCAGGGTGCGGGGCTTA-AGCTTATCT-----TCACTACGT

CGAACATGCTGATATCTTCACAGCCGCCGAGCTCGGTAANNNNNNNNNNNNNNNNNNNNN

NNNNNNNNNNNNNNNNNNNNNNNNNNNNNNNNNNNNNNNNNNNNNNNNNNNNNNNNNNNN

NNNNNNNNNNNNNNNNNNNNNNNNNNNNNNNNNNNNNNNNNNNNNNNNNNNNNNNNNNNN

NNNNNNNNNNNNNNNNNNNNNNNNNNNNNNNNNNNNNNNNNNTGCTGCTTTCTGGTGCGT

T----GTCCATCACCGCGATACCGCCGATCAATATACTGACCTCATAGGCAAACCATCTC

TGGCGAGCACGGCCTCGACACCAATGGCGTGTACGTACCTCCTATTCCTGCCTATCTCAA

TTTTGCTGACAACTGCACAGTTACAATGGCACTTCCGAGCTCCAGCTCGAGCGCATGAAC

GTCTACTTCAACGAGGTAAACGTGCTATTTCAACGAGCCCAAGCTTTGCTGACACGCTAT

TAGGCCTCCGGCAACAAGTATGTCCCTCGCGCCGTCCTCGTCGATCTTGAGCCCGGTACC

ATGGACGCCGTN

>Diaporthe_celtidis_NCYU_19_0357

????????????????????????????????????????????????????????????

????????????????????????????????????????????????????????????

????????????????????????????????????????????????????????????

????????????????????????????????????????????????????????????

????????????????????????????????????????????????????????????

????????????????????????????????????????????????????????????

????????????????????????????????????????????????????????????

????????????????????????????????????????????????????????????

????????????????????????????????????????????????????????????

????????????????????????????????????????????????????????????

???????????????????????????NNNNNNNNNTCCGTTGGTGAACCAGCGGAGGGA

-CATTGCTG-CCCCGGCGCACCCAGAAACCCTTTGTGAACTTATACCGTTGCCTCGGCTG

GCCGGGGGGCCCCCTGAGACAGGGAGCA-GCCCGCCGGCGGCCAGACAAACTCTTGTTTC

T-TAGTGGATCTCT-AATGAATCAAAACTTTCAACAACGGATCTCTTGGTTCTGGCATCG

ATGAAGAACGCAGCGAAATGCGATAAGTAATGTGAATTGCAGAATTCAGTGAATCATCGA

ATCTTTGAACGCACATTGCGCCCCCTGGTATTCCGGGGGGCATGCCTGTTCGAGCGTCAT

TTCACCCCTCAAGCCTGGCTTGGTGTTGGGGCACCGAAGGGCGGGCCCTTAAATCCAGCG

GCGAGCCAGAGGAAACGCCTTGCGTAGTAG-ATCTCGC-CCTGCCCTCA-AGCGCCCTGC

NNNNNGAGAAGGAAGGTTAGTAAACATTACTGCGTTTGCGCGGCTTCGTCAGGGCATTTT

CACCCCTCCCTTTGGATTTTCAGGGTGCGGGGCTTACGGCTTA--TCGCTGCCACCACCA

ATCAGAAGCTGACATCTCTATAGCCGCCGAGCTCGGTAAGGGNNNNNNNNNNNNNNNNNN

NNNNNNNNNNNNNNNNNNNNNNNNNNNNNNNNNNNNNNNNNNNNNNNNNNNNNNNNNNNN

NNNNNNNNNNNNNNNNNNNNNNNNNNNNNNNNNNNNNNNNNNNNNNNNNNNNNNNNNNNN

NNNNNNNNNNNNNNNNNNNNTTTCCCAGGGTAACCAAATCGGTGCTGCTTTCTGGTGCGT

CCAGAGTCGACCACCGCGACAGTAGCTCGTAGCATACTGACATCGTAGGCAAACCATCTC

TGGCGAGCACGGCCTCGACAGCAATGGCGTGTATGCACCTCCTATCCCTGCCTATCTCGT

CCTCCCTGACAATGGCACAGCTACAACGGCACTTCTGAGCTCCAGCTCGAGCGCATGAAC

GTCTACTTCAACGAGGTAAGCACGTTGGTTTGACCATCTGCGGCCTAGCTAACGCGTTAT

CAGGCCTCCGGCAACAAGTATGTGCCTCGCGCCGTCCTCGTCGATCTCGAGCCCGGTACC

ATGGATGCCGTC

>Diaporthe_ceratozamiae_CBS_131306

????????????????????????????????????????????????????????????

????????????????????????????????????????????????????????????

????????????????????????????????????????????????????????????

????????????????????????????????????????????????????????????

????????????????????????????????????????????????????????????

????????????????????????????????????????????????????????????

????????????????????????????????????????????????????????????

????????????????????????????????????????????????????????????

????????????????????????????????????????????????????????????

????????????????????????????????????????????????????????????

???????????????????????????AACAAGGTCTCCGTTGGTGAACCAGCGGAGGGA

TCATTGCTGCCCCAGGCGCACCCAGAAACCCTTTGTGAACTTTT-TTGTTGCCTCGGCAT

GCTGGGGGGTCCCCTGAGACAGGGAGCAGGCACGCCGGCGGCCAAGTTAACTCTTGTTTT

TACATTGAAACTCTAAATGAATCAAAACTTTCAACAACGGATCTCTTGGTTCTGGCATCG

ATGAAGAACGCAGCGAAATGCGATAAGTAATGTGAATTGCAGAATTCAGTGAATCATCGA

ATCTTTGAACGCACATTGCGCCCTCTGGTATTCCGGAGGGCATGCCTGTTCGAGCGTCAT

TTCAACCCTCAAGCACTGCTTGGTGTTGGGGCACTGCGAAGCAGGCCCTGAAATCTAGTG

GCGAGCTCGCCAGGACCCCGAGCGTAGTAGACCCTCGCTAAGGCCCTGGCGGTGCCCTGC

TGAAA???????????????????????????????????????????????????????

????????????????????????????????????????????????????????????

????????????????????????????????????????????????????????????

????????????????????????????????????????????????????????????

????????????????????????????????????????????????????????????

????????????????????????????????????????????????????????????

????????????????????????????????????????????????????????????

????????????????????????????????????????????????????????????

????????????????????????????????????????????????????????????

????????????????????????????????????????????????????????????

????????????????????????????????????????????????????????????

????????????

>Diaporthe_cercidis_CFCC_52565

NNNNNNNNNNNNNNNNNNNNNNNNCCGCTCTTGCTGCTGCGCATGCTAACGGACCGTTTT

CGGCTTGCAGGATAAGGATGGCGATGGTTAGTGCGGCCGCTCTCACACAGCACGCGTCAT

GCTCGATCCTCCGCGACGGCCTGCGCGTAACAATGCGTTATCACTATTGCGAGTTGCTGA

GGTGCAGGACAAATCACCACCAAGGAGCTCGGCACCGTCATGCGATCCCTGGGCCAGAAC

CCTTCCGAGTCTGAGCTGCAAGATATGATTAACGAGGTCGACGCCGACAACAACGGCACC

ATTGACTTTGGTACGTCCAGATATACGCCCATCCGCGCCCTCCACCGGAGGTGTCAAGAA

GCCTCACCGCTACAAGCCTGGTACCGTCGCTCTGCGTGAGATCCGTCGCTACCAGAAGAG

CACTGAGCTGCTGATCCGCAAGCTCCCCTTCCAGCGTCTGGTATGCAGGTCCGTGAGATC

GCCCAGGACTTCAAGTCCGACCTCCGCTTCCAGTCCTCCGCCATCGGTGCCCTGCAGGAG

TCCGTCGAGTCTTACCTCGTCTCCCTCTTCGAGGACACCAACCTGTGCGCCATCCACGCC

AAGCGTGTCACCATCCAGTCGGTACGTNNNNNNNNNNNNNNNNNNNNNNNNNNNNNNNNN

NNNNNNNNNNNNNNNNNNNNNNNNNNNNCCCTTTGTGAACTTTTACTGTTGCCTCGGCAT

GCTGGGGGGTCCCCTGAGACAGGGAGCAGGCACGCCGGCGGCCAAGTTAACTCTTGTTTT

TACACTGAAACTCTAAATGAATCAAAACTTTCAACAACGGATCTCTTGGTTCTGGCATCG

ATGAAGAACGCAGCGAAATGCGATAAGTAATGTGAATTGCAGAATTCAGTGAATCATCGA

ATCTTTGAACGCACATTGCGCCCTCTGGTATTCCGGAGGGCATGCCTGTTCGAGCGTCAT

TTCAACCCTCAAGCATTGCTTGGTGTTGGGGCACTGAAGGGCAGGCCCTGAAATCTAGTG

GCGAGCTCGCTAGGACCCCGAGCGTAGTAGACCCTCGCTAAGGCCCTGGCGGTGCCCTGC

TGAAAGAGAAGGAAGGTTAGTAAACATCATCACGTTGGTACGGCTTCGTCAGCGCATTTT

CACCCCTCGCTCTGGATTTTCAGGGTGCGGGGCTTAGAGCTTATCT-----CTACCGCTG

GAAACATGCTGATATCTACATAGCCGCTGAGCTTGGCAAGGGTCGCACCTGAGCCCCACC

ATCGCGACCCACCCCCTGGGACACCCAAATAAAACGCGTCGATTGCTAACGTGTTTTTCT

CGACTACAGGTTCACCTTCAGACCGGCCAATGCGTAAGTTGCTGTCACGACGGA-CCTTA

TCATCGCCACCCGTAGCACGTTTCCCAGGGTAACCAAATCGGTGCTGCTTTCTGGTGC--

-----GTCCACCACCGCGACATGACCTCGAGCCATACTGACCTCGCAGGCAAACCATCTC

TGGCGAGCACGGCCTCGACAGCAATGGCGTGTATGCACCTCCTATTTCTGCCTTTCTCGT

CTGCCCTGACAATCACACAGTTACAACGGCACTTCCGAGCTCCAGCTCGAGCGCATGAAC

GTCTACTTCAATGAGGTAAGTACGTCA--TTGACCATCTGCAGCCTTGCTAACGCGTTAT

CAGGCCTCCGGCAACAAGTATGTCCCTCGCGCCGTCCTCGTCGATCTCGAGCCCGGTACT

ATGGACGCCGNN

>Diaporthe_cerradensis_CMRP4331

NNNNNNNNNNNNNNNNNNNNNTTTCCGCCGTTGCTGTTGCGCATGCTAACGGCCCGTTTT

CGGCCTGCAGGATAAGGATGGCGATGGTTAGTGTTGTCACTCTCAGCTAGCACGCGTCGG

GCTCGACCCGCCGCGACGGCCTGCGCGTAAGCGACCGACGTCACTATGACGAGTAGCTAA

GGTGTAGGACAAATCACCACCAAGGAGCTCGGCACTGTCATGCGGTCCCTGGGTCAAAAC

CCCTCCGAGTCCGAGCTGCAGGACATGATCAACGAGGTCGACGCCGACAACAACGGCACC

ATTGACTTTGGTAAGTTCAAATGCTCGCCTGTCCGCGCCCTCTACCGGAGGTGTCAAGAA

GCCTCACCGCTACAAGCCTGGTACCGTCGCTCTGCGTGAGATCCGTCGCTACCAGAAGAG

CACCGAGCTGCTGATCCGCAAGCTCCCCTTCCAGCGTCTGGTATGCAGGTTCGTGAGATC

GCCCAGGACTTCAAGTCCGACCTGCGCTTCCAGTCTTCCGCCATCGGTGCCCTGCAGGAG

TCCGTCGAGTCGTACCTCGTCTCCCTCTTCGAGGACACCAACCTGTGTGCCATCCACGCC

AAGCGTGTCACCATCCAGTCGGTATGTAACAAGGTCTCCGTTGGTGAACCAGCGGAGGGA

TCATTGCTGCTTC-GGCGCACCCAGAAACCCTTTGTGAACTTTA-CTGTTGCCTCGGCAG

GCCGGGGGGCCCCTCGGAAGGAGGAGCA-GCCCGCCGGCGGCCAACTAAACTCTTGTTTC

T-TAGTGAATCTCTAAATGAATCAAAACTTTCAACAACGGATCTCTTGGTTCTGGCATCG

ATGAAGAACGCAGCGAAATGCGATAAGTAATGTGAATTGCAGAATTCAGTGAATCATCGA

ATCTTTGAACGCACATTGCGCCCTCTGGTATTCCGGAGGGCATGCCTGTTCGAGCGTCAT

TTCAACCCTCAAGCACTGCTTGGTGTTGGGGCACCGAAGGGCGGGCCCTGAAAACTAGTG

GCGAGCTCGCCAGGACCCCGAGCGTAGTAGA-TCTCGTTAAGGCCCTGGCGGTGCACTGC

TGAAANNNNNNNNNNNNNNNNNNNNNNCGTTGCGTCTGCGCGGCTTCGTCAAGGCATTTT

CACCCCTCCCTCTGGATTTTCAGGGTGCGGGGCTTACCGCTTATCTCGCTGCTACCACCG

GAAACACGCTGATTTCTACACAGCCGCCGAGCTGGGTAAGGGNNNNNNNNNNNNNNNNNN

NNNNNNNNNNNNNNNNNNNNNNNNNNNNNNNNNNNNNNNNNNNNNNNNNNNNNNNNNNNN

NNNNNNNNNNNNNNNNNNNNNNNNNNNNNNNNNNNNNNNNNNNNNNNNNNNNNNNNNNNN

NNNNNNNNNNNNNNNNNNNNNNNNNNNNNNNNNNNNNNNNNNNNNNNNNNNNNNNNNNNN

NNNNNNNCGACGACCGCGAGACTAGCTGGTAGCATACTGACTTCGTAGGCAAACCATCTC

TGGCGAGCACGGCCTCGACAGCAATGGCGTGTATGCACCTCCTATCCCTGTCCGCCTCGT

CCTCCCTGATGATCGCACAGTTACAACGGCACTTCCGAGCTCCAGCTCGAGCGCATGAAC

GTCTACTTCAACGAGGTCAGTACGTCGTTTTGATCGTCTACGGCCTTGCTAACGCGTTAT

CAGGCTTCCGGCAACAAGTATGTGCCTCGCGCCGTCCTCGTCGATCTCGAGCCCGGTACC

ATGGACGCCGTC

>Diaporthe_cf_heveae_1_CBS_852_97

CTTTGTAAGTCCTGTTC------CCTGACGTTGCTGTAGCGCATGCTAACGGACCGTTTT

CGGCCTCCAGGATAAGGATGGCGATGGTTAGTGCGGGTCCTTCTTCCCTTCACGCGTCAC

GATCGACTCGCCGTGATGCCCTGCGCGCGACCAAGCGCCATGACTATCCGGACGCACTAA

GGTGTAGGACAAATCACCACAAAGGAGCTCGGAACGGTCATGCGATCGCTGGGTCAGAAC

CCGTCCGAGTCTGAGCTGCAGGATATGATTAATGAGGTCGATGCCGACAACAATGGCACT

ATCGACTTTGGTACGTCTAGATGTTTTCTCGTCCGCGCCCTCCACCGGAGGTGTCAAGAA

GCCTCACCGCTACAAGCCCGGTACCGTCGCTCTGCGTGAGATTCGTCGCTACCAGAAGTC

GACCGAGCTCCTGATCCGCAAGCTCCCCTTCCAGCGTCTGGTATGCAGGTTCGTGAGATC

GCCCAGGACTTCAAGTCCGATCTCCGCTTCCAGTCTTCCGCCATCGGTGCTCTGCAGGAG

TCCGTCGAGTCTTACCTCGTCTCTCTCTTCGAGGACACCAATCTGTGCGCCATCCACGCC

AAGCGTGTCACCATCCAGTCGGTACGTAACAAGGTCTCCGTTGGTGAACCAGCGGAGGGA

TCATTGCTG-CCTCGGCGCACCCAGAAACCCTTTGTGAACTTTATTTGTTGCCTCGGAAG

GCTGGGGGGCCCCCCTCTCTGGGGAGCAGGCCCGCCGGCGGCCAAGTAAACTCTTGTTTT

TAC-CTGGAACTCTAAATGAATCAAAACTTTCAACAACGGATCTCTTGGTTCTGGCATCG

ATGAAGAACGCAGCGAAATGCGATAAGTAATGTGAATTGCAGAATTCAGTGAATCATCGA

ATCTTTGAACGCACATTGCGCCCTCTGGTATTCCGGAGGGCATGCCTGTTCGAGCGTCAT

TTCAACCCTCAAGCCTGGCTTGGTGATGGGGCACTGCAGAGCAGGCCCTGAAATACAGTG

GCGAGCTCGCCAGTACCCCGAGCGCAGTAGACCCTCGTTAAGGTGCTGGCGGTGCCCTGC

TGAAAGAGAAGGAAGGTGAGTAAATATGCCCTCGCTTGCACGGCTTCGTCATCGCATTTT

CACCCCTCGTTCTGGATTTTCAGGGTGCGGGGCTTAGAGCTTATCTCATAATGACCACTG

GCAACATGCTGATGTTTTACCAGCCGCCGAGCTTGGTAAGGGTCGCACCTGAGCCCCACC

ATCGCGACCCACCCCCCGAGACACCAAAATAAGACGCGTCGAATGCTGACTTGTTTTTCT

CGTCCACAGGTTCACCTCCAGACCGGCCAATGCGTAAGTTGCTGTCACCACCGCACCTTA

T---CGCCCCTTCTAGCACGTTTCCCAGGGTAACCAAATCGGTGCTGCTTTCTGGTGCGT

C----GTCCATCATCGCGACACGACCTCAGAACATACTGACCTCGCAGGCAAACCATCTC

TGGCGAGCACGGCCTCGACAGCAATGGCGTGTACGCACCTCCTATTCCTGACTGTTTTGT

TCCCGCTGACGGCCTCACAGTTACAACGGCACTTCTGAGCTCCAGCTCGAGCGCATGAAC

GTCTACTTCAACGAGGTAAGTGTGCTATTTTCACGAATCCCAGCTGTGCTGACACGTTAT

CAGGCCTCCGGCAACAAGTATGTGCCTCGCGCCGTCCTCGTCGATCTCGAGCCCGGTACC

ATGGACGCCGTC

>Diaporthe_cf_heveae_2_CBS_681_84

NNNNNNNNNTCTCCACCGACACGCCCGCCCTTACTGTTGCGCATGCTAACGGACCGTTTT

CGGCTTGTAGGATAAGGATGGCGATGGTTAGTGCGGCCGCTCTCACACAGCACGCGTCAT

GCTCGATCCGCCGCGATGGCCTGCGCGCAACCAAGCGTTATCACTATTGCGAGTTGCTGA

GGTGCAGGACAAATCACCACCAAGGAGCTCGGCACGGTCATGCGGTCCCTGGGACAGAAC

CCGTCCGAGTCTGAGCTGCAAGATATGATTAACGAGGTCGACGCCGACAACAACGGCACC

ATTGACTTTGGTATGTCGAGATATTCGCCTGTCCGCGCCCTCCACCGGAGGTGTCAAGAA

GCCTCACCGCTACAAGCCTGGTACCGTCGCTCTGCGTGAGATCCGTCGCTACCAGAAGAG

CACCGAGCTGCTGATCCGCAAGCTCCCCTTCCAGCGTCTGGTATGCAGGTTCGTGAGATC

GCCCAGGACTTCAAGTCCGACCTCCGCTTCCAGTCCTCCGCCATCGGTGCCCTGCAGGAG

TCTGTCGAGTCTTACCTCGTCTCCCTCTTCGAGGACACCAACCTGTGCGCCATCCACGCC

AAGCGTGTCACCATCCAGTCGGTACGTAACAAGGTCTCCGTTGGTGAACCAGCGGAGGGA

TCATTGCTGCCCCAGGCGCACCCAGAAACCCTTTGTGAACTTTTACTGTTGCCTCGGCAT

GCTGGGGGGTCCCTTGAGACGAGGAGCAGGCACGCCGGCGGCCAAGTTAACTCTTGTTTT

TACACTGAAACTCTAAATGAATCAAAACTTTCAACAACGGATCTCTTGGTTCTGGCATCG

ATGAAGAACGCAGCGAAATGCGATAAGTAATGTGAATTGCAGAATTCAGTGAATCATCGA

ATCTTTGAACGCACATTGCGCCCTCTGGTATTCCGGAGGGCATGCCTGTTCGAGCGTCAT

TTCAACCCTCAAGCATTGCTTGGTGTTGGGGCACTGAAAAGCAGGCCCTGAAATCTAGTG

GCGAGCTCGCCAGGACCCCGAGCGTAGTAGACCCTCGCTAAGGCCCTGGCGGTGCCCTGC

TGAAAGAGAAGGAAGGTTAGTAAACATCATCATGTTGGTACGGCTTCGTCAGCGCATTTT

CACCCCTCGCTCTGGATTTTCAGGGTGCGGGGCTTAGAGCTTATCTTGCTACTACCACCG

GAAACATGCTGATATCTACACAGCCGCCGAGCTTGGTAAGGGTCGCACCTGAGCCCCACC

ATCGCGACCCACCCCCTGGGACACCCAGATAAAACGCGTCGATTGCTAACGTGTTTTTCT

CGACTACAGGTTCACCTTCAGACCGGCCAATGCGTAAGTTGCTGTCACGCCGGG-CCTTA

TCATCGCCACCTGTAGCACGTTTCCCAGGGTAACCAAATCGGTGCTGCTTTCTGGTGC--

-----GTCCACCGCCGCGATACGACCTCGAACCATACTGACCTCGCAGGCAAACCATCTC

TGGCGAGCACGGCCTCGACAGCAATGGCGTGTATGCACCTCCTATTCCTACCTCTCTCGT

CTGCCCTGACAATCACACAGTTACAACGGCACTTCCGAGCTCCAGCTCGAGCGCATGAAC

GTCTACTTCAACGAGGTAAGTACGTCG--TTGACCATCTGCAGCCTTGCTAACGCGTTAT

CAGGCCTCCGGCAACAAGTATGTCCCTCGCGCCGTCCTCGTCGATCTCGAGCCCGGTACC

ATGGACGCCGTC

>Diaporthe_chamaeropis_CBS_454_81

CTTTGTAATCTCCAGCCGATATGCCCGCC--TGCTGTTGCGCATGCTAACGGACCGTTTT

CGGCTTCCAGGATAAGGATGGCGATGGTTAGTACGGCCGCTCCCACCCAACACGCGTCAC

GCTCGATCCGCCCCGATGGCCTGCGCGCAAACAAGCGTCACCACTATCACGAGTTGCTGA

GGTGTAGGACAAATCACCACCAAGGAGCTCGGCACGGTCATGCGGTCCCTGGGTCAGAAC

CCGTCCGAGTCGGAGCTGCAAGATATGATTAACGAGGTCGACGCCGACAACAACGGCACC

ATTGACTTTGGTACGTTCAGATGCTCGCCTCTCCGCGCCCTCCACCGGAGGTGTCAAGAA

GCCTCACCGCTACAAGCCTGGTACCGTCGCTCTGCGTGAGATCCGTCGTTACCAGAAGAG

CACTGAGCTGCTGATCCGCAAGCTCCCCTTCCAGCGTCTGGTATGCAGGTTCGTGAGATC

GCCCAGGACTTTAAGTCCGACCTCCGCTTCCAGTCCTCCGCCATCGGTGCCCTGCAGGAG

TCCGTCGAGTCTTACCTCGTCTCCCTGTTCGAGGACACCAACCTGTGCGCCATCCACGCC

AAGCGTGTCACCATCCAGTCGGTACGTAACAAGGTCTCCGTTGGTGAACCAGCGGAGGGA

TCATTGCTGCCCCTGGCGCACCCAGAAACCCTTTGTGAACTTTTACTGTTGCCTCGGCAG

GCCGTGGGGTCCCTTCTCAAAAGGAGCA-GCCGGCCGGTGGCCAAATTAACTC-TGTTTT

TAAACTGAAACTCTAAATGAATCAAAACTTTCAACAACGGATCTCTTGGTTCTGGCATCG

ATGAAGAACGCAGCGAAATGCGATAAGTAATGTGAATTGCAGAATTCAGTGAATCATCGA

ATCTTTGAACGCACATTGCGCCCTCTGGTATTCCGGAGGGCATGCCTGTTCGAGCGTCAT

TTCAACCCTCAAGCCTGGCTTGGTGTTGGGGCACTGGAGAGCAGGCCCTGAAATATAGTG

GCGAGCTCGCCAGGACTCCGAGCGTAGTAGACCCTCGCTAAGG-CCTGGCGGTGCCCTGC

TGAAAGAGAAGGAAGGTTAGTAAACACCATCATGTTCGTGCGGTTTCGTGGGCGCATTTT

CACCCCGCCCTCTGGATTTTCAGGGTGCGGGGCTTAGAGCTTATCTCACA---------A

AAAACATGCTGANNNNNNNNNNNNNNNNNNNNNNNNNNNNNNTTCGCGCCTGAACCCCAC

CATCGCGACACACCCCTAGGACACTCAGGTAAGACGCGTCGATTGCTAACATGTTTTTCT

CGCCTACAGGTTCACCTTCAGACCGGTCAATGCGTAAGTTGCTGTCACACCGGA-CCTTA

TCATCGCCACCTTTAGCACGTTTCCCAGGGTAACCAAATCGGTGCTGCTTTCTGGTGCGT

CC-AAGTCAACCACCGCGAGTCGACCTCGAAGCATACTGACCTCGCAGGCAAACCATCTC

TGGCGAGCACGGTCTCGACAGCAATGGCGTGTATGCACCTCCTATTCCTGTCTTTCTCGT

-TGCACTGACAATCACACAGTTACAACGGCACTTCCGAGCTCCAGCTCGAGCGCATGAAC

GTCTATTTCAACGAGGTAAGTACGTTG--TT-ACCATTCACAGCTTTGCTAACGCGTTAT

CAGGCCTCCGGCAACAAGTATGTGCCCCGCGCCGTCCTCGTCGATCTCGAGCCCGGTACC

ATGGACGCCGTC

>Diaporthe_charlesworthii_BRIP_54884m

????????????????????????????????????????????????????????????

????????????????????????????????????????????????????????????

????????????????????????????????????????????????????????????

????????????????????????????????????????????????????????????

????????????????????????????????????????????????????????????

????????????????????????????????????????????????????????????

????????????????????????????????????????????????????????????

????????????????????????????????????????????????????????????

????????????????????????????????????????????????????????????

????????????????????????????????????????????????????????????

???????????????????????????AACAAGGTCTCCGTTGGTGAACCAGCGGAGGGA

TCATTGCTGCCCCGGGCGCACCCAGAAACCCTTTGTGAACTTTTACTGTTGCCTCGGCAG

GCTGGGGGGCCCCTCCGGGTGTTGAGACAGCCCGCCGGCGGCCAACCTAACTCTTGTTTT

TACACTGAGACTCTAAATGAATCAAAACTTTCAACAACGGATCTCTTGGTTCTGGCATCG

ATGAAGAACGCAGCGAAATGCGATAAGTAATGTGAATTGCAGAATTCAGTGAATCATCGA

ATCTTTGAACGCACATTGCGCCCTCTGGCATTCCGGAGGGCATGCCTGTTCGAGCGTCAT

TTCAACCCTCAAGCCTGGCTTGGTGATGGGGCGCTGAAGAGCAGGCCCTGAAATTCAGTG

GCGAGCCCGCCAGGACCCCGAGCGCAGTAGACCCTCGCTAAGGCCCTGGCGGTGCCCTGC

TGAAANNGAAGGAAGGTTAGTAAACACCATCATGCTCGTGCGGCTTCGTCAGCGCATTTT

CACCCCTCGTTCTGGATTTTCAGGGTGCGGGGCTTAGGGCTTATCTCACTACTACCACCG

AATCT-CGCTGATATCTGAACAGCCGCCGAGCTTGGCAAGGGTCGCACCTGAGCCCCACC

ATCGCGACCCACCCCCTGGGACACCCAGATAAGACGCGTCGATTGCTAATGTGTTTTTCT

CGCCTATAGGTTCACCTTCAGACCGGCCAATGCGTAAGTTGCTGTCAC-AGCGGACCTTA

TCATCGCCACCTGTAGCACGTTTCCCAGGGTAACCAAATCGGTGCTGCTTTCTGGTGCGT

CCCAAGTCCACCACCGCGATGCGACCTCGAAACATGCTGACTTCGTAGGCAAACCATCTC

TGGCGAGCACGGCCTCGACAGCAATGGCGTGTATGCACCTCCTATTCCTGCCCATCTTGG

CTTCCCTGACAATTGCACAGTTACAACGGCACTTCTGAGCTCCAGCTCGAGCGTATGAAC

GTCTACTTCAACGAGGTGAGTACATCATCCTCACCATCCTCAACCTTGCTAACGGCTTAT

CAGGCCTCCGGCAACAAGTATGTTCCTCGCGCCGTCCTCGTCGATCTCGAGCCCGGTACT

ATGGACGCCGTC

>Diaporthe_chensiensis_CFCC_52567

NNNNNNNNNNNNNNNNNNNNNNNNCCGCCCTTGCTGGTGCGCATGCTAACGGCCCGTTTT

CGGCTTGTAGGATAAGGACGGCGATGGTTAGTGCGGCCGCTCTCACCTAGCACGCGTCAT

GTTCGATCCACCGCGACAGTCTGCGCGCAGCCAAGCGTTATCACTATCACGAGTTGCTGA

GGTGTAGGACAAATCACCACCAAGGAGCTCGGCACGGTCATGCGATCCCTGGGCCAGAAC

CCGTCCGAGTCTGAGCTGCAAGATATGATTAACGAGGTCGACGCCGACAACAATGGCACC

ATTGACTTTGGTACGTCCAGATGCTCGCGCTTCCGCGCCCTCCACCGGAGGTGTCAAGAA

GCCTCACCGCTACAAGCCTGGTACCGTCGCTCTGCGTGAGATCCGTCGCTACCAGAAGAG

CACCGAGCTGCTGATCCGCAAGCTCCCTTTCCAGCGTCTGGTATGCAGGTCCGTGAGATC

GCCCAGGACTTCAAGTCCGACCTCCGCTTCCAGTCTTCCGCCATCGGCGCCCTGCAGGAG

TCGGTCGAGTCTTACCTCGTCTCCCTCTTCGAGGACACCAACCTGTGCGCCATCCACGCC

AAGCGTGTCACCATCCAGTCGGTATGTNNNNNNNNNNNNNNNNNNNNNNNNNNNNNNNNN

NNNNNNNNNNNNNNNNNNNNNNNNNNNNCCCTTTGTGAACTTTTACTGTTGCCTCGGCTA

GCTGGGGGGCCCCTCCGGGTGTTGAGACAGCCCGCCGGCGGCCAACCTAACTCTTGTTTT

TACACTGAAACTCTAAATGAATCAAAACTTTCAACAACGGATCTCTTGGTTCTGGCATCG

ATGAAGAACGCAGCGAAATGCGATAAGTAATGTGAATTGCAGAATTCAGTGAATCATCGA

ATCTTTGAACGCACATTGCGCCCTCTGGTATTCCGGAGGGCATGCCTGTTCGAGCGTCAT

TTCAACCCTCAAGCCTGGCTTGGTGATGGGGCACTGAGAAGCAGGCCCTGAAATTCAGTG

GCGAGCTCGCCAGGACCCCGAGCGCAGTAGACCCTCGCTAAGGCCCTGGCGGTGCCCTGC

TGAAAGAGAAGGAAGGTTAGTAAATACCATCATGCTCGCGCGGCTTCGTCAGCGCATTTT

CACCCCTCGCTTTGGATTTTCAGGGTGCGGGGCTTAGGGCTTATCTCACCACCACCACCG

AACATATGCTGATATCTACACAGCCGCCGAGCTTGGTAAGGGTCGCACCTGAGCCCCACC

ATCGCGACCCACCCCCTGGGACACCCAGATAAGACGCGTCGATTGCTAACGTGTTTTTCT

CGCCTCTAGGTTCACCTTCAGACCGGCCAATGCGTAAGTTGCTGTCAC-AGCGGACCTTA

TCATCGCCACCTGTAGCACGTTTCCCAGGGTAACCAAATCGGTGCTGCTTTCTGGTGCGT

TACAAGTCCACCGCCGCGATACGACCTCGAAGGATGCTGACCTCGTAGGCAAACCATCTC

TGGCGAGCACGGCCTCGACAGCAATGGCGTGTATGCACCTCCTATTCCTGCCCATCTTGG

CTTCCCTGACAATCGCACAGTTACAACGGCACTTCTGAGCTCCAGCTCGAGCGCATGAAC

GTCTACTTCAACGAGGTAAGTACATCATTCCGACCATCTCCAACCTCGCTAACGCGTCAT

CAGGCCTCCGGCAACAAGTATGTTCCTCGCGCCGTCCTCGTCGATCTCGAGCCCGGTACC

ATGGACGCCGNN

>Diaporthe_chiangmaiensis_NI207

????????????????????????????????????????????????????????????

????????????????????????????????????????????????????????????

????????????????????????????????????????????????????????????

????????????????????????????????????????????????????????????

????????????????????????????????????????????????????????????

????????????????????????????????????????????????????????????

????????????????????????????????????????????????????????????

????????????????????????????????????????????????????????????

????????????????????????????????????????????????????????????

????????????????????????????????????????????????????????????

???????????????????????????NNNNNNNNNTCCGTTGGTGAACCAGCGGAGGGA

TCATTGCTGCCCCAGGCGCACCCAGAAACCCTTTGTGAACTTTTACTGTTGCCTCGGCAT

GCTGGGGGGTCCCTCGAGACGAGGAGCAGGCACGCCGGCGGCCAAGTTAACTCTTGTTTT

TACACTGAAACTCTAAATGAATCAAAACTTTCAACAACGGATCTCTTGGTTCTGGCATCG

ATGAAGAACGCAGCGAAATGCGATAAGTAATGTGAATTGCAGAATTCAGTGAATCATCGA

ATCTTTGAACGCACATTGCGCCCTCTGGTATTCCGGAGGGCATGCCTGTTCGAGCGTCAT

TTCAACCCTCAAGCATTGCTTGGTGTTGGGGCACTGAAAAGCAGGCCCTGAAATCTAGTG

GCGAGCTCGCCAGGACCCCGAGCGTAGTAGACCCTCGCTAAGGCCCTGGCGGTGCCCTGC

NNNNNNNNNNNNNNNNNNNNNNNNNNNCATCATGTTGGTATGGCTCCGTCAGCGCATTTT

CACCCCTCGCTCTGGATTTTCAGGGTGCGGGGCTTAGAGCTTATCTACCA-CCACCTCAG

GAAAAATGCTGATATCTACACAGCCGCCGAGCTTGGTAANNN??????????????????

????????????????????????????????????????????????????????????

????????????????????????????????????????????????????????????

????????????????????????????????????????????????????????????

????????????????????????????????????????????????????????????

????????????????????????????????????????????????????????????

????????????????????????????????????????????????????????????

????????????????????????????????????????????????????????????

????????????????????????????????????????????????????????????

????????????

>Diaporthe_chinensis_MFLUCC_19_0101

NNNNNNNNNNNNNNNNNNNNNNNCCCGCCCTTGCTGTCGCGCATGCTAACGGACCGTTTT

CGGCTCGCAGGATAAGGATGGCGATGGTTAGTGCAGCCACTCCCAACTAGCACGCGTCAC

TCTTGATCCGCTACGACGGTCTACGCGCAACCGACCGTCATCACCATCACGAGTTGCTAA

GGTGTAGGACAAATCACCACCAAGGAGCTCGGCACGGTCATGCGGTCCCTGGGTCAAAAC

CCCTCCGAGTCCGAGCTGCAGGATATGATCAATGAGGTCGACGCCGACAACAATGGCACC

ATTGACTTTGGTAAGTCTAGATGCTCGTATA?????????????????????????????

????????????????????????????????????????????????????????????

????????????????????????????????????????????????????????????

????????????????????????????????????????????????????????????

????????????????????????????????????????????????????????????

???????????????????????????NNNNNNNNNTCCGTTGGTGAACCAGCGGAGGGA

TCATTGCTGCTTC-GGCGCACCCAGAAACCCTTTGTGAACTTTA-CTGTTGCCTCGGCAG

GCCGGGAGGCCCCCCGAGACGGGGAGCA-GCCCGCCGGCGGCCAACCAAACTCTTGTTTC

TACAGTGAATCTCTAAATGAATCAAAACTTTCAACAACGGATCTCTTGGTTCTGGCATCG

ATGAAGAACGCAGCGAAATGCGATAAGTAATGTGAATTGCAGAATTCAGTGAATCATCGA

ATCTTTGAACGCACATTGCGCCCTCTGGTATTCCGGAGGGCATGCCTGTTCGAGCGTCAT

TTCAACCCTCAAGCCTGGCTTGGTGTTGGGGCACTGAAGGGCAGGCCCTGAAATCTAGTG

GCGAGCTCGCTAGGACCCCGAGCGTAGTAGA-TCTCGTTAAGGCCCTGGCGGTGCCCTGC

NNNNNNNNNNNNNNNNNNNNNNNNNNNCATTGCATCCGCGAGGCTTCGTCAAGGCATTTT

CACCCCTCGCTCTGGATTTTCAGGGTGCGGGGCTTACGGCTTATCTCGCTGCCTCCTCCG

GAATCATGCTGATTTAAAAACAGCCGCCGAGCTTGGTAANNNNNNNNNNNNNNNNNNNNN

NNNNNNNNNNNNNNNNNNNNNNNNNNNNNNNNNNNNNNNNNNNNNNNNNNNNNNNNNNNN

NNNNNNNNNNNNNNNNNNNNNNNNNNNNNNNNNNNNNNNNNNNNNNNNNNNNNNNNNNNN

NNNNNNNNNNNNNNNNNNNNNNNNNNNNNNNNNNNNNNNNNNNNNNNNNNNNNNNNNNNN

NNNNNNCCTACCATCGCGACACTAGCTCGCATCATACTGACCTCTTAGGCAAACCATCTC

TGGCGAGCACGGCCTCGACAGCAATGGCGTGTATGTACCTCCTATTCCTGCCCATCTCGT

TCCCCCTGACAATTGCATAGTTACAACGGCACTTCCGAGCTCCAGCTCGAGCGCATGAAC

GTCTACTTCAACGAGGTCAGTACGTCGCTCTGACCATCTACGGTCTTGCTAACGCGTTAT

CAGGCCTCCGGCAACAAGTATGTGCCTCGCGCCGTCCTCGTCGATCTCGAGCCCGGTACC

ATGGATGCCGTC

>Diaporthe_chongqingensis_PSCG_435

NNNNNNNNNNNNNNNNNNNNNNNNNAGCCCTCACTGTCGCGTATGCTAACGGACCGTTCT

CGGCCTCCAGGATAAGGATGGCGATGGTTAGTGCAGCTGCTCCCAGCTTGTACGCGTCAC

GATCGACCGGCCGCGACGCCTTGCGCGCAACCAAGC-CCATAACTACTAGGAGCTGCTAA

GGTGTAGGACAAATCACCACAAAGGAGCTCGGCACGGTCATGCGATCTCTGGGTCAGAAC

CCGTCCGAGTCTGAGCTGCAAGATATGATCAACGAGGTCGACGCCGACAACAATGGAACC

ATCGACTTTGGTACGTCCAGATGCTCGCTTGTCCGCGCCCTCCACCGGAGGTGTCAAGAA

GCCTCACCGCTACAAGCCTGGTACCGTCGCTCTGCGTGAGATTCGTCGCTACCAGAAGTC

CACTGAGCTTCTGATCCGCAAGCTGCCCTTCCAGCGTCTGGTACGCAGGTTCGTGAGATT

GCCCAGGACTTCAAGTCCGACCTCCGCTTCCAGTCCTCCGCCATCGGTGCCCTGCAGGAG

TCCGTCGAGTCCTACCTCGTCTCCCTCTTCGAGGACACCAACCTGTGCGCCATCCACGCC

AAGCGTGTCACCATCCAGTCGGTATGTNNNNNNNNNNNNNNNNNNNNNNNNNNNNNNNNN

NNNNNNNNNNNNNNNNNNNNNNNNNNNNCCCTTTGTGAACTTTTACTGTTGCCTCGGCAG

GCCGGGGGGCCCCTCCTGACGAGGAGCAGGCTCGCCGGCGGCCAAGTTAACTCTTGTTTT

TATTGTGAAACTCTAAATGAATCAAAACTTTCAACAACGGATCTCTTGGTTCTGGCATCG

ATGAAGAACGCAGCGAAATGCGATAAGTAATGTGAATTGCAGAATTCAGTGAATCATCGA

ATCTTTGAACGCACATTGCGCCCTCTGGTATTCCGGAGGGCATGCCTGTTCGAGCGTCAT

TTCAACCCTCAAGCCTGGCTTGGTGATGGGGCACTGAAAGGCAGGCCCTGAAATTCAGTG

GCGAGCTCGCCAGGACTCCGAGCGCAGTAGACCCTCGCTAAGG-ACTGGCGGTGCCCTGC

TGAAANNNNNNNNNNNNNNNNNNNNNNCCTGGCGTTCCCACGGCGTCATCAGCGCATTTT

CACCCCTCGCTCTGGATTTTCAGGGTGCGGGGCTTAGAGCTTATCTCGCCACCACTACCT

TGGACATGCTGACATCTTCACAGCCGCTGAGCTCGGTAAGGGNNNNNNNNNNNNNNNNNN

NNNNNNNNNNNNNNNNNNNNNNNNNNNNNNNNNNNNNNNNNNNNNNNNNNNNNNNNNNNN

NNNNNNNNNNNNNNNNNNNNNNNNNNNNNNNNNNNNNNNNNNNNNNNNNNNNNNNNNNNN

NNNNNNNNNNNNNNNNNNNNNNNNNNNNNNNNNNNNNNNNNNNNNNNNNNNNNNNNGAGG

G----ACTCATCACCGCGACACGACCTCGCAACATACTGACCTCGTAGGCAAACCATCTC

TGGCGAGCACGGCCTCGACACCAATGGCGTGTATGCACCTCCTATTCCTGCCCATCTCGG

CTTGGCTGACAATTGCACAGCTACAACGGCACTTCCGAGCTTCAGCTCGAGCGCATGAAC

GTCTACTTCAACGAGGTAAGTATGACATTTTGACGAGCTCCCGCTTTGCTGACCGCTTAT

CAGGCTTCCGGCAACAAGTATGTTCCTCGCGCCGTCCTCGTCGATCTCGAGCCCGGTACC

ATGGACGCCGTC

>Diaporthe_chromolaenae_MFLUCC_17_1422

????????????????????????????????????????????????????????????

????????????????????????????????????????????????????????????

????????????????????????????????????????????????????????????

????????????????????????????????????????????????????????????

????????????????????????????????????????????????????????????

????????????????????????????????????????????????????????????

????????????????????????????????????????????????????????????

????????????????????????????????????????????????????????????

????????????????????????????????????????????????????????????

????????????????????????????????????????????????????????????

???????????????????????????AACAAGGTCTCCGTTGGTGAACCAGCGGAGGGA

TCATTGCTGCTTC-GGCGCACCCAGAAACCCTTTGTGAACTTA--TTGTTGCCTCGGCAG

GCCGGGAGGCCCCCTGAAACAGGGAGCA-GCCCGCCGGCGGCCAACCAAACTCTTGTTTC

TATAGTGAATCTCTAAATGAATCAAAACTTTCAACAACGGATCTCTTGGTTCTGGCATCG

ATGAAGAACGCAGCGAAATGCGATAAGTAATGTGAATTGCAGAATTCAGTGAATCATCGA

ATCTTTGAACGCACATTGCGCCCTCTGGTATTCCGGAGGGCATGCCTGTTCGAGCGTCAT

TTCAACCCTCAAGCCTGGCTTGGTGATGGGGCACTGTAGGGCAGGCCCTGAAATCTAGTG

GCGAGCTCGCCAGGACCCCGAGCGTAGTAGA-TCTCGCTAAGGCCCTGGCGGTGCCCTGC

TGAAA???????????????????????????????????????????????????????

????????????????????????????????????????????????????????????

????????????????????????????????????????????????????????????

????????????????????????????????????????????????????????????

????????????????????????????????????????????????????????????

????????????????????????????????????????????????????????????

????????????????????????????????????????????????????????????

????????????????????????????????????????????????????????????

????????????????????????????????????????????????????????????

????????????????????????????????????????????????????????????

????????????????????????????????????????????????????????????

????????????

>Diaporthe_chrysalidocarpi_SAUCC194_35

TTTTGTAATTTCCAACCGACACGCCCGCCCTTGCTGTTGCGCATGCTAACGGACCGTTTT

CGGTTTGTAGGATAAGGATGGCGATGGTTAGTGCGGCCGCTCTCACACAGCACGCGTCAT

GCTCGATCCTCCGCGACGGCCTGCGCGCAACCAAGCGTTATCACTATTGCGAGTTGCTGA

GGTGCAGGACAAATTACCACCAAGGAGCTCGGCACCGTCATGCGATCCCTGGGCCAGAAC

CCTTCCGAGTCTGAGCTGCAAGATATGATTAACGAGGTCGACGCCGACAACAACGGCACC

ATTGACTTTGGTACGTCCAGATATACGCCCATCCGCGCCCTCCACCGGAGGTGTCAAGAA

GCCTCACCGCTACAAGCCTGGTACCGTCGCTCTGCGTGAGATCCGTCGCTACCAGAAGAG

CACTGAGCTGCTGATCCGCAAGCTCCCCTTCCAGCGTCTGGTATGCAGGTCCGTGAGATC

GCCCAGGACTTCAAGTCCGACCTCCGCTTCCAGTCCTCCGCCATCGGTGCCCTGCAGGAG

TCCGTCGAGTCTTACCTCGTCTCCCTCTTTGAGGACACCAACCTGTGCGCCATCCACGCC

AAGCGTGTCACCATCCAGTCGGTACGTNNNNNNNTCTCCGTTGGTGAACCAGCGGAGGGA

TCATTGCTGCCCTAGGCGCACCCAGAAACCCTTTGTGAACTTTTACTGTTGCCTCGGCAT

GCTGGGGGGCCCCCTGAGACAGGGAGCAGGCACGCCGGCGGCCAAGTTAACTCTTGTTTT

TACACTGAAACTCTAAATGAATCAAAACTTTCAACAACGGATCTCTTGGTTCTGGCATCG

ATGAAGAACGCAGCGAAATGCGATAAGTAATGTGAATTGCAGAATTCAGTGAATCATCGA

ATCTTTGAACGCACATTGCGCCCTCTGGTATTCCGGAGGGCATGCCTGTTCGAGCGTCAT

TTCAACCCTCAAGCATTGCTTGGTGTTGGGGCACTGAAGGGCAGGCCCTGAAATCTAGTG

GCGAGCTCGCCAGGACCCCGAGCGTAGTAGACCCTCGCTAAGGCCCTGGCGGTGCCCTGC

TGAAANNNNNNNNNNNNNNNNNGCGTACATCATGTTGGTACGGCTTCGTCAGCGCATTTT

CACCCCTCGCTCTGGATTTTCAGGGTGCGGGGCTTAGAGCTTATCT-----CTACCGCTG

GAAACATGCTGATATCTACATAGCCGCTGAGCTTNNNNNNNNNNNNNNNNNNNNNNNNNN

NNNNNNNNNNNNNNNNNNNNNNNNNNNNNNNNNNNNNNNNNNNNNNNNNNNNNNNNNNNN

NNNNNNNNNNNNNNNNNNNNNNNNNNNNNNNNNNNNNNNNNNNNNNNNNNNNNNNNNNNN

NNNNNNNNNNNNNNNNNNNNNNNNNNNNNNNNNNNNNNNNNNNNNNNNNNNNNNNNNNNN

NNNNNNNNNNNNNNCGCGATATGACCTCGAGCCATACTGACCTCGCAGGCAAACCATCTC

TGGCGAGCACGGCCTCGACAGCAATGGCGTGTATGCACCTCCTATTTCTGCCTTTCTCGT

CTGCTCTGACAATCACACAGTTACAACGGCACTTCCGAGCTCCAGCTCGAGCGCATGAAC

GTCTACTTCAATGAGGTAAGTATGTCG--TTGACCATCTGCAGCCTTGCTAACGCGTTAT

CAGGCCTCCGGCAACAAGTATGTCCCTCGCGCCGTCCTCGTCGATCTCGAGCCCGGTACT

ATGGACGCCGTC

>Diaporthe_cichorii_MFLUCC_17_1023

NNNNNNNNNNNNNNNNNNCCTTGCCCGCCATTGCCTTTGCGCATGCTAACGGACCGTTTT

CGGCCTGCAGGATAAGGATGGCGATGGTTAGTGCGGTCACTCTCAGCTACCACGCGTCAT

ACTCGATCCGCCGCGACGGTCTGCGCGTGAGCGAGCAACCTCATGATCACGAGATGCTAA

GGTGTAGGACAAATCACCACCAAGGAGCTCGGCACAGTCATGCGGTCCCTTGGTCAAAAC

CCTTCCGAGTCCGAGCTGCAGGACATGATCAACGAGGTCGACGCCGACAACAATGGCACC

ATTGACTTTGGTAAGTCTAGATGTCCACCCA?????????????????????????????

????????????????????????????????????????????????????????????

????????????????????????????????????????????????????????????

????????????????????????????????????????????????????????????

????????????????????????????????????????????????????????????

???????????????????????????NNNNNNNNNNNNNNNNNNNNNNNNNNNNNNNNN

NNNNNNNNNNNNNNNNNNNNNNCCAGAACCCTTTGTGAACTTATACTGTTGCCTCGGCAG

GCCGGGGGGCCCCCTGGGACAGGGAGCA-GCCCGCCGGCGGCCAACCAAACTC-TGTTTC

TATAGTGGATCTCTAAATGAATCAAAACTTTCAACAACGGATCTCTTGGTTCTGGCATCG

ATGAAGAACGCAGCGAAATGCGATAAGTAATGTGAATTGCAGAATTCAGTGAATCATCGA

ATCTTTGAACGCACATTGCGCCCTCTGGTATTCCGGAGGGCATGCCTGTTCGAGCGTCAT

TTCAACCCTCAAGCCTGGCTTGGTGATGGGGCACTGAAGGGCAGGCCCTGAAATCTAGTG

GCGAGCTCGCCAGGACCCCGAGCGTAGTAGA-TCTCGCTAAGGCCCTGGCGGTGCCCTGC

TGAAANNNNNNNNNNNNNNNNNNNNNNGATTGTGCCTGCGCGGCTTCGTCGAGCCATTTT

CACCCCTCCCTCTGGGTTTTCAGGGTGCGGGGCTTACCGCTTATCTCACCGTCAACACCG

AACAGACGCTGATTTCCACACAGCTGCCGAGCTGGGTAAGGGNNNNNNNNNNNNNNNNNN

NNNNNNNNNNNNNNNNNNNNNNNNNNNNNNNNNNNNNNNNNNNNNNNNNNNNNNNNNNNN

NNNNNNNNNNNNNNNNNNNNNNNNNNNNNNNNNNNNNNNNNNNNNNNNNNNNNNNNNNNN

NNNNNNNNNNNNNNNNNNNNNNNNNNNNNNNNNNNNNNNNNNNNNNNNNNNNNNNNNNNN

NNNNNNNNNACGACCGCGAGATTAGCTCGCAACGTACTGACCTCGTAGGCAAACCATCTC

TGGCGAGCACGGTCTCGACAGCAATGGCGTGTATGCACCTCCTGTTTCTGCCCATCTCGT

CCTCCCTGATGTTTGCACAGTTACAACGGCACTTCTGAGCTCCAGCTCGAGCGCATGAAC

GTCTACTTCAACGAGGTGAGTACGTCTTTTTGAGCCTCCTCGACCTTACTGACGCGCTTT

CAGGCCTCCGGCAACAAGTATGTGCCTCGCGCCGTCCTCGTCGATCTCGAGCCCGGTACC

ATGGACGCCGTC

>Diaporthe_cinerascens_CBS_719_96

CTTTGTAATCTCCAGCCGATATGCCCGCC--TGCTGTTGCGCATGCTAACGGACAGTTTT

CGGCTTCCAGGATAAGGATGGCGATGGTTAGTACGGCCGCTCCCACTCAACACGCGTCAC

GCTCGATCCGCCCCGATGGCCTGCGCGCAAAGAAGCGTTACCAATATCACGAGTTGCTGA

GGTGTAGGACAAATCACCACCAAGGAGCTCGGCACGGTCATGCGGTCCCTGGGTCAGAAC

CCGTCCGAGTCTGAGCTGCAAGATATGATTAACGAGGTCGACGCCGACAACAACGGCACC

ATTGACTTCGGTACGTTCAGACGCTCGCCTCTCCGCGCCCTCCACCGGAGGTGTCAAGAA

GCCTCACCGCTACAAGCCTGGTACCGTCGCTCTGCGTGAGATCCGTCGTTACCAGAAGAG

CACCGAGCTGCTGATCCGCAAGCTCCCCTTCCAGCGTCTGGTACGCAGGTTCGTGAGATC

GCCCAGGACTTCAAGTCCGACCTCCGCTTCCAGTCCTCCGCCATCGGTGCCCTGCAGGAG

TCCGTTGAGTCTTACCTCGTCTCCCTGTTCGAGGACACCAACCTGTGCGCCATCCACGCC

AAGCGTGTCACCATCCAGTCGGTATGTAACAAGGTCTCCGTTGGTGAACCAGCGGAGGGA

TCATTGCTGCCCCCGGCGCACCCAGAAACCCTTTGTGAACTTTTACTGTTGCCTCGGCAG

GCCGGGGGGTCCCTT--GACAAGGAGCA-GCCGGCCGGTGGCCAAGTTAACTC-TGTTTT

TACACTGAAACTCTAAATGAATCAAAACTTTCAACAACGGATCTCTTGGTTCTGGCATCG

ATGAAGAACGCAGCGAAATGCGATAAGTAATGTGAATTGCAGAATTCAGTGAATCATCGA

ATCTTTGAACGCACATTGCGCCCTCTGGTATTCCGGAGGGCATGCCTGTTCGAGCGTCAT

TTCAACCCTCAAGCCTGGCTTGGTGTTGGGGCACTGAGAAGCAGGCCCTGAAATGTAGTG

GCGAGCTCGCCAGGACTCCGAGCGCAGTAGACCCTCGCTAAGG-CCTGGCGGTGCCCTGC

TGAAAGAGAAGGAAGGTGAGTAAACGCTATCATATTCGTGCGGCTTCGTCGGCACATTTT

CACCCCGCCCTCTGGATTTTCAGGGTGCGGGGCTTAGAGCTTATCTCACC---------A

ACAACAAGCTGATCCTCTTACAGCCGCCGAGCTTGGTAAGGGTCGCACCTGAGCCCCACC

ATCGCGACCCACCCCCTAGGACACTCAGGTAAGACGCGTCGATTGCTAACATGTTTTTCT

CGCCTACAGGTTCATCTTCAGACCGGTCAATGCGTAAGTTGCTGTCACACCGGA-CCTTA

TCATCGCCACCTGCAGCACGTTTCCCAGGGTAACCAAATCGGTGCTGCTTTCTGGTGCGT

CC-TAGTCAACCACCGCGAGTCGACCTCGAAGTATACTGACCTCGCAGGCAAACCATCTC

TGGCGAGCACGGTCTCGACAGCAATGGCGTGTATGCACCTCCTATTCCTGTCTTTCTCGT

-TGCCCTGACAATCACACAGTTACAACGGCACTTCCGAGCTCCAGCTCGAGCGCATGAAC

GTCTACTTCAACGAGGTAAGTACGTTG--TT-ACCATCCACAGCTTTGCTAACGTGTTAT

CAGGCCTCCGGCAACAAGTATGTGCCCCGCGCCGTCCTCGTCGATCTCGAGCCCGGTACC

ATGGACGCCGTC

>Diaporthe_cinnamomi_CFCC_52569

????????????????????????????????????????????????????????????

????????????????????????????????????????????????????????????

????????????????????????????????????????????????????????????

????????????????????????????????????????????????????????????

????????????????????????????????????????????????????????????

???????????????????????????????TCCGCGCCCTCCACCGGAGGTGTCAAGAA

GCCTCACCGCTACAAGCCTGGTACCGTCGCTCTGCGTGAGATCCGTCGCTACCAGAAGAG

CACCGAGCTGCTGATCCGCAAGCTCCCCTTCCAGCGTCTGGTATGCAGGTTCGTGAGATC

GCCCAGGACTTCAAGTCCGACCTGCGCTTCCAGTCTTCCGCCATCGGTGCCCTGCAGGAG

TCCGTCGAGTCCTACCTCGTCTCCCTCTTTGAGGACACCAACCTGTGCGCCATCCACGCC

AAGCGTGTCACCATCCAGTCGGTACGTNNNNNNNNNNNNNNNNNNNNNNNNNNNNNNNNN

NNNNNNNNNNNNNNNNNNNNNNNNNNNNCCCTTTGTGAACTTTA-CTGTTGCCTCGGCAG

GCCGGGAGGCCCCCCGAGACGGGGAGCA-GCCCGCCGGCGGCCAAGTTAACTCTTGTTTC

TACCCTGAATCTCTAAATGAATCAAAACTTTCAACAACGGATCTCTTGGTTCTGGCATCG

ATGAAGAACGCAGCGAAATGCGATAAGTAATGTGAATTGCAGAATTCAGTGAATCATCGA

ATCTTTGAACGCACATTGCGCCCTCTGGTATTCCGGAGGGCATGCCTGTTCGAGCGTCAT

TTCAACCCTCAAGCCTGGCTTGGTGCTGGGGCACTGAGAAGCAGGCCCTGAAATCTAGTG

GCGAGCTCGCTAGGACCCCGAGCGTAGTAAA-TCTCGTTAAGGCCCTGGCGGTGCCCTGC

TGAAAGAGAAGGAAGGTTAGTAAACATCATTGCATCTGCGCGGCTTCGTCAAGGCATTTT

CACCCCTCGCTCTGGATTTTCAGGGTGCGGGGCTTACGGCTTATCTCGCTGCTAGTACCG

GAATTATGCTGATTCCTACACAGCCGCCGAGCTTGGTAAGGGTCGCACCTAAGCCCCACC

ATCGCGACCCACCCCCTGGGACACCCAGATAAGACGCGTCGATTGCTAACATGTTTTTCT

CGACCACAGGTTCACCTTCAGACCGGCCAATGCGTAAGTTGCTGTCACCACCGGACCTTA

T---CGCCACCTGTAGCACGTTTCCCAGGGTAACCAAATCGGTGCTGCTTTCTGGTGCGT

CCCAAGCCTACCACCGCGAGACTAGCTCGCAACATACTGACCTCGTAGGCAAACCATCTC

TGGCGAGCACGGCCTCGACAGCAATGGCGTGTATGTACCTCCTATTCCTGCCCATCTCGT

CCCCCCTGACAACTGCACAGTTACAACGGCACTTCTGAGCTCCAGCTCGAGCGCATGAAC

GTCTACTTCAACGAGGTAAGTACGTCGTTTTGTCCATCTACTGTCTTGCTAACGCATTAT

CAGGCCTCCGGCAACAAGTATGTGCCTCGCGCCGTCCTCGTCGATCTCGAGCCCGGTACC

ATGGACGCCGNN

>Diaporthe_cissampeli_CBS_141331

????????????????????????????????????????????????????????????

????????????????????????????????????????????????????????????

????????????????????????????????????????????????????????????

????????????????????????????????????????????????????????????

????????????????????????????????????????????????????????????

???????????????????????????????TCCGCGCCCTCCACCGGAGGTGTCAAGAA

GCCTCACCGCTACAAGCCTGGTACCGTCGCTCTGCGTGAGATCCGTCGTTACCAGAAGAG

CACCGAGCTGCTGATCCGCAAGCTCCCCTTCCAGCGTCTGGTATGCAGGTTCGTGAGATC

GCCCAGGACTTCAAGTCCGACCTCCGCTTCCAGTCCTCTGCCATCGGTGCCCTGCAGGAG

TCCGTCGAGTCTTACCTCGTCTCCCTGTTCGAGGACACCAACCTGTGCGCCATCCACGCC

AAGNNNNNNNNNNNNNNNNNNNNNNNNAACAAGGTCTCCGTTGGTGAACCAGCGGAGGGA

TCATTGCTGCCCCAGGCGCACCCAGAAACCCTTTGTGAACTTTTACCGTTGCCTCGGCGG

GCCGGGGGGTCCCTT--GACAAGGAGCA-GCCGGCCGGTGGCCAAATAAACTC-TGTTTT

TACACTGAAACTCTAAATGAATCAAAACTTTCAACAACGGATCTCTTGGTTCTGGCATCG

ATGAAGAACGCAGCGAAATGCGATAAGTAATGTGAATTGCAGAATTCAGTGAATCATCGA

ATCTTTGAACGCACATTGCGCCCTCCGGTATTCCGGAGGGCATGCCTGTTCGAGCGTCAT

TTCAACCCTCAAGCCTGGCTTGGTGTTGGGGCACTGAAGAGCAGGCCCTGAAATATAGTG

GCGAGCTCGCCAGGACTCCGAGCGTAGTAGACCCTCGCTAAGG-CCTGGCGGTGCCCTGC

TGAAA???????????????????????????????????????????????????????

????????????????????????????????????????????????????????????

??????????????????????????????????????????NNNNNNNNNNNNNNNNNN

NNNNNNNNNNNNNNNNNNNNNNNNNNNNNNNNNNNNNNNNNNNNNNNNNNNNNNNNNNNN

NNNNNNNNNNNNNNNNNNNNNNNNNNNNNNNNNNNNNNNNNNNNNNNNNNNNNNNNNNNN

NNNNCGCCACCCATAGCACGTTTCCCAGGGTAACCAAATCGGTGCTGCTTTCTGGTGCGT

CC-AAGTCCACCACCGCGATACGACCTCGAAGCATACTGACCTCGCAGGCAAACCATCTC

TGGCGAGCACGGTCTCGACAGCAATGGCGTGTATGCACCTCCTATTTCTGTCTTTCTCGT

-TGCCCTGACAGTCACACAGTTACAACGGCACTTCCGAGCTCCAGCTCGAGCGCATGAAC

GTCTACTTCAACGAGGTAAGTACGTTG--TTGACCATCTACAGCTTGGCTAACGCGTTAC

CAGGCCTCCGGCAACAAGTATGTGCCCCGCGCCGTCCTCGTCGATCTCGAGCCCGGTACC

ATGGACGCCGTC

>Diaporthe_citri_CBS_135422

CTTTGTAATCTTTAGCCGACATGCCCGCCCTTACTCTTGCGCATGCTAACGGACCGTTTT

CGGCTTGTAGGATAAGGATGGCGATGGTTAGTGCGGCCGCTCTCACCCTGCACGCGTCAT

GCCCGATCCGCCGCGACAGCCTGCGCGCAACCAAGCGTTATCACTATCACGAGTTGCTGA

GGTGTAGGACAAATCACCACCAAAGAGCTCGGCACGGTCATGCGATCCCTGGGTCAGAAC

CCGTCCGAGTCTGAGCTGCAAGATATGATTAACGAGGTCGACGCCGACAACAATGGCACC

ATTGACTTTGGTACGTCCAAATGCTCGCCCTTCCGCGCCCTCCACCGGAGGTGTCAAGAA

GCCTCACCGCTACAAGCCTGGTACCGTCGCTCTGCGTGAGATCCGTCGGTACCAGAAGAG

CACCGAGCTGATGATCCCCAAGCTCCCCTTCCAGCGTTTGGTATGCAGGTCCGTGAGATC

GCCCAGGACTTCAAGTCCGACCTCCGCTTCCAGTCTTCCGCCGTCGGTGCCCTGCAGGAG

TCGGTCGAGTCTTACCTCGTCTCCCTCTTCGAGGACACCAACCTGTGCGCCATCCACGCC

AAGCGCGTCACCGTCCAGTCGGTATGTNNNNNNNNNNNNNNNNNNNNNNNTGCGGAGGGA

TCATTGCTGCCCCAGGCGCACCCAGAAACCCTTTGTGAACTCTTACTGTTGCCTCGGCAG

GCCGGGGGGCCCCCCGAGACGGGGAGCAGGCCCGCCGGCGGCCAAGTCAACTCTTGTTTT

TACACTGAAACTCTAAATGAATCAAAACTTTCAACAACGGATCTCTTGGTTCTGGCATCG

ATGAAGAACGCAGCGAAATGCGATAAGTAATGTGAATTGCAGAATTCAGTGAATCATCGA

ATCTTTGAACGCACATTGCGCCCTCTGGTATTCCGGAGGGCATGCCTGTTCGAGCGTCAT

TTCAACCCTCAAGCCTGGCTTGGTGATGGGGCACTGGGGAGCAGGCCCTGAAATCCAGTG

GCGAGCTCGCCAGGACCCCGAGCGCAGTAGACCCTCGCTAAGGCCCTGGCGGTGCCCTGC

TGAAAGAGAAGGAAGGTTAGTAAACACCATCATGCTCGCGCGGC-TCGTCAGCGCATTTT

CACCCCTCGCTTTGGATTTTCAGGGTGCGGGGCTTAGGGCTTATCTCACCACCACCACCG

AATATATGCTGATATCTACACAGCCGCCGAGCTTGGTAAGGGNNNNNNNNNNNNNNNNNN

NNNNNNNNNNNNNNNNNNNNNNNNNNNNNNNNNNNNNNNNNNNNNNNNNNNNNNNNNNNN

NNNNNNNNNNNNNNNNNNNNNNNNNNNNNNNNNNNNNNNNNNNNNNNNNNNNNNNNNNNN

NNNNNNNNNNNNNNNNNNNNNNNNNNNNNNNNNNNNNNNNGGTGCTGCTTTCTGGTGCGT

CCCAAGCCCACCACCACAAGTCGCCCTCGAGGCATACTGACCTCGTAGGCAAACCATCTC

TGGCGAGCACGGCCTCGACAGCAATGGCGTGTATGCACCTCCTATTCCTGCCCATCTTGG

CTTCCCTGACAATTGCACAGTTACAACGGCACTTCTGAGCTCCAGCTCGAGCGCATGAAC

GTCTACTTCAACGAGGTAAGTACAGCATTTTGACCATCTGCAACCTTGCTAACGCGTTAT

CAGGCCTCCGGCAACAAGTATGTTCCTCGCGCCGTCCTCGTCGATCTCGAGCCCGGTACC

ATGGACGCCGTC

>Diaporthe_citriasiana_CBS_134240

CTTCGTAAGTCACCATCGCCTCACCCGCCCTTGCTGTCGCGCATGCTAACGGACCGTTTT

CGGATCGCAGGATAAGGATGGCGATGGTTAGTGCAACCACTTCCAACTAGCACGCGTCAC

GCTTGATCCGCTGCGACGGTCTGCGCGCAAGCGACCGACATCGCCATCACGAGTTACTAA

GGCGTAGGACAAATCACCACCAAGGAACTCGGCACGGTCATGCGGTCCCTGGGTCAAAAC

CCCTCCGAGTCCGAGCTGCAGGATATGATCAATGAGGTCGACGCCGACAACAATGGCACC

ATTGACTTTGGTAAGTCCAGATGCTCGCTTATCCGCGCCCTCCACCGGAGGTGTCAAGGA

GCCTCACCGGCACAAGCGTGGGACCGTGGCTAGGCGTGAGATCCGTCGCTACCAGAAGAG

CACCGAGCTGCTCATCCGCAAGCTCCCCTTCCAGCGTCTGGTATGCAGGTTCGTGAGATC

GCCCAGGACTTCAAGTCCGACCTGCGCTTCCAGTCTTCCGCCATCGGTGCCCTGCAGGAG

TCCGTCGAGTCCTACCTCGTCTCCCTCTTCGAGGACACCAACCTGTGCGCCATCCACGCC

AAGCGTGTCACCATCCAGTCGGTACGTAACAAGGTCTCCGTTGGTGAACCAGCGGAGGGA

TCATTGCTGCCCC-GGCGCACCCAGAAACCCTTTGTGAACTCTA-CTGTTGCCTCGGCAG

GCCGGGAGGCCCCCCGGGACGGGGAGCA-GCCCGCCGGCGGCCAAGCCAACTCTTGTTTC

TACAGTGAATCTCTAAATGAATCAAAACTTTCAACAACGGATCTCTTGGTTCTGGCATCG

ATGAAGAACGCAGCGAAATGCGATAAGTAATGTGAATTGCAGAATTCAGTGAATCATCGA

ATCTTTGAACGCACATTGCGCCCTCTGGTATTCCGGAGGGCATGCCTGTTCGAGCGTCAT

TTCAACCCTCAAGCCTGGCTTGGTGCTGGGGCACTGAGGAGCAGGCCCTGAAATCTAGTG

GCGAGCTCGCCAGGACCCCGAGCGCAGTAGA-TCTCGTTAAGGCCCTGGCGGTGCCCTGC

TGAAAGAGAAGGAAGGTTAGTAAACATCATTGCATCTGCGCGGCTTCGTCAAGGCATTTT

CACCCCTCGCTCTGGATTTTCAGGGTGCGGGGCTTACGGCTTATCTCGCTGCCACCTCCG

GAATTATGCTGATTTCTACACAGCCGCCGAGCTTGGTAAGGGNNNNNNNNNNNNNNNNNN

NNNNNNNNNNNNNNNNNNNNNNNNNNNNNNNNNNNNNNNNNNNNNNNNNNNNNNNNNNNN

NNNNNNNNNNNNNNNNNNNNNNNNNNNNNNNNNNNNNNNNNNNNNNNNNNNNNNNNNNNN

NNNNNNNNNNNNNNNNNNNNNNNNNNNNGGTAACCAAATCGGTGCTGCTTTCTGGTGCGT

CCCAAGCCTACCACCGCGACACTAGCTCGCAACATACTGACCTCGTAGGCAAACCATCTC

TGGCGAGCACGGTCTCGACAGCAATGGCGTGTATGCACCTCCTATTCCTGCCCATCTCGT

CCTCCCTGACAATTGCACAGTTACAACGGCACTTCCGAGCTCCAGCTCGAGCGCATGAAC

GTCTACTTCAACGAGGTAAGTACGTCGTTTTGTCCATCTACGGTCTTGCTAACGCGTCAT

CAGGCCTCCGGCAACAAGTATGTGCCTCGCGCCGTCCTCGTCGATCTCGAGCCCGGTACC

ATGGACGCCGTC

>Diaporthe_citrichinensis_CBS_134242

CTTTGTAATCTCCAGCCGACATGCCCGCCCTTGCTGGTGCGCATGCTAACGGACCGTTTT

CGGCTTGTAGGATAAGGATGGCGATGGTTAGTGCGGCCGCTCTCACCTAGCACGCGTCTT

GCTCGAACCGCCGCGACAGCCTGCGCGCAACCACGCGTTATCACTATCACGAGTTGCTGA

GGTGTAGGACAAATCACCACCAAGGAGCTCGGCACGGTCATGCGGTCCCTTGGTCAGAAC

CCGTCCGAGTCTGAGCTGCAAGATATGATTAACGAGGTCGACGCCGACAACAATGGCACC

ATTGACTTTGGTACGTCCAGATGCTCGCCCTTCCGCGCCCTCCACCGGAGGTGTCAAGAA

GCCTCACCGCTACAAGCCTGGTACCGTCGCTCTGCGTGAGATTCGTCGCTACCAGAAGAG

CACTGAGCTGCTGATCCGCAAGCTCCCCTTCCAGCGTCTGGTATGCAGGTCCGTGAGATC

GCCCAGGACTTCAAGTCCGACCTCCGCTTCCAGTCTTCCGCCATCGGCGCCCTGCAGGAG

TCGGTCGAGTCTTACCTCGTCTCCCTCTTCGAGGACACCAACCTGTGCGCCATCCACGCC

AAGCGTGTCACCATCCAGTCGGTATGTAACAAGGTCTCCGTTGGTGAACCAGCGGAGGGA

TCATTGCTGCCCCAGGCGCACCCAGAAACCCTTTGTGAACTTTTACTGTTGCCTCGGAAG

GCTGGGGGGCCCCTCCGGGTGTTGAGACAGCCCGCCGGCGGCCAAGTTAACTCTTGTTTT

TACACTGAAACTCTAAATGAATCAAAACTTTCAACAACGGATCTCTTGGTTCTGGCATCG

ATGAAGAACGCAGCGAAATGCGATAAGTAATGTGAATTGCAGAATTCAGTGAATCATCGA

ATCTTTGAACGCACATTGCGCCCTCTGGTATTCCGGAGGGCATGCCTGTTCGAGCGTCAT

TTCAACCCTCAAGCCTGGCTTGGTGATGGGGCACTGAGAAGCAGGCCCTGAAATTCAGTG

GCGAGCTCGCCAGGACCCCGAGCGCAGTAGACCCTCGCTAAGGCCCTGGCGGTGCCCTGC

TGAAAGAGAAGGAAGGTTAGTAAACACCATGATGCTCTTGTGGCTTCGTCAGCGCATTTT

CACCCCTCGCTTTGGATTTTCAGGGTGCGGGGCTTAGGGCTTATCTCACCACCACTACCG

AACATATGCTGATATCTACACAGCCGCCGAGCTTGGTAAGGGNNNNNNNNNNNNNNNNNN

NNNNNNNNNNNNNNCCTGGGACACCCAGATAAGACGCGTCGATTGCTAACGTGTTTTTCT

TGCCTGCAGGTTCACCTTCAGACCGGCCAATGCGTAAGTTGCTGTCAC-AGCGGACCTTA

TCATCGCCACCTGTAGCACGTTTCCCAGGGTAACCAAATCGGTGCTGCTTTCTGGTGCGT

CCCAAGTCCACCGCCGCGATACGACCTCGAAACATGCTGACCTCATAGGCAAACCATCTC

TGGCGAGCACGGCCTCGACAGCAATGGCGTGTATGCACCTCCTATTCCTGCCCATCTTGG

TTTCCCTGACAATTGCACAGTTACAACGGCACTTCTGAGCTCCAGCTCGAGCGCATGAAC

GTCTACTTCAACGAGGTAAGTACATCATTCCGACCGTCTCCAACCTTGCTAACGCGTTAT

CAGGCCTCCGGCAACAAGTATGTTCCTCGCGCCGTCCTAGTCGATCTCGAGCCCCGTNNN

NNNNNNNNNNNN

>Diaporthe_clematidina_MFLUCC_17_2060

NNNNNNNNNNNNNNNNNNNNNNNNNNNNNCTTGCTGTCGCGCATGCTAACGGACCGTTTT

CGGCCTGCAGGATAAGGATGGCGATGGTTAGTGCGGCCGCTCTCACCTAGCACGCGTCAT

GCTCGATCCGCCGCGACGGCCTGCGCGCAACCAGGCGATACCATTTTCACGAGTTGCTGA

GGTGTAGGACAAATCACCACCAAGGAGCTCGGCACGGTCATGCGGTCCCTAGGCCAGAAC

CCGTCCGAGTCTGAGCTGCAAGACATGATCAACGAGGTCGACGCCGACAACAATGGCACC

ATCGACTTTGGTACGTCCGGATGCTCGCCCA?????????????????????????????

????????????????????????????????????????????????????????????

????????????????????????????????????????????????????????????

????????????????????????????????????????????????????????????

????????????????????????????????????????????????????????????

???????????????????????????NNNNNNNNNNNNNNNNNNNNNNNNNNNNNNNNN

NNNNNNNNNCCCCAGGCGCACCCAGAAACCCTTTGTGAACTT-TATTGTTGCCTCGGCTA

GCTGGGGCCCCTCACCCGGTGAGGAGCG-GCGCGCCGGCGGCCAACCAAACTCTGTTTCT

T--AGTGAATCTCTAAATGAATCAAAACTTTCAACAACGGATCTCTTGGTTCTGGCATCG

ATGAAGAACGCAGCGAAATGCGATAAGTAATGTGAATTGCAGAATTCAGTGAATCATCGA

ATCTTTGAACGCACATTGCGCCCTCTGGTATTCCGGAGGGCATGCCTGTTCGAGCGTCAT

TTCAACCCTCAAGCCTGGCTTGGTGTTGGGGCGCTGAGAAGCAGGCCCTGAAATTCAGTG

GCGAGCTCGCCAGGACTCCGAGCGCAGTAG-ATCTCGTTAAGG-CCTGGCGGTGCCCTGC

TGAAANNNNNNNNNNNNNNNNNNNNNNCATTATGGTTGCGACTGTCCGTTGGCGCATTTT

CACCCCTCGCTCTGGAATTTCAGGGTGCGGGGCTTAGGGCTTATCTCACCACCATAATCA

CAACCATGCTAATATCCCCACAGCCGCCGAGCTTGGTAAGGGNNNNNNNNNNNNNNNNNN

NNNNNNNNNNNNNNNNNNNNNNNNNNNNNNNNNNNNNNNNNNNNNNNNNNNNNNNNNNNN

NNNNNNNNNNNNNNNNNNNNNNNNNNNNNNNNNNNNNNNNNNNNNNNNNNNNNNNNNNNN

NNNNNNNNNNNNNNNNNNNNNNNNNNNNNNNNNNNNNNNNNNNNNNNNNNNNNNNNNNNN

NNNNNNNNNNNCACCGCGGTACGGCCTCGAAGCATACTGACCTCGCAGGCAAACCATCTC

TGGCGAGCACGGCCTCGACAGCAATGGCGTGTATGTACCTCCTTTTCCTGCCCATCTCGT

CCTCCTTGACAGTCGCACAGTTACAACGGCACTTCCGAGCTCCAGCTCGAGCGCATGAAC

GTCTACTTCAACGAGGTATGTACGCCGTTCTGACCATCCTCAGCCTCGCTAACGCGTTCT

CAGGCCTCCGGCAACAAGTATGTGCCTCGCGCCGTCCTCGTCGATCTCGAGCCCGGTACC

ATGGACGCCGTC

>Diaporthe_collariana_MFLUCC_17_2636

CTTTGTAATCTCTAGCCGACATGCCCGCCCTTGCTGGTGCGCATGCTAACGGACCGTTTT

CGGCTTGTAGGATAAGGATGGCGATGGTTAGTGCGGCCGCTTTCACCTAGCACGCGTCAT

GTTCGATCCACCGCGACGGTCTGCGCGCAGCCAAGCG-TATCACTATCACGAGTTGCTGA

GGTGTAGGGCAAATCACTACCAAGGAGCTCGGCACGGTCATGCGATCCCTGGGTCAGAAC

CCGTCTGAGTTTGAGCTGCAAGATATGATTAACGAGGTCGACGCCGACAACAATGGCACC

ATTGACTTTGGTACGTCCGGATGCTCGCGTT?????????????????????????????

????????????????????????????????????????????????????????????

????????????????????????????????????????????????????????????

????????????????????????????????????????????????????????????

????????????????????????????????????????????????????????????

???????????????????????????AACAAGGTCTCCGTTGGTGAACCAGCGGAGGGA

TCATTGCTGCCCCAGGCGCACCCAGAAACCCTTTGTGAACTTTTACTGTTGCCTCGGCTA

GCTGGGGGGCCCCTCTTCTGGAGGAGCAGGCACGCCGGCGGCCAACCCAACTCTTGTTTT

TACACTGAGACTCTAAATGAATCAAAACTTTCAACAACGGATCTCTTGGTTCTGGCATCG

ATGAAGAACGCAGCGAAATGCGATAAGTAATGTGAATTGCAGAATTCAGTGAATCATCGA

ATCTTTGAACGCACATTGCGCCCTCTGGTATTCCGGAGGGCATGCCTGTTCGAGCGTCAT

TTCAACCCTCAAGCCTGGCTTGGTGATGGGGCACTGAAGGGCAGGCCCTGAAATTCAGTG

GCGAGCTCGCCAGGACCCCGAGCGCAGTAGACCCTCGCTAAGGCCCTGGCGGTGCCCTGC

GAAAAGAGAAGGAAGGTTAGTAAATACCATCATGCTCGCGCGGCTTCGTCACCGCATTTT

CACCCCTCGCTTTGGATTTTCAGGGTGCGGGGCTTAGGGCTTATCTCACCACTACTACTG

AATATATGCTGATATCTACACAGCCGCCGAGCTTGGTAAGGGNNNNNNNNNNNNNNNNNN

NNNNNNNNNNNNNNNNNNNNNNNNNNNNNNNNNNNNNNNNNNNNNNNNNNNNNNNNNNNN

NNNNNNNNNNNNNNNNNNNNNNNNNNNNNNNNNNNNNNNNNNNNNNNNNNNNNNNNNNNN

NNNNNNNNNNNNNNNNNNNNNNNNNNNNNNNNNNNNNNNNNACGGTGCTTTCTGGTGCGT

CCCAAGTCCACCGCCGCGATACGACCTCGAAGCATACTGACCTCGCAGGCAAACCATCTC

TGGCGAGCACGGCCTCGACAGCAATGGCGTGTATGCACCTCCTATTCCTGCCCATCTTGG

CTTCCCTGACAATTGCACAGTTACAACGGCACTTCTGAGCTGCAGCTCGAGCGCATGAAC

GTCTACTTCAACGAGGTAAGTACATCATTCCGACCATCTCCAACCTCGCTAACGTGTTAT

CAGGCCTCCGGCAACAAGTATGTTCCTCGCGCCGTCCTCGTCGATCTCGAGCCCGGTACC

ATGGACGCCGTC

>Diaporthe_compacta_LC3083

????????????????????????????????????????????????????????????

????????????????????????????????????????????????????????????

????????????????????????????????????????????????????????????

????????????????????????????????????????????????????????????

????????????????????????????????????????????????????????????

???????????????????????????????TCCGCGCCCTCCACCGGAGGTGTCAAGAA

GCCTCACCGCTACAAGCCTGGTACCGTCGCTCTGCGTGAGATCCGTCGCTACCAGAAGAG

CACCGAGCTGCTCATCCGCAAGCTCCCCTTCCAGCGTCTGGTATGCAGGTCCGTGAGATC

GCCCAGGACTTCAAGTCCGACCTGCGCTTCCAGTCTTCCGCCATCGGTGCCCTTCAGGAG

TCCGTCGAGTCTTACCTCGTCTCCCTCTTTGAGGACACCAACCTGTGCGCCATCCACGCC

AAGCGTGTCACCATCCAGTCGGTACGTNNNNNNNNNNNNNNNNNNNNNNNNNNNNNGGGA

TCATTGCTGCCTC-GGCGCACCCAGAAACCCTTTGTGAACTTA--CTGTTGCCTCGGCAG

GCCGGCAGACCCCCTGAGACAGGGAGCA-GCCCGCCGGCGGCCAACCAAACTC-TGTTTC

TATAGTGAATCTCTAAATGAATCAAAACTTTCAACAACGGATCTCTTGGTTCTGGCATCG

ATGAAGAACGCAGCGAAATGCGATAAGTAATGTGAATTGCAGAATTCAGTGAATCATCGA

ATCTTTGAACGCACATTGCGCCCTCTGGTATTCCGGAGGGCATGCCTGTTCGAGCGTCAT

TTCAACCCTCAAGCCTGGCTTGGTGATGGGGCAGTGCAAGGCACGCCCTGAAATTCAGTG

GCGAGCTCGCCAGGACCCCGAGCGTAGTAGA-TCTCGCTAAGGCCCTGGCGGTGCCCTGC

TGAAAGAGAAGGAAGGTCAGTAAACATCATCGCGTCTGCGCCGCTTCGT--GAGCATTTT

CACCCCTCGTTCTGGACTTTCAGGGTGCGGGGCTTACCGCTTATCGCGCTGCCCACATTG

AAAAAATGCTGATTTCCATACAGCCGCCGNNNNNNNNNNNNNNNNNNNNNNNNNNNNNNN

NNNNNNNNNNNNNNNNNNNNNNNNNNNNNNNNNNNNNNNNNNNNNNNNNNNNNNNNNNNN

NNNNNNNNNNNNNNNNNNNNNNNNNNNNNNNNNNNNNNNNNNNNNNNNNNNNNNNNNNNN

NNNNNNNNNNNNNNNNNNNNNNNNNNNNNNNNNNNNNNNNNNNNNNNNNNNNNNNNNNNN

NNNNNNNNNNNNNNNNNNNNNNNAGCTCGCAACATACTGACCTCGTAGGCAAACCATCTC

TGGCGAGCACGGCCTCGACAGCAATGGCGTGTATGCACCTCCTATTCCTGCCCATCCCGT

CCTCCCTGATGATTGCACAGTTACAACGGCACTTCCGAGCTCCAGCTCGAGCGCATGAAC

GTCTACTTCAACGAGGTATGTACGTCGTTTTGCCCATCTACTGCCTTGCTAACGCGTTGT

CAGGCTTCCGGCAACAAGTATGTGCCCCGCGCTGTCCTCGTCGATCTCGAGCCCGGTACC

ATGGACGCCGTC

>Diaporthe_conica_CFCC_52571

NNNNNNNNNNNNNNNNNNNNNNNNCCGCCCTTGCTGGTGCGCATGCTAACGGACCGTTTT

CGGCTTGTAGGATAAAGATGGCGATGGTTAGTGCGGCCGCTCTCACCTAGCACGCGTCAT

GCTCGATCCGCCGCGACAGCCTGCGCGCAACCAAGCGTTATCACTATCACGAGTTGCTGA

GGTGTAGGACAAATCACCACCAAGGAGCTCGGCACGGTCATGCGGTCCCTTGGTCAGAAC

CCGTCCGAGTCTGAGCTGCAAGATATGATTAACGAGGTCGACGCCGACAACAATGGCACC

ATTGACTTTGGTACGTCCAGACGCTCGCCCTTCCGCGCCCTCCACCGGAGGTGTCAAGAA

GCCTCACCGCTACAAGCCCGGTACCGTCGCTCTGCGTGAGATCCGTCGCTACCAGAAGAG

CACTGAGCTGCTGATCCGCAAGCTCCCCTTCCAGCGTCTGGTATGCAGGTCCGTGAGATC

GCCCAGGACTTCAAGTCCGACCTTCGCTTCCAGTCTTCCGCCATTGGCGCCCTGCAGGAG

TCGGTCGAGTCTTACCTCGTCTCCCTCTTCGAGGACACCAACCTGTGCGCCATCCACGCC

AAGCGTGTCACCATCCAGTCGGTATGTNNNNNNNNNNNNNNNNNNNNNNNNNNNNNNNNN

NNNNNNNNNNNNNNNNNNNNNNNNNNNNCCCTTTGTGAACTTTTACTGTTGCCTCGGAAG

GCTGGGGGGCCCCTCCGGGTGTTGAGACAGCCCGCCGGCGGCCAAGCCAACTCTTGTTTT

TACACTGAAACTCTAAATGAATCAAAACTTTCAACAACGGATCTCTTGGTTCTGGCATCG

ATGAAGAACGCAGCGAAATGCGATAAGTAATGTGAATTGCAGAATTCAGTGAATCATCGA

ATCTTTGAACGCACATTGCGCCCTCTGGTATTCCGGAGGGCATGCCTGTTCGAGCGTCAT

TTCAACCCTCAAGCCTGGCTTGGTGATGGGGCACTGGGGAGCAGGCCCTGAAATTCAGTG

GCGAGCTCGCCAGGACCCCGAGCGCAGTAGACCCTCGCTAAGGCCCTGGCGGTGCCCTGC

TGAAAGAGAAGGAAGGTTAGTAAACACCATCATCCTCGTGTGGCTTCGTCAGCGCATTTT

CACCCCTCGTCCTGGACTTTCAGGGTGCGGGGCTTAGGGCTTATCTCACCACCACTACCG

AACATATGCTGATATCTACACAGCCGCCGAGCTTGGTAAGGGTCGCACCTGAGCCCCACC

ATCCCGACCCACCCC--------CCCAGATAAGACGCGTCGATTGCTAACGTGTTTTTCT

CGCCTACAGGTTCACCTTCAGACCGGCCAATGCGTAAGTCGCTGTCAC-AGCGGACCTTA

TCATCGCCACCTGTAGCACGTTTCCCAGGGTAACCAAATCGGTGCTGCTTTCTGGTGCGT

CCCAAGTCACACACCGTGA------CTCAAGGCATGCTGACCTCGTAGGCAAACCATCTC

TGGCGAGCACGGCCTCGACAGCAATGGCGTGTATGTATCTCCTGTTCCTGCCCATCTTGG

CTACCCTGACAATTGCACAGTTATAACGGCACTTCTGAGCTCCAGCTCGAGCGCATGAAC

GTCTACTTCAACGAGGTAAGTACAGCATTTTGGCCATCTGCAACCATGCTAACGCGTTTT

CAGGCCTCCGGCAACAAGTATGTTCCTCGCGCCGTCCTCGTCGATCTCGAGCCCGGTACC

ATGGACGCCGNN

>Diaporthe_constrictospora_CGMCC_3_20096

NNNNNNNNNNNNNNNNNNNNNNNNNNNGCCTTGCTATTGCGCATGCTAACGGACTGTTCT

CGGACCACAGGACAAGGACGGCGATGGTTAGTGTGGCTGCCTCGACTCCTGACGCTTTAC

CCACGATCCGCCGCGACAGCTTGCGCACGATCACGCGTCGTTACTGTCATGGCTTGCTAA

GGTGCAGGACAAATCACCACCAAGGAGCTCGGAACTGTCATGCGATCGCTTGGTCAGAAC

CCCTCCGAGTCTGAGTTGCAAGATATGATCAACGAGGTCGACGCCGACAATAATGGCACC

ATTGACTTTGGTACGTGCAGACCCCTGCTCATCCGCGCCCTCCACCGGAGGTGTCAAGAA

GCCTCACCGCTACAAGCCTGGTACCGTCGCTCTGCGTGAGATCCGTCGCTACCAGAAGAG

CACCGAGCTGCTGATCCGCAAGCTCCCCTTCCAGCGTCTGGTAAGCAGGTTCGTGAGATC

GCCCAGGACTTCAAGTCCGACCTGCGCTTCCAGTCTTCCGCCATCGGTGCCCTGCAGGAG

TCCGTCGAGTCTTACCTCGTCTCCCTGTTCGAGGACACCAACCTGTGCGCCATCCACGCC

AAGCGTGTCACCATCCAGTCGGTATGTAACAAGGTCTCCGTTGGTGAACCAGCGGAGGGA

TCATTGCTGCCCCTGGCGCACCCAGACACCCTTTGTGAACCTCCATCGTTGCCTCGGCAG

GCCGGGAGGGCCCCCCGAGAGGGGAGCA-GCCCGCCGGTGGCC--CCAAACTCTTGTTTT

TACAGTGTACCTCTAAATGAATCAAAACTTTCAACAACGGATCTCTTGGTTCTGGCATCG

ATGAAGAACGCAGCGAAATGCGATAAGTAATGTGAATTGCAGAATTCAGTGAATCATCGA

ATCTTTGAACGCACATTGCGCCCTCTGGTATTCCGGAGGGCATGCCTGTTCGAGCGTCAT

TTCAACCCTCAAGCCCGGCTTGGTGCTGGGGCGCTGAGGAGCAGGCCCTGAAATTCAGTG

GCGGGCCCGCCAGGACTCCGAGCGCAGTAGAATCTCGTTACCGTCC-GGCGCGGCCCTGC

NNNNN???????????????????????????????????????????????????????

????????????????????????????????????????????????????????????

??????????????????????????????????????????NNNNNNNNNNNNNNNNNN

NNNNNNNNNNNNNNNNNNNNNNNNNNNNNNNNNNNNNNNNNNNNNNNNNNNNNNNNNNNN

NNNNNNNNNNNNNNNNNNNNNNNNNNNNNNNNNNNNNNNNNNNNNNNNNNNNNNNNNNNN

NNNNNNNNNNNNNNNNNNNNNNNNNNNNNNNNNNNNNNNNNNNNNNNNNNNNNNNNNNNN

NNNNTGACACCTACAGTTAGACAACCTCACGACATACTGACCTCGTAGGCAAACCATCTC

CGGCGAGCACGGCCTCGACAGCAATGGCGTGTACGTACCTCCCACCCATCACCATACCAC

TGCTCCTGACGATCGCACAGCTACAACGGCAGTTCTGAACTCCAGCTGGAGCGCATGAAT

GTCTACTTCAATGAGGCAAGTGTACCA--CAGGATCGGTCCATCTTTGCTAACGCGTCTT

TAGGCTTCTGGCGACAAGTATGTTCCCCGCGCCGTCCTCGTAGATCTCGAGCCCGGTACC

ATGGACGCCGTC

>Diaporthe_convolvuli_CBS_124654

CTTTGTAAGTTATCTCCGCCTTGCCCGCCATTACTGTTGCGCATGCTAACGGACCGTTTT

CGACCTGCAGGATAAGGATGGCGATGGTTAGTGCGGTCACTCTCAGCTAGCACGCGTCGT

ACTCGATCCGCCGCGACGGTCTGCGCGCGAGCGACCGTCATCAATATCAC----TGCTAA

GGTGTAGGACAAATCACCACCAAGGAGCTCGGCACAGTCATGCGGTCGCTTGGTCAAAAC

CCTTCCGAGTCCGAGCTGCAGGACATGATCAACGAGGTCGACGCTGACAACAACGGCACC

ATTGACTTTGGTAAGTCTCTCAACTGTCACATCCGCGCCCTCCACCGGAGGTGTCAAGAA

GCCTCACCGCTACAAGCCTGGTACCGTCGCTCTGCGTGAGATCCGTCGCTACCAGAAGAG

CACCGAGCTGCTGATCCGCAAGCTCCCCTTCCAGCGTCTGGTATGCAGGTCCGTGAGATC

GCCCAGGACTTCAAGTCCGACCTGCGCTTCCAGTCTTCCGCCATCGGTGCTCTTCAGGAG

TCCGTCGAGTCTTACCTCGTCTCTCTCTTCGAGGACACCAACCTGTGCGCCATCCATGCC

AAGCGTGTCACCATCCAGTCGGTACGTAACAAGGTCTCCGTTGGTGAACCAGCGGAGGGA

TCATTGCTGCCTC-GGCGCACCCAGAAACCCTTTGTGAACTTATACTGTTGCCTCGGCAG

GCCGGGAGGCCCCCTGAGACAGGGAGCA-GCCCGCCGGCGGCCAACCAAACTCTTGTTTC

TACAGTGGATCTCTAAATGAATCAAAACTTTCAACAACGGATCTCTTGGTTCTGGCATCG

ATGAAGAACGCAGCGAAATGCGATAAGTAATGTGAATTGCAGAATTCAGTGAATCATCGA

ATCTTTGAACGCACATTGCGCCCTCTGGTATTCCGGAGGGCATGCCTGTTCGAGCGTCAT

TTCAACCCTCAAGCCTGGCTTGGTGATGGGGCGCTGACGGGCAGGCCCTGAAATCTAGTG

GCGAGCTCGCCAGGACCCCGAGCGTAGTAGA-TCTCGCTAAGGCCCTGGCGGTGCCCTGC

TGAAAGAGAAGGAAGGTTAGTAAACATCGTTGTGTCTGCGAGGCTTCGTCAAGCCATTTT

CACCCCTCCCTCTGGATTTTCAGGGTGCGGGGCTTACCGCTTATCTCAGCTAC-ACAC-G

AAAACATGCTGATTTCTACACAGCCGCCGAGCTGGGTAAGGGTCA--CATCAGCCCCACC

ATCGCGACCCACCCCCTGCGACACCCAGATAACACGCGTCGATTGCTAACGTGTTTTCCT

CGCCCACAGGTTCACCTTCAGACCGGCCAATGCGTAAGTTGTCGTTACCGCCAGACCTTA

T---CGCCACCCGTAGCACGTTTCCCAGGGTAACCAAATCGGTGCTGCTTTCTGGTGCGT

CCCAAACCTACCACCGCGAGGCGAGCTCCAAGCATGCTGACCTCGTAGGCAAACCATCTC

TGGCGAGCACGGTCTCGACAGCAATGGCGTGTATGCACCCCCTATTCCTGCCCACC----

TCTCCCTGATGATCGCACAGTTACAACGGCACTTCCGAGCTCCAGCTCGAGCGCATGAAC

GTCTACTTCAACGAGGTATGTACGTCGTTTTGA-CCCTCTCGCCATTGCTAACGCGTCTT

CAGGCTTCCGGCAACAAGTATGTGCCCCGCGCCGTCCTCGTCGATCTCGAGCCCGGTACC

ATGGACGCCGTC

>Diaporthe_coryli_CFCC_53083

CTTTGTAATCTCCAGCCGACACGCCCGCCCTTGCTGGTGCGCATGCTAACGGACCGTTTT

CGGCTTGTAGGATAAGGATGGCGATGGTTAGTGCGGCCGCTCTCACCTAGCACGCGTCTT

GCTCGAACCGCCGCGACAGCCTGCGCGCAACCACGCGTTATCACTATCACGAGTTGCTGA

GGTGTAGGACAAATCACCACCAAGGAGCTCGGCACGGTCATGCGGTCCCTTGGTCAGAAC

CCGTCCGAGTCTGAGCTGCAAGATATGATTAACGAGGTCGACGCCGACAACAATGGCACC

ATTGACTTTGGTACGTCCAGATGCTCGCCCTTCCGCGCCCTCCACCGGAGGTGTCAAGAA

GCCTCACCGCTACAAGCCTGGTACCGTCGCTCTGCGTGAGATCCGTCGCTACCAGAAGAG

CACTGAGCTGCTGATCCGCAAGCTCCCCTTCCAGCGTCTGGTATGCAGGTCCGTGAGATC

GCCCAGGACTTCAAGTCCGACCTCCGCTTCCAGTCTTCCGCCATCGGCGCCCTGCAGGAG

TCGGTCGAGTCTTACCTCGTCTCCCTCTTCGAGGACACCAACCTGTGCGCCATCCACGCC

AAGCGTGTCACCATCCAGTCGGTATGTNNNNNNNNNNNNNNNNNNNNNNNNNNNNNNNNN

NNNNNNNNNNNNNNNNNNNNNNNNNNNNCCCTTTGTGAACTTTTACTGTTGCCTCGGAAG

GCTGGGGGGCCCCTCCGGGTGTTGAGACAGCCCGCCGGCGGCCAAGTTAACTCTTGTTTT

TACACTGAAACTCTAAATGAATCAAAACTTTCAACAACGGATCTCTTGGTTCTGGCATCG

ATGAAGAACGCAGCGAAATGCGATAAGTAATGTGAATTGCAGAATTCAGTGAATCATCGA

ATCTTTGAACGCACATTGCGCCCTCTGGTATTCCGGAGGGCATGCCTGTTCGAGCGTCAT

TTCAACCCTCAAGCCTGGCTTGGTGATGGGGCACTGAAGAGCAGGCCCTGAAATTCAGTG

GCGAGCTCGCCAGGACCCCGAGCGCAGTAGACCCTCGCTAAGGCCCTGGCGGTGNNNNNN

NNNNNNNNNNNNAAGGTTAGTAAACACCATGATGCTCGTGTGGCTTCGTCAGCGCATTTT

CACCCCTCGCTTTGGATTTTCAGGGTGCGGGGCTTAGGGCTTATCTCACCACCACTACCG

AACATATGCTGATATCTACACAGCCGCCGAGCTTGGTAANNNTCGCACCTGGGCCCCACC

ATCGCGACCCACCCCCTGGGACACCCAGATAAGACGCGTCGATTGCTAACGTGTTTTTCT

CGCCTGCAGGTTCACCTTCAGACCGGCCAATGCGTAAGTTGCTGTCAC-AGCGGACCTTA

TCATCGCCACCTGTAGCACGTTTCCCAGGGTAACCAAATCGGTGCTGCTTTCTGGTGCGT

CCCAATTCCACCGCCGCGATACGACCTCGAAACATGCTGACCTCGTAGGCAAACCATCTC

TGGCGAGCACGGCCTCGACAGCAATGGCGTGTATGCACCTCCTATTCCTGCCCATCTTGG

TTTCCCTGACAATTGCACAGTTACAACGGCACTTCTGAGCTCCAGCTCGAGCGCATGAAC

GTCTACTTCAACGAGGTAAGTACATCATTCCAACCGTCTCCAACCTTGCTAACGCGTTAT

CAGGCCTCCGGCAACAAGTATGTTCCTCGCGCCGTCCTCGTCGATCTCGAGCCCGGTACC

ATGGACGCCGTN

>Diaporthe_corylicola_CFCC_53986

NNNNNNNNNNNNNNNNNNNNNNNNNNGCCCTTGCTGTTGCGCATGCTAACGGAGAGTTTT

GGGCTTGTAGGATAAGGATGGCGATGGTTAGTGCGGCCGCTCTCACCCAGCACGCGTCAC

GCTCGATCCACCGCGACGGCCTGCGCGCAGCCAAGCGACATTACTATCACGACTTGCTGA

GGTGTAGGACAAATCACCACCAAGGAGCTCGGCACGGTCATGCGGTCCCTGGGTCAGAAC

CCGTCCGAGTCTGAGCTACAAGATATGATTAACGAGGTCGACGCCGACAACAACGGCACC

ATTGACTTTGGTACGTCCAGATGTTCGCCTATCCGCGCCCTCCACCGGAGGTGTCAAGAA

GCCTCACCGCTACAAGCCTGGTACCGTCGCTCTGCGTGAGATCCGTCGCTACCAGAAGAG

CACCGAGCTGTTGATCCGCAAGCTCCCCTTCCAGCGTCTGGTATGCAGGTCCGTGAGATC

GCCCAGGACTTCAAGTCCGACCTCCGCTTCCAGTCTTCTGCCATCGGTGCCCTGCAGGAG

TCCGTCGAGTCTTACCTCGTCTCACTCTTCGAGGACACCAACCTGTGCGCCATCCACGCC

AAGCGTGTCACCATCCAGTCGGTAGGTNNNNNNNNNNNNNNNNNNNNNNNNNNNNNNNNN

TCATTGCTGCCCCAGGCGCACCCAGAAACCCTTTGTGAACTTTTACTGTTGCCTCGGCAG

GCCGGGGGGCCCCTCGAGACGAGGAGCAGGCCCGCCGGCGGCCAAGTTAACTCTCATTTT

TACACTGAAACTCTAAATGAATCAAAACTTTCAACAACGGATCTCTTGGTTCTGGCATCG

ATGAAGAACGCAGCGAAATGCGATAAGTAATGTGAATTGCAGAATTCAGTGAATCATCGA

ATCTTTGAACGCACATTGCGCCCTCTGGTATTCCGGAGGGCATGCCTGTTCGAGCGTCAT

TTCAACCCTCAAGCCTGGCTTGGTGATGGGGCACTGAAGGGCAGGCCCTGAAATTCAGTG

GCGAGCTCGCCAGGACCCCGAGCGCAGTAGACCCTCGCTAAGGCCCTGGCGGTGCCCTGC

TG-AANNNNNNNNNNNNNNNNNNNNNNCAGCATGCTCACGCGGCTTCGTCAGCGCATTTT

CACCCCTCGCTCTGGATTTTCAGGGTGCGGGGCTTAGAGCTTATCTCACCACCACATCGG

AAAACATGCTGACATCTACACAGCCGCCGAGCTTGGCAANNNNNNNNNNNNNNNNNNNNN

NNNNNNNNNNNNNNNNNNNNNNNNNNNNNNNNNNNNNNNNNNNNNNNNNNNNNNNNNNNN

NNNNNNNNNNNNNNNNNNNNNNNNNNNNNNNNNNNNNNNNNNNNNNNNNNNNNNNNNNNN

NNNNNNNNNNNNNNNNNNNNNNNNNNNNNNNNNNNNNNNNNNTGCTGCTTTCTGGTGCGT

CCAAAACCCACCGCGGCGAGTCGACCTCGAAGCATACTGACCTCGTAGGCAAACTATCTC

TGGCGAGCACGGCCTCGACAGCAATGGCGTGTATGCATCTCCTATTGCTACCTTTCTCGT

-TGCCCTGACAATCACACAGTTACAACGGCAGTTCTGAGCTCCAGCTCGAGCGCATGAAC

GTCTACTTCAACGAGGTAAGTACGTCG--TTGACCATCTACAGCTTTTCTAACGCGTCAT

CAGGCCTCCGGCAACAAGTATGTTCCTCGCGCCGTCCTCGTCGATCTCGAGCCCGGTACC

ATGGACGCCGTN

>Diaporthe_crataegi_CBS_114435

NNNNNNNNNNNNNNNNNNACATGCCTCCCACTGCTGTTGCGCATGCTAACGGACCGTTTT

CGGCCTGCAGGATAAGGATGGCGATGGTTAGTGCGGCCGCTCACGCCCGCCACGCGTCAC

GCTCGACCCGCCGCGACAGCTCGCGCGCGACCAAGCGTCATCACTATCAGGAGTTGCTGA

AGTGTAGGACAAATCACCACAAAGGAGCTCGGCACGGTCATGCGATCGCTGGGTCAGAAC

CCGTCCGAGTCTGAGCTGCAAGATATGATTAACGAAGTCGACGCCGACAACAATGGCACC

ATTGACTTTGGTACGTCCAGATG-TCGCTCATCCGCGCCCTCCACCGGAGGTGTCAAGAA

GCCTCACCGCTACAAGCCTGGTACCGTCGCTCTACGTGAGATCCGTCGCTACCAGAAGTC

CACCGAGCTGCTGATCCGCAAGCTCCCCTTCCAGCGTCTGGTATGCAGGTTCGCGAGATC

GCCCAGGACTTCAAGTCCGACCTCCGCTTCCAGTCTTCCGCCATCGGTGCCCTGCAGGAG

TCCGTCGAGTCTTACCTCGTCTCCCTCTTTGAGGACACCAACCTGTGCGCCATCCACGCC

AAGCGTGTCACCATCCAGTCGGTATGTAACAAGGTCTCCGTTGGTGAACCAGCGGAGGGA

TCATTGCTGCCCACGGCGCACCCAGAAACCCTTTGTGAACTCTAACTGTTGCCTCGGCAG

GCCGGGGGGCCCCTCGTGACGAGGAGCAGGCCCGCCGGCGGCCAAGCCAACTCTTGTTTT

TAAACCGAGACTCTAAATGAATCAAAACTTTCAACAACGGATCTCTTGGTTCTGGCATCG

ATGAAGAACGCAGCGAAATGCGATAAGTAATGTGAATTGCAGAATTCAGTGAATCATCGA

ATCTTTGAACGCACATTGCGCCCTCTGGTATTCCGGAGGGCATGCCTGTTCGAGCGTCAT

TTCAACCCTCAAGCCTGGCTTGGTGATGGGGCACTGGAGGGCAGGCCCTGAAACTCAGTG

GCGAGCTCGCCAGGACCCCGAGCGCAGTAGACCCTCGCTAAGGCCCTGGCGGTGCCCTGC

TGAAAGAGAAGGAAGGTTAGTAAACATCCTCATGGTTGCGCGGCTTCGTCAGCACGTTTT

GACCCCTCGCTCTGGATTTTCAGGGTGCGGGGCTTAGAGCTTATCTCACCACAACCTCCG

GAATAATGCTAACATTTCCACAGCCGCCGAGCTCGGTAAGGGTCGCACCTGAGCCCCACC

ATCGCGACCCACCCCCTGAGACACCCAGATAAGACGCGTCAATTGCTAACGTGTTTTTCT

CGCCCACAGGTTCACCTCCAGACCGGCCAATGCGTAAGTTGCTGTCACCACCGTACCTTA

T---CGCCACCTGTAGCACGTTTCCCAGGGTAACCAAATCGGTGCTGCTTTCTGGTGCGT

CCCAGCTCTACCACCGCGAGACGGCCTCGCAGCATACTGACCTCGTAGGCAAACCATCTC

TGGCGAGCACGGCCTCGACAGCAATGGCGTGTATGCACC---TATCCGTACCCA-----T

CCTCTCTGACAATTGCACAGTTACAACGGCACTTCCGAGCTCCAGCTCGAGCGCATGAAT

GTCTACTTCAACGAGGTAAGTATATGATTTCGACCATGTCCAGTCTTGCTAACGCATTAT

CAGGCCTCCGGCAACAAGTATGTGCCTCGCGCCGTCCTCGTCGATCTCGAGCCCGGTACC

ATGGACGCCGTC

>Diaporthe_crotalariae_CBS_162_33

CTTTGTA-CCCCCAGCTGACATGCCCGCCGTACCCGTTGCGCATGCTAACGGACCGTTTT

CGGCTTGTAGGATAAGGATGGCGATGGTTAGTCCAACCGGTCCCACCCAATACGCGTCAC

GCTCGATCCGCCGCGACGGCCTGCGCGTTTACAGGCGACGTCGATATCGCGAGTTGCTGA

G--ATAGGACAAATCACCACCAAGGAGCTCGGCACAGTCATGCGATCCCTGGGCCAGAAC

CCCTCCGAGTCCGAGCTGCAAGACATGATTAACGAGGTTGACGCCGACAACAATGGCACC

ATTGACTTTGGTACGTTCAGCTGCTCGCCCATCCGCACCCTCCACCGGAGGTGTCAAGAA

ACCTCACCGCTACAAGCCTGGTACTGTCGCTCTGCGTGAGATCCGTCGCTACCAGAAGAG

CACCGAGCTGCTGATCCGCAAGCTCCCCTTCCAGCGTCTGGTATGCAGGTCCGTGAGATC

GCCCAGGACTTCAAGTCCGACCTCCGCTTCCAGTCTTCCGCCATCGGTGCCCTGCAGGAG

TCTGTCGAGTCTTACCTCGTCTCTCTCTTCGAAGACACCAACCTGTGCGCCATCCACGCC

AAGCGTGTCACCATCCAGTCGGTACGTNACAAGGTCTCCGTTGGTGAACCAGCGGAGGGA

TCATTGCTGCCCCAGGCGCACCCAGAAACCCTTTGTGAACTCTTACTGTTGCCTCGGCTG

GCCGGGGGGCCCCTCGAGACGAGGAGCAGGCCCGCCGGCGGCCAAGCCAACTCTTGTTTT

TACACCGAAACTCTAAATGAATCAAAACTTTCAACAACGGATCTCTTGGTTCTGGCATCG

ATGAAGAACGCAGCGAAATGCGATAAGTAATGTGAATTGCAGAATTCAGTGAATCATCGA

ATCTTTGAACGCACATTGCGCCCTCTGGTATTCCGGAGGGCATGCCTGTTCGAGCGTCAT

TTCAACCCTCAAGCCTGGCTTGGTGTTGGGGCACTGGAGGGCAGGCCCTGAAATCTAGTG

GCGAGCTCGCCAGGACCCCGAGCGCAGTAGACCCTCGCTGAGGCCCTGGCGGTGCCCTGC

TGAAAGAGAAGGAAGGTCAGTAAACATTCCTGCGTTTGCACGGCGTCGTCACCGCATTTT

CACCCCTCGCTCTGGATTTTCAGAGTGCGGGGCTTAGTGCTTATCACACCACCACCACTG

GGAAGATGCTGACATCTCTACAGCCGCTGAGCTCGGTAAGGGTCGCACCTGAGCCCCACC

ATCGCGACTGTCAGCCTGCGACACCTGGATGAGACGCGTCCATTGCTAACCTGTTTTCCT

TGCCTCCAGGTTCACCTCCAGACCGGCCAATGCGTAAGTTGCTTTCACCACCAGACCTCA

TCATCGCCACCCGTAGCACGTTTCGCAGGGTAACCAAATCGGTGCTGCTTTCTGGTGCGT

GCCGAGCCTGCCACCGCGAGACGTCCTAGAAGCTTGCTGACCTCCTAGGCAAACCATTTC

TGGCGAGCACGGTCTCGACAGCAATGGCGTGTATGCACCTCCTATTCCTGCTCTTCTCGC

CCTCCCTGACAATTGCACAGCTACAACGGCACCTCCGAGCTCCAGCTCGAGCGCATGAAC

GTCTACTTCAACGAGGTAAGTTTGTCAT---GACCCCGCCCTTCCTTGCTGACGGATTAT

CAGGCTTCCGGCAACAAATATGTCCCTCGCGCCGTCCTCGTCGATCTCGAGCCCGGTACC

ATGGACGCTGTC

>Diaporthe_crousii_CAA823

NNNNNNNNNNNNNNNNNNNNNNNNNNNNNNNNNNTGTTGCGCATGCTAACGGACCGTATT

CGGCCTCCAGGATAAGGATGGCGATGGTTAGTGCGGCTGCCTCTACCCTAAACGCGTCAC

GATCGATCCGCCGCGACAGCTTGCGCGCGATCAAGCGCCATCACTGCTAAGAGTTGCTGA

GGTGTAGGACAAATCACCACGAAGGAGCTCGGAACGGTCATGCGATCGCTGGGCCAGAAC

CCGTCCGAGTCTGAGCTGCAAGATATGATCAACGAGGTCGATGCCGACAACAATGGCACC

ATCGACTTTGGTACGTTCAGATGTCTGCTGATCCGCGCCCTCCACCGGAGGTGTCAAGAA

GCCTCACCGTTACAAGCCTGGTACCGTCGCTCTGCGTGAGATCCGTCGCTACCAGAAGTC

CACCGAGCTGCTGATCCGCAAGCTCCCCTTCCAGCGTCTGGTATGCAGGTTCGTGAGATC

GCCCAGGACTTCAAGTCCGATCTCCGCTTCCAGTCCTCCGCCATCGGTGCCCTGCAGGAG

TCTGTCGAGTCTTACCTCGTCTCCCTTTTCGAGGACACCAACCTGTGCGCCATCCACGCC

AAGCGTGTCACCATCCAGTCGGTACGTNNNNNNNNNNNNNNNNNNNNNNNNNNNNAGGGA

TCATTGTTGCTCCG-GCGCATCCAGAAACCCTTTGTGAACTTATACTGTTGCCTCGGCAG

GCTGGGGGGTCCCTCTCGGTGAGGAGCAGGCCCGCCGGCGGCCAAGTTAACTCTTGTTTT

TACACTGAAACTCTAAATGAATCAAAACTTTCAACAACGGATCTCTTGGTTCTGGCATCG

ATGAAGAACGCAGCGAAATGCGATAAGTAATGTGAATTGCAGAATTCAGTGAATCATCGA

ATCTTTGAACGCACATTGCGCCCTCTGGTATTCCGGAGGGCATGCCTGTTCGAGCGTCAT

TTCAACCCTCAAGCCTGGCTTGGTGATGGGGCACTGAAGGGCAGGCCCTGAAATTCAGTG

GCGAGCTCGCCAGGACCCCGAGCGCAGTAGACCCTCGCTAAGGTCTTGGTGCGGCCCTGC

TGAAANNNNNNNAAGGTTAGTAAATATCAACATGCTTCTACGGCTGCGTCATCGCATTTT

CACCCCTCGTTCTGGATTTTCAGGGTGCGGGGCTTAGACCTTATCTCACAACCACCACCT

GAAACGTGCTGACGTTCTACCAGCCGCCGAGCTTGGCAAGGGTCGCACCTGAGCCCCACC

ATCGCGACCCACCCCCTGAGACACTCAGATAAGACGCGTCGAATGCTAACTTTTTTTTCT

CGTTCACAGGTTCACCTTCAGACCGGCCAATGCGTAAGTTGCTGTCACCACCGCACCTTA

T---CGCCCCCTGTAGCACGTTTTCCAGGGTAACCAAATCGGTGCTGCTTTCTGGTGCGT

C----GTCCATCATCGCGACACGACCTCGCAACATATTGACTTCGTAGGCAAACCATCTC

TGGCGAGCACGGCCTCGACAGCAATGGCGTGTACGTACCTCCTATTCCTACCCGTCTCGT

CCACCTTGACAGCTTCACAGTTACAACGGCTCTTCTGAGCTCCAGCTCGAGCGTATGAAC

GTCTACTTCAACGAGGTCAGTTTTTTACACCCACGATCTCCAGCTTTGCTGACACCTTAT

CAGGCTTCCGGCAACAAGTATGTGCCTCGCGCTGTCCTCGTCGATCTCGAGCCCGGTACC

ATGGACGCCGTC

>Diaporthe_cucurbitae_DAOM_42078

????????????????????????????????????????????????????????????

????????????????????????????????????????????????????????????

????????????????????????????????????????????????????????????

????????????????????????????????????????????????????????????

????????????????????????????????????????????????????????????

???????????????????????????????TCCGCGCCCTCCACCGGAGGTGTCAAGAA

GCCTCACCGCTACAAGCCTGGTACCGTCGCTCTGCGTGAGATCCGTCGCTACCAGAAGAG

CACCGAGCTGCTCATCCGCAAGCTGCCCTTCCAGCGTCTGGTAAGCAGGTTCGTGAGATC

GCCCAGGACTTCAAGTCCGACCTGCGCTTCCAGTCTTCCGCCATCGGCGCCCTCCAGGAG

TCCGTCGAGTCCTACCTCGTCTCCCTCTTCGAGGACACCAACCTGTGCGCCATCCACGCC

AAGCGTGTCACCATCCAGTCGGTACGTAACAAGGTCTCCGTTGGTGAACCAGCGGAGGGA

TCATTGCTGCCCC-GGCGCACCCAGAAACCCTTTGTGAACTTATACTGTTGCCTCGGCAG

GCCGGGAGGCCCCCTGAGACAGGGAGCA-GCCCGCCGGCGGCCAACCAAACTC-TGTTTC

TATAGTGAATCTCTAAATGAATCAAAACTTTCAACAACGGATCTCTTGGTTCTGGCATCG

ATGAAGAACGCAGCGAAATGCGATAAGTAATGTGAATTGCAGAATTCAGTGAATCATCGA

ATCTTTGAACGCACATTGCGCCCTCTGGTATTCCGGAGGGCATGCCTGTTCGAGCGTCAT

TTCAACCCTCAAGCCTGGCTTGGTGATGGGGCACTGAAGGGCAGGCCCTGAAATCTAGTG

GCGAGCTCGCCAGGACCCCGAGCGTAGTAGA-TCTCGCTAAGGCCCTGGCGGTGCCCTGC

TGAAAGAGAAGGAAGGTCAGTAAACATCATTGTGCCTGCGCGGCTTCGTCAAGCCATTTT

CACCCCTCCCTCTGGGTTTTCAGGGTGCGGGGCTTACCGCTTATCTCACCGTCAACACCG

AACAGACGCTGATTTCCATACAGCCGCCGAGCTGGGTAAGGGNNNNNNNNNNNNNNNNNN

NNNNNNNNNNNNNNNNNNNNNNNNNNNNNNNNNNNNNNNNNNNNNNNNNNNNNNNNNNNN

NNNNNNNNNNNNNNNNNNNNNNNNNNNNNNNNNNNNNNNNNNNNNNNNNNNNNNNNNNNN

NNNNNNNNNNNNNNNNNNNNNNNNNNNNNNNNNNNNNNNNNNNNNNNNNNNNNNGTGCGT

CAAGCCGCCACGGCCGCGAGATTAGCTCGCAACACACTGACCTCGTAGGCAAACCATCTC

TGGCGAGCACGGTCTCGACAGCAATGGCGTGTATGCACCTCCTATTTCTGCCCATCTCGT

CCTCCTTGATGTTTGCACAGTTACAACGGCACTTCTGAGCTTCAGCTCGAGCGCATGAAC

GTCTACTTCAACGAGGTGAGTACGTCTTTTTGAGCGTTCTTGCCTTTACTGACGCGCTTT

CAGGCCTCCGGCAACAAGTATGTGCCTCGCGCCGTCCTCGTCGATCTCGAGCCCGGTACC

ATGGACGCCGTC

>Diaporthe_cuppatea_CBS_117499

NNNNNNNNNNNNNNNNNNNNNNNNNNGCCATTGCCTTTGCGCATGCTAACGGACCGTTTT

CGGCCTGCAGGATAAGGATGGCGATGGTTAGTGCGGTCACTCTCAGCTACCACGCGTCAT

ATTCGATCCGCCGCGACGGTCTGCGCGTGAGCGAGCGACCTCACGATCACGAGATGCTAA

GGTGTAGGACAAATCACCACCAAGGAGCTCGGCACAGTCATGCGGTCCCTGGGTCAAAAC

CCTTCCGAGTCCGAGCTGCAGGACATGATCAACGAGGTCGATGCCGACAACAATGGCACC

ATTGACTTTGGTAAGTCTAGATGCCCACCCGTCCGCGCCCTCCACCGGAGGTGTCAAGAA

GCCTCACCGCTACAAGCCTGGTACCGTCGCTCTGCGTGAGATCCGTCGCTACCAGAAGAG

CACCGAGCTGCTGATCCGCAAGCTCCCCTTCCAGCGTCTGGTAAGCAGGTTCGTGAGATC

GCCCAGGACTTCAAGTCCGACCTGCGCTTCCAGTCTTCTGCCATCGGTGCCCTTCAGGAG

TCCGTCGAGTCTTACCTCGTCTCCCTCTTTGAGGACACCAACCTGTGCGCCATCCACGCC

AAGCGTGTCACCATCCAGTCGGTACGTNNNNNNNNNNNNNNNNNNNNNNNNNNNNNNGGA

TCATTGCTGCTTT-GGCGCACCCAGAAACCCTTTGTGAACTTA--CTGTTGCCTCGGCAG

GCCGGAAGGCCCCCTGAAACAGGGAGCA-GCCCGCCGGCGGCCAACCAAACTC-TGTTTC

TATAGTGAATCTCTAAATGAATCAAAACTTTCAACAACGGATCTCTTGGTTCTGGCATCG

ATGAAGAACGCAGCGAAATGCGATAAGTAATGTGAATTGCAGAATTCAGTGAATCATCGA

ATCTTTGAACGCACATTGCGCCCTCTGGTATTCCGGAGGGCATGCCTGTTCGAGCGTCAT

TTCAACCCTCAAGCCCGGCTTGGTGATGGGGCACTGAAGGGCAGGCCCTGAAATCTAGTG

GCGAGCTCGCCAGGACCCCGAGCGTAGTAGA-TCTCGCTAAGGCCCTGGCGGTGCCCTGC

TGAAANNNNNNNNNNGTCAGTAAACATCATAGTGTCTGCGCGGCTTCGTCAAGCCATTTT

CACCCCTCCCTCTGGGTTTTCAGGGTGCGGGGCTTACCGCTTATCTCGTCGCCAACACCG

AATAGACGCTGATTTCCACACAGCCGCCGAGCTGGGTAAGGGNNNNNNNNNNNNNNNNNN

NNNNNNNNNNNNNNNNNNNNNNNNNNNNNNNNNNNNNNNNNNNNNNNNNNNNNNNNNNNN

NNNNNNNNNNNNNNNNNNNNNNNNNNNNNNNNNNNNNNNNNNNNNNNNNNNNNNNNNNNN

NNNNNNNNNNNNNNNNNNNNNNNNNNNNNNNNNNNNNNNNNNNNNNNNNNNNNNNNNNNN

NNNNNNNNNNNNNNNNNNNNNNNAGCTCGCAATATGCTGACCTCGTAGGCAAACCATCTC

TGGCGAGCACGGTCTCGACAGCAATGGCGTGTATGCACCTCCTATT-CTGTCCTTCTCGT

CCTCCCTGATGTTTGCACAGTTACAACGGCACTTCCGAGCTCCAGCTCGAGCGCATGAAC

GTCTACTTCAACGAGGTGAGTACGTCTTTTGGAGCGTTCTCGACTCTGCTAACGCGCTTT

CAGGCCTCCGGCAACAAGTATGTGCCTCGCGCCGTCCTCGTCGATCTCGAGCCCGGTACC

ATGGACGCCGTC

>Diaporthe_cynaroidis_CBS_122676

CTTTGTAAGTTCTCTTC------CCCGCCGTGGCTGTTGCGCATGCTAACGGACCGTTGT

CGGCCTCCAGGATAAGGATGGCGATGGTTAGTGCGGCTGCCTCTACCCTAAACGCGTCAC

GATCGATCCGCCGCGACAGCTTGCGCGCGATCAAGCGCCGTCACTACCAGGAGTTGCTAA

AGTGTAGGACAAATCACCACGAAGGAGCTCGGGACGGTCATGCGATCGCTGGGCCAGAAC

CCGTCCGAGTCTGAGCTGCAAGATATGATCAACGAGGTCGATGCCGACAACAATGGCACC

ATCGACTTTGGTACGTTCAGATGTCTGCTGATCCGCGCCCTCCACCGGAGGTGTCAAGAA

GCCTCACCGTTACAAGCCCGGTACCGTCGCTCTGCGTGAGATCCGTCGCTACCAGAAGTC

CACTGAGCTGCTGATCCGCAAGCTCCCCTTCCAGCGTCTGGTATGCAGGTTCGTGAGATC

GCCCAGGACTTCAAGTCCGATCTCCGTTTCCAGTCCTCCGCCATCGGTGCCCTGCAGGAG

TCTGTCGAGTCTTACCTCGTCTCCCTGTTCGAGGACACCAACCTGTGCGCCATCCACGCC

AAGCGTGTCACCATCCAGTCGGTACGTAACAAGGTCTCCGTTGGTGAACCAGCGGAGGGA

TCATTGTTGCTCCG-GCGCATCCAGAAACCCTTTGTGAACTTATACTGTTGCCTCGGCAG

GCTGGGGGGTCCCTCTCGGTGAGGAGCAGGCCCGCCGGCGGCCAAGTTAACTCTTGTTTT

TACACTGAAACTCTAAATGAATCAAAACTTTCAACAACGGATCTCTTGGTTCTGGCATCG

ATGAAGAACGCAGCGAAATGCGATAAGTAATGTGAATTGCAGAATTCAGTGAATCATCGA

ATCTTTGAACGCACATTGCGCCCTCTGGTATTCCGGAGGGCATGCCTGTTCGAGCGTCAT

TTCAACCCTCAAGCCTGGCTTGGTGATGGGACACTGAAAGGCAGGTCCTGAAATTCAGTG

GCGAGCTCGCCAGGACCCCGAGCGCAGTAGACCCTCGCTAAGGTCTTGGTGTGGCCCTGC

TGAAAGAGAAGGAAGGTTAGTAAATATCCACATGCTTCTAAGGCTGCGTCATCGCATTTT

CACCCCTCGTTCTGGATTTTCAGGGTGCGGGGCTTAGACCTTATCTCACAACCACC---T

GAAACGTGCTGACGCTTTACCAGCCGCCGAGCTTGGTAAGGGTCGCACCTGAGCCCCACC

ATCGCGACCCACCCCCTGAGACACTCAGATAAGACGCGTCGAATGCTAACTTTTTTTTTT

CGTTCACAGGTTCACCTTCAGACCGGCCAATGCGTAAGTTGCTGTCACCACCGCACCTTA

T---CGCCCCCTGTAGCACGTTTTCCAGGGTAACCAAATCGGTGCTGCCTTCTGGTTCGT

C----CTCTATCATCGCGACACGACCTCGCAACATATTGACTTCGTAGGCAAACCATCTC

TGGCGAGCACGGCCTCGACAGCAATGGCGTGTATGTACCTCCTATTGCTACCCGTCTCGT

CCGCCCTGACAGCCTCACAGTTACAACGGCTCTTCTGAGCTCCAGCTCGAGCGCATGAAC

GTCTACTTCAACGAGGTCAGTTTTTTATACCCACGATCTCCAGCTTTGCTGACACCTTAT

CAGGCTTCCGGCAACAAGTATGTGCCTCGCGCTGTCCTCGTCGATCTCGAGCCCGGTACC

ATGGACGCCGTC

>Diaporthe_cytosporella_CBS_137020

NNNNNNNNNNNNNNNNNNNNNNNNNNNNNNNNNNNNNNNNNNNNNNNNNNNNNNNNNNNN

NNNNNNNNNNNNNNNNNNNNNNNNNNNNNNNNNNNNNNNNNNNNNNNNNNNNNNNNNNNN

NNNNNNNNNNNNNNNNNNNNNNNNNNNNNNNNNNNNNNNNNNNNNNNNNNNNNNNNNNNN

NNNNNNNNNNNNNNNNNNNNCAAGGAGCTCGGCACGGTCATGCGGTCCCTGGGTCAGAAC

CCGTCCGAGTCGGAGCTGCAAGATATGATTAACGAGGTCGACGCCGACAACAACGGCACC

ATTGACTTTGGTACGTTCAGATGCTCGCCTCTCCGCGCCCTCCACCGGAGGTGTCAAGAA

GCCTCACCGCTACAAGCCTGGTACCGTCGCTCTGCGTGAGATCCGTCGTTACCAGAAGAG

CACTGAGCTGCTGATCCGCAAGCTCCCCTTCCAGCGTCTGGTATGCAGGTTCGTGAGATC

GCCCAGGACTTCAAGTCCGACCTCCGCTTCCAGTCCTCCGCCATCGGTGCCCTGCAGGAG

TCCGTCGAGTCTTACCTCGTCTCCCTGTTCGAGGACACCAACCTGTGCGCCATCCACGCC

AAGCGTGTCACCATCCAGTCGGTACGTNNNNNNNNNNNNNNNNNNNNNNNTGCGGAGGGA

TCATTGCTGCCCCTGGCGCACCCAGAAACCCTTTGTGAACTTTTACTGTTGCCTCGGCAG

GCCGTGGGGTCCCTTCTCAAAAGGAGCA-GCCGGCCGGTGGCCAAATTAACTC-TGTTTT

TAAACTGAAACTCTAAATGAATCAAAACTTTCAACAACGGATCTCTTGGTTCTGGCATCG

ATGAAGAACGCAGCGAAATGCGATAAGTAATGTGAATTGCAGAATTCAGTGAATCATCGA

ATCTTTGAACGCACATTGCGCCCTCTGGTATTCCGGAGGGCATGCCTGTTCGAGCGTCAT

TTCAACCCTCAAGCCTGGCTTGGTGTTGGGGCACTGGAGAGCAGGCCCTGAAATATAGTG

GCGAGCTCGCCAGGACTCCGAGCGTAGTAGACCCTCGCTAAGG-CCTGGCGGTGCCCTGC

TGAAAGAGAAGGAAGGTTAGTAAACACCATCATGTTCGTGCGG-TTCGTGGGCGCATTTT

CACCCCGCCCTCTGGATTTTCAGGGTGCGGGGCTTAGAGCTTATCTCACA---------A

AAAACATGCTGACTTCTTCACAGCCGCCGAGCTTGGTAAGGGNNNNNNNNNNNNNNNNNN

NNNNNNNNNNNNNNNNNNNNNNNNNNNNNNNNNNNNNNNNNNNNNNNNNNNNNNNNNNNN

NNNNNNNNNNNNNNNNNNNNNNNNNNNNNNNNNNNNNNNNNNNNNNNNNNNNNNNNNNNN

NNNNNNNNNNNNNNNNNNNNNNNNNNNNNNNNNNNNNNNNGGTGCTGCTTTCTGGTGCGT

CC-AAGTCAACCACCGCGAGTCGACCTCGAAGCATACTGACCTCGCAGGCAAACCATCTC

TGGCGAGCACGGTCTCGACAGCAATGGCGTGTACGCACCTCCTATTCCTGTCTTTCTCGT

-TGCACTAACAATCACACAGTTACAACGGCACTTCCGAGCTCCAGCTCGAGCGCATGAAC

GTCTATTTCAACGAGGTAAGTACGTTG--TT-ACCATCTACAGCTTTGCTAACGCGTTAT

CAGGCCTCCGGCAACAAGTATGTGCCCCGCGCCGTCCTCGTCGATCTCGAGCCCGGTACC

ATGGACGCCGTC

>Diaporthe_decedens_CBS_109772

CTTTGTAATCTCCAGCCGGCACGCCCGCCCTTGCTCTTGCGCATGCTAACGGGGAGTCTT

GGACTTGTAGGATAAGGATGGCGATGGTTAGTGCGGCCGCTCTCACCCAGCACGCGTCAC

GCTCGATCCACCGCGACGGCCTGCGCGCAACCAAGCGACATCGCTATCACGACTTGCTGA

GGTGTAGGACAAATCACCACCAAGGAGCTCGGCACAGTCATGCGGTCCCTGGGTCAGAAC

CCGTCCGAGTCTGAGCTGCAAGATATGATTAACGAGGTCGACGCCGACAACAACGGCACC

ATTGACTTTGGTACGTCCAGATGACCGCCTATCCGCGCCCTCCACCGGAGGTGTCAAGAA

GCCTCACCGCTACAAGCCTGGTACCGTCGCCCTGCGTGAGATCCGTCGCTACCAGAAGAG

CACCGAGCTGCTGATTCGCAAGCTCCCCTTCCAGCGTCTGGTATGCAGGTTCGTGAGATC

GCCCAGGACTTCAAGTCCGACCTCCGCTTCCAGTCTTCCGCCATCGGTGCCCTGCAGGAG

TCCGTCGAGTCTTACCTCGTCTCCCTCTTTGAGGACACCAACCTGTGCGCCATCCACGCC

AAGCGTGTCACCATCCAGTCTGTATGTAACAAGGTCTCCGTTGGTGAACCAGCGGAGGGA

TCATTGCTGCCCCAGGCGCACCCAGAAACCCTTTGTGAACTTTTACTGTTGCCTCGGCAG

GCCGGGGGGCCCCTCGAGACGAGGAGCAGGCCCGCCGGCGGCCAAGTTAACTCTTGTTTT

TACACTGGCACTCTAAATGAATCAAAACTTTCAACAACGGATCTCTTGGTTCTGGCATCG

ATGAAGAACGCAGCGAAATGCGATAAGTAATGTGAATTGCAGAATTCAGTGAATCATCGA

ATCTTTGAACGCACATTGCGCCCTCTGGTATTCCGGAGGGCATGCCTGTTCGAGCGTCAT

TTCAACCCTCAAGCCTGGCTTGGTGATGGGGCACTGAAGGGCAGGCCCTGAAATTCAGTG

GCGAGCTCGCCAGGACCCCGAGCGCAGTAGACCCTCGCTAAGGCCCTGGCGGTGCCCTGC

TGAAAGAGAAGGAAGGTTAGTAAACAACACCATGCTCTCGCGGCTTCGTCAGCGCATTTT

CACCCCTCGCTCTGGATTTTCAGGGTGCGGGGCTTAGAGCTTATCT---TGCCCCCACCG

AAAACATGCTGACATCTACATAGCCGCCGAGCTTGGTAAGGGTCGCACCTGAGCCCCACC

ATCGCGACCCACCCCCTGGGACACCCAGATAAGACGCGTCGATTGCTAACATGTTTTTTC

TGCCTATAGGTTCACCTCCAGACCGGCCAATGCGTAAGTTGCTGTCACAGCGGA-TCTTA

TCGTCGCCACCTGTAGCACGTTTCCCAGGGTAACCAAATCGGTGCTGCTTTCTGGTGCGT

CCAAACCCCACCGCGGCAAGTCGACCTTACAGCATACTGACCTCGTAGGCAAACCATCTC

TGGCGAGCACGGCCTCGACAGCAATGGCGTGTATGCATCTCCTATTTCTGCCTTTCTCGT

-TGCCCTGACAATCACACAGTTACAACGGCACTTCCGAGCTCCAGCTCGAGCGCATGAAC

GTCTACTTCAACGAGGTAAGTACGTCG--TTGACCATCTACAGCTTCTCTAACGCGTCAT

CAGGCCTCCGGCAACAAGTATGTTCCTCGCGCCGTCCTTGTCGATCTCGAGCCCGGTACC

ATGGACGCCGTC

>Diaporthe_delonicis_MFLU_16_1059

????????????????????????????????????????????????????????????

????????????????????????????????????????????????????????????

????????????????????????????????????????????????????????????

????????????????????????????????????????????????????????????

????????????????????????????????????????????????????????????

????????????????????????????????????????????????????????????

????????????????????????????????????????????????????????????

????????????????????????????????????????????????????????????

????????????????????????????????????????????????????????????

????????????????????????????????????????????????????????????

???????????????????????????NNNNNNNNNNNNNNNNNNNNNNNNNCCGGAGGA

TCATTGCTGCCCTAGGCGCACCCAGAAACCCTTTGTGAACTTTTACTGTTGCCTCGGCAT

GCTGGGGGGTCCCCTGATACAGGGAGCAGGCACGCCGGCGGCCAAGTTAACTCTTGTTTT

TTCACTGAAACTCTAAATGAATCAAAACTTTCAACAACGGATCTCTTGGTTCTGGCATCG

ATTAAAAACGCAACGAAATGCGATAAGTAATGTGAATTGCATAATTCAGTGAATCATCGA

ATCTTTGAACGCACATTGCGCCCTCTGGTATTCCGGAGGGCATGCCTGTTCGAGCGTCAT

TTCAACCCTCAAGCATTGCTTGGTGTTGGGGCACTGCCAAGCAGGCCCTGAAATCTAGTG

GCGAGCTCGCCAGGACCCCGAGCGCAGTAGACCCTCGCTAAGGCCCTGGCGGTGCCCTGC

TGAAA???????????????????????????????????????????????????????

????????????????????????????????????????????????????????????

??????????????????????????????????????????NNNNNNNNNNNNNNNNNN

NNNNNNNNNNNNNNNNNNNNNNNNNNNNNNNNNNNNNNNNNNNNNNNNNNNNNNNNNNNN

NNNNNNNNNNNNNNNNNNNNNNNNNNNNNNNNNNNNNNNNNNNNNNNNNNNNNNNNNNNN

NNNNNNNNNNNNNNNNNNNNNNNNNNNNNNNNNCCAAAACGGTGCTGCTTTCTGGTGC--

-----GTCCACCACTGCGATACGACCTCGAGCCATACTGACCTCGCAGGCAAACCATCTC

TGGCGAGCACGGCCTCGACAGCAATGGCGTGTATGCACCTCCTATTTCTGCCTTTCTCGT

CTGCCCTGACAATCATACAGTTACAACGGCACTTCCGAGCTCCAGCTCGAGCGCATGAAC

GTCTACTTCAACGAGGTCAGTAAGTCG--TTGACCATCTGCACCTTTGCTAACGCGTTAT

CAGGCCTCCGGCAACAAGTATGTCCCTCGCGCCGTCCTCGTCGATCTCGAGCCCGGTACC

ATGGACGCCGTC

>Diaporthe_detrusa_CBS_109770

CTTTGTAAGTCATA-TCCACATGCCCGCCCTTGCTGTTGCGCATGCTAACGGACCGTTTT

CGGCCTGCAGGATAAGGATGGCGATGGTTAGTGTGGCCGCACCCACGGAGCACGCGTCAT

CCTCGATCCGCCGCGACGGCCTGCGCGCGAACAGGCGTCAGCACTGTCAGGGTTTGCTAA

GGCGTAGGACAAATCACCACGAAGGAGCTCGGCACTGTCATGCGGTCTCTGGGTCAGAAC

CCATCTGAGTCTGAACTGCAAGATATGATTAATGAGGTCGATGCCGACAACAATGGCACC

ATTGACTTTGGTACGTCAGAACGCTCGCCCATCCGCGCCCTCCACCGGAGGTGTCAAGAA

GCCTCACCGCTACAAGCCTGGTACCGTCGCTCTGCGTGAGATCCGTCGTTACCAGAAGAG

CACCGAGCTGCTGATCCGCAAGCTCCCCTTCCAGCGTCTGGTATGCAGGTCCGTGAGATC

GCCCAGGACTTCAAGTCCGACCTCCGCTTCCAGTCTTCCGCCATCGGTGCCCTGCAGGAG

TCCGTGGAGTCTTACCTCGTCTCTCTCTTCGAGGACACCAACCTGTGTGCCATCCACGCC

AAGCGTGTCACCATCCAGTCGGTATGTAACAAGGTCTCCGTTGGTGAACCAGCGGAGGGA

TCATTGCTGCCCCCGGCGCACCCAGAAACCCTTTGTGAACTTTTACTGTTGCCTCGGCAG

GCCGGGGGGCCCCTCGAGACGAGGAGCAGGCCCGCCGGCGGCCAAGTTAACTCTTGTTTT

TACACTGAAACTCTAAATGAATCAAAACTTTCAACAACGGATCTCTTGGTTCTGGCATCG

ATGAAGAACGCAGCGAAATGCGATAAGTAATGTGAATTGCAGAATTCAGTGAATCATCGA

ATCTTTGAACGCACATTGCGCCCTCTGGTATTCCGGAGGGCATGCCTGTTCGAGCGTCAT

TTCAACCCTCAAGCCTGGCTTGGTGATGGGGCACTGAGAAGCAGGCCCTGAAATTCAGTG

GCGAGCTCGCCAGGACCCCGAGCGCAGTAGACCCTCGCTAAGGCCCTGGCGGTGCCCTGC

TGAAAGAGAAGGAAGGTTAGTAAACATCATCGCTCCTGCGCAGCTCCGTCAGCGCATTTT

CACCCCTCGTTCTGGATTTTCAGGGTGCGGGGCTTAGAGCTTATCTCACTACCACC---G

GAAAGATGCTAACAACTATACAGCCGCCGAGCTTGGTAAGGGTCGCACCTGAGCCCCACC

ATCGCGACCCACCCCCTGGGACACCCAGGTAAGACGCGTCGATTGCTAACGTGTTTTTCT

CCCCCGCAGGTTCACCTTCAGACCGGCCAATGCGTAAGTTGCTGTCACACCGGACCCTTG

T---CGCCACCTGTAGCACGTTTCCCAGGGTAACCAAATCGGTGCTGCTTTCTGGTGCGT

CCCAAACTCACCACCGCGATACGACCTCGCGGCATACTGACCTCGTAGGCAAACCATCTC

TGGCGAGCACGGCCTCGACAGCAATGGCGTGTATGCACCTCCTGTTCCTGCACGTCTCGC

CATCCCTGACAATTGCACAGTTACAACGGCACTTCCGAGCTCCAGCTCGAGCGCATGAAC

GTCTACTTCAACGAGGTATGTACATTGTTTCGACCGTCTCCAACCTCGCTAACGCGTTAT

CAGGCTTCCGGCAACAAGTATGTTCCTCGCGCGGTCCTCGTCGATCTCGAGCCCGGCACC

ATGGACGCCGTC

>Diaporthe_diospyricola_CBS_136552

????????????????????????????????????????????????????????????

????????????????????????????????????????????????????????????

????????????????????????????????????????????????????????????

????????????????????????????????????????????????????????????

????????????????????????????????????????????????????????????

????????????????????????????????????????????????????????????

????????????????????????????????????????????????????????????

????????????????????????????????????????????????????????????

????????????????????????????????????????????????????????????

????????????????????????????????????????????????????????????

???????????????????????????AACAAGGTCTCCGTTGGTGAACCAGCGGAGGGA

TCATTGCTGCCCCAGGCGCACCCAGAAACCCTTTGTGAACTTTTACTGTTGCCTCGGCAG

GCCGGGGGGTCCCTCCTCAAGAGGAGCAGGCCCGCCGGCGGCCAATTTAACTC-TGTTTT

TAAACTGAAACTCTAAATGAATCAAAACTTTCAACAACGGATCTCTTGGTTCTGGCATCG

ATGAAGAACGCAGCGAAATGCGATAAGTAATGTGAATTGCAGAATTCAGTGAATCATCGA

ATCTTTGAACGCACATTGCGCCCTCTGGTATTCCGGAGGGCATGCCTGTTCGAGCGTCAT

TTCAACCCTCAAGCCTGGCTTGGTGTTGGGGCACTGAAAAGCAGGCCCTGAAATATAGTG

GCGGGCTCGCCAGGACTCCGAGCGTAGTAGACCCTCGCTAAGG-CCTGGCGGTGCCCTGC

TGAAA???????????????????????????????????????????????????????

????????????????????????????????????????????????????????????

????????????????????????????????????????????????????????????

????????????????????????????????????????????????????????????

????????????????????????????????????????????????????????????

????????????????????????????????????????????????????????????

????????????????????????????????????????????????????????????

????????????????????????????????????????????????????????????

????????????????????????????????????????????????????????????

????????????????????????????????????????????????????????????

????????????????????????????????????????????????????????????

????????????

>Diaporthe_discoidispora_ICMP20662

????????????????????????????????????????????????????????????

????????????????????????????????????????????????????????????

????????????????????????????????????????????????????????????

????????????????????????????????????????????????????????????

????????????????????????????????????????????????????????????

???????????????????????????????TCCGCGCCCTCCACCGGAGGTGTCAAGAA

GCCTCACCGCTACAAGCCTGGCACCGTCGCTCTGCGTGAGATCCGTCGCTACCAGAAGAG

CACCGAGCTGCTGATCCGCAAGCTCCCCTTCCAGCGTCTGGTATGCAGGTTCGTGAGATC

GCCCAGGACTTCAAGTCCGACCTGCGCTTCCAGTCTTCCGCCATCGGTGCCCTTCAGGAG

TCCGTCGAGTCCTACCTCGTCTCCCTCTTTGAGGACACCAACCTGTGCGCCATCCACGCC

AAGCGTGTCACCATCCAGTCGGTACGTNNNNNNNNNNNNNNNNNNNNNNNNNNNNNNNNN

NNNNNNCTGCCTC-GGCGCACCCAGAAACCCTTTGTGAACTTTA-CTGTTGCCTCGGCAG

GCCGGGAGGCCCCCCGAGACGGGGAGCA-GCCCGCCGGCGGCCAAGTTAACTCTTGTTTC

TACCATGAATCTCTAAATGAATCAAAACTTTCAACAACGGATCTCTTGGTTCTGGCATCG

ATGAAGAACGCAGCGAAATGCGATAAGTAATGTGAATTGCAGAATTCAGTGAATCATCGA

ATCTTTGAACGCACATTGCGCCCTCTGGTATTCCGGAGGGCATGCCTGTTCGAGCGTCAT

TTCAACCCTCAAGCCTGGCTTGGTGTTGGGGCACTGAGAAGCAGGCCCTGAAATCTAGTG

GCGAGCTCGCTAGGACCCCGAGCGTAGTAGA-TCTCGTTAAGGCCCTGGCGGTGCCCTGC

TGAAAGAGAAGGAAGGTTAGTAAACATCATCGATTCTGCGCGGCTTCGTAAAGGCATTTT

CACCCCTCGCTCTGGATTTTCAGGGTGCGGGGCTTACGGCTTATCTCGTTGCTACTACCG

GAACTAAGCTGACTTCTACACAGCCGCCGAGCTTGGTAAGGGNNNNNNNNNNNNNNNNNN

NNNNNNNNNNNNNNNNNNNNNNNNNNNNNNNNNNNNNNNNNNNNNNNNNNNNNNNNNNNN

NNNNNNNNNNNNNNNNNNNNNNNNNNNNNNNNNNNNNNNNNNNNNNNNNNNNNNNNNNNN

NNNNNNNNNNNNNNNNNNNNNNNNNNNNNNNNNNNNNNNNNNNNNNNNNNNNNNNNNNNN

NNNNNNNNNNNNNNNNNNNNNNNAGCTCGCAACATACTGACCTAGTAGGCAAACCATCTC

TGGCGAGCACGGTCTCGACAGCAATGGCGTGTATGCAACTCCTATTCCTGCCCATATCGT

CCTCCCTGACAATTGCACAGTTACAACGGCACTTCCGAGCTCCAGCTCGAGCGCATGAAC

GTCTACTTCAACGAGGTAAGTACGTCGTTTTGTCCATCTACAGTCTTGCTAACGCGTTAC

CAGGCCTCCGGCAACAAGTATGTGCCTCGCGCCGTCCTCGTCGATCTCGAGCCCGGTACC

ATGGACGCCGTC

>Diaporthe_dorycnii_MFLUCC_17_1015

????????????????????????????????????????????????????????????

????????????????????????????????????????????????????????????

????????????????????????????????????????????????????????????

????????????????????????????????????????????????????????????

????????????????????????????????????????????????????????????

????????????????????????????????????????????????????????????

????????????????????????????????????????????????????????????

????????????????????????????????????????????????????????????

????????????????????????????????????????????????????????????

????????????????????????????????????????????????????????????

???????????????????????????NNNNNNNNNNNNNNNNNNNNNNNNNNNNNNNNN

NNNNNNNNNNNNNNNNNNNNNNCAGAAACCCTTTGTGAACTCTTACTGTTGCCTCGGCAG

GCCGGGGGGTCCCTCCTCAGGAGGAGCA-GCCGGCCGGTGGCCAATCCAACTC-TGTTTT

TACACTGAAACTCTAAATGAATCAAAACTTTCAACAACGGATCTCTTGGTTCTGGCATCG

ATGAAGAACGCAGCGAAATGCGATAAGTAATGTGAATTGCAGAATTCAGTGAATCATCGA

ATCTTTGAACGCACATTGCGCCCTCTGGTATTCCGGAGGGCATGCCTGTTCGAGCGTCAT

TTCAACCCTCAAGCCTGGCTTGGTGCTGGGGCACTGGGGAGCAGGCCCTGAAATACAGTG

GCGAGCTCGCCAGGACTCCGAGCGCAGTAGACCCTCGCTAAGG-CCTGGCGGTGCCCTGC

TGAAANNNNNNNNNNNNNNNNNNNNNNCATCGTGGTCGTGCGGCTTCGTCGGCTCATTTT

CACCCCGCCCACTGGATCTTCAGGGTGCGGGGCTTAGAGCTTATCTCATAACTGCGTCCA

AAAACATGCTGACTTCTTTACAGCCGCCGAGCTTGGTAAGGGNNNNNNNNNNNNNNNNNN

NNNNNNNNN-NNNNNNNNNNNNNNNNNNNNNNNNNNNNNNNNNNNNNNNNNNNNNNNNNN

NNNNNNNNNNNNNNNNNNNNNNNNNNNNNNNNNNNNNNNNNNNNNNNNNNNNNNNNNNNN

NNNNNNNNNNNNNNNNNNNNNNNNNNNNNNNNNNNNNNNNNNNNNNNNNNNNNNNNNNNN

NNNNNNNNNNNNACCGCGAGTCGACCTCGAAGCATACTGACCTCGCAGGCAAACCATCTC

TGGCGAGCACGGTCTCGACAGCAATGGCGTGTATGTACCTCCTATTCCTGCCTTTCTCGT

-TGCTCTGACAATCACACAGTTACAACGGCACTTCCGAGCTTCAGCTCGAGCGCATGAAC

GTCTACTTCAACGAGGTAAGTACGTTG--TT-ACCATCTACAGCTTTGCTAACGCGTTAT

CAGGCCTCCGGCAACAAGTATGTGCCCCGCGCCGTCCTCGTCGATCTCGAGCCCGGTACC

ATGGACGCCGTC

>Diaporthe_drenthii_BRIP_66524

????????????????????????????????????????????????????????????

????????????????????????????????????????????????????????????

????????????????????????????????????????????????????????????

????????????????????????????????????????????????????????????

????????????????????????????????????????????????????????????

????????????????????????????????????????????????????????????

????????????????????????????????????????????????????????????

????????????????????????????????????????????????????????????

????????????????????????????????????????????????????????????

????????????????????????????????????????????????????????????

???????????????????????????AACAAGGTCTCCGTTGGTGAACCAGCGGAGGGA

TCATTGCTGCCCTAGGCGCACCCAGAAACCCTTTGTGAACTTTTACTGTTGCCTCGGCAT

GCTGGGGGGTCCCCTGAGACAGGGAGCAGGCACGCCGGCGGCCAAGTTAACTCTTGTTTT

TACACTGAAACTCTAAATGAATCAAAACTTTCAACAACGGATCTCTTGGTTCTGGCATCG

ATGAAGAACGCAGCGAAATGCGATAAGTAATGTGAATTGCAGAATTCAGTGAATCATCGA

ATCTTTGAACGCACATTGCGCCCTCTGGTATTCCGGAGGGCATGCCTGTTCGAGCGTCAT

TTCAACCCTCAAGCATTGCTTGGTGTTGGGGCACTGAAGGGCAGGCCCTGAAATCTAGTG

GCGAGCTCGCCAGGACCCCGAGCGTAGTAGACCCTCGCTAAGGCCCTGGCGGTGCCCTGC

TGAAAGAGAAGGAAGGTTAGTAAACACCATCATGTTGGTACAGCTTCGTCAGCGCATTTT

CACCCCTCGCTCTGGATTTTCAGGGTGCGGGGCTTAGAGCTTATCT-----CTACCGCCG

GAAACATGCTGATATCTACACAGCCGCCGAGCTTGGCAAGGGNNNNNNNTGAGCCCCACC

ATCGCGACCCACCCCCTGGGCCACCCAGATAAAACGCGTCAATTGCTAACGTGTTTTTCT

CGACCACAGGTTCACCTTCAGACCGGCCAATGCGTAAGTTGCTGTCACGACGGA-CCTTA

TCATCCCCACCCGTAGCACCTTTCCCAGGGTAACCAAATCGGTGCTGCTTTCTGGTGC--

-----GTCCACCACCGCGTTACGACCTCTAACCATACTGACCTCGCAGGCAAACCATCTC

TGGCGAGCACGGCCTCAACAGCAATGGCGTGTATGCACCTCCTATTCCTGCCTATCTCGT

CAGCCCTGACAATCATACAGTTACAACGGCACTTCCGAGCTCCAGCTCGAGCGCATGAAC

GTCTACTTCAACGAGGTAAGTACGTCG--TTGGCCATCTGCAGCTTTGCTAACGCGTTAT

CAGGCCTCCGGCAACAAGTATGTCCCTCGCGCCGTCCTCGTCGATCTCGAGCCCGGTACC

ATGGACGCCGTC

>Diaporthe_durionigena_VTCC_930005

????????????????????????????????????????????????????????????

????????????????????????????????????????????????????????????

????????????????????????????????????????????????????????????

????????????????????????????????????????????????????????????

????????????????????????????????????????????????????????????

????????????????????????????????????????????????????????????

????????????????????????????????????????????????????????????

????????????????????????????????????????????????????????????

????????????????????????????????????????????????????????????

????????????????????????????????????????????????????????????

???????????????????????????NNNNNNNNNNNNNNNNNNNNNNNNNNNNNNNNN

NNNNNNNNNNNNNNNNNNNNNNNNNNNNCCCTTTGTGAACTTAT-CTGTTGCCTCGGTAG

GCCGGGAGGCCCCCTGAAACAGGGAGCA-GCCCGCCGGCGGCCAACTAAACTCTTGTTTC

TATAGTGAATCTCTAAATGAATCAAAACTTTCAACAACGGATCTCTTGGTTCTGGCATCG

ATGAAGAACGCAGCGAAATGCGATAAGTAATGTGAATTGCAGAATTCAGTGAATCATCGA

ATCTTTGAACGCACATTGCGCCCTCTGGTATTCCGGAGGGCATGCCTGTTCGAGCGTCAT

TTCAACCCTCAAGCCTGGCTTGGTGATGGGGCACTGGAGGGCAGGCCCTGAAATCTAGTG

GCGAGCTCGCTAGGACCCCGAGCGTAGTAGA-TCTCGTTAAGGCCCTGGCGGTGCCCTGC

TGAAANNNNNNNNNNNNNNNNNNNNNNCGTTGTGTCTGCGACGCTTCGTCAAGGCATTTT

CACCCCTCCCTCTGGATTTTCAGGGTGCGGGGCTTACCGCTTATCTCAGTGAC-ACACCG

AAAACATGCTGATTTCTACACAGCCGCCGAGCTGGGTAAGGGNNNNNNNNNNNNNNNNNN

NNNNNNNNNNNNNNNNNNNNNNNNNNNNNNNNNNNNNNNNNNNNNNNNNNNNNNNNNNNN

NNNNNNNNNNNNNNNNNNNNNNNNNNNNNNNNNNNNNNNNNNNNNNNNNNNNNNNNNNNN

NNNNNNNNNNNNNNNNNNNNNNNNNNNNNNNNNNNNNNNCGGTGCTGCTTTCTGGTGCGT

CCCAAGCCTGCCACCGCGAGGCGAGCTCGAAGCATACTGACCTCGTAGGCAAACCATCTC

TGGCGAGCACGGTCTCGACAGCAATGGCGTGTATGCACCTCCTATTCCTGCCCGCCTCGT

TCTCCCTGATGATCGCACAGTTACAACGGCACTTCCGAGCTCCAGCTCGAGCGCATGAAC

GTCTACTTCAACGAGGTCAGTACGTCGTTTTGA-ACCTCTCGCCCCTGCTAACGCGTTTT

CAGGCCTCCGGCAACAAGTATGTGCCCCGCGCCGTCCTCGTCGATCTCGAGCCCGGTACC

ATGGACGCCGTC

>Diaporthe_elaeagni_glabrae_CGMCC_3_18287

NNNNNNNNNNNNNNNNNNNNNNNNNNNNNNNTGCTGTTGCGCATGCTAACGGACCGTTTT

CGGCTTGCAGGATAAGGATGGCGATGGTTAGTGCGGCCGGTCCCACCCACCACGTGTCAC

GCTCGATCCCCCGCGACGGCCTGCGCGCAAACAAGCCTTACCGCTATCACGAGTTGCTGA

GGTGTAGGACAAATCACCACCAAGGAGCTCGGCACAGTCATGCGGTCCCTGGGTCAGAAT

CCGTCCGAGTCTGAGCTGCAAGATATGATTAACGAGGTCGACGCCGACAACAATGGCACC

ATTGACTTTGGTATGTCTAGATACTTGCTTGTCCGCGCCCTCCACCGGAGGTGTCAAGAA

GCCTCACCGCTACAAGCCTGGTACCGTCGCTCTGCGTGAGATCCGTCGTTACCAGAAGAG

CACCGAGCTGCTGATCCGCAAGCTCCCCTTCCAGCGTCTGGTAAGCAGGTTCGCGAGATC

GCCCAGGACTTCAAGTCCGACCTCCGCTTCCAGTCCTCCGCCATCGGTGCCCTGCAGGAG

TCCGTCGAGTCTTACCTCGTCTCCCTGTTCGAGGACACCAACCTGTGCGCCATTCACGCC

AAGCGTGTCACCATCCAGTCGGTATGTNNNNNNNNNNNNNNNNNNNNNNNNNNNNAGGGA

TCATTGCTGCCCCAGGCGCACCCAGAAACCCTTTGTGAACTTTTACTGTTGCCTCGGCAG

GCCGTGGGGTCCCTCGAGACGAGGAGCA-GCCGGCCGGTGGCCAAGTTAACTC-TGTTTT

TACACTGAAACTCTAAATGAATCAAAACTTTCAACAACGGATCTCTTGGTTCTGGCATCG

ATGAAGAACGCAGCGAAATGCGATAAGTAATGTGAATTGCAGAATTCAGTGAATCATCGA

ATCTTTGAACGCACATTGCGCCCTCTGGTATTCCGGAGGGCATGCCTGTTCGAGCGTCAT

TTCAACCCTCAAGCCTGGCTTGGTGATGGGGCACTGAAGAGCATGCCCTGAAATACAGTG

GCGAGCTCGCCAGGACTCCGAGCGCAGTAGACCCTCGCTAAGG-CCTGGCGGTGCCCTGC

TGAAAGAGAAGGAAGGTTAGTAAGCATCATCATGTTGGTA-GGCTTGGTCGGCGCATTTT

CACCCCTCCCTCTGGATTTTCAGGGTGCGGGGCTTAGAGCTTATCTCACC---------G

AAAACATGCTAACATCTATACAGCCGCCGAGCTTGGTAAGGGNNNNNNNNNNNNNNNNNN

NNNNNNNNNNNNNNNNNNNNNNNNNNNNNNNNNNNNNNNNNNNNNNNNNNNNNNNNNNNN

NNNNNNNNNNNNNNNNNNNNNNNNNNNNNNNNNNNNNNNNNNNNNNNNNNNNNNNNNNNN

NNNNNNNNNNNNNNNNNNNNNNNNNNNNNNNNNNNNNNNNGGTGCTGCTTTCTGGTGCGT

CC-AAGTCCACCACCGCGAT-CGACCTTGAAGCATACTGACCTCCTAGGCAAACCATCTC

TGGCGAGCACGGTCTCGACAGCAATGGCGTGTATGCACCTCCTATTTCTGTCTTTATCGT

-TGCCCTGACAATCACACAGTTACAACGGCACTTCCGAGCTCCAGCTCGAGCGCATGAAC

GTCTACTTCAACGAGGTAAGTACGTCG--TTGACCATCTGCGTCTTTGCTGACGCGTTAT

CAGGCCTCCGGCAACAAGTATGTGCCCCGCGCCGTCCTCGTCGATCTCGAGCCCGGTACC

ATGGACGCCGTC

>Diaporthe_eleagni_CBS_504_72

CTTTGTAATCTCTAGTCGACATGCCCGCC--TGCTGTTGCCCATGCTAACGGACCTTTTT

TGGCTTGCAGGATAAGGATGGCGATGGTTAGTGCGGCCGGTCCCACCCACCACGCGTCAC

GCTAGAGCT-CCGCGACGGCCTGCGCGCAAACAAGCGTTACCGCTATCACGAGTTGCTGA

GGTGTAGGACAAATCACCACCAAGGAGCTCGGCACAGTCATGCGGTCCCTGGGTCAGAAC

CCGTCTGAGTCTGAGCTGCAAGATATGATTAACGAGGTCGACGCCGACAACAATGGCACC

ATTGACTTTGGTATGTCTAGAGACTCGCCTGTCCGCGCCCTCCACCGGAGGTGTCAAGAA

GCCTCACCGCTACAAGCCTGGTACCGTCGCTCTGCGTGAGATCCGTCGTTACCAGAAGAG

CACCGAGCTGCTGATCCGCAAGCTCCCCTTCCAGCGTCTGGTATGCAGGTTCGTGAGATC

GCCCAGGACTTCAAGTCCGACCTCCGCTTCCAGTCCTCCGCCATCGGTGCCCTGCAGGAG

TCCGTCGAGTCTTACCTCGTCTCCCTGTTCGAGGACACCAACCTGTGCGCCATCCACGCC

AAGCGTGTCACCATCCAGTCGGTACGTAACAAGGTCTCCGTTGGTGAACCAGCGGAGGGA

TCATTGCTGCCCCTGGCGCACCCAGAAACCCTTTGTGAACTTTTACTGTTGCCTCGGCAG

GCCGGGGGGTCCCTCCTCAGGAGGAGCA-GCCGGCCGGTGGCCAAGTTAACTC-TGTTTT

TACACTGAAACTCTAAATGAATCAAAACTTTCAACAACGGATCTCTTGGTTCTGGCATCG

ATGAAGAACGCAGCGAAATGCGATAAGTAATGTGAATTGCAGAATTCAGTGAATCATCGA

ATCTTTGAACGCACATTGCGCCCTCTGGTATTCCGGAGGGCATGCCTGTTCGAGCGTCAT

TTCAACCCTCAAGCCTGGCTTGGTGATGGGGCACTGGGGAGCAGGCCCTGAAATACAGTG

GCGAGCTCGCCAGGACTCCGAGCGCAGTAGACCCTCGCTAAGG-CCTGGCGGTGCCCTGC

TGAAAGAGAAGGAAGGTTAGTAAACATCACCATGTTGGTACAGCTTCGTCGGCGCATTTT

CACCCCTCCCTCTGGATTTTCATGGTGCGGGGCTTAGAGCTTATCTCACC---------G

AAAACATGCTGACATCTGTACAGCCGCCGAGCTTGGTAAGGGTCGCACCTGAGCCCCACC

ATCGCGACCCACCCCCTGGGACACCCAGATAAGACGCGTCCATTGCTAACATGTTTTTCT

CGCCCACAGGTTCACCTGCAGACCGGCCAATGCGTAAGTTGCTGTCACACCAGA-CCCTA

TCATCGCCACCTGTAGCACGTTTCCCAGGGTAACCAAATCGGTGCTGCTTTCTGGTGCGT

CC-AAGTCCACCACCGCGAT-CGACCTTGAAGCATACTGACCTCGTAGGCAAACCATCTC

TGGCGAGCACGGTCTCGACAGCAATGGCGTGTATGCACCTCCTATTTCTGTCTTTCTCGT

-----CTGACAATCACACAGTTACAACGGCACTTCCGAGCTCCAGCTCGAGCGCATGAAC

GTCTACTTCAACGAGGTAAGTACGTCG--TTGACCATCTGCGGCTTTGCTAACGCGTCAT

CAGGCCTCCGGCAACAAGTATGTGCCCCGCGCCGTCCTCGTCGATCTCGAGCCCGGTACC

ATGGACGCCGTC

>Diaporthe_eres_CGMCC_3_17084

????????????????????????????????????????????????????????????

????????????????????????????????????????????????????????????

????????????????????????????????????????????????????????????

????????????????????????????????????????????????????????????

????????????????????????????????????????????????????????????

????????????????????????????????????????????????????????????

????????????????????????????????????????????????????????????

????????????????????????????????????????????????????????????

????????????????????????????????????????????????????????????

????????????????????????????????????????????????????????????

???????????????????????????NNNNNNNNNNNNNNNNNNNNNNNNNNNNNNNNN

NNNNNNNNNNNNNNNNNNNNNNNNNNNACCCTTTGTGAACTTTTACTGTTGCCTCGGCTA

GCTGGGGGGCCCCTCCGGGTGTTGAGACGGCACGCCGGCGGCCAGCCCAACTCTTGTTTT

TACACTGAAACTCTAAATGAATCAAAACTTTCAACAACGGATCTCTTGGTTCTGGCATCG

ATGAAGAACGCAGCGAAATGCGATAAGTAATGTGAATTGCAGAATTCAGTGAATCATCGA

ATCTTTGAACGCACATTGCGCCCTCTGGTATTCCGGAGGGCATGCCTGTTCGAGCGTCAT

TTCAACCCTCAAGCCTGGCTTGGTGATGGGGCACTGAGAAGCAGGCCCTGAAATTCAGTG

GCGAGCTCGCCAGGACCCCGAGCGCAGTAGACCCTCGCTAAGGCCCTGGCGGTGCCCTGC

TGAAAGAGAAGGAAGGTTAGTAAATATCATCATGCTCGCGCGGCCTCGCCAGCGCATTTT

CACCCCTCGCTTTGGATTTTCAGAGTGCGGGGCTTAGGGCTTATCTTGTCACCACCACCG

AATATATGCTGATATCTACACAGCCGCCGAGCTTGGTAAGGGNNNNNNNNNNNNNNNNNN

NNNNNNNNNNNNNNNNNNNNNNNNNNNNNNNNNNNNNNNNNNNNNNNNNNNNNNNNNNNN

NNNNNNNNNNNNNNNNNNNNNNNNNNNNNNNNNNNNNNNNNNNNNNNNNNNNNNNNNNNN

NNNNNNNNNNNNNNNNNNNNNNNNNNNNNNNNNNNNNNNNNNTGCTGCTTTCTGGTGCGT

TCCAAGTCCACCGCCGCGATACGACCTCGAAGCATGCTGACCTCGTAGGCAAACCATCTC

TGGCGAGCACGGCCTCGACAGCAATGGCGTGTATGCACCTCCTATTCCTGTCCATCTTGG

CTTCCCTGACAATCGCATAGTTACAACGGCACTTCTGAGCTCCAGCTCGAGCGCATGAAC

GTCTACTTCAACGAGGTAAGTACATCATTCCGACCATCTCCAACCTCACTAACGTGTTAT

CAGGCCTCCGGCAACAAGTATGTTCCTCGCGCCGTCCTCGTCGATCTCGAGCCCGGTACC

ATGGACGCCGTC

>Diaporthe_ellipsospora_CGMCC_3_20099

NNNNNNNNNNNNNNNNNNNNNCACCCGCCCTTGCTGTCGCGCATGCTAACGGACCGTTTT

CGGCCTGCAGGATAAGGATGGCGATGGTTAGTGCGGCCGCTCTCACCTAGCACGCGTCAT

GCTCGATCCGCCGCGACGGCCTGCGCGCAAGCAGGCGATATCACTTTCACGAGTTGCTGA

GGTGTAGGACAAATCACCACCAAGGAGCTCGGCACGGTCATGCGGTCCCTGGGCCAGAAC

CCGTCCGAGTCTGAGCTGCAAGACATGATCAACGAGGTCGACGCCGACAACAATGGCACC

ATCGACTTTGGTACGTCCGGATGCTCGCCCATCCGCGCCCTCCACCGGAGGTGTCAAGAA

GCCTCACCGCTACAAGCCTGGTACCGTCGCTCTGCGTGAGATCCGTCGCTACCAGAAGAG

CACCGAGCTGCTGATCCGCAAGCTCCCCTTCCAGCGTCTGGTAAGCAGGTTCGTGAGATC

GCCCAGGACTTCAAGTCCGACCTGCGCTTCCAGTCTTCCGCCATCGGTGCCCTGCAGGAG

TCCGTCGAGTCTTACCTCGTCTCCCTGTTCGAGGACACCAACCTGTGCGCCATCCACGCC

AAGCGTGTCACCATCCAGTCGGTATGTAACCAAGTTTTCGGTGGTGAACCCAGGGGAGGG

TTCATGGTGCCCCCGGGGCCCCCCGGAACCCTTTGTGAAATT-TTATGGTGCCTTGGTAA

GTGGGGGCCCCCCTCGAGACAAGGAGCG-GCGCGCCGGTGGCCCATTAAACTCTGTTTCT

T--AGTGAATCTCTAAATGAATCAAAACTTTCAACAACGGATCTCTTGGTTCTGGCATCG

ATGAAGAACGCAGCGAAATGCGATAAGTAATGTGAATTGCAGAATTCAGTGAATCATCGA

ATCTTTGAACGCACATTGCGCCCTCTGGTATTCCGGAGGGCATGCCTGTTCGAGCGTCAT

TTCAACCCTCAAGCCTGGCTTGGTGTTGGGGCACTGAAGGGCAGGCCCTGAAATACAGTG

GCGAGCTCGCCAGGACTCCGAGCGCAGTAG-ATCTCGTTAAGG-CCTGGCGGTGCCCTGC

NNNNNNNNNNNNNNNNNNNNNNNNNNNCATCATGGTTGCGACTGTCCGTTGGCGCATTTT

CACCCCTCGCTCTGCAATTTCAGGGTGCGGGCCTTAGGGCTTATCTCACCATCATAACCA

CAACCATGCTAATATCCCCACAGCCGCCGAGCTTGGTNNNNNNNNNNNNNNNNNNNNNNN

NNNNNNNNNNNNNNNNNNNNNNNNNNNNNNNNNNNNNNNNNNNNNNNNNNNNNNNNNNNN

NNNNNNNNNNNNNNNNNNNNNNNNNNNNNNNNNNNNNNNNNNNNNNNNNNNNNNNNNNNN

NNNNNNNNNNNNNNNNNNNNNNNNNNNNNNNNNNNNNNNNNNNNNNNNNNNNNNNNNNNN

NNNNNAGCCACCACCGCGGTACGACCTCGAAGCATACTGACCTCGCAGGCAAACCATCTC

TGGCGAGCACGGCCTCGACAGCAATGGCGTGTATGTACCTCCTTTTCCTGCCCATCTCGT

CCCCCTTGACAGTCGCACAGTTACAACGGCACTTCCGAGCTCCAGCTCGAGCGCATGAAC

GTCTACTTCAACGAGGTATGTACGCCGTTCTGGCCATCCACAGCCTTGCTAACGCGTTAT

CAGGCCTCCGGCAACAAGTATGTGCCTCGCGCCGTCCTCGTCGATCTCGAGCCCGGTACC

ATGGACGCCGTC

>Diaporthe_endocitricola_ZHKUCC20_0012

NNNNNNNNNNNNNNNNNGACTCGCCCGCCCTTGCTGTTGCGCATGCTAACGGACCGTTTT

CGGCTTGTAGGATAAGGATGGCGATGGTTAGTGCGGCCGCTCTCACACAGCACGCGTCAT

GCTCGATCCTCCGCGACGGCCTGCGCGCAACCAAGCGTTATCACTATTGCGAGTTGCTGA

GGTGCAGGACAAATCACCACCAAGGAGCTCGGCACGGTCATGCGATCCCTGGGCCAGAAC

CCTTCCGAGTCTGAGCTGCAAGATATGATCAACGAGGTCGACGCCGACAACAACGGCACC

ATTGACTTTGGTACGTCCAGATATACGCCCA?????????????????????????????

????????????????????????????????????????????????????????????

????????????????????????????????????????????????????????????

????????????????????????????????????????????????????????????

????????????????????????????????????????????????????????????

???????????????????????????NNNNNNNNNTCCGTAGGTGAACCTGCGGAGGGA

TCATTGCTGCCCCAGGCGCACCCAGAAACCCTTTGTGAACTTTT-TTGTTGCCTCGGCAT

GCTGGTAGGCCCCTC-CGGTGAGGAGACGGCACGCCGGCGGCCAAGTTAACTCTTGTTTT

TACACTGAAACTCTAAATGAATCAAAACTTTCAACAACGGATCTCTTGGTTCTGGCATCG

ATGAAGAACGCAGCGAAATGCGATAAGTAATGTGAATTGCAGAATTCAGTGAATCATCGA

ATCTTTGAACGCACATTGCGCCCTCTGGTATTCCGGAGGGCATGCCTGTTCGAGCGTCAT

TTCAACCCTCAAGCATTGCTTGGTGTTGGGGCACTGCGAAGCAGGCCCTGAAATCTAGTG

GCGAGCTCGCCAGGACCCCGAGCGCAGTAGACCCTCGCTAAGGCCCTGGCGGTGCCCTGC

TGAAAGAGAAGGAAGGTTAGTAAACATCATCATGTTGGTACGGCTTCGTCAGCGCATTTT

CACCCCTCGCTCTGGATTTTCAGGGTGCGGGGCTTAGAGCTTATCT-----CTACCGCCG

GAAACATGCTGATATCTACATAGCCGCCGAGCTTGGTAAGGGNNNNNNNNNNNNNNNNNN

NNNNNNNNNNNNNNNNNNNNNNNNNNNNNNNNNNNNNNNNNNNNNNNNNNNNNNNNNNNN

NNNNNNNNNNNNNNNNNNNNNNNNNNNNNNNNNNNNNNNNNNNNNNNNNNNNNNNNNNNN

NNNNNNNNNNNNNNNNNNNNNNNNNNNNGGTAACCAAATCGGTGCTGCTTTCTGGTGC--

-----GTCCACCACCGCGATATGACCTCGAGCCATACTGACCTCGCAGGCAAACCATCTC

TGGCGAGCACGGCCTCGACAGCAATGGCGTGTATGCACCTCCTATTCCTACCTTTCTCGT

CTGCCCTGACAATCATACAGTTACAACGGCACTTCCGAGCTCCAGCTCGAGCGCATGAAC

GTCTACTTCAACGAGGTAAGTACGTCG--CTGACCATCTGCAGCCTTGCTAACGCGTTAT

CAGGCCTCCGGCAACAAGTATGTCCCTCGCGCCGTCCTCGTCGATCTCGAGCCCGGTACC

ATGGACGCCGTC

>Diaporthe_endophytica_CBS_133811

CTTTGTGAGTTATCTCCGCCTTGCCCGCCGTTGCCGTTGCGCATGCTAACGGACCGTTTT

CGGCCTGCAGGATAAGGATGGCGATGGTTAGTGCGGTCACTCCCGGCTAGCACGCGTCAT

GCTCGATCCGCCGCGACGGTCTGCGCGCGAGCGACCGTCATCAATATCACGGGTTGCTAA

GATGTAGGACAAATCACCACCAAGGAGCTCGGCACAGTCATGCGGTCGCTTGGTCAAAAC

CCTTCCGAGTCCGAGCTGCAGGACATGATCAACGAGGTCGACGCCGACAACAACGGCACC

ATTGACTTTGGTAAGTCTCAACTGT--TACATCCGCGCCCTCCACCGGAGGTGTCAAGAA

GCCTCACCGCTACAAGCCTGGTACCGTCGCTCTGCGTGAGATCCGTCGCTACCAGAAGAG

CACCGAGCTGCTGATCCGCAAGCTCCCCTTCCAGCGTCTGGTATGCAGGTCCGTGAGATC

GCCCAGGACTTCAAGTCCGACCTGCGCTTCCAGTCTTCCGCCATCGGTGCTCTCCAGGAG

TCCGTCGAGTCTTACCTCGTCTCCCTCTTTGAGGACACCAACCTGTGCGCCATCCACGCC

AAGCGTGTCACCATCCAGTCGGTACGTAACAAGGTCTCCGTTGGTGAACCAGCGGAGGGA

TCATTGCTGCTTC-GGCGCACCCAGAAACCCTTTGTGAACTTA--TTGTTGCCTCGGTAG

GCCGGGAGGCCCCCTGAAACAGGGAGCA-GCCCGCCGGCGGCCAACCAAACTCTTGTTTC

TACAGTGAATCTCTAAATGAATCAAAACTTTCAACAACGGATCTCTTGGTTCTGGCATCG

ATGAAGAACGCAGCGAAATGCGATAAGTAATGTGAATTGCAGAATTCAGTGAATCATCGA

ATCTTTGAACGCACATTGCGCCCTCTGGTATTCCGGAGGGCATGCCTGTTCGAGCGTCAT

TTCAACCCTCAAGCCTGGCTTGGTGATGGGGCACTGAAGGGCAGGCCCTGAAATCTAGTG

GCGAGCTCGCCAGGACCCCGAGCGTAGTAGA-TCTCGCTAAGGCCCTGGCGGTGCCCTGC

TGAAAGAGAAGGAAGGTTAGTAAACATCGTTGTGTCTACGAGGCTTCGTCAAGGCATTTT

CACCCCTCCCTCTGGATTTTCAGGGTGCGGGGCTTACCGCTTATCTCAGTGAC-GCAC-G

AAAAGATGCTGATTTCTGAACAGCCGCCGAGCTGGGTAAGGGTCGCACCTGAGCCCCACC

ATCGCGACCCACCCCCTGCGACACCCAGATAACACGCGTCGATTGCTAACATGTTTTTCT

CGCCCACAGGTTCACCTTCAGACCGGCCAATGCGTAAGTTGCTGTCACCGCCAGGGCTTA

T---CGCCACCCGTAGCACGTTTCCCAGGGTAACCAAATCGGTGCTGCTTTCTGGTGCGT

CCCAAACCTACCACCGCGAGGCGAGCTCGAAGCATACTGACCTCGTAGGCAAACCATCTC

TGGCGAGCACGGTCTCGACAGCAATGGCGTGTATGCACCTCCTATTCCTGCCCACCTGGT

CCTCCCTGATGATCGCACAGTTACAACGGCACTTCCGAGCTCCAGCTCGAGCGCATGAAC

GTCTACTTCAACGAGGTATGTACGTCGTTTTGA-AGCTCTCGCCCTTGCTAACGCGTTTT

CAGGCTTCCGGCAACAAGTATGTGCCCCGCGCCGTCCTCGTCGATCTCGAGCCCGGTACC

ATGGACGCCGTC

>Diaporthe_eres_CBS_138594

CTTTGTAATTTCCAGCCGACATGCCCGCCCTTGCTGGTGCGCATGCTAACGGACCGTTTT

CGGCTTGTAGGATAAGGATGGCGATGGTTAGTGCGGCCGCTCCCACCTAGCACGCGTCAT

GTTCGATCCGCCGCGACAGCCTGCGCGCAACCAAGCGTTATCACTATCACGAGTTGCTGA

GGTGTAGGGCAAATCACCACCAAGGAGCTCGGCACGGTCATGCGATCCCTGGGTCAGAAC

CCGTCCGAGTCTGAGCTGCAAGATATGATTAACGAGGTCGACGCCGACAACAATGGCACC

ATTGACTTTGGTACGTCCAGATGCTCGCGCTTCCGCGCCCTCCACCGGAGGTGTCAAGAA

GCCTCACCGCTACAAGCCTGGTACCGTCGCTCTGCGTGAGATCCGTCGCTACCAGAAGAG

CACCGAGCTGCTGATCCGCAAGCTCCCCTTCCAGCGTCTGGTATGTAGGTCCGTGAGATC

GCCCAGGACTTCAAGTCCGACCTCCGCTTCCAGTCTTCCGCCATCGGTGCCCTGCAGGAG

TCGGTTGAGTCTTACCTCGTCTCCCTCTTCGAGGACACCAACCTGTGCGCCATCCACGCC

AAGCGTGTCACCATCCAGTCGGTACGTNNNNNNNNNNNNNNNNNNNNNNNNGCGGAGGGA

TCATTGCTGCCCCAGGCGCACCCAGAAACCCTTTGTGAACTTTTACTGTTGCCTCGGCTA

GCTGGGGGGCCCCTCCGGGTGTTGAGACAGCCCGTCGGCGGCCAACCTAACTCTTGTTTT

TACACTGAAACTCTAAATGAATCAAAACTTTCAACAACGGATCTCTTGGTTCTGGCATCG

ATGAAGAACGCAGCGAAATGCGATAAGTAATGTGAATTGCAGAATTCAGTGAATCATCGA

ATCTTTGAACGCACATTGCGCCCTCTGGTATTCCGGAGGGCATGCCTGTTCGAGCGTCAT

TTCAACCCTCAAGCCTGGCTTGGTGATGGGGCACTGAGAAGCAGGCCCTGAAATTCAGTG

GCGAGCTCGCCAGGACCCCGAGCGCAGTAGACCCTCGCTAAGGCCCTGGCGGTGCCCTGC

TGAAAGAGAAGGAAGGTTAGTAAATATCATCATGCTCGCGCGGCCTCGCCAGCGCATTTT

CACCCCTCGCTTTGGATTTTCAGAGTGCGGGGCTTAGGGCTTATCTTGTCACCACCACCG

AATATATGCTGATATCTACACAGCCGCCGAGCTTGGTAAGGGNNNNNNNNNNNNNNNNNN

NNNNNNNNNNNNNNNNNNNNNNNNNNNNNNNNNNNNNNNNNNNNNNNNNNNNNNNNNNNN

NNNNNNNNNNNNNNNNNNNNNNNNNNNNNNNNNNNNNNNNNNNNNNNNNNNNNNNNNNNN

NNNNNNNNNNNNNNNNNNNNNNNNNNNNNNNNNNNNNNNNGGTGCTGCTTTCTGGTGCGT

TCCAAGTCCACCGCCGCGATACGACCTCGAAGCATGCTGACCTCGTAGGCAAACCATCTC

TGGCGAGCACGGCCTCGACAGCAATGGCGTGTATGCACCTCCTATGCCTGTCCATCTTGA

CTTCCCTGACAATCGCACAGTTACAACGGCACTTCTGAGCTCCAGCTCGAGCGCATGAAC

GTCTACTTCAACGAGGCAAGTACAACATTCCGACCATCTCCAAGTTCGCTAACGCGTTAT

CAGGCCTCCGGCAACAAGTATGTTCCTCGCGCCGTCCTCGTCGATCTCGAGCCCGGTACC

ATGGACGCCGTC

>Diaporthe_etinsideae_BRIP_64096a

????????????????????????????????????????????????????????????

????????????????????????????????????????????????????????????

????????????????????????????????????????????????????????????

????????????????????????????????????????????????????????????

????????????????????????????????????????????????????????????

????????????????????????????????????????????????????????????

????????????????????????????????????????????????????????????

????????????????????????????????????????????????????????????

????????????????????????????????????????????????????????????

????????????????????????????????????????????????????????????

???????????????????????????AACAAGGTCTCCGTTGGTGAACCAGCGGAGGGA

TCATTGCTGCTTC-GGCGCACCCAGAAACCCTTTGTGAACTTATACTGTTGCCTCGGCTG

GCCGGGAGGCCCCCTGAAACAGGGAGCA-GCCCGCCGGCGGCCAACCAAACTCTTGTTTC

T-TAGTGAATCTCT-AATGAATCAAAACTTTCAACAACGGATCTCTTGGTTCTGGCATCG

ATGAAGAACGCAGCGAAATGCGATAAGTAATGTGAATTGCAGAATTCAGTGAATCATCGA

ATCTTTGAACGCACATTGCGCCCTCTGGTATTCCGGAGGGCATGCCTGTTCGAGCGTCAT

TTCAACCCTCAAGCCTAGCTTGGTGTTGGGGCACCGGAGGGCGGGCCCTGAAATCTAGTG

GCGAGCTCGCCAGGACCCCGAGCGTAGTAGA-TCTCGTTAAGGCCCTGGCGGTGCCCTGC

TGAAANNGAAGGAAGGTTAGTAAACATTACTGCGTTTGCGCGGCTTCGTCAGGGCATTTT

CACCCCTCCCTTTGGATTTTCAGGGTGCGGGGCTTACGGCTTA--TCGCTGCCACCACCA

ATCAGAAGCTGACATCTCTACAGCCGCCGAGCTCGGCAAGGGTCGCACCTCAGCCCCACC

ATCGCGACCCACCCCCTGGGACACCCAGATAAGACGCGTCGATTGCTAACATGTTTTTCT

CGCCCACAGGTTCACCTCCAGACTGGCCAATGCGTAAGTTGCTGTCACCGCCCGACCTTA

T---CGCCACCTGTAGCACGTTTCCCAGGGTAACCAAATCGGTGCTGCTTTCTGGTGCGT

CCAGAGTCGACCGCCGCGACAGTAGCTCGTAGCATACTGACATCGTAGGCAAACCATCTC

TGGCGAGCACGGCCTCGACAGCAATGGCGTGTATGCACCTCCTATTCCTGCCTATCTCGT

CCTACCTGACAATGGCACAGCTACAACGGCACTTCTGAGCTCCAGCTCGAGCGCATGAAC

GTCTACTTCAACGAGGTAAGCACGTTGTTTTGACCACCTGCGGCCTAGCTAACGCGTTAT

CAGGCCTCCGGCAACAAGTATGTGCCTCGCGCCGTCCTCGTCGATCTCGAGCCCGGTACC

ATGGATGCCGTC

>Diaporthe_eucalyptorum_CBS_132525

????????????????????????????????????????????????????????????

????????????????????????????????????????????????????????????

????????????????????????????????????????????????????????????

????????????????????????????????????????????????????????????

????????????????????????????????????????????????????????????

????????????????????????????????????????????????????????????

????????????????????????????????????????????????????????????

????????????????????????????????????????????????????????????

????????????????????????????????????????????????????????????

????????????????????????????????????????????????????????????

???????????????????????????AACAAGGTCTCCGTTGGTGAACCAGCGGAGGGA

TCATTGCTGCCCCAGGCGCACCCAGAAACCCTTTGTGAACTTTTACTGTTGCCTCGGCAT

GCTGGGGGGTCCCTTGAGACAAGGAGCAGGCACGCCGGCGGCCAAGTTAACTCTTGTTTT

TACACTGAAACTCTAAATGAATCAAAACTTTCAACAACGGATCTCTTGGTTCTGGCATCG

ATGAAGAACGCAGCGAAATGCGATAAGTAATGTGAATTGCAGAATTCAGTGAATCATCGA

ATCTTTGAACGCACATTGCGCCCTCTGGTATTCCGGAGGGCATGCCTGTTCGAGCGTCAT

TTCAACCCTCAAGCACTGCTTGGTGTTGGGGCACTGAAGGGCAGGCCCTGAAATCTAGTG

GCGAGCTCGCCAGGACCCCGAGCGCAGTAGACCCTCGCTAAGGCCCTGGCGGTGCCCTGC

TGAAA???????????????????????????????????????????????????????

????????????????????????????????????????????????????????????

????????????????????????????????????????????????????????????

????????????????????????????????????????????????????????????

????????????????????????????????????????????????????????????

????????????????????????????????????????????????????????????

????????????????????????????????????????????????????????????

????????????????????????????????????????????????????????????

????????????????????????????????????????????????????????????

????????????????????????????????????????????????????????????

????????????????????????????????????????????????????????????

????????????

>Diaporthe_eugeniae_CBS_444_82

NNNNNNNNNNNNNNACCGACTCGCCCGCCCTTGCTGTTGCGCATGCTAACGGACCGTTTT

CGGCTTGTAGGATAAGGATGGCGATGGTTAGTGCGGCCGCTCTCACGCAGCACGCGTCAT

GCTCGATCCTCCGCGACGGCCTGCGCGCAACCAAGCGTTATCACTATTGCGAGTTGCTGA

GGTGCAGGACAAATCACCACCAAGGAGCTCGGCACCGTCATGCGATCCCTGGGCCAGAAC

CCTTCCGAGTCTGAGCTGCAAGATATGATTAACGAGGTCGACGCCGACAACAACGGCACC

ATTGACTTTGGTACGTCCAGATATACGCCCATCCGCGCCCTCCACCGGAGGTGTCAAGAA

GCCTCACCGCTACAAGCCTGGTACCGTCGCTCTGCGTGAGATCCGTCGCTACCAGAAGAG

CACTGAGCTGCTGATCCGCAAGCTGCCCTTCCAGCGTCTGGTATGCAGGTCCGTGAGATC

GCCCAGGACTTCAAGTCCGACCTCCGCTTCCAGTCTTCCGCCATCGGTGCCCTGCAGGAG

TCCGTCGAGTCTTACCTCGTCTCCCTCTTCGAGGACACCAACCTGTGCGCCATCCACGCC

AAGCGTGTCACCATCCAGTCGGTACGTAACAAGGTCTCCGTTGGTGAACCAGCGGAGGGA

TCATTGCTGCCCCAGGCGCACCCAGAAACCCTTTGTGAACTTTT-TTGTTGCCTCGGCAT

GCTGGTAGGCCCCTC-CGGTGAGGAGACGGCACGCCGGCGGCCAAGTTAACTCTTGTTTT

TACACTGAAACTCTAAATGAATCAAAACTTTCAACAACGGATCTCTTGGTTCTGGCATCG

ATGAAGAACGCAGCGAAATGCGATAAGTAATGTGAATTGCAGAATTCAGTGAATCATCGA

ATCTTTGAACGCACATTGCGCCCTCTGGTATTCCGGAGGGCATGCCTGTTCGAGCGTCAT

TTCAACCCTCAAGCATTGCTTGGTGTTGGGGCACTGCGAAGCAGGCCCTGAAATCTAGTG

GCGAGCTCGCCAGGACCCCGAGCGTAGTAGACCCTCGCTAAGGCCCTGGCGGTGCCCTGC

TGAAAGAGAAGGAAGGTTAGTAAACATCATCATGTTGGTACAGCTTCGTCAGCGCATTTT

CACCCCTCGCTCTGGATTTTCAGGGTGCGGGGCTTAGAGCTTATCTAGTTATTACCACCG

GAAACATGCTGATATCTACATAGCCGCCGAGCTTGGTAAGGGTCGCACCTGAGCCCCACC

ATCGCGACCCACCCCCTGGGACACCCAGATAAAACGCGTCGATTGCTAACGTGTTTTCCT

CGACTACAGGTTCACCTTCAGACCGGCCAATGCGTAAGTTGCTGTCACGTCGGA-CCTTA

TCAACGCCACCTGTAGCACGTTTCCCAGGGTAACCAAATCGGTGCTGCTTTCTGGTGC--

-----GTCCACTACCGCGATACGATCTCGAACCCTACTGACCTCGCAGGCAAACCATCTC

TGGCGAGCACGGCCTCGACAGCAATGGCGTGTATGCACCTCCTATTCCTACCTCTCTCGT

CTGCCCTGACAATCATACAGTTACAACGGCACTTCCGAGCTCCAGCTCGAGCGCATGAAC

GTCTACTTCAACGAGGTAAGTACGTCG--TTGACCATGTGCAGCCTTGCTAACGCGTTAT

CAGGCCTCCGGCAACAAGTATGTCCCTCGCGCCGTCCTCGTCGATCTTGAGCCCGGTACC

ATGGACGCCGTC

>Diaporthe_fibrosa_CBS_109751

CTTTGTAAGTCATCTTCCACATGCCCGCCCTTGCTGTTGCGCGTGCTAACGGACCGTTTT

CGGCCTGCAGGATAAGGATGGCGATGGTTAGTGTGGCCGCACCCACGGAGCACGCGTCAT

GCTCGATCCGTCGCGACGGCCTGCGCGCGAACAGGCGTCAGCACTATCAGGGATTGCTAA

GGCGTAGGACAAATCACCACGAAGGAGCTCGGCACTGTCATGCGGTCTCTGGGCCAGAAC

CCGTCCGAGTCTGAACTGCAAGATATGATTAACGAGGTCGATGCCGACAACAATGGCACC

ATTGACTTTGGTACGTCCAGACGCTCGCCCATCCGCGCCCTCCACCGGAGGTGTCAAGAA

GCCTCACCGCTACAAGCCTGGTACCGTCGCTCTGCGTGAGATCCGTCGCTACCAGAAGAG

CACCGAGCTGCTGATCCGCAAGCTCCCCTTCCAGCGCCTGGTATGCAGGTCCGCGAGATC

GCTCAGGACTTCAAGTCCGACCTCCGCTTCCAGTCTTCCGCCATCGGCGCCCTGCAGGAG

TCCGTCGAGTCTTACCTCGTCTCCCTCTTCGAGGACACCAACCTGTGCGCCATCCACGCC

AAGCGTGTCACCATCCAGTCGGTATGTAACAAGGTCTCCGTTGGTGAACCAGCGGAGGGA

TCATTGCTGCCCCCGGCGCACCCAGAAACCCTTTGTGAACTTTTACTGTTGCCTCGGCAG

GCCGGGGGGCCCCTCGAGACGAGGAGCAGGCCCGCCGGCGGCCAAGACAACTCCTGTTTT

TACACTGAAACTCTAAATGAATCAAAACTTTCAACAACGGATCTCTTGGTTCTGGCATCG

ATGAAGAACGCAGCGAAATGCGATAAGTAATGTGAATTGCAGAATTCAGTGAATCATCGA

ATCTTTGAACGCACATTGCGCCCTCTGGTATTCCGGAGGGCATGCCTGTTCGAGCGTCAT

TTCACCCCTCAAGCCTGGCTTGGTGATGGGGCACTGAAGGGCAGGCCCTGAAATTCAGTG

GCGAGCTCGCCAGGACCCCGAGCGCAGTAGACCCTCGCTAAGGCCCTGGCGGTGCCCTGC

TGAAAGAGAAGGAAGGTTAGTAAACATCATCGCTCCTGCGCAGCTCCATTAGCGCATTTT

CACCCCTCGTTCTGGATTTTCAGGGTGCGGGGCTTAGAGCTTATCTCACCACCACC---G

GAAAGAAGCTAACATCTATACAGCCGCCGAGCTTGGTAAGGGTCGCACCTGAGCCCCACC

ATCGCGACCTACCCCCTGGGACACCCAGATAAGACGCGTCGATTGCTAATGTGCTTTTCT

CTCCCGCAGGTCCACCTTCAGACCGGCCAATGCGTAAGTTGCTGTCACCACCGGACCCTA

T---CGCCACCTGTAGCACGTTTGCCAGGGTAACCAAATCGGTGCTGCTTTCTGGTGCGT

CCCAAACTCACCACCGCGATACGACCTCGCGGCATACTGACCTCGCAGGCAAACCATCTC

TGGCGAGCACGGCCTCGACAGCAATGGCGTGTATGCACCTCCTACTCCTGCACATCTCGC

CCTCCCTGACAATTGCACAGTTACAACGGCACTTCCGAGCTCCAGCTCGAGCGCATGAAC

GTCTACTTCAACGAGGTATGTGCGTTTTTTCGGTCATCGCCAACCTTGCTAACTCGTTAT

CAGGCCTCCGGCAACAAGTATGTTCCTCGCGCGGTCCTCGTCGATCTCGAGCCCGGTACC

ATGGACGCCGTC

>Diaporthe_fici_septicae_MFLU_18_2588

????????????????????????????????????????????????????????????

????????????????????????????????????????????????????????????

????????????????????????????????????????????????????????????

????????????????????????????????????????????????????????????

????????????????????????????????????????????????????????????

????????????????????????????????????????????????????????????

????????????????????????????????????????????????????????????

????????????????????????????????????????????????????????????

????????????????????????????????????????????????????????????

????????????????????????????????????????????????????????????

???????????????????????????NNNNNNNNNTCCGTTGGTGAACCAGCGGAGGGA

-CATTGCTGCCCC-GGCGCACCCAGAAACCCTTTGTGAACTTTA-CTGTTGCCTCGGCAG

GCCGGGAGGCCCCCCGAGACGGGGAGCA-GCCCGCCGGCGGCCAACTAAACTCTTGTTTC

TACAGTGGATCTCTAAATGAATCAAAACTTTCAACAACGGATCTCTTGGTTCTGGCATCG

ATGAAGAACGCAGCGAAATGCGATAAGTAATGTGAATTGCAGAATTCAGTGAATCATCGA

ATCTTTGAACGCACATTGCGCCCTCTGGTATTCCGGAGGGCATGCCTGTTCGAGCGTCAT

TTCAACCCTCAAGCCTGGCTTGGTGTTGGGGCACTGAAGGGCAGGCCCTGAAATCTAGTG

GCGAGCTCGCTAGGACCCCGAGCGTAGTAGA-TCTCGTTAAGGCCCTGGCGGCGCCCTGC

NNNNNGAGAAGGAAGGTTAGTAAACATCATTGCATCTGCGCGGCTTCGTCAAGGCATTTT

CACCCCTCGCTCTGGATTTTCAGGGTGCGGGGCTTACCGCTTATCTCGCCGCCACCACCG

GAATTATGCTGATTTCTACACAGCCGCCGAGCTTGGTAAGGGNNNNNNNNNNNNNNNNNN

NNNNNNNNNNNNNNNNNNNNNNNNNNNNNNNNNNNNNNNNNNNNNNNNNNNNNNNNNNNN

NNNNNNNNNNNNNNNNNNNNNNNNNNNNNNNNNNNNNNNNNNNNNNNNNNNNNNNNNNNN

NNNNNNNNNNNNNNNNNNNNTTTCCCAGGGTAACCAAATCGGTGCTGCTTTCTGGTGCGT

CCAGAGTCGACCACCTCGACACTAGCTCGCATCAAACTGACCTCGTAGGCAAACCATCTC

TGGCGAGCACGGTCTCGACAGCAATGGCGTGTATGCACCTCCTATTCCTGCCCATCTCGT

CCCCCCTGACGATTGTACAGTTACAACGGCACTTCCGAGCTCCAGCTCGAGCGCATGAAC

GTCTACTTCAACGAGGTAAGTACGTCATTTTGTCCATCTACGGTCTTGCTAACGCGTTAT

CAGGCCTCCGGCAACAAGTATGTGCCTCGCGCCGTCCTCGTCGATCTCGAGCCCGGTACC

ATGGACGCCGTC

>Diaporthe_foeniculina_CBS_111553

CTTTGTAATCTCCAGCCGATATGCCCGCC--TGCTGTTGCGCATGCTAACGGACCGTTTT

CGGCTTCCAGGATAAGGATGGCGATGGTTAGTACGGCCGCTCCCACCCAACACGCGTCAC

GCTCGATCCGCCCCGATGGCCTGCGCGCAAGCAAGCGTTACCACTATCACGAGTTGCTGA

GGTGTAGGACAAATCACCACCAAGGAGCTCGGCACGGTCATGCGGTCCCTGGGTCAGAAC

CCGTCCGAGTCGGAGCTGCAAGATATGATTAACGAGGTCGACGCCGACAACAACGGCACC

ATTGACTTTGGTACGTTCAGATGCTCGCCTCTCCGCGCCCTCCACCGGAGGTGTCAAGAA

GCCTCACCGCTACAAGCCTGGTACCGTCGCTCTGCGTGAGATCCGTCGTTACCAGAAGAG

CACTGAGCTGCTGATCCGCAAGCTCCCCTTCCAGCGTCTGGTATGCAGGTTCGTGAGATC

GCCCAGGACTTCAAGTCCGACCTCCGCTTCCAGTCCTCCGCCATCGGTGCCCTGCAGGAG

TCCGTCGAGTCTTACCTCGTCTCCCTGTTCGAGGACACCAACTTGTGCGCCATCCACGCC

AAGCGTGTCACCATCCAGTCGGTACGTAACAAGGTCTCCGTTGGTGAACCAGCGGAGGGA

TCATTGCTGCCCCTGGCGCACCCAGAAACCCTTTGTGAACTTTTACTGTTGCCTCGGCAG

GCCGTGGGGTCCCTCAGACAGAGGAGCA-GCCGGCCGGTGGCCAAATTAACTC-TGTTTT

TACACTGAAACTCTAAATGAATCAAAACTTTCAACAACGGATCTCTTGGTTCTGGCATCG

ATGAAGAACGCAGCGAAATGCGATAAGTAATGTGAATTGCAGAATTCAGTGAATCATCGA

ATCTTTGAACGCACATTGCGCCCTCTGGTATTCCGGAGGGCATGCCTGTTCGAGCGTCAT

TTCAACCCTCAAGCCTGGCTTGGTGTTGGGGCACTGGAGAGCAGGCCCTGAAATATAGTG

GCGAGCTCGCCAGGACTCCGAGCGTAGTAGACCCTCGCTAAGG-CCTGGCGGTGCCCTGC

TGAAAGAGAAGGAAGGTTAGTAAACACCATCATGTTCGTGTGGCTTCGTCGGCGCATTTT

CACCCCGCCCTCTGGATTTTCAGGGTGCGGGGCTTAGAGCTTATCTCACA---------A

AAAACATGCTGACTTCTTCACAGCCGCCGAGCTTGGTAAGGGTTCGCGCCTGAACCCCAC

CATCGCGACACACCCCTAGGACACTCAGGTAAGACGCGTCGATTGCTAACATGTTTTTCT

CGCCTACAGGTTCACCTTCAGACCGGTCAATGCGTAAGTTGCTGTCACACCGGA-CCTTA

TCATCGCCACCTGTAGCACGTTTCCCAGGGTAACCAAATCGGTGCTGCTTTCTGGTGCGT

CC-AAGTCAACCACCGCGAGTCGACCTCGAAGCATACTGACCTCGCAGGCAAACCATCTC

TGGCGAGCACGGTCTCGACAGCAATGGCGTGTACGCACCTCCTATTCCTGTCTTTCTCGT

-TGCACTGACAATCACACAGTTACAACGGCACTTCCGAGCTCCAGCTCGAGCGCATGAAC

GTCTACTTCAACGAGGTAAGTACGTTG--TT-ACCATCTACAGCTTTGCTAACGCGTTAT

CAGGCCTCCGGCAACAAGTATGTGCCCCGCGCCGTCCTCGTCGATCTCGAGCCCGGTACC

ATGGACGCCGTC

>Diaporthe_foikelawen_CBS_145289

CTTTGTAAGTTCTCTTC------CCCGCCGTTGCTGTTGCGCATGCTAACGCACCGTTTT

CGGCCTCCAGGATAAGGATGGCGATGGTTAGTGCGGCTGCCCCAACCCCATACGCGTCAC

GATCGATTCGCCGCGACAGCATGCGCGCGATCAAGCGCCATCACTACCAGGAGTTGCTAA

GGTGTAGGACAAATCACCACAAAGGAGCTCGGAACGGTCATGCGATCGCTGGGTCAGAAC

CCGTCCGAGTCTGAGCTGCAAGATATGATCAACGAGGTCGATGCCGACAACAATGGCACC

ATCGACTTTGGTACGTTCAGGTGTCTGCTCA?????????????????????????????

????????????????????????????????????????????????????????????

????????????????????????????????????????????????????????????

????????????????????????????????????????????????????????????

????????????????????????????????????????????????????????????

???????????????????????????NNNNNNNNNNNNNNNNNNNNNNNTGCGGAGGGA

TCATTGTTGCTCAC-GCGCATCCAGAAACCCTTTGTGAACTTATACTGTTGCCTCGGCAG

GCTGGGGGCCCCCCATCGGTGGGGAGCAGGCCCGCCGGCGGCCAAGTTAACTCTTGTTTT

TACACTGAAACTCTAAATGAATCAAAACTTTCAACAACGGATCTCTTGGTTCTGGCATCG

ATGAAGAACGCAGCGAAATGCGATAAGTAATGTGAATTGCAGAATTCAGTGAATCATCGA

ATCTTTGAACGCACATTGCGCCCTCTGGTATTCCGGAGGGCATGCCTGTTCGAGCGTCAT

TTCAACCCTCAAGCTTGGCTTGGTGATGGGGCACTGCAGGGCAGGCCCTTAAATTCAGTG

GCGAGCTCGCCAGGACCCCGAGCGCAGTAGACCCTCGCTAAGGTCTTGGTGCGGCCCTGC

TGAAANNGAAGGAAGGTTAGTAAATATTCACACGCTTGCAAGGCTGCGTCATCGCATTTT

CACCCCTCGTTCTGGATTTTCAGGGTGCGGGGCTTAGAGCTTATCTCACAACCATCACCT

GGAATGTGCTAACACTTTACTAGCCGCCGAGCTTGGCAAGGGNNNNNNNNNNNNNNNNNN

NNNNNNNNNNNNNNNNNNNNNNNNNNNNNNNNNNNNNNNNNNNNNNNNNNNNNNNNNNNN

NNNNNNNNNNNNNNNNNNNNNNNNNNNNNNTGCGTAAGTTGCTGTCACCACCGCACCTTA

T---CGCCCCCTGTAGCACGTTTTCCAGGGTAACCAAATCGGTGCTGCTTTCTGGTGCGT

T----GTCCATCATCGCGACACGACCTCGCAACATATTGACTTCGTAGGCAAACCATCTC

TGGCGAGCACGGCCTCGACAGCAATGGCGTGTACGTACCTCCTATTTCTACCGGTCTCGT

CCGCCCTGACAGCTTCACAGTTACAACGGCTCTTCTGAGCTCCAGCTCGAGCGCATGAGC

GTCTACTTCAACGAGGTCAGTGTTTTATACCCACGATTTCCAGCTTTGCTGACACCTTAT

CAGGCCTCCGGCAACAAGTATGTTCCTCGCGCTGTCCTCGTCGATCTCGAGCCCGGTACC

ATGGACGCCGTC

>Diaporthe_fraxini_angustifoliae_BRIP_54781

????????????????????????????????????????????????????????????

????????????????????????????????????????????????????????????

????????????????????????????????????????????????????????????

????????????????????????????????????????????????????????????

????????????????????????????????????????????????????????????

????????????????????????????????????????????????????????????

????????????????????????????????????????????????????????????

????????????????????????????????????????????????????????????

????????????????????????????????????????????????????????????

????????????????????????????????????????????????????????????

???????????????????????????AACAAGGTCTCCGTTGGTGAACCAGCGGAGGGA

TCATTGCTGCCCCAGGCGCACCCAGAAACCCTTTGTGAACTTTT-TTGTTGCCTCGGCAT

GCTGGTAGGCCCCTC-CGGTGAGGAGACGGCACGCCGGCGGCCAAAACAACTC-TGTTTT

TACACTGAAACTCTAAATGAATCAAAACTTTCAACAACGGATCTCTTGGTTCTGGCATCG

ATGAAGAACGCAGCGAAATGCGATAAGTAATGTGAATTGCAGAATTCAGTGAATCATCGA

ATCTTTGAACGCACATTGCGCCCTCTGGTATTCCGGAGGGCATGCCTGTTCGAGCGTCAT

TTCAACCCTCAAGCATTGCTTGGTGTTGGGGCACTGAGAAGCAGGCCCTGAAATCTAGTG

GCGAGCTCGCCAGGACCCCGAGCGCAGTAGACCCTCGCTAAGGCCCTGGCGGTGCCCTGC

TGAAANNNNNNNNNNNNNNNNNNNNNNCATCATGTTGGTACGGCTTCGTCAGCGCATTTT

CACCCCTCGCTCTGGATTTTCAGGGTGCGGGGCTTAGAGCTTATCTAGTTACTACCACCG

GAAACATGCTGATATCTACATAGCCGCCGAGCTTGGCAAGGGTCGCACCTGAGCCCCACC

ATCGCGACCCACCCCCTGGGACACCCAGATAAAACGCGTCGATTGCTAACGTGTTTTTCT

CGACTACAGGTTCACCTTCAGACCGGCCAATGCGTAAGTCGCTGTCACGACGGA-CCTTA

TCATCGCCACCCGTAGCACGTTTCCCAGGGTAACCAAATCGGTGCTGCTTTCTGGTGC--

-----GTCCACCACCGCGATACGACCTCGAAACATACTGACCTCGCAGGCAAACCATCTC

TGGCGAGCACGGCCTCGACAGCAATGGCGTGTATGCACCTCCTATTCCTACCTCTCTCGT

CTGCCCTGACAATCACACAGTTACAACGGCACTTCCGAGCTCCAGCTCGAGCGCATGAAC

GTCTACTTCAACGAGGTAAGTACGTCG--TTTACCATCTGCAGCCTTGCTAACGCGTTAT

CAGGCCTCCGGCAACAAGTATGTTCCTCGCGCCGTCCTCGTCGATCTCGAGCCCGGTACC

ATGGACGCCGTC

>Diaporthe_fraxinicola_CFCC_52582

NNNNNNNNNNNNNNNNNNNNNNNNCCGCCCTTGCTGGTGCGCATGCTAACGGACCGTTTT

CGGCTTGTAGGATAAGGATGGCGATGGTTAGTGCGGCCGCTCTCACCTAGCACGCGTCTT

GCTCGAACCGCCGCGACAGCCTGCGCGCAACCACGCGTTATCACTATCACGAGTTGCTGA

GGTGTAGGACAAATCACCACCAAGGAGCTCGGCACGGTCATGCGGTCCCTTGGTCAGAAC

CCGTCCGAGTCTGAGCTGCAAGATATGATTAACGAGGTCGACGCCGACAACAATGGCACC

ATTGACTTTGGTACGTCCAGATGCTCGCCCT?????????????????????????????

????????????????????????????????????????????????????????????

????????????????????????????????????????????????????????????

????????????????????????????????????????????????????????????

????????????????????????????????????????????????????????????

???????????????????????????NNNNNNNNNNNNNNNNNNNNNNNNNNNNNNNNN

NNNNNNNNNNNNNNNNNNNNNNNNNNNNCCCTTTGTGAACTTTTACTGTTGCCTCGGAAG

GCTGGGGGGCCCCTCCGGGTGTTGAGACAGCCCGCCGGCGGCCAAGTTAACTCTTGTTTT

TACACTGAAACTCTAAATGAATCAAAACTTTCAACAACGGATCTCTTGGTTCTGGCATCG

ATGAAGAACGCAGCGAAATGCGATAAGTAATGTGAATTGCAGAATTCAGTGAATCATCGA

ATCTTTGAACGCACATTGCGCCCTCTGGTATTCCGGAGGGCATGCCTGTTCGAGCGTCAT

TTCAACCCTCAAGCCTGGCTTGGTGATGGGGCACTGAGAAGCAGGCCCTGAAATTCAGTG

GCGAGCTCGCCAGGACCCCGAGCGCAGTAGACCCTCGCTAAGGCCCTGGCGGTGCCCTGC

TGAAAGAGAAGGAAGGTTAGTAAACACCATGATGCTCTTGTGGCTTCGTCAGCGCATTTT

CACCCCTCGCTTTGGATTTTCAGGGTGCGGGGCTTAGGGCTTATCTCACCACCACTACCG

AACATATGCTGATATCTACACAGCCGCCGAGCTTGGTAAGGG??????????????????

????????????????????????????????????????????????????????????

????????????????????????????????????????????????????????????

????????????????????????????????????????????????????????????

????????????????????????????????????????????????????????????

????????????????????????????????????????????????????????????

????????????????????????????????????????????????????????????

????????????????????????????????????????????????????????????

????????????????????????????????????????????????????????????

????????????

>Diaporthe_fructicola_MAFF_246408

CTTTGTGAGTTATCTCCGCCTTGCCCGCCGTTGCCGTTGCGCATGCTAACGGACCGTTTT

CGGCCTGCAGGATAAGGATGGCGATGGTTAGTGCGGTCACTGCCGGCTAGCACGCGTCAT

GCTCGATCCGCCGCGACGGTCTGCGCGCGAGCGACCGTCATCAACATCACGAGTTGCTAA

GATGTAGGACAAATCACCACCAAGGAGCTCGGCACAGTCATGCGGTCGCTTGGTCAAAAC

CCTTCCGAGTCCGAGCTGCAGGACATGATCAACGAGGTCGACGCCGACAACAACGGCACC

ATTGACTTTGGTAAGTCTGAACTGT--TACATCCGCGCCCTCCACCGGAGGTGTCAAGAA

GCCTCACCGCTACAAGCCTGGTACCGTCGCTCTGCGTGAGATCCGTCGCTACCAGAAGAG

CACCGAGCTGCTGATCCGCAAGCTCCCCTTCCAGCGTCTGGTATGCAGGTCCGTGAGATC

GCCCAGGACTTCAAGTCCGACCTGCGCTTCCAGTCTTCCGCCATCGGTGCTCTCCAGGAG

TCCGTCGAGTCTTACCTCGTCTCCCTCTTCGAGGACACCAACCTGTGCGCCATCCACGCC

AAGCGTGTCACCATCCAGTCGGTACGTAACAAGGTCTCCGTTGGTGAACCAGCGGAGGGA

TCATTGCTGCTTC-GGCGCACCCAGAAACCCTTTGTGAACTTA--TTGTTGCCTCGGTAG

GCCGGGAGGCCCCCTGAAACAGGGAGCA-GCCCGCCGGCGGCCAACCAAACTCTTGTTTC

TACAGTGAATCTCTAAATGAATCAAAACTTTCAACAACGGATCTCTTGGTTCTGGCATCG

ATGAAGAACGCAGCGAAATGCGATAAGTAATGTGAATTGCAGAATTCAGTGAATCATCGA

ATCTTTGAACGCACATTGCGCCCTCTGGTATTCCGGAGGGCATGCCTGTTCGAGCGTCAT

TTCAACCCTCAAGCCTGGCTTGGTGATGGGGCACTGAAGGGCAGGCCCTGAAATCTAGTG

GCGAGCTCGCCAGGACCCCGAGCGTAGTAGA-TCTCGCTAAGGCCCTGGCGGTGCCCTGC

TGAAAGAGAAGGAAGGTTAGTAAACATCGTTGTGTATGCGAGGCTTCGTCAAGGCATTTT

CACCCCTCCCTCTGGATTTTCAGGGTGCGGGGCTTACCGCTTATCTCAGTGAC-GCACCG

AAAAGATGCTGATTTCTGAACAGCCGCCGAGCTGGGTAAGGGTCGCACCTGAGCCCCACC

ATCGCGACCCACCCCCTGCGACACCCAGACAACACGCGTCGATTGCTAACATGTTTTTCT

CGCCCACAGGTTCACCTTCAGACCGGCCAATGCGTAAGTTGCTGTCACCGCCAGACCTTA

T---CGCCACCCGTAGCACGTTTCCCAGGGTAACCAAATCGGTGCTGCTTTCTGGTGCGT

CCCAAGCCTACCACCGCGAGGCGAGCTCGAAGCATACTGACCTCGTAGGCAAACCATCTC

TGGCGAGCACGGTCTCGACAGCAATGGCGTGTATGCACCTCCTATTCCTGCCCACCTGGT

CCTCCCTGATGATCGCACAGTTACAACGGCACTTCCGAGCTCCAGCTCGAGCGCATGAAC

GTCTACTTCAACGAGGTATGTACGTCGTTTTGA-CCCTCTCGCCCTTGCTAACGCGTTTT

CAGGCTTCCGGCAACAAGTATGTGCCCCGCGCCGTCCTCGTCGATCTCGAGCCCGGTACC

ATGGACGCCGTC

>Diaporthe_fujianensis_JZB320149

NNNNNNNNNNNNNNNNNNNNNNNNNNGCTCTTGCTGTTGCGCATGCTAACGGACCGTTTT

CGGCTTGTAGGATAAGGATGGCGATGGTTAGTGCGGCCGCTCTCACACAGCACGCGTCAC

GCTCGATCCGCCGCGACGGCCTGCGCGCAACCAAGCGTTATCACTATTGCGAGTTGCTGA

GGTGTAGGACAAATCACCACCAAGGAGCTCGGCACGGTCATGCGATCCCTGGGACAGAAC

CCGTCCGAGTCTGAGCTGCAAGACATGATTAACGAGGTCGACGCCGACAACAACGGCACC

ATTGACTTTGGTACGTCCAGAAGCACGCCCG?????????????????????????????

????????????????????????????????????????????????????????????

????????????????????????????????????????????????????????????

????????????????????????????????????????????????????????????

????????????????????????????????????????????????????????????

???????????????????????????AACAAGGTCTCCGTTGGTGAACCAGCGGAGGGA

TCATTGCTGCCCCAGGCGCACCCAGAAACCCTTTGTGAACTTTTACTGTTGCCTCGGCAT

GCCGGGGGGCCCCTCGAGACGAGGAGCAGGCACGCCGGCGGCCAAGTTAACTCTTGTTTT

TACACTGAAACTCTAAATGAATCAAAACTTTCAACAACGGATCTCTTGGTTCTGGCATCG

ATGAAGAACGCAGCGAAATGCGATAAGTAATGTGAATTGCAGAATTCAGTGAATCATCGA

ATCTTTGAACGCACATTGCGCCCTCTGGTATTCCGGAGGGCATGCCTGTTCGAGCGTCAT

TTCAACCCTCAAGCATTGCTTGGTGTTGGGGCACTGAAGGGCAGGCCCTGAAATCTAGTG

GCGAGCTCGCCAGGACCCCGAGCGCAGTAGACCCTCGCTAAGGCCCTGGCGGTGCCCTGC

NNNNN???????????????????????????????????????????????????????

????????????????????????????????????????????????????????????

??????????????????????????????????????????NNNNNNNNNNNNNNNNNN

NNNNNNNNNNNNNNNNNNNNNNNNNNNNNNNNNNNNNNNNNNNNNNNNNNNNNNNNNNNN

NNNNNNNNNNNNNNNNNNNNNNNNNNNNNNNNNNNNNNNNNNNNNNNNNNNNNNNNNNNN

NNNNNNNNNNNNNNNNNNNNNNNNNNNNNNNNNNNNNNNNNNNNNNNNNNNNNNNCAA--

-----GTCCACCACCGCGATACGACCTCGAACCATACTGACCTTGCAGGCAAACCATCTC

TGGCGAGCACGGCCTCGACAGCAATGGCGTGTATGCACCTCCTATTCCTACCTCTCTCGT

CTGCCCTGACAATCACACAGTTACAACGGCAGTTCCGAGCTCCAGCTCGAGCGCATGAAC

GTCTACTTCAACGAGGTAAGTACGTCG--TTGACCATCTACAGCCTTGCTAACGCGTTAT

CAGGCCTCCGGCAACAAGTATGTTCCTCGCGCCGTCCTTGTCGATCTCGAGCCCGGTACC

ATGGACGCCGTC

>Diaporthe_fukushii_MAFF625034

????????????????????????????????????????????????????????????

????????????????????????????????????????????????????????????

????????????????????????????????????????????????????????????

????????????????????????????????????????????????????????????

????????????????????????????????????????????????????????????

????????????????????????????????????????????????????????????

????????????????????????????????????????????????????????????

????????????????????????????????????????????????????????????

????????????????????????????????????????????????????????????

????????????????????????????????????????????????????????????

???????????????????????????NNNNNNNNNNNNNNNNNNNNNNNNNNNNNNNNN

NNNNNNNNNNNNNNNNNNNNNNNNNNNNCCCTTTGTGAACTTTTACTGTTGCCTCGGCAT

GCCGGGGGGCCCCTCCTCGGGAGGAGCAGGCACGCCGGCGGCCAACCTAACTCTTGTTTT

TACACTGAAACTCTAAATGAATCAAAACTTTCAACAACGGATCTCTTGGTTCTGGCATCG

ATGAAGAACGCAGCGAAATGCGATAAGTAATGTGAATTGCAGAATTCAGTGAATCATCGA

ATCTTTGAACGCACATTGCGCCCTCTGGTATTCCGGAGGGCATGCCTGTTCGAGCGTCAT

TTCAACCCTCAAGCCTGGCTTGGTGATGGGGCACTGAGAAGCAGGCCCTGAAATTCAGTG

GCGAGCTCGCCAGGACCCCGAGCGCAGTAGACCCTCGCTAAGGCCCTGGCGGTGCCCTGC

NNNNNCGAGAAGAAGGTTAGTAAATATCATCATGCTCGCGCGGCCTCGCCAGCGCATTTT

CACCCCTCGCTTTGGATTTTCAGAGTGCGGGGCTTAGGGCTTATCTTGTCACCACCACCG

AATATATGCTGATATCTACACAGCCGCCGAGCTTGGTAAGGG??????????????????

????????????????????????????????????????????????????????????

????????????????????????????????????????????????????????????

????????????????????????????????????????????????????????????

????????????????????????????????????????????????????????????

????????????????????????????????????????????????????????????

????????????????????????????????????????????????????????????

????????????????????????????????????????????????????????????

????????????????????????????????????????????????????????????

????????????

>Diaporthe_fulvicolor_PSCG_051

NNNNNNNNNNNNNNNNNNNNNNNCCCGCCCTTGCTGTTGCGCATGCTAACGGACCGTTTT

CGGCTCGTAGGATAAGGATGGCGATGGTTAGTGCGGCCGCTCTCACACAGCACGCGTCAT

GCTCGATCCTCCGCGACGGCCTGCGCGCCACCAAGCGTTATCACTATTGCGAGTTGCTGA

GGTGCAGGACAAATCACCACCAAGGAGCTCGGCACAGTCATGCGTTCCCTGGGCCAGAAC

CCTTCCGAGTCTGAGCTGCAAGATATGATTAACGAGGTCGACGCCGACAACAACGGCACC

ATTGACTTTGGTACGTCCAGATATACGCCCATCCGCGCCCTCCACCGGAGGTGTCAAGAA

GCCTCACCGCTACAAGCCTGGTACCGTCGCTCTGCGTGAGATCCGTCGCTACCAGAAGAG

CACTGAGCTGCTGATCCGCAAGCTCCCCTTCCAGCGTCTGGTATGCAGGTCCGTGAGATC

GCCCAGGACTTCAAGTCCGACCTCCGCTTCCAGTCCTCCGCCATCGGTGCCCTGCAGGAG

TCCGTCGAGTCTTACCTCGTCTCCCTCTTCGAGGACACCAACCTGTGCGCCATCCACGCC

AAGCGTGTCACCATCCAGTCGGTACGTNNNNNNNNNNNNNNNNNNNNNNNNNNNNNNNNN

NNNNNNNNNNNNNNNNNNNNNNNNNNNNCCCTTTGTGAACTTTTACTGTTGCCTCGGCAT

GCTGGGGGGCCCCCTGAGACAGGGAGCAGGCACGCCGGCGGCCAAGTTAACTCTTGTTTT

TACACTGAAACTCTAAATGAATCAAAACTTTCAACAACGGATCTCTTGGTTCTGGCATCC

ATGAAAAACGCAGCGAAATGCGATAAGTAATGTGAATTGCAAAATTCAGTGAATCATCCA

ATCTTTGAACGCACATTGCGCCCTCTGGTATTCCGGAAGGCATGCCTGTTCGAGCGTCAT

TTCAACCCTCAAGCATTGCTTGGTGTTGGGGCACTGCGAAGCAGGCCCTGAAATCTAGTG

GCGAGCTCGCTAGGACCCCGAGCGTAATAAACCCTCCCTAAGGCCCTGGCGGTGCCCTGC

TGAAANNNNNNNNNNNNNNNNNNNNNNCATCACGTTGGTACGGCTTCGTCAGCGCATTTT

CACCCCTCGCTCTGGATTTTCAGGGTGCGGGGCTTAGAGCTTATCT-----CTACCGCTG

GAAACATGCTGATATCTACATAGCCGCCGAGCTTGGTAAGGGNNNNNNNNNNNNNNNNNN

NNNNNNNNNNNNNNNNNNNNNNNNNNNNNNNNNNNNNNNNNNNNNNNNNNNNNNNNNNNN

NNNNNNNNNNNNNNNNNNNNNNNNNNNNNNNNNNNNNNNNNNNNNNNNNNNNNNNNNNNN

NNNNNNNNNNNNNNNNNNNNNNNNNNNNNNNNNNNNNNNNNNNNNNNNNNNNNNNNNNNN

NNNNAGGGGGCTAACGCGATATGACCTCGAGCCATACTGACCTCGCAGGCAAACCATCTC

TGGCGAGCACGGCCTCGACAGCAATGGCGTGTATGCACCTCCTATTCCTACCTCTCTCGT

CTGCCTTGACAATCACACAGTTACAACGGCACTTCCGAGCTCCAGCTCGAGCGCATGAAC

GTCTACTTCAACGAGGTAAGTACGTCG--TTGGCCATCTGCAGCTTTGCTAACGTGTTAT

CAGGCCTCCGGCAACAAGTATGTCCCTCGCGCCGTCCTCGTCGATCTCGAGCCCGGTACC

ATGGACGCCGTC

>Diaporthe_fusicola_CGMCC_3_17087

CTTTGTAAGTTATTTTC------GCAGCCCTCACTGTCGCGCATGCTAACGGACCGTTCT

CGGCCTCCAGGATAAGGATGGCGATGGTTAGTGCAGCTGCTCCCAGCTTGTACGCGTCAC

GATCGACCGGCCGCGACGCCTTGCGCGCAACCAAGC-CCATAACTACTAGGAGCTGCTAA

GGTGTAGGACAAATCACCACAAAGGAGCTCGGCACGGTCATGCGATCTCTGGGTCAGAAC

CCGTCCGAGTCTGAGCTGCAGGATATGATCAACGAGGTCGACGCCGACAACAATGGAACC

ATCGACTTTGGTACGTCCAGATGCTCGCTTG?????????????????????????????

????????????????????????????????????????????????????????????

????????????????????????????????????????????????????????????

????????????????????????????????????????????????????????????

????????????????????????????????????????????????????????????

???????????????????????????NNNNNNNNNNNNNNNNNNNNNNNNNNNNNNNNN

NNNNNNNNNNNNNNNNNNNNNNNNNNNACCCTTTGTGAACTTTTACTGTTGCCTCGGCAG

GCCGGGGGGCCCCTCCTGACGAGGAGCAGGCTCGCCGGCGGCCAAGTTAACTCTTGTTTT

TATTGTGAAACTCTAAATGAATCAAAACTTTCAACAACGGATCTCTTGGTTCTGGCATCG

ATGAAGAACGCAGCGAAATGCGATAAGTAATGTGAATTGCAGAATTCAGTGAATCATCGA

ATCTTTGAACGCACATTGCGCCCTCTGGTATTCCGGAGGGCATGCCTGTTCGAGCGTCAT

TTCAACCCTCAAGCCTGGCTTGGTGATGGGGCACTGCGAGGCAGGCCCTGAAATTCAGTG

GCGAGCTCGCCAGGACTCCGAGCGCAGTAGACCCTCGCTAAGG-ACTGGCGGTGCCCTGC

TGAAAGAGAAGGAAGGTTAGTAAACATCCTGGCGTTCCCACGGCGTCATCAGCGCATTTT

CACCCCTCGCTCTGGATTTTCAGGGTGCGGGGCTTAGAGCTTATCTCACC---------T

TGCACATGCTGACATCTTCACAGCCGCTGAGCTCGGTAAGGGNNNNNNNNNNNNNNNNNN

NNNNNNNNNNNNNNNNNNNNNNNNNNNNNNNNNNNNNNNNNNNNNNNNNNNNNNNNNNNN

NNNNNNNNNNNNNNNNNNNNNNNNNNNNNNNNNNNNNNNNNNNNNNNNNNNNNNNNNNNN

NNNNNNNNNNNNNNNNNNNNNNNNNNNNNNNNNNNNNNNCGGTGCTGCTTTCTGGTGCGT

C----GTCCATCACCGCGACACGACCTCGCAACATACTGACCTCGTAGGCAAACCATCTC

TGGCGAGCACGGCCTCGACACCAATGGCGTGTATGCACCTCCTATTCCTGCCCATCTCGG

CTTAGCTGACAATTGCACAGCTACAACGGCACTTCCGAGCTCCAGCTCGAGCGCATGAAC

GTCTACTTCAACGAGGTAAGTATGACATTTTGACGAGCTCCCGCTTTGCTGACCGCTTAT

CAGGCTTCCGGCAACAAGTATGTTCCTCGCGCCGTCCTCGTCGATCTCGAGCCCGGTACC

ATGGACGCCGTC

>Diaporthe_fusiformis_JZB320156

NNNNNNNNNNNNNNNNNNNNNNNNNNGCTCTTGCTGTTGCGCATGCTAACGGACCGTTTT

CGGCTTGTAGGATAAGGATGGCGATGGTTAGTGCGGCCGCTCTCACACAGCACGCGTCAC

GCTCGATCCGCCGCGACGGCCTGCGCGCAACCAAGCGTTATCACTATTGCGAGTTGCTGA

GGTGTAGGACAAATCACCACCAAGGAGCTCGGCACGGTCATGCGATCCCTGGGACAGAAC

CCGTCCGAGTCTGAGCTGCAAGACATGATTAACGAGGTCGACGCCGACAACAACGGCACC

ATTGACTTTGGTACGTCCAGAAGCACGCCCG?????????????????????????????

????????????????????????????????????????????????????????????

????????????????????????????????????????????????????????????

????????????????????????????????????????????????????????????

????????????????????????????????????????????????????????????

???????????????????????????AACAAGGTCTCCGTTGGTGAACCAGCGGAGGGA

TCATTGCTGCCCCAGGCGCACCCAGAAACCCTTTGTGAACTTTTACTGTTGCCTCGGCAT

GCTGGGGGGTCCCTCCCGGTGAGGAGCAGGCACGCCGGCGGCCAAGTTAACTCTTGTTTT

TACACTGAAACTCTAAATGAATCAAAACTTTCAACAACGGATCTCTTGGTTCTGGCATCG

ATGAAGAACGCAGCGAAATGCGATAAGTAATGTGAATTGCAGAATTCAGTGAATCATCGA

ATCTTTGAACGCACATTGCGCCCTCTGGTATTCCGGAGGGCATGCCTGTTCGAGCGTCAT

TTCAACCCTCAAGCATTGCTTGGTGTTGGGGCACTGAGAAGCAGGCCCTGAAATCTAGTG

GCGAGCTCGCCAGGACCCCGAGCGCAGTAGACCCTCGCTAAGGCCCTGGGGGTGCCCTGC

NNNNNNNNNNNNNNNNNNNNNNNNNNNCATCATGTTGGTACGGCTTCGTCAGCGCATTTT

CACCCCTCGCTCTGGA-TTTCAGGGTGCGGGGCTTAGAGCTTATCT-----ATGCCGTTC

GAAACATGCTGATATCTACACAGCCGCCGAGCTTGGTAAGGGNNNNNNNNNNNNNNNNNN

NNNNNNNNNNNNNNNNNNNNNNNNNNNNNNNNNNNNNNNNNNNNNNNNNNNNNNNNNNNN

NNNNNNNNNNNNNNNNNNNNNNNNNNNNNNNNNNNNNNNNNNNNNNNNNNNNNNNNNNNN

NNNNNNNNNNNNNNNNNNNNNNNNNNNNNNNNNNNNNNNNNNNNNNNNNNNNNNNNCA--

-----GTCCACCACCGCGATACGACCTCGAGCCATACTGACATCGCAGGCAAACCATCTC

TGGCGAGCACGGCCTCGACAGCAATGGCGTGTATGCACCTCCTATTCCTACTCTTCTTGT

CTGCCCTGACAATCACACAGTTACAACGGCTCTTCCGAGCTCCAGCTCGAGCGCATGAAC

GTCTACTTCAACGAGGTAAGTACGTCG--TTGACCATCTGCAGCCTTGCTAACGCGTTAT

CAGGCCTCCGGCAACAAGTATGTCCCTCGCGCCGTCCTCGTCGATCTCGAGCCCGGTACC

ATGGACGCCGTC

>Diaporthe_ganjae_CBS_180_91

CTTTGTAAGTTATATCCGCCTTGCCCGCCGTTGCTGTTGCGCATGCTAACGGACCGTTTT

CGGCCTGCAGGATAAGGATGGCGATGGTTAGTGTGGTCACCCTCAGCCAGCACGCGTCAT

GCTCGATCCGCCGCGACGGTCTGCGCGAGAGCGATCGTCATCACCATGACGAGCTGCTAA

GGTGCAGGACAAATCACCACCAAGGAGCTCGGCACGGTCATGCGGTCCCTGGGCCAAAAC

CCCTCCGAGTCCGAGCTGCAGGACATGATCAACGAGGTCGACGCCGACAACAACGGCACC

ATTGACTTTGGTAGGTCCACATGCCCTCCCATCCGCGCCCTCCACCGGAGGTGTCAAGAA

GCCTCACCGCTACAAGCCTGGTACCGTCGCTCTGCGTGAGATCCGTCGCTACCAGAAGAG

CACCGAGCTGCTCATCCGCAAGCTCCCCTTCCAGCGTCTGGTATGCAGGTTCGTGAGATC

GCCCAGGACTTCAAGTCCGACCTGCGCTTCCAGTCTTCCGCCATCGGTGCCCTTCAGGAG

TCCGTCGAGTCTTACCTCGTCTCCCTCTTTGAGGACACCAACCTGTGCGCCATCCACGCC

AAGCGTGTCACCATCCAGTCGGTACGTAACAAGGTCTCCGTTGGTGAACCAGCGGAGGGA

TCATTGCTGCCCC-GGCGCACCCAGAAACCCTTTGTGAACCTA--CTGTTGCCTCGGCAG

GCCGGCAGACCCCCTGAGACAGGGAGCA-GCCCGCCGGCGGCCGACCAAACTC-CGTTTC

TATAGTGAATCTCTAAATGAATCAAAACTTTCAACAACGGATCTCTTGGTTCTGGCATCG

ATGAAGAACGCAGCGAAATGCGATAAGTAATGTGAATTGCAGAATTCAGTGAATCATCGA

ATCTTTGAACGCACATTGCGCCCTCTGGTATTCCGGAGGGCATGCCTGTTCGAGCGTCAT

TTCAACCCTCAAGCCTGGCTTGGTGATGGGGCAGTGCAAGGCACGCCCTGAAATTCAGTG

GCGAGCTCGCCAGGACCCCGAGCGTAGTAGA-TCTCGCTAAGGCCCTGGCGGTGCCCTGC

TGAAANNNNNNNNNNNNNNNNNNNNNNCATCGCGTCTGCGCCGCTTCGT--CAGCATTTT

CACCCCTCGTCCTGGACTTTCAGGGTGCGGGGCTTACCGCTTATCGCGCTGCCCACATTG

AAAACATGCTGATTTCCATACAGCCGCCGAGCTGGGTAAGGGTCGCACCTCAGCCCCACC

ATCGCGACCCTCCCCTTGTGACACCCGTGTAACACGCGTCGATTGCTAACATGTTTTTCT

GGCCTGCAGGTTCACCTCCAGACCGGCCAATGCGTAAGTTGCTGTCACCGCCCGACCTTA

T---CGCCACCCGTAGCACGTTTCCCAGGGTAACCAAATCGGTGCTGCTTTCTGGTGCGT

CCGAGGCCTACCGCCGCGAGACCAGCTCGCAACATACTGACCTAGTAGGCAAACCATCTC

TGGCGAGCACGGTCTCGACAGCAATGGCGTGTATGCACCTCCTATTCCTGCCCATCTCGT

CCTCCCTGATGATTGCACAGTTACAACGGCACTTCCGAGCTCCAGCTCGAGCGCATGAAC

GTCTACTTCAACGAGGTATGTACGTCGTTTTGCCCATCTACTGCCTTGCTAACGCGTTGT

CAGGCTTCCGGCAACAAGTATGTGCCCCGCGCTGTCCTCGTCGATCTCGAGCCCGGTACC

ATGGACGCCGTC

>Diaporthe_ganzhouensis_CFCC_53087

CTTTGTAATCTCCAGCCGACACGCCCGCCCTTGCTGGTGCGCATGCTAACGGACCGTTTT

CGGCTTGTAGGATAAGGATGGCGATGGTTAGTGCGGCCGATCTCACCTAGCACGCGTCAT

GATCGATCCGCCGCGACAGCCTGCGCGCAACCACGCGTTATCACTATCACGAGTTGCTGA

GGTGTAGGACAAATCACCACCAAGGAGCTCGGCACGGTCATGCGGTCCCTTGGTCAGAAC

CCGTCCGAGTCTGAGCTGCAAGATATGATCAACGAGGTCGACGCCGACAACAATGGCACC

ATTGACTTTGGTACGTCCAGATGCTCGCCCTTCCGCGCCCTCCACCGGAGGTGTCAAGAA

GCCTCACCGCTACAAGCCTGGTACCGTCGCTCTGCGTGAGATCCGTCGCTACCAGAAGAG

CACTGAGCTGCTGATCCGCAAGCTGCCCTTCCAGCGTCTGGTATGCAGGTCCGTGAGATC

GCCCAGGACTTCAAGTCCGACCTCCGCTTCCAGTCTTCCGCCATCGGCGCCCTGCAGGAG

TCGGTCGAGTCTTACCTCGTCTCCCTCTTCGAGGACACCAACCTGTGCGCCATCCACGCC

AAGCGTGTCACCATCCAGTCGGTATGTNNNNNNNNNNNNNNNNNNNNNNNNNNNNNNNNN

NNNNNNNNNNNNNNNNNNNNNNNNNNNNCCCTTTGTGAACTTTTACTTTTGCCTCGGAAG

GCTGGGGGGCCCCTCCGGGTGTTGAGACAGCCCGCCGGCGGCCAAGCTAACTCTTGTTTT

TACACTGAAACTCTAAATGAATCAAAACTTTCAACAACGGATCTCTTGGTTCTGGCATCG

ATGAAGAACGCAGCGAAATGCGATAAGTAATGTGAATTGCAGAATTCAGTGAATCATCGA

ATCTTTGAACGCACATTGCGCCCTCTGGTATTCCGGAGGGCATGCCTGTTCGAGCGTCAT

TTCAACCCTCAAGCCTGGCTTGGTGATGGGGCACTGAGAAGCAGGCCCTGAAATTCAGTG

GCGAGCTCGCCAGGACCCCGAGCGCAGTAGACCCTCGCTAAGGCCCTGGCGGTGNNNNNN

NNNNNNNNNNNNAAGGTTAGTAAACACCATGATGCTCGTGTGGCTTCGTCAGCGCATTTT

CACCCCTCGCTTTGGATTTTCAGGGTGCGGGGCTTAGGGCTTATCTCACCACCACCACCG

AACACATGCTGATATCTACACAGCCGCCGAGCTTGGTAANNNTCGCACCTGAGCCCCACC

ATCGCGACCCACCCCCTGGGACACCCAGATAAGACGCGTCGATTGCTAACGTGTTTTTCT

CGCCTGCAGGTTCACCTTCAGACCGGCCAATGCGTAAGTTGCTGTCAC-AGCGGACCTTA

TCATCGCCACCTGTAGCACGTTTCCCAGGGTAACCAAATCGGTGCTGCTTTCTGGTGCGT

CCCAATTCCACCGCCGCGATACGACCTCGAAACATGCTGACCTCGTAGGCAAACCATCTC

TGGCGAGCACGGCCTCGACAGCAATGGCGTGTATGCACCTCCTATTCCTGCCCATCTTGG

TTTCCCTGACAATTGCGCAGTTACAACGGCACTTCTGAGCTCCAGCTCGAGCGCATGAAC

GTCTACTTCAACGAGGTAAGTACAGCAATTTGACCATCTGTAACC-TGCTAACGCGTTAT

CAGGCCTCCGGCAACAAGTATGTTCCTCGCGCCGTTCTCGTCGATCTCGAGCCCGGTACC

ATGGACGCCGTN

>Diaporthe_gardeniae_CBS_288_56

CTTTGTAACCTCTAGCCGACATGCCCGCCCTTGCTGGTGCGCATGCTAACGGACCGTTTT

CGGCTTGTAGGATAAGGATGGCGATGGTTAGTGCGGCCGCTTTCACCTAGCACGCGTCAT

GCTCGATGCGCCGCGACAGCCTGCGTGCAACCAAGCGTTATCACTATCACGAGTTGCTGA

GGTGTAGGACAAATCACCACCAAGGAGCTCGGCACGGTCATGCGGTCCCTTGGTCAGAAC

CCGTCCGAGTCTGAGCTGCAAGATATGATTAACGAAGTCGACGCCGACAACAATGGCACC

ATTGACTTTGGTACGTCTATATGCTCGCCCTTCCGCGCCCTCCACCGGAGGTGTCAAGAA

GCCTCACCGCTACAAGCCTGGTACCGTCGCTCTGCGTGAGATCCGTCGCTACCAGAAGAG

CACCGAGCTGCTGATCCGCAAGCTCCCCTTTCAGCGTCTGGTATGCAGGTCCGTGAGATC

GCCCAGGACTTCAAGTCCGACCTCCGCTTCCAGTCTTCCGCCATCGGTGCTCTGCAGGAG

TCCGTCGAGTCTTACCTCGTCTCCCTCTTCGAGGACACCAACCTGTGCGCCATCCATGCC

AAGCGTGTCACCATCCAGTCGGTACGTAACAAGGTCTCCGTTGGTGAACCAGCGGAGGGA

TCATTGCTGCCCCAGGCGCACCCAGAAACCCTTTGTGAACTTTTGCTGTTGCCTCGGCCA

GCTGGGGGGTCCCTCAGGGTGTTGAGAGAGCACGCCGGCGGCCAACCCAACTCTTGTTTT

TACACTGAAACTCTAAATGAATCAAAACTTTCAACAACGGATCTCTTGGTTCTGGCATCG

ATGAAGAACGCAGCGAAATGCGATAAGTAATGTGAATTGCAGAATTCAGTGAATCATCGA

ATCTTTGAACGCACATTGCGCCCTCTGGTATTCCGGAGGGCATGCCTGTTCGAGCGTCAT

TTCAACCCTCAAGCCTGGCTTGGTGATGGGGCACTGAAGGGCAGGCCCTGAAATTCAGTG

GCGGGCTCGCCAGGACCCCGAGCGCAGTAGACCCTCGCTAAGGCCCTGGCGGTGCCCTGC

TGAAAGAGAAGGAAGGTTAGTAAA---CATCATGCTCGCGCGGCTTCGTCAGCGCATTTT

CACCCCTCGTTCTGGATTTTCAGGGTGCGGGGCTTAGTGCTTATCTCAAAACCACTACCG

AATGCATGCTGACATCTCCATAGCCGCCGAGCTTGGTAAGGGTCGCACCTGAGCCCCACC

ATCGCGACCCACCCCCCGGGACACCCAGATAAGACGCGTCTATTGCTAACATGTTTTTCT

CGCCTACAGGTTCACCTTCAGACCGGCCAATGCGTAGGTTGCTGTCAT-GGCGGACCTTA

TCATCGCCACCTGTAGCACGTTTCCCAGGGTAACCAAATCGGTGCTGCTTTCTGGTGCGT

CCCAAGTCCAGCGCCGCGATACGACCTCGAAGCATACTGACCTCGTAGGCAAACCATCTC

TGGCGAGCACGGCCTCGACAGCAATGGCGTGTATGCACCTCCTATTCCTGCCCATCTTGG

CTTCCCTGACAATTGCAAAGTTACAACGGCACTTCTGAGCTCCAGCTCGAGCGCATGAAC

GTCTACTTCAACGAGGTAAGTACATCATTCCGACCATCCTCAACCTTGCTAACGCGTTAT

CAGGCTTCCGGCAACAAGTATGTTCCTCGCGCCGTCCTCGTCGATCTCGAGCCCGGTACC

ATGGACGCCGTC

>Diaporthe_garethjonesii_MFLUCC_12_0542a

NNNNNNNNNNNNNNNNNNNNNNNGCAGCCCTCACTGTCGCGCATGCTAACGGACCGTTCT

CGGCCTCCAGGATAAGGATGGCGATGGTTAGTGCAGCTGCTCCCAGCTTGTACGCGTCAC

GATCGACCCGCCGCGACGGCTTGCGCGCAACCAAGC-CCATAACTACTAGGAGCTGCTGA

GGTGTAGGACAAATCACCACAAAGGAGCTCGGCACGGTCATGCGATCTCTGGGTCAGAAC

CCGTCCGAGTCTGAGCTGCAGGATATGATCAACGAGGTCGACGCCGACAATAATGGAACC

ATCGACTTTGGTACGTCCAGATGCTCGCTCG?????????????????????????????

????????????????????????????????????????????????????????????

????????????????????????????????????????????????????????????

????????????????????????????????????????????????????????????

????????????????????????????????????????????????????????????

???????????????????????????NNNNNNNNNNNNNNNNNNNNNNNNNCGGAGGGA

TCATTGCTGCCTC-GGCGCACCCAGAAACCCTTTGTGAACTTTTACTGTTGCCTCGGCAG

GCCGGGGGGCCCCTCCTGACGAGGAGCAGGCTCGCCGGCGGCCAAGTTAACTCTTGTTTT

TATTGTGAAACTCTAAATGAATCAAAACTTTCAACAACGGATCTCTTGGTTCTGGCATCG

ATGAAGAACGCAGCGAAATGCGATAAGTAATGTGAATTGCAGAATTCAGTGAATCATCGA

ATCTTTGAACGCACATTGCGCCCTCTGGTATTCCGGAGGGCATGCCTGTTCGAGCGTCAT

TTCAACCCTCAAGCCTGGCTTGGTGATGGGGCACTGGAAGGCAGGCCCTGAAATTCAGTG

GCGAGCTCGCCAGGACTCCGAGCGCAGTAGACCCTCGCTAAGG-ACTGGCGGTGCCCTGC

TGAAAGAGAAGGAAGGTTAGTAAACATTCTGGCGTTTCCACGGCGTCATCAGCGCATTTT

CACCCCTCGCTTTGGATTTTCAGGGTGCGGGGCTTAGAGCTTATCTCGCC---------T

TGGACATGCTGACATCTTCACAGCCGCTGAGCTCGGTAAGGGNNNNNNNNNNNNNNNNNN

NNNNNNNNNNNNNNNNNNNNNNNNNNNNNNNNNNNNNNNNNNNNNNNNNNNNNNNNNNNN

NNNNNNNNNNNNNNNNNNNNNNNNNNNNNNNNNNNNNNNNNNNNNNNNNNNNNNNNNNNN

NNNNNNNNNNNNNNNNNNNNNNNNNNNNNNNNNNNNNNNNGGTGCTGCTTTCTGGTGCGT

C----GTCCATCACCGCGACACGACCTCGCAACATACTGACCTCGTAGGCAAACCATCTC

TGGCGAGCACGGCCTCGACACCAATGGCGTGTATGCACCTCCTATTCCTGCCCATCTCGG

CTCGGCTGACAATTGCACAGCTACAACGGCACTTCCGAGCTCCAGCTCGAGCGCATGAAC

GTCTACTTCAACGAGGTAAGTATGACATTTTGACGAGCTCCCGCTTTGCTGACCGCTTAT

CAGGCTTCCGGCAACAAGTATGTTCCTCGCGCCGTCCTCGTCGATCTCGAGCCCGGTACC

ATGGACGCCGTC

>Diaporthe_globoostiolata_MFLUCC_23_0025

????????????????????????????????????????????????????????????

????????????????????????????????????????????????????????????

????????????????????????????????????????????????????????????

????????????????????????????????????????????????????????????

????????????????????????????????????????????????????????????

????????????????????????????????????????????????????????????

????????????????????????????????????????????????????????????

????????????????????????????????????????????????????????????

????????????????????????????????????????????????????????????

????????????????????????????????????????????????????????????

???????????????????????????AACAAGGTCTCCGTTGGTGAACCAGCGGAGGGA

TCATTGCTGCCCCAGGCGCACCCAGAAACCCTTTGTGAACTTTTACTGTTGCCTCGGCAT

GCCGGGGGGCCCCTCGAGACGAGGAGCAGGCACGCCGGCGGCCAAGTTAACTCTTGTTTT

TACACTGAAACTCTAAATGAATCAAAACTTTCAACAACGGATCTCTTGGTTCTGGCATCG

ATGAAGAACGCAGCGAAATGCGATAAGTAATGTGAATTGCAGAATTCAGTGAATCATCGA

ATCTTTGAACGCACATTGCGCCCTCTGGTATTCCGGAGGGCATGCCTGTTCGAGCGTCAT

TTCAACCCTCAAGCATTGCTTGGTGTTGGGGCACTGAAGGGCAGGCCCTGAAATCTAGTG

GCGAGCTCGCCAGGACCCCGAGCGCAGTAGACCCTCGCTAAGGCCCTGGCGGTGCCCTGC

TGAAAGAGAAGGAAGGTTAGTAAACATCATCATGTTGGTACGGCTTCGTCAGCGCATTTT

CACCCCTCGCTCTGGA-TTTCAGGGTGCGGGGCTTAGAGCTTATCT-----ATGCCGTTC

GAAACATGCTGATATCTACACAGCCGCCGAGCTTGGTAAGGGNNNNNNNNNNNNNNNNNN

NNNNNNNNNNNNNNNNNNNNNNNNNNNNNNNNNNNNNNNNNNNNNNNNNNNNNNNNNNNN

NNNNNNNNNNNNNNNNNNNNNNNNNNNNNNNNNNNNNNNNNNNNNNNNNNNNNNNNNNNN

NNNNNNNNNNNNNNNNNNNNNNNNNNNTGGTAACCAAATCGGTGCTGCTTTCTGGTGCGT

CC-AAGTCCACCGCCGCGATACGACCTCGAACCATACTGACCTCGCAGGCAAACCATCTC

TGGCGAGCACGGCCTCGACAGCAATGGCGTGTATGCACCTCCTATTCCTACCTCTCTCGT

CTGCCCTGACAATCACACAGTTACAACGGCTCTTCCGAGCTCCAGCTCGAGCGCATGAAC

GTCTACTTCAACGAGGTAAGTACGTCG--TTGACCATCTGCAGCCTTGCTAACGCGTTAT

CAGGCCTCCGGCAACAAGTATGTTCCTCGCGCCGTCCTCGTCGATCTCGAGCCCGGTACC

ATGGACGCCGTC

>Diaporthe_gossiae_BRIP_59730a

????????????????????????????????????????????????????????????

????????????????????????????????????????????????????????????

????????????????????????????????????????????????????????????

????????????????????????????????????????????????????????????

????????????????????????????????????????????????????????????

????????????????????????????????????????????????????????????

????????????????????????????????????????????????????????????

????????????????????????????????????????????????????????????

????????????????????????????????????????????????????????????

????????????????????????????????????????????????????????????

???????????????????????????AACAAGGTCTCCGTTGGTGAACCAGCGGAGGGA

TCATTGCTGCCCCAGGCGCACCCAGAAACCCTTTGTGAACTTTT-TTGTTGCCTCGGCA-

GCTGGTAGACCCTTTATGGTAAAGAGACGGCACGCCGGCGGCCAAGTTAACTCTTGTTTT

TACACTGAAACTCTAAATGAATCAAAACTTTCAACAACGGATCTCTTGGTTCTGGCATCG

ATGAAGAACGCAGCGAAATGCGATAAGTAATGTGAATTGCAGAATTCAGTGAATCATCGA

ATCTTTGAACGCACATTGCGCCCTCTGGTATTCCGGAGGGCATGCCTGTTCGAGCGTCAT

TTCAACCCTCAAGCATTGCTTGGTGTTGGGGCACTGCCAAGCAGGCCCTGAAATCTAGTG

GCGAGCTCGCCAGGACCCCGAGCGCAGTAGACCCTCGCTAAGGCCCTGGCGGTGCCCTGC

NNNNNGAGAAGGAAGGTTAGTAAACATCATCATGTTGGTACAGCTTCGTCAGCGCATTTT

CACCCCTCGCTCTGGA-TTTCAGGGTGCGGGGCTTAGAGCTTATCTACCA-CCACTTCAG

GAAACATGCTGATATCTACATAGCCGCCGAGCTTGGTAANNNTCGCACCTGAGCCCCACC

ATCGCGACCCACCCCCTGGGACACCCAGATAAAACGCGTCGATTGCTAACGTGTTTTTCT

CGACTACAGGTTCACCTTCAGACCGGCCAATGCGTAAGTTGCTGTCACGACGGA-CCTTA

TCATCGCCACCCGTAGCACGTTTCCCAGGGTAACCAAATCGGTGCTGCTTTCTGGTGC--

-----GTCCACCACCGCGATACGACCTCGAACCATACTGACCTCGCAGGCAAACCATCTC

TGGCGAGCACGGCCTCGACAGCAATGGCGTGTATGCACCTCCTATTCCTACATCACTCGT

CTGCCCTGACAATCACATAGTTACAACGGCACTTCCGAGCTCCAGCTCGAGCGCATGAAC

GTCTACTTCAACGAGGTAAGTACGTCG--TTGGCCATCTGCAGCCTTGCTAACGCGTTAT

CAGGCCTCCGGCAACAAGTATGTCCCTCGCGCCGTCCTCGTCGATCTCGAGCCCGGTACC

ATGGACGCCGTC

>Diaporthe_goulteri_BRIP_55657a

????????????????????????????????????????????????????????????

????????????????????????????????????????????????????????????

????????????????????????????????????????????????????????????

????????????????????????????????????????????????????????????

????????????????????????????????????????????????????????????

????????????????????????????????????????????????????????????

????????????????????????????????????????????????????????????

????????????????????????????????????????????????????????????

????????????????????????????????????????????????????????????

????????????????????????????????????????????????????????????

???????????????????????????AACAAGGTCTCCGTTGGTGAACCAGCGGAGGGA

TCATTGCTGCCCCTGGCGCACCCAGAAACCCTTTGTGAACTTA--CCGTTGCCTCGGCAG

GCCGGGGGGCCCCCCGAGACGGGGAGCA-GCCCGCCGGCGGCCAACCAAACTCTTGTTTC

T-TAGTGAATCTCTAAATGAATCAAAACTTTCAACAACGGATCTCTTGGTTCTGGCATCG

ATGAAGAACGCAGCGAAATGCGATAAGTAATGTGAATTGCAGAATTCAGTGAATCATCGA

ATCTTTGAACGCACATTGCGCCCTCTGGTATTCCGGAGGGCATGCCTGTTCGAGCGTCAT

TTCAACCCTCAAGCCTGGCTTGGTGATGGGGCACTGGAGGGCAGGCCCTGAAATCTAGTG

GCGAGCTCGCTAGGACCCCGAGCGTAGTAGA-TCTCGTTAAGGCCCTGGCGGTGCCCTGC

TGAAAGAGAAGGAAGGTTAGTAAAAACTACTG--CTGTGGCGGCTTCGTCAAGCCATTTT

CACCCCTCCCTCTGGATTTTCAGAGTGCGGGCCTTACGGCCTATCTAGCAAACACCACCG

AATACATGCTGATGAGTATACAGCCGCCGAGCTGGGCAAGGGNNNNNNNNNNNNNNNNNN

ATCGCGACCCACCCCCTTGGACACCCAGGTAAGACGCGTCGATTGCTAACGTGTTTCTCT

CGCCCACAGGTTCACCTCCAGACCGGCCAGTGCGTAAGTTGCTGTCACACCCGGACCTTA

T---CGCCACCTGTAGCACGTTTCCCAGGGTAACCAAATCGGTGCTGCTTTCTGGTGCGT

CCCAAGCCTACGACCGCGATACCAGCTCGCATCATACTGACCTCATAGGCAAACCATCTC

TGGCGAGCACGGTCTCGACAGCAATGGCGTGTATGCACCTCCTATCCCCATCCATCTCGT

TCTCCCTGACAATCGCACAGCTACAATGGCACCTCCGAGCTCCAGCTCGAGCGCATGAAC

GTCTACTTCAACGAGGTAAGTACTTCGTTTTGATCACCTGCGGCCCTGCTAACGCGTTCT

CAGGCCTCCGGCAACAAGTATGTGCCTCGCGCCGTCCTCGTCGATCTCGAGCCCGGTACC

ATGGACGCCGTC

>Diaporthe_grandiflori_SAUCC194_84

NNNNNCCACTCTCAGCCGACATGCCCGCCCTTGCTGGTGCGCATGCTAACGGACCGTTTT

CGGCTTGTAGGATAAGGATGGCGATGGTTAGTGCGGCCGCTCTCACATAGCACGCGTCAT

GTTCGATCCGCCGCGACAGTCTGCGCGCAGCCAAGCGTTATCACTATCACGAGTTGCTGA

GGTGTAGGACAAATCACCACCAAGGAGCTCGGCACGGTCATGCGATCCCTGGGCCAGAAC

CCGTCTGAGTCCGAGCTGCAAGATATGATTAACGAGGTCGACGCCGACAACAATGGCACC

ATTGACTTTGGTACGTCCAGATGCTTGCGCTTCCGCGCCCTCTACCGGAGGTGTCAAGAA

GCCTCACCGCTACAAGCCTGGTACCGTCGCTCTGCGTGAGATCCGTCGCTACCAGAAGAG

CACCGAGCTGCTGATCCGCAAGCTCCCCTTCCAGCGTCTGGTATGCAGGTCCGTGAGATC

GCCCAGGACTTCAAGTCCGACCTCCGCTTCCAGTCTTCCGCCATCGGTGCACTGCAGGAG

TCGGTCGAGTCTTACCTCGTCTCCCTCTTCGAGGACACCAACCTGTGCGCCATCCACGCC

AAGCGTGTCACCATCCAGTCGGTATGTNNNNNNNTCTCCGTTGGTGAACCAGCGGAGGGA

TCATTGCTGCCCCAGGCGCACCCAGAAACCCTTTGTGAACTTTTACTGTTGCCTCGGCTA

GCTGGGGGGCCCATTAGGGTATTGAGACGGCACGCCGGCGGCCAAGTTAACTCTTGTTTT

TACACTGAAACTCTAAATGAATCAAAACTTTCAACAACGGATCTCTTGGTTCTGGCATCG

ATGAAGAACGCAGCGAAATGCGATAAGTAATGTGAATTGCAGAATTCAGTGAATCATCGA

ATCTTTGAACGCACATTGCGCCCTCTGGTATTCCGGAGGGCATGCCTGTTCGAGCGTCAT

TTCAACCCTCAAGCCTGGCTTGGTGATGGGGCACTGGAGGGCAGGCCCTGAAATTCAGTG

GCGAGCTCGCCAGGACCCCGAGCGCAGTAGACCCTCGCTAAGGCCCTGGCGGTGCCCTGC

TGAAANNNNNNNNNNGCGTTTACGCATCATCATGCTCGCGCGGCTTCGTCAGCGCATTTT

CACCCCTCGCTTTGGATTTTCAGGGTGCGGGGCTTAGGGCTTATCTGACCGCCCCCACCG

AATTCATGCTGATATTTCCACAGCCGCCGAGCTTNNNNNNNNNNNNNNNNNNNNNNNNNN

NNNNNNNNNNNNNNNNNNNNNNNNNNNNNNNNNNNNNNNNNNNNNNNNNNNNNNNNNNNN

NNNNNNNNNNNNNNNNNNNNNNNNNNNNNNNNNNNNNNNNNNNNNNNNNNNNNNNNNNNN

NNNNNNNNNNNNNNNNNNNNNNNNNNNNNNNNNNNNNNNNNNNNNNNNNNNNNNNNNNNN

NCTCAGTCCACCGCCGCGATACGACCTCGAAGCATACTGACCTCGTAGGCAAACCATCTC

TGGCGAGCACGGCCTCGACAGCAATGGCGTGTATGCACCTCCTATTCCTGCCCATCTTGG

CTTCCCTGACAATTGCACAGTTACAACGGCACTTCTGAGCTCCAGCTCGAGCGCATGAAC

GTCTACTTCAACGAGGTAAGTACATCATTCCGACCATCTCCAACCTTGCTAACGCGTTAT

CAGGCCTCCGGCAACAAGTATGTTCCCCGCGCCGTCCTCGTCGATCTCGAGCCCGGTACC

ATGGACGCCGTC

>Diaporthe_griceae_BRIP_67014a

????????????????????????????????????????????????????????????

????????????????????????????????????????????????????????????

????????????????????????????????????????????????????????????

????????????????????????????????????????????????????????????

????????????????????????????????????????????????????????????

????????????????????????????????????????????????????????????

????????????????????????????????????????????????????????????

????????????????????????????????????????????????????????????

????????????????????????????????????????????????????????????

????????????????????????????????????????????????????????????

???????????????????????????AACAAGGTCTCCGTTGGTGAACCAGCGGAGGGA

TCATTGCTGCCTC-GGCGCACCCAGAAACCCTTTGTGAACTTA--TTGTTGCCTCGGCAG

GCCGGGAGGCCCCCTGAGACAGGGAGCA-GCCCGCCGGCGGCCAGCTAAACTCTTGTTTC

TACAGTGAATCTCTAAATGAATCAAAACTTTCAACAACGGATCTCTTGGTTCTGGCATCG

ATGAAGAACGCAGCGAAATGCGATAAGTAATGTGAATTGCAGAATTCAGTGAATCATCGA

ATCTTTGAACGCACATTGCGCCCTCTGGTATTCCGGAGGGCATGCCTGTTCGAGCGTCAT

TTCAACCCTCAAGCCTGGCTTGGTGATGGGGCACTGAAGGGCAGGCCTTGAAATCTAGTG

GCGAGCTCGCCAGGACCCCGAGCGTAGTAGA-TCTCGCCAAGGCCCTGGCGGTGCCCTGC

TGAAANAGAAGGAAGGTTAGTAAACATCGTTTACACTGCGAGGCTTCGTCAAGGCATTTT

CACCCCTCTCTCTGGATTTTCAGGGTGCGGGGCTTACCGCTTATCTCAGTGCC-ACCCCG

AAAACATGCTGATTTCTACACAGCCGCCGAGCTGGGCAAGGGTCGCACCTCAGCCCCACC

ATCGCGGCCCACCACTTGCGACACCCA---GAGACGCGTCGATTGCTAACATGTTTTTCT

TGCCCACAGGTTCACCTTCAGACCGGCCAATGCGTAAGTTGCTGTCACCGTCGGACCTTC

T---CGCCACCCGTAGCACGTTTCCCAGGGTAACCAAATCGGTGCTGCTTTCTGGTGCGT

CCTCTGCCTACCACCGCGAGGCTGGCTCGTAGTCTACTGACCTCGTAGGCAAACCATCTC

TGGCGAGCACGGTCTCGACAGCAATGGCGTGTATGCACCTCCTATTCCTGACTCCCTCGT

CCTCCCTGATGATCGCACAGTTACAACGGCACCTCCGAGCTCCAGCTCGAGCGCATGAAC

GTCTACTTCAACGAGGTAAGCACGTCACTTTGA-CCGTCCCGGACTTGCTAACGCGTTCT

CAGGCCTCCGGCAACAAGTATGTGCCCCGCGCCGTCCTCGTCGATCTCGAGCCCGGTACC

ATGGACGCCGTC

>Diaporthe_guangdongensis_ZHKUCC20_0014

CTTTGTAAGTCATCTTCGCCTTGCCCGCCGTTGCCGTTGCGCATGCTAACGGACCGTTTT

CGGCCTGCAGGATAAGGATGGCGATGGTTAGTGCGGTCACCCTCAGCTAGCACGCGTCAC

ACTCGATCCGCCGCGACGGTCTGCGCGCGAGCGACCGTCATCACCATCACGAGTTGCTAA

GGTGTAGGACAAATCACCACCAAGGAGCTCGGCACAGTCATGCGGTCCCTTGGTCAAAAC

CCTTCCGAGTCCGAGCTGCAGGACATGATCAACGAGGTCGACGCCGACAACAATGGCACC

ATTGACTTTGGTGAGTCTA-ATTCTGGCACA?????????????????????????????

????????????????????????????????????????????????????????????

????????????????????????????????????????????????????????????

????????????????????????????????????????????????????????????

????????????????????????????????????????????????????????????

???????????????????????????NNNNNNNNNTCCGTAGGTGAACCTGCGGAGGGA

TCATTGCTGCCTC-GGCGCACCCAGAAACCCTTTGTGAACTTA--CTGTTGCCTCGGCAG

GCCGGAAGGCCCCCTGGAACAGGGAGCA-GCCCGCCGGCGGCCAACTAAACTCTTGTTTC

TATAGTGAATCTCTAAATGAATCAAAACTTTCAACAACGGATCTCTTGGTTCTGGCATCG

ATGAAGAACGCAGCGAAATGCGATAAGTAATGTGAATTGCAGAATTCAGTGAATCATCGA

ATCTTTGAACGCACATTGCGCCCTCTGGTATTCCGGAGGGCATGCCTGTTCGAGCGTCAT

TTCAACCCTCAAGCCTGGCTTGGTGATGGGGCACTGGGGAGCAGGCCCTGAAATCTAGTG

GCGAGCTCGCCAGGACCCCGAGCGTAGTAGA-TCTCGCTAAGGCCCTGGCGGTGCCCTGC

TGAAAGAGAAGGAAGGTTAGTAAACATCGTTGTGTCTGCGAGGCTTCGTCAAGGCATTTT

CACCCCTCCCTCCGGATTTTCAGGGTGCGGGGCTTACCGCTTATCTCTGTGAC-GCACCG

GAAGCATGCTGATTTCTGTACAGCCGCCGAGCTGGGTAAGGGNNNNNNNNNNNNNNNNNN

NNNNNNNNNNNNNNNNNNNNNNNNNNNNNNNNNNNNNNNNNNNNNNNNNNNNNNNNNNNN

NNNNNNNNNNNNNNNNNNNNNNNNNNNNNNNNNNNNNNNNNNNNNNNNNNNNNNNNNNNN

NNNNNNNNNNNNNNNNNNNNNNNNNNNNGGTAACCAAATCGGTGCTGCTTTCTGGTGCGT

CCCGAGCCTACCACCGCGAGGCGAGCTCGAAACAGACTGACCTCGTAGGCAAACCATCTC

TGGCGAGCACGGTCTCGACAGCAATGGCGTGTATGGACCTCCTATTCCTGACTACCTCGT

CCTCCCTGATGATCGCACAGTTACAACGGCACTTCCGAGCTCCAGCTCGAGCGCATGAAC

GTCTACTTCAACGAGGCAAGTACGTCGCCTTGA-CCGTCTTGCCCTTGCTAACGCGTATT

CAGGCCTCCGGCAACAAGTATGTGCCCCGCGCCGTCCTCGTCGATCTCGAGCCCGGTACC

ATGGACGCCGTC

>Diaporthe_guangxiensis_JZB320094

NNNNNNNNNNNNNNNNNNNNNNNNCCGCTCTTGCTGCTGCGCATGCTAACGGACCGTTTT

CGGCTTGCAGGATAAGGATGGCGATGGTTAGTGCGGCCGCTCTCACACAGCACGCGTCAT

GCTCGATCCTCCGCGACGGCCTGCGCGTAACAATGCGTTATCACTATTGCGAGTTGCTGA

GGTGCAGGACAAATCACCACCAAGGAGCTCGGCACCGTCATGCGATCCCTGGGCCAGAAC

CCTTCCGAGTCTGAGCTGCAAGATATGATTAACGAGGTCGACGCCGACAACAACGGCACC

ATTGACTTTGGTACGTCCAGATATACGCCCA?????????????????????????????

????????????????????????????????????????????????????????????

????????????????????????????????????????????????????????????

????????????????????????????????????????????????????????????

????????????????????????????????????????????????????????????

???????????????????????????NNNNNNNNNNNNNNNNNNNNNNNNNNNNNNNNN

NAATTGCTG-CCTACGCGCACCCAG-AACCCTTTGTGAACTTTTACTGTTGCCTCGGCAT

GCTGGGGGGTCCCCTGAGACAGGGAGCAGGCACGCCGGCGGCCAAGTTAACTCTTGTTTT

TACACTGAAACTCTAAATGAATCAAAACTTTCAACAACGGATCTCTTGGTTCTGGCATCG

ATGAAGAACGCAGCGAAATGCGATAAGTAATGTGAATTGCAGAATTCAGTGAATCATCGA

ATCTTTGAACGCACATTGCGCCCTCTGGTATTCCGGAGGGCATGCCTGTTCGAGCGTCAT

TTCAACCCTCAAGCATTGCTTGGTGTTGGGGCACTGCGAAGCAGGCCCTGAAATCTAGTG

GCGAGCTCGCCAGGACCCCGAGCGCAGTAGACCCTCGCTAAGGCCCTGGCGGCGCCCTGC

TGAAANNNNNNNNNNNNNNNNNNNNNNCATCATGTTGGTACAGCTTCGTCAGCGCATTTT

CACCCCTCGCTCTGGATTTTCAGGGTGCGGGGCTTAGAGCTTATCTAGTTACTACCACCG

GAAACATGCTGATATCTACATAGCCGCCGAGCTTGGTAAGGGNNNNNNNNNNNNNNNNNN

NNNNNNNNNNNNNNNNNNNNNNNNNNNNNNNNNNNNNNNNNNNNNNNNNNNNNNNNNNNN

NNNNNNNNNNNNNNNNNNNNNNNNNNNNNNNNNNNNNNNNNNNNNNNNNNNNNNNNNNNN

NNNNNNNNNNNNNNNNNNNNNNNNNNNNNNNNNNNNNNNNGGTGCTGCTTTCTGGTGCGT

TC-AAGTCCACCACCGCGACATGACCTCGAGCCATACTGACCTCGCAGGCAAACCATCTC

TGGCGAGCACGGCCTCGACAGCAATGGCGTGTATGCACCTCCTATTTCTGCCTTTCTCGT

CTGCCCTGACAATCACACAGTTACAACGGCACTTCCGAGCTCCAGCTCGAGCGCATGAAC

GTCTACTTCAATGAGGTAAGTACGTCA--TTGACCATCTGCAGCCTTGCTAACGCGTTAT

CAGGCCTCCGGCAACAAGTATGTCCCTCGCGCCGTCCTCGTCGATCTCGAGCCCGGTACT

ATGGACGCCGTC

>Diaporthe_guizhouensis_GZAAS_20_0338

NNNNNNNNNNNNNNNNC------CCCGCCGTTGCTGCTGCGCATGCTAACGCACCGTTTT

CGGCCTCCAGGATAAGGATGGTGATGGTTAGTGCGGCTTCCCCAACCCTTTACGCGTCAC

GATCGATTCGCCGCGACAGCATGCGCGCGATCAAGCGCCATCACTACCAGGAGTTGCTAA

GGTGTAGGACAAATCACCACAAAGGAGCTCGGAACGGTCATGCGATCGCTGGGTCAGAAC

CCGTCCGAGTCTGAGCTGCAAGATATGATCAACGAGGTCGATGCCGACAACAATGGCACC

ATCGACTTTGGTACGTCCAGGTGTCTGCTCA?????????????????????????????

????????????????????????????????????????????????????????????

????????????????????????????????????????????????????????????

????????????????????????????????????????????????????????????

????????????????????????????????????????????????????????????

???????????????????????????NNNNNNNNNNNNNNNNNNNNNNNNNNGCGGAGG

GACATTGTTCTCCG-GCGCATCCAGAAACCCTTTGTGAACTTATACTGTTGCCTCGGCAG

GCTGGGGGGCCCCTCTCGGTGAGGAGCAGGCCCGCCGGCGGCCAAGTTAACTCCTGTTTT

TACACTGAAACTCTAAATGAATCAAAACTTTCAACAACGGATCTCTTGGTTCTGGCATCG

ATGAAGAACGCAGCGAAATGCGATAAGTAATGTGAATTGCAGAATTCAGTGAATCATCGA

ATCTTTGAACGCACATTGCGCCCTCTGGTATTCCGGAGGGCATGCCTGTTCGAGCGTCAT

TTCAACCCTCAAGCCTGGCTTGGTGATGGGGCACTGCAGGGCAGGTCCTTAAATTCAGTG

GCGAGCTCGCTAGGACCCCGAGCGCAGTAGACCCTCGCTAAGGTCTTGGTGCGGCCCTGC

TGAAANNNNNNNNNNNNNNNNNNNNNNCCCCACGCTTGCACTGCTGCGTCTTCGCATTTT

CACCCCTCGTTCTGGATTTTCAGGGTGCGGGGCTTAGAGCTTATCTCACAACCATCACCT

GGAATGTGCTAACACTTTACCAGCCGCTGAGCTCGGAAAGGGNNNNNNNNNNNNNNNNNN

NNNNNNNNNNNNNNNNNNNNNNNNNNNNNNNNNNNNNNNNNNNNNNNNNNNNNNNNNNNN

NNNNNNNNNNNNNNNNNNNNNNNNNNNNNNNNNNNNNNNNNNNNNNNNNNNNNNNNNNNN

NNNNNNNNNNNNNNNNNNNNNNNNNNNNNNNNNNNNNNNNNNNNNNNNNNNNNNNNNNNN

NNNNNNTTCATCACCGCGAAACGACCTCGCAACATATTGACTTCGTAGGCAAACCATCTC

TGGCGAGCACGGCCTCGACAGCAATGGCGTGTACGTACCTCCTATTCCTACCCGCCTCGT

CCGCTCTGACAGCTTCACAGTTACAACGGCTCTTCTGAGCTCCAGCTCGAGCGCATGAAC

GTCTACTTCAACGAGGTCAGTTTTTTATACCCACGATCTCCAGCTTTGCTGACACCTTAT

CAGGCCTCCGGCAACAAGTATGTTCCTCGCGCTGTCCTCGTCGATCTCGAGCCCGGTACC

ATGGACGCCGTC

>Diaporthe_gulyae_BRIP_54025

????????????????????????????????????????????????????????????

????????????????????????????????????????????????????????????

????????????????????????????????????????????????????????????

????????????????????????????????????????????????????????????

????????????????????????????????????????????????????????????

????????????????????????????????????????????????????????????

????????????????????????????????????????????????????????????

????????????????????????????????????????????????????????????

????????????????????????????????????????????????????????????

????????????????????????????????????????????????????????????

???????????????????????????NNNNNNNNNNNNNNNNNNNNNNNNNNNNAGGGA

TCATTGCTGCCTC-GGCGCACCCAGAAACCCTTTGTGAACTTATACTGTTGCCTCGGCAG

GCCGGAAGGCCCCCTGAGACAGGGAGCA-GCCCGCCGGCGGCCAACCAAACTC-TGTTTC

TATAGTGAATCTCTAAATGAATCAAAACTTTCAACAACGGATCTCTTGGTTCTGGCATCG

ATGAAGAACGCAGCGAAATGCGATAAGTAATGTGAATTGCAGAATTCAGTGAATCATCGA

ATCTTTGAACGCACATTGCGCCCTCTGGTATTCCGGAGGGCATGCCTGTTCGAGCGTCAT

TTCAACCCTCAAGCCTGGCTTGGTGATGGGGCACTGAAGGGCAGGCCCTGAAATCTAGTG

GCGAGCTCGCCAGGACCCCGAGCGTAGTAGA-TCTCGCTAAGGCCCTGGCGGTGCCCTGC

TGAAANAGAAGGAAGGTCAGTAAACATCATTGTGCCTGCGCGGCTTCGTCAAGCCATTTT

CACCCCTCCCTCTGGGTTTTCAGGGTGCGGGGCTTACCGCTTATCTCACCGTCAACACCG

AACAGACGCTGATTTTCATACAGCCGCCGAGCTGGGCAAGGGTCGCACCTCAGCCCCACC

ATCGCGACCCACCCCCTGCGACACCCAGATAAGACGCGTCGATTGCTAACATGTCGTTCT

CTCCTACAGGTTCACCTTCAGACCGGCCAATGCGTAAGTTGCTGTCACGCCGAGACCTTA

T---CGCCACCCGTAACACGTTTCCCAGGGTAACCAAATCGGTGCTGCTTTCTGGTGCGT

CAAGCCGCCACGGCCGCGAGATTAGCTCGCAACACACTGACCTCGTAGGCAAACCATCTC

TGGCGAGCACGGTCTCGACAGCAATGGCGTGTATGCACCTCCTATTCCTGCCCATCTCGT

CCTCCCTGATGTTTGCACAGTTACAACGGCACTTCTGAGCTCCAGCTCGAGCGCATGAAC

GTCTACTTCAACGAGGTGAGTACGTCTTTTTGAGCGTTCTCGCCTTTACTGACGCGCTTT

CAGGCCTCCGGCAACAAGTATGTGCCTCGCGCCGTCCTCGTCGATCTCGAGCCCGGTACC

ATGGACGCCGTC

>Diaporthe_guttulata_CGMCC_3_20100

NNNNNNNNNNNNNNNNNNNNNNNCCCGCCGTTGCCTTTGCGCATGCTAACGGACCGTTTT

CGGCCTGCAGGATAAGGATGGCGATGGTTAGTGCGGTCACTCTCAGCTACCACGCGTCAT

ACTCGATCCGCCGCGACGGTCTGCGCGTGAGCGAGCGACCTCATGATCACGAGATGCTAA

GGTGTAGGACAAATCACCACCAAGGAGCTCGGCACGGTCATGCGATCCCTGGGTCAGAAC

CCGTCCGAGTCTGAGCTGCAAGATATGATTAACGAGGTCGACGCCGACAACAATGGCACC

ATTGACTTTGGTAAGTCTAGATGTCCACCCATCCGCGCCCTCCACCGGAGGTGTCAAGAA

GCCTCACCGCTACAAGCCTGGTACCGTCGCTCTGCGTGAGATCCGTCGCTACCAGAAGAG

CACCGAGCTGCTGATCCGCAAGCTCCCCTTCCAGCGTCTGGTAAGCAGGTTCGTGAGATC

GCCCAGGACTTCAAGTCCGACCTGCGCTTCCAGTCTTCCGCCATCGGTGCCCTGCAGGAG

TCCGTCGAGTCTTACCTCGTCTCCCTGTTCGAGGACACCAACCTGTGCGCCATCCACGCC

AAGCGTGTCACCATCCAGTCGGTACGTNNNNNNNNNNNNNNNNNNNNNNNNNNNNNNNNN

NNNNNNNNNNNTC-GGCGCACCCAGAAACCCTTTGTGAACTTATACTGTTGCCTCGGCAG

GCCGGAGGGCCCCCTGAGACAGGGAGCA-GCCCGCCGGCGGCCAACCAAACTC-TGTTTC

TATAGTGAATCTCTAAATGAATCAAAACTTTCAACAACGGATCTCTTGGTTCTGGCATCG

ATGAAGAACGCAGCGAAATGCGATAAGTAATGTGAATTGCAGAATTCAGTGAATCATCGA

ATCTTTGAACGCACATTGCGCCCTCTGGTATTCCGGAGGGCATGCCTGTTCGAGCGTCAT

TTCAACCCTCAAGCCTGGCTTGGTGATGGGGCACTGAAGGGCAGGCCCTGAAATCTAGTG

GCGAGCTCGCCAGGACCCCGAGCGTAGTAGA-TCTCGCTAAGGCCCTGGCGGTGCCCTGC

NNNNNNNNNNNNNNNNNNNNNNNNNNNCATTGTGCCTGCGCGGCTTCGTCAAGCCATTTT

CACCCCTCCCTCTGGGTTTTCAGGGTGCGGGGCTTACCGCTTATCTCACCGTCAACACCG

AACAGACGCTGATTTCCATACAGCCGCCGAGCTTGGTNNNNNNNNNNNNNNNNNNNNNNN

NNNNNNNNNNNNNNNNNNNNNNNNNNNNNNNNNNNNNNNNNNNNNNNNNNNNNNNNNNNN

NNNNNNNNNNNNNNNNNNNNNNNNNNNNNNNNNNNNNNNNNNNNNNNNNNNNNNNNNNNN

NNNNNNNNNNNNNNNNNNNNNNNNNNNNNNNNNNNNNNNNNNNNNNNNNNNNNNNNNNNN

NCAGCCGCCACGACCGCGAGATTAGCTCGCAACATACTGACCTCGTAGGCAAACCATCTC

TGGCGAGCACGGTCTCGACAGCAATGGCGTGTATGCACCTCCTATTCCTGCCCATCTCGT

CCTCCCTGATGTTTGCACAGTTACAACGGCACTTCTGAGCTCCAGCTCGAGCGCATGAAC

GTCTACTTCAACGAGGTGAGTACGTCTTTTTGAGCCTTCTCGACCTTACTGACGCGCTTT

CAGGCCTCCGGCAACAAGTATGTGCCTCGCGCCGTCCTCGTCGATCTCGAGCCCGGTACC

ATGGACGCCGTC

>Diaporthe_hartii_BRIP_60285e

????????????????????????????????????????????????????????????

????????????????????????????????????????????????????????????

????????????????????????????????????????????????????????????

????????????????????????????????????????????????????????????

????????????????????????????????????????????????????????????

????????????????????????????????????????????????????????????

????????????????????????????????????????????????????????????

????????????????????????????????????????????????????????????

????????????????????????????????????????????????????????????

????????????????????????????????????????????????????????????

???????????????????????????AACAAGGTCTCCGTTGGTGAACCAGCGGAGGGA

TCATTGCTGCCCCAGGCGCACCCAGAAACCCTTTGTGAACTCTTACTGTTGCCTCGGCAG

GCCGGGGGGCCCCTCGAGACGAGGAGCAGGCCCGCCGGCGGCCAAGCCAACTCTTGTTTT

TACACCGAAACTCTAAATGAATCAAAACTTTCAACAACGGATCTCTTGGTTCTGGCATCG

ATGAAGAACGCAGCGAAATGCGATAAGTAATGTGAATTGCAGAATTCAGTGAATCATCGA

ATCTTTGAACGCACATTGCGCCCTCTGGTATTCCGGAGGGCATGCCTGTTCGAGCGTCAT

TTCAACCCTCAAGCCTGGCTTGGTGTTGGGGCACTGAAGGGCAGGCCCTGAAATCTAGTG

GCGGGCTCGCCAGGACCCCGAGCGCAGTAGACCCTCGCTGAGGCCCTGGCGGTGCCCTGC

?????NNAGAGGAAGGTCAGTAAACATCCCTTCGTTTGCGCCGCGTCGCCACCGCATTTT

CACCCCTCGCTCTTGATTTTCAGAGTGCGGGGCTTAGTGCTTATCT-----CCGCCTCAG

AGAAGATGCTGACATTTCTACAGCCGCTGAGCTCGGCAAGGGTCACACCTGAGCCCCACC

ATCGCGACCGTCAGCCTGCGACACCTGGATGAGACGCGCCCATTGCTAACTTATTTTCCT

TGCCTCCAGGTTCACCTCCAGACCGGCCAATGCGTAAGTTGCTTTCACCACCAGACCTTA

TCATCGCCACCCATAGCACGTTTCGCAGGGTAACCAAATCGGTGCTGCTTTCTGGTGCGT

GCTGAGCCTGCCACCGCGATATGTCCTAGAAGCATGCTGACCTCCTAGGCAAACCATCTC

TGGCGAGCACGGTCTCGACAGCAATGGCGTGTATGCACCTCCTATTCCTGCTCCTCTCGC

CCTCCCTGACAATTGCACAGCTACAACGGCACCTCCGAGCTCCAGCTCGAGCGCATGAAC

GTCTACTTCAACGAGGTAAGTTTGTCCT---GACCCCGCCCTTCCTTGCTGACGCATTAT

CAGGCTTCCGGCAACAAATATGTCCCTCGCGCCGTCCTCGTCGATCTCGAGCCCGGTACC

ATGGATGCTGTC

>Diaporthe_helianthi_CBS_592_81

NNNNNNNNNNNNNNNNNNNNNNNNNNGCCGTTGCTGTTGCGCGTGCTAATGGACCGTTTT

CTGCCTGCAGGATAAGGACGGCGATGGTTAGTGCGGTCACTCTCAGCTGGCACGCGTCAC

AATCGATCCGCCGCAACGGTCTGCGCTCGAGCGACCGTCATTAATATCACGAGTTGCTAA

GGTGTAGGACAAATCACCACCAAGGAGCTGGGCACAGTCATGCGGTCCCTTGGTCAAAAC

CCTTCCGAGTCCGAGCTGCAGGACATGATCAACGAGGTCGACGCCGACAACAATGGCACC

ATTGACTTTGGTACGTTTAGATTCTCGTACATCCGCGCCCTCCACCGGAGGTGTCAAGAA

GCCCCACCGCTACAAGCCTGGTACCGTCGCTCTGCGTGAGATCCGTCGTTATCAGAAGAG

CACCGAGCTGCTGATTCGCAAGCTCCCCTTCCAGCGTCTGGTATGTAGGTTCGTGAGATC

GCCCAGGACTTCAAGTCCGATCTCCGCTTCCAGTCTTCCGCCATCGGTGCCCTGCAGGAG

TCTGTCGAGTCTTACCTCGTCTCCCTCTTTGAGGACACCAACCTGTGCGCCATCCACGCC

AAGCGTGTCACCATCCAGTCGGTATGTAACAAGGTCTCCGTTGGTGAACCAGCGGAGGGA

TCATTGCTGCCCCCGGCGCACCCAGAAACCCTTTGTGAACTTAT-CTGTTGCCTCGGCAG

GCCGGGGGGCCCCCTGGAACAGGGAGCA-GCCCGCCGGCGGCCGACCAAACTCTTGTTTC

TACAGTGGATCTCTAAATGAATCAAAACTTTCAACAACGGATCTCTTGGTTCTGGCATCG

ATGAAGAACGCAGCGAAATGCGATAAGTAATGTGAATTGCAGAATTCAGTGAATCATCGA

ATCTTTGAACGCACATTGCGCCCTCTGGTATTCCGGAGGGCATGCCTGTTCGAGCGTCAT

TTCAACCCTCAAGCCTGGCTTGGTGATGGGGCACTGCAGGGCAGGCCCTGAAATCCAGCG

GCGAGCCCGCCGGGACCCCGAGCGTAGTAGT-TCTCGCTAAGGCCCTGGCGGCGCCCTGC

TGAAAGAGAAGGAAGGTTAGTAAACATCGTTGTGCATGCGAGGCTTCGTCAAGGCATTTT

CACCCCTCCCTCTGGATTTTCAGGGTGCGGGGCTTACCGCTTATCTCACCAGTACAACCC

AGAACGGACTGATTTCTATATAGCCGCCGAGTTGGGTAAGGGTCGCACCTCAACCCCACC

ATCGCGACCCACCCCCTACGACACCCAGATAGGACGCGTCGATTGCTAACATGTTTTCCT

CGCCTACAGGTTCACCTTCAGACCGGCCAATGCGTAAGTTGCTGTCACGGCCGGGCCGTT

T---CGCCACCTGTAGCACGTTTCCCAGGGTAACCAAATCGGTGCTGCTTTCTGGTGCGT

CCCAAGCCTACCACCGCGAGGCTGGCTCGTAGTCTGCTGACCCCGCAGGCAAACCATCTC

TGGCGAGCACGGTCTCGACAGCAATGGCGTGTATGTACCTCCTATTCCTGACTTACTCGT

CCTCACTAATGATCGCACAGCTACAACGGCACTTCCGAGCTCCAGCTCGAGCGCATGAAC

GTCTACTTCAACGAGGTACGCACCTCGTTTTGA-CCCTCTCGCCCTTGCTAACGTGTTTT

CAGGCTTCCGGCAACAAGTATGTGCCTCGCGCCGTCCTCGTCGATCTCGAGCCCGGTACC

ATGGACGCCGTC

>Diaporthe_helicis_CBS_138596

CTTTGTAATCTCCAGCCGACATGCCCGCCCTTGCTGGTGCGCATGCTAACGGACCGTTTT

CGGCTTGTAGGATAAGGATGGCGATGGTTAGTGCAGCCGCTCCCACCTAGCACGCGTCAT

GTTCGATCCGCCGCGACAGCCTGCGCGCAACCAAGCGTTATCACTATCACGAGTTGCTGA

GGTGTAGGACAAATCACCACCAAGGAGCTCGGCACGGTCATGCGATCCCTGGGTCAGAAC

CCGTCCGAGTCTGAGCTGCAAGATATGATTAACGAGGTCGACGCCGACAACAATGGCACC

ATTGACTTTGGTACGTCCAGATGCTCGCGCTTCCGCGCCCTCCACCGGAGGTGTCAAGAA

GCCTCACCGCTACAAGCCTGGTACCGTCGCTCTGCGTGAGATCCGTCGCTACCAGAAGAG

CACCGAGCTGCTGATCCGCAAGCTCCCTTTCCAGCGTCTGGTATGTAGGTCCGTGAGATC

GCCCAGGACTTCAAGTCCGACCTCCGCTTCCAGTCTTCCGCCATCGGTGCCCTGCAGGAG

TCGGTCGAGTCTTACCTCGTCTCTCTCTTCGAGGACACCAACCTGTGCGCCATCCACGCC

AAGCGTGTCACCATCCAGTCGGTACGTNNNNNNNNNNNNNNNNNNNNNNNNGCGGAGGGA

TCATTGCTGCCCCAGGCGCACCCAGAAACCCTTTGTGAACTTTTACTGTTGCCTCGGCAT

GCCGGGGGGCCCCTCTTCTGGAGGAGCAGGCACGCCGGCGGCCAACCTAACTCTTGTTTT

TACACTGAAACTCTAAATGAATCAAAACTTTCAACAACGGATCTCTTGGTTCTGGCATCG

ATGAAGAACGCAGCGAAATGCGATAAGTAATGTGAATTGCAGAATTCAGTGAATCATCGA

ATCTTTGAACGCACATTGCGCCCTCTGGTATTCCGGAGGGCATGCCTGTTCGAGCGTCAT

TTCAACCCTCAAGCCTGGCTTGGTGATGGGGCACTGAGAAGCAGGCCCTGAAATCCAGTG

GCGAGCTCGCCAGGACCCCGAGCGCAGTAGACCCTCGCTAAGGCCCTGGCGGTGCCCTGC

TGAAAGAGAAGGAAGGTTAGTAAATATCATCATGCTCGCGCGGCCTCGCCAGCGCATTTT

CACCCCTCGCTTTGGATTTTCAGAGTGCGGGGCTTAGGGCTTATCTTGTCACCACCACCG

AATATATGCTGATATCTACACAGCCGCCGAGCTTGGTAAGGGNNNNNNNNNNNNNNNNNN

NNNNNNNNNNNNNNNNNNNNNNNNNNNNNNNNNNNNNNNNNNNNNNNNNNNNNNNNNNNN

NNNNNNNNNNNNNNNNNNNNNNNNNNNNNNNNNNNNNNNNNNNNNNNNNNNNNNNNNNNN

NNNNNNNNNNNNNNNNNNNNNNNNNNNNNNNNNNNNNNNNGGTGCTGCTTTCTGGTGCGT

TCCAAGTCCACCGCCGTGATACGACCTCGAAGCATGCTGACCTCGTAGGCAAACCATCTC

TGGCGAGCACGGCCTCGACAGCAATGGCGTGTATGCACCTCCTATTCCTGCCCATCTTGG

CTTCCCTGACAATCGCATAGTTACAACGGCACTTCTGAGCTCCAGCTCGAGCGCATGAAC

GTCTACTTCAACGAGGTAAGTACATCATTCCGACCATCTCCAACCTCGCTAACGTGTTAT

CAGGCCTCCGGCAACAAGTATGTTCCTCGCGCCGTCCTCGTCGATCTCGAGCCCGGTACC

ATGGACGCCGTC

>Diaporthe_heliconiae_SAUCC194_77

TTTTGTAATTTCCAGCCGACATGCCCGCCCTTGCTGGTGCGCATGCTAACGGACCGTTTT

CGGCTTGTAGGATAAGGATGGCGATGGTTAGTGCGGCCGCTCCCACCTAGCACGCGTCAT

GTTCGATCCGCCGCGACAGCCTGCGCGCAACCAAGCGTTATCACTCTCACGAGTTGCTGA

GGTGTAGGACAAATCACCACCAAGGAGCTCGGCACGGTCATGCGATCCCTGGGTCAGAAC

CCGTCCGAGTCTGAGCTGCAAGATATGATTAACGAGGTCGACGCCGACAACAATGGCACC

ATTGACTTTGGTACGTCCAGATGCTCGCGCTTCCGCGCCCTCCACCGGAGGTGTCAAGAA

GCCTCACCGCTACAAGCCTGGTACCGTCGCTCTGCGTGAGATCCGTCGCTACCAGAAGAG

CACCGAGCTGCTGATCCGCAAGCTCCCCTTCCAGCGTCTGGTATGCAGGTCCGTGAGATC

GCCCAGGACTTCAAGTCCGACCTCCGCTTCCAGTCCTCCGCCATCGGTGCCCTGCAGGAG

TCGGTCGAGTCTTACCTCGTCTCCCTCTTCGAGGACACCAACCTGTGCGCCATCCACGCC

AAGCGTGTCACCATCCAGTCGGTACGTNNNNNNNTCTCCGTTGGTGAACCAGCGGAGGGA

TCATTGCTGCCCCAGGCGCACCCAGAAACCCTTTGTGAACTTTTACTGTTGCCTCGGCTA

GCTGGGGGGCCCCTCTTCTGGAGGAGCAGGCACGCCGGCGGCCAACCTAACTCTTGTTTT

TACACTGAAACTCTAAATGAATCAAAACTTTCAACAACGGATCTCTTGGTTCTGGCATCG

ATGAAGAACGCAGCGAAATGCGATAAGTAATGTGAATTGCAGAATTCAGTGAATCATCGA

ATCTTTGAACGCACATTGCGCCCTCTGGTATTCCGGAGGGCATGCCTGTTCGAGCGTCAT

TTCAACCCTCAAGCCTGGCTTGGTGATGGGGCACTGAAGGGCAGGCCCTGAAATTCAGTG

GCGAGCTCGCCAGGACCCCGAGCGCAGTAGACCCTCGCTAAGGCCCTGGCGGTGCCCTGC

TGAAANNNNNNNNNNGTTAGTAAATACCATGATGCCCGCGCGGCTTCGTCAGCGCATTTT

CACCCCTCGCTTTGGATTTTCAGGGTGCGGGGCTTAGGGCTTATCTCACTACCACCACCG

AGTATATGCTGATATCTACACAGCCGCCGAGCTTNNNNNNNNNNNNNNNNNNNNNNNNNN

NNNNNNNNNNNNNNNNNNNNNNNNNNNNNNNNNNNNNNNNNNNNNNNNNNNNNNNNNNNN

NNNNNNNNNNNNNNNNNNNNNNNNNNNNNNNNNNNNNNNNNNNNNNNNNNNNNNNNNNNN

NNNNNNNNNNNNNNNNNNNNNNNNNNNNNNNNNNNNNNNNNNNNNNNNNNNNNNNNNNNN

NCTCAGTCCACCGCCGCGATACGACCTCGAAGCATACTGACCTCGTAGGCAAACCATCTC

TGGCGAGCACGGCCTCGACAGCAATGGCGTGTATGCACCTCCTATTCCTGCCCATCTTGG

ATTCCCTGACAATTGCACAGTTACAACGGCACTTCTGAGCTGCAGCTCGAGCGCATGAAC

GTCTACTTCAACGAGGTAAGTACAGCATTCCGACCTTCCTCAACCTTGCTAACGCGTTAT

CAGGCCTCCGGCAACAAGTATGTTCCTCGCGCCGTCCTCGTCGATCTCGAGCCCGGTACC

ATGGATGCCGTC

>Diaporthe_heterophyllae_CBS_143769

NNNNNNNNNNNNNNNNNNNNNNNNNNNNNCTTGCTGGTGCGCATGCTAACGGACCGTTTT

CGGCTTGTAGGATAAGGATGGCGATGGTTAGTGCGGCCGCTCTCACATAGCACGCGTCAT

GTTCGATCCGACGCGACAGTCTGCGCGCAGCCAAGCGTTATCACTATCACGAGTTACTGA

GGTGTAGGACAAATCACCACCAAGGAGCTCGGCACGGTCATGCGATCCCTGGGCCAGAAC

CCGTCCGAGTCTGAGCTGCAAGATATGATTAACGAGGTCGACGCCGACAACAATGGCACC

ATTGACTTTGGTACGTCCAGATGCTCGCGCTTCCGCGCCCTCTACCGGAGGTGTCAAGAA

GCCTCACCGCTACAAGCCTGGTACCGTCGCTCTGCGTGAGATCCGTCGCTACCAGAAGAG

CACCGAGCTGCTGATCCGCAAGCTCCCCTTCCAGCGTCTGGTATGCAGGTCCGTGAGATC

GCCCAGGACTTCAAGTCCGACCTCCGCTTCCAGTCTTCCGCCATCGGTGCCCTGCAGGAG

TCGGTCGAGTCTTACCTCGTCTCCCTCTTCGAGGACACCAACCTGTGCGCCATCCACGCC

AAGCGTGTCACCATCCAGTCGGTATGTAACAAGGTCTCCGTTGGTGAACCAGCGGAGGGA

TCATTGCTGCCCCAGGCGCACCCAGAAACCCTTTGTGAACTTTTACTGTTGCCTCGGCAT

GCCGGGGGGCCCCTC-TCCGGAGGAGCAGGCACGCCGGCGGCCAAGTTAACTCTTGTTTT

TACACTGAAACTCTAAATGAATCAAAACTTTCAACAACGGATCTCTTGGTTCTGGCATCG

ATGAAGAACGCAGCGAAATGCGATAAGTAATGTGAATTGCAGAATTCAGTGAATCATCGA

ATCTTTGAACGCACATTGCGCCCTCTGGTATTCCGGAGGGCATGCCTGTTCGAGCGTCAT

TTCAACCCTCAAGCCTGGCTTGGTGATGGGGCACTGAAGGGCAGGCCCTGAAATTCAGTG

GCGAGCTCGCCAGGACCCCGAGCGCAGTAGACCCTCGCTAAGGCCCTGGCGGTGCCCTGC

TGAAANNNNNNNNNNNNNNNNNNNNNCCATCATGCTCGCGCGGCTTCGTCAGCGTATTTT

CACCCCTCGCTCTGGATTTTCAGGGTGCGGGGCTTAGGGCTTATCTAACCACCCCCACCG

AATCCATGCTGATATTTCCACAGCCGCCGAGCTTGGCAAGGGNNNNNNNNNNNNNNNNNN

NNNNNNNNNNNNNNNNNNNNNNNNNNNNNNNNNNNNNNNNNNNNNNNNNNNNNNNNNNNN

NNNNNNNNNNNNNNNNNNNNNNNNNNNNNNNNNNNNNNNNNNNNNNNNNNNNNNNNNNNN

NNNNNNNNNNNNNNNNNNNNNNNNNNNNNNNNNNNNNNNNNNNNNNNNNNNNNNNNNNNN

NCCAAGTCCACCGCCGCGATACGACCTCGAAGCATACTGACCTCGTAGGCAAACCATCTC

TGGCGAGCACGGCCTCGACAGCAATGGCGTGTATGCACCTCCTATTCCTGCCCATCTTGG

CTTCCCTGACAATTGCACAGTTACAACGGCAGTTCTGAGCTCCAGCTCGAGCGCATGAAC

GTCTATTTCAACGAGGTAAGTACATCATTCCGACCATCTCCAACCTTGCTAACGCGTTAT

CAGGCCTCCGGCAACAAGTATGTTCCCCGCGCCGTCCTCGTCGATCTCGAGCCCGGTACC

ATGGACGCCGTC

>Diaporthe_heterostemmatis_SAUCC194_85

NNNNNNNNGGCAATCTCGCCTTGCCCGCCGTTGCCGTTGCGCATGCTAACGGACCGTTTT

CGGCCTGCAGGATAAGGATGGCGATGGTTAGTGCGGTCACTCCCGGCTAGCACGCGTCAT

GCTCGATCCGCCGCGACGGTCTGCGCGCGAGCGACCGTCATCAATATCACGAGTTGCTAA

GATGTAGGACAAATCACCACCAAGGAGCTCGGCACAGTCATGCGGTCGCTTGGTCAAAAC

CCTTCCGAGTCCGAGCTGCAGGACATGATCAACGAGGTCGACGCCGACAACAACGGCACC

ATTGACTTTGGTAAGTCTCAACTGT--CACATCCGCGCCCTCCACCGGAGGTGTCAAGAA

GCCTCACCGCTACAAGCCTGGTACCGTCGCTCTGCGTGAGATCCGTCGCTACCAGAAGAG

CACCGAGCTGCTGATCCGCAAGCTCCCCTTCCAGCGTCTGGTATGCAGGTCCGTGAGATC

GCCCAGGACTTCAAGTCCGACCTGCGCTTCCAGTCTTCCGCCATCGGTGCTCTCCAGGAG

TCCGTCGAGTCTTACCTCGTCTCCCTCTTCGAGGACACCAACCTGTGCGCCATCCACGCC

AAGCGTGTCACCATCCAGTCGGTACGTNNNNNNNTCTCCGTTGGTGAACCAGCGGAGGGA

TCATTGCTGCTTC-GGCGCACCCAGAAACCCTTTGTGAACTTA--TTGTTGCCTCGGCAG

GCCGGGAGGCCCCCTGAAACAGGGAGCA-GCCCGCCGGCGGCCAACCAAACTCTTGTTTC

TATAGTGAATCTCTAAATGAATCAAAACTTTCAACAACGGATCTCTTGGTTCTGGCATCG

ATGAAGAACGCAGCGAAATGCGATAAGTAATGTGAATTGCAGAATTCAGTGAATCATCGA

ATCTTTGAACGCACATTGCGCCCTCTGGTATTCCGGAGGGCATGCCTGTTCGAGCGTCAT

TTCAACCCTCAAGCCTGGCTTGGTGATGGGGCACTGTAGGGCAGGCCCTGAAATCTAGTG

GCGAGCTCGCCAGGACCCCGAGCGTAGTAGA-TCTCGCTAAGGCCCTGGCGGTGCCCTGC

TGAAANNNNNNNNNNNNNNNNNNGCGTCGTTGTGTCTACGAGGCTTCGTCAAGGCATTTT

CACCCCTCCCTCTGGATTTTCAGGGTGCGGGGCTTACCGCTTATCTCAGTGAC-GCACCG

AAAAGATGCTGATTTCTGAACAGCCGCCGAGCTGNNNNNNNNNNNNNNNNNNNNNNNNNN

NNNNNNNNNNNNNNNNNNNNNNNNNNNNNNNNNNNNNNNNNNNNNNNNNNNNNNNNNNNN

NNNNNNNNNNNNNNNNNNNNNNNNNNNNNNNNNNNNNNNNNNNNNNNNNNNNNNNNNNNN

NNNNNNNNNNNNNNNNNNNNNNNNNNNNNNNNNNNNNNNNNNNNNNNNNNNNNNNNNNCT

C---AGCCTACCACCGCGAGGCGAGCCCGAAGCATACTGACCTCGTAGGCAAACCATCTC

TGGCGAGCACGGTCTCGACAGCAATGGCGTGTATGCACCTCCTATTCCTGCCCACCTGGT

CCTCCCTGATGATCGCACAGTTACAACGGCACTTCCGAGCTCCAGCTCGAGCGCATGAAC

GTCTACTTCAACGAGGTATGTACGTCGTTTTGA-CCCTCTCGCCCTTGCTAACGCGTTTT

CAGGCTTCCGGCAACAAGTATGTGCCCCGCGCCGTCCTCGTCGATCTCGAGCCCGGTACC

ATGGACGCCGTC

>Diaporthe_hickoriae_CBS_145_26

CTTTGTAATCTCCAGCCGACATGCCCGCCCTGCTGTTTGCGCATGCTAACGGACCGTTTT

CGGCTTGCAGGATAAGGATGGCGATGGTTAGTGCGGTCGCTCCCACACCACACGCGTAAC

GCCTGGTCCGCCGCGACGGCCTGCGCGCAAACAAGCGGTACCGCTATGACGAGTTGCTGA

GGTGTAGGACAAATCACCACCAAGGAGCTCGGCACGGTCATGCGGTCCCTGGGTCAGAAC

CCGTCCGAGTCTGAGCTGCAAGATATGATCAACGAGGTCGACGCCGACAACAATGGCACC

ATTGACTTTGGTACGTCCAGGTGCTCGCCCGTCCGCGCCCTCCACCGGAGGTGTCAAGAA

GCCTCACCGCTACAAGCCTGGTACCGTCGCTCTGCGTGAGATCCGTCGTTACCAGAAGAG

CACCGAGCTGCTGATCCGCAAGCTGCCCTTCCAGCGCCTGGTATGCAGGTTCGTGAGATC

GCCCAGGACTTCAAGTCCGACCTCCGCTTCCAGTCCTCCGCCATCGGTGCCCTGCAGGAG

TCCGTCGAGTCTTACCTCGTCTCCCTGTTTGAGGACACCAACCTGTGCGCCATCCACGCC

AAGCGTGTCACCATCCAGTCGGTACGTAACAAGGTCTCCGTTGGTGAACCAGCGGAGGGA

TCATTGCTGCCCCAGGCGCACCCAGAAACCCTTTGTGAACTTTTACTGTTGCCTCGGCAG

GCCGGGGGGCCCCTCCCCAGGAGGAGCAGGCCCGCCGGCGGCCAAGCCAACTC-TGTTTT

TACACTGGAACTCTAAATGAATCAAAACTTTCAACAACGGATCTCTTGGTTCTGGCATCG

ATGAAGAACGCAGCGAAATGCGATAAGTAATGTGAATTGCAGAATTCAGTGAATCATCGA

ATCTTTGAACGCACATTGCGCCCTCTGGTATTCCGGAGGGCATGCCTGTCCGAGCGTCAT

TTCAACCCTCAAGCCTGGCTTGGTGTTGGGGCACTGGGGAGCAGGCCCTGAAATACAGTG

GCGGGCTCGCCAGGACTCCGAGCGCAGTAGACCCTCGCTAAGG-CCTGGCGGTGCCCTGC

TGAAAGAGAAGGAAGGTCAGTAAACATCATCGTGCTCTCGCGGCCTCGTCGGCGCATTTT

GACCCCTCCCTCTGGACTTTCAGGGTGCGGGGCTTAGAACTTATCTCACC---------G

AAACCATGCTGACTATCATACAGCCGCCGAGCTTGGTAAGGGTCGCACCTGAGCCCCACC

ATCGCGACCCACCCCCTGCGACACCCAGATAAGACGCGTCGATTGCTAACATGTTTTCCT

CGCCCACAGGTTCACCTCCAGACCGGCCAATGCGTAAGTTGCTGTCACACCGGA-CCTTA

TCATCGCCA-CCGTAGCACGTTTCCCAGGGTAACCAAATCGGTGCTGCTTTCTGGTGCGT

CC-AAGTCCACCACCGCGATACGACCTTGAAGCATACTGACCTCACAGGCAAACCATCTC

TGGCGAGCACGGTCTCGACAGCAATGGCGTGTATGCACCTCCTATTTCTGTTCTTCTCGT

-GGCCCTGACAATCACACAGTTACAACGGCACTTCCGAGCTCCAGCTCGAGCGCATGAAC

GTCTACTTCAACGAGGTAAGTACGTTG--TTGACCATCTAAAGCGTTGCTAACGCGTTAT

CAGGCCTCCGGCAACAAGTATGTGCCCCGCGCCGTCCTCGTCGATCTCGAGCCCGGTACC

ATGGACGCCGTC

>Diaporthe_hispaniae_CBS_143351

NNNNNNNNNNNNNNNTCCACATGCCCGCCCTTGCTGTTGCGCATGCTAACGGACCGTTTT

CGGCCTGCAGGATAAGGATGGCGATGGTTAGTGTGCCCGCACCCACGGAGCACGCGTCAT

GCTCGATCCGCCGCGACGGCCTGCGCGCGAACAGGCGTCAGCACTATCAGGGATTGCTAA

GGCGTAGGACAAATCACCACGAAGGAGCTCGGCACTGTCATGCGGTCTCTGGGCCAGAAC

CCGTCCGAGTCTGAACTGCAAGATATGATTAACGAGGTCGATGCCGACAACAATGGCACC

ATTGACTTTGGTACGTCCACACGCTCGCCCATCCGCGCCCTCCACCGGAGGTGTCAAGAA

GCCTCACCGCTACAAGCCTGGTACTGTCGCTCTGCGTGAGATCCGTCGTTACCAGAAGAG

CACCGAGCTGCTGATCCGCAAGCTCCCCTTCCAGCGTCTGGTATGCAGGTCCGTGAGATC

GCCCAGGACTTCAAGTCCGACCTCCGCTTCCAGTCTTCCGCCATCGGTGCCCTGCAGGAG

TCCGTCGAGTCTTACCTCGTCTCCCTCTTTGAGGATACCAACCTGTGCGCCATCCACGCC

AAGCGTGTCACCATCCAGTCGGTATGTAACAAGGTCTCCGTTGGTGAACCAGCGGAGGGA

TCATTGCTGCCCCTGGCGCACCCAGAAACCCTTTGTGAACTCTTACCGTTGCCTCGGCAG

GCCGGGGGGCCCCTCGAGACGAGGAGCAGGCCCGCCGGCGGCCAAGTTAACTCTTGTTTT

TACACTGAAACTCTAAATGAATCAAAACTTTCAACAACGGATCTCTTGGTTCTGGCATCG

ATGAAGAACGCAGCGAAATGCGATAAGTAATGTGAATTGCAGAATTCAGTGAATCATCGA

ATCTTTGAACGCACATTGCGCCCTCTGGTATTCCGGAGGGCATGCCTGTTCGAGCGTCAT

TTCAACCCTCAAGCCTGGCTTGGTGATGGGGCACTGGGGAGCAGGCCCTGAAATCCAGTG

GCGAGCTCGCCAGGACCCCGAGCGCAGTAGACCCTCGCTGAGGCCCTGGCGGTGCCCTGC

TGAAAGAGAAGGAAGGTTAGTAAACATCATCGCGCCTGTGCAGCTCCGTCAGCGCATTTT

CACCCCTCGTTCTGGATTTTCAGGGTGCGGGGCTTAGAGCTTATCTCCCCACCACCGCCG

GAATGAAGCTAACATCTATACAGCCGCCGAGCTTGGCAAGGGNNNNNNNNNNNNNNNNNN

NNNNNNNNNNNNNNNNNNNNNNNNNNNNNNNNNNNNNNNNNNNNNNNNNNNNNNNNNNNN

NNNNNNNNNNNNNNNNNNNNNNNNNNNNNNNNNNNNNNNNNNNNNNNNNNNNNNNNNNNN

NNNNNNNNNNNNNNNNCACGTTTCCCAGGGTAACCAAATCGGTGCTGCTTTCTGGTGCGT

TC-AAGTCCACCGCCGCGATACGACCTCGAAGCATACTGACCTCGCAGGCAAACCATCTC

TGGCGAGCACGGTCTCGACAGCAATGGCGTGTATGCACCTCCTATTCCTGTCTTTCTCGT

-TGCCCTGACAATCACACAGTTACAACGGCACTTCCGAGCTCCAGCTCGAGCGCATGAAC

GTCTACTTCAACGAGGTAAGTACGTTG--TTGACCATCTACAGCTTCGCTAACGCGTTAT

CAGGCCTCCGGCAACAAGTATGTGCCCCGCGCCGTCCTCGTCGATCTCGAGCCCGGTACC

ATGGACGCCGTC

>Diaporthe_hongkongensis_CBS_115448

CTTTGTAATCTCCAGCCGACACGCCCGCCCTTGCTGTTGCGCATGCTAACGGACCGTTTT

CGGCTTGTAGGATAAGGATGGCGATGGTTAGTGCGGCCGCTCTCACATAGCACGCGTCAT

GCTCGATCCGCCGCGACGGCCTGCGCGCAACCAAGCGTTATCACTATTGCGAGTTGCTGA

GGTGTAGGACAAATCACCACCAAGGAGCTCGGCACGGTCATGCGATCCCTGGGACAGAAC

CCGTCCGAGTCTGAGCTGCAAGATATGATTAACGAGGTCGACGCCGACAACAACGGCACC

ATTGACTTTGGTACGTCCAAAAGCACGCCCGTCCGCGCCCTCCACCGGAGGTGTCAAGAA

GCCTCACCGCTACAAGCCTGGTACCGTCGCTCTGCGTGAGATCCGTCGCTACCAGAAGAG

CACTGAGCTGCTGATCCGCAAGCTCCCCTTCCAGCGTCTGGTATGCAGGTCCGTGAGATC

GCCCAGGACTTCAAGTCCGACCTCCGCTTCCAGTCTTCCGCCATCGGTGCCCTGCAGGAG

TCCGTCGAGTCTTACCTCGTCTCCCTCTTCGAGGACACCAACCTGTGCGCCATCCACGCC

AAGCGTGTCACCATCCAGTCGGTATGTAACAAGGTCTCCGTTGGTGAACCAGCGGAGGGA

TCATTGCTGCCCCAGGCGCACCCAGAAACCCTTTGTGAACTTTTACTGTTGCCTCGGCAT

GCTGGGGGGCCCCTCGAGACGAGGAGCAGGCACGCCGGCGGCCAAGTTAACTCTTGTTTT

TACACTGAAACTCTAAATGAATCAAAACTTTCAACAACGGATCTCTTGGTTCTGGCATCG

ATGAAGAACGCAGCGAAATGCGATAAGTAATGTGAATTGCAGAATTCAGTGAATCATCGA

ATCTTTGAACGCACATTGCGCCCTCTGGTATTCCGGAGGGCATGCCTGTTCGAGCGTCAT

TTCAACCCTCAAGCATTGCTTGGTGTTGGGGCACTGAAAAGCAGGCCCTGAAATCTAGTG

GCGAGCTCGCCAGGACCCCGAGCGCAGTAGACCCTCGCTAAGGCCCTGGCGGTGCCCTGC

TGAAAGAGAAGGAAGGTTAGTAAACATCATCATGTTGGTACGGCTTCGTCAGCGCATTTT

CACCCCTCGCTCTGGA-TTTCAGGGTGCGGGGCTTAGAGCTTATCT-----ATGCCGTTC

GAAACATGCTGATATCTACACAGCCGCCGAGCTTGGTAAGGGTCGCACCTGAGCCCCACC

ATCGCGACCCACCCCCTGGGACACCCAGATAAAACGCGTCGATTGCTAACGTGTTTTTCT

CGACTACAGGTTCACCTTCAGACCGGCCAATGCGTAAGTTGCTGTCACGCCAGA-CCTTA

TCATCGCCACCCGTAGCACGTTTCCCAGGGTAACCAAATCGGTGCTGCTTTCTGGTGCGT

CC-AAGTCCACCACCGCGATACGACCTCGAGCCATACTGACATCGCAGGCAAACCATCTC

TGGCGAGCACGGCCTCGACAGCAATGGCGTGTATGCACCTCCTATTCCTACTCTTCTTGT

CTGCCCTGACAATCACACAGTTACAACGGCTCTTCCGAGCTCCAGCTCGAGCGCATGAAC

GTCTACTTCAACGAGGTAAGTACGTCG--TTGACCATCTGCAGCCTTGCTAACGCGTTAT

CAGGCCTCCGGCAACAAGTATGTCCCTCGCGCCGTCCTCGTCGATCTCGAGCCCGGTACC

ATGGACGCCGTC

>Diaporthe_hordei_CBS_481_92

CTTTGTAAGTTATCTTCGCCTTACCCGCCGTTGCCGTTGCGCGTGCTAACGGACCGTTTT

CGGCCTGCAGGATAAGGATGGTGATGGTTAGTGCGGTCACTCTCAGCTTGAACGCGTCAT

ACTCGATCCGCCGCGACGGTCTGCGCTCGAGCGACCATCTTCAATATCACGAGTTGCTAA

GGTGTAGGACAAATCACCACCAAGGAGCTCGGCACAGTCATGCGGTCCCTTGGTCAAAAC

CCTTCCGAGTCCGAGCTGCAGGACATGATCAACGAGGTCGACGCCGACAACAATGGCACC

ATTGACTTTGGTAAGTCTAGATTCTCTCACATCCGCGCCATCCACCGGAGGTGTCAAGAA

GCCTCACCGCTACAAGCCTGGTACCGTCGCTCTGCGTGAGATCCGTCGCTACCAGAAGAG

CACTGAGCTGCTGATCCGCAAGCTCCCCTTCCAGCGCCTGGTATGCAGGTCCGTGAGATC

GCCCAGGACTTCAAGTCCGACCTGCGCTTCCAGTCTTCCGCCATCGGTGCCCTTCAGGAG

TCCGTCGAGTCTTACCTCGTCTCCCTCTTTGAGGACACCAACCTGTGCGCCATCCACGCC

AAGCGTGTCACCATCCAGTCGGTACGTAACAAGGTCTCCGTTGGTGAACCAGCGGAGGGA

TCATTGCTGCCTC-GGCGCACCCAGAAACCCTTTGTGAACTTAT-CTGTTGCCTCGGCAG

GCCGGGGGGCCCCCTGAGACAGGGAGCA-GCCCGCCGGCGGCCAACCAAACTCTTGTTTC

TACAGTGAATCTCTAAATGAATCAAAACTTTCAACAACGGATCTCTTGGTTCTGGCATCG

ATGAAGAACGCAGCGAAATGCGATAAGTAATGTGAATTGCAGAATTCAGTGAATCATCGA

ATCTTTGAACGCACATTGCGCCCTCTGGTATTCCGGAGGGCATGCCTGTTCGAGCGTCAT

TTCAACCCTCAAGCCTGGCTTGGTGATGGGGCACTGAAGGGCAGGCCCTGAAATCTAGTG

GCGAGCTCGCCAGGACCCCGAGCGTAGTAGA-TCTCGCTAAGGCCCTGGCGGTGCCCTGC

TGAAAGAGAAGGAAGGTTAGTAAACATC---GTCTCTGCGAGGCTTCGTCAAGGCATTTT

CACCCCTCCCTCTTGATTTTCAGGGTGCGGGGCTTACCGCTTATCTCAGCGCC-ACCC-G

AAAACATGCTGA---CTATACAGCCGCTGAGCTGGGTAAGGGTCGCAACTCAGCCCCACC

ATCGCGACCCACCCCTTGCGACACCCA---GAGACGCGTCGATTGCTAACATATTTTCCT

TGCCCACAGGTTCACCTCCAGACCGGCCAATGCGTAAGTTGCTGTCACCGTCGGACCTTA

C---CGCCACCCGTAGCACATTTCCCAGGGTAACCAAATCGGTGCTGCTTTCTGGTGCGT

CCCGAGCCTACCACCGCGAGGCTAGCTCGTAGCCTACTGACCTCGTAGGCAAACCATCTC

TGGCGAGCACGGTCTCGACAGCAATGGCGTGTATGCACCTCCTATTCCTGACTTCCTCGT

CCTGCCTGATGATCGCACAGTTACAACGGCACTTCCGAGCTCCAGCTCGAGCGCATGAAT

GTCTACTTCAACGAGGTAAGTACGTCGCTTTGA-CCGTCTCGGACTTGCTAACGCGTTTT

CAGGCCTCCGGCAACAAGTATGTGCCCCGGGCCGTCCTCGTCGACCTCGAGCCCGGTACC

ATGGACGCCGTC

>Diaporthe_howardiae_BRIP_59697a

????????????????????????????????????????????????????????????

????????????????????????????????????????????????????????????

????????????????????????????????????????????????????????????

????????????????????????????????????????????????????????????

????????????????????????????????????????????????????????????

????????????????????????????????????????????????????????????

????????????????????????????????????????????????????????????

????????????????????????????????????????????????????????????

????????????????????????????????????????????????????????????

????????????????????????????????????????????????????????????

???????????????????????????AACAAGGTCTCCGTTGGTGAACCAGCGGAGGGA

TCATTGCTGCCCCAGGCGCACCCAGAAACCCTTTGTGAACTTTTACTGTTGCCTCGGCAT

GCTGGGGGGCCCCCTGAGACAGGGAGCAGGCACGCCGGCGGCCAAGTTAACTCTTGTTTT

TACACTGAAACTCTAAATGAATCAAAACTTTCAACAACGGATCTCTTGGTTCTGGCATCG

ATGAAGAACGCAGCGAAATGCGATAAGTAATGTGAATTGCAGAATTCAGTGAATCATCGA

ATCTTTGAACGCACATTGCGCCCTCTGGTATTCCGGAGGGCATGCCTGTTCGAGCGTCAT

TTCAACCCTCAAGCATTGCTTGGTGTTGGGGCACTGCCAAGCAGGCCCTGAAATCTAGTG

GCGAGCTCGCCAGGACCCCGAGCGCAGTAGACCCTCGCTAAGGCCCTGGCGGTGCCCTGC

TGAAAGAGAAGGAAGGTTAGTACATATCGTCATGTTGGTATGGCTTCGTCAGCGCATTTT

CACCCCTCGCTCTGGATTTTCAGGGTGCGGGGCTTAGAGCTTATCTCGCTACTACCACCG

GAAACATGCTGATATCTTCATAGCCGCCGAGCTTGGCAAGGGTCGCACCTGAGCCCCACC

ATCGCGACCCACCCCCTGGGACACCCAGATAAAACGCGTCGATTGCTAACGTGTTTTTCT

TGACTACAGGTTCACCTTCAGACCGGCCAATGCGTAAGTTGCTGTCACGCCAGA-CCTTA

TCATCGCCACCCATAGCACGTTTCCCAGGGTAACCAAATCGGTGCTGCCTTCTGGTGC--

-----GTCCACCACCGCGATACGACCTCGAACCATACTGACCTCGCAGGCAAACCATCTC

TGGCGAGCACGGCCTCGACAGCAATGGCGTGTATGCACCTCCTATTTCTACCTCTCT-GT

CTGCCCTGACAATCATACAGTTACAACGGCACTTCCGAGCTCCAGCTCGAGCGCATGAAC

GTCTACTTCAACGAGGTAAGTACGTCG--TTGGCCATCTGCAGCCTTGCTAACGCGTTAT

CAGGCCTCCGGCAACAAGTATGTCCCTCGCGCCGTCCTCGTCGATCTCGAGCCCGGTACC

ATGGACGCCGNN

>Diaporthe_hsinchuensis_HK_04_1

NNNNATAAGTCACAACC-ACATGCGCGCCCTTGCTGTTGCGCATGCTAACGGACCGTTTT

CGGCTTGTAGGATAAGGATGGCGATGGTTAGTGCGGACGTCTCCCCCCACCACGTGTCAC

GCTCGATCCGCCGCGACGGCCTGCGCGCAACCAAGCGTTATCATCATCGCGAGTTACTGA

GGTGTAGGACAAATCACCACCAAGGAGCTCGGCACGGTAATGCGATCCCTGGGTCAGAAT

CCGTCTGAGTCGGAGCTCCAAGATATGATTAACGAGGTCGACGCCGACAACAACGGCACC

ATTGACTTTGGTACGTCCAGATGTACGCCCGTCCGCGCCCTCCACCGGAGGTGTCAAGAA

GCCTCACCGCTACAAGCCTGGTACCGTCGCTCTGCGTGAGATCCGTCGCTACCAGAAGAG

CACCGAGCTGCTGATCCGCAAGCTCCCCTTCCAGCGTCTGGTATGCAGGTTCGTGAGATC

GCCCAGGACTTCAAGTCCGACCTGCGCTTCCAGTCTTCCGCCATCGGTGCCCTGCAGGAG

TCCGTCGAGTCTTACCTCGTCTCCCTCTTTGAGGACACCAACCTGTGCGCCATCCATGCC

AAGCGTGTCACCATCCAGTCGGTACGTNNNNNNNNNNNNNNNNNTGGGGGACGCGGAGGG

ACATTGCTGCCCCAGGCGCACCCAGAAACCCTTTGTGAACTTTTATTGTTGCCTCGGCAT

GCCGGGGGGCCCCTCGAGACGAGGAGCAGGCACGCCGGCGGCCAAGTTAACTCTTGTTTT

TACACTGAAACTCTAAATGAATCAAAACTTTCAACAACGGATCTCTTGGTTCTGGCATCG

ATGAAGAACGCAGCGAAATGCGATAAGTAATGTGAATTGCAGAATTCAGTGAATCATCGA

ATCTTTGAACGCACATTGCGCCCTCCGGTATTCCGGAGGGCATGCCTGTTCGAGCGTCAT

TTCAACCCTCAAGCATTGCTTGGTGTTGGGGCACTGAAGGGCAGGCCCTGAAATTCAGTG

GCGAGCTCGCCAGGACCCCGAGCGCAGTAGACCCTCGCTAAGGCCCTGGCGGTGCCCTGC

TGAAANNNNNNTATTCTCTGTGAACATCATCATACTCTTGCGGCTTTGTCAGCGCATTTT

CACCCCTCCCTCTGGATTTTCAGGGTGCGGGGCTTAGAGCTTATCTCACCACCACGACGG

AAACTAGGCTGACAACTATACAGCTGCCGAGCTTGGTAAGGGNNNNNNNNNNNNNNNNNN

NNNNNNNNNNNNNNNNNNNNNNNNNNNNNNNNNNNNNNNNNNNNNNNNNNNNNNNNNNNN

NNNNNNNNNNNNNNNNNNNNNNNNNNNNNNNNNNNNNNNNNNNNNNNNNNNNNNNNNNNN

NNNNNNNNNNNNNNNNNNNNNNNNNNNNNNNNNNNNNNNNNNNNNNNNNNNNNGCGGGGC

CA---GTCCACCGCCGCGATACGACCTCGAAGCATGCTGACCTCGTAGGCAAACCATCTC

TGGCGAGCACGGCCTCGACAGCAATGGCGTGTATGCACCTCCTATTCCTGCCTT------

CTGCCCTGACAATCACACAGTTACAACGGCACTTCCGAGCTCCAGCTCGAGCGCATGAAC

GTCTACTTCAACGAGGTAAGCACGCCA--TTAACCATCTGCAACCTTGCTAACGCGTTAT

CAGGCCTCCGGCAACAAGTATGTGCCCCGCGCCGTCCTCGTCGATCTCGAGCCCGGTACC

ATGGACGCCGTC

>Diaporthe_huangshanensis_CNUCC_201903

????????????????????????????????????????????????????????????

????????????????????????????????????????????????????????????

????????????????????????????????????????????????????????????

????????????????????????????????????????????????????????????

????????????????????????????????????????????????????????????

???????????????????????????????TCCGCGCCCTCCACCGGAGGTGTCAAGAA

GCCTCACCGCTACAAGCCTGGTACCGTCGCTCTGCGTGAGATCCGTCGCTACCAGAAGAG

CACTGAGCTGCTGATCCGCAAGCTCCCCTTCCAGCGTCTGGTATGCAGGTCCGTGAGATC

GCCCAGGACTTCAAGTCCGACCTCCGCTTCCAGTCCTCCGCCATCGGTGCCCTGCAGGAG

TCCGTCGAGTCTTACCTCGTCTCCCTCTTCGAGGACACCAACCTGTGCGCCATCCACGCC

AAGCGTGTCACCATCCAGTCGGTACGTNNNNNNNNNNNNNNNNNNNNNNNNNNNNNNGGG

GGCTTGGCT-ACTAGGCGCACCCAGAAACCCTTTGTGAACTTTT-TTGTTGCCTCGGCAT

GCTGGTAGGCCCCTC-CGGTGAGGAGACGGCACGCCGGCGGCCAAGTTAACTC-TGTTTT

TACACTGAAACTCTAAATGAATCAAAACTTTCAACAACGGATCTCTTGGTTCTGGCATCG

ATGAAGAACGCAGCGAAATGCGATAAGTAATGTGAATTGCAGAATTCAGTGAATCATCGA

ATCTTTGAACGCACATTGCGCCCTCTGGTATTCCGGAGGGCATGCCTGTTCGAGCGTCAT

TTCAACCCTCAAGCATTGCTTGGTGTTGGGGCACTGCGAAGCAGGCCCTGAAATCTAGTG

GCGAGCTCGCCAGGACCCCGAGCGTAGTAGACCCTCGCTAAGGCCCTGGCGGTGCCCTGC

TGAAANNNNNNNNNNNNNGTTTTTCCTCATCATGTTGGTACGGCTTCGTCAGCGCATTTT

CACCCCTCGCTCTGGATTTTCAGGGTGCGGGGCTTAGAGCTTATCT-----CTACCGCCG

GAAACATGCTGATATCTACATAGCCGCCGAGCTTGGTAAGGGNNNNNNNNNNNNNNNNNN

NNNNNNNNNNNNNNNNNNNNNNNNNNNNNNNNNNNNNNNNNNNNNNNNNNNNNNNNNNNN

NNNNNNNNNNNNNNNNNNNNNNNNNNNNNNNNNNNNNNNNNNNNNNNNNNNNNNNNNNNN

NNNNNNNNNNNNNNNNNNNNNNNNNNNNNNNNNNNNNNNNNNNNNNNNNNNNNNNNNNNN

NNNNNNNNNNNTAACGCGATATGACCTCGAGCCATACTGACCTCGCAGGCAAACCATCTC

TGGCGAGCACGGCCTCGACAGCAATGGCGTGTATGCACCTCCTATTCCTACCTTTCTCGT

CTGCCCTGACAATCATACAGTTACAACGGCACTTCCGAGCTCCAGCTCGAGCGCATGAAC

GTCTACTTCAACGAGGTAAGTACGTCG--TTGGCCATCTGCAGCTTTGCTAACGCGTTAT

CAGGCCTCCGGCAACAAGTATGTCCCTCGCGCCGTCCTCGTCGATCTCGAGCCCGGTACT

ATGGACGCCGTC

>Diaporthe_hubeiensis_JZB320123

CTTTGTAAGTCATTTCC--GGCACCCGCCCTTGCTGTTGTCCATGCTAACGGACCGTTTT

CGGCCCGCAGGATAAGGATGGCGATGGTTAGTGTGGTCACCCTCAGCTAGCACGCGTCAT

GCTCGAACCGCCGCGACGGCCTGCGCTCAAGCGACCGTCATCG-CATCACCAGTTGCTAA

GGTGCAGGACAAATCACCACCAAGGAGCTCGGCACGGTCATGCGGTCCCTGGGTCAAAAC

CCCTCCGAGTCTGAGCTGCAGGACATGATTAACGAGGTCGATGCCGACAACAATGGCACC

ATTGACTTTGGTAAGCCAAGATGCTCGCCCG?????????????????????????????

????????????????????????????????????????????????????????????

????????????????????????????????????????????????????????????

????????????????????????????????????????????????????????????

????????????????????????????????????????????????????????????

???????????????????????????NNNNNNNNNNNNNNNNNNNNNNNNNNNNNNNNN

NNNNNNNNNCTTC-GGCGCACCCAGAAACCCTTTGTG-ACTTATACTGTTGCCTCGGCTG

GCCGGGAGGCCCCCTGAGACAGGGAGCA-GCCCGCCGGCGGCCAAACAAACTCTTGTTTC

T-TAGTGAATCTCT-AATGAATCAAAACTTTCAACAACGGATCTCTTGGTTCTGGCATCG

ATGAAGAACGCAGCGAAATGCGATAAGTAATGTGAATTGCAGAATTCAGTGAATCATCGA

ATCTTTGAACGCACATTGCGCCCTCTGGTATTCCGGAGGGCATGCCTGTTCGAGCGTCAT

TTCAACCCTCAAGCCTGGCTTGGTGTTGGGGCACCGAAGGGCGGGCCCTGAAATCTAGTG

GCGAGCTCGCCAGGACCCCGAGCGTAGTAGA-TCTCGTTAAGGCCCTGGCGGTGCCCTGC

TGAAAGAGAAGGAAGGTTAGT------TACTGCGTTTGCGCGGCTTCGTCAGGGCATTTT

CACCCCTCCCTCTGGATTTTCAGGGTGCGGGGCTTACGGCTTATCTCGCTGCCACCACCA

ATCAGAAGCTGACATCTCTATAGCCGCCGAGCTCGGTAAGGGNNNNNNNNNNNNNNNNNN

NNNNNNNNNNNNNNNNNNNNNNNNNNNNNNNNNNNNNNNNNNNNNNNNNNNNNNNNNNNN

NNNNNNNNNNNNNNNNNNNNNNNNNNNNNNNNNNNNNNNNNNNNNNNNNNNNNNNNNNNN

NNNNNNNNNNNNNNNNNNNNNNNNNNNNNNNNNNNNNNNNGGTGCTGCTTTCTGGTGCGT

TCAGAGTCGACCACCGCGACAGTAGCTCGTGGCATACTGACATCGTAGGCAAACCATCTC

TGGCGAGCACGGCCTCGACAGCAATGGCGTGTATGCACCTCCTATTCCTGCCTATCTCGT

CCTACCTGATAATGGCACAGCTACAACGGCACTTCTGAGCTCCAGCTCGAGCGCATGAAC

GTCTACTTCAACGAGGTGAGCACGTTGTTTTGACCACCTGCAGCCTAGCTAACGCGTTAT

CAGGCCTCCGGCAACAAGTATGTGCCTCGCGCCGTCCTCGTCGATCTCGAGCCCGGTACC

ATGGATGCCGTC

>Diaporthe_humulicola_CT2018_1

CTTTGTAAGTCGTCTTTCACATGTACGCCCTTGCTGTTGCGCGTGCTAACGGACTA-TTT

CGGACTGCAGGATAAGGACGGTGATGGTTAGTCCGGGCGCTCCTAACTAGCACGCGTCAC

GCTTGATCTGCCGCGACGGCCTGCGCGCGAGCGAGCGTCTTCATTATCAGGAGTTGCTAA

GATGTAGGACAAATCACCACGAAGGAGCTCGGCACGGTCATGCGGTCCCTGGGCCAGAAC

CCGTCCGAGTCTGAGCTGCAAGATATGATTAACGAGGTCGACGCCGATAATAATGGCACC

ATTGACTTTGGTATGCCCACATTCTCGCCCANNNNNNNNNTCCACCGGAGGTGTCAAGAA

GCCTCACCGCTACAAGCCTGGTACCGTCGCTCTGCGTGAGATCCGTCGCTACCAGAAGAG

CACTGAGCTGCTGATCCGCAAGCTCCCCTTCCAGCGTCTGGTAAGCAGGTCCGTGAGATC

GCACAGGACTTCAAGTCCGACCTCCGCTTCCAGTCCTCGGCCATCGGCGCACTTCAGGAG

TCTGTCGAGTCTTACCTCGTCTCCCTCTTTGAGGACACCAACCTCTGCGCCATTCACGCC

AAGCGTGTCACTATCCAGTCGGTATGTAACAAGGTCTCCGTTGGTGAACCAGCGGAGGGA

TCATTGCTGCCCCAGGCGCACCCAGAAACCCTTTGTGAACTTTTACTGTTGCCTCGGCAG

GCCGGGGGGCCCCTCGAGACGAGGAGCAGGCCCGCCGGCGGCCAACCAAACTCTTGTTTT

TACACTGAAACTCTAAATGAATCAAAACTTTCAACAACGGATCTCTTGGTTCTGGCATCG

ATGAAGAACGCAGCGAAATGCGATAAGTAATGTGAATTGCAGAATTCAGTGAATCATCGA

ATCTTTGAACGCACATTGCGCCCTCTGGTATTCCGGAGGGCATGCCTGTTCGAGCGTCAT

TTCAACCCTCAAGCCTGGCTTGGTGCTGGGGCGCTGAGGGGCAGGCCCTGAAATCCAGTG

GCGAGCCCGCCAGGACCCCGAGCGCAGTAGACCCTCGCTACGGCCCTGGCGGTGCCCTGC

TGAAAGAGAAGGAAGGTCAGTAAACATCATCATGTCCGGGCGACTTCGTCCGCGCATTTT

CACCCCTCGCTCTGGATTTTCAGGGTGCGGGGCTTAGGGCTTATCTCGCCGCCGACACCA

AAACTGTGCTGACATCTACACAGCCGCTGAGCTTGGTAAGGGAGGTCGCCGAGTGCCTGC

AGGGTTTCCCACCCGCTGGTATACGCTGCTGATCCGCGAGGTTCCCCGACCGCCCACCTT

CTCCGTCGTACCCTCCCCCAAAGTCTCCGACACCGTCGTTGAGCCCTCACCCTGTCCGTC

CACCAGCTGTCGAGAACGAGACCTTCTGCATCGACAACGAGGCGCTGTCTGCATGCGCAC

GCCAACCCTCCTACGGCGGTCATGTCCGGCGTCACGGTCTCCCTGCGGTCAGCTGAACTC

TGACCTGCGCAAGCTGGCCGTGAACATGGTGCCCTTCCCCCGCACTTCTTCATGCTTTGC

CCCCT--------CCCACTCCTTCCGCGCCGTCACCGTTCCCGAGCTCACCCAGCAGATG

TTCGACCCTAAGAACATGATGGCAACGGCCTGACGTGCTCTGCGCGCGCTGACAATCTAG

CACGTCCAGAGCAACGAGTGGATCCCCAACAACGTCCTCGTCGGTAACTCGACTGCTATC

CAGGA-GCTGTT

>Diaporthe_hunanensis_HNZZ023

NNNNNNNNNNNNNNNNNNNNNNNNNNGCCCTTGCTGTTGCGCATGCTAACGGACCGTTTT

CGGCTCGTAGGATAAGGATGGCGATGGTTAGTGCGGCCGCTCTCACACAGCACGCGTCAT

GCTCGATCCTCCGCGACGGCCTGCGCGCCACCAAGCGTTATCACTATTGCGAGTTGCTGA

GGTGCAGGACAAATCACCACCAAGGAGCTCGGCACAGTCATGCGTTCCCTGGGCCAGAAC

CCTTCCGAGTCTGAGCTGCAAGATATGATTAACGAGGTCGACGCCGACAACAACGGCACC

ATTGACTTTGGTACGTCCAGATATACGCCCATCCGCGCCCTCCACCGGAGGTGTCAAGAA

GCCTCACCGCTACAAGCCTGGTACCGTCGCTCTGCGTGAGATCCGTCGCTACCAGAAGAG

CACTGAGCTGCTGATCCGCAAGCTCCCCTTCCAGCGTCTGGTATGCAGGTCCGTGAGATC

GCCCAGGACTTCAAGTCCGACCTCCGCTTCCAGTCCTCCGCCATCGGTGCCCTGCAGGAG

TCCGTCGAGTCTTACCTCGTCTCCCTCTTCGAGGACACCAACCTGTGCGCCATCCACGCC

AAGCGTGTCACCATCCAGTCGGTACGTNNNNNNNNNNNNNNNNNNNNNNNNNNNNNNNNN

NNNNNNNNNNNNNNNNNNNNNNNNNNNNCCCTTTGTGAACTTTTACTGTTGCCTCGGCAT

GCTGGGGGGCCCCCTGAGACAGGGAGCAGGCACGCCGGCGGCCAAGTTAACTCTTGTTTT

TACACTGAAACTCTAAATGAATCAAAACTTTCAACAACGGATCTCTTGGTTCTGGCATCG

ATGAAGAACGCAGCGAAATGCGATAAGTAATGTGAATTGCAGAATTCAGTGAATCATCGA

ATCTTTGAACGCACATTGCGCCCTCTGGTATTCCGGAGGGCATGCCTGTTCGAGCGTCAT

TTCAACCCTCAAGCATTGCTTGGTGTTGGGGCACTGCGAAGCAGGCCCTGAAATCTAGTG

GCGAGCTCGCTAGGACCCCGAGCGTAGTAGACCCTCGCTAAGGCCCTGGCGGTGCCCTGC

TGAAANNNNNNNAAGGTTAGTAAACATCATCACGTTGGTACGGCTTCGTCAGCGCATTTT

CACCCCTCGCTCTGGATTTTCAGGGTGCGGGGCTTAGAGCTTATCT-----CTACCGCTG

GAAACATGCTGATATCTACATAGCCGCCGAGCTTGGTAANNNNNNNNNNNNNNNNNNNNN

NNNNNNNNNNNNNNNNNNNNNNNNNNNNNNNNNNNNNNNNNNNNNNNNNNNNNNNNNNNN

NNNNNNNNNNNNNNNNNNNNNNNNNNNNNNNNNNNNNNNNNNNNNNNNNNNNNNNNNNNN

NNNNNNNNNNNNNNNNNNNNNNNNNNNNNNNNNNNNNNNNNNTGCTGCTTTCTGGTGC--

-----GTCCACCACCGCGACATGACCTCGAGCCATACTGACCTCGCAGGCAAACCATCTC

TGGCGAGCACGGCCTCGACAGCAATGGCGTGTATGCACCTCCTATTTCTGCCTTTCTCGT

CTGCCCTGACAATCACACAGTTACAACGGCACTTCCGAGCTCCAGCTCGAGCGCATGAAC

GTCTACTTCAATGAGGTAAGTACGTCA--TTGACCATCTGCAGCCTTGCTAACGCGTTAT

CAGGCCTCCGGCAACAAGTATGTCCCTCGCGCCGTCCTCGTCGATCTCGAGCCCGGTACT

ATGGACGCCGTN

>Diaporthe_hungariae_CBS_143353

NNNNNNNNNNNNNNNTCCACATGCCCGCCCTTGCTGTTGCGCATGCTAACGGACCGTTTT

CGGCCTGCAGGATAAGGATGGCGATGGTTAGTGTGCCCGCACCCACGGAGCACGCGTCAT

GCTCGATCCGCCGCGACGGCCTGCGCGCGAACAGGCGTCAGCACTATCAGGGATTGCTAA

GGCGTAGGACAAATCACCACGAAGGAGCTCGGCACTGTCATGCGGTCTCTGGGCCAGAAC

CCGTCCGAGTCTGAACTGCAAGATATGATTAACGAGGTCGATGCCGACAACAATGGCACC

ATTGACTTTGGTACGTCCACACGCTCGCCCATCCGCGCCCTCCACCGGAGGTGTCAAGAA

GCCTCACCGCTACAAGCCTGGTACTGTCGCTCTGCGTGAGATCCGTCGTTACCAGAAGAG

CACCGAGCTGCTGATCCGCAAGCTCCCCTTCCAGCGTCTGGTATGCAGGTCCGTGAGATC

GCCCAGGACTTCAAGTCCGACCTCCGCTTCCAGTCTTCCGCCATCGGTGCCCTGCAGGAG

TCCGTCGAGTCTTACCTCGTCTCCCTCTTTGAGGATACCAACCTGTGCGCCATCCACGCC

AAGCGTGTCACCATCCAGTCGGTATGTAACAAGGTCTCCGTTGGTGAACCAGCGGAGGGA

TCATTGCTGCCCCTGGCGCACCCAGAAACCCTTTGTGAACTCTTACCGTTGCCTCGGCAG

GCCGGGGGGCCCCTCGAGACGAGGAGCAGGCCCGCCGGCGGCCAAGTTAACTCTTGTTTT

TACACTGAAACTCTAAATGAATCAAAACTTTCAACAACGGATCTCTTGGTTCTGGCATCG

ATGAAGAACGCAGCGAAATGCGATAAGTAATGTGAATTGCAGAATTCAGTGAATCATCGA

ATCTTTGAACGCACATTGCGCCCTCTGGTATTCCGGAGGGCATGCCTGTTCGAGCGTCAT

TTCAACCCTCAAGCCTGGCTTGGTGATGGGGCACTGGGGAGCAGGCCCTGAAATCCAGTG

GCGAGCTCGCCAGGACCCCGAGCGCAGTAGACCCTCGCTGAGGCCCTGGCGGTGCCCTGC

TGAAAGAGAAGGAAGGTTAGTAAACATCATCGCGCCTGTGCAGCTCCGTCAGCGCATTTT

CACCCCTCGTTCTGGATTTTCAGGGTGCGGGGCTTAGAGCTTATCTCCCCACCACCGCCG

GAATGAAGCTAACATCTATACAGCCGCCGAGCTTGGCAAGGGNNNNNNNNNNNNNNNNNN

NNNNNNNNNNNNNNNNNNNNNNNNNNNNNNNNNNNNNNNNNNNNNNNNNNNNNNNNNNNN

NNNNNNNNNNNNNNNNNNNNNNNNNNNNNNNNNNNNNNNNNNNNNNNNNNNNNNNNNNNN

NNNNNNNNNNNNNNNNNNNNNNNNNNNNNNTAACCAAATTGGTGCTGCTTTCTGGTGCGT

TCCAAGTCCACCGCCGCGATACGACCTCGAAGCATGCTGACCTCGTAGGCAAACCATCTC

TGGCGAGCACGGCCTCGACAGCAATGGCGTGTATGCACCTCCTATGCCTGTCCATCTTGA

CTTCCCTGACAATCGCACAGTTACAACGGCACTTCTGAGCTCCAGCTCGAGCGCATGAAC

GTCTACTTCAACGAGGCAAGTACAACATTCCGACCATCTCCAAGTTCGCTAACGCGTTAT

CAGGCCTCCGGCAACAAGTATGTTCCTCGCGCCGTCCTCGTCGATCTCGAGCCCGGTACC

ATGGACGCCGTC

>Diaporthe_iberica_CECT_21218

NNNNNNNNNNNNNNNNNNNNNNNNNNNNNNNNNNNNNNNCGCATGCTAACAGACCGTTTT

CGGCCTGCAGGATAAGGATGGCGATGGTTAGTGTGGCCACCCTCAGCTAGCACGCGTCAT

ACTCGATCCGCCGCGACGGTCTGCGCGTAAGCGAACGTCGTCG-CATCATCAATTGCTAA

GGTGTAGGGCAAATCACCACCAAGGAGCTCGGCACGGTCATGCGGTCCCTGGGTCAAAAC

CCCTCCGAGTCCGAGCTGCAGGACATGATTAACGAGGTGGATGCCGACAACAATGGCACC

ATTGACTTNNNNNNNNNNNNNNNNNNNNNNNTCCGCGCCCTCCACCGGAGGTGTCAAGAA

GCCTCACCGCTACAAGCCTGGTACCGTCGCTCTGCGTGAGATCCGTCGCTACCAGAAGAG

CACCGAGCTGCTGATCCGCAAGCTCCCCTTCCAGCGTCTGGTATGCAGGTCCGTGAGATC

GCCCAGGACTACAAGTCCGACCTGCGCTTCCAGTCTTATGCCATCGGTGCCCTGCAGGAG

TCCGTCGAGTGTTACCTCGTCTCCCTCTTCGAGGACACCAACCTGTGCGCCATCCACCCC

AAGCGTGTCACCATCCNNNNNNNNNNNNNNNNNNNNNNNNNNNNNNNNNNNNNNNNNNNN

NNNNNNNNNNNNNNNNNNNNNNNNNNNNNNNNNNNNNNNNNNTTACTGTTGTCTCGGTAG

GCCGGGAGGCCCCCTGAGACGGGGAGCA-GCCCGCCGGCGGCCAACCAGACTCTTGTTTC

T-TAGTGAATCTCT-AATGAATCAAAACTTTCAACAACGGATCTCTTGGTTCTGGCATCG

ATGAAGAACGCAGCGAAATGCGATAAGTAATGTGAATTGCAGAATTCAGTGAATCATCGA

ATCTTTGAACGCACATTGCGCCCTCTGGTATTCCGGAGGGCATGCCTGTTCGAGCGTCAT

TTCAACCCTCAAGCATTGCTTGGTGTTGGGGCACGGAAGGGCCGGCCCTGAAATCTAGTG

GCGAGCTCGCCAGGACCCCGAGCGTAGTAGTATCTCGTTAAGGCCCTGGCGGTGCCCTGC

NNNNNNNNNNNNNNNNNNNNNNNNNNNNNNNNCGTTTGTGCGGCTTCGTCAAGGCATTTT

CACCCCTCGCTCTGGATTTTGATGGTGCGGGGCTTACCGCTTATCTCTCTGCTACCAACA

CTTACAGTCTCACACCTGAAAATATCTCGATCGTGTGTGTTGTCGCACCTCAGCCCCACC

ATCGCGACCCACCCCCTGGGACACCAGCATAAGACGCGTCGATTGCTAACATGTTTTTCT

CGCCCACAGGTTCACCTTCAGACCGGCCAATGCGTAAGTTGCTGTCACCACCGCACCTTA

T---CGCCACCTGTAGCACGTTTCCCAGGGTAACCAAATCGGTGCTGCTTTCTGGTGCGT

ACCAAGTCGACCACCGCGACACTAGCTCGTAATATGCTGACCTCGCAGGCAAACCATCTC

TGGCGAGCACGGCCTCGACAGCAATGGCGTGTATGCACCTCCTATTCCTAACGGTCTTGT

CCTCCTTAACAATTGCACAGTTACAACGGCACCTCCGAGCTCCAGCTCGAGCGCATGAAC

GTCTACTTCAACGAGGTAAGTACGTCGTTGTGACCACCCACAGCCTCGCTAACGCGTTAC

CAGGCCTCCGGCAACAAGTATGTGCCTCGCGCCGTCCTCGTCGATCTCGAGCCCGGTACC

ATGGACGCCGTC

>Diaporthe_ilicicola_FPH2015502

????????????????????????????????????????????????????????????

????????????????????????????????????????????????????????????

????????????????????????????????????????????????????????????

????????????????????????????????????????????????????????????

????????????????????????????????????????????????????????????

???????????????????????????????TCCGCGCCATCCACCGGAGGTGTCAAGAA

GCCTCACCGCTACAAGCCCGGTACCGTCGCTCTGCGTGAGATTCGTCGCTACCAGAAGTC

GACCGAGCTCCTGATCCGCAAGCTCCCCTTCCAGCGTCTGGTATGCAGGTTCGTGAGATT

GCCCAGGACTTCAAGTCCGATCTCCGCTTCCAGTCTTCCGCCATCGGTGCTCTGCAGGAG

TCCGTCGAGTCTTACCTCGTCTCTCTCTTCGAGGATACCAACCTGTGCGCCATCCACGCC

AAGCGTGTCACCATCCAGTCGGTATGTNNNNNNNTCTCCGTTGGTGAACCAGCGGAGGGA

TCATTGCTGCCCCCGGCGCACCCAGAAACCCTTTGTGAACTTTATTTGTTGCCTCGGAAG

GCTGGGGGGCCCCCCCCGGTGGGGAGCAGGCCCGCCGGCGGCCAAGCAAACTCTTGTTTT

TAC-CTGGAACTCTAAATGAATCAAAACTTTCAACAACGGATCTCTTGGTTCTGGCATCG

ATGAAGAACGCAGCGAAATGCGATAAGTAATGTGAATTGCAGAATTCAGTGAATCATCGA

ATCTTTGAACGCACATTGCGCCCTCTGGTATTCCGGAGGGCATGCCTGTTCGAGCGTCAT

TTCAACCCTCAAGCCTGGCTTGGTGATGGGGCACTGGGAAGCAGGCCCTGAAATACAGTG

GCGAGCCCGCCAGCACCCCGAGCGCAGTAGACCCTCGCTAAGGTGCTGGTGGTGCCCTGC

TGAAA???????????????????????????????????????????????????????

????????????????????????????????????????????????????????????

??????????????????????????????????????????TCGCACCTGAGCCCCACC

ATCGCGACCCACCCCCCGAGACACCCAAATAAGACGCGTCGAATGCTAACCTGTTTTCCT

CATCCACAGGTTCACCTCCAGACCGGCCAATGCGTAAGTTGCTGTCACCACCGCACCCTA

T---CGCCCCCTGTAGCACGTTTTCCAGGGTAACCAAATCGGTGCTGCTTTCTGGTGCGT

C----GTCCATCATCGCGACACGACCTTACAACATACTGACCTCGTAGGCAAACCATCTC

TGGCGAGCACGGCCTCGACAGCAATGGCGTGTATGCACCTCCTATTACTGCCTATTTTGT

CTACGCTGACGGCCTCACAGTTACAACGGCACTTCTGAGCTCCAGCTCGAGCGCATGAAC

GTCTACTTCAACGAGGTAAGTGTGCTATTTCCACGGAATCCAGCTGTGCTGACACGTTAT

CAGGCCTCCGGCAACAAGTATGTGCCTCGCGCCGTCCTCGTCGATCTCGAGCCCGGTACC

ATGGACGCCGTC

>Diaporthe_impulsa_CBS_114434

CTTTGTAAGTCATTCTCCACATGCCCGCCCTTGCTGTTGCGCATGCTAACGGACCGTTTT

CGGCCTGCAGGATAAGGATGGCGATGGTTAGTGTGGCCGCACCCACGGAGCACGCGTCAT

GCTCGATACGGCGCGACGGCCTGCGCGCGAGCAGGCGTCAGCACTATCAGGGATTGCTAA

GGCGTAGGACAAATCACCACGAAGGAGCTCGGCACTGTCATGCGGTCTCTGGGTCAGAAC

CCGTCCGAGTCTGAACTGCAAGATATGATTAACGAGGTCGATGCCGACAACAATGGCACC

ATTGACTTTGGTACGTCCAGACGCTCGCCCATCCGCGCCCTCCACCGGAGGTGTCAAGAA

GCCTCACCGCTACAAGCCTGGTACCGTCGCTCTGCGTGAGATCCGTCGCTACCAGAAGAG

CACCGAGCTGCTGATCCGCAAGCTCCCCTTCCAGCGTCTGGTATGCAGGTCCGTGAGATC

GCCCAGGACTTCAAGTCCGACCTCCGCTTCCAGTCTTCCGCCATCGGTGCCCTGCAGGAG

TCCGTCGAGTCTTACCTCGTCTCCCTCTTCGAGGACACCAACCTGTGCGCCATCCACGCC

AAGCGTGTCACCATCCAGTCGGTATGTAACAAGGTCTCCGTTGGTGAACCAGCGGAGGGA

TCATTGCTGCCCCCGGCGCACCCAGAAACCCTTTGTGAACTTTTACTGTTGCCTCGGCAG

GCCGGGGGGCCCCTCGAGACGAGGAGCAGGCCCGCCGGCGGCCAAGTCAACTCTTGTCTC

TACACTGAAACTCTAAATGAATCAAAACTTTCAACAACGGATCTCTTGGTTCTGGCATCG

ATGAAGAACGCAGCGAAATGCGATAAGTAATGTGAATTGCAGAATTCAGTGAATCATCGA

ATCTTTGAACGCACATTGCGCCCTCTGGTATTCCGGAGGGCATGCCTGTTCGAGCGTCAT

TTCAACCCTCAAGCCTGGCTTGGTGATGGGGCACTGAAGGGCAGGCCCTGAAATTCAGTG

GCGGGCTCGCCAGGACCCCGAGCGCAGTAGACCCTCGCTAAGGCCCTGGCGGTGCCCTGC

TGAAAGAGAAGGAAGGTTAGTAAACATCATCGCGCCTGCGCAGCTCCGTCAGCGCATTTT

CACCCCTCGTTCTGGATTTTCAGGGTGCGGGGCTTAGAGCTTATCTCACCACCACC---G

GAAAGAAGCTAACATCTATACAGCCGCCGAGCTTGGTAAGGGTCGCACCTGAGCCCCACC

ATCGCGACCCAGCCCCTGGGACACCCAGATAAGACGCGTCGATTGCTAACGTGTTTTTCT

CGCCCGCAGGTTCACCTTCAGACCGGCCAATGCGTAAGTTGTTGTCACCACCGGACCTCG

T---CGCCACCTGTAGCACGTTTCCCAGGGTAACCAAATCGGTGCTGCTTTCTGGTGCGT

CCCAAACTCGCCACCGCGATACGACCTCGTGGCATACTGACCTCGTAGGCAAACCATCTC

TGGCGAGCACGGCCTCGACAGCAATGGCGTGTATGCACCTCCTATTCCTGCACATCTCGC

CCTCCCTGACAATTGCACAGTTACAACGGCACTTCCGAGCTCCAGCTCGAGCGCATGAAC

GTCTACTTCAACGAGGTATGTGCGTTTTTCCGGTCATCGCCAACCTTGCTAACGCCTTAT

CAGGCCTCCGGCAACAAGTATGTTCCTCGCGCTGTCCTCGTCGATCTCGAGCCCGGTACC

ATGGATGCCGTC

>Diaporthe_incompleta_CGMCC_3_18288

CTTTGTAAGTCGTCTTCGACATGCCCGCCCTTGCTGTCGCGCATGCTAACGGACCGTTTT

CGGCTTGCAGGATAAGGATGGCGATGGTTAGTGCGGCCGCTCTCACCTAGCACGCGTCAT

GCTCGATCCGTCGCGACGGCCTGCGCGCAAGCAGGCGATATCACTTTCACGAGTTACTGA

GGTGTAGGACAAATCACCACCAAGGAGCTCGGCACGGTCATGCGGTCCCTGGGCCAGAAC

CCGTCCGAGTCTGAGCTGCAAGACATGATTAACGAGGTCGACGCCGACAACAATGGCACC

ATCGACTTTGGTACGTCCATATGCTCGCCCATCCGCGCCCTCCACCGGAGGTGTCAAGAA

GCCTCACCGCTACAAGCCTGGTACCGTCGCTCTGCGTGAGATCCGTCGTTACCAGAAGAG

CACCGAGCTGCTGATCCGCAAGCTCCCCTTCCAGCGTCTGGTATGCAGGTTCGTGAGATC

GCCCAGGACTTCAAGTCCGACCTGCGCTTCCAGTCTTCCGCCATCGGTGCCCTGCAGGAG

TCCGTCGAGTCTTACCTCGTCTCCCTCTTTGAGGACACCAACCTGTGCGCCATCCACGCC

AAGCGTGTCACCATCCAGTCGGTACGTNNNNNNNNNNNNNNNNNNNNNNNNNNNNNNNNN

NNNNNNNNNNNNNNNNNNNNCCCAGAAACCCTTTGTGAACTC-TACTGTTGCCTCGGTTA

GCTGGTGGGCCCCCTGAGACAGGGAGCG-GCGCGCCGGCGGCCAACTTAACTCTGTTTCT

T--AGTGAATCTCTAAATGAATCAAAACTTTCAACAACGGATCTCTTGGTTCTGGCATCG

ATGAAGAACGCAGCGAAATGCGATAAGTAATGTGAATTGCAGAATTCAGTGAATCATCGA

ATCTTTGAACGCACATTGCGCCCTCTGGTATTCCGGAGGGCATGCCTGTTCGAGCGTCAT

TTCAACCCTCAAGCCTTGCTTGGTGTTGGGGCACTGGAGAGCAGGCCCTGAAATTCAGTG

GCGAGCTCGCCAGGACTCCGAGCGTAGTAG-ATCTCGCTAAGG-CCTGGCGGTGCCCTGC

TGAAAGAGAAGGAAGGTTAGTAAACACCATCATGGTTGCGACTGTCCGTTGGCGCATTTT

CACCCCTCGCTCTGGAATTTCAGGGTGCGGGGCTTAGGGCTTATCTCACCATAATCTTCA

CAACCATGCTGATATCCCCACAGCCGCCGAGCTTGGTAANNNNNNNNNNNNNNNNNNNNN

NNNNNNNNNNNNNNNNNNNNNNNNNNNNNNNNNNNNNNNNNNNNNNNNNNNNNNNNNNNN

NNNNNNNNNNNNNNNNNNNNNNNNNNNNNNNNNNNNNNNNNNNNNNNNNNNNNNNNNNNN

NNNNNNNNNNNNNNNNNNNNNNNNNNNNNNNNNNNNNNNNGGTGCTGCTTTCTGGTGCGT

CC-AAGTCCACCGCCGCGAGTCGACGCCGAAGCATACTGACCTCGCAGGCAAACCATCTC

TGGCGAGCACGGTCTCGACAGCAATGGCGTGTATGCACCTCCTATTTCTGTCTTTCTCGT

-TGCCCTGACAATCACACAGTTACAACGGCACTTCCGAGCTCCAGCTCGAGCGCATGAAC

GTCTACTTCAACGAGGTAAGTACGTTG--TTGACCATCTAAAGCTCTGCTAATGCGTTAT

CAGGCCTCCGGCAACAAGTATGTGCCCCGCGCCGTCCTCGTCGATCTCGAGCCCGGTACC

ATGGACGCCGTC

>Diaporthe_inconspicua_CBS_133813

NNNNNNNNNNNCCAGCCGATATGCCCGCCCTTGCTGTTGCGCATGCTAACGGACCGTTCT

CGGCTTGCAGGATAAGGATGGCGATGGTTAGTGCGGCCGGTCCCATACGACACGCGTCGT

GCTCGATCCGCCGCGATGGCCTGCGCGCAACCGAGCGTCACCACTACCACGAGTTGCTGA

GGTGTAGGACAAATCACCACCAAGGAGCTCGGCACAGTCATGCGGTCCCTGGGCCAGAAC

CCGTCCGAGTCTGAGCTGCAAGATATGATTAACGAGGTCGATGCCGACAACAATGGCACC

ATTGACTTTGGTATGTTCAGATGCTCACCCGTCCGCGCCCTCCACCGGAGGTGTCAAGAA

GCCTCACCGCTACAAGCCTGGTACCGTCGCTCTGCGTGAGATCCGTCGTTACCAGAAGAG

CACCGAGCTTCTGATCCGCAAGCTCCCCTTCCAGCGTCTGGTATGCAGGTTCGTGAGATC

GCCCAGGACTTCAAGTCCGACCTCCGCTTCCAGTCCTCCGCCATCGGTGCCCTGCAGGAG

TCCGTCGAGTCTTACCTCGTCTCCCTGTTCGAGGACACCAACCTGTGCGCCATCCACGCT

AAGCGTGTCACCATCCAGTCGGTACGTAACAAGGTCTCCTTTGTTGAACCAGCGGAGGGA

TCATTGCTGCCCCTGGCGCACCCAGAAACCCTTTGTGAACTTTTACTGTTGCCTCGGCAG

GCCGGGGGGTCCCTC--GACGAGGAGCA-GGCGGCCGGTGGCCAAGTTAACTC-TGTTTT

TACACTGAAACTCTAAATGAATCAAAACTTTCAACAACGGATCTCTTGGTTCTGGCATCG

ATGAAGAACGCAGCGAAATGCGATAAGTAATGTGAATTGCAGAATTCAGTGAATCATCGA

ATCTTTGAACGCACATTGCGCCCTCTGGTATTCCGGAGGGCATGCCTGTTCGAGCGTCAT

TTCAACCCTCAAGCCTGGCTTGGTGTTGGGGCACTGAAGGGCATGCCCTGAAATATAGTG

GCGAGCTCGCCAGGACTCCGAGCGTAGTAGACCCTCGCTAAGG-CCTGGCGGTGCCCTGC

TGAAAGAGAAGGAAGGTTAGTAGACATTCCCCTGTTCTGGCGGCTTGGTCGGCGCATTTT

GACCCCTCCCTATGGACTTTCAGGGTGCGGGGCTTAGAGCTTATCTCACC---------G

AAAAAATGCTGANNNNNNNNNNNNNNNNNNNNNNNNNNNNNNTCGCACCTGAGCCCCACC

ATCGCGACC-----CCTGGGACACCCAGCTAAGACGCGTCGATTGCTAACTTGTTTTCCC

CGCCTATAGGTTCACCTTCAGACCGGCCAATGCGTAAGTTGCTGTCACACCAGA-CCTTA

TCATCGCCACCTGTAGCACGTTTCCCAGGGTAACCAAATCGGTGCTGCTTTCTGGTGCGT

CC-AAGTCCACCACCGCGACTCGACCCTGAAGCATACTGACCTCGCAGGCAAACCATCTC

TGGCGAGCACGGTCTCGACAGCAATGGCGTGTATGCACCTCCTATTACTGTCTTTCTCGT

-TACCCTGACAATCACATAGTTACAACGGCACTTCCGAGCTTCAGCTCGAGCGCATGAAC

GTCTACTTCAACGAGGTAAGTACGCTG--TTGACCACCTACAGCTTTACTGACGCGTTCT

CAGGCCTCCGGCAACAAGTATGTGCCCCGCGCCGTCCTCGTCGATCTCGAGCCCGGTACC

ATGGACGCCGTC

>Diaporthe_infecunda_CBS_133812

CTTTGTAAGTTATCTTCGCCTGGCCCGCCGTTGCTTTTGCGCATGCTAACGGACCGTTTT

CGGCCTGCAGGATAAGGATGGCGATGGTTAGTGTGGTCACGCTCAGCTAGCACGCGTCAT

ACTCGATCCGCCGCGACGGTCTGCGCGCGAGCGACCGTCATCACTATCACGAGTTGCTAA

GGTGTAGGACAAATCACCACCAAGGAGCTCGGCACAGTCATGCGGTCCCTTGGTCAAAAC

CCTTCCGAGTCCGAGCTGCAGGACATGATCAACGAGGTCGACGCCGACAACAATGGCACC

ATTGACTTTGGTAAGTCTGGATGCTCATCCCTCCGCGCCCTCCACCGGAGGTGTCAAGAA

GCCTCACCGCTACAAGCCTGGTACCGTCGCTCTGCGTGAGATCCGTCGCTACCAGAAGAG

CACCGAGCTGCTGATCCGCAAGCTGCCCTTCCAGCGTCTGGTATGTAGGTCCGTGAGATC

GCCCAGGACTTCAAGTCCGACCTGCGCTTCCAGTCATCCGCCATCGGTGCCCTTCAGGAG

TCCGTCGAGTCTTACCTCGTCTCCCTCTTTGAGGACACCAACCTGTGCGCCATCCACGCC

AAGCGTGTCACCATCCAGTCGGTACGTAACAAGGTCTCCGTTGGTGAACCAGCGGAGGGA

TCATTGCTGCTTC-GGCGCACCCAGAAACCCTTTGTGAACTTA--TTGTTGCCTCGGTAG

GCCGGGAGGCCCCCTGAAACAGGGAGCA-GCCCGCCGGCGGCCAACTAAACTCTTGTTTC

TATAGTGAATCTCTAAATGAATCAAAACTTTCAACAACGGATCTCTTGGTTCTGGCATCG

ATGAAGAACGCAGCGAAATGCGATAAGTAATGTGAATTGCAGAATTCAGTGAATCATCGA

ATCTTTGAACGCACATTGCGCCCCCTGGTATTCCGGGGGGCATGCCTGTTCGAGCGTCAT

TTCAACCCTCAAGCCTGGCTTGGTGATGGGGCACTGAGGAGCAGGCCCTGAAATTCAGTG

GCGAGCTCGCCAGGACCCCGAGCGTAGTAGA-TCTCGCTAAGGCCCTGGCGGTGCCCTGC

TGAAAGAGAAGGAAGGTCAGTAAATATCATGGTGTTTGCGCGGCTGCGTCGAGCCATTTT

CACCCCTCCCTCTGGATTTTCAGGGTGCGGGGCTTACCGCTTATCTCACTGCCAGCACCG

ACAACATGCTGATTCCCACACAGCCGCCGAGCTGGGTAAGGGTCGCACCTCAGCCCCACC

ATCGCGACCCACCCCCTGCGACACCCAGATAAGACGCGTCGATTGCTAACATGTTTTTCT

TGCCCACAGGTTCACCTTCAGACCGGCCAATGCGTAAGTTGCTGTCACCGCCCGACCTTA

T---CGCCACCCATAGCACGTTTCCCAGGGTAACCAAATCGGTGCTGCTTTCTGGTGCGT

ACCGAGCCTGCCACCGCGAGACCACCTCCAAGCATACTGACCTTGTAGGCAAACCATCTC

TGGCGAGCACGGTCTCGACAGCAATGGCGTGTATGTACCTCCTATTCCTGCCCATCTCGT

CCTCCCTGATGATCGCACAGTTACAACGGCACTTCCGAGCTCCAGCTCGAGCGCATGAAC

GTCTACTTCAACGAGGTAAGCACGTCTTTTTGA-CCGTCTCGGCCTTGCTAATGCGCTCT

TAGGCCTCCGGCAACAAGTATGTGCCTCGCGCCGTCCTCGTCGATCTCGAGCCCGGTACC

ATGGACGCCGTC

>Diaporthe_infertilis_CBS_230_52

CTTTGTAAGTTATCTTCGCCTTGCCCGCCGTTGCCGTTGCGCATGCTAACTGACCGTTTT

CGGCCTACAGGATAAGGATGGCGATGGTTAGTGCGGTCACTCTCAGCTAGCACGCGTCAT

ACTCGATCCGCCGCGACGGTCTGCGCGCGAGCGACCGTCATCAATATCACGAGTTGCTAA

GGTGCAGGACAAATCACCACCAAGGAGCTCGGCACAGTCATGCGGTCCCTGGGCCAGAAC

CCTTCCGAGTCCGAGCTGCAGGACATGATCAACGAGGTCGACGCCGACAACAACGGCACC

ATTGACTTTGGTGAGTCCAGATTCTCGCACATCCGCGCCCTCCACCGGAGGTGTCAAGAA

GCCTCACCGCTACAAGCCTGGTACCGTCGCTCTGCGTGAGATCCGTCGCTACCAGAAGAG

CACCGAGCTGCTGATCCGCAAGCTCCCCTTCCAGCGTCTGGTATGCAGGTCCGTGAGATC

GCCCAGGACTTCAAGTCCGACCTGCGCTTCCAGTCTTCCGCCATCGGTGCGCTCCAGGAG

TCCGTCGAGTCTTACCTCGTCTCCCTCTTTGAGGACACCAACCTGTGCGCCATCCACGCC

AAGCGTGTCACCATCCAGTCGGTACGTAACAAGGTCTCCGTTGGTGAACCAGCGGAGGGA

TCATTGCTGCTTC-GGCGCACCCAGAAACCCTTTGTGAACTTA--CTGTTGCCTCGGCAG

GCCGGAAGGCCCCCTGAGACAGGGAGCA-GCCCGCCGGCGGCCAACTAAACTCTTGTTTC

TATAGTGAATCTCTAAATGAATCAAAACTTTCAACAACGGATCTCTTGGTTCTGGCATCG

ATGAAGAACGCAGCGAAATGCGATAAGTAATGTGAATTGCAGAATTCAGTGAATCATCGA

ATCTTTGAACGCACATTGCGCCCTCTGGTATTCCGGAGGGCATGCCTGTTCGAGCGTCAT

TTCAACCCTCAAGCCTGGCTTGGTGATGGGGCACTGCAGGGCAGGCCCTGAAATCTAGTG

GCGAGCTCGCCAGGACCCCGAGCGTAGTAGA-TCTCGCTAAGGCCCTGGCGGTGCCCTGC

TGAAAGAGAAGGAAGGTTAGTAAATATCGTCGCGTCTGCGAGGCTTCGTCAAGGCATTTT

CCCCCCTCCCTCTGGATTTTCAGGGTGCGGGGCTTACCGCTTATCTCAGTCAT-GCAC-G

AAACCATGCTGATTCCTACACAGCCGCTGAGCTGGGTAAGGGTCGCACATCAGCCCCACC

ATCGCGACCCACCCCCTTCGACACCCAGATAACACGCGTCGATTGCTAACATGTTTTCCT

CGCCCACAGGTTCACCTTCAGACCGGCCAATGCGTAAGTTACTGTCACCGCCAGACCTTA

T---CGCCACCCGTAGCACGTTTCCCAGGGTAACCAAATCGGTGCTGCTTTCTGGTGCGT

CCCAAGCCTACTGCCGCGAGGCGAGCTCGAAGCATACTGACCTCGTAGGCAAACCATCTC

TGGCGAGCACGGTCTCGACAGCAATGGCGTGTATGTACCTCCTATTCCTGCCGACCTCGT

CCTCCCTGATGATCGCACAGTTACAACGGCACTTCCGAGCTCCAGCTCGAGCGCATGAAC

GTCTACTTCAACGAGGTAAGTACGTTGCTTTGA-GCCTCTCGCCCTAGCTAACGCGTATT

CAGGCCTCCGGCAACAAGTATGTCCCCCGCGCCGTCCTCGTCGATCTCGAGCCCGGTACC

ATGGACGCCGTC

>Diaporthe_irregularis_CGMCC_3_20092

NNNNNNNNNNNNNNNNNGACACGCCCGCCCTTGCTGTTGCGCACGCTAACGGACCGTTTT

CGGCTTGCAGGATAAGGATGGCGATGGTTAGTGCGGCTGCTCTCACCCGGCACGCGTCAC

GCTCGATCCGCCGCGACAGCCTGCGCGCAACAAAGCGTCACCACTACCACGAGTTGCTGA

GGTGTAGGACAAATCACCACAAAGGAGCTTGGCACGGTCATGCGGTCCCTGGGTCAGAAC

CCGTCCGAGTCTGAGCTGCAAGATATGATTAACGAGGTCGACGCCGACAACAACGGCACC

ATTGACTTTGGTACGTCCAGATGCTCGCCCA?????????????????????????????

????????????????????????????????????????????????????????????

????????????????????????????????????????????????????????????

????????????????????????????????????????????????????????????

????????????????????????????????????????????????????????????

???????????????????????????NNNNNNNNNNNNNNNNNNNNNNNNNNNNNNNNN

NNNNNNNNNNNNCAGGCGCACCCAGAAACCCTTTGTGAACTCTTACTGTTGCCTCGGCAG

GCCGGGGGGCCCCTCGAGACGAGGAGCAGGCCCGCCGGCGGCCAAGCTAACTCTTGTCTT

TACACTGAAACTCTAAATGAATCAAAACTTTCAACAACGGATCTCTTGGTTCTGGCATCG

ATGAAGAACGCAGCGAAATGCGATAAGTAATGTGAATTGCAGAATTCAGTGAATCATCGA

ATCTTTGAACGCACATTGCGCCCTCTGGTATTCCGGAGGGCATGCCTGTTCGAGCGTCAT

TTCAACCCTCAAGCCTGGCTTGGTGATGGGGCGCTGGGGAGCAGGCCCTGAAATTCAGCG

GCGAGCTCGCCAGGACCCCGAGCGCAGTAGACCCTCGCTAAGGCCCTGGCGGTGCCCTGC

NNNNNNNNNNNNNNNNNNNNNNNNNNNCATCATGCTCGCACTGCTTCGTCAGCACAATTT

CACCCCTCGCTCTGGATTTTCAGGGTGCGGGGCTTAGAGCTTATCTCATCACCAACACCG

AACAGATGCTGATATCTACACAGCCGCCGAGCTTGGTNNNNNNNNNNNNNNNNNNNNNNN

NNNNNNNNNNNNNNNNNNNNNNNNNNNNNNNNNNNNNNNNNNNNNNNNNNNNNNNNNNNN

NNNNNNNNNNNNNNNNNNNNNNNNNNNNNNNNNNNNNNNNNNNNNNNNNNNNNNNNNNNN

NNNNNNNNNNNNNNNNNNNNNNNNNNNNNNNNNNNNNNNNNNNNNNNNNNNNNNNNNNNN

NNNAAGTCCACCACCGCGGGTCGACCTCGAGGCATACTGACCTCGTAGGCAAACCATCTC

TGGCGAGCACGGCCTCGACAGCAATGGCGTGTATGCACCTCCTATTCCTGCCCAT-----

-----CTGACAATTGCACAGTTACAACGGCACTTCTGAGCTCCAGCTCGAGCGCATGAAC

GTCTACTTCAACGAGGTAAGGACATCATTTCGACCGTGTCCAACGTTGCTAACGCGTTGT

CAGGCCTCCGGCAACAAGTATGTTCCTCGCGCCGTCCTCGTCGATCTCGAGCCGGGTACC

ATGGACGCCGTC

>Diaporthe_isoberliniae_CBS_137981

????????????????????????????????????????????????????????????

????????????????????????????????????????????????????????????

????????????????????????????????????????????????????????????

????????????????????????????????????????????????????????????

????????????????????????????????????????????????????????????

????????????????????????????????????????????????????????????

????????????????????????????????????????????????????????????

????????????????????????????????????????????????????????????

????????????????????????????????????????????????????????????

????????????????????????????????????????????????????????????

???????????????????????????NACAAGGTCTCTGTTGGTGAACCAGCGGAGGGA

TCATTGCTGCCCCTGGCGCACCCAGAAACCCTTTGTGAACTTTTACTGTTGCCTCGGCAG

GCCGTGGGTCCCTCTGAGACAAGGAGCA-GCCGGCCGGTGGCCAAGTTAACTC-TGTTTT

TACACTGAAACTCTAAATGAATCAAAACTTTCAACAACGGATCTCTTGGTTCTGGCATCG

ATGAAGAACGCAGCGAAATGCGATAAGTAATGTGAATTGCAGAATTCAGTGAATCATCGA

ATCTTTGAACGCACATTGCGCCCTCTGGTATTCCGGAGGGCATGCCTGTTCGAGCGTCAT

TTCAACCCTCAAGCCTGGCTTGGTGTTGGGGCACTGACAAGCAGGCCCTGAAATATAGTG

GCGAGCTCGCCAGGACTCCGAGCGTAGTAAGTTCTCGCTAAGG-CCTGGCGGTGCCCTGC

TGAAA???????????????????????????????????????????????????????

????????????????????????????????????????????????????????????

??????????????????????????????????????????TCGCACCTGAGCCCCACC

ATCGCGACCCTCCCCCCGGGACAGCCAGATAAGACGCGTCGATTGCTAACATGTTTTTCT

CGTCCACAGGTTCACCTTCAGACCGGCCAATGCGTAAGTTGCTGTCACACCGGA-CCTTA

TCATCGCCACCTGTAGCACGTTTCCCAGGGTAACCAAATCGGTGCTGCTTTCTGGTGCGT

CC-AAGTCCACCACCGCGACACGACCTTGGAGCATACTGACCTCGCAGGCAAACCATCTC

TGGCGAGCACGGTCTCGACAGCAATGGCGTGTATGTACCTCCTATTTCTGTCTTTCTCGT

-TGCCCTGACAATCATACAGTTACAACGGCACTTCCGAGCTCCAGCTCGAGCGCATGAAC

GTCTACTTCAACGAGGTAAGTATGTTG--TTGACCATCCACAGTCTTGCTAACGCGTTAT

CAGGCCTCCGGCAACAAGTATGTGCCCCGCGCCGTCCTCGTCGATCTCGAGCCCGGCACC

ATGGACGCCGTC

>Diaporthe_italiana_MFLUCC18_0090

CTTTGTAAGGTGTCCTT------CCCACCGTGGCTGTTGCGCATGCTAACGGACCGTTGT

CGGCCTCCAGGATAAGGATGGCGATGGTGAGTGCGGCTGCCTCTACCCTAAACGCGTCAC

GATCGATCCGCCGCGACAGCTTGCGCGCGATCAAGCGCCATCACTAGCAGGAGTTGCTAA

AGTGTAGGACAAATCACCACGAAGGAGCTCGGAACGGTCATGCGATCGCTGGGTCAGAAC

CCGTCCGAGTCTGAGCTGCAAGATATGATCAACGAGGTCGATGCCGACAACAATGGCACC

ATCGATTTTGGTACGTTCAGATGTCTGCTGA?????????????????????????????

????????????????????????????????????????????????????????????

????????????????????????????????????????????????????????????

????????????????????????????????????????????????????????????

????????????????????????????????????????????????????????????

???????????????????????????AACAAGGTCTCCGTTGGTGAACCAGCGGAGGGA

TCATTGTTGCTCCG-GCGCATCCAGAAACCCTTTGTGAACTTATACTGTTGCCTCGGCAG

GCTGGGGGGTCCCTCTCGGTGAGGAGCAGGCCCGCCGGCGGCCAAGTTAACTCTTGTTTT

TACACTGAAACTCTAAATGAATCAAAACTTTCAACAACGGATCTCTTGGTTCTGGCATCG

ATGAAGAACGCAGCGAAATGCGATAAGTAATGTGAATTGCAGAATTCAGTGAATCATCGA

ATCTTTGAACGCACATTGCGCCCTCTGGTATTCCGGAGGGCATGCCTGTTCGAGCGTCAT

TTCAACCCTCAAGCCTGGCTTGGTGATGGGACAGTGAAAGGCAGGTCCTGAAATTCAGTG

GCGAGCTCGCCAGGACCCCGAGCGCAGTAGACCCTCGCTAAGGTAATGGTGGGAGCCTGC

GAAAACCTTACCAAGCTCGGCGGCTCACCACACGCCTCTACGGCTGCGTCATCGCATTTT

CACCCCTCGTTCTGGATTTTCAGGGTGCGGGGCTTAGACCTTATCTCACAACCACCACCG

GAAACGTGCTGATACTTTACCAGCCGCCGAGCTTGGCAAACCNNNNNNNNNNNNNNNNNN

ATCGCGACCCACCCCCTGAGACACTCAGATAAGACGCGTCGAATGCTGACTTTTTTTCCT

CGTTCACAGGTTCACCTTCAGACCGGCCAATGCGTAAGTTGCTGTCACCACCGCACCTTA

T---CGCCCCCTGTAGCACGTTTTCCAGGGTAACCAAATCGGTGCTGCTTTCTGGTGCGT

C----CTCCATCATCGCGACAGGACCTCGCAACATATTGACTTCGTAGGCAAACCATCTC

TGGCGAGCACGGCCTCGACAGCAATGGCGTGTATGTACCTCCTATTCCTACTCGTCTCGT

CCGCCCTGACAGCTTCACAGTTACAACGGCTCTTCTGAGCTCCAGCTCGAGCGCATGAAC

GTCTACTTCAACGAGGTCAGTTTTTTATACCCACGATCTCCAACTTTGCTGACACCTTAT

CAGGCTTCCGGCAACAAGTATGTGCCTCGCGCTGTCCTCGTCGATCTCGAGCCCGGTACC

ATGGACGCCGTC

>Diaporthe_jinxiu_CGMCC_3_20269

NNNNNNNNNNNNNNNNNNNNNNNCCCGCCCTTGCTGTCGCGCATGCTAACGGACCGTTTT

CGGCCTGCAGGATAAGGATGGCGATGGTTAGTGCGGCCGCTCTCACCTAGCACGCGTCAT

GCTCGATCCGCCGCGACGGCCTGCGCGCAAGCAGGCGATATCACTTTCACGAGTTGCTGA

GGTGTAGGACAAATCACCACCAAGGAGCTCGGCACGGTCATGCGGTCCCTGGGCCAGAAC

CCGTCCGAGTCTGAGCTGCAAGACATGATCAACGAGGTCGACGCCGACAACAATGGCACC

ATCGACTTTGGTACGTCCGGATGCTCGCCCATCCGCGCCCTCCACCGGAGGTGTCAAGAA

GCCTCACCGCTACAAGCCTGGTACCGTCGCTCTGCGTGAGATCCGTCGCTACCAGAAGAG

CACCGAGCTGCTGATCCGCAAGCTCCCCTTCCAGCGTCTGGTAAGCAGGTCCGTGAGATC

GCCCAGGACTTCAAGTCCGACCTCCGCTTCCAGTCTTCGGCCATCGGTGCCCTGCAGGAG

TCGGTCGAGTCTTACCTCGTCTCCCTCTTCGAGGACACCAACCTGTGCGCCATCCACGCC

AAGCGCGTCACCATCCAGTCGGTATGTNNNNNNNNNCCCACTGGTGA--CAGCGGAGGGA

-CATTGCTGCCCCAGGCGCACCCAGAAACCCTTTGTGAACTT-TATTGTTGCCTCGGCTA

GCTGGGGGGCCCCTTGAGACAAGGAGCG-GCGCGCCGGCGGCCCATTTAACTCTGTTTCT

T--AGTGAATCTCTAAATGAATCAAAACTTTCAACAACGGATCTCTTGGTTCTGGCATCG

ATGAAGAACGCAGCGAAATGCGATAAGTAATGTGAATTGCAGAATTCAGTGAATCATCGA

ATCTTTGAACGCACATTGCGCCCTCTGGTATTCCGGAGGGCATGCCTGTTCGAGCGTCAT

TTCAACCCTCAAGCCTGGCTTGGTGTTGGGGCACTGAGGGGCAGGCCCTGAAATATAGTG

GCGAGCTCGCCAGGACTCCGAGCGCAGTAG-ATCTCGTTAAGG-CCTGGCGGTGCCCTGC

TGAAAGAGAAGGAAGGTTAGTAGACACCATCATGGTTGCGACTGTCCGTTGGCGCATTTT

CACCCCTCGCTCTGCAATTTCAGGGTGCGGGGCTTAGGGCTTATCTCACCACCATAACCA

CAATCATGCTAATACCCCCCAAGCCNNNNNNNNNNNNNNNNNNNNNNNNNNNNNNNNNNN

NNNNNNNNNNNNNNNNNNNNNNNNNNNNNNNNNNNNNNNNNNNNNNNNNNNNNNNNNNNN

NNNNNNNNNNNNNNNNNNNNNNNNNNNNNNNNNNNNNNNNNNNNNNNNNNNNNNNNNNNN

NNNNNNNNNNNNNNNNNNNNNNNNNNNNNNNNNNNNNNNNNNNNNNNNNNNNNNATCAAG

TCTGAGTAGTATGCTACAGTACGACCTCAAAGCATACTGACCTCGTAGGCAAACCATCTC

TGGCGAGCACGGCCTCGACAGCAATGGCGTGTATGTACCTCCTTTTCCTGCCCATCTCGT

CCTCCTTGACAGTCGCACAGTTACAACGGCACTTCCGAGCTCCAGCTCGAGCGCATGAAC

GTCTACTTCAACGAGGTATGTACGCCGTTCTGACCATCCACAGCCTTGCTAACGCGTTAT

CAGGCCTCCGGCAACAAGTATGTGCCTCGCGCCGTCCTCGTCGATCTCGAGCCCGGTACC

ATGGACGCCGTC

>Diaporthe_juglandicola_CFCC_51134

CTTTGTAAGTCATTCTCCACATCCCCGCCCCTACTGTTGCGCATGCTAACCGCCCTTTTT

CGGTCTGCAGGATAAGGATGGCGATGGTTAGTGTGGCCGCACCCACCGACCACGCGCCAT

GCTCGATCCGCCGCGACGGCCTGCGCGCGAGCAGGCGTCACCACTATCAGGGATTGCTAA

GGCGTAGGACAAATCACCACGAAGGAGCTCGGCACTGTCATGCGGTCCCTGGGCCAGAAC

CCGTCCGAGTCTGAGCTGCAAGATATGATTAACGAGGTCGATGCCGACAACAATGGCACC

ATTGACTTTGGTATGTTCAGACGCTCGCTCT?????????????????????????????

????????????????????????????????????????????????????????????

????????????????????????????????????????????????????????????

????????????????????????????????????????????????????????????

????????????????????????????????????????????????????????????

???????????????????????????NNNNNNNNNNNNNNNNNNNNNNNNNNNNNNNNN

NNNNNNNNNNNNNNNNNNNNNNNNNNNNCCCTTTGTGAACTCTTACTGTTGCCTCGGCAG

GCCGGGGGGCCCCTCCCGGTGAGGAGCAGGCCCGCCGGCGGCCAAGCCAACTCTTGTTTT

TACACTGAAACTCTAAATGAATCAAAACTTTCAACAACGGATCTCTTGGTTCTGGCATCG

ATGAAGAACGCAGCGAAATGCGATAAGTAATGTGAATTGCAGAATTCAGTGAATCATCGA

ATCTTTGAACGCACATTGCGCCCTCTGGTATTCCGGAGGGCATGCCTGTTCGAGCGTCAT

TTCAACCCTCAAGCCTGGCTTGGTGATGGGGCGCTGAGGAGCAGGCCCTGAAATCCAGTG

GCGAGCCCGCCGGGACCCCGAGCGCAGTAGACCCTCGCTAAGGCCCTGGCGGCGCCCTGC

TGAAANNNNNGGAAGGTTAGTAAACATCATCCTTCCTGCGCAGCTCCATCCGCGCATTTT

CACCCCTCGTTCTGGATTTTCAGGGTGCGGGGCTTAGAGCTTATCACACTTCTACC---G

GAAAGAGACTGACAACTATACAGCCGCCGAGCTTGGCAAGGGTCGCACCTGAGCCCCACC

ATCGCGACCCACCCCCTGGGACGCCCAGATAAGACGCGTCGATTGCTTACATGCTTTTCT

CTCCTGCAGGTTCACCTTCAGACCGGCCAATGCGTAAGTTGCTGTCACGCCGGACCCTTA

T---CGCCACCTGTAGCACGTTTCCCAGGGCAACCAAATCGGTGCTGCTTTTTGGTGCGT

CCCAAACTCAACACCGCGATACGACCTCGCGGCATACTGACCTCGTAGGCAAACCATCTC

TGGCGAGCACGGCCTCGACAGCAATGGCGTGTATGCACTTCCTATTCCTGCACATCTCGC

CCTCCCTGACAATTGCACAGTTACAACGGCACTTCCGAGCTCCAGCTCGAGCGCATGAGC

GTCTACTTCAACGAGGTATGTACGTTGTTTCGACCGTCTTCATCCTCGCTGACGCGTTAT

CAGGCCTCCGGCAACAAGTATGTTCCTCGTGCGGTCCTCGTCGATCTCGAGCCCGGTACC

ATGGACGCCGTC

>Diaporthe_kadsurae_CFCC_52586

NNNNNNNNNNNNNNNNNNNNNNNNNNCCCCTCACTGTCGCGCATGCTAACGGACCGTTCT

CGGCCTCCAGGATAAGGATGGCGATGGTTAGTGCAGCTGCTCCCAGCTTGTACGCGTCAC

GATCGACCCGCCGCGACGGCTTGCGCGCAACCAAGC-CCATAACTACTATGAGCTGCTAA

GGTGTAGGACAAATCACCACAAAGGAGCTCGGCACGGTCATGCGATCTCTGGGTCAGAAC

CCGTCCGAGTCTGAGCTGCAGGATATGATCAACGAGGTCGACGCCGACAACAATGGAACC

ATCGACTTTGGTACGTCCAGATGCTCGCTTGTCCGCGCCCTCCACCGGAGGTGTCAAGAA

GCCTCACCGCTACAAGCCTGGTACCGTCGCTCTGCGTGAGATTCGTCGCTACCAGAAGTC

CACTGAGCTTCTGATCCGCAAGCTGCCCTTCCAGCGTCTGGTACGCAGGTTCGTGAGATT

GCCCAGGACTTCAAGTCCGACCTCCGCTTCCAGTCCTCCGCCATCGGTGCCCTGCAGGAG

TCCGTCGAGTCCTACCTCGTCTCCCTCTTCGAGGACACCAACCTGTGCGCCATCCACGCC

AAGCGTGTCACCATCCAGTCGGTATGTNNNNNNNNNNNNNNNNNNNNNNNNNNNNNNNNN

NNNNNNNNNNNNNNNNNNNNNNNNNNNNCCCTTTGTGAACTTTTACTGTTGCCTCGGCAG

GCCGGGGGGCCCCTCCTGACGAGGAGCAGGCTCGCCGGCGGCCAAGTTAACTCTTGTTTT

TATTGTGAAACTCTAAATGAATCAAAACTTTCAACAACGGATCTCTTGGTTCTGGCATCG

ATGAAGAACGCAGCGAAATGCGATAAGTAATGTGAATTGCAGAATTCAGTGAATCATCGA

ATCTTTGAACGCACATTGCGCCCTCTGGTATTCCGGAGGGCATGCCTGTTCGAGCGTCAT

TTCAACCCTCAAGCCTGGCTTGGTGATGGGGCACTGCGAGGCAGGCCCTGAAATTCAGTG

GCGAGCTCGCCAGGACTCCGAGCGCAGTAGACCCTCGCTAAGG-ACTGGCGGTGCCCTGC

TGAAAGAGAAGGAAGGTTAGTAAACATCCTGGCGTTCCCACGGCGTCATCAGCGCATTTT

CACCCCTCGCTCTGGATTTTCAGGGTGCGGGGCTTAGAGCTTATCTCACCACCACTACCT

TGCACATGCTGACATCTTCACAGCCGCTGAGCTCGGTAAGGGTCGCACCTGAGCCCCACC

ATCGCGACCCACCCCCTGAAACACTTCCATAAGACGCGTCGATTGCTAACATGTTTTTCT

CGCCCACAGGTTCATCTCCAGACCGGCCAATGCGTAAGTTGCTGTCACCACCGCACCTTA

TCGCCG---CCTGTAGCACGTTTCCCAGGGTAACCAAATCGGTGCTGCTTTCTGGTGCGT

C----GTCCATCACCGCGACACGACCTCGCAACATACTGACCTCGTAGGCAAACCATCTC

TGGCGAGCACGGCCTCGACACCAATGGCGTGTATGCACCTCCTATTCCTGCCCATCTCGG

CTCGGCTGACAATTGCACAGCTACAACGGCACTTCCGAGCTCCAGCTCGAGCGCATGAAC

GTCTACTTCAACGAGGTAAGTATGTCATGTTAACGAGCCCCCGCTTTGCTGACCGCTTAT

CAGGCTTCCGGCAACAAGTATGTTCCTCGCGCCGTCCTCGTCGATCTCGAGCCCGGTACC

ATGGACGCCGNN

>Diaporthe_kochmanii_BRIP_54033

????????????????????????????????????????????????????????????

????????????????????????????????????????????????????????????

????????????????????????????????????????????????????????????

????????????????????????????????????????????????????????????

????????????????????????????????????????????????????????????

????????????????????????????????????????????????????????????

????????????????????????????????????????????????????????????

????????????????????????????????????????????????????????????

????????????????????????????????????????????????????????????

????????????????????????????????????????????????????????????

???????????????????????????NNNNNNNNNNNNNNNNNNNNNNNNNNNNAGGGA

TCATTGCTGCTTC-GGCGCACCCAGAAACCCTTTGTGAACTTA--TTGTTGCCTCGGTAG

GCCGGGAGGCCCCCTGAAACAGGGAGCA-GCCCGCCGGCGGCCAACCAAACTCTTGTTTC

TACAGTGAATCTCTAAATGAATCAAAACTTTCAACAACGGATCTCTTGGTTCTGGCATCG

ATGAAGAACGCAGCGAAATGCGATAAGTAATGTGAATTGCAGAATTCAGTGAATCATCGA

ATCTTTGAACGCACATTGCGCCCTCTGGTATTCCGGAGGGCATGCCTGTTCGAGCGTCAT

TTCAACCCTCAAGCCTGGCTTGGTGATGGGGCACTGGAAAGCAGGCCCTGAAATCTAGTG

GCGAGCTCGCCAGGACCCCGAGCGTAGTAGA-TCTCGCTAAGGCCCTGGCGGTGCCCTGC

TGAAANNGAAGGAAGGTTAGTAAACATCGTTGTGCCTGCGAGGCTTCGTCAAGGCATTTT

CACCCCTCCCTCTGGATTTTCAGGGTGCGGGGCTTACCGCTTATCTCAGTGAC-GCAC-G

AAAAGATGCTGATTTCTGAACAGCCGCCGAGCTGGGCAAGGG??????????????????

????????????????????????????????????????????????????????????

????????????????????????????????????????????????????????????

????????????????????????????????????????????????????????????

????????????????????????????????????????????????????????????

????????????????????????????????????????????????????????????

????????????????????????????????????????????????????????????

????????????????????????????????????????????????????????????

????????????????????????????????????????????????????????????

????????????

>Diaporthe_kongii_BRIP_54031

????????????????????????????????????????????????????????????

????????????????????????????????????????????????????????????

????????????????????????????????????????????????????????????

????????????????????????????????????????????????????????????

????????????????????????????????????????????????????????????

????????????????????????????????????????????????????????????

????????????????????????????????????????????????????????????

????????????????????????????????????????????????????????????

????????????????????????????????????????????????????????????

????????????????????????????????????????????????????????????

???????????????????????????NNNNNNNNNNNNNNNNNNNNNNNNNNNNAGGGA

TCATTGCTGCTTC-GGCGCACCCAGAAACCCTTTGTGAACTTA--TTGTTGCCTCGGTAG

GCCGGAAAGCCCCCTGAAACAGGGAGAA-GCCCGCCGGCGGCCAACCAAACTCTTGTTTC

TACAGTGAATCTCTAAATGAATCAAAACTTTCAACAACGGATCTCTTGGTTCTGGCATCG

ATGAAGAACGCAGCGAAATGCGATAAGTAATGTGAATTGCAGAATTCAGTGAATCATCGA

ATCTTTGAACGCACATTGCGCCCTCTGGTATTCCGGAGGGCATGCCTGTTCGAGCGTCAT

TTCAACCCTCAAGCCTGGCTTGGTGATGGGGCACTGAAGGGCAGGCCCTGAAATCTAGTG

GCGAGCTCGCCAGGACCCCGAGCGTAGTAGA-TCTCGCTAAGGCCCTGGCGGTGCCCTGC

TGAAANNNAAGGAAGGTTAGTAAACATCGTTGTGTCTGCGAGGCTTCGTCAAGGCATTTT

CACCCCTCCCTCTGGATTTTCAGGGTGCGGGGCTTACCGCTTATCTCAGTGAC-GCAC-G

AAAAGATGCTGATTTCTGAACAGCCGCCGAGCTGGGCAAGGGTCGCACCTGAGCCCCACC

ATCGCGACCCACCCCCTGCGACACCCAGATAACACGCGTCGATTGCTAACATGTTTTTCT

CGCCCACAGGTTCACCTTCAGACCGGCCAATGCGTAAGTTGCTGTCACCGCCAGACCTTA

T---CGCCACCCGTAGCACGTTTCCCAGGGTAACCAAATCGGTGCTGCTTTCTGGTGCGT

CTCAAGCCTACCACCGCGAGGCGAGCTCGAAGCATACTGACCTCGTAGGCAAACCATCTC

TGGCGAGCACGGTCTCGACAGCAATGGCGTGTATGCACCTCCTATTCCTGCCCACCTGGT

CCTCCCTGATGATCGCACAGTTACAACGGCACTTCCGAGCTCCAGCTCGAGCGCATGAAC

GTCTACTTCAACGAGGTATGTACGTCGTTTTGA-ACCTCTCGCCCTTGCTAACGCGTTTT

CAGGCTTCCGGCAACAAGTATGTGCCCCGCGCCGTCCTCGTCGATCTCGAGCCCGGTACC

ATGGACGCCGTC

>Diaporthe_krabiensis_MFLUCC_17_2481

????????????????????????????????????????????????????????????

????????????????????????????????????????????????????????????

????????????????????????????????????????????????????????????

????????????????????????????????????????????????????????????

????????????????????????????????????????????????????????????

????????????????????????????????????????????????????????????

????????????????????????????????????????????????????????????

????????????????????????????????????????????????????????????

????????????????????????????????????????????????????????????

????????????????????????????????????????????????????????????

???????????????????????????NNNNNNNNNNCCGTTGGTGAACCAGCGGAGGGA

TCATTGCTGCCCCAGGCGCACCCAGAAACCCTTTGTGAACTTTTACTGTTGCCTCGGCAT

GCTGGGGGGCCCCCTGAGACAGGGAGCAGGCACGCCGGCGGCCAAGTTAACTCTTGTTTT

TACACTGAAACTCTAAATGAATCAAAACTTTCAACAACGGATCTCTTGGTTCTGGCATCG

ATGAAGAACGCAGCGAAATGCGATAAGTAATGTGAATTGCAGAATTCAGTGAATCATCGA

ATCTTTGAACGCACATTGCGCCCTCTGGTATTCCGGAGGGCATGCCTGTTCGAGCGTCAT

TTCAACCCTCAAGCATTGCTTGGTGTTGGGGCACTGCGAAGCAGGCCCTGAAATCTAGTG

GCGAGCTCGCCAGGACCCCGAGCGCAGTAGACCCTCGCTAAGGCCCTGGCGGTGCCCTGC

TGAAANNNNNNAACCGACAATAAAGGGAATAATGTTGGTACGGCTTCGTCAGCGCATTTT

CACCCCTCGCTCTGGATTTTCAGGGTGCGGGGCTTAGAGCTTATCTAGTTACTACCACAA

GAAACATGCTGATATCTACATAGCCGCCGAGCTTGGTAAGGGNNNNNNNNNNNNNNNNNN

NNNNNNNNNNNNNNNNNNNNNNNNNNNNNNNNNNNNNNNNNNNNNNNNNNNNNNNNNNNN

NNNNNNNNNNNNNNNNNNNNNNNNNNNNNNNNNNNNNNNNNNNNNNNNNNNNNNNNNNNN

NNNNNNNNNNNNNNNNNNNNNNNNNNNNNNNNNNNNNNNNNNNNNNNNNNNNNNNNNNNN

NNNNNTGCCGTACACGCGATATGACCTCGAA-CATACTGACATCGCAGGCAAACCATCTC

TGGCGAGCACGGCCTCGACAGCAATGGCGTGTATGCACCTCCTATTCCTGCCTCTCTCGT

CTGCCCTGACAATCACACAGGTACAACGGCACTTCCGAGCTCCAGCTCGAGCGCATGAAC

GTCTACTTCAACGAGGTAAGTACGTCG--TTGACCATCTGCAGCTTTGCTAACGCGTTAT

CAGGCCTCCGGCAACAAGTATGTCCCTCGCGCCGTCCTCGTCGATCTCGAGCCCGGTACC

ATGGACGCCGTC

>Diaporthe_lenispora_CGMCC_3_20101

NNNNNNNNNNNNNNNNNNNNNNNCCCGCCCTTG--ATTGCGCATGCTAACGGACCGTTTC

CGGCTTGTAGGATAAGGATGGCGATGGTTAGTGCGACAGCTCTCACCTATCACGCGTCAC

GCTCGATCCGCCGCGACGGCCTGCGCGCAACTCAGCGTTATCATTATTACGAGTTGCTGA

GGTGTAGGACAAATCACCACCAAGGAGCTCGGCACGGTCATGCGATCCCTGGGTCAGAAC

CCGTCCGAGTCTGAGCTGCAAGATATGATTAACGAGGTCGACGCCGACAACAATGGCACC

ATTGACTTTGGTACGTCCAGATGCTTGCCCATCCGCGCCCTCCACCGGAGGTGTCAAGAA

GCCTCACCGCTACAAGCCTGGTACCGTCGCTCTGCGTGAGATCCGTCGCTACCAGAAGAG

CACCGAGCTGCTGATCCGCAAGCTCCCCTTCCAGCGTCTGGTAAGCAGGTTCGTGAGATC

GCCCAGGACTTCAAGTCCGACCTGCGCTTCCAGTCTTCCGCCATCGGTGCCCTGCAGGAG

TCCGTCGAGTCTTACCTCGTCTCCCTGTTCGAGGACACCAACCTGTGCGCCATCCACGCC

AAGCGTGTCACCATCCAGTCGGTACGTATCACAGTGTCTGTTGGGGAACCACAGGAGGGG

TCTCTGTGGCCCCCCGGGCGCCCCCAAAAACCTTGTGAAAATCTTTAGTTGCCTCTGCAT

TCCGGGGGGTCCCTTGAGACGAGGAGCAGGCACGCCGGCGGCCAAGTTAACTCTTGTTTT

TACACTGAAACTCTAAATGAATCAAAACTTTCAACAACGGATCTCTTGGTTCTGGCATCG

ATGAAGAACGCAGCGAAATGCGATAAGTAATGTGAATTGCAGAATTCAGTGAATCATCGA

ATCTTTGAACGCACATTGCGCCCTCTGGTATTCCGGAGGGCATGCCTGTTCGAGCGTCAT

TTCAACCCTCAAGCCTGGCTTGGTGATGGGGCACTGCAGGGCACGCCCTCAAATCTAGTG

GCGAGCTCGCCAGGACCCCGAGCGCAGTAGACCCTCGCTAAGGCCCTGGCGGTGCCCTGC

NNNNNNNNNNNNNNNNNNNNNNNNNNNCATCATGCTGGTGCGGCTTCGTCAGCACATTTT

CACCCCTCGCTCTGGACTTTCAGGGTGCGGGGCTTAGAGCTTATCTCACCACCACCACCG

AAGAGATGCTGACATCTACACAGCCGCCGAGCTTGGTNNNNNNNNNNNNNNNNNNNNNNN

NNNNNNNNNNNNNNNNNNNNNNNNNNNNNNNNNNNNNNNNNNNNNNNNNNNNNNNNNNNN

NNNNNNNNNNNNNNNNNNNNNNNNNNNNNNNNNNNNNNNNNNNNNNNNNNNNNNNNNNNN

NNNNNNNNNNNNNNNNNNNNNNNNNNNNNNNNNNNNNNNNNNNNNNNNNNNNNNNNNNNN

NNNNAGCCTACCACCGCGGTACGACCTCGAAGCATACTGACCTCGTAGGCAAACCATCTC

TGGCGAGCACGGCCTCGACAGCAATGGCGTGTATGCACCTCCTATTTCTGCCTCTCTCGT

CTGCCCTGACAATCACACAGTTACAACGGCTCTTCCGAGCTCCAGCTCGAGCGCATGAAC

GTCTACTTCAACGAGGTAAGTACGTCG--TTGACCATATACGGCTTTGCTAACGCGTTAT

CAGGCCTCCGGCAACAAGTATGTTCCCCGCGCCGTCCTCGTCGATCTCGAGCCCGGTACC

ATGGACGCCGTC

>Diaporthe_leucospermi_CBS_111980

CTTTGTAAGTTATCTTCGCCTTGCCCGCCATTGCTTTTGCGCATGCTAACGGACCGTTTT

CGGCCTGCAGGATAAGGATGGCGATGGTTAGTGTGGTCACTCTCAGCTAGCACGCGTCAT

ACTCGATCCGCCGCGACGGTCTGCGCGCGAGCGACCGTCATCACTATCACGAGTTGCTAA

GGTGTAGGACAAATCACCACCAAGGAGCTCGGCACAGTCATGCGGTCCCTTGGTCAAAAC

CCTTCCGAGTCCGAGCTGCAGGACATGATCAACGAGGTCGACGCCGACAACAATGGCACC

ATTGACTTTGGTAAGTCTGGATGCTCATCCCTCCGCGCCCTCCACCGGAGGTGTCAAGAA

GCCTCACCGCTACAAGCCTGGTACCGTCGCTCTGCGTGAGATCCGTCGCTACCAGAAGAG

CACCGAGCTGCTGATCCGCAAGCTGCCCTTCCAGCGTCTGGTATGTAGGTCCGTGAGATC

GCCCAGGACTTCAAGTCCGACCTGCGCTTCCAGTCTTCCGCCATCGGTGCCCTTCAGGAG

TCCGTCGAGTCTTACCTCGTCTCCCTCTTTGAGGACACCAACCTGTGCGCCATCCACGCC

AAGCGTGTCACCATCCAGTCGGTACGTAACAAGGTCTCCGTTGGTGAACCAGCGGAGGGA

TCATTGCTGCTTC-GGCGCACCCAGAAACCCTTTGTGAACTTA--CTGTTGCCTCGGTAA

GCTGGTAAGCCCCCTGAAACGGGGAGCA-GCCCGCCGGCGGCCAACTAAACTCTTGTTTC

TATAGTGAATCTCTAAATGAATCAAAACTTTCAACAACGGATCTCTTGGTTCTGGCATCG

ATGAAGAACGCAGCGAAATGCGATAAGTAATGTGAATTGCAGAATTCAGTGAATCATCGA

ATCTTTGAACGCACATTGCGCCCCCTGGTATTCCGGGGGGCATGCCTGTTCGAGCGTCAT

TTCAACCCTCAAGCCTGGCTTGGTGATGGGGCACTGAGGAGCAGGCCCTGAAATTCAGTG

GCGAGCTCGCCAGGACCCCGAGCGTAGTAGA-TCTCGCTAAGGCCCTGGCGGTGCCCTGC

TGAAANNNNNNNAAGGTCAGTAAATATCATGGTGTTTGCGCGGCTGCGTCGAGCCATTTT

CACCCCTCCCTCTGGATTTTCAGGGTGCGGGGCTTACCGCTTATCTCACTGCCAGCACCG

ACAACATGCTGATTCCCACACAGCCGCCGAGCTGNNNNNNNNNNNNNNNNNNNNNNNNNN

NNNNNNNNNNNNNNNNNNNNNNNNNNNNNNNNNNNNNNNNNNNNNNNNNNNNNNNNNNNN

NNNNNNNNNNNNNNNNNNNNNNNNNNNNNNNNNNNNNNNNNNNNNNNNNNNNNNNNNNNN

NNNNNNNNNNNNNNNNNNNNNNNNNNNNNNNNNNNNNNNNNNNNNNNNNNNNTGGTGCGT

ACCGAGTCTGCCACCGCGAGACCACCTCCAAGCATACTGACCTTGTAGGCAAACCATCTC

TGGCGAGCACGGTCTCGACAGCAATGGCGTGTATGTACCTCCTATTCCTGCCTATCTCGT

CCTCCCTGATGATCGCACAGTTACAACGGCACTTCCGAGCTCCAGCTCGAGCGCATGAAC

GTCTACTTCAACGAGGTAAGCACGTCTTTTTGA-CAGTCTCGGCCTTGCTAATGCGCTCT

TAGGCCTCCGGCAACAAGTATGTGCCTCGCGCCGTCCTCGTCGATCTCGAGCCCGGTACC

ATGGACGCCGTC

>Diaporthe_limonicola_CBS_142549

NNNNNNNNNNNNNNNNNNNNNNNNNNNNNNNNNNNNNNNNNNNNNCTAACGGACCGTTTT

CGGCTTGTAGGATAAGGATGGCGATGGTTAGTGCGGCCGCTCTCACACAGCACGCGTCAT

GCTCGATCCCCCGCGACGGCCTGCGCGCAACCAAGCGTTATCACTATTGCGAGTTGCTGA

GGTGCAGGACAAATCACCACCAAGGAGCTCGGCACGGTCATGCGATCCCTGGGCCAGAAC

CCTTCCGAGTCTGAGCTGCAAGATATGATCAACGAGGTCGACGCCGACAACAACGGCACC

ATTGACTTTGGTACGTCCGGATATACGCCCATCCGCGCCCTCCACCGGAGGTGTCAAGAA

GCCTCACCGCTACAAGCCTGGTACCGTCGCTCTGCGTGAGATCCGTCGCTACCAGAAGAG

CACTGAGCTGCTGATCCGCAAGCTCCCCTTCCAGCGTCTGGTATGCAGGTCCGTGAGATC

GCCCAGGACTTCAAGTCCGATCTCCGCTTCCAGTCTTCCGCCATCGGTGCCCTGCAGGAG

TCCGTCGAGTCTTACCTCGTCTCCCTCTTCGAGGACACCAACCTGTGCGCCATCCACGCC

AAGCGTGTCACCATCCAGTCGGTACGTNNNNNNNNNNNNNNNNNNNNNNNNNNNNNNNNN

TCATTGCTGCCCCAGGCGCACCCAGAAACCCTTTGTGAACTTTTACTGTTGCCTCGGCAT

GCTGGTAGGCCCCTC-CGGTGAGGAGACGGCACGCCGGCGGCCAAGTTAACTCTTGTTTT

TACACTGAAACTCTAAATGAATCAAAACTTTCAACAACGGATCTCTTGGTTCTGGCATCG

ATGAAGAACGCAGCGAAATGCGATAAGTAATGTGAATTGCAGAATTCAGTGAATCATCGA

ATCTTTGAACGCACATTGCGCCCTCTGGTATTCCGGAGGGCATGCCTGTTCGAGCGTCAT

TTCAACCCTCAAGCACTGCTTGGTGTTGGGGCACTGCGAAGCAGGCCCTGAAATCTAGTG

GCGAGCTCGCCAGGACCCCGAGCGCAGTAGACCCTCGCTAAGGCCCTGGCGGTGCCCTGC

TGAAAGAGAAGGAAGGTTAGTAAACATCATCATGTTGGTACAGCTTCGTCAGCGCATTTT

CACCCCTCGCTCTGGATTTTCAGGGTGCGGGGCTTAGAGCTTATCTAGTTACTACCACCG

GAAACATGCTGATATCTACATAGCCGCCGAGCTTGGTAAGGGNNNNNGTAACACCCCACC

ATCGCGACCCACCCCCTTGGACACCCAGATAAAACGCGTCGATTGCTAACGTGTTTTTCT

TGACTACAGGTTCACCTTCAGACCGGCCAATGCGTAAGTTGCTGTCACGCCGGA-CCTTA

TCATCGCCACCCGTAGCACGTTTCCCAGGGTAACCAAATCGGTGCTGCTTTCTGGTGC--

-----GTCCACCGCCGCGATACGACCTCGAACCATACTGACCTCGCAGGCAAACCATCTC

TGGCGAGCACGGCCTCGACAGCAATGGCGTGTATGCACCTCCTATTCCTACCTTTCTCGT

CTGCCCTGACAATCACACAGTTACAACGGCACTTCCGAGCTCCAGCTCGAGCGCATGAAC

GTCTACTTCAACGAGGTAAGTACGTCG--TTGGCCATCTGCAGCTTTGCTAACGCGTTAT

CAGGCCTCCGGCAACAAGTATGTCCCTCGCGCCGTCCTCGTCGATCTCGAGCCCGGTANN

NNNNNNNNNNNN

>Diaporthe_litchicola_BRIP_54900

????????????????????????????????????????????????????????????

????????????????????????????????????????????????????????????

????????????????????????????????????????????????????????????

????????????????????????????????????????????????????????????

????????????????????????????????????????????????????????????

????????????????????????????????????????????????????????????

????????????????????????????????????????????????????????????

????????????????????????????????????????????????????????????

????????????????????????????????????????????????????????????

????????????????????????????????????????????????????????????

???????????????????????????AACAAGGTCTCCGTTGGTGAACCAGCGGAGGGA

TCATTGCTGCCCCAGGCGCACCCAGAAACCCTTTGTGAACTTTT-TTGTTGCCTCGGCAT

GCTGGTAGGCCCCTC-CGGTGAGGAGACGGCACGCCGGCGGCCAAAACAACTC-TGTTTT

TACACTGAAACTCTAAATGAATCAAAACTTTCAACAACGGATCTCTTGGTTCTGGCATCG

ATGAAGAACGCAGCGAAATGCGATAAGTAATGTGAATTGCAGAATTCAGTGAATCATCGA

ATCTTTGAACGCACATTGCGCCCTCTGGTATTCCGGAGGGCATGCCTGTTCGAGCGTCAT

TTCAACCCTCAAGCATTGCTTGGTGTTGGGGCACTGCGAAGCAGGCCCTGAAATCTAGTG

GCGAGCTCGCCAGGACCCCGAGCGCAGTAGACCCTCGCTAAGGCCCTGGCGGTGCCCTGC

TGAAANNNNNGGAAGGTTAGTAAACATCATCATGTTGGTACGGCTTCGTCAGCGCATTTT

CACCCCTCGCTCTGGATTTTCAGGGTGCGGGGCTTAGAGCTTATCTCGCTATTACCACCC

GAAACATGCTGATATCTACACAGCCGCCGAGCTTGGCAAGGGTCGCACCTGAGCCCCACC

ATCGCGAACCACCCCCTGGGACACCCAGATAAAACGCGTCGATTGCTAACGTGTTTTTCT

CGACTACAGGTTCACCTTCAGACCGGCCAATGCGTAAGTTGCTGTCACGCCGGA-CCTTA

TCATCGCCACCCGTAGCACGTTTCCCAGGGTAACCAAATCGGTGCTGCTTTCTGGTGC--

-----GTCCACCACCGCGATATGACCTCGAAACATACTGACCTCGCAGGCAAACCATCTC

TGGCGAGCACGGCCTCGACAGCAATGGCGTGTATGCACCTCCTATTCCTACCTTTCTCGT

CTGCCCTGACAATCATACAGTTACAACGGCACTTCCGAGCTCCAGCTCGAGCGCATGAAC

GTCTACTTCAACGAGGTAAGTACGTCG--TTCACCATCTGCAGCCTTGCTAACGCGTTAT

CAGGCCTCCGGCAACAAGTATGTCCCTCGCGCCGTCCTCGTCGATCTCGAGCCCGGTACC

ATGGACGCCGTC

>Diaporthe_litchii_SAUCC194_22

NNNNNNNACTCTCTAGCGACATGCCCGCCCTTGCTGGTGCGCATGCTAACGGACCGTTTT

CGGCTTGCAGGATAAGGATGGCGATGGTTAGTGCGGCCGCTCTCACCTAGCACGCGTCAT

GTTCGATCCACCGCGACGGTCTGCGCGCAGCCAAGCG-TATCACTATCACGAGTTGCTGA

GGTGTAGGACAAATCACTACCAAGGAGCTCGGCACGGTCATGCGATCCCTGGGTCAGAAC

CCGTCTGAGTCTGAGCTGCAAGATATGATTAACGAGGTCGACGCCGACAACAATGGCACC

ATCGACTTTGGTACGTCCAGATGCTCGCGTTTCCGCGCCCTCCACCGGAGGTGTCAAGAA

GCCTCACCGCTACAAGCCTGGTACCGTCGCTCTGCGTGAGATCCGTCGCTACCAGAAGAG

CACCGAGCTGCTGATCCGCAAGCTCCCTTTCCAGCGTCTGGTATGCAGGTCCGTGAGATC

GCCCAGGACTTCAAGTCCGACCTCCGCTTCCAGTCTTCCGCCATCGGTGCCCTGCAGGAG

TCGGTCGAGTCTTACCTCGTCTCCCTCTTCGAGGACACCAACCTGTGCGCCATCCACGCC

AAGCGTGTCACCATCCAGTCGGTATGTNNNNNNNTCTCCGTTGGTGAACCAGCGGAGGGA

TCATTGCTGCCCCAGGCGCACCCAGAAACCCTTTGTGAACTTTTACTGTTGCCTCGGCTA

GCTGGGGGGCCCCTCTTCTGGAGGAGCAGGCACGCCGGCGGCCAACCTAACTCTTGTTTT

TACACTGAAACTCTAAATGAATCAAAACTTTCAACAACGGATCTCTTGGTTCTGGCATCG

ATGAAGAACGCAGCGAAATGCGATAAGTAATGTGAATTGCAGAATTCAGTGAATCATCGA

ATCTTTGAACGCACATTGCGCCCTCTGGTATTCCGGAGGGCATGCCTGTTCGAGCGTCAT

TTCAACCCTCAAGCCTGGCTCGGTGATGGGGCACTGAAGGGCAGGCCCTGAAATTCAGTG

GCGAGCTCGCCAGGACCCCGAGCGTAGTAGACCCTCGCTAAGGCCCTGGCGGTGCCCC-C

GAAAANNNNNNNNNNNNNNNNNNNNNNCATCATGCTCGCGCGGCTTCGTCACCGCATTTT

CACCCCTCGCTTTGGATTTTCAGGGTGCGGGGCTTAGGGCTTATCTCACCACTACTACTG

AATATATGCTGATATCTACACAGCCGCCGAGCNNNNNNNNNNNNNNNNNNNNNNNNNNNN

NNNNNNNNNNNNNNNNNNNNNNNNNNNNNNNNNNNNNNNNNNNNNNNNNNNNNNNNNNNN

NNNNNNNNNNNNNNNNNNNNNNNNNNNNNNNNNNNNNNNNNNNNNNNNNNNNNNNNNNNN

NNNNNNNNNNNNNNNNNNNNNNNNNNNNNNNNNNNNNNNNNNNNNNNNNNNNNNNNNNNN

NCTCGAGCCACCACTGCGAGTCGACCTCGAAGCATACTGATCTCGTAGGCAAACCATCTC

TGGCGAGCACGGTCTCGACAGCAATGGCGTGTATGCACCTCCTATTCCTGCCCATCTTGG

CTCCCCTGACAATTGCACAGTTACAACGGCACTTCTGAGCTCCAGCTCGAGCGCATGAAC

GTCTACTTCAACGAGGTAAGTACATCATTCCGACCGTCCTCAACCTTGCTAACGCGTTAT

CAGGCCTCCGGCAACAAGTATGTTCCTCGCGCCGTCCTCGTCGATCTCGAGCCCGGTACC

ATGGACGCCGTC

>Diaporthe_lithocarpus_CGMCC_3_15175

????????????????????????????????????????????????????????????

????????????????????????????????????????????????????????????

????????????????????????????????????????????????????????????

????????????????????????????????????????????????????????????

????????????????????????????????????????????????????????????

????????????????????????????????????????????????????????????

????????????????????????????????????????????????????????????

????????????????????????????????????????????????????????????

????????????????????????????????????????????????????????????

????????????????????????????????????????????????????????????

???????????????????????????NNNNNNNNNNNNNNNNNNNNNNNNNNNNAGGGA

TCATTGCTGCCCCAGGCGCACCCAGAAACCCTTTGTGAACTTTTACTGTTGCCTCGGCAT

GCTGGGGGGCCCCTCGAGACGAGGAGCAGGCACGCCGGCGGCCAAGTTAACTCTTGTTTT

TACACTGAAACTCTAAATGAATCAAAACTTTCAACAACGGATCTCTTGGTTCTGGCATCG

ATGAAGAACGCAGCGAAATGCGATAAGTAATGTGAATTGCAGAATTCAGTGAATCATCGA

ATCTTTGAACGCACATTGCGCCCTCTGGTATTCCGGAGGGCATGCCTGTTCGAGCGTCAT

TTCAACCCTCAAGCATTGCTTGGTGTTGGGGCACTGAAGGGCAGGCCCTGAAATCTAGTG

GCGAGCTCGCCAGGACCCCGAGCGCAGTAGACCCTCGCTAAGGCCCTGGCGGTGCCCTGC

TGAAAGAGAAGGAAGGTTAGTAAACATCATCATGTTGGTACGGCTTCGTCAGCGCATTTT

CACCCCTCGCTCTGGA-TTTCAGGGTGCGGGGCTTAGAGCTTATCT-----ATGCCGTTC

GAAACATGCTGATATCTACACAGCCGCCGAGCTTGGTAAGGGNNNNNNNNNNNNNNNNNN

NNNNNNNNNNNNNNNNNNNNNNNNNNNNNNNNNNNNNNNNNNNNNNNNNNNNNNNNNNNN

NNNNNNNNNNNNNNNNNNNNNNNNNNNNNNNNNNNNNNNNNNNNNNNNNNNNNNNNNNNN

NNNNNNNNNNNNNNNNNNNNNNNNNNNNNNNNNNNNNNNNNNTGCTGCTTTCTGGTGCGT

CA-AAGTCCACCACCGCGATACGACCTCGAACCATACTGACCTCGCAGGCAAACCATCTC

TGGCGAGCACGGCCTCGACAGCAATGGCGTGTATGCACCTCCTATTCCTACTCTTCTTGT

CTGCCCTGACAATCACACAGTTACAACGGCTCTTCCGAGCTCCAGCTCGAGCGCATGAAC

GTCTACTTCAACGAGGTAAGTACGTCG--TTGACCATCTGCAGCCTTGCTAACGCGCCAT

CAGGCCTCCGGCAACAAGTATGTCCCTCGCGCCGTCCTCGTCGATCTCGAGCCCGGTACC

ATGGACGCCGTC

>Diaporthe_litoricola_MFLUCC_16_1195

????????????????????????????????????????????????????????????

????????????????????????????????????????????????????????????

????????????????????????????????????????????????????????????

????????????????????????????????????????????????????????????

????????????????????????????????????????????????????????????

????????????????????????????????????????????????????????????

????????????????????????????????????????????????????????????

????????????????????????????????????????????????????????????

????????????????????????????????????????????????????????????

????????????????????????????????????????????????????????????

???????????????????????????NNNNNNNTCTCCGTTGGTGAACCAGCGGAGGGA

TCATTGCTGCCCCGGGCGCACCCAGGAACCCTTTGTGAACTCATACTGTTGCCTCGGCAG

GCCGGGGGGCCCCTCGAGACGAGGAGCAGGCCCGCCGGCGGCCAAGCCAA-CCCTGTTTT

TAAACCGAAACTCTAAATGAATCAAAACTTTCAACAACGGATCTCTTGGTTCTGGCATCG

ATGAAGAACGCAGCGAAATGCGATAAGTAATGTGAATTGCAGAATTCAGTGAATCATCGA

ATCTTTGAACGCACATTGCGCCCTCTGGTATTCCGGAGGGCATGCCTGTTCGAGCGTCAT

TTCAACCCTCAAGCCCGGCTTGGTGCTGGGGCGCTGCAGGGCAGGCCCTGAAATACAGTG

GCGAGCCCGCCAGGACCCCGAGCGCAGTAGACCCTCGCTGAGG-CCTGGCGGTGCCCTGC

TGAAA???????????????????????????????????????????????????????

????????????????????????????????????????????????????????????

????????????????????????????????????????????????????????????

????????????????????????????????????????????????????????????

????????????????????????????????????????????????????????????

????????????????????????????????????????????????????????????

????????????????????????????????????????????????????????????

????????????????????????????????????????????????????????????

????????????????????????????????????????????????????????????

????????????????????????????????????????????????????????????

????????????????????????????????????????????????????????????

????????????

>Diaporthe_eres_CGMCC_3_17089

????????????????????????????????????????????????????????????

????????????????????????????????????????????????????????????

????????????????????????????????????????????????????????????

????????????????????????????????????????????????????????????

????????????????????????????????????????????????????????????

????????????????????????????????????????????????????????????

????????????????????????????????????????????????????????????

????????????????????????????????????????????????????????????

????????????????????????????????????????????????????????????

????????????????????????????????????????????????????????????

???????????????????????????NNNNNNNNNNNNNNNNNNNNNNNNNNNNNNNNN

NNNNNNNNNNNNNNNNNNNNNNNNNNNACCCTTTGTGAACTTTTACTGTTGCCTCGGCTA

GCTGGGGGGCCCCTCCGGGTGTTGAGACAGCCCGCCGGCGGCCAACCCAACTCTTGTTTT

TACACTGAAACTCTAAATGAATCAAAACTTTCAACAACGGATCTCTTGGTTCTGGCATCG

ATGAAGAACGCAGCGAAATGCGATAAGTAATGTGAATTGCAGAATTCAGTGAATCATCGA

ATCTTTGAACGCACATTGCGCCCTCTGGTATTCCGGAGGGCATGCCTGTTCGAGCGTCAT

TTCAACCCTCAAGCCTGGCTTGGTGATGGGGCACTGAAGGGCAGGCCCTGAAATTCAGTG

GCGAGCTCGCCAGGACCCCGAGCGCAGTAGACCCTCGCTAAGGCCCTGGCGGTGCCCTGC

TGAAANNNNNNNNNNNNNNGTAAATATCATCATGCTCGCGCGGCCTCGCCAGCGCATTTT

CACCCCTCGCTTTGGATTTTCAGAGTGCGGGGCTTAGGGCTTATCTTGTCACCACCACCG

AATATATGCTGATATCTACACAGCCGCCGAGCTTGGTAAGGGNNNNNNNNNNNNNNNNNN

NNNNNNNNNNNNNNNNNNNNNNNNNNNNNNNNNNNNNNNNNNNNNNNNNNNNNNNNNNNN

NNNNNNNNNNNNNNNNNNNNNNNNNNNNNNNNNNNNNNNNNNNNNNNNNNNNNNNNNNNN

NNNNNNNNNNNNNNNNNNNNNNNNNNNNNNNNNNNNNNNNGGTGCTGCTTTCTGGTGCGT

TCCAAGTCCACCGCCGCGATACGACCTCGAAGCATGCTGACCTCGTAGGCAAACCATCTC

TGGCGAGCACGGCCTCGACAGCAATGGCGTGTATGCACCTCCTATTCCTGCCCATCTTGG

CTTTCCTGACAATCGCACAGTTACAACGGCACTTCTGAGCTCCAGCTCGAGCGCATGAAC

GTCTACTTCAACGAGGTAAGTACAACATTCCGACCATCTCCAAGTTCGCTAACGCGTTAT

CAGGCCTCCGGCAACAAGTATGTTCCTCGCGCCGTCCTCGTCGATCTCGAGCCCGGTACC

ATGGACGCCGTC

>Diaporthe_longicolla_FAU_599

CTTTGTAAGTTATATCCGCCTCGCCCGCCGTTGCTGTTGCGCATGCTAACGGACCGTTTT

CGGCCTGCAGGATAAGGATGGCGATGGTTAGTGCGGTCACTCTCAGCTAGCACGCGTCAT

ACTCGATCCGCCGCGACGGTCTGCGCGCGACCGACCGCCATCAATATCACGAGTTGCTAA

GGTGTAGGACAAATCACCACCAAGGAGCTCGGCACAGTCATGCGGTCCCTTGGTCAAAAC

CCTTCCGAGTCCGAGCTGCAGGACATGATCAACGAGGTCGACGCCGACAACAACGGCACC

ATTGACTTTGGTGAGTCTAGATCCTCGTACATCCGCGCCCTCCACCGGAGGTGTCAAGAA

GCCTCACCGCTACAAGCCTGGTACCGTCGCTCTGCGTGAGATCCGTCGCTACCAGAAGAG

CACCGAGCTGCTGATCCGCAAGCTCCCCTTCCAGCGTCTGGTATGCAGGTCCGTGAGATC

GCCCAGGACTTCAAGTCCGACCTGCGCTTCCAGTCTTCCGCCATCGGTGCCCTGCAGGAG

TCCGTCGAGTCTTACCTCGTCTCCCTCTTTGAGGACACCAACCTGTGCGCCATCCACGCC

AAGCGTGTCACCATCCAGTCGGTACGTNNNNNNNNNNNNNNNNNNNNNNNNGCGGAGGGA

TCATTGCTGCTTC-GGCGCACCCAGAAACCCTTTGTGAACTTA--CTGTTGCCTCGGCAG

GCCGGAAGGCCCCCTGAGACAGGGAGCA-GCCCGCCGGCGGCCAACCAAACTCTTGTTTC

TACAGTGAATCTCTAAATGAATCAAAACTTTCAACAACGGATCTCTTGGTTCTGGCATCG

ATGAAGAACGCAGCGAAATGCGATAAGTAATGTGAATTGCAGAATTCAGTGAATCATCGA

ATCTTTGAACGCACATTGCGCCCTCTGGTATTCCGGAGGGCATGCCTGTTCGAGCGTCAT

TTCAACCCTCAAGCCTGGCTTGGTGATGGGGCACTGGGGAGCAGGCCCTGAAATCTAGTG

GCGAGCTCGCTAGGACCCCGAGCGTAGTAGA-TCTCGTTAAGGCCCTGGCGGTGCCCTGC

TGAAANAGAAGGAAGGTCAGTAAATACCGTTGTGTCTGCGACGCTCCGTCAAGGCATTTT

CACCCCTCCCTCTGGATTTTCAGGGTGCGGGGCTTACCGCTTATCTCAGTGAC-ACAC-C

AAAACATGCTGATTTCTACACAGCCGCCGAGCTGGGTAAGGGNNNNNNNNNNNNNNNNNN

NNNNNNNNNNNNNNNNNNNNNNNNNNNNNNNNNNNNNNNNNNNNNNNNNNNNNNNNNNNN

NNNNNNNNNNNNNNNNNNNNNNNNNNNNNNNNNNNNNNNNNNNNNNNNNNNNNNNNNNNN

NNNNNNNNNNNNNNNNNNNNNNNNNNNNNNNNNNNNNNNNNGTGCTGCTTTCTGGTGCGT

CCCAAGCCTACCACCGCGAGGCGAGCTCGAAGCATACTGACCTCGTAGGCAAACCATCTC

TGGCGAGCACGGTCTCGACAGCAATGGCGTGTATGCACCTCCTATCCCTGCCCGCCTCGT

CCTCCCTGATGATCGCACAGTTACAACGGCACTTCCGAGCTCCAGCTCGAGCGCATGAAC

GTCTACTTCAACGAGGTAAGTACGTCGTTTTGACACCTCTCGCCTTTGCTAACGCGTTTT

CAGGCCTCCGGCAACAAGTATGTGCCCCGCGCCGTCCTCGTCGATCTCGAGCCCGGTACC

ATGGACGCCGTC

>Diaporthe_longispora_CBS_194_36

NNNNNNNNNNNNNNNNNGCCTTGCCCGCCGTTGCTGTCGCGCATGCTAACGGACCGTTTT

CGGCCTGCAGGATAAGGATGGCGATGGTTAGTGTGGTCACTACTAGCTAGCATGCGTCAC

ACTCGATCCCCCGCGACGGTCTGCGCGCGAGCGACCGTCGTCACTGTCA-GGATTGCTAA

GGTGTAGGACAAATCACCACCAAGGAGCTCGGCACGGTCATGCGTTCCCTGGGTCAAAAC

CCCTCCGAGTCCGAGCTGCAGGATATGATCAACGAGGTCGACGCCGACAACAATGGCACC

ATTGACTTTGGTAAGTCTAGATGCTCGCCCATCCGCGCCCTCCACCGGAGGTGTCAAGAA

GCCTCACCGCTACAAGCCTGGTACCGTCGCTCTGCGTGAGATCCGTCGCTACCAGAAGAG

CACTGAGCTGCTGATCCGCAAGCTCCCCTTCCAGCGTCTGGTATGCAGGTCCGTGAGATC

GCCCAGGACTTCAAGTCCGACCTGCGCTTCCAGTCTTCTGCCATCGGTGCCCTGCAGGAG

TCTGTCGAGTCTTACCTCGTCTCTCTCTTCGAGGACACCAACCTGTGCGCCATCCATGCC

AAGCGTGTCACCATCCAGTCGGTACGTAACAAGGTCTCCGTTGGTGAACCAGCGGAGGGA

TCATTGCTGCTTC-GGCGCACCCAGAAACCCTTTGTGAACTCTA-CTGTTGCCTCGGCAG

GCCGGGGGGCCCCTCTTCCCGAGGAGCA-GCCCGCCGGCGGCCAACCAAACTCTTGTTTC

T-TAGTGAGTCTCTAAATAAATCAAAACTTTCAACAACGGATCTCTTGGTTCTGGCATCG

ATGAAGAACGCAGCGAAATGCGATAAGTAATGTGAATTGCAGAATTCAGTGAATCATCGA

ATCTTTGAACGCACATTGCGCCCTCTGGTATTCCGGAGGGCATGCCTGTTCGAGCGTCAT

TTCAACCCTCAAGCTCTGCTTGGTGATGGGGCACCGGAGGGCGGGCCCTGAAATCTAGTG

GCGAGCTCGCCAGGACCCCGAGCGTAGTAGA-TCTCGCCAAGGCCCTGGCGGTGCCCTGC

TGAAAGAGAAGGAAGGTCAGTAAACACCATTGCGTTTGCGCGGCTTCGTCCAGGCATTTT

CACCCCTCCCTCTGGATTTCTAGGGTGCGGGGCTTACGGCTTATCTTGCTGCCACCACCG

GAAAGAAGCTGATTTCTACACAGCCGCCGAGCTGGGTAAGGGTCGCACCTCAGCCCCACC

ATCGCGACCCGCCCCCTGCTGCACCCAGATAAGACACGTCGATTGCTAACATGTTTTTCT

CGCCCACAGGTTCACCTTCAGACCGGCCAATGCGTAAGTTGCTGTCACACCCGGACCTCA

T---CGCCACCTGTAGCACGTTTCCCAGGGTAACCAAATCGGTGCTGCTTTCTGGTGCGT

CCCAAGTCTGCCGCCGCGACACTAGCTCGCGACATACTGACCTCGTAGGCAAACCATCTC

TGGCGAGCACGGTCTCGACAGCAATGGCGTGTATGTACCTCTTATTCCTGCCTATCTCGT

CCTCCCTGACAATTGCACAGTTACAACGGCACTTCCGAGCTCCAGCTCGAGCGCATGAAC

GTCTACTTCAACGAGGTAAGTACGTCGTTTTGACCA--CACGGCCTTGCTAACGCGTTAT

CAGGCTTCCGGCAACAAGTATGTGCCTCGCGCCGTCCTCGTCGATCTCGAGCCCGGTACC

ATGGATGCCGTC

>Diaporthe_lonicerae_MFLUCC_17_0963

NNNNNNNNNNNNNNNNNGACATGCCCGACCTTGCTGGTGCGCATGCTAACGGACCGTTTT

CGGCTTGTAGGATAAGGACGGCGATGGTTAGTGCGGCCGCTCCCAGCTAGCACGCGTCAT

GTTCGATCCGCCGCGACAGCCTGCGCGCAACCAAGCGTTATCACTATCACGAGTTGCTGA

GGTGTAGGACAAATCACCACCAAGGAGCTCGGCACGGTCATGCGATCCCTGGGTCAGAAC

CCGTCCGAGTCTGAGCTGCAAGATATGATTAACGAGGTCGACGCCGACAACAATGGCACC

ATTGACTTTGGTACGTCCAGATGCTCGCGCT?????????????????????????????

????????????????????????????????????????????????????????????

????????????????????????????????????????????????????????????

????????????????????????????????????????????????????????????

????????????????????????????????????????????????????????????

???????????????????????????NNNNNNNNNNNNNNNNNNNNNNNNNNNNNNNNN

NNNNNNNNNNNNNNNNNNNNNNCAGAAACCCTTTGTGAACTTTTACTGTTGCCTCGGCTA

GCTGGGGGGCCCCTCCGGGTGTTGAGACAGCCCGTCGGCGGCCAACCTAACTCTTGTTTT

TACACTGAAACTCTAAATGAATCAAAACTTTCAACAACGGATCTCTTGGTTCTGGCATCG

ATGAAGAACGCAGCGAAATGCGATAAGTAATGTGAATTGCAGAATTCAGTGAATCATCGA

ATCTTTGAACGCACATTGCGCCCTCTGGTATTCCGGAGGGCATGCCTGTTCGAGCGTCAT

TTCAACCCTCAAGCCTGGCTTGGTGATGGGGCACTGAGAAGCAGGCCCTGAAATTCAGTG

GCGAGCTCGCCAGGACCCCGAGCGCAGTAGACCCTCGCTAAGGCCCTGGCGGTGCCCTGC

TGAAANNNNNNNNNNNNNNNNNNNNNNCATCATGCTCGCGCGGCCTCGCCAGCGCATTTT

CACCCCTCGCTTTGGATTTTCAGAGTGCGGGGCTTAGGGCTTATCTTGTCACCACCACCG

AATATATGCTGATATCTACACAGCCGCCGAGCTTGGTAAGGGNNNNNNNNNNNNNNNNNN

NNNNNNNNNNNNNNNNNNNNNNNNNNNNNNNNNNNNNNNNNNNNNNNNNNNNNNNNNNNN

NNNNNNNNNNNNNNNNNNNNNNNNNNNNNNNNNNNNNNNNNNNNNNNNNNNNNNNNNNNN

NNNNNNNNNNNNNNNNNNNNNNNNNNNNNNNNNNNNNNNNNNNNNNNNNNNNNNNNNNNN

NNNNNNNNNNNNGCCGCGATACGACCTCGAAGCATGCTGACCTCGTAGGCAAACCATCTC

TGGCGAGCACGGCCTCGACAGCAATGGCGTGTATGCACCTCCTATGCCTGTCCATCTTGA

CTTCCCTGACAATCGCACAGTTACAACGGCACTTCTGAGCTCCAGCTCGAGCGCATGAAC

GTCTACTTCAACGAGGTAAGTACAACATTCCGACCATCTCCAAGTTCGCTAACGCGTTAT

CAGGCCTCCGGCAACAAGTATGTTCCTCGCGCCGTCCTCGTCGATCTCGAGCCCGGTACC

ATGGACGCCGTC

>Diaporthe_lovelaceae_BRIP_60163a

????????????????????????????????????????????????????????????

????????????????????????????????????????????????????????????

????????????????????????????????????????????????????????????

????????????????????????????????????????????????????????????

????????????????????????????????????????????????????????????

????????????????????????????????????????????????????????????

????????????????????????????????????????????????????????????

????????????????????????????????????????????????????????????

????????????????????????????????????????????????????????????

????????????????????????????????????????????????????????????

???????????????????????????NNNNNNNNNNNNNNNNNNNNNNNNNNNNNNNNN

NNNNNNNNNCCCCAGGCGCACCCAGAAACCCTTTGTGAACTTTTACTGTTGCCTCGGTAC

GCTGGGGGGTCCCTCCCGGTGAGGAGCAGGCACGCCGGCGGCCAAGTTAACTCTTGTTTT

TACACTGAAACTCTAAATGAATCAAAACTTTCAACAACGGATCTCTTGGTTCTGGCATCG

ATGAAGAACGCAGCGAAATGCGATAAGTAATGTGAATTGCAGAATTCAGTGAATCATCGA

ATCTTTGAACGCACATTGCGCCCTCCGGTATTCCGGAGGGCATGCCTGTTCGAGCGTCAT

TTCAACCCTCAAGCCTGGCTTGGTGTTGGGGCACTGGGAAGCAGGCCCTGAAATCTAGTG

GCGAGCTCGCCAGGACCCCGAGCGTAGTAGACCCTCGCTAAGGCCCTGGCGGTGCCCTGC

GAAAANNNNAGGAAGGTTAGTAAACATCATGGTGTTCGTGTGGCTTCGTCAGCGCATTTT

CACCCCTCGCTCTGGATTTTCAGGGTGCGGGGCTTAGAGCTTATCTCACCACCACCAACG

AAAACATGCTAACATCTAAATAGCCGCCGAGCTTGGCAAGGGTCGCACCTGAACCCCACC

ATCGCGACCCACCCCCTGGGACACCCAGATAAGACGCGTCGATTGCTAACATGTTTTTCT

TGCTTATAGGTTCACCTCCAGACCGGCCAATGCGTAAGTTGCTGTCACACCGGA-CCGTA

TCATCGCCACCTGTAGCACGTTTCCCAGGGTAACCAAATCGGTGCTGCTTTCTGGTGCGT

CC-AAGCCCACCACTGTGATACGACCTCGAAGCATACTGACCTCGTAGGCAAACCATCTC

TGGCGAGCACGGCCTCGACAGCAATGGCGTGTATGCACCTCCTATTCCTGCCCATTTCGT

CCTCCCTGACAATCACACAGTTACAACGGCACTTCCGAGCTCCAGCTCGAGCGCATGAAC

GTCTACTTCAACGAGGTAAGTACGTCG--CTGACCATCTACAGCTTTGCTAACGCGTTAT

CAGGCCTCCGGCAACAAGTATGTTCCCCGCGCCGTCCTCGTCGATCTCGAGCCCGGTACC

ATGGACGCCGNN

>Diaporthe_lusitanicae_CBS_123212

CTTTGTAAGTTATTCTCGCCCTGCCCGCCATTGCCTTTGCGCATGCTAACGGACCGTTTT

CGGCCTGCAGGATAAGGATGGCGATGGTTAGTGCGGTCACTCTCAGCTACCACGCGTCAT

ACTCGATCCGCCTCGACGGTCTGCGCGTGAGCGAGCGACCTCATGACCACGAGATGCTAA

GGTGTAGGACAAATCACCACCAAGGAGCTCGGCACAGTTATGCGGTCCCTTGGTCAAAAC

CCTTCCGAGTCCGAGCTGCAGGACATGATCAACGAGGTCGACGCCGACAACAATGGCACC

ATTGACTTTGGTAAGTCTAGATGTCCACCCGTCCGCGCCCTCCACCGGAGGTGTCAAGAA

GCCTCACCGCTACAAGCCTGGTACCGTCGCTCTGCGTGAGATCCGTCGCTACCAGAAGAG

CACCGAGCTGCTGATCCGCAAGCTCCCCTTCCAGCGTCTGGTAAGCAGGTTCGTGAGATC

GCCCAGGACTTCAAGTCCGACCTGCGCTTCCAGTCTTCTGCCATTGGTGCCCTTCAGGAG

TCCGTCGAGTCTTACCTCGTCTCCCTTTTCGAGGACACCAACCTGTGCGCCATCCACGCC

AAGCGTGTCACCATCCAGTCGGTACGTAACAAGGTCTCCGTTGGTGAACCAGCGGAGGGA

TCATTGCTGCTTC-GGCGCACCCAGAAACCCTTTGTGAACTTA--CTGTTGCCTCGGCAG

GCCGGGAGGCCCCCCGAAAGGGGGAGCA-GCCCGCCGGCGGCCAACTAAACTC-TGTTTC

TATAGTGAATCTCTAAATGAATCAAAACTTTCAACAACGGATCTCTTGGTTCTGGCATCG

ATGAAGAACGCAGCGAAATGCGATAAGTAATGTGAATTGCAGAATTCAGTGAATCATCGA

ATCTTTGAACGCACATTGCGCCCTCTGGTATTCCGGAGGGCATGCCTGTTCGAGCGTCAT

TTCAACCCTCAAGCCTGGCTTGGTGATGGGGCACTGAAGGGCAGGCCCTGAAATCTAGTG

GCGAGCTCGCCAGGACCCCGAGCGTAGTAGA-TCTCGCTAAGGCCCTGGCGGTGCCCTGC

TGAAAGAGAAGGAAGGTCAGTAAACATCATTGTGCCTGCGCGGCTTCGT--AGGCATTTT

CACCCCTCCCTCTGGGTTTTCAGGGTGCGGGGCTTACCGCTTATCTCACCGCCAACACCG

AAAAGACGCTGATTTTCACCCAGCCGCCGAGCTGGGTAA-GGTCGCACCTCAGCCCCACC

ATCGCGACCCACCCCCTGCGACACCCAGATAAGACGCGTCGATTGCTAACATGTTTTTCT

CTCCTACAGGTTCACCTTCAGACCGGCCAATGCGTAAGTTGCTGTCACGCC-CGACCTTA

T---CGCCACCCGTAGCACGTTTCCCAGGGTAACCAAATCGGTGCTGCTTTCTGGTGCGT

CAAGCCGCCACGACCGCGAGACTGGCTCGCAATATACTGACCTCGTAGGCAAACCATCTC

TGGCGAGCACGGTCTCGACAGCAATGGCGTGTATGCACCTCCTATTCCTGCCCTTCTCAT

CCTCCCTGATGTTTGCACAGTTACAACGGCACTTCCGAGCTCCAGCTCGAGCGCATGAAC

GTCTACTTCAACGAGGTGAGTACGTCTTTTTGAGCGACCTCGACTTTGCTAACGCGCTTT

CAGGCCTCCGGCAACAAGTATGTGCCTCGCGCCGTCCTCGTCGATCTCGAGCCCGGTACC

ATGGACGCCGTC

>Diaporthe_lutescens_SAUCC194_36

NNNNNNNNNNNNNNGCCGATATGCCCGCCCTTGCTGTTGCGCATGCTAACGGACCGTTCT

CGGCTTGCAGGATAAGGATGGCGATGGTTAGTGCGGCCGGTCCCACATGACACGCGTCAT

GCTCGATCCGCCGCGATGGCCTGCGCGCATCCGAGCGTCACCACTACCACGAGTTGCTGA

GGTGTAGGACAAATCACCACCAAGGAGCTCGGCACAGTCATGCGGTCCCTGGGCCAGAAC

CCGTCCGAGTCTGAGCTGCAAGATATGATTAACGAGGTCGACGCCGACAACAATGGCACC

ATTGACTTTGGTATGTTCAGATGCTCACCCGTCCGCGCCCTCCACCGGAGGTGTCAAGAA

GCCTCACCGCTACAAGCCTGGTACCGTCGCTCTGCGTGAGATCCGTCGTTACCAGAAGAG

CACCGAGCTTCTGATCCGCAAGCTCCCCTTCCAGCGTCTGGTATGCAGGTTCGTGAGATC

GCCCAGGACTTCAAGTCCGACCTCCGCTTCCAGTCCTCCGCCATCGGTGCCCTGCAGGAG

TCCGTCGAGTCTTACCTCGTCTCCCTGTTCGAGGACACCAACCTGTGCGCCATCCACGCT

AAGCGTGTCACCATCCAGTCGGTACGTNNNNNNNTCTCCGTTGGTGAACCAGCGGAGGGA

TCATTGCTGCCCCTGGCGCACCCAGAAACCCTTTGTGAACTTTTACTGTTGCCTCGGCAG

GCCGTTGGGACCCTTGTGACAAGGAGCA-GCCGGCCGGTGGCCAAGTTAACTCTTG-TAT

TAATTTGTCTCTCTAAATGAATCAAAACTTTCAACAACGGATCTCTTGGTTCTGGCATCG

ATGAAGAACGCAGCGAAATGCGATAAGTAATGTGAATTGCAGAATTCAGTGAATCATCGA

ATCTTTGAACGCACATTGCGCCCTCTGGTATTCCGGAGGGCATGCCTGTTCGAGCGTCAT

TTCAACCCTCAAGCCTAGCTTGGTGTTGGGGCACTGAAGGGCAGGCCCTGAAATATAGTG

GCGAGCTCGCCAGGACTCCGAGCGTAGTAGACCCTCGCTAAGG-CCTGGCGGTGCCCTGC

GAAAANNNNNNNNNNNNNNNNNGCGTGTCCCCTGTTCTGGCGGCTTGGTCGGCGCATTTT

GACCCCTCCCTCTGGACTTTCAGGGTGCGGGGCTTAGAGCTTATCTCACCAATACGTCCG

AAAAAATGCTGACATCTTCACAGCCGCCGAGCTTNNNNNNNNNNNNNNNNNNNNNNNNNN

NNNNNNNNNNNNNNNNNNNNNNNNNNNNNNNNNNNNNNNNNNNNNNNNNNNNNNNNNNNN

NNNNNNNNNNNNNNNNNNNNNNNNNNNNNNNNNNNNNNNNNNNNNNNNNNNNNNNNNNNN

NNNNNNNNNNNNNNNNNNNNNNNNNNNNNNNNNNNNNNNNNNNNNNNNNNNNNNNNNNNN

NT-CAGTCCACCACCGCGACTCGACCCTGAAGCATACTGACCTCGCAGGCAAACCATCTC

TGGCGAGCACGGTCTCGACAGCAATGGCGTGTATGCACCTCCTATTACTGTCTTTCTCGT

-TACCCTGACAATCACATAGTTACAACGGCACTTCCGAGCTTCAGCTCGAGCGCATGAAC

GTCTACTTCAACGAGGTAAGTACGCTG--TTGACCACCTACAGCTTTACTGACGCGTTCT

CAGGCCTCCGGCAACAAGTATGTGCCCCGCGCCGTCCTCGTCGATCTCGAGCCCGGTACC

ATGGACGCCGTC

>Diaporthe_macadamiae_BRIP_66526

????????????????????????????????????????????????????????????

????????????????????????????????????????????????????????????

????????????????????????????????????????????????????????????

????????????????????????????????????????????????????????????

????????????????????????????????????????????????????????????

????????????????????????????????????????????????????????????

????????????????????????????????????????????????????????????

????????????????????????????????????????????????????????????

????????????????????????????????????????????????????????????

????????????????????????????????????????????????????????????

???????????????????????????AACAAGGTCTCCGTTGGTGAACCAGCGGAGGGA

TCATTGCTGCCCCTGGCGCACCCAGAAACCCTTTGTGAACTTTTACTGTTGCCTCGGCAG

GCCGTGGGGTCCCTT--AACAAGGAGCA-GCCGGCCGGTGGCCAAATTAACTC-TGTTTT

TACACTGAAACTCTAAATGAATCAAAACTTTCAACAACGGATCTCTTGGTTCTGGCATCG

ATGAAGAACGCAGCGAAATGCGATAAGTAATGTGAATTGCAGAATTCAGTGAATCATCGA

ATCTTTGAACGCACATTGCGCCCTCTGGTATTCCGGAGGGCATGCCTGTTCGAGCGTCAT

TTCAACCCTCAAGCCTGGCTTGGTGATGGGGCACTGAAGGGCAGGCCCTGAAATACAGTG

GCGAGCTCGCCAGGACTCCGAGCGTAGTAGACCCTCGCTAAGG-CCTGGCGGTGCCCTGC

TGAAAGAGAAGGAAGGTTAGTAAACACCATCATGTTCGTGCGGCTTCGTTGGCGCATTTT

CACCCCTCCCTCTGGATTTTCAGGGTGCGGGGCTTAGAGCTTATCTTACCACTACGTCCA

AAAATATGCTGACATCTACACAGCCGCCGAGCTTGGCAAGGGTCGCACCTGAGCCCCACC

ATCGCGACCCTCCCCCTGGGACACCCAGTTAAGACGCGTCGATTGCTAACATGTTTTTCT

CGCCCACAGGTTCACCTTCAGACCGGCCAATGCGTAAGTCA------CACCGGA-CCTTA

TCATCGCCACCTGTAGCACGTTTCCCAGGGTAACCAAATCGGTGCTGCTTTCTGGTGCGT

CC-AAGTCCACCGCCGCGAGTCGACCCCGAAGCATACTGACCTCGCAGGCAAACCATCTC

TGGCGAGCACGGTCTCGACAGCAATGGCGTGTATGCACCTCCTATTTCTGTCTTTCTCGT

-TGCCCTGACAATCACACAGTTACAACGGCACTTCCGAGCTCCAGCTCGAGCGCATGAAC

GTCTACTTCAACGAGGTAAGTACGTTG--TTGACCATCTAAAGCTCTGCTAATGCGTTAT

CAGGCCTCCGGCAACAAGTATGTGCCCCGCGCCGTCCTCGTCGATCTCGAGCCCGGTACC

ATGGACGCCGTC

>Diaporthe_machili_SAUCC194_111

NNNNNNNNNNNTGAATCGGCTTGCCCGCCACTGCTTTTGCGCATGCTAACGGACCGTTTT

CGGCCTGCAGGATAAGGATGGCGATGGTTAGTGTGGTCACTCTCAGCTAGCACGCGTCAT

ACTCGATCCGCCGCGACGGTCTGCGCGCGAGCGACCGTCATCACTATCACGAGTTGCTAA

GGTGTAGGACAAATTACCACCAAGGAGCTCGGCACAGTCATGCGGTCCCTTGGTCAAAAC

CCTTCCGAGTCCGAGCTGCAGGACATGATCAACGAGGTCGACGCCGACAACAATGGCACC

ATTGACTTTGGTAAGCCTGGATGCTTATCCCTCCGCGCCCTCCACCGGAGGTGTCAAGAA

GCCTCACCGCTACAAGCCTGGTACCGTCGCTCTGCGTGAGATCCGTCGCTACCAGAAGAG

CACCGAGCTGCTGATCCGCAAGCTCCCCTTCCAGCGTCTGGTATGCAGGTCCGTGAGATC

GCCCAGGACTTCAAGTCCGACCTGCGCTTCCAGTCTTCCGCCATCGGTGCCCTTCAGGAG

TCCGTCGAGTCTTACCTCGTCTCCCTCTTTGAGGACACCAACCTGTGCGCCATCCACGCC

AAGCGTGTCACCATCCAGTCGGTACGTNNNNNNNTCTCCGTTGGTGAACCAGCGGAGGGA

TCATTGCTGCTTC-GGCGCACCCAGAAACCCTTTGTGAACTTA--CTGTTGCCTCGGCAG

GCCGGGAGGCCCCCCGAGACGGGGAGCA-GCCCGCCGGCGGCCAACTAAACTCTTGTTTC

TATAGTGAATCTCTAAATGAATCAAAACTTTCAACAACGGATCTCTTGGTTCTGGCATCG

ATGAAGAACGCAGCGAAATGCGATAAGTAATGTGAATTGCAGAATTCAGTGAATCATCGA

ATCTTTGAACGCACATTGCGCCCCCTGGTATTCCGGGGGGCATGCCTGTTCGAGCGTCAT

TTCAACCCTCAAGCCTGGCTTGGTGATGGGGCACTGAGGAGCAGGCCCTGAAATTCAGTG

GCGAGCTCGCCAGGACCCCGAGCGTAGTAGA-TCTCGCTAAGGCCCTGGCGGTGCCCTGC

TGAAANNNNNNNNNNNNNNNGATTCTTCATGGTTTTTGCACGGCTGCGTCAAGCCATTTT

CGCCCCTCCCTCTGGATTTTCAGGGTGCGGGGCTTACGGCTTATCTCACTGCCTGCTCCG

ACAACATGCTGAATCCCACACAGCCGCCGAGCTGNNNNNNNNNNNNNNNNNNNNNNNNNN

NNNNNNNNNNNNNNNNNNNNNNNNNNNNNNNNNNNNNNNNNNNNNNNNNNNNNNNNNNNN

NNNNNNNNNNNNNNNNNNNNNNNNNNNNNNNNNNNNNNNNNNNNNNNNNNNNNNNNNNNN

NNNNNNNNNNNNNNNNNNNNNNNNNNNNNNNNNNNNNNNNNNNNNNNNNNNNNNNNNNNN

NTCGAGCCTGCCACCGCGAGACTAGCTCGCAACATACTGACCTCGTAGGCAAACCATCTC

TGGCGAGCACGGTCTCGACAGCAATGGCGTGTATGTACCTCCTATTCCTGCCCATCTCGT

CCTCCCTGATGATCGCACAGTTACAACGGCACTTCCGAGCTCCAGCTCGAGCGCATGAAC

GTCTACTTCAACGAGGTAAGCACGGCTCTTTGA-CCGTCTCGGCCTTGCTAATGCGCTCT

CAGGCCTCCGGCAACAAGTATGTGCCTCGCGCCGTCCTCGTCGATCTCGAGCCCGGTACC

ATGGACGCCGTC

>Diaporthe_macintoshii_BRIP_55064a

????????????????????????????????????????????????????????????

????????????????????????????????????????????????????????????

????????????????????????????????????????????????????????????

????????????????????????????????????????????????????????????

????????????????????????????????????????????????????????????

????????????????????????????????????????????????????????????

????????????????????????????????????????????????????????????

????????????????????????????????????????????????????????????

????????????????????????????????????????????????????????????

????????????????????????????????????????????????????????????

???????????????????????????AACAAGGTCTCCGTTGGTGAACCAGCGGAGGGA

TCATTGCTGCCCCTGGCGCACCCAGAAACCCTTTGTGAACTTTTACTGTTGCCTCGGCAG

GCCGTGGGGTCCCTTAGACAAAGGAGCA-GCCGGCCGGTGGCCAAGTTAACTC-TGTTTT

TAAACTGAAACTCTAAATGAATCAAAACTTTCAACAACGGATCTCTTGGTTCTGGCATCG

ATGAAGAACGCAGCGAAATGCGATAAGTAATGTGAATTGCAGAATTCAGTGAATCATCGA

ATCTTTGAACGCACATTGCGCCCTCTGGTATTCCGGAGGGCATGCCTGTTCGAGCGTCAT

TTCAACCCTCAAGCCTGGCTTGGTGATGGGGCACTGAAGGGCAGGCCCTGAAATATAGTG

GCGAGCTCGCCAGGACTCCGAGCGCAGTAGACCCTCGCTAAGG-CCTGGCGGTGCCCTGC

TGAAANNGAAGGAAGGTCAGTAAACATCATCATGCTC--GTGGCTTCATCGGCGCATTTT

GACCCCTCCCTCTGGATTTTCAGGGTGCGGGGCTTAGAGCTTATCTCACC---------A

GTACCATGCTGATATCTATACAGCCGCTGAGCTTGGCAAGGGTCGCACCTGAGCCCCACC

ATCGCGACCCACCCCCTGGGACACTCAGATAAGACGCGTCGACTGCTAACATGTTTTTCT

CGCCCACAGGTTCACCTTCAGACCGGCCAATGCGTAAGTTGCTGTCACACCGGA-CCTTA

TCATCGCCACCCGTAGCACGTTTCCCAGGGTAACCAAATCGGTGCTGCTTTCTGGTGCGT

CC-AAGCCCACCTCCGCGATACGACCTCGAAGCATACTGACCTCGCAGGCAAACCATCTC

TGGCGAGCACGGTCTCGACAGCAATGGCGTGTATGCACCTCCTATTTCTGTCTTTCTCGT

-TGCCCTGACAATCACACAGTTACAACGGCACTTCCGAGCTCCAGCTCGAGCGCATGAAC

GTCTACTTCAACGAGGTAAGTACGTTG--TTGACCACCCACAGCTTGGCTAACGCGTTAC

CAGGCCTCCGGCAACAAGTATGTGCCCCGCGCCGTCCTCGTCGATCTCGAGCCCGGTACC

ATGGACGCCGTC

>Diaporthe_eres_CGMCC_3_15181

????????????????????????????????????????????????????????????

????????????????????????????????????????????????????????????

????????????????????????????????????????????????????????????

????????????????????????????????????????????????????????????

????????????????????????????????????????????????????????????

????????????????????????????????????????????????????????????

????????????????????????????????????????????????????????????

????????????????????????????????????????????????????????????

????????????????????????????????????????????????????????????

????????????????????????????????????????????????????????????

???????????????????????????NNNNNNNNNNNNNNNNNNNNNNNNNNNNAGGGA

TCATTGCTGCCCCAGGCGCACCCAGAAACCCTTTGTGAACTTTTACTGTTGCCTCGGCAT

GCCGGGGGGCCCCTCTCCTGGAGGAGCAGGCACGCCGGCGGCCAACCTAACTCTTGTTTT

TACACTGAAACTCTAAATGAATCAAAACTTTCAACAACGGATCTCTTGGTTCTGGCATCG

ATGAAGAACGCAGCGAAATGCGATAAGTAATGTGAATTGCAGAATTCAGTGAATCATCGA

ATCTTTGAACGCACATTGCGCCCTCTGGTATTCCGGAGGGCATGCCTGTTCGAGCGTCAT

TTCAACCCTCAAGCCTGGCTTGGTGATGGGGCACTGAAGGGCAGGCCCTGAAATTCAGTG

GCGAGCTCGCCAGGACCCCGAGCGCAGTAGACCCTCGCTAAGGCCCTGGCGGTGCCCTGC

TGAAANNNNNNNNNNNNNNNNNNNNNNCATCATGCTCGCGCGGCCTCGCCAGCGCATTTT

CACCCCTCGCTTTGGATTTTCAGAGTGCGGGGCTTAGGGCTTATCTTGTCACCACCACCG

AATATATGCTGATATCTACACAGCCGCCGAGCTTGGTAAGGG??????????????????

????????????????????????????????????????????????????????????

????????????????????????????????????????????????????????????

????????????????????????????????????????????????????????????

????????????????????????????????????????????????????????????

????????????????????????????????????????????????????????????

????????????????????????????????????????????????????????????

????????????????????????????????????????????????????????????

????????????????????????????????????????????????????????????

????????????

>Diaporthe_malorum_CBS142383

CTTTGTAAGTTATCTCCGCCTTGCCCGCCGTTGCTTTTGCGCATGCTAACGGCCCGTTTT

CGGCTTGCAGGATAAGGATGGCGATGGTTAGTGCCGCCACTCCCAACTAGCACGCGTCGC

GCTCGATCCGCCGCGACGGTCTGCGCGCAGGCGACCGTCATCACTATCACGGGTAGCTAA

GGTCTAGGGCAAATCACCACCAAGGAGCTCGGCACGGTCATGCGGTCCCTGGGCCAAAAC

CCCTCCGAGTCCGAGCTGCAGGATATGATCAACGAGGTCGACGCCGACAACAATGGCACC

ATTGACTTTGGTAAGTCCAGATCCTCGCCCATCCGCGCCCTCCACCGGAGGTGTCAAGAA

GCCTCACCGCTACAAGCCTGGTACCGTCGCTCTGCGTGAGATCCGTCGCTACCAGAAGAG

CACCGAGCTGCTGATCCGCAAGCTCCCCTTCCAGCGTCTGGTATGCAGGTTCGTGAGATC

GCCCAGGACTTCAAGTCCGACCTGCGCTTCCAGTCTTCCGCCATCGGTGCCCTGCAGGAG

TCCGTCGAGTCTTACCTCGTCTCCCTCTTCGAGGACACCAACCTGTGCGCCATCCACGCC

AAGCGTGTCACCATCCAGTCGGTACGTNNNNNNNNNNNNNNNNNNNNNNNNNNNNAGGGA

TCATTGCTGCCTC-GGCGCACCCAGAAACCCTTTGTGAACTTTA-CTGTTGCCTCGGCAG

GCCGGGAAGCCCCTCGTAACGAGGAGCA-GCCCGCCGGCGGCCAAGTTAACTCTTGTTTT

TACACTGAAACTCTAAATGAATCAAAACTTTCAACAACGGATCTCTTGGTTCTGGCATCG

ATGAAGAACGCAGCGAAATGCGATAAGTAATGTGAATTGCAGAATTCAGTGAATCATCGA

ATCTTTGAACGCACATTGCGCCCTCTGGTATTCCGGAGGGCATGCCTGTTCGAGCGTCAT

TTCAACCCTCAAGCCTGGCTTGGTGATGGGGCACTGAGGAGCAGGCCCTGAAATCTAGTG

GCGAGCTCGCCAGGACCCCGAGCGCAGTAGACCCTCGCTAAGGCCCTGGCGGTGCCCTGC

TGAAANNNNNNNNAGGTTAGTAAAAATCGTCACTGTTGCGCCGCATCGTCAGCTCATTTT

GACCCCTCCCTCTGGATTTTCAGGGTGCGGGGCTTA-GGCTTATCTCGCTGCCAGCACCC

GAAACATGCTGATCTCTTCACAGCCGCCGAGCTTNNNNNNNNNNNNNNNNNNNNNNNNNN

NNNNNNNNNNNNNNNNNNNNNNNNNNNNNNNNNNNNNNNNNNNNNNNNNNNNNNNNNNNN

NNNNNNNNNNNNNNNNNNNNNNNNNNNNNNNNNNNNNNNNNNNNNNNNNNNNNNNNNNNN

NNNNNNNNNNNNNNNNNNNNNNNNNNNNNNNNNNNNNNNNNNNNNNNNNNNNTGGTGCGT

CCCGAGTCGAGCACCGCGATACGAGCTCGAAGCATACTGACCTTCTAGGCAAACCATCTC

TGGCGAGCACGGTCTCGACAGCAATGGCGTGTATGCACCTCCTATCCCTGCCCGTCTTGT

CCTCCCTGACAATTGGACAGTTACAACGGCACTTCCGAGCTCCAGCTCGAGCGCATGAAC

GTCTACTTCAACGAGGTAAGTACGTCGTTTTGACCATCTGCGGCCTTGCTAACGCGTTAT

CAGGCTTCCGGCAACAAGTATGTGCCTCGCGCCGTCCTCGTCGATCTCGAGCCCGGTACC

ATGGATGCCGTC

>Diaporthe_manihotia_CBS_505_76

CTTTGTAAGTCATATCCGCCTCGCCTGTCGTTGCTGTTGCGCATGCTAACGGACCGTTCT

CGGCCTGCAGGATAAGGATGGCGATGGTTAGTGTGGTCACCCTAAGCCAGCACGCGTCAT

GCTCGATCCGCCGCGACGGTCTGCGCGCGAGCGACCGTCATCACTATCACGAGTTGCTAA

GGTGCAGGACAAATCACCACCAAGGAGCTCGGCACGGTCATGCGGTCCCTGGGCCAAAAC

CCCTCCGAGTCCGAGCTGCAGGACATGATCAACGAGGTCGACGCCGACAACAACGGCACC

ATTGACTTTGGTAGGTTCACATGTCCACCCATCCGCGCCCTCCACCGGAGGTGTCAAGAA

GCCTCACCGCTACAAGCCTGGTACCGTCGCTCTGCGTGAGATCCGTCGCTACCAGAAGAG

CACCGAGCTGCTCATCCGCAAGCTCCCCTTCCAGCGTCTGGTATGCAGGTTCGTGAGATC

GCCCAGGACTTCAAGTCCGACCTGCGCTTCCAGTCTTCCGCCATCGGTGCTCTCCAGGAG

TCCGTCGAGTCTTACCTCGTCTCCCTCTTTGAGGACACCAACCTGTGCGCCATCCACGCC

AAGCGTGTCACCATCCAGTCGGTACGTNNNNNNNNNNNNNNNNNNNNNNNNGCGGAGGGA

TCATTGCTGCTTC-GGCGCACCCAGAAACCCTTTGTGAACTTA--CTGTTGCCTCGGCAG

GCCGGCAGACCCCCTGAAACAGGGAGCA-GCCCGCCGGCGGCCAACTAAACTC-TGTTTC

TATAGTGAATCTCTAAATGAATCAAAACTTTCAACAACGGATCTCTTGGTTCTGGCATCG

ATGAAGAACGCAGCGAAATGCGATAAGTAATGTGAATTGCAGAATTCAGTGAATCATCGA

ATCTTTGAACGCACATTGCGCCCTCTGGTATTCCGGAGGGCATGCCTGTTCGAGCGTCAT

TTCAACCCTCAAGCCTGGCTTGGTGATGGGGCAGTGCAGAGCACGCCCTGAAATTCAGTG

GCGAGCTCGCCAGGACCCCGAGCGTAGTAGA-TCTCGCTAAGGCCCTGGCGGTGCCCTGC

TGAAAGAGAAGGAAGGTCAGTAAACACCATTGAGCCTGCGCGGCTTCGT--CAGCATTTT

CACCCCTCGTTCTGGACTTTCAGGGTGCGGGGCTTACCGCTTATCGCGCTGCCCACCACG

AAAACATGCTGATTTCCATACAGCCGCCGAGCTGGGTAAGGGTCGCACCTCAGCCCCACC

ATCGCGACCCTCCCCTTGCGACACCCGCGTAACACGCGTCGATTGCTAACATGTTTTTCT

CGCCTGCAGGTTCACCTCCAGACCGGCCAATGCGTAAGTTGCTGTCACCGCCCGACCTTA

T---CGCCACCCGTAGCACGTTTCCCAGGGTAACCAAATCGGTGCTGCTTTCTGGTGCGT

CCGAGGCCTACCGCCGCGAGACCAGCTCGCAACATACTGACCTCGTAGGCAAACCATCTC

TGGCGAGCACGGCCTCGACAGCAATGGCGTGTATGCACCTCCTATTCCTGCCCATCTCGT

CCTCCCTGATGATCGCACAGTTACAACGGCACTTCTGAGCTCCAGCTCGAGCGCATGAAC

GTCTACTTCAACGAGGTATGTACGTCGTTTTGCCCATCTGCTGCCTTGCTAACGCGTTAT

CAGGCTTCCGGCAACAAGTATGTGCCCCGCGCTGTCCTCGTCGATCTCGAGCCCGGTACC

ATGGACGCCGTC

>Diaporthe_marina_MFLU_17_2622

????????????????????????????????????????????????????????????

????????????????????????????????????????????????????????????

????????????????????????????????????????????????????????????

????????????????????????????????????????????????????????????

????????????????????????????????????????????????????????????

????????????????????????????????????????????????????????????

????????????????????????????????????????????????????????????

????????????????????????????????????????????????????????????

????????????????????????????????????????????????????????????

????????????????????????????????????????????????????????????

???????????????????????????NNNNNNNNNNNNNNNNNNNNNACAGCGGAGGGA

-CATTGCTGCCCC-GGCGCACCCAGAAACCCTTTGTGAACTT--TTAGTTGCCTCGGTCG

GCGGGGGGGCCCCCC--------------GCCCGCCGGCGGCCAAATTAACTCTTGTTTT

T--CCTGAAACTCTAAATGAATCAAAACTTTCAACAACGGATCTCTTGGTTCTGGCATCG

ATGAAGAACGCGGCGAAATGCGATAAGTAATGTGAATTGCAGAATTCAGTGAGTCATCGA

ATCTTTGAACGCACATTGCGCCCTCTGGTATTCCGGAGGGCATGCCTGTTCGAGCGTCAT

TTCAACCCTCAAGCCTGGCCTGGTGTTGGGGCACTGCGAGGGAGGCCCTGAAATTCAGTG

GCGAGCTCCCCAGGACCCCGAGCGCAGTAGACCCTCGCTAAGGCCCTGGCGGTGCCCTGC

TGGAA???????????????????????????????????????????????????????

????????????????????????????????????????????????????????????

????????????????????????????????????????????????????????????

????????????????????????????????????????????????????????????

????????????????????????????????????????????????????????????

????????????????????????????????????????????????????????????

????????????????????????????????????????????????????????????

????????????????????????????????????????????????????????????

????????????????????????????????????????????????????????????

????????????????????????????????????????????????????????????

????????????????????????????????????????????????????????????

????????????

>Diaporthe_maritima_DAOMC_250563

????????????????????????????????????????????????????????????

????????????????????????????????????????????????????????????

????????????????????????????????????????????????????????????

????????????????????????????????????????????????????????????

????????????????????????????????????????????????????????????

????????????????????????????????????????????????????????????

????????????????????????????????????????????????????????????

????????????????????????????????????????????????????????????

????????????????????????????????????????????????????????????

????????????????????????????????????????????????????????????

???????????????????????????NNNNNNNNNNNNNNNNNNNNNNNNNNNNNNNNN

NCATTGCTGCCCCAGGCGCACCCAGAAACCCTTTGTGAACTTTTACTGTTGCCTCGGCTA

GCTGGGGGGCCCCTCCGGGTGTTGAGACGGCCCGCCGGCGGCCAACCTAACTCTTGTTTT

TACACTGAAACTCTAAATGAATCAAAACTTTCAACAACGGATCTCTTGGTTCTGGCATCG

ATGAAGAACGCAGCGAAATGCGATAAGTAATGTGAATTGCAGAATTCAGTGAATCATCGA

ATCTTTGAACGCACATTGCGCCCTCTGGTATTCCGGAGGGCATGCCTGTTCGAGCGTCAT

TTCAACCCTCAAGCCTGGCTTGGTGATGGGGCACTGAAAAGCAGGCCCTGAAATTCAGTG

GCGAGCTCGCCAGGACCCCGAGCGCAGTAGACCCTCGCTAAGGCCCTGGCGGTGCCCTGC

TGAAAGAGAAGGAAGGTTAGTAAATATCATCATGCTCGCGCGGCCTCGCCAGCGCATTTT

CACCCCTCGCTTTGGATTTTCAGAGTGCGGGGCTTAGGGCTTATCTTGTCACCACCACCG

AATATATGCTGATATCTTCACAGCCGCCGAGCTTGGTAAGGGNNNNNNNNNNNNNNNNNN

NNNNNNNNNNNNNNNNNNNNNNNNNNNNNNNNNNNNNNNNNNNNNNNNNNNNNNNNNNNN

NNNNNNNNNNNNNNNNNNNNNNNNNNNNNNNNNNNNNNNNNNNNNNNNNNNNNNNNNNNN

NNNNNNNNNNNNNNNNNNNNNNNNNNNNNNNNNNNNNNNNGGTGCTGCTTTCTGGTGCGT

TCCAAGTCCACCGCCGCGATACGACCTCGAAGCATGCTGACCTCGTAGGCAAACCATCTC

TGGCGAGCACGGCCTCGACAGCAATGGCGTGTATGCACCTCCTATTCCTGCCCATCTTGG

CTTCCCTGACAATCGCATAGTTACAACGGCACTTCTGAGCTCCAGCTCGAGCGCATGAAC

GTCTACTTCAACGAGGTAAGTACATCATTCCGACCATCTCCAACCTCGCTAACGTGTTAT

CAGGCCTCCGGCAACAAGTATGTTCCTCGCGCCGTCCTCGTCGATCTCGAGCCCGGTACC

ATGGACGCCGTC

>Diaporthe_masirevicii_BRIP_57892a

????????????????????????????????????????????????????????????

????????????????????????????????????????????????????????????

????????????????????????????????????????????????????????????

????????????????????????????????????????????????????????????

????????????????????????????????????????????????????????????

????????????????????????????????????????????????????????????

????????????????????????????????????????????????????????????

????????????????????????????????????????????????????????????

????????????????????????????????????????????????????????????

????????????????????????????????????????????????????????????

???????????????????????????AACAAGGTCTCCGTTGGTGAACCAGCGGAGGGA
[truncated: 738,091 more chars]
